# Catalytic Atroposelective Aerobic Oxidation Approaches to Axially Chiral Molecules

**DOI:** 10.1021/acs.joc.3c00417

**Published:** 2023-06-16

**Authors:** Lenin Kumar Verdhi, Asit Ghosh, Natalia Fridman, Alex M. Szpilman

**Affiliations:** † The Department of Chemical Sciences, Ariel University, Ariel 4070000, Israel; ‡ Schulich Faculty of Chemistry, Technion, Haifa 3200009, Israel

## Abstract

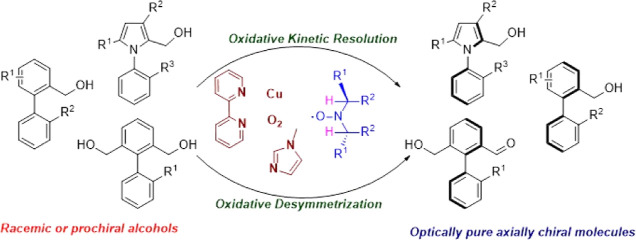

A copper and chiral nitroxide co-catalyzed aerobic enantioselective oxidation process has been developed that allows access to axially chiral molecules. Two complementary atroposelective approaches, oxidative kinetic resolution (OKR) and desymmetrization, were studied using ambient air as the stoichiometric terminal oxidant. OKR of *rac*-*N*-arylpyrrole alcohols and *rac*-biaryl alcohols affords the optically pure compounds with *er* up to 3.5:96.5 and 5.5:94.5, respectively. Desymmetrization of prochiral diols provides axially chiral biaryl compounds with *er* up to 99:1.

## Introduction

**Figure 1:**
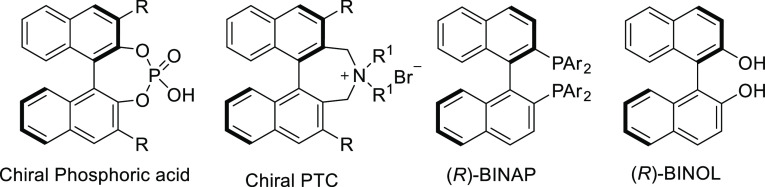
Selected privileged chiral catalyst and ligand scaffolds with axially chiral biaryl frameworks.

Axially chiral molecules are prevalent structural motifs in a wide range of natural products, pharmaceuticals, and functional materials.^1^ In addition, a plethora of chiral catalysts and privileged chiral ligands^1f^ have been developed based on axially chiral biaryl frameworks (Figure 1).^2^

**Scheme 1:**
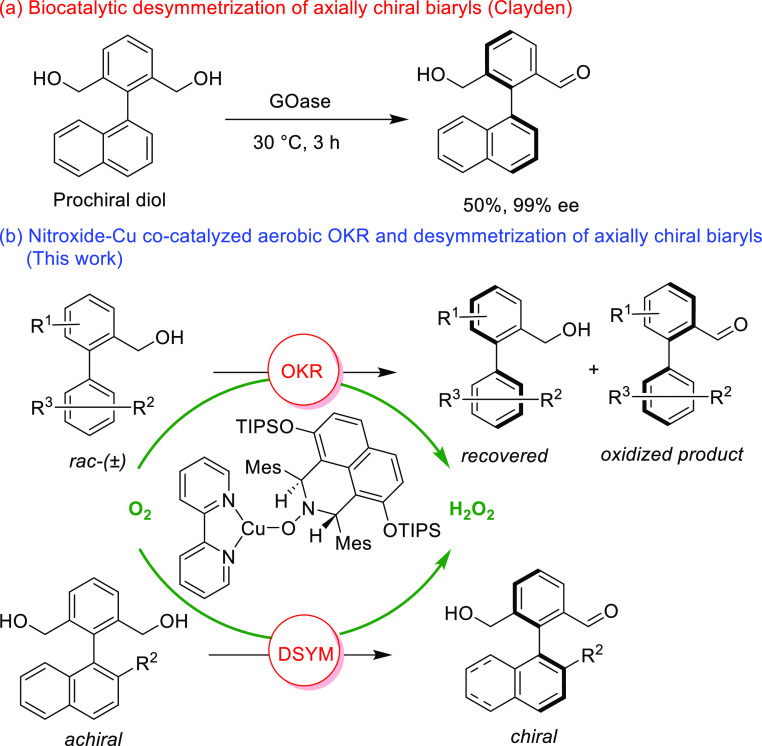
Oxidative Desymmetrization and Kinetic Resolution Approaches for the Synthesis of Atropisomers

Consequently, vast efforts have been made by the chemical community to prepare these atropisomers in an efficient and stereoselective manner.^3^ One intriguing methodology is the use of enzymes for the atroposelective desymmetrization.^4^ Clayden and Turner’s groups successfully demonstrated the ability of a copper-containing enzyme, a variant of galactose oxidase (GOase M_3–5_) for oxidative desymmetrization of prochiral substrates containing a pair of enantiotopic hydroxymethyl groups to access axially chiral compounds with up to 99% *ee* (Scheme 1a).^5^ A complementary approach using man-made chiral catalysts would be of high interest.

Semmelhack pioneered the use of atmospheric air as an oxidant in copper-nitroxide-catalyzed aerobic oxidation of alcohols to the corresponding carbonyl compounds.^6^ Based on this work, Stahl has developed several highly efficient nitroxide copper co-catalytic systems for efficient aerobic alcohol oxidation.^7^ The Stahl group^8^ and we^9^ also extensively studied the mechanism of this reaction. Interestingly, enantioselective oxidations using atmospheric oxygen as a terminal oxidant are quite rare.^10,11^

**Scheme 2:**
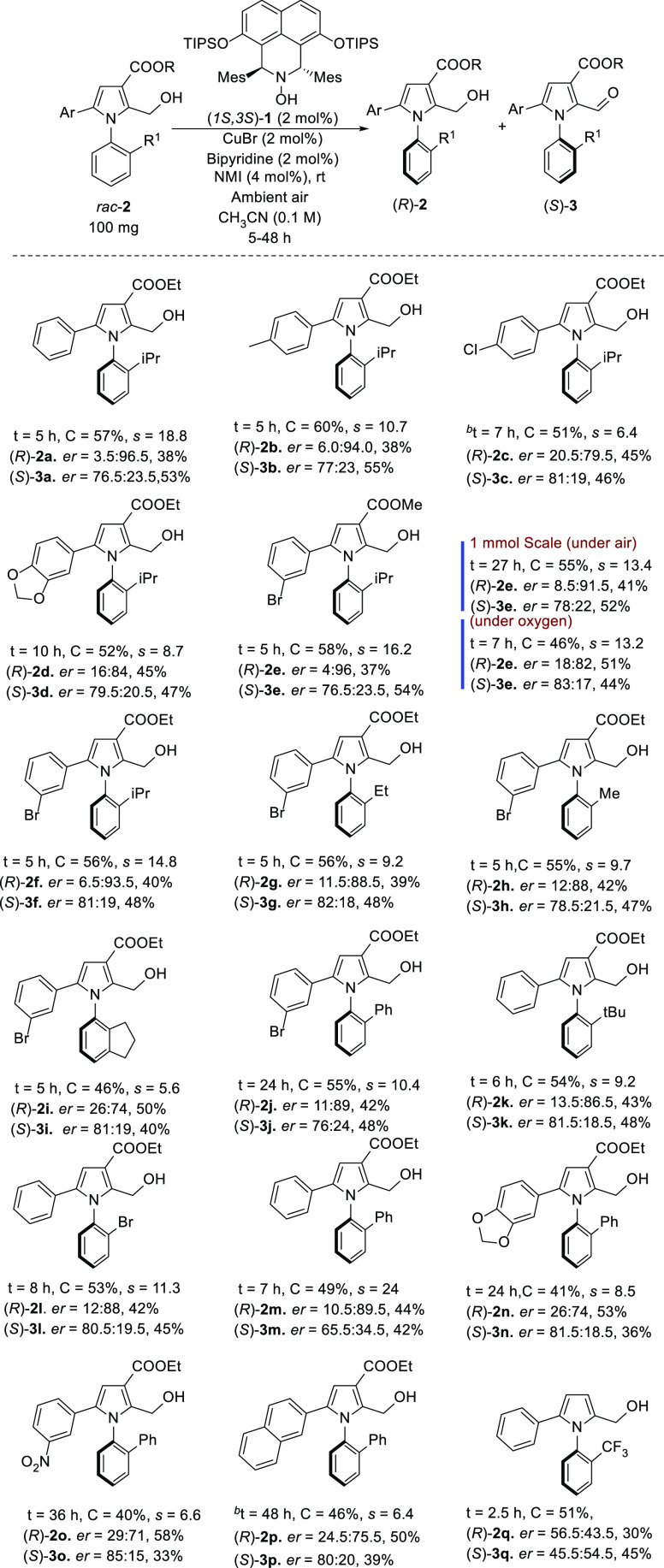
OKR of *rac*-*N*-Arylpyrroles to Access Axially Chiral *N*-Arylpyrroles Unless otherwise stated, reactions were carried out with 100 mg of **2**, (1*S*,3*S*)-**1** (2 mol %), Cu(I)Br (2 mol %), bipyridine (2 mol %), and *N*-methyl imidazole (4 mol %) in CH_3_CN (0.1 M). *er* values were determined by chiral HPLC. In all cases, 10 mg of product **3** was reduced to the corresponding alcohol prior to determining *er*. The selectivity factor (*s*) was calculated by *s* = ln[(1 – *C*) (1 – *ee*)]/ln[(1 – *C*) (1 + *ee*)]. *C* represents conversion (by ^1^H NMR analysis). *ee* = *ee* of the recovered alcohol. ^b^Reaction was carried out in a mixture of CH_3_CN–EtOAc (1:1) (0.05 M) mixture.

While nitroxides are outstanding catalysts and reagents for chemical synthesis^12^ and even have seen use in enantioselective oxidation of alcohols,^13^ their use in atroposelective oxidative kinetic resolution (OKR) and desymmetrization is still in infancy with the work by Blandin being the first example.^14^ As part of our longstanding interest in nitroxides,^9,15^ we recently reported our preliminary findings^16^ on chiral nitroxide-copper co-catalyzed aerobic OKR of axially chiral *N*-aryl-pyrroles (Scheme 1b) using readily available (prepared in only five steps)^16^ chiral α-hydrogen pre-catalyst **1** (97% *ee*, see Scheme 2 for the structure). In this full paper, we now report our complete findings on the OKR of biaryl systems and explore the use of this catalyst system in atroposelective desymmetrization reactions as well (Scheme 1b)

## Results and Discussion

Our reaction protocol closely followed the conditions we had developed earlier^15e,16^ which in turn were based on Stahl’s conditions.^7^ Significantly, these exact conditions are compatible with various functional groups that are sensitive to oxidation including alkenes, alkynes, thiophenes, and electron-rich aromatic systems,^15e^ see also ref (7). Thus, very little optimization work was needed apart from determining that 2 mol % of nitroxide and copper pre-catalyst were needed for efficient reactions. At lower catalyst loadings, the reaction was arrested, while at higher catalyst loadings, there was no change in stereoselectivity. Accordingly, as depicted in Scheme 2, the optimized OKR process requires only 2 mol % of the chiral hydroxyl amine precatalyst (1*S*,3*S*)-**1**. 2 mol % of inexpensive copper(I) bromide, 2 mol % of bipyridine, and 4 mol % of *N*-methyl imidazole were used as additional catalytic components. Importantly, ambient air was used as the oxygen source in the acetonitrile solvent at room temperature. Under these catalytic conditions, the chiral hydroxylamine precatalyst (1*S*,3*S*)-**1** oxidizes to generate chiral nitroxide radical, which participates in the catalytic process. Subjecting the *N*-arylpyrrole *rac*-**2a** to the OKR conditions for 5 h afforded the recovered alcohol (*R*)-**2a** in 38% isolated yield with 3.5:96.5 *er* at 57% conversion (C), as determined by HPLC (see the Experimental Section and Supporting Information for details). The oxidized product aldehyde (*S*)-**3a** was obtained in 53% yield with 76.5:23.5 *er*. This corresponds to a selectivity factor (s) of 18.8 (Scheme 2). It should be noted that axially chiral *N*-phenylpyrroles are important structural components of pharmaceuticals and natural products. Also, axially chiral pyrroles can be employed as ligands and catalysts in asymmetric catalysis.^17^ The methyl congener *rac*-**2b** was resolved with an s factor of 10.7 at 60% conversion with the unreacted alcohol (*R*)-**2b** isolated in 38% and 6.0:94.0 *er*. The aldehyde (*S*)-**3b** was isolated in 55% yield and like **3a** in a lower *er* of 77:23. The chloro-analogue *rac*-**2c** of **2b** was resolved with a *s* value of 6.4 at 51% conversion. However, this may be due to a solvent effect and not necessarily an electronic effect as the OKR of substrates **2c** (and **2p** see below) was carried out in a solvent mixture of acetonitrile and ethyl acetate (1:1) due to the poor solubility of the substrates. The acetal protected catechol-substituted *N*-arylpyrrole **2d** was resolved with an s value of 8.7, affording the resolved alcohol (*R*)-**2d** in 45% yield and an *er* of 16:84 and the oxidized product (*S*)-**3d** with an *er* of 79.5:20.5 and 47% yield based on 52% conversion. The 3-bromophenyl-substituted *rac*-**2e** was resolved in acetonitrile with an *s*-value of 16.2 to give the recovered (*R*)-**2e** in 37% with an *er* of 4:96, and the corresponding aldehyde (*S*)-**3e** was isolated in 54% yield with an *er* of 76.5:23.5. The conversion was 58% after 5 h. The bromo-substituent is of course useful for further functionalization. The resolution of **2e** was scaled up to 1 mmol. At this scale, the reaction time required to reach 55% conversion increased to 27 h under air with an *s*-value of 13.4.

**Figure 2:**
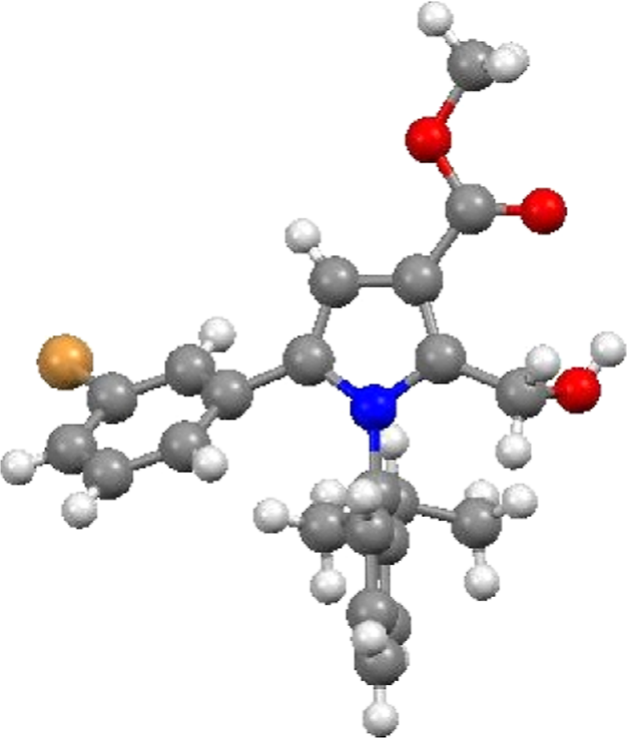
X-ray structure of the crystals of (*R*)-**2e** showing the absolute configuration, as determined by X-ray crystallography.

This is not surprising for a heterogeneous reaction, in which oxygen solubility in the organic solvent is in fact the rate-determining step. Indeed, **2e** could be resolved at the same 1 mmol scale under a pure oxygen atmosphere with a reduced reaction time of 7 h at 46% conversion with an *s*-value of 13.2. The corresponding ethyl ester *rac*-**2f** was resolved with an *s*-value of 14.8 at 56% conversion after 5 h. The absolute configuration of resolved (*R*)-**2e** was determined by X-ray crystallography (Figure 2) and the absolute configuration of all other products in Scheme 2 assigned accordingly.

We next examined the effect of the 2-substituent of the phenyl moiety of the *N*-aryl-pyrrole. As shown for compounds **2a–2f**, alcohols with an isopropyl substituent generally gave high *s*-values. In comparison, the 2-ethyl substituted in *rac*-**2g** was resolved with an *s*-value of 9.2 at 56% conversion after 5 h compared to 14.8 for isopropyl-substituted **2f** at the same conversion and reaction time. There was no significant difference between **2g** and 2-methyl-substituted **2h** which was resolved with an *s*-value of 9.7 at 55% conversion after 5 h. Adding a five-membered ring between the 2 and 3 positions on the phenyl moiety as in **2i** also led to a drop in the *s*-value to 5.6 and slightly retarded the reaction rate, so after 5 h, 46% conversion was achieved. 2-Phenyl-substituted **2j** was resolved with an *s*-value of 10.4 at 55% conversion after 24 h similar to what was achieved for 2-ethyl-substituted **2g**. This indicates that steric rather than electronic effect plays a role in the atroposelective oxidation step in this part of the substrate.

For comparison with *rac*-**2a**, we also tested **2k** with a sterically bulky 2-*tert*-butyl group. Resolution of *rac*-**2k** afforded the resolved alcohol (*R*)-**2k** with an *er* of 13.5:86.5 at 43% isolated yield with an *s*-value of 9.2 at 54% conversion although an additional hour was needed to reach that conversion. Compound **2l** with a 2-bromo substituent was resolved at 53% conversion after 8 h with an *s*-value of 11.3. Interestingly, swapping the 2-substituent in the phenyl moiety to a phenyl group as in **2m** afforded the (*R*)-alcohol **2m** in 10.5:89.5 *er* at 49% conversion corresponding to an *s*-value of 24.

We then examined compounds for which the 2-phenyl substituent on the phenyl ring of the *N*-phenylpyrrole system was left as a constant, but the pyrrole phenyl ring substituted was varied. For formaldehyde acetal-protected catechol **2n**, the reaction rate was very low taking 24 h to reach 41% conversion. As *N*-aryl-pyrroles may act as ligands for copper, it is possible that product inhibition caused this dropping reaction rate. The *s*-value for this OKR was 8.5. With a 3-nitro substituted phenyl group on the pyrrole in **2o**, the reaction was also retarded, and the OKR took 36 h to reach 40% conversion with an *s*-value of 6.6. Similarly with a 2-naphthyl group on the pyrrole as in **2p**, 48 h was required to reach 46% conversion with an *s*-value of 6.4.

The major limitation found for this OKR process is the requirement to have an ester group at position 3 of the pyrrole ring for successful resolution. Although oxidation occurred, resolution of the substrate *rac*-**2q** with no ester group did not show any appreciable enantioselectivity. Although there is no concrete evidence, the presence of an ester group seems to be very specific for axially chiral pyrroles as it was also observed by the Tan’s group during the development of asymmetric Paal–Knorr synthesis to access axially chiral pyrroles.^17a^

After the successful resolution of axially chiral pyrroles, we turned our attention to the resolution of racemic bis-benzene-type alcohols. Initially, the same catalytic conditions used for OKR of racemic *N*-phenylpyrrole alcohols **2** have been tested for biaryl alcohol **4a** (Table 1, entry 1). In general, alcohols **4** reacted faster than *N*-arylpyrrole alcohols **2**. Most substrates reached 50% conversion in 2.5–5 h of oxidation. Thus, after 2.5 h, racemic alcohol **4a** was oxidized to the corresponding aldehyde **5a** at 64% conversion, and the chiral *R*-alcohol was recovered in 5.5:94.5 *er* with a selectivity factor of 8.4. The product aldehyde **5a** was obtained in 60:40 *er*. In order to check the influence of the solvent on stereoselectivity, the reaction was performed in different solvents while keeping the other parameters constant (Table 1, entries 2–5). Though the conversion in dichloromethane was similar to acetonitrile, the enantioselectivity diminished (Table 1, entry 2). In dimethylformamide, the reaction was slow resulting in the recovered **4a** in 34.5:65.5 *er* and the aldehyde product **5a** in 90:10 *er* with an *s*-value of 12.0 (Table 1, entry 4). The reaction was also quite slow in ethyl acetate and tetrahydrofuran producing only 4 and 2% conversion, respectively, after 20 h (Table 1, entries 3 and 5). The catalytic reactivity of other copper(I) salts was also investigated for **4a**. However, the reactivity and stereoselectivity were lower for copper chloride and copper iodide in comparison to copper bromide (Table 1, entries 6–7). Hence, the use of 2 mol % of copper bromide in acetonitrile in the presence of 2 mol % of chiral hydroxyl amine precatalyst (1*S*,3*S*)-**1** gave the best result for the OKR of **4a** in terms of yield and *er* and was again chosen for further studying the scope of the reaction.

**Table 1:**
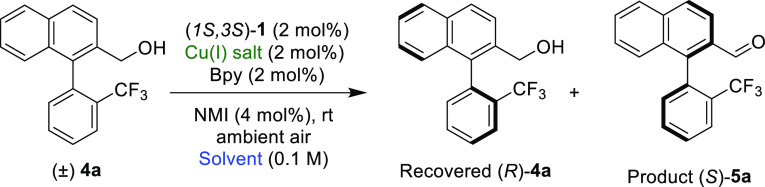
Optimization of Reaction Conditions^a^

entry	solvent	Cu(I)-salt	*t* (h)	*C*^b^	(*R*)-**4a** (%)^c^	(*S*)-**5a** (%)^c^	*er***4a**	*er***5a**	*s*
1	MeCN	CuBr	2.5	64	28	54	5.5:94.5	60:40	8.4
2	DCM	CuBr	3.5	75	19	72	23.5:76.5	55:45	2.2
3	EtOAc	CuBr	20	4					
4	DMF	CuBr	6	28	69	23	34.5:65.5	90:10	12.0
5	THF	CuBr	20	2					
6	MeCN	CuCl	5	38	55	34	42.5:57.5	60.5:39.5	1.9
7	MeCN	CuI	4.5	19	74	17	45.5:54.5	61.5:38.5	2.4

a Reactions were carried out with 100 mg of **4a**, (1*S*,3*S*)-**1** (2 mol %), Cu(I)-salt (2 mol %), bipyridine (2 mol %), and *N*-methyl imidazole (4 mol %) in solvent (0.1 M).

b *C* represents conversion (by ^1^H NMR analysis). Enantiomeric ratio (*er*) values were determined by chiral HPLC. In all cases, 10 mg of product **5** was reduced to the corresponding alcohol and determined *er*.

c Isolated yields. The selectivity factor (*s*) was calculated by *s* = ln[(1 – C) (1 – *ee*)]/ln[(1 – *C*) (1 + *ee*)] where *ee* = enantiomeric excess of recovered alcohol **4a**.

The absolute configuration of the known recovered alcohols **4b** and **4j** and product aldehydes **5b**, **5f**, and **5g** was assigned by comparing the optical rotation of the obtained products to data that have been described previously^18−22^ (see the Experimental Section and Supporting Information for details), and the configuration of the remaining compounds was assigned by analogy.

It was found that both substrates with electron-donating and electron-withdrawing groups on either part of the benzene rings are well tolerated. Bi-naphthyl alcohol *rac*-**4b** was resolved in 3 h at 56% conversion with an s value of 6.5. Other compounds with one substituted benzene ring and one naphthyl ring gave similar *s*-values. Thus, *rac*-**4c** was resolved with 60% conversion in 3.5 h to give the recovered alcohol (*R*)-**4c** in 35% isolated yield with an *er* of 19:81 (*s*-value 4.3). Differentially protected catechol **4d** with one less oxygen on the benzene than **4c** reacted even faster with an *s*-value of 2.9 and 64% conversion in 3 h. Compound **4e** that has two identical methyl protecting groups on the catechol moiety underwent OKR with an *s*-value of 3.7.

Compound **4f** with a methyl substituent in the 2-position of the bottom naphthyl and no substituents on the upper phenyl ring apart from the methyl alcohol was resolved with an *er* of 30:70 (44% isolated yield) corresponding to an *s*-value of 4.8. Racemic alcohol **4g** with an electron-donating methyl group on the phenyl ring and a methyl substituent on the naphthyl in the 4-position was resolved with an *s*-factor 4.1 in 3.5 h at 66% conversion with an *er* of 19:81. Substrates **4h** and **4i** with 2-CF_3_ groups in the lower phenyl ring were resolved with higher selectivity factors of 6.3 and 7.3, respectively. In contrast, *Rac*-alcohols **4j** and **4k**, with the upper ring being of the naphthyl type, were found to be poor substrates for OKR and yielded low *er* for the recovered alcohols.

However, a *rac*-alcohol with an upper naphthyl group and a lower 2-phenyl-phenyl group **4l** was resolved with high *er* (8:92, 20% isolated yield at 65% conversion in 5 h) and a selectivity factor of 6.6. Racemic alcohols **4m–4q** containing alkoxy group at meta-position with respect to the hydroxymethyl group were resolved successfully. While compound **4m** substituted with a trifluoromethyl group in the lower phenyl group was resolved with an *s*-value of 4.6 in 3 h at 63% conversion, a similar compound with methyl substitution **4q** was resolved with a higher *s*-value 6.1 in 3 h, indicating that a methyl group is better than the trifluoromethyl group in the atropo-differentiating step. Racemic alcohol **4n** with a lower naphthyl ring and a methoxy substitution on the phenyl ring was resolved in 3 h at 70% conversion with a *s*-factor 6.1. Replacing methoxy group in **4n** with the ethoxy group as in compound **4o** did not have great impact except slightly reducing the rate of oxidation. Compound **4o** was resolved in 3 h with an *s*-value of 6.1 at 64% conversion. Compound **4p** substituted with a benzyloxy group was resolved successfully with an *s*-value of 6.3. Replacing the lower naphthyl group of **4n** with a 2-methyl-phenyl group, i.e., compound **4q**, allowed resolution with an *s*-value of 6.1.

Finally, compound **4r** with an iodo-substituent on the lower phenyl group was resolved on a 1 mmol scale in only 3.5 h with an *s*-value of 4.3, allowing the isolation of recovered (*R*)-**4r** in 22% isolated yield based on 76% conversion. The corresponding aldehyde oxidation product was isolated in 73% yield and a 62.5:37.5 *er*.

These axially chiral iodine-substituted compounds can be elaborated to further transformations using iodine as a handle or could potentially be used as chiral oxidants for enantioselective processes α-tosylation of carbonyl compounds.^23^

**Scheme 3:**
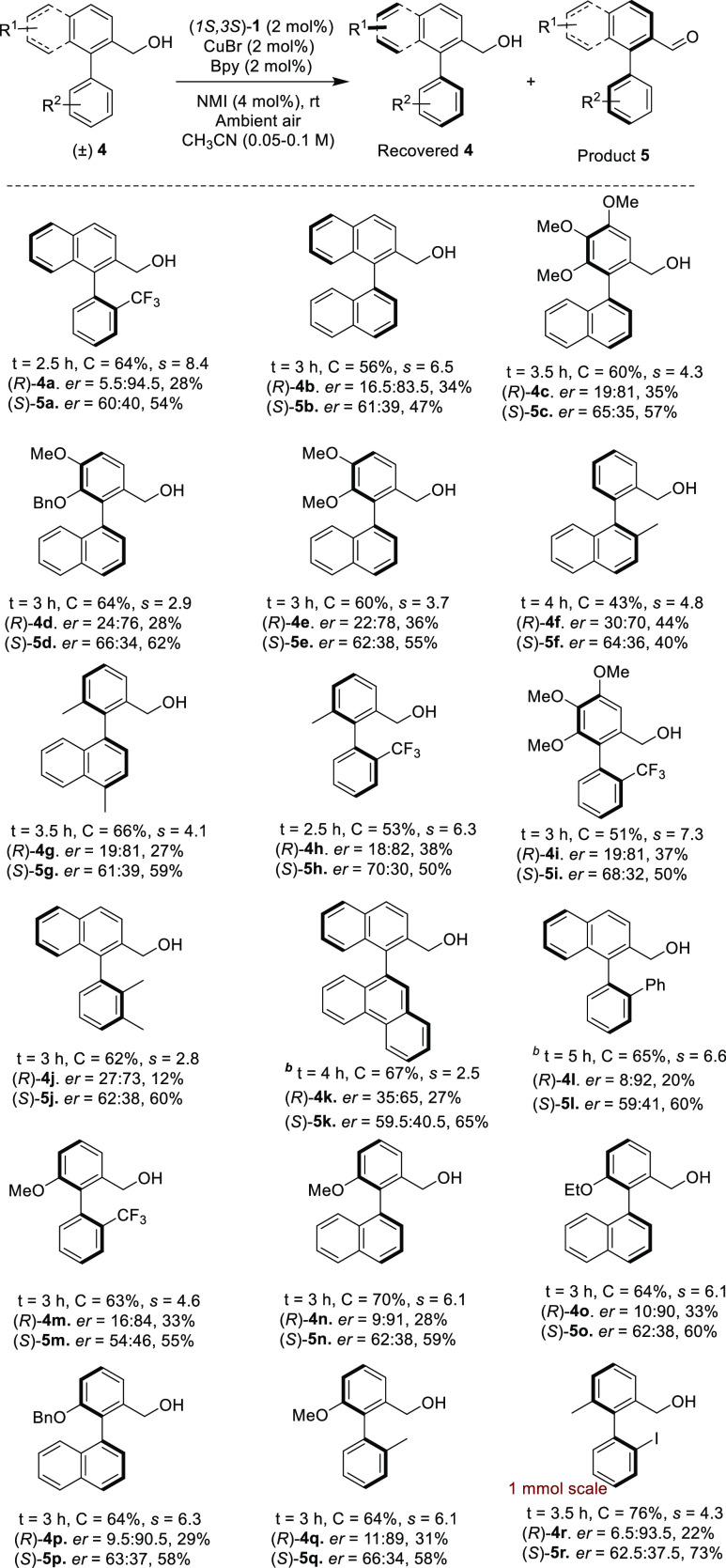
OKR of *rac*-Biaryl Alcohols to Access Axially Chiral Biaryl Compounds Unless otherwise stated, reactions were carried out with 100 mg of **4**, (1*S*,3*S*)-**1** (2 mol %), Cu(I)Br (2 mol %), bipyridine (2 mol %), and *N*-methyl imidazole (4 mol %) in CH_3_CN (0.1 M). *er* values were determined by chiral HPLC. In all cases, 10 mg of product **5** was reduced to the corresponding alcohol and determined *er*. The selectivity factor (*s*) was calculated by *s* = ln[(1 – *C*) (1 – *ee*)]/ln[(1 – *C*) (1 + *ee*)]. C represents conversion (by ^1^H NMR analysis). *ee* = *ee* of the recovered alcohol. ^b^Reaction was carried out in a mixture of CH_3_CN–EtOAc (1:1) (0.05 M).

It is noteworthy to mention that unlike the OKR of *rac*-*N*-aryl pyrroles (Scheme 2), there is no requirement to have an ester group ortho to the hydroxy methyl group for the successful resolution of *rac*-biaryls (Scheme 3). However, the OKR of *rac*-aryl pyrroles exhibited higher orders of enantioselectivities compared to the resolution of *rac*-biaryls.

**Scheme 4:**
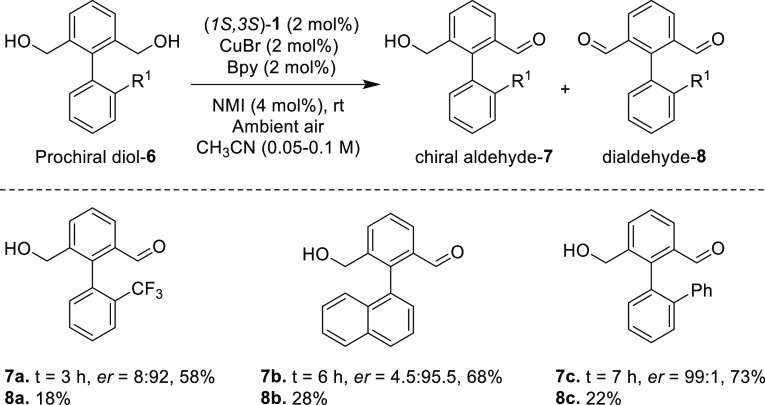
Oxidative Desymmetrization of Prochiral Diols Unless otherwise stated, reactions were carried out with 100 mg of **6**, (1*S*,3*S*)-**1** (2 mol %), Cu(I)Br (2 mol %), bipyridine (2 mol %), and *N*-methyl imidazole (4 mol %) in CH_3_CN (0.1 M). *er* values were determined by chiral HPLC.

We next addressed the possibility of applying this copper-chiral nitroxide oxidation process to the desymmetrization of prochiral diols to synthesize atropisomers (Scheme 4). To this end, we synthesized prochiral diols **6a–6c** from the commercially available starting materials (see the Supporting Information). Achiral diol **6a** was subjected to desymmetrization using the same set of reaction conditions established for resolution. Remarkably, after 3 h of oxidation, optically pure mono aldehyde **7a** was obtained in 58% of isolated yield with 8:92 *er*. Along with chiral monoaldehyde **7a**, achiral dialdehyde **8a** was also formed in 18% yield. Formation of dialdehyde is due to the over oxidation of monoaldehyde **7a**. Similarly, achiral diols **6b** and **6c** were also subjected to the desymmetrization. In the case of substrate **6b**, after 6 h of oxidation, desired product **7b** was isolated in 68% yield with 4.5:95.5 *er* along with 28% of dialdehyde **8b**. Substrate substituted with phenyl group **6c** also underwent desymmetrization and afforded the desired chiral monoaldehyde **7c** in 73% yield with an exceptional *er* 99:1 in 7 h of oxidation. These impressive outcomes demonstrate the potential of our newly developed chiral hydroxylamine precatalyst (1*S*,3*S*)-**1** in enantioselective oxidations.

The examined axially chiral compounds in this study were found to be configurationally stable on storage and even when standing in solution for several days. Thus, the few percent stereo-erosion observed must have occurred during the oxidation step.

In conclusion, we have developed efficient copper and chiral nitroxide-catalyzed enantioselective oxidation protocols to synthesize axially chiral *N*-arylpyrroles and axially chiral biaryl compounds in high enantioselectivities. Importantly, ambient air was used as a stoichiometric terminal oxidant. A diverse range of racemic substrates were resolved with moderate to good selectivity factors. Additionally, desymmetrization of prochiral diols may be achieved with high enantioselectivities. Further enantioselective oxidation processes using this elegant copper-chiral nitroxide protocol is underway in our laboratory.

## Experimental Section

### General Information

Unless indicated otherwise, all non-aqueous reactions were carried out using oven-dried (120 °C) glassware under a positive pressure of argon. Commercially available reagents were used without further purification. Except if specified otherwise, reactions were magnetically stirred and monitored by thin-layer chromatography (TLC) using Merck Silica Gel 60 F254 plates and visualized under UV light. In addition, TLC plates were stained using potassium permanganate and 2,4-dinitrophenylhydrazine stains. Chromatographic purification of products (flash chromatography) was performed on silica gel (230–400 mesh). Concentration under reduced pressure was performed by rotary evaporation at 40 °C at the appropriate pressure. Yields refer to chromatographically purified compounds. NMR spectroscopy: NMR spectra were recorded on a Bruker spectrometer operating at 400 MHz and 101 MHz for ^1^H and ^13^C acquisitions, respectively. Chemical shifts (δ) for ^1^H NMR are reported in parts per million (ppm) relative to tetramethylsilane in CDCl_3_ (δ 0.00 ppm) or residual chloroform in CDCl_3_ (δ 7.26 ppm). All the ^13^C chemical shifts were reported in ppm with reference to CDCl_3_ (δ 77.16 ppm). All ^13^C spectra were measured with complete proton decoupling. Data are reported as follows: s = singlet, d = doublet, t = triplet, q = quartet, m = multiplet, and br = broad signal; coupling constants in Hz. IR spectroscopy: IR spectra were recorded on a JASCO FT-IR spectrometer (as neat) in the 600–3600 cm^–1^ region. Absorptions are given in wavenumbers (cm^–1^). Chiral HPLC: enantiomeric excess was determined by Thermo Scientific HPLC and Shimadzu HPLC instruments, using a Lux 5 μm Cellulose-1 (250 × 4.60 mm) column, a Lux 5 μm Amylose-2 (250 × 4.60 mm) column, Chiralpak AD-H (250 × 4.60 mm), Chiralpak AD-H (150 × 4.60 mm), and Cellulose-1 OD-H (250 × 4.60 mm) column with hexane and isopropanol (IPA) as solvents. Mass spectra: high-resolution mass spectra were obtained using a Xevo G2-XS-QToF device. Data were acquired using the resolution mode under positive/negative electrospray ionization. The acquisition range was 50–1200 *m*/*z*. The capillary voltage was 2.0 kV, and the cone voltage was 40 V. Scan time was 0.5 s. In all cases, samples were injected direct to MS using methanol as the eluent. Optical activity: optical activity of the compounds was measured by a Bellingham Stanley polarimeter ADP 450. Absolute stereochemistry: absolute stereochemistry of products was determined by comparing the optical rotation of known compounds. The absolute stereochemistry of remaining compounds was assigned by analogy. Selectivity factor (s): the selectivity factor of these kinetic resolution process was calculated by Kagan’s equation.^24^ X-ray structure: X-ray crystallographic data were measured on a Bruker Apex-II instrument.

#### Synthesis of Chiral Hydroxylamine (1*S*,3*S*)-**1**

The chiral hydroxylamine precatalyst (1*S*,3*S*)-**1** was synthesized by the previously reported procedure from our group.^16^

#### General Procedure for the OKR of Racemic *N*-Arylpyrrole Alcohols (**2a–q**)^16^

In a 20 mL glass vial, 100 mg of racemic **2** (1 equiv) was dissolved in analytical grade acetonitrile (0.1 M). CuBr (2 mol %), bipyridine (2 mol %), chiral hydroxylamine (1*S*,3*S*)-**1** (2 mol %), and *N*-methylimidazole (4 mol %, 0.1 M acetonitrile solution) were added to the reaction mixture. The resulting red/brown colored reaction mixture was stirred at RT (23–25 °C) open to air for specified time. The reaction progress was monitored by ^1^H NMR analysis. After the specified conversion, the solvent from the reaction mixture was removed under the reduced pressure. The crude reaction mixture was subjected to flash column chromatography using hexane and ethyl acetate as the eluent to afford aldehyde **3** and recovered alcohol **2**.

##### (*R*)-Ethyl 2-(Hydroxymethyl)-1-(2-isopropylphenyl)-5-phenyl-1*H*-pyrrole-3-carboxylate (**2a**)^16^

According to the general procedure for kinetic resolution, substrate **2a** (100 mg, 0.275 mmol) with catalyst (1*S*,3*S*)-**1** (4.22 mg, 0.0055 mmol) in acetonitrile (2.80 mL) at 23–25 °C afforded aldehyde **3a** and recovered **2a** in 5 h at 57% conversion. Isolated yield: 38% (38 mg). Physical state: brown colored sticky material, *R*_f_ = 0.38 (20% EtOAc/hexane). ^1^H NMR (400 MHz, CDCl_3_): δ 7.41 (dd, *J* = 7.2, 1.6 Hz, 1H), 7.36–7.26 (m, 3H), 7.15–7.10 (m, 3H), 7.09–7.04 (m, 2H), 6.85 (s, 1H), 4.53–4.34 (m, 4H), 4.27 (t, *J* = 7.2 Hz, 1H), 2.34–2.22 (m, 1H), 1.41 (t, *J* = 7.2 Hz, 3H), 0.97 (d, *J* = 7.2 Hz, 3H), 0.60 (d, *J* = 6.8 Hz, 3H). ^13^C{^1^H} NMR (101 MHz, CDCl_3_): δ 166.7, 147.1, 141.7, 135.1, 134.8, 132.0, 130.0, 129.5, 128.3 (2C), 128.2 (2C), 127.1, 127.1, 126.5, 114.1, 109.7, 60.6, 55.6, 27.7, 24.6, 22.3, 14.6. IR (neat): 3455, 2964, 2869, 1675, 1492, 1451, 1241, 1081, 755 cm^–1^. HRMS (ES+) *m*/*z*: [M + Na]^+^ calcd for C_23_H_25_NO_3_Na, 386.1732; found, 386.1729. [α]_D_^20^ = −60.2 (*c* = 0.38, CHCl_3_). HPLC: *er* = 3.5:96.5. The *er* was determined by chiral HPLC analysis using a Lux 5 μm cellulose-1 (250 × 4.6 mm) column [hexane/isopropanol (80:20), 0.7 mL/min, λ = 280 nm, *t*_minor_ = 6.24 min, *t*_major_ = 8.06 min].

##### (*S*)-Ethyl 2-Formyl-1-(2-isopropylphenyl)-5-phenyl-1*H*-pyrrole-3-carboxylate (**3a**)^16^

Isolated yield: 53% (53 mg). Physical state: pale yellow sticky material, *R*_f_ = 0.46 (20% EtOAc/hexane). ^1^H NMR (400 MHz, CDCl_3_): δ 10.40 (s, 1H), 7.41 (m, 1H), 7.29–7.23 (m, 3H), 7.22–7.14 (m, 3H), 7.12–7.09 (m, 2H), 7.00 (s, 1H), 4.42 (q, *J* = 7.2 Hz, 2H), 2.30–2.23 (m, 1H), 1.42 (t, *J* = 7.2 Hz, 3H), 0.96 (d, *J* = 7.2 Hz, 3H), 0.64 (d, *J* = 6.8 Hz, 3H). ^13^C{^1^H} NMR (101 MHz, CDCl_3_): δ 181.3, 163.8, 145.3, 141.3, 136.1, 133.1, 130.4, 129.7, 129.0 (2C), 128.8, 128.4, 128.3 (2C), 126.8, 126.1, 125.0, 112.9, 61.1, 27.9, 24.1, 22.4, 14.5. IR (neat): 2964, 2869, 1711, 1666, 1467, 1219, 1092, 755 cm^–1^. HRMS (ES+) *m*/*z*: [M + Na]^+^ calcd for C_23_H_23_NO_3_Na, 384.1576; found, 384.1572. [α]_D_^20^ = +14.5 (*c* = 0.45, CHCl_3_). HPLC: *er* = 76.5:23.5 (10 mg of aldehyde was reduced to the corresponding alcohol with NaBH4 and determined the *er*). The *er* was determined by chiral HPLC analysis using a Lux 5 μm cellulose-1 (250 × 4.6 mm) column [hexane/isopropanol (80:20), 0.7 mL/min, λ = 280 nm, *t*_major_ = 6.17 min, *t*_minor_ = 7.86 min].

##### (*R*)-Ethyl 2-(Hydroxymethyl)-1-(2-isopropylphenyl)-5-(*p*-tolyl)-1*H*-pyrrole-3-carboxylate (**2b**)^16^

According to the general procedure for kinetic resolution, substrate **2b** (100 mg, 0.265 mmol) with catalyst (1*S*,3*S*)-**1** (4.06 mg, 0.0053 mmol) in acetonitrile (2.65 mL) at 23–25 °C afforded aldehyde **3b** and recovered **2b** in 5 h at 60% conversion. Isolated yield: 38% (38 mg). Physical state: brown sticky material, *R*_f_ = 0.41 (20% EtOAc/hexane). ^1^H NMR (400 MHz, CDCl_3_): δ 7.43–7.39 (m, 1H), 7.35–7.27 (m, 3H), 6.99–6.91 (m, 4H), 6.81 (s, 1H), 4.53–4.33 (m, 4H), 4.29 (t, *J* = 7.2 Hz, 1H), 2.34–2.28 (m, 1H), 2.23 (s, 3H), 1.41 (t, *J* = 6.8 Hz, 3H), 0.98 (d, *J* = 6.8 Hz, 3H), 0.63 (d, *J* = 6.8 Hz, 3H). ^13^C{^1^H} NMR (101 MHz, CDCl_3_): δ 166.7, 147.1, 141.5, 136.9, 135.2, 134.9, 129.9, 129.5, 129.1, 128.9 (2C), 128.2 (2C), 127.1, 126.5, 114.0, 109.2, 60.5, 55.6, 27.7, 24.6, 22.4, 21.2, 14.6. IR (neat): 3478, 2968, 2867, 1671, 1490, 1247, 1004, 780 cm^–1^. HRMS (ES+) *m*/*z*: [M + Na]^+^ calcd for C_24_H_27_NO_3_Na:400.1889; found, 400.1884. [α]_D_^20^ = −39.1 (*c* = 0.38, CHCl_3_). HPLC: *er* = 6.0:94.0. The *er* was determined by chiral HPLC analysis using a Lux 5 μm cellulose-1 (250 × 4.6 mm) column [hexane/isopropanol (80:20), 0.6 mL/min, λ = 254 nm, *t*_minor_ = 6.84 min, *t*_major_ = 7.43 min].

##### (*S*)-Ethyl 2-Formyl-1-(2-isopropylphenyl)-5-(*p*-tolyl)-1*H*-pyrrole-3-carboxylate (**3b**)^16^

Isolated yield: 55% (55 mg). Physical state: pale yellow solid, mp: 96–100 °C, *R*_f_ = 0.60 (20% EtOAc/hexane). ^1^H NMR (400 MHz, CDCl_3_): δ 10.37 (s, 1H), 7.42–7.38 (m, 1H), 7.26–7.25 (m, 3H), 7.00–6.93 (m, 5H), 4.40 (q, *J* = 7.2 Hz, 2H), 2.30–2.21 (m, 4H), 1.40 (d, *J* = 7.2 Hz, 3H), 0.95 (d, *J* = 6.8 Hz, 3H), 0.65 (d, *J* = 7.2 Hz, 3H). ^13^C{^1^H} NMR (101 MHz, CDCl_3_): δ 181.2, 163.9, 145.3, 141.5, 138.4, 136.3, 133.0, 129.6, 129.0 (2C), 128.8 (3C), 127.5, 126.8, 126.1, 125.0, 112.7, 61.0, 27.9, 24.1, 22.5, 21.3, 14.5. IR (neat): 2965, 2924, 2867, 1709, 1657, 1432, 1219, 1059, 771 cm^–1^. HRMS (ES+) *m*/*z*: [M + Na]^+^ calcd for C_24_H_25_NO_3_Na, 398.1732; found, 398.1729. [α]_D_^20^ = +34.8 (*c* = 0.45, CHCl_3_). HPLC: *er* = 77:23 (10 mg of aldehyde was reduced to the corresponding alcohol with NaBH_4_ and determined the *er*). The *er* was determined by chiral HPLC analysis using a Lux 5 μm cellulose-1 (250 × 4.6 mm) column [hexane/isopropanol (80:20), 0.6 mL/min, λ = 254 nm, *t*_major_ = 6.80 min, *t*_minor_ = 7.53 min].

##### (*R*)-Ethyl-5-(4-chlorophenyl)-2-(hydroxymethyl)-1-(2-isopropylphenyl)-1*H*-pyrrole-3-carboxylate (**2c**)^16^

According to the general procedure for kinetic resolution, substrate **2c** (100 mg, 0.25 mmol) with catalyst (1*S*,3*S*)-**1** (3.85 mg, 0.0050 mmol) in a mixture (1:1) of acetonitrile (2.50 mL) and ethyl acetate (2.50 mL) at 23–25 °C afforded aldehyde **3c** and recovered **2c** in 7 h at 51% conversion. Isolated yield: 45% (45 mg). Physical state: pale yellow solid, mp: 117–121 °C, *R*_f_ = 0.42 (20% EtOAc/hexane). ^1^H NMR (400 MHz, CDCl_3_): δ 7.46–7.42 (m, 1H), 7.34–7.22 (m, 3H), 7.13–7.09 (m, 2H), 7.01–6.98 (m, 2H), 6.85 (s, 1H), 4.51–4.35 (m, 4H), 4.25 (t, *J* = 7.2 Hz, 1H), 2.32–2.22 (m, 1H), 1.41 (t, *J* = 6.8 Hz, 3H), 0.98 (d, *J* = 6.8 Hz, 3H), 0.65 (d, *J* = 6.9 Hz, 3H). ^13^C{^1^H} NMR (101 MHz, CDCl_3_): δ 166.5, 147.1, 142.0, 134.5, 133.8, 133.1, 130.5, 130.2, 129.4, 129.4 (2C), 128.4 (2C), 127.3, 126.7, 114.3, 110.0, 60.6, 55.7, 27.7, 24.6, 22.4, 14.6. IR (neat): 3507, 2966, 1679, 1557, 1490, 1245, 1080, 753 cm^–1^. HRMS (ES+) *m*/*z*: [M + Na]^+^ calcd for C_23_H_24_ClNO_3_Na, 420.1342; found, 420.1342. [α]_D_^20^ = −32.6 (*c* = 0.45, CHCl_3_). HPLC: *er* = 20.5:79.5. The *er* was determined by chiral HPLC analysis using a Lux 5 μm cellulose-1 (250 × 4.6 mm) column [hexane/isopropanol (90:10), 0.7 mL/min, λ = 280 nm, *t*_minor_ = 7.52 min, *t*_major_ = 7.92 min].

##### (*S*)-Ethyl 5-(4-Chlorophenyl)-2-formyl-1-(2-isopropylphenyl)-1*H*-pyrrole-3-carboxylate (**3c**)^16^

Isolated yield: 46% (46 mg). Physical state: off-white solid, mp: 107–112 °C, *R*_f_ = 0.56 (20% EtOAc/hexane). ^1^H NMR (400 MHz, CDCl_3_): δ 10.39 (s, 1H), 7.45–7.40 (m, 1H), 7.29–7.25 (m, 3H), 7.16–7.14 (m, 2H), 7.04–7.02 (m, 2H), 6.99 (s, 1H), 4.42 (q, *J* = 7.2 Hz, 2H), 2.26–2.16 (m, 1H), 1.43 (t, *J* = 7.1 Hz, 3H), 0.96 (d, *J* = 6.9 Hz, 3H), 0.68 (d, *J* = 6.9 Hz, 3H). ^13^C{^1^H} NMR (101 MHz, CDCl_3_): δ 181.4, 163.7, 145.3, 139.9, 135.9, 134.6, 133.3, 130.1 (2C), 129.9, 128.9, 128.7, 128.6 (2C), 127.0, 126.3, 124.9, 113.1, 61.2, 28.0, 24.1, 22.5, 14.5. IR (neat): 2962, 2868, 1700, 1672, 1461, 1434, 1226, 1095, 759 cm^–1^. HRMS (ES+) *m*/*z*: [M + Na]^+^ calcd for C_23_H_22_ClNO_3_Na, 418.1186; found, 418.1190. [α]_D_^20^ = +28.9 (*c* = 0.35, CHCl_3_). HPLC: *er* = 81:19 (10 mg of aldehyde was reduced to the corresponding alcohol with NaBH_4_ and determined the *er*). The *er* was determined by chiral HPLC analysis using a Lux 5 μm cellulose-1 (250 × 4.6 mm) column [hexane/isopropanol (90:10), 0.7 mL/min, λ = 280 nm, *t*_major_ = 7.40 min, *t*_minor_ = 7.79 min].

##### (*R*)-Ethyl 5-(Benzo[*d*][1,3]dioxol-5-yl)-2-(hydroxymethyl)-1-(2-isopropylphenyl)-1*H*-pyrrole-3-carboxylate (**2d**)^16^

According to the general procedure for kinetic resolution, substrate **2d** (100 mg, 0.245 mmol) with catalyst (1*S*,3*S*)-**1** (3.76 mg, 0.0049 mmol) in acetonitrile (2.50 mL) at 23–25 °C afforded aldehyde **3d** and recovered **2d** in 10 h at 52% conversion. Isolated yield: 45% (45 mg). Physical state: pale yellow solid, mp: 112–115 °C, *R*_f_ = 0.36 (20% EtOAc/hexane). ^1^H NMR (400 MHz, CDCl_3_): δ 7.42 (m, 1H), 7.33–7.27 (m, 3H), 6.75 (s, 1H), 6.60–6.54 (m, 3H), 5.86 (s, 2H), 4.50–4.33 (m, 4H), 4.27 (t, *J* = 7.2 Hz, 1H), 2.36–2.25 (m, 1H), 1.41 (t, *J* = 7.1 Hz, 3H), 0.98 (d, *J* = 6.9 Hz, 3H), 0.71 (d, *J* = 6.9 Hz, 3H). ^13^C{^1^H} NMR (101 MHz, CDCl_3_): δ 166.6, 147.4, 147.1, 146.8, 141.4, 134.8, 134.7, 130.0, 129.4, 127.2, 126.6, 126.0, 122.4, 113.9, 109.3, 108.9, 108.1, 101.1, 60.5, 55.6, 27.7, 24.6, 22.5, 14.6. IR (neat): 3467, 2965, 1674, 1476, 1226, 1037, 754 cm^–1^. HRMS (ES+) *m*/*z*: [M + Na]^+^ calcd for C_24_H_25_NO_5_Na: 430.1631; found, 430.1631. [α]_D_^20^ = −53.5 (*c* = 0.45, CHCl_3_). HPLC: *er* = 16:84. The *er* was determined by chiral HPLC analysis using a Lux 5 μm cellulose-1 (250 × 4.6 mm) column [hexane/isopropanol (80:20), 0.7 mL/min, λ = 280 nm, *t*_minor_ = 7.84 min, *t*_major_ = 8.63 min].

##### (*S*)-Ethyl-5-(benzo[*d*][1,3]dioxol-5-yl)-2-formyl-1-(2-isopropylphenyl)-1*H*-pyrrole-3-carboxylate (**3d**)^16^

Isolated yield: 47% (47 mg). Physical state: brown colored solid, mp: 119–122 °C, *R*_f_ = 0.46 (20% EtOAc/hexane). ^1^H NMR (400 MHz, CDCl_3_): δ 10.36 (s, 1H), 7.44–7.39 (m, 1H), 7.30–7.22 (m, 3H), 6.91 (s, 1H), 6.63–6.63 (m, 2H), 6.55–6.54 (m, 1H), 5.89 (s, 2H), 4.41 (q, *J* = 7.2 Hz, 2H), 2.26 (m, 1H), 1.42 (t, *J* = 7.2 Hz, 3H), 0.97 (d, *J* = 6.8 Hz, 3H), 0.74 (d, *J* = 6.8 Hz, 3H). ^13^C{^1^H} NMR (101 MHz, CDCl_3_): δ 181.2, 163.8, 147.8, 147.6, 145.3, 141.0, 136.1, 132.9, 129.8, 128.8, 126.9, 126.2, 124.9, 124.2, 123.4, 112.7, 109.2, 108.3, 101.4, 61.1, 28.0, 24.1, 22.6, 14.5. IR (neat): 2957, 2866, 1708, 1665, 1468, 1218, 1028, 772 cm^–1^. HRMS (ES+) *m*/*z*: [M + Na]^+^ calcd for C_24_H_23_NO_5_Na, 428.1474; found, 428.1475. [α]_D_^20^ = +16.0 (*c* = 0.37, CHCl_3_). HPLC: *er* = 79.5:20.5 (10 mg of aldehyde was reduced to the corresponding alcohol with NaBH_4_ and determined the *er*). The *er* was determined by chiral HPLC analysis using a Lux 5 μm cellulose-1 (250 × 4.6 mm) column [hexane/isopropanol (80:20), 0.7 mL/min, λ = 280 nm, *t*_major_ = 7.78 min, *t*_minor_ = 8.61 min].

##### (*R*)-Methyl 5-(3-Bromophenyl)-2-(hydroxymethyl)-1-(2-isopropylphenyl)-1*H*-pyrrole-3-carboxylate (**2e**)^16^

According to the general procedure for kinetic resolution, substrate **2e** (100 mg, 0.23 mmol) with catalyst (1*S*,3*S*)-**1** (3.57 mg, 0.0046 mmol) in acetonitrile (2.4 mL) at 23–25 °C afforded aldehyde **3e** and recovered **2e** in 5 h at 58% conversion. Isolated yield: 37% (37 mg). Physical state: light brown colored solid, mp: 112–114 °C, *R*_f_ = 0.26 (20% EtOAc/hexane). ^1^H NMR (400 MHz, CDCl_3_): δ 7.47–7.43 (m, 1H), 7.35–7.29 (m, 3H), 7.27–7.24 (m, 2H), 7.00–6.92 (m, 2H), 6.87 (s, 1H), 4.45 (ddd, *J* = 38.8, 13.7, 7.2 Hz, 2H), 4.18 (t, *J* = 7.2 Hz, 1H), 3.91 (s, 3H), 2.34–2.23 (m, 1H), 0.99 (d, *J* = 6.8 Hz, 3H), 0.68 (d, *J* = 6.8 Hz, 3H). ^13^C{^1^H} NMR (101 MHz, CDCl_3_): δ 166.9, 147.0, 142.3, 134.4, 133.9, 133.4, 131.1, 130.3, 130.0, 129.7, 129.3, 127.3, 126.7, 126.5, 122.3, 114.0, 110.4, 55.5, 51.8, 27.8, 24.6, 22.4. IR (neat): 3446, 2963, 2868, 1682, 1439, 1242, 1079, 775 cm^–1^. HRMS (ES+) *m*/*z*: [M + Na]^+^ calcd for C_22_H_22_BrNO_3_Na, 450.0681; found, 450.0675, [α]_D_^20^ = −20.1 (*c* = 0.35, CHCl_3_). HPLC: *er* = 4:96. The *er* was determined by chiral HPLC analysis using a Lux 5 μm cellulose-1 (250 × 4.6 mm) column [hexane/isopropanol (80:20), 0.7 mL/min, λ = 280 nm, *t*_minor_ = 7.02 min, *t*_major_ = 9.22 min].

##### (*S*)-Methyl 5-(3-Bromophenyl)-2-formyl-1-(2-isopropylphenyl)-1*H*-pyrrole-3-carboxylate (**3e**)^16^

Isolated yield: 54% (54 mg). Physical state: yellow solid, mp: 98–102 °C, *R*_f_ = 0.49 (20% EtOAc/hexane). ^1^H NMR (400 MHz, CDCl_3_): δ 10.39 (s, 1H), 7.46–7.42 (m, 1H), 7.35–7.26 (m, 4H), 7.23 (dd, *J* = 7.8, 1.5 Hz, 1H), 7.05–6.97 (m, 3H), 3.95 (s, 3H), 2.29–2.18 (m, 1H), 0.97 (d, *J* = 6.8 Hz, 3H), 0.71 (d, *J* = 6.8 Hz, 3H). ^13^C{^1^H} NMR (101 MHz, CDCl_3_): δ 181.4, 164.1, 145.3, 139.4, 135.7, 133.5, 132.3, 131.9, 131.4, 130.0, 129.8, 128.6, 127.3, 127.0, 126.4, 124.4, 122.3, 113.2, 52.2, 28.0, 24.1, 22.4. IR (neat): 2965, 2874, 1713, 1671, 1434, 1325, 1223, 757 cm^–1^. HRMS (ES+) *m*/*z*: [M + Na]^+^ calcd for C_22_H_20_BrNO_3_Na, 448.0524; found, 448.0519. [α]_D_^20^ = +20.1 (*c* = 0.45, CHCl_3_). HPLC: *er* = 76.5:23.5 (10 mg of aldehyde was reduced to the corresponding alcohol with NaBH_4_ and determined the *er*). The *er* was determined by chiral HPLC analysis using a Lux 5 μm cellulose-1 (250 × 4.6 mm) column [hexane/isopropanol (80:20), 0.7 mL/min, λ = 280 nm, *t*_major_ = 7.04 min, *t*_minor_ = 9.27 min].

##### (*R*)-Ethyl 5-(3-Bromophenyl)-2-(hydroxymethyl)-1-(2-isopropylphenyl)-1*H*-pyrrole-3-carboxylate (**2f**)^16^

According to the general procedure for kinetic resolution, substrate **2f** (100 mg, 0.23 mmol) with catalyst (1*S*,3*S*)-**1** (3.46 mg, 0.0045 mmol) in acetonitrile (2.3 mL) at 24–25 °C afforded aldehyde **3f** and recovered **2f** in 5 h at 56% conversion. Isolated yield: 40% (40 mg). Physical state: brown colored sticky material, *R*_f_ = 0.48 (20% EtOAc/hexane). ^1^H NMR (400 MHz, CDCl_3_): δ 7.47–7.43 (m, 1H), 7.34–7.31 (m, 3H), 7.27–7.23 (m, 2H), 7.00–6.92 (m, 2H), 6.88 (s, 1H), 4.52–4.35 (m, 4H), 4.27 (t, *J* = 7.2 Hz, 1H), 2.34–2.24 (m, 1H), 1.42 (t, *J* = 7.2 Hz, 3H), 0.99 (d, *J* = 7.2 Hz, 3H), 0.68 (d, *J* = 6.8 Hz, 3H). ^13^C{^1^H} NMR (101 MHz, CDCl_3_): δ 166.5, 147.0, 142.2, 134.5, 134.0, 133.3, 131.1, 130.3, 130.0, 129.6, 129.3, 127.3, 126.7, 126.5, 122.2, 114.3, 110.4, 60.7, 55.6, 27.7, 24.6, 22.4, 14.6. IR (neat): 3457, 2963, 2868, 1677, 1491, 1240, 1075, 776 cm^–1^. HRMS (ES+) *m*/*z*: [M + Na]^+^ calcd for C_23_H_24_BrNO_3_Na, 464.0837; found, 464.0833. [α]_D_^20^ = −24.8 (*c* = 0.4, CHCl_3_). HPLC: *er* = 6.5:93.5. The *er* was determined by chiral HPLC analysis using a Lux 5 μm cellulose-1 (250 × 4.6 mm) column [hexane/IPA (80:20), 0.7 mL/min, λ = 280 nm, *t*_minor_ = 6.58 min, *t*_major_ = 7.77 min].

##### (*S*)-Ethyl 5-(3-Bromophenyl)-2-formyl-1-(2-isopropylphenyl)-1*H*-pyrrole-3-carboxylate (**3f**)^16^

Isolated yield: 48% (48 mg). Physical state: pale yellow solid, mp: 80–84 °C, *R*_f_ = 0.60 (20% EtOAc/hexane). ^1^H NMR (400 MHz, CDCl_3_): δ 10.40 (s, 1H), 7.46–7.41 (m, 1H), 7.35–7.22 (m, 5H), 7.05–6.98 (m, 3H), 4.42 (q, *J* = 7.2 Hz, 2H), 2.30–2.17 (m, 1H), 1.43 (t, *J* = 7.2 Hz, 3H), 0.97 (d, *J* = 7.2 Hz, 3H), 0.71 (d, *J* = 6.8 Hz, 3H). ^13^C{^1^H} NMR (101 MHz, CDCl_3_): δ 181.4, 163.7, 145.3, 139.4, 135.8, 133.4, 132.4, 131.9, 131.4, 130.0, 129.7, 128.6, 127.3, 127.0, 126.4, 124.9, 122.3, 113.3, 61.2, 28.0, 24.1, 22.5, 14.5. IR (neat): 2964, 2871, 1711, 1668, 1492, 1434, 1326, 1219, 756 cm^–1^. HRMS (ES+) *m*/*z*: [M + Na]^+^ calcd for C_23_H_22_BrNO_3_Na, 462.0681; found, 462.0681. [α]_D_^20^ = +15.1 (*c* = 0.35, CHCl_3_). HPLC: *er* = 81:19 (10 mg of aldehyde was reduced to the corresponding alcohol with NaBH_4_ and determined the *er*). The *er* was determined by chiral HPLC analysis using a Lux 5 μm cellulose-1 (250 × 4.6 mm) column [hexane/isopropanol (80:20), 0.7 mL/min, λ = 280 nm, *t*_major_ = 6.55 min, *t*_minor_ = 7.75 min].

##### (*R*)-Ethyl 5-(3-Bromophenyl)-1-(2-ethylphenyl)-2-(hydroxymethyl)-1*H*-pyrrole-3-carboxylate (**2g**)^16^

According to the general procedure for kinetic resolution, substrate **2g** (100 mg, 0.23 mmol) with catalyst (1*S*,3*S*)-**1** (3.57 mg, 0.0046 mmol) in acetonitrile (2.4 mL) at 23–25 °C afforded aldehyde **3g** and recovered **2g** in 5 h at 56% conversion. Isolated yield: 39% (39 mg). Physical state: brown colored sticky material, *R*_f_ = 0.33 (20% EtOAc/hexane). ^1^H NMR (400 MHz, CDCl_3_): δ 7.42 (td, *J* = 7.6, 1.6 Hz, 1H), 7.32–7.23 (m, 5H), 6.97 (t, *J* = 8.0 Hz, 1H), 6.91 (dt, *J* = 8.0, 1.6 Hz, 1H), 6.88 (s, 1H), 4.50–4.35 (m, 4H), 4.28 (t, *J* = 7.2 Hz, 1H), 2.25–2.06 (m, 2H), 1.41 (t, *J* = 7.2 Hz, 3H), 0.95 (t, *J* = 7.6 Hz, 3H). ^13^C{^1^H} NMR (101 MHz, CDCl_3_): δ 166.5, 142.1, 141.9, 135.5, 134.0, 132.9, 130.8, 129.97, 129.96, 129.7, 129.5, 129.3, 126.9, 126.1, 122.3, 114.4, 110.7, 60.6, 55.6, 23.5, 14.6, 13.6. IR (neat): 3454, 2972, 2935, 1677, 1594, 1437, 1241, 1079, 775 cm^–1^. HRMS (ES+) *m*/*z*: [M + Na]^+^ calcd for C_22_H_22_BrNO_3_Na, 450.0681; found, 450.0673. [α]_D_^20^ = −22.0 (*c* = 0.4, CHCl_3_). HPLC: *er* = 11.5:88.5. The *er* was determined by chiral HPLC analysis using a Lux 5 μm cellulose-1 (250 × 4.6 mm) column [hexane/isopropanol (80:20), 0.5 mL/min, λ = 280 nm, *t*_minor_ = 9.82 min, *t*_major_ = 10.99 min].

##### (*S*)-Ethyl 5-(3-Bromophenyl)-1-(2-ethylphenyl)-2-formyl-1*H*-pyrrole-3-carboxylate (**3g**)^16^

Isolated yield: 48% (48 mg). Physical state: pale yellow liquid, *R*_f_ = 0.56 (20% EtOAc/hexane). ^1^H NMR (400 MHz, CDCl_3_): δ 10.40 (s, 1H), 7.40 (td, *J* = 7.6, 1.2 Hz, 1H), 7.35–7.29 (m, 3H), 7.24 (dt, *J* = 7.5, 1.6 Hz, 1H), 7.13 (dd, *J* = 8.0, 1.2 Hz, 1H), 7.04–7.00 (m, 2H), 6.96 (dt, *J* = 8.0, 1.2 Hz, 1H), 4.42 (q, *J* = 7.2 Hz, 2H), 2.26–2.06 (m, 2H), 1.43 (t, *J* = 7.2 Hz, 3H), 0.98 (t, *J* = 7.2 Hz, 3H). ^13^C{^1^H} NMR (101 MHz, CDCl_3_): δ 181.4, 163.6, 140.7, 139.1, 136.8, 133.2, 132.4, 131.7, 131.4, 129.8, 129.7, 129.0, 128.4, 127.1, 126.6, 124.9, 122.4, 113.5, 61.2, 23.4, 14.5, 13.3. IR (neat): 2981, 2934, 1704, 1670, 1495, 1439, 1219, 769 cm^–1^. HRMS (ES+) *m*/*z*: [M + Na]^+^ calcd for C_22_H_20_BrNO_3_Na, 448.0524; found, 448.0525. [α]_D_^20^ = +27.3 (*c* = 0.4, CHCl_3_). HPLC: *er* = 82:18 (10 mg of aldehyde was reduced to the corresponding alcohol with NaBH_4_ and determined the *er*). The *er* was determined by chiral HPLC analysis using a Lux 5 μm cellulose-1 (250 × 4.6 mm) column [hexane/isopropanol (80:20), 0.5 mL/min, λ = 280 nm, *t*_major_ = 9.75 min, *t*_minor_ = 11.13 min].

##### (*R*)-Ethyl 5-(3-Bromophenyl)-2-(hydroxymethyl)-1-(*o*-tolyl)-1*H*-pyrrole-3-carboxylate (**2h**)^16^

According to the general procedure for kinetic resolution, substrate **2h** (100 mg, 0.24 mmol) with catalyst (1*S*,3*S*)-**1** (3.70 mg, 0.0046 mmol) in acetonitrile (2.4 mL) at 24–25 °C afforded aldehyde **3h** and recovered **2h** in 5 h at 55% conversion. Isolated yield: 42% (42 mg). Physical state: brown colored solid, mp: 68–71 °C, *R*_f_ = 0.32 (20% EtOAc/hexane). ^1^H NMR (400 MHz, CDCl_3_): δ 7.38–7.34 (td, *J* = 7.6, 1.6 Hz, 1H), 7.31–7.22 (m, 5H), 7.00–6.96 (td, *J* = 7.6, 0.4 Hz, 1H), 6.91–6.89 (m, 1H), 6.88 (s, 1H), 4.44 (d, *J* = 6.8 Hz, 2H), 4.37 (q, *J* = 6.8 Hz, 2H), 4.22 (t, *J* = 7.2 Hz, 1H), 1.88 (s, 3H), 1.41 (t, *J* = 7.1 Hz, 3H). ^13^C{^1^H} NMR (101 MHz, CDCl_3_): δ 166.4, 141.6, 136.7, 136.1, 134.0, 132.8, 131.4, 130.8, 130.0 (2C), 129.7, 129.2, 127.1, 126.1, 122.4, 114.5, 110.8, 60.6, 55.5, 17.4, 14.6. IR (neat): 3462, 2979, 1678, 1594, 1461, 1241, 1078, 775 cm^–1^. HRMS (ES+) *m*/*z*: [M + Na]^+^ calcd for C_21_H_20_BrNO_3_Na, 436.0524; found, 436.0515. [α]_D_^20^ = −7.3 (*c* = 0.4, CHCl_3_). HPLC: *er* = 12:88. The *er* was determined by chiral HPLC using a Lux 5 μm cellulose-1 (250 × 4.6 mm) column [hexane/isopropanol (90:10), 1.0 mL/min, λ = 280 nm, *t*_minor_ = 7.74 min, *t*_major_ = 8.70 min].

##### (*S*)-Ethyl 5-(3-Bromophenyl)-2-formyl-1-(*o*-tolyl)-1*H*-pyrrole-3-carboxylate (**3h**)^16^

Isolated yield: 47% (47 mg). Physical state: colorless sticky material, *R*_f_ = 0.52 (20% EtOAc/hexane). ^1^H NMR (400 MHz, CDCl_3_): δ 10.41 (s, 1H), 7.36–7.30 (m, 3H), 7.25–7.20 (m, 2H), 7.10 (d, *J* = 7.6 Hz, 1H), 7.03 (t, *J* = 8.0 Hz, 1H), 7.00 (s, 1H), 6.95 (ddd, *J* = 7.9, 1.6, 1.2 Hz, 1H), 4.42 (q, *J* = 7.2 Hz, 2H), 1.91 (s, 3H), 1.43 (t, *J* = 7.1 Hz, 3H). ^13^C{^1^H} NMR (101 MHz, CDCl_3_): δ 181.5, 163.6, 138.9, 137.5, 135.6, 132.9, 132.5, 131.7, 131.4, 130.9, 129.8, 129.4, 128.3, 127.0, 126.8, 125.0, 122.4, 113.5, 61.2, 17.4, 14.5. IR (neat): 2982, 2877, 1710, 1667, 1496, 1435, 1220, 1064, 771 cm^–1^. HRMS (ES+) *m*/*z*: [M + Na]^+^ calcd for C_21_H_18_BrNO_3_Na, 434.0368; found, 434.0366. [α]_D_^20^ = +23.3 (*c* = 0.35, CHCl_3_). HPLC: *er* = 78:22 (10 mg of aldehyde was reduced to the corresponding alcohol with NaBH_4_ and determined the *er*). The *er* was determined by chiral HPLC using a Lux 5 μm cellulose-1 (250 × 4.6 mm) column [hexane/isopropanol (90:10), 1.0 mL/min, λ = 280 nm, *t*_major_ = 7.69 min, *t*_minor_ = 8.63 min].

##### (*R*)-Ethyl 5-(3-Bromophenyl)-1-(2,3-dihydro-1*H*-inden-4-yl)-2-(hydroxymethyl)-1*H*-pyrrole-3-carboxylate (**2i**)^16^

According to the general procedure for kinetic resolution, substrate **2i** (100 mg, 0.23 mmol) with catalyst (1*S*,3*S*)-**1** (3.59 mg, 0.0046 mmol) in acetonitrile (2.4 mL) at 23–25 °C afforded aldehyde **3i** and recovered **2i** in 5 h at 46% conversion. Isolated yield: 50% (50 mg). Physical state: brown colored sticky material, *R*_f_ = 0.31 (20% EtOAc/hexane). ^1^H NMR (400 MHz, CDCl_3_): δ 7.30–7.20 (m, 4H), 7.02 (d, *J* = 7.6 Hz, 1H), 6.98 (t, *J* = 7.6 Hz, 1H), 6.90–6.88 (m, 1H), 6.85 (s, 1H), 4.49 (d, *J* = 7.2 Hz, 2H), 4.37 (q, *J* = 7.2 Hz, 2H), 4.25 (t, *J* = 7.2 Hz, 1H), 2.98–2.83 (m, 2H), 2.62–2.54 (m, 1H), 2.33–2.22 (m, 1H), 2.04–1.93 (m, 1H), 1.91–1.82 (m, 1H), 1.41 (t, *J* = 7.1 Hz, 3H). ^13^C{^1^H} NMR (101 MHz, CDCl_3_): δ 166.4, 146.8, 142.7, 141.5, 134.2, 133.4, 132.7, 130.7, 129.9, 129.7, 127.6, 126.2, 126.1, 125.5, 122.3, 114.4, 110.8, 60.6, 55.6, 33.3, 30.6, 25.1, 14.6. IR (neat): 3441, 2943, 2844, 1677, 1477, 1243, 753 cm^–1^. HRMS (ES+) *m*/*z*: [M + Na]^+^ calcd for C_23_H_22_BrNO_3_Na, 462.0681; found, 462.0679; [α]_D_^20^ = +29.1 (*c* = 0.5, CHCl_3_). HPLC: *er* = 26:74. The *er* was determined by chiral HPLC using a Lux 5 μm cellulose-1 (250 × 4.6 mm) column [hexane/IPA (90:10), 1.0 mL/min, λ = 280 nm, *t*_minor_ = 5.93 min, *t*_major_ = 8.03 min].

##### (*S*)-Ethyl 5-(3-Bromophenyl)-1-(2,3-dihydro-1*H*-inden-4-yl)-2-formyl-1*H*-pyrrole-3-carboxylate (**3i**)^16^

Isolated yield: 40% (40 mg). Physical state: pale yellow sticky material, *R*_f_ = 0.51 (20% EtOAc/hexane). ^1^H NMR (400 MHz, CDCl_3_): δ 10.40 (s, 1H), 7.34 (ddd, *J* = 8.0, 2.0, 1.1 Hz, 1H), 7.29–7.26 (m, 2H), 7.16 (t, *J* = 7.6 Hz, 1H), 7.03 (t, *J* = 8.0 Hz, 1H), 6.97 (s, 1H), 6.96–6.90 (m, 2H), 4.41 (q, *J* = 7.2 Hz, 2H), 3.02–2.86 (m, 2H), 2.61–2.53 (m, 1H), 2.37–2.29 (m, 1H), 2.08–1.98 (m, 1H), 1.94–1.83 (m, 1H), 1.42 (t, *J* = 7.2 Hz, 3H). ^13^C{^1^H} NMR (101 MHz, CDCl_3_): δ 181.5, 163.7, 146.2, 141.9, 138.8, 134.6, 132.8, 132.6, 131.7, 131.3, 129.8, 127.2, 127.0, 125.42, 125.36, 125.0, 122.4, 113.5, 61.1, 33.3, 30.7, 25.5, 14.5. IR (neat): 2939, 2844, 1711, 1677, 1433, 1223, 1092, 772 cm^–1^. HRMS (ES+) *m*/*z*: [M + Na]^+^ calcd for C_23_H_20_BrNO_3_Na, 460.0524; found, 460.0523. [α]_D_^20^ = −10.4 (*c* = 0.30, CHCl_3_). HPLC: *er* = 81:19 (10 mg of aldehyde was reduced to the corresponding alcohol with NaBH_4_ and determined the *er*). The *er* was determined by chiral HPLC using a Lux 5 μm cellulose-1 (250 × 4.6 mm) column [hexane/isopropanol (90:10), 1.0 mL/min, λ = 280 nm, *t*_major_ = 5.92 min, *t*_minor_ = 7.56 min].

##### (*R*)-Ethyl 1-([1,1′-Biphenyl]-2-yl)-5-(3-bromophenyl)-2-(hydroxymethyl)-1*H*-pyrrole-3-carboxylate (**2j**)^16^

According to the general procedure for kinetic resolution, substrate **2j** (100 mg, 0.21 mmol) with catalyst (1*S*,3*S*)-**1** (3.22 mg, 0.0042 mmol) in acetonitrile (2.1 mL) at 23–25 °C afforded aldehyde **3j** and recovered **2j** in 24 h at 55% conversion. Isolated yield: 42% (42 mg). Physical state: brown colored sticky material, *R*_f_ = 0.24 (20% EtOAc/hexane). ^1^H NMR (400 MHz, CDCl_3_): δ 7.52–7.48 (m, 3H), 7.36–7.34 (m, 1H), 7.21–7.19 (m, 2H), 7.10 (t, *J* = 6.8 Hz, 2H), 6.84 (t, *J* = 8.0 Hz, 1H), 6.67 (t, *J* = 1.6 Hz, 1H), 6.57–6.55 (m, 3H), 6.49 (ddd, *J* = 7.8, 1.6, 1.0 Hz, 1H), 4.66 (AB q, *J* = 14.0 Hz, 2H), 4.40–4.31 (m, 3H), 1.40 (t, *J* = 7.2 Hz, 3H). ^13^C{^1^H} NMR (101 MHz, CDCl_3_): δ 166.5, 141.5, 140.5, 137.5, 134.3, 134.0, 133.3, 131.3, 131.1, 129.8, 129.7, 129.6, 129.1, 128.4, 128.3 (2C), 128.0 (2C), 127.5, 126.2, 121.9, 114.6, 110.5, 60.6, 55.7, 14.5. IR (neat): 3454, 2924, 2854, 1676, 1597, 1438, 1236, 1078, 776, 697 cm^–1^. HRMS (ES+) *m*/*z*: [M + Na]^+^ calcd for C_26_H_22_BrNO_3_Na, 498.0681; found, 498.0674. [α]_D_^20^ = −51.5 (*c* = 0.4, CHCl_3_). HPLC: *er* = 11:89. The *er* was determined by chiral HPLC using a Lux 5 μm cellulose-1 (250 × 4.6 mm) column [hexane/isopropanol (80:20), 0.7 mL/min, λ = 254 nm, *t*_minor_ = 7.57 min, *t*_major_ = 10.12 min].

##### (*S*)-Ethyl 1-([1,1′-Biphenyl]-2-yl)-5-(3-bromophenyl)-2-formyl-1*H*-pyrrole-3-carboxylate (**3j**)^16^

Isolated yield: 48% (48 mg). Physical state: colorless solid, mp: 78–83 °C, *R*_f_ = 0.46 (20% EtOAc/hexane). ^1^H NMR (400 MHz, CDCl_3_): δ 10.48 (s, 1H), 7.52–7.41 (m, 3H), 7.30 (d, *J* = 7.6 Hz, 2H), 7.17 (d, *J* = 7.6 Hz, 1H), 7.10 (t, *J* = 7.6 Hz, 2H), 6.91 (t, *J* = 8.0 Hz, 1H), 6.70 (s, 1H), 6.68 (s, 1H), 6.56–6.53 (m, 3H), 4.39 (q, *J* = 7.2 Hz, 2H), 1.41 (t, *J* = 7.2 Hz, 3H). ^13^C{^1^H} NMR (101 MHz, CDCl_3_): δ 181.8, 163.6, 139.6, 139.2, 137.8, 135.5, 133.2, 132.3, 131.8, 131.0, 130.9, 129.6, 129.4, 129.3, 128.2 (2C), 128.14 (2C), 128.08, 127.4, 127.0, 125.2, 122.0, 113.3, 61.1, 14.5. IR (neat): 2978, 2879, 1708, 1663, 1439, 1213, 1069, 753 cm^–1^. HRMS (ES+) *m*/*z*: [M + Na]^+^ calcd for C_26_H_20_BrNO_3_Na, 496.0524; found, 496.0522. [α]_D_^20^ = −23.7 (*c* = 0.4, CHCl_3_). HPLC: *er* = 76:24 (10 mg of aldehyde was reduced to the corresponding alcohol with NaBH_4_ and determined the *er*). The *er* was determined by chiral HPLC using a Lux 5 μm cellulose-1 (250 × 4.6 mm) column [hexane/isopropanol (80:20), 0.7 mL/min, λ = 280 nm, *t*_major_ = 7.69 min, *t*_minor_ = 10.14 min].

##### (*R*)-Ethyl 1-(2-(*tert*-Butyl)phenyl)-2-(hydroxymethyl)-5-phenyl-1*H*-pyrrole-3-carboxylate (**2k**)^16^

According to the general procedure for kinetic resolution, substrate **2k** (100 mg, 0.26 mmol) with catalyst (1*S*,3*S*)-**1** (4.06 mg, 0.0053 mmol) in acetonitrile (2.7 mL) at 23–25 °C afforded aldehyde **3k** and recovered **2k** in 6 h at 54% conversion. Isolated yield: 43% (43 mg). Physical state: brown sticky material, *R*_f_ = 0.40 (20% EtOAc/hexane). ^1^H NMR (400 MHz, CDCl_3_): δ 7.46 (dd, *J* = 8.4, 1.6 Hz, 1H), 7.35 (td, *J* = 7.2, 1.6 Hz, 1H), 7.21 (td, *J* = 8.0, 1.6 Hz, 1H), 7.10–7.03 (m, 4H), 7.00–6.97 (m, 2H), 6.78 (s, 1H), 4.46–4.25 (m, 5H), 1.34 (t, *J* = 7.2 Hz, 3H), 0.88 (s, 9H). ^13^C{^1^H} NMR (101 MHz, CDCl_3_): δ 166.8, 147.6, 142.6, 135.5, 134.4, 132.5, 132.1, 130.3, 129.7, 128.3 (2C), 127.9 (2C), 127.0, 126.8, 114.0, 110.0, 60.6, 56.2, 36.3, 31.3, 14.6. IR (neat): 3445, 2967, 2908, 1673, 1478, 1238, 1083, 755 cm^–1^. HRMS (ES+) *m*/*z*: [M + Na]^+^ calcd for C_24_H_27_NO_3_Na, 400.1889; found, 400.1886. [α]_D_^20^ = −30.0 (*c* = 0.4, CHCl_3_). HPLC: *er* = 13.5:86.5. The *er* was determined by chiral HPLC using a Lux 5 μm cellulose-1 (250 × 4.6 mm) column [hexane/isopropanol (80:20), 0.5 mL/min, λ = 280 nm, *t*_minor_ = 9.21 min, *t*_major_ = 10.88 min].

##### (*S*)-Ethyl 1-(2-(*tert*-Butyl)phenyl)-2-formyl-5-phenyl-1*H*-pyrrole-3-carboxylate (**3k**)^16^

Isolated yield: 48% (48 mg). Physical state: pale yellow liquid, *R*_f_ = 0.52 (20% EtOAc/hexane). ^1^H NMR (400 MHz, CDCl_3_): δ 10.41 (s, 1H), 7.50 (dd, *J* = 8.4, 1.6 Hz, 1H), 7.40 (td, *J* = 7.2, 1.6 Hz, 1H), 7.26–7.17 (m, 4H), 7.10–7.06 (m, 3H), 6.99 (s, 1H), 4.41 (q, *J* = 6.8 Hz, 2H), 1.43 (t, *J* = 7.2 Hz, 3H), 0.93 (s, 9H). ^13^C{^1^H} NMR (101 MHz, CDCl_3_): δ 181.6, 163.8, 146.0, 141.7, 135.7, 134.1, 130.9, 130.9, 130.2, 129.3, 128.8 (2C), 128.42 (2C), 128.39, 126.4, 125.0, 113.1, 61.1, 36.3, 31.3, 14.5. IR (neat): 2969, 2907, 1711, 1665, 1466, 1213, 1092, 757 cm^–1^. HRMS (ES+) *m*/*z*: [M + Na]^+^ calcd for C_24_H_25_NO_3_Na, 398.1732; found, 398.1728. [α]_D_^20^ = +43.4 (*c* = 0.4, CHCl_3_). HPLC: er = 81.5:18.5 (10 mg of aldehyde was reduced to the corresponding alcohol with NaBH_4_ and determined the *er*). The *er* was determined by chiral HPLC using a Lux 5 μm cellulose-1 (250 × 4.6 mm) column [hexane/isopropanol (80:20), 0.5 mL/min, λ = 280 nm, *t*_major_ = 9.12 min, *t*_minor_ = 10.86 min].

##### (*R*)-Ethyl 1-(2-Bromophenyl)-2-(hydroxymethyl)-5-phenyl-1*H*-pyrrole-3-carboxylate (**2l**)^16^

According to the general procedure for kinetic resolution, substrate **2l** (100 mg, 0.26 mmol) with catalyst (1*S*,3*S*)-**1** (3.96 mg, 0.0052 mmol) in acetonitrile (2.6 mL) at 24–25 °C afforded aldehyde **3l** and recovered **2l** in 8 h at 53% conversion. Isolated yield: 42% (42 mg). Physical state: colorless sticky material, *R*_f_ = 0.19 (20% EtOAc/hexane). ^1^H NMR (400 MHz, CDCl_3_): δ 7.74 (d, *J* = 8.0 Hz, 1H), 7.46–7.36 (m, 3H), 7.28–7.19 (m, 5H), 6.93 (s, 1H), 4.83 (d, *J* = 12.0 Hz, 1H), 4.47 (q, *J* = 7.2 Hz, 2H), 4.37–4.32 (m, 2H), 1.51 (t, *J* = 7.2 Hz, 3H). ^13^C{^1^H} NMR (101 MHz, CDCl_3_): δ 166.5, 141.1, 136.9, 134.8, 133.5, 131.8, 131.2, 130.8, 128.5 (3C), 128.3 (2C), 127.4, 124.1, 114.6, 110.3, 60.6, 55.8, 14.6. IR (neat): 3455, 2980, 1674, 1480, 1239, 1093, 757 cm^–1^; HRMS (ES+) *m*/*z*: [M + Na]^+^ calcd for C_20_H_18_BrNO_3_Na, 422.0368; found, 422.0368. [α]_D_^20^ = +39.5 (*c* = 0.4, CHCl_3_). HPLC: *er* = 12:88. The *er* was determined by chiral HPLC using a Lux 5 μm cellulose-1 (250 × 4.6 mm) column [hexane/isopropanol (80:20), 0.7 mL/min, λ = 280 nm, *t*_minor_ = 7.99 min, *t*_major_ = 9.60 min].

##### (*S*)-Ethyl 1-(2-Bromophenyl)-2-formyl-5-phenyl-1*H*-pyrrole-3-carboxylate (**3l**)^16^

Isolated yield: 45% (45 mg). Physical state: pale yellow liquid, *R*_f_ = 0.40 (20% EtOAc/hexane). ^1^H NMR (400 MHz, CDCl_3_): δ 10.44 (s, 1H), 7.63 (dd, *J* = 7.6, 1.2 Hz, 1H), 7.33–7.20 (m, 8H), 7.00 (s, 1H), 4.45 (q, *J* = 6.8 Hz, 2H), 1.46 (t, *J* = 6.8 Hz, 3H). ^13^C{^1^H} NMR (101 MHz, CDCl_3_): δ 181.3, 163.7, 141.0, 138.2, 133.2, 132.6, 130.4, 130.2, 130.0, 129.1 (2C), 128.7, 128.4 (2C), 128.0, 125.2, 123.2, 113.3, 61.1, 14.5. IR (neat): 2983, 2884, 1710, 1664, 1468, 1218, 1083, 754 cm^–1^. HRMS (ES+) *m*/*z*: [M + Na]^+^ calcd for C_20_H_16_BrNO_3_Na, 420.0211; found, 420.0207. [α]_D_^20^ = +56.3 (*c* = 0.4, CHCl_3_). HPLC: *er* = 80.5:19.5 (10 mg of aldehyde was reduced to the corresponding alcohol with NaBH_4_ and determined the *er*). The *er* was determined by chiral HPLC using a Lux 5 μm cellulose-1 (250 × 4.6 mm) column [hexane/isopropanol (80:20), 0.7 mL/min, λ = 280 nm, *t*_major_ = 8.38 min, *t*_minor_ = 9.89 min].

##### (*R*)-Ethyl 1-([1,1′-Biphenyl]-2-yl)-2-(hydroxymethyl)-5-phenyl-1*H*-pyrrole-3-carboxylate (**2m**)^16^

According to the general procedure for kinetic resolution, substrate **2m** (100 mg, 0.25 mmol) with catalyst (1*S*,3*S*)-**1** (3.86 mg, 0.0050 mmol) in acetonitrile (2.5 mL) at 24–25 °C afforded aldehyde **3m** and recovered **2m** in 7 h at 49% conversion. Isolated yield: 44% (44 mg). Physical state: off-white solid, mp: 97–100 °C, *R*_f_ = 0.24 (20% EtOAc/hexane). ^1^H NMR (400 MHz, CDCl_3_): δ 7.49–7.44 (m, 3H), 7.36–7.33 (m, 1H), 7.18–7.14 (m, 1H), 7.10–7.06 (m, 3H), 7.03–6.98 (m, 2H), 6.64–6.60 (m, 4H), 6.58 (s, 1H), 4.64 (dd, *J* = 13.6, 3.2 Hz, 1H), 4.54 (dd, *J* = 13.6, 6.0 Hz, 1H), 4.41–4.26 (m, 3H), 1.38 (t, *J* = 7.2 Hz, 3H). ^13^C{^1^H} NMR (101 MHz, CDCl_3_): δ 166.7, 141.1, 140.6, 137.8, 134.9, 134.7, 132.0, 131.2, 129.9, 129.6, 128.3, 128.2 (2C), 128.1 (2C), 127.9 (2C), 127.8 (2C), 127.3, 126.7, 114.3, 110.0, 60.5, 55.8, 14.5. IR (neat): 3495, 2925, 1666, 1482, 1249, 1080, 1006, 740, 698 cm^–1^. HRMS (ESI-) *m*/*z*: [M + Na]^+^ calcd for C_26_H_23_NO_3_Na, 420.1576; found, 420.1580. [α]_D_^20^ = −33.8 (*c* = 0.4, CHCl_3_). HPLC: *er* = 10.5:89.5. The *er* was determined by chiral HPLC using a Lux 5 μm cellulose-1 (250 × 4.6 mm) column [hexane/isopropanol (80:20), 1 mL/min, λ = 280 nm, *t*_minor_ = 7.55 min, *t*_major_ = 9.90 min].

##### (*S*)-Ethyl 1-([1,1′-Biphenyl]-2-yl)-2-formyl-5-phenyl-1*H*-pyrrole-3-carboxylate(**3m**)^16^

Isolated yield: 42% (42 mg). Physical state: green colored sticky material, *R*_f_ = 0.45 (20% EtOAc/hexane). ^1^H NMR (400 MHz, CDCl_3_): δ 10.43 (s, 1H), 7.50–7.40 (m, 3H), 7.30–7.26 (m, 1H), 7.20–7.13 (m, 2H), 7.09–7.05 (m, 4H), 6.71 (s, 1H), 6.69 (dd, *J* = 8.4, 1.2 Hz, 2H), 6.59 (dd, *J* = 8.4, 1.2 Hz, 2H), 4.42–4.32 (m, 2H), 1.40 (t, *J* = 7.2 Hz, 3H). ^13^C{^1^H} NMR (101 MHz, CDCl_3_): δ 181.7, 163.8, 141.3, 139.4, 138.1, 135.9, 133.0, 130.9, 130.4, 129.5, 129.4, 128.7 (2C), 128.2 (2C), 128.11, 128.07 (2C), 128.0 (2C), 127.9, 127.2, 125.2, 113.1, 61.0, 14.5. IR (neat): 2984, 1710, 1664, 1213, 751, 696 cm^–1^. HRMS (ES+) *m*/*z*: [M + Na]^+^ calcd for C_26_H_21_NO_3_Na, 418.1419; found, 418.1411. [α]_D_^20^ = +13.5 (*c* = 0.3, CHCl_3_). HPLC: *er* = 65.5:34.5 (10 mg of aldehyde was reduced to the corresponding alcohol with NaBH_4_ and determined the *er*). The *er* was determined by chiral HPLC using a Lux 5 μm cellulose-1 (250 × 4.6 mm) column [hexane/isopropanol (80:20), 0.7 mL/min, λ = 280 nm, *t*_major_ = 7.46 min, *t*_minor_ = 9.77 min].

##### (*R*)-Ethyl 1-([1,1′-Biphenyl]-2-yl)-5-(benzo[*d*][1,3]dioxol-5-yl)-2-(hydroxymethyl)-1*H*-pyrrole-3-carboxylate-(**2n**)^16^

According to the general procedure for kinetic resolution, substrate **2n** (100 mg, 0.22 mmol) with catalyst (1*S*,3*S*)-**1** (3.47 mg, 0.0045 mmol) in acetonitrile (2.3 mL) at 23–25 °C afforded aldehyde **3n** and recovered **2n** in 24 h at 41% conversion. Isolated yield: 53% (53 mg). Physical state: colorless oil, *R*_f_ = 0.29 (20% EtOAc/hexane). ^1^H NMR (400 MHz, CDCl_3_): δ 7.52–7.35 (m, 4H), 7.18 (ddd, *J* = 6.3, 3.6, 1.2 Hz, 1H), 7.15–7.08 (m, 2H), 6.67 (d, *J* = 7.2 Hz, 2H), 6.50–6.42 (m, 2H), 6.11–6.04 (m, 2H), 5.85 (s, 2H), 4.58 (AB q, *J* = 13.6 Hz, 2H), 4.40–4.18 (m, 3H), 1.38 (t, *J* = 7.2 Hz, 3H). ^13^C{^1^H}NMR (101 MHz, CDCl_3_): δ 166.6, 147.1, 146.5, 140.8, 140.5, 137.8, 134.6, 131.2, 129.9, 129.6, 128.3, 128.2 (2C), 128.1 (2C), 127.4, 126.0, 122.0, 114.2, 109.7, 108.7, 107.8, 101.0, 60.5, 55.8, 14.5. IR (neat): 3419, 2978, 1675, 1478, 1225, 1034, 745 cm^–1^. HRMS (ES+) *m*/*z*: [M + Na]^+^ calcd for C_27_H_23_NO_5_Na, 464.1474; found, 464.1474. [α]_D_^20^ = −6.6 (*c* = 0.53, CHCl_3_). HPLC: *er* = 26:74. The *er* was determined by chiral HPLC with a Lux 5 μm cellulose-1 column [hexane/isopropanol (80:20), 0.7 mL/min, λ = 220 nm, *t*_minor_ = 9.87 min, *t*_major_ = 13.54 min].

##### (*S*)-Ethyl 1-([1,1′-Biphenyl]-2-yl)-5-(benzo[*d*][1,3]dioxol-5-yl)-2-formyl-1*H*-pyrrole-3-carboxylate-(**3n**)^16^

Isolated yield: 36% (36 mg). Physical state: colorless oil, *R*_f_ = 0.40 (20% EtOAc/hexane). ^1^H NMR (400 MHz, CDCl_3_): δ 10.39 (s, 1H), 7.53–7.31 (m, 4H), 7.16 (d, *J* = 6.8 Hz, 1H), 7.10 (t, *J* = 7.2 Hz, 2H), 6.66 (d, *J* = 7.8 Hz, 2H), 6.62 (s, 1H), 6.53 (d, *J* = 8.0 Hz, 1H), 6.17 (d, *J* = 8.4 Hz, 1H), 6.11 (s, 1H), 5.90 (s, 2H), 4.37 (q, *J* = 7.2 Hz, 2H), 1.39 (t, *J* = 7.2 Hz, 3H). ^13^C{^1^H} NMR (101 MHz, CDCl_3_): δ 181.5, 163.8, 147.6, 147.3, 141.0, 139.3, 138.1, 135.9, 132.8, 130.9, 129.5, 129.4, 128.2 (2C), 128.1 (2C), 128.0, 127.3, 125.2, 124.2, 123.0, 112.9, 109.0, 108.0, 101.3, 61.1, 14.5. IR (neat): 2920, 2852, 1707, 1656, 1459, 1335, 1216, 916 cm^–1^. HRMS (ES+) *m*/*z*: [M + Na]^+^ calcd for C_27_H_21_NO_5_Na, 462.1317; found, 462.1317; [α]_D_^20^ = −15.3 (*c* = 0.3, CHCl_3_). HPLC: *er* = 81.5:18.5 (10 mg of aldehyde was reduced to the corresponding alcohol with NaBH4 and determined the *er*). The *er* was determined by chiral HPLC with a Lux 5 μm cellulose-1 column [hexane/isopropanol (80:20), 0.7 mL/min, λ = 220 nm, *t*_major_ = 10.14 min, *t*_minor_ = 14.06 min].

##### (*R*)-Ethyl 1-([1,1′-Biphenyl]-2-yl)-2-(hydroxymethyl)-5-(3-nitrophenyl)-1*H*-pyrrole-3-carboxylate (**2o**)^16^

According to the general procedure for kinetic resolution, substrate **2o** (100 mg, 0.23 mmol) with catalyst (1*S*,3*S*)-**1** (3.47 mg, 0.0045 mmol) in acetonitrile (2.3 mL) at 23–25 °C afforded aldehyde **3o** and recovered **2o** in 36 h at 40% conversion. Isolated yield: 58% (58 mg). Physical state: off-white solid, mp: 89–92 °C, *R*_f_ = 0.20 (20% EtOAc/hexane). ^1^H NMR (400 MHz, CDCl_3_): δ 7.92 (ddd, *J* = 8.4, 2.4, 0.8 Hz, 1H), 7.56–7.51 (m, 3H), 7.35–7.32 (m, 2H), 7.18–7.12 (m, 2H), 7.04 (t, *J* = 8.0 Hz, 2H), 6.85 (ddd, *J* = 8.0, 1.6, 1.2 Hz, 1H), 6.66 (s, 1H), 6.50–6.48 (m, 2H), 4.77 (dd, *J* = 13.7, 6.7 Hz, 1H), 4.66 (dd, *J* = 13.7, 7.6 Hz, 1H), 4.43–4.31 (m, 2H), 4.24 (t, *J* = 7.2 Hz, 1H), 1.41 (t, *J* = 6.8 Hz, 3H). ^13^C{^1^H} NMR (101 MHz, CDCl_3_): δ 166.3, 147.9, 141.9, 140.2, 137.3, 133.9, 133.6, 133.2, 132.3, 131.4, 130.2, 129.7, 128.8, 128.6, 128.3 (2C), 127.9 (2C), 127.7, 122.7, 121.4, 114.9, 111.2, 60.8, 55.6, 14.5. IR (neat): 3441, 2981, 1677, 1514, 1345, 1248, 1078, 735 cm^–1^. HRMS (ES+) *m*/*z*: [M + Na]^+^ calcd for C_26_H_22_N_2_O_5_Na, 465.1426; found, 465.1427. [α]_D_^20^ = −26.9 (*c* = 0.55, CHCl_3_). HPLC: *er* = 29:71. The *er* was determined by chiral HPLC with a Lux 5 μm cellulose-1 column [hexane/isopropanol (80:20), 0.7 mL/min, λ = 220 nm, *t*_minor_ = 11.21 min, *t*_major_ = 15.17 min].

##### (*S*)-Ethyl 1-([1,1′-Biphenyl]-2-yl)-2-formyl-5-(3-nitrophenyl)-1*H*-pyrrole-3-carboxylate (**3o**)^16^

Isolated yield: 33% (33 mg). Physical state: pale yellow white solid, mp: 158–161 °C, *R*_f_ = 0.33 (20% EtOAc/hexane). ^1^H NMR (400 MHz, CDCl_3_): δ 10.53 (s, 1H), 8.03 (ddd, *J* = 8.4, 2.4, 1.2 Hz, 1H), 7.54–7.46 (m, 3H), 7.42 (t, *J* = 1.6 Hz, 1H), 7.30–7.28 (m, 1H), 7.22 (t, *J* = 8.0 Hz, 1H), 7.18–7.14 (m, 1H), 7.07–7.03 (m, 2H), 6.91 (ddd, *J* = 7.6, 1.6, 0.8 Hz, 1H), 6.77 (s, 1H), 6.51–6.48 (m, 2H), 4.42 (q, *J* = 7.2 Hz, 2H), 1.42 (t, *J* = 7.2 Hz, 3H). ^13^C{^1^H} NMR (101 MHz, CDCl_3_): δ 182.0, 163.5, 147.9, 138.9, 138.3, 137.6, 135.1, 134.1, 133.6, 132.1, 131.0, 130.0, 129.5, 128.9, 128.4, 128.3 (2C), 128.0 (2C), 127.7, 125.2, 123.7, 122.7, 113.7, 61.3, 14.5. IR (neat): 2984, 1710, 1665, 1502, 1347, 1213, 1070, 735 cm^–1^. HRMS (ES+) *m*/*z*: [M + Na]^+^ calcd for C_26_H_20_N_2_O_5_Na, 463.1270; found, 463.1270. [α]_D_^20^ = −17.9 (*c* = 0.35, CHCl_3_). HPLC: *er* = 85:15 (10 mg of aldehyde was reduced to the corresponding alcohol with NaBH_4_ and determined the *er*). The *er* was determined by chiral HPLC using a Lux 5 μm cellulose-1 (250 × 4.6 mm) column [hexane/isopropanol (80:20), 0.7 mL/min, λ = 220 nm, *t*_major_ = 11.69 min, *t*_minor_ = 15.92 min].

##### (*R*)-Ethyl 1-([1,1′-Biphenyl]-2-yl)-2-(hydroxymethyl)-5-(naphthalen-2-yl)-1*H*-pyrrole-3-carboxylate (**2p**)^16^

According to the general procedure for kinetic resolution, substrate **2p** (100 mg, 0.22 mmol) with catalyst (1*S*,3*S*)-**1** (3.42 mg, 0.0044 mmol) in a mixture (1:1) of acetonitrile (2.3 mL) and ethyl acetate (2.3 mL) at 23–25 °C afforded aldehyde **3p** and recovered **2p** in 48 h at 46% conversion. Isolated yield: 50% (50 mg). Physical state: off-white solid, mp: 129–132 °C, *R*_f_ = 0.23 (20% EtOAc/hexane). ^1^H NMR (400 MHz, CDCl_3_): δ 7.72–7.69 (m, 1H), 7.52–7.45 (m, 5H), 7.41–7.37 (m, 2H), 7.32–7.30 (m, 1H), 7.18–7.14 (m, 1H), 7.04–7.00 (m, 3H), 6.83 (dd, *J* = 8.4, 1.6 Hz, 1H), 6.70 (s, 1H), 6.54 (dd, *J* = 8.4, 1.2 Hz, 2H), 4.65 (ddd, *J* = 21.4, 13.6, 6.0 Hz, 2H), 4.43–4.30 (m, 3H), 1.41 (t, *J* = 7.2 Hz, 3H). ^13^C{^1^H} NMR (101 MHz, CDCl_3_): δ 166.7, 141.3, 140.6, 137.7, 134.84, 134.77, 133.1, 132.1, 131.3, 129.9, 129.6, 129.4, 128.4, 128.2 (2C), 128.1 (2C), 128.09, 127.5, 127.4, 127.3, 126.9, 126.1, 126.0, 125.9, 114.5, 110.5, 60.6, 55.8, 14.6. IR (neat): 3525, 3056, 2926, 1677, 1482, 1228, 1078, 739 cm^–1^. HRMS (ES+) *m*/*z*: [M + Na]^+^ calcd for C_30_H_25_NO_3_Na, 470.1732; found, 470.1735. [α]_D_^20^ = +9.7 (*c* = 0.50, CHCl_3_). HPLC: *er* = 24.5:75.5. The *er* was determined by chiral HPLC with a Lux 5 μm cellulose-1 column [hexane/isopropanol (80:20), 0.7 mL/min, λ = 280 nm, *t*_minor_ = 7.85 min, *t*_major_ = 11.89 min].

##### (*S*)-Ethyl 1-([1,1′-Biphenyl]-2-yl)-2-formyl-5-(naphthalen-2-yl)-1*H*-pyrrole-3-carboxylate (**3p**)^16^

Isolated yield: 39% (39 mg). Physical state: off-white solid, mp: 113–117 °C, *R*_f_ = 0.46 (20% EtOAc/hexane). ^1^H NMR (400 MHz, CDCl_3_): δ 10.45 (s, 1H), 7.73 (d, *J* = 8.0 Hz, 1H), 7.54–7.43 (m, 7H), 7.25–7.24 (m, 1H), 7.13–7.08 (m, 1H), 7.09 (s, 1H), 7.00 (t, *J* = 8.0 Hz, 2H), 6.84–6.81 (m, 2H), 6.51 (dd, *J* = 8.4, 1.2 Hz, 2H), 4.40 (qd, *J* = 7.2, 1.6 Hz, 2H), 1.41 (t, *J* = 7.1 Hz, 3H). ^13^C{^1^H} NMR (101 MHz, CDCl_3_): δ 181.7, 163.8, 141.3, 139.5, 138.1, 136.0, 133.1, 132.8, 132.6, 130.9, 129.5, 129.4, 128.5, 128.3, 128.2 (2C), 128.1 (2C), 128.0, 127.8, 127.6, 127.5, 127.2, 126.7, 126.4, 125.9, 125.3, 113.5, 61.1, 14.5. IR (neat): 3056, 2924, 1710, 1663, 1482, 1213, 1069, 750 cm^–1^. HRMS (ES+) *m*/*z*: [M + Na]^+^ calcd for C_30_H_23_NO_3_Na, 468.1576; found, 468.1573. [α]_D_^20^ = −31.4 (*c* = 0.30, CHCl_3_). HPLC: *er* = 80:20 (10 mg of aldehyde was reduced to the corresponding alcohol with NaBH_4_ and determined the *er*). The *er* was determined by chiral HPLC using a Lux 5 μm cellulose-1 (250 × 4.6 mm) column [hexane/isopropanol (80:20), 0.7 mL/min, λ = 280 nm, *t*_major_ = 7.92 min, *t*_minor_ = 11.94 min].

##### (*R*)-(5-Phenyl-1-(2-(trifluoromethyl)phenyl)-1*H*-pyrrol-2-yl)methanol (**2q**)^16^

According to the general procedure for kinetic resolution, substrate **2q** (100 mg, 0.31 mmol) with catalyst (1*S*,3*S*)-**1** (4.83 mg, 0.0063 mmol) in acetonitrile (3.2 mL) at 23–25 °C afforded aldehyde **3q** and recovered **2q** in 2.5 h at 51% conversion. Isolated yield: 30% (30 mg). Physical state: colorless sticky material, *R*_f_ = 0.23 (20% EtOAc/hexane). ^1^H NMR (400 MHz, CDCl_3_): δ 7.70–7.61 (dd, *J* = 7.6, 1.2 Hz, 1H), 7.60–7.50 (m, 2H), 7.46 (m, 1H), 7.14–7.00 (m, 5H), 6.39 (d, *J* = 3.6 Hz, 1H), 6.33 (d, *J* = 4.0 Hz, 1H), 4.38 (d, *J* = 13.2 Hz, 1H), 4.15 (d, *J* = 13.2 Hz, 1H). ^13^C{^1^H} NMR (101 MHz, CDCl_3_): δ 137.2, 136.6, 135.9, 132.9, 132.4, 129.2, 129.0, 128.7, 128.1, 127.7, 127.4 (q, *J* = 5.1 Hz), 126.5, 122.8 (q, *J* = 275.7 Hz), 109.7, 108.9, 56.6. ^19^F NMR (376 MHz, CDCl_3_): −60.9. IR (neat): 3355, 3066, 2927, 1600, 1457, 1311, 1122, 755 cm^–1^. HRMS (ES+) *m*/*z*: [M + Na]^+^ calcd for C_18_H_14_F_3_NONa, 340.0925; found, 340.0950. HPLC: *er* = 56.5:43.5. The *er* was determined by chiral HPLC using a Lux 5 μm cellulose-1 (250 × 4.6 mm) column [hexane/isopropanol (80:20), 0.7 mL/min, λ = 254 nm, *t*_major_ = 7.94 min, *t*_minor_ = 8.53 min].

##### (*S*)-5-Phenyl-1-(2-(trifluoromethyl)phenyl)-1*H*-pyrrole-2-carbaldehyde (**3q**)^16^

Isolated yield: 45% (45 mg). Physical state: pale yellow liquid, *R*_f_ = 0.36 (20% EtOAc/hexane). ^1^H NMR (400 MHz, CDCl_3_): δ 9.42 (s, 1H), 7.69 (dd, *J* = 7.6, 1.2 Hz, 1H), 7.63–7.44 (m, 2H), 7.37 (d, *J* = 7.2 Hz, 1H), 7.23–7.05 (m, 6H), 6.55 (d, *J* = 4.0 Hz, 1H). ^13^C{^1^H} NMR (101 MHz, CDCl_3_): δ 178.5, 143.7, 136.4, 135.1, 132.5, 131.5, 130.8, 129.4, 128.6 (2C), 128.5, 128.3 (2C), 128.2, 127.4 (q, *J* = 5.1 Hz), 123.0, 122.9 (q, *J* = 272.7 Hz), 111.6. ^19^F NMR (376 MHz, CDCl_3_): −60.9. IR (neat): 3320, 3066, 2807, 1662, 1457, 1311, 1122, 759 cm^–1^. HRMS (ES+) *m*/*z*: [M + H]^+^ calcd for C_18_H_13_F_3_NO, 316.0949; found, 316.0971. HPLC: *er* = 45.5:54.5 (10 mg of aldehyde was reduced to the corresponding alcohol with NaBH_4_ and determined the *er*). The *er* was determined by chiral HPLC using a Lux 5 μm cellulose-1 (250 × 4.6 mm) column [hexane/isopropanol (80:20), 0.7 mL/min, λ = 254 nm, *t*_minor_ = 7.95 min, *t*_major_ = 8.53 min].

##### Procedure for 1 mmol Scale OKR of Racemic *N*-Aryl Pyrrole Alcohol Using Ambient Air (**2e**)^16^

In a 25 mL single-neck round-bottom flask, racemic **2e** (429 mg, 1 mmol, 1 equiv) was dissolved in 10 mL (0.1 M) of analytical grade acetonitrile. Copper(I) bromide (2.87 mg, 0.02 mmol, 0.02 equiv), bipyridine (3.12 mg, 0.02 mmol, 0.02 equiv), hydroxylamine precatalyst (1*S*,3*S*)-**1** (15.32 mg, 0.02 mmol, 0.02 equiv), and *N*-methyl imidazole [3.28 mg, 0.04 mmol, 0.04 equiv (0.4 mL of 0.1 M acetonitrile solution)] were added to the reaction flask. The resulting brown colored reaction mixture was stirred at room temperature (23–25 °C) open to air. The reaction progress was monitored by ^1^H NMR analysis of the crude reaction mixture. After 27 h of oxidation, at 55% conversion, the reaction was stopped. The acetonitrile was removed from the reaction mixture under reduced pressure at room temperature on a Rotavapor. The crude reaction mixture was subjected to flash column chromatography using hexane and ethyl acetate as the eluent to obtain recovered alcohol **2e** and the product aldehyde **3e**.

##### Recovered Alcohol (*R*)-**2e**

Isolated yield: 41% (176 mg). Physical state: light brown colored solid, *R*_f_ = 0.26 (20% EtOAc/hexane). HPLC: *er* = 8.5:91.5. The *er* was determined by chiral HPLC analysis using a Lux 5 μm cellulose-1 (250 × 4.6 mm) column [hexane/isopropanol (80:20), 0.7 mL/min, λ = 280 nm, *t*_minor_ = 6.74 min, *t*_major_ = 8.46 min].

##### Product Aldehyde (*S*)-**3e**

Isolated yield: 52% (225 mg). Physical state: pale yellow solid, mp: 98–102 °C, *R*_f_ = 0.49 (20% EtOAc/hexane). HPLC: *er* = 78:22 (10 mg of aldehyde was reduced to the corresponding alcohol with NaBH_4_ and determined the *er*). The *er* was determined by chiral HPLC analysis using a Lux 5 μm cellulose-1 (250 × 4.6 mm) column [hexane/isopropanol (80:20), 0.7 mL/min, λ = 280 nm, *t*_major_ = 6.65 min, *t*_minor_ = 8.13 min].

##### Procedure for 1 mmol Scale OKR of Racemic *N*-Aryl Pyrrole Alcohol Using Pure Oxygen (**2e**)^16^

In a 100 mL single-neck round-bottom flask, racemic **2e** (429 mg, 1 mmol, 1 equiv) was dissolved in 10 mL (0.1 M) of analytical grade acetonitrile. Copper(I) bromide (2.87 mg, 0.02 mmol, 0.02 equiv), bipyridine (3.12 mg, 0.02 mmol, 0.02 equiv), hydroxylamine precatalyst (1*S*,3*S*)-**1** (15.32 mg, 0.02 mmol, 0.02 equiv), and *N*-methyl imidazole [3.28 mg, 0.04 mmol, 0.04 equiv (0.4 mL of 0.1 M acetonitrile solution)] were added to the reaction flask. The reaction flask was attached to oxygen balloon and stirred at room temperature (23–25 °C) under an oxygen atmosphere. The reaction progress was monitored by ^1^H NMR analysis of the crude reaction mixture. After 7 h of oxidation, at 46% conversion, the reaction was stopped. The acetonitrile was removed from the reaction mixture under reduced pressure at room temperature on a Rotavapor. The crude reaction mixture was subjected to flash column chromatography using hexane and ethyl acetate as the eluent to obtain recovered alcohol **2e** and the product aldehyde **3e**.

##### Recovered Alcohol (*R*)-**2e**

Isolated yield: 51% (221 mg). Physical state: light brown colored solid, *R*_f_ = 0.26 (20% EtOAc/hexane). HPLC: *er* = 18:82. The *er* was determined by chiral HPLC analysis using a Lux 5 μm cellulose-1 (250 × 4.6 mm) column [hexane/isopropanol (80:20), 0.7 mL/min, λ = 280 nm, *t*_minor_ = 6.74 min, *t*_major_ = 8.46 min].

##### Product Aldehyde (*S*)-**3e**

Isolated yield: 44% (188 mg). Physical state: pale yellow solid, mp: 98–102 °C, *R*_f_ = 0.49 (20% EtOAc/hexane). HPLC: *er* = 83:17 (10 mg of aldehyde was reduced to the corresponding alcohol with NaBH_4_ and determined the *er*). The *er* was determined by chiral HPLC analysis using a Lux 5 μm cellulose-1 (250 × 4.6 mm) column [hexane/isopropanol (80:20), 0.7 mL/min, λ = 280 nm, *t*_major_ = 6.65 min, *t*_minor_ = 8.13 min].

#### General Procedure for the OKR of Racemic Biaryl Alcohols (**4a–r**)

In a 20 mL glass vial, 100 mg of racemic **4** (1 equiv) was dissolved in analytical grade acetonitrile (0.05–0.1 M). CuBr (2 mol %), bipyridine (2 mol %), chiral hydroxylamine (1*S*,3*S*)-**1** (2 mol %), and *N*-methylimidazole (4 mol %, 0.1 M acetonitrile solution) were added to the reaction mixture. The resulting red/brown colored reaction mixture was stirred at RT (23–25 °C) open to air for specified time. The reaction progress was monitored by ^1^H NMR analysis. After the specified conversion, the solvent from the reaction mixture was removed under the reduced pressure. The crude reaction mixture was subjected to flash column chromatography using hexane and ethyl acetate as the eluent to afford aldehyde **5** and recovered alcohol **4**.

##### (*R*)-(1-(2-(Trifluoromethyl)phenyl)naphthalen-2-yl)methanol (**4a**)

According to the general procedure for kinetic resolution, substrate **4a** (100 mg) with catalyst (1*S*,3*S*)-**1** at 23–25 °C afforded aldehyde **5a** and recovered **4a** in 2.5 h at 64% conversion. Isolated yield: 28% (28 mg). Physical state: colorless solid, mp: 77–79 °C, *R*_f_ = 0.16 (20% EtOAc/hexane). ^1^H NMR (400 MHz, CDCl_3_): δ 7.95 (d, *J* = 8.4 Hz, 1H), 7.87 (d, *J* = 8 Hz, 2H), 7.74 (d, *J* = 8.4 Hz, 1H), 7.65–7.58 (m, 2H), 7.46 (ddd, *J* = 6.8 Hz, 1.2 Hz, 1H), 7.35 (ddd, *J* = 6.8 Hz, 1.2 Hz, 1H), 7.30 (d, *J* = 7.2 Hz, 1H), 7.12 (d, *J* = 8.4 Hz, 1H), 4.48 (AB q, *J* = 12.4 Hz, 2H). ^13^C{^1^H} NMR (101 MHz, CDCl_3_): δ 137.2, 136.3, 132.9, 132.7, 132.6, 131.9, 129.8 (q, *J* = 118 Hz, CF_3_), 128.9, 128.3, 127.9, 126.6, 126.5 (q, *J* = 21.2 Hz), 126.3, 125.9, 125.5, 122.7, 63.4. ^19^F NMR (376 MHz, CDCl_3_): −60.1. IR (neat): 3224, 1495, 1313, 1167, 1117, 770 cm^–1^. HRMS (ESI-) *m*/*z*: [M – H]^−^ calcd for C_18_H_12_F3O, 301.0840; found, 301.0819. [α]_D_^25^ = +49.5 (*c* = 0.15, CHCl_3_). HPLC: *er* = 5.5:94.5. The *er* was determined by chiral HPLC using a Lux 5 μm cellulose-1 (250 × 4.6 mm) column [hexane/isopropanol (90:10), 1.0 mL/min, λ = 280 nm, *t*_minor_ = 8.29 min *t*_major_ = 11.64 min].

##### (*S*)-1-(2-(Trifluoromethyl)phenyl)-2-naphthaldehyde (**5a**)

Isolated yield: 54% (54 mg). Physical state: colorless solid, mp: 76–80 °C, *R*_f_ = 0.32 (10% EtOAc/hexane). ^1^H NMR: (400 MHz, CDCl_3_): δ 9.77 (s, 1H), 8.08 (d, *J* = 8.8 Hz, 1H), 7.99 (d, *J* = 8.8 Hz, 1H), 7.94–7.90 (m, 2H), 7.72–7.64 (m, 2H), 7.61 (ddd, *J* = 6.8 Hz, 1.2 Hz, 1H), 7.46–7.39 (m, 2H), 7.32 (d, *J* = 8.8 Hz, 1H). ^13^C{^1^H} NMR (101 MHz, CDCl_3_): δ 191.6, 142.7, 135.9, 134.6, 132.9, 132.8, 131.7, 131.5, 130.5 (q, *J* = 119.6 Hz, CF_3_), 129.3, 129.0, 128.9, 128.3, 127.6, 127.1, 126.4 (q, *J* = 20 Hz), 125.2, 122.5, 122.1. ^19^F NMR (376 MHz, CDCl_3_): −59.4. IR (neat): 3063, 2847, 2738, 1691, 1313, 1116, 820, 748 cm^–1^. HRMS (ESI-) *m*/*z*: [M]^+^ calcd for C_18_H_11_F_3_O, 300.0762; found, 300.0761. [α]_D_^25^ = +31.8 (*c* = 0.30, CHCl_3_). HPLC: *er* = 60:40 (10 mg of aldehyde was reduced to the corresponding alcohol with NaBH_4_ and determined the *er*). The *er* was determined by chiral HPLC using a Lux 5 μm cellulose-1 (250 × 4.6 mm) column [hexane/isopropanol (90:10), 1.0 mL/min, λ = 280 nm, *t*_major_ = 8.24 min, *t*_minor_ = 11.54 min].

##### (*R*)-1,1′-Binaphthalen]-2-ylmethanol (**4b**)

According to the general procedure for kinetic resolution, substrate **4b** (100 mg) with catalyst (1*S*,3*S*)-**1** at 23–25 °C afforded aldehyde **5b** and recovered **4b** in 3 h at 56% conversion. Isolated yield: 34% (34 mg). Physical state: colorless solid, mp: 115–120 °C, *R*_f_ = 0.20 (20% EtOAc/hexane). ^1^H NMR (400 MHz, CDCl_3_): δ 8.02–7.92 (m, 4H), 7.80 (d, *J* = 8.8 Hz, 1H), 7.61 (t, *J* = 6.8 Hz, 1H), 7.50–7.40 (m, 3 H), 7.29–7.17 (m, 4H), 4.43 (AB q, *J* = 12.4 Hz, 2H). ^13^C {^1^H} NMR (101 MHz, CDCl_3_): δ 136.9, 135.9, 135.8, 133.8, 133.3, 133.1, 132.9, 128.52, 128.51, 128.3, 128.1, 128.0, 126.8, 126.6, 126.3, 126.2, 125.98, 125.94, 125.92, 125.6, 63.6. IR (neat): 3282, 3058, 2921, 1504, 1248, 1036, 782 cm^–1^. HRMS (ES+) *m*/*z*: [M + Na]^+^ calcd for C_21_H_16_ONa, 307.1099; found, 307.1092. [α]_D_^25^ = +49.8 (*c* = 0.30, CHCl_3_) [reported literature: [α]_D_^20^ = +46.3 (*c* = 1.0, CHCl_3_), 94% *ee* for *R*-isomer].^18^

HPLC: *er* = 16.5:83.5. The *er* was determined by chiral HPLC using a Lux 5 μm cellulose-1 (250 × 4.6 mm) column [hexane/isopropanol (90:10), 1.0 mL/min, λ = 254 nm, *t*_minor_ = 9.76 min *t*_major_ = 15.33 min].

##### (*S*)-[1,1′-Binaphthalene]-2-carbaldehyde (**5b**)

Isolated yield: 47% (47 mg). Physical state: colorless solid, mp: 119–122 °C, *R*_f_ = 0.50 (10% EtOAc/hexane). ^1^H NMR (400 MHz, CDCl_3_): δ 9.69 (d, *J* = 1.2 Hz, 1H), 8.17 (d, *J* = 8.4 Hz, 1H), 8.05–8.02 (m, 2H), 8.00–7.96 (m, 2H), 7.65–7.58 (m, 2H), 7.52–7.48 (m, 2H), 7.39–7.29 (m, 3H), 7.23–7.20 (m, 1H). ^13^C NMR (101 MHz, CDCl_3_): δ 192.6, 145.0, 136.3, 133.6, 133.5, 133.2, 133.1, 132.3, 129.3, 129.1, 129.0, 128.8, 128.5, 128.4, 127.9, 127.1, 126.9, 126.5, 126.4, 125.2, 122.2. IR (neat): 3333, 3060, 2865, 1676, 1593, 1504, 1240, 805 cm^–1^. HRMS (ES+) *m*/*z*: [M + H]^+^ calcd for C_21_H_15_O, 283.1123; found, 283.1122. [α]_D_^25^ = −7.6 (*c* = 0.45, CHCl_3_) [reported literature: [α]_D_ = −104.0 (*c* = 1.0, CHCl_3_), 98% *ee* for *S*-isomer].^19^

##### (*R*)-(3,4,5-Trimethoxy-2-(naphthalen-1-yl)phenyl)methanol (**4c**)

According to the general procedure for kinetic resolution, substrate **4c** (100 mg, 0.308 mmol) with catalyst (1*S*,3*S*)-**1** (4.73 mg, 0.0061 mmol) in acetonitrile (4.1 mL, 0.075 M) at 23–25 °C afforded aldehyde **5c** and recovered **4c** in 3.5 h at 60% conversion.

Isolated yield: 35% (35 mg). Physical state: colorless solid, mp: 151–154 °C, *R*_f_ = 0.59 (50% EtOAc/hexane). ^1^H NMR (400 MHz, CDCl_3_): δ 7.89 (t, *J* = 7.2 Hz, 2H), 7.53 (dd, *J* = 6.8 Hz, 1.6 Hz, 1H), 7.45 (m, 2H), 7.37 (m, 3H), 7.00 (s, 1H), 4.20 (AB q, *J* = 12.8 Hz, 2H), 3.97 (s, 3H), 3.93 (s, 3H), 3.50 (s, 3H). ^13^C{^1^H} NMR (101 MHz, CDCl_3_): δ 153.4, 151.9, 141.7, 135.5, 134.0, 133.8, 132.9, 128.5, 128.1, 127.7, 126.4, 126.0, 125.6, 125.5, 106.9, 63.1, 61.3, 61.1, 56.2. IR (neat): 3486, 2931, 1592, 1457, 1392, 1326, 1103, 779 cm^–1^. HRMS (ES+) *m*/*z*: [M + Na]^+^ calcd for C_20_H_20_O_4_Na, 347.1259; found, 347.1248. [α]_D_^25^ = +70.1 (*c* = 0.35, CHCl_3_). HPLC: *er* = 19:81. The *er* was determined by chiral HPLC analysis using a Chiralpak AD-H (150 × 4.6 mm) column [hexane/isopropanol (90:10), 0.8 mL/min, λ = 254 nm, *t*_minor_ = 6.17 min, *t*_major_ = 8.15 min].

##### (*S*)-3,4,5-Trimethoxy-2-(naphthalen-1-yl)benzaldehyde (**5c**)

Isolated yield: 57% (57 mg). Physical state: brown colored solid, mp: 110–113 °C *R*_f_ = 0.22 (20% EtOAc/hexane). ^1^H NMR (400 MHz, CDCl_3_): δ 9.36 (s, 1H), 7.93 (t, *J* = 8.0Hz, 2H), 7.55 (dd, *J* = 6.8 Hz, 1.2 Hz, 1H), 7.50 (ddd, *J* = 6.0 Hz, 2.0 Hz, 1H), 7.45 (s, 1H), 7.42 (m, 3H), 4.04 (s, 3H), 4.01 (s, 3H), 3.50 (s, 3H). ^13^C{^1^H} NMR (101 MHz, CDCl_3_): δ 191.1, 153.6, 151.9, 147.8, 133.5, 133.5, 132.6, 131.1, 130.5, 128.9, 128.7, 128.5, 126.7, 126.2, 125.9, 125.1, 105.2, 61.4, 61.2, 56.3. IR (neat): 2938, 1681, 1577, 1469, 1326, 1106, 779 cm^–1^. HRMS (ES+) *m*/*z*: [M + Na]^+^ calcd for C_20_H_18_O_4_Na, 345.1103; found, 345.1091. [α]_D_^25^ = +9.8 (*c* = 0.50, CHCl_3_). HPLC: *er* = 65.5:34.5 (10 mg of aldehyde was reduced to the corresponding alcohol with NaBH_4_ and determined the *er*). The *er* of the product was determined by chiral HPLC using a Chiralpak AD-H (150 × 4.6 mm) column [hexane/isopropanol (90:10), 0.8 mL/min, λ = 254 nm, *t*_major_ = 8.90 min, *t*_minor_ = 8.93 min].

##### (*R*)-(3-(Benzyloxy)-4-methoxy-2-(naphthalen-1-yl)phenyl)methanol (**4d**)

According to the general procedure for kinetic resolution, substrate **4d** (100 mg, 0.269 mmol) with catalyst (1*S*,3*S*)-**1** (4.14 mg, 0.0054 mmol) in acetonitrile (3.6 mL, 0.075 M) at 23–25 °C afforded aldehyde **5d** and recovered **4d** in 3 h at 64% conversion. Isolated yield: 28% (28 mg). Physical state: colorless solid, mp: 76–80 °C, *R*_f_ = 0.48 (50% EtOAc/hexane). ^1^H NMR (400 MHz, CDCl_3_): δ 7.90 (m, 2H), 7.52–7.44 (m, 3H), 7.37–7.31 (m, 3H), 7.10–7.00 (m, 4H), 6.63 (d, *J* = 6.8 Hz, 2H), 4.67 (q, *J* = 10.8 Hz, 2H), 4.27–4.18 (m, 2H), 3.96 (s, 3H). ^13^C{^1^H} NMR (101 MHz, CDCl_3_): δ 152.8, 146.1, 137.4, 134.2, 134.0, 133.7, 133.0, 128.6, 128.3, 128.0, 127.9, 127.9, 127.5, 127.5, 126.4, 125.9, 125.8, 125.4, 124.1, 112.0, 74.9, 63.2, 56.1. IR (neat): 3309, 2938, 1442, 1257, 1002, 782 cm^–1^. HRMS (ES+) *m*/*z*: [M + Na]^+^ calcd for C_25_H_22_O_3_Na, 393.1467; found, 393.1468; [α]_D_^25^ = +63.6 (*c* = 0.28, CHCl_3_). HPLC: *er* = 24:76. The *er* of the product was determined by chiral HPLC analysis using a Chiralpak AD-H (150 × 4.6 mm) column [hexane/isopropanol (90:10), 1.0 mL/min, λ = 254 nm, *t*_minor_ = 8.88 min, *t*_major_ = 22.64 min].

##### (*S*)-3-(Benzyloxy)-4-methoxy-2-(naphthalen-1-yl)benzaldehyde (**5d**)

Isolated yield: 62% (62 mg). Physical state: brown colored solid, mp: 115–118 °C, *R*_f_ = 0.31 (20% EtOAc/hexane). ^1^H NMR (400 MHz, CDCl_3_): δ 9.38 (d, *J* = 0.8 Hz, 1H), 7.97–7.91 (m, 3H), 7.53–7.35 (m, 5H), 7.17 (dd, *J* = 8.8 Hz, 0.8 Hz, 1H), 7.13–7.02 (m, 3H), 6.65 (d, *J* = 7.2 Hz, 2H), 4.66 (q, *J* = 10.8 Hz, 2H), 4.03 (s, 3H). ^13^C{^1^H} NMR (101 MHz, CDCl_3_): δ 191.0, 158.1, 145.7, 139.2, 136.9, 133.4, 133.2, 131.2, 129.1, 128.8, 128.6, 128.3, 127.9, 127.9, 126.7, 126.2, 126.1, 125.1, 124.7, 111.8, 75.0, 56.2. IR (neat): 3050, 2846, 1677, 1573, 1249, 786 cm^–1^. HRMS (ES+) *m*/*z*: [M + Na]^+^ calcd for C_25_H_20_O_3_Na, 391.1310; found, 391.1312. [α]_D_^20^ = −13.5 (*c* = 0.52, CHCl_3_). HPLC: *er* = 66:34 (10 mg of aldehyde was reduced to the corresponding alcohol with NaBH_4_ and determined the *er*). The *er* of the product was determined by chiral HPLC using a Chiralpak AD-H (150 × 4.6 mm) column [hexane/isopropanol (90:10), 0.8 mL/min, λ = 254 nm, *t*_major_ = 9.67 min, *t*_minor_ = 24.88 min].

##### (*R*)-(3,4-Dimethoxy-2-(naphthalen-1-yl)phenyl)methanol (**4e**)

According to the general procedure for kinetic resolution, substrate **4e** (100 mg, 0.34 mmol) with catalyst (1*S*,3*S*)-**1** (4.14 mg, 0.0068 mmol) in acetonitrile (4.5 mL, 0.075 M) at 23–25 °C afforded aldehyde **5e** and recovered **4e** in 3 h at 60% conversion. Isolated yield: 36% (36 mg). Physical state: colorless solid, mp: 124–127 °C, *R*_f_ = 0.54 (50% EtOAc/hexane). ^1^H NMR (400 MHz, CDCl_3_): δ 7.90 (d, *J* = 8.4 Hz, 2H), 7.54 (dd, *J* = 6.8 Hz, 1.2 Hz, 1H), 7.49–7.42 (m, 2H), 7.38–7.34 (m, 2H), 7.30 (d, *J* = 8.4 Hz, 1H), 7.05 (d, *J* = 8.4 Hz, 1H) 4.16 (m, 2H), 3.94 (s, 3H), 3.45 (s, 3H). ^13^C{^1^H} NMR (101 MHz, CDCl_3_): δ 152.5, 147.2, 134.1, 133.8, 133.7, 133.0, 132.5, 128.4, 128.1, 127.2, 126.4, 126.0, 125.7, 125.4, 123.9, 112.0, 63.1, 60.9, 55.9. IR (neat): 3301, 2935, 1585, 1461, 1257, 1002, 782 cm^–1^. HRMS (ES+) *m*/*z*: [M + Na]^+^ calcd for C_19_H_18_O_3_Na, 317.1154; found, 317.1150. [α]_D_^25^ = +33.8 (*c* = 0.36, CHCl_3_). HPLC: *er* = 24:76. The *er* of the product was determined by chiral HPLC analysis using a Chiral Lux 5u cellulose-1 (250 × 4.6 mm) column [hexane/isopropanol (90:10), 1.0 mL/min, λ = 280 nm, *t*_minor_ = 12.29 min, *t*_major_ = 17.78 min].

##### (*S*)-3,4-Dimethoxy-2-(naphthalen-1-yl)benzaldehyde (**5e**)

Isolated yield: 55% (55 mg). Physical state: brown colored solid, mp: 144–148 °C, *R*_f_ = 0.26 (20% EtOAc/hexane). ^1^H NMR (400 MHz, CDCl_3_): δ 9.31 (d, *J* = 0.8 Hz, 1H), 7.95–7.91 (m, 3H), 7.56 (dd, *J* = 8 Hz, 0.4 Hz, 1H), 7.49 (ddd, *J* = 6 Hz, 1.6 Hz, 1H), 7.44–7.37 (m, 3H), 7.14 (dd, *J* = 8.8 Hz, 0.8 Hz, 1H), 4.02 (s, 3H), 3.46 (s, 3H). ^13^C{^1^H} NMR (101 MHz, CDCl_3_): δ 190.9, 157.9, 147.0, 138.7, 133.4, 133.1, 131.3, 129.1, 128.7, 128.4, 128.4, 126.7, 126.2, 126.0, 125.1, 124.5, 111.8, 61.1, 56.2. IR (neat): 3725, 2931, 1681, 1573, 1257, 782 cm^–1^. HRMS (ES+) *m*/*z*: [M + H]^+^ calcd for C_19_H_17_O_3_: 293.1178; found, 293.1174. [α]_D_^25^ = −23.1 (*c* = 0.45, CHCl_3_). HPLC: *er* = 62:38 (10 mg of aldehyde was reduced to the corresponding alcohol with NaBH_4_ and determined the *er*). The *er* of the product was determined by chiral HPLC using a Chiral Lux 5u cellulose-1 (250 × 4.6 mm) column [hexane/isopropanol (90:10), 0.8 mL/min, λ = 280 nm, *t*_major_ = 12.36 min, *t*_minor_ = 18.18 min].

##### (*R*)-(2-(2-Methylnaphthalen-1-yl)phenyl)methanol (**4f**)

According to the general procedure for kinetic resolution, substrate **4f** (100 mg, 0.403 mmol) with catalyst (1*S*,3*S*)-**1** (6.17 mg, 0.00805 mmol) in acetonitrile (5.4 mL, 0.075 M) at 23–25 °C afforded aldehyde **5f** and recovered **4f** in 4 h at 43% conversion.

Isolated yield: 44% (44 mg). Physical state: colorless sticky material, *R*_f_ = 0.30 (20% EtOAc/hexane). ^1^H NMR (400 MHz, CDCl_3_): δ 7.86 (d, *J* = 8.4 Hz, 1H), 7.81 (dd, *J* = 8.4 Hz, 1H), 7.67 (d, *J* = 8.4 Hz, 1H), 7.50 (td, *J* = 7.2 Hz, 1.6 Hz, 1H), 7.46–7.39 (m, 3H), 7.34–7.30 (m, 1H), 7.23 (d, *J* = 8.8 Hz, 1H), 7.17 (dd, *J* = 7.2 Hz, 1.2 Hz, 1H), 4.25 (AB q, *J* = 12.8 Hz, 2H), 2.18 (s, 3H). ^13^C{^1^H}NMR (101 MHz, CDCl_3_): δ 139.5, 138.1, 136.0, 133.6, 132.8, 132.1, 130.4, 128.7, 128.1, 128.1, 127.9, 127.8, 127.7, 126.4, 125.6, 125.1, 63.3, 20.7. IR (neat): 3567, 3359, 3050, 1443, 1029, 752 cm^–1^. HRMS (ES+) *m*/*z*: [M + Na]^+^ calcd for C_18_H_16_ONa, 271.1099; found, 271.1100. [α]_D_^20^ = +3.4 (*c* = 0.43, CHCl_3_). HPLC: *er* = 30:70. The *er* of the product was determined by chiral HPLC analysis using a Chiral Lux 5u cellulose-1 (250 × 4.6 mm) column [hexane/isopropanol (90:10), 0.5 mL/min, λ = 280 nm, *t*_minor_ = 13.59 min, *t*_major_ = 14.4 min].

##### (*S*)-2-(2-Methylnaphthalen-1-yl)benzaldehyde (**5f**)

Isolated yield: 40% (40 mg). Physical state: brown colored sticky material, *R*_f_ = 0.47 (10% EtOAc/hexane). ^1^H NMR (400 MHz, CDCl_3_): δ 9.50 (d, *J* = 0.8 Hz, 1H), 8.13 (dd, *J* = 7.6 Hz, 1.2 Hz, 1H), 7.85 (t, *J* = 7.2 Hz, 2H), 7.72 (td, *J* = 7.6 Hz, 1.6 Hz, 1H), 7.58 (m, 1H), 7.44–7.39 (m, 2H), 7.35–7.29 (m, 2H), 7.19 (d, *J* = 8.4 Hz, 1H), 2.18 (s, 3H). ^13^C{^1^H} NMR (101 MHz, CDCl_3_): δ 192.2, 143.9, 134.8, 134.4, 134.4, 133.5, 133.4, 131.9, 131.7, 128.5, 128.4, 128.3, 128.1, 127.4, 126.7, 125.8, 125.3, 20.9. IR (neat): 3054, 2842, 1693, 1592, 759 cm^–1^. HRMS (ES+) *m*/*z*: [M + H]^+^ calcd for C_18_H_15_O, 247.1123; found, 247.1131. [α]_D_^25^ = +8.4 (*c* = 0.30, CHCl_3_) [reported literature: [α]_D_^20^ = −7.6 (*c* = 1.50, CHCl_3_), 90% *ee* for *R*-isomer].^20^ HPLC: *er* = 63.5:36.5 (10 mg of aldehyde was reduced to the corresponding alcohol with NaBH_4_ and determined the *er*). The *er* of the product was determined by chiral HPLC using a Chiral Lux 5u cellulose-1 (250 × 4.6 mm) column [hexane/isopropanol (90:10), 0.5 mL/min, λ = 280 nm, *t*_major_ = 13.56 min, *t*_minor_ = 14.37 min].

##### (*R*)-(3-Methyl-2-(4-methylnaphthalen-1-yl)phenyl)methanol (**4g**)

According to the general procedure for kinetic resolution, substrate **4g** (100 mg, 0.381 mmol) with catalyst (1*S*,3*S*)-**1** (5.84 mg, 0.00805 mmol) in acetonitrile (5.1 mL, 0.075 M) at 23–25 °C afforded aldehyde **5g** and recovered **4g** in 3.5 h at 66% conversion.

Isolated yield: 27% (27 mg). Physical state: colorless solid, mp: 96–100 °C, *R*_f_ = 0.33 (20% EtOAc/hexane). ^1^H NMR (400 MHz, CDCl_3_): δ 8.08 (dd, *J* = 8.4 Hz, 0.8 Hz, 1H), 7.53 (ddd, *J* = 6.4 Hz, 1.6 Hz, 1H), 7.46 (d, *J* = 7.6 Hz, 1H), 7.39 (t, *J* = 7.2 Hz, 3H), 7.35–7.28 (m, 2H), 7.20 (d, *J* = 7.2 Hz, 1H) 4.22 (AB q, *J* = 12.8 Hz, 2H), 2.77 (s, 3H), 1.92 (s, 3H). ^13^C{^1^H} NMR (101 MHz, CDCl_3_): δ 139.8, 138.8, 137.6, 135.4, 134.2, 133.0, 132.1, 129.3, 127.9, 126.5, 126.2, 125.9, 125.9, 125.3, 124.7, 63.8, 20.2, 19.6. IR (neat): 3343, 2927, 1450, 1045, 755 cm^–1^. HRMS (ES+) *m*/*z*: [M + Na]^+^ calcd for C_19_H_18_ONa, 285.1255; found, 285.1260. [α]_D_^25^ = +22.5 (*c* = 0.27, CHCl_3_). HPLC: *er* = 18.5:81.5. The *er* of the product was determined by chiral HPLC analysis using a Chiral Lux 5u cellulose-1 (250 × 4.6 mm) column [hexane/isopropanol (90:10), 0.5 mL/min, λ = 280 nm, *t*_minor_ = 14.28 min, *t*_major_ = 16.03 min].

##### (*S*)-3-Methyl-2-(4-methylnaphthalen-1-yl)benzaldehyde (**5g**)

Isolated yield: 59% (59 mg). Physical state: colorless solid, mp: 114–118 °C, *R*_f_ = 0.47 (10% EtOAc/hexane). ^1^H NMR (400 MHz, CDCl_3_): δ 9.48 (d, *J* = 0.8 Hz, 1H), 8.09 (dd, *J* = 8.8 Hz, 1.2 Hz, 1H), 7.94 (d, *J* = 7.6 Hz, 1H), 7.57–7.51 (m, 2H), 7.47 (t, *J* = 8.0 Hz, 1H), 7.40–7.36 (m, 2H), 7.29 (d, *J* = 8.4 Hz, 1H), 7.22 (d, *J* = 7.2 Hz, 1H), 2.77 (s, 3H), 1.97 (s, 3H). ^13^C{^1^H} NMR (101 MHz, CDCl_3_): δ 192.8, 144.1, 138.6, 135.5, 135.4, 134.9, 132.7, 132.7, 132.6128.0, 127.7, 126.46, 126.4, 126.3, 126.1, 126.1, 124.7, 124.6, 19.7, 19.6. IR (neat): 2946, 2838, 1681, 1238, 771 cm^–1^. HRMS (ES+) *m*/*z*: [M + H]^+^ calcd for C_19_H_17_O, 261.1279; found, 261.1286. [α]_D_^20^ = −18.3 (*c* = 0.50, CHCl_3_) [reported literature: [α]_D_^20^ = +59.5 (*c* = 0.50, CHCl_3_), 68% *ee* for *R*-isomer].^21^ HPLC: *er* = 61:39 (10 mg of aldehyde was reduced to the corresponding alcohol with NaBH_4_ and determined the *er*). The *er* of the product was determined by chiral HPLC using a Chiral Lux 5u cellulose-1 (250 × 4.6 mm) column [hexane/isopropanol (90:10), 0.5 mL/min, λ = 280 nm, *t*_major_ = 14.93 min, *t*_minor_ = 16.8 min].

##### (*R*)-(6-Methyl-2′-(trifluoromethyl)-[1,1′-biphenyl]-2-yl)methanol (**4h**)

According to the general procedure for kinetic resolution, substrate **4h** (100 mg, 0.375 mmol) with catalyst (1*S*,3*S*)-**1** (5.76 mg, 0.00751 mmol) in acetonitrile (3.8 mL, 0.1 M) at 23–25 °C afforded aldehyde **5h** and recovered **4h** in 2.5 h at 53% conversion.

Isolated yield: 38% (38 mg). Physical state: off-white solid, mp: 91–93 °C, *R*_f_ = 0.34 (20% EtOAc/hexane). ^1^H NMR (400 MHz, CDCl_3_): δ 7.78 (d, *J* = 7.8 Hz, 1H), 7.60 (t, *J* = 7.4 Hz, 1H), 7.50 (t, *J* = 7.6 Hz, 1H), 7.40 (d, *J* = 7.5 Hz, 1H), 7.33 (t, *J* = 7.6 Hz, 1H), 7.20 (d, *J* = 7.5 Hz, 2H), 4.25 (AB q, *J* = 13.2 Hz, 2H), 1.95 (s, 3H). ^13^C{^1^H} NMR (101 MHz, CDCl_3_): δ 139.1, 138.4, 137.2, 136.7, 132.1, 131.6, 129.0, 128.6 (q, *J* = 116 Hz, CF3), 128.4, 127.9, 126.5 (q, *J* = 20 Hz), 125.4, 124.8, 122.7, 63.3, 20.3. ^19^F NMR (376 MHz, CDCl_3_): δ −60.75. IR (neat): 3266, 2931, 1446, 1311, 1168, 1114, 763 cm^–1^. HRMS (ES-) *m*/*z*: [M – H]^+^ calcd for C_15_H_13_F_3_O, 265.0840; found, 265.0833. [α]_D_^25^ = +35.0 (*c* = 0.38, CHCl_3_). HPLC: *er* = 18:82. The *er* of the product was determined by chiral HPLC analysis using a Chiral Lux 5u amylose-2 (250 × 4.6 mm) column [hexane/isopropanol (90:10), 0.2 mL/min, λ = 220 nm, *t*_minor_ = 24.14 min, *t*_major_ = 25.16 min].

##### (*S*)-6-Methyl-2′-(trifluoromethyl)-[1,1′-biphenyl]-2-carbaldehyde (**5h**)

Isolated yield: 50% (50 mg). Physical state: brown oil, *R*_f_ = 0.40 (10% EtOAc/hexane). ^1^H NMR (400 MHz, CDCl_3_): δ 9.58 (s, 1H), 7.93–7.75 (m, 2H), 7.64 (td, *J* = 7.5, 0.7 Hz, 1H), 7.60–7.54 (m, 1H), 7.51 (ddd, *J* = 7.6, 1.4, 0.7 Hz, 1H), 7.45 (t, *J* = 7.6 Hz, 1H), 7.29–7.21 (m, 1H), 2.02 (s, 3H). ^13^C{^1^H} NMR (101 MHz, CDCl_3_): δ 191.7, 141.6, 137.8, 136.2, 135.4, 134.5, 132.0, 131.7, 129.3 (q, *J* = 120 Hz, CF3), 128.5, 128.4, 126.4 (q, *J* = 20 Hz), 125.2, 122.5, 19.8. ^19^F NMR (376 MHz, CDCl_3_): δ −60.21. IR (neat): 3355, 2854, 1689, 1589, 1311, 1110, 763 cm^–1^; HRMS (ES-)*m*/*z*: [M – H]^+^ calcd for C_15_H_11_F_3_O, 263.0684; found, 263.0679. [α]_D_^25^ = +36.1(*c* = 0.40, CHCl_3_). HPLC: *er* = 70:30 (10 mg of aldehyde was reduced to the corresponding alcohol with NaBH_4_ and determined the *er*). The *er* of the product was determined by chiral HPLC using a Chiral Lux 5u Amylose-2 (250 × 4.6 mm) column [hexane/isopropanol (90:10), 0.2 mL/min, λ = 220 nm, *t*_major_ = 23.97 min, *t*_minor_ = 24.96 min].

##### (*R*)-(4,5,6-Trimethoxy-2′-(trifluoromethyl)-[1,1′-biphenyl]-2-yl)methanol (**4i**)

According to the general procedure for kinetic resolution, substrate **4i** (100 mg, 0.292 mmol) with catalyst (1*S*,3*S*)-**1** (4.47 mg, 0.00584 mmol) in acetonitrile (2.95 mL, 0.1 M) at 23–25 °C afforded aldehyde **5i** and recovered **4i** in 3 h at 51% conversion.

Isolated yield: 37% (37 mg). Physical state: colorless solid, mp: 102–105 °C, *R*_f_ = 0.1 (20% EtOAc/hexane). ^1^H NMR (400 MHz, CDCl_3_): δ 7.76 (d, *J* = 7.8 Hz, 1H), 7.57 (t, *J* = 7.4 Hz, 1H), 7.49 (t, *J* = 7.6 Hz, 1H), 7.32–7.18 (m, 1H), 6.91 (s, 1H), 4.22 (AB q, *J* = 12.9 Hz, 2H), 3.93 (s, 3H), 3.87 (s, 3H), 3.67 (s, 3H). ^13^C{^1^H} NMR (101 MHz, CDCl_3_): δ 153.8, 151.4, 141.0, 135.3, 134.8, 132.3, 131.6, 129.6 (q, *J* = 116.8 Hz, CF3), 127.9, 126.4 (q, *J* = 20 Hz), 125.5, 124.2, 122.8, 106.1, 62.8, 60.9, 56.1. ^19^F NMR (376 MHz, CDCl_3_): δ −60.27. IR (neat): 3494, 2931, 1457, 1315, 1103, 771 cm^–1^. HRMS (ES+) *m*/*z*: [M + Na]^+^ calcd for C_17_H_17_F_3_O_4_Na, 365.0977; found, 365.0980. [α]_D_^25^ = +10.4 (*c* = 0.37, CHCl_3_). HPLC: *er* = 19:81. The *er* of the product was determined by chiral HPLC analysis using a Chiral Pak AD-H (150 × 4.6 mm) column [hexane/isopropanol (95:5), 0.3 mL/min, λ = 280 nm, *t*_minor_ = 26.86 min, *t*_major_ = 32.82 min].

##### (*S*)-4,5,6-Trimethoxy-2′-(trifluoromethyl)-[1,1′-biphenyl]-2-carbaldehyde (**5i**)

Isolated yield: 50% (50 mg). Physical state: brown colored solid, mp: 79–81 °C, *R*_f_ = 0.15 (10% EtOAc/hexane). ^1^H NMR (400 MHz, CDCl_3_): δ 9.43 (s, 1H), 7.80 (d, *J* = 7.7 Hz, 1H), 7.58 (m, 2H), 7.32 (m, 2H), 3.97 (s, 6H), 3.68 (s, 3H). ^13^C{^1^H} NMR (101 MHz, CDCl_3_): δ 190.1, 153.9, 151.5, 147.2, 132.7, 131.4, 130.2 (q, *J* = 118 Hz, CF3) 128.5, 128.0, 126.3 (q, *J* = 20 Hz) 125.3, 122.6, 104.9, 61.0, 56.2. IR (neat): 2931, 1681, 1461, 1311, 1103, 775 cm^–1^. ^19^F NMR (376 MHz, CDCl_3_): δ −59.50. HRMS (ES+) *m*/*z*: [M + Na]^+^ calcd for C_17_H_15_F_3_O_4_Na, 363.0820; found, 363.0821. [α]_D_^25^ = +6.8 (*c* = 0.40, CHCl_3_). HPLC: *er* = 68:32 (10 mg of aldehyde was reduced to the corresponding alcohol with NaBH_4_ and determined the *er*). The *er* of the product was determined by chiral HPLC using a Chiral Pak AD-H (150 × 4.6 mm) column [hexane/isopropanol (95:5), 0.3 mL/min, λ = 280 nm, *t*_major_ = 26.69 min, *t*_minor_ = 33.13 min].

##### (*R*)-(1-(2,3-Dimethylphenyl)naphthalen-2-yl)methanol (**4j**)

According to the general procedure for kinetic resolution, substrate **4j** (100 mg, 0.381 mmol) with catalyst (1*S*,3*S*)-**1** (5.84 mg, 0.00762 mmol) in acetonitrile (3.85 mL, 0.1 M) at 23–25 °C afforded aldehyde **5j** and recovered **4j** in 3 h at 62% conversion. Isolated yield: 12% (12 mg). Physical state: sticky material, *R*_f_ = 0.34 (20% EtOAc/hexane). ^1^H NMR (400 MHz, CDCl_3_): δ 7.89 (t, *J* = 9.2 Hz, 2H), 7.70 (d, *J* = 8.4 Hz, 1H), 7.45 (m, 1H), 7.37–7.30 (m, 1H), 7.30–7.24 (m, 2H), 7.20 (t, *J* = 7.6 Hz, 2H), 7.02–6.97 (d, *J* = 7.2 Hz, 1H), 4.54–4.43 (m, 2H), 2.38 (s, 3H), 1.83 (s, 3H). ^13^C {^1^H} NMR (101 MHz, CDCl3): δ 138.0, 137.7, 137.4, 135.7, 135.6, 133.1, 132.6, 129.5, 128.1, 128.0, 127.9, 126.5, 126.2, 125.9, 125.8, 125.7, 63.7, 20.7, 16.5. IR (neat): 3270, 2923, 1450, 1014, 744 cm^–1^. HRMS (ES+) *m*/*z*: [M + Na]^+^ calcd for C_19_H_18_ONa, 285.1255; found, 285.1248. [α]_D_^25^ = +155.3 (*c* = 0.12, CHCl_3_) [reported literature: [α]_D_^24^ = −49.6 (*c* = 1.040, CH_2_Cl_2_), 96% *ee* for *S*-isomer].^22^

HPLC: *er* = 27:73. The *er* of the product was determined by chiral HPLC analysis using a Chiral Lux 5u cellulose-1 (250 × 4.6 mm) column [hexane/isopropanol (90:10), 1.0 mL/min, λ = 280 nm, *t*_minor_ = 6.28 min, *t*_major_ = 6.96 min].

##### (*S*)-1-(2,3-Dimethylphenyl)-2-naphthaldehyde (**5j**)

Isolated yield: 60% (60 mg). Physical state: brown colorless solid, mp: 123–127 °C, *R*_f_ = 0.5 (10% EtOAc/hexane). ^1^H NMR (400 MHz, CDCl_3_): δ 9.86 (d, *J* = 0.8 Hz, 1H), 8.11 (d, *J* = 8.6 Hz, 1H), 7.96 (dd, *J* = 8.7, 0.7 Hz, 2H), 7.64 (ddd, *J* = 8.2, 6.7, 1.4 Hz, 1H), 7.56–7.42 (m, 2H), 7.35 (d, *J* = 7.4 Hz, 1H), 7.27 (dd, *J* = 12.5, 4.9 Hz, 1H), 7.12 (d, *J* = 7.4 Hz, 1H), 2.43 (s, 3H), 1.90 (s, 3H). ^13^C{^1^H} NMR (101 MHz, CDCl_3_): 192.9, 147.2, 137.4, 136.3, 136.1, 134.9, 132.5, 131.2, 130.2, 128.9, 128.9, 128.4, 128.2, 127.6, 127.0, 125.4, 122.1, 20.3, 16.9. IR (neat): 2927, 2861, 1681, 1454, 767 cm^–1^. HRMS (ES+) *m*/*z*: [M]^+^ calcd for C_19_H_16_O, 260.1201; found: 260.1190. [α]_D_^25^ = +14.0 (*c* = 0.50, CHCl_3_). HPLC: *er* = 62:31 (10 mg of aldehyde was reduced to the corresponding alcohol with NaBH_4_ and determined the *er*). The *er* of the product was determined by chiral HPLC using a Chiral Lux 5u cellulose-1 (250 × 4.6 mm) column [hexane/isopropanol (90:10), 1.0 mL/min, λ = 280 nm, *t*_major_ = 6.23 min, *t*_minor_ = 6.88 min].

##### (*R*)-(1-(Phenanthren-9-yl)naphthalen-2-yl)methanol (**4k**)

According to the general procedure for kinetic resolution, substrate **4k** (100 mg, 0.299 mmol) with catalyst (1*S*,3*S*)-**1** (4.85 mg, 0.00598 mmol) in acetonitrile-EtOAc (1:1) (5.3 mL, 0.05 M) at 23–25 °C afforded aldehyde **5k** and recovered **4k** in 4 h at 67% conversion. Isolated yield: 27% (27 mg). Physical state: brown colored solid, mp: 83–85 °C, *R*_f_ = 0.31 (20% EtOAc/hexane). ^1^H NMR (400 MHz, CDCl_3_): δ 8.80 (t, *J* = 7.3 Hz, 2H), 8.01 (d, *J* = 8.4 Hz, 1H), 7.93 (d, *J* = 8.2 Hz, 1H), 7.87 (dd, *J* = 7.8, 1.2 Hz, 1H), 7.81 (d, *J* = 8.5 Hz, 1H), 7.77–7.60 (m, 4H), 7.45 (ddd, *J* = 8.1, 6.7, 1.3 Hz, 1H), 7.36 (ddd, *J* = 8.1, 7.0, 1.1 Hz, 1H), 7.33–7.17 (m, 3H), 4.50 (AB q, *J* = 12.8 Hz, 2H). ^13^C{^1^H} NMR (101 MHz, CDCl_3_): δ 137.0, 135.7, 134.6, 133.3, 133.2, 132.1, 131.6, 130.6, 130.5, 128.9, 128.6, 128.1, 127.16, 127.12, 126.9, 126.8, 126.4, 126.0, 125.9, 123.1, 122.8, 63.7. IR (neat): 3567, 3394, 3054, 2923, 1781, 1446, 1149, 728 cm^–1^. HRMS (AP+) *m*/*z*: [M + Na]^+^ calcd for C_25_H_18_ONa, 357.1255; found: 357.1258. [α]_D_^25^ = +82.6 (*c* = 0.27, CHCl_3_). HPLC: *er* = 35:65. The *er* of the product was determined by chiral HPLC analysis using a Chiral Lux 5u cellulose-1 (250 × 4.6 mm) column [hexane/isopropanol (80:20), 1.0 mL/min, λ = 280 nm, *t*_minor_ = 17.04 min, *t*_major_ = 22.98 min].

##### (*S*)-1-(Phenanthren-9-yl)-2-naphthaldehyde (**5k**)

Isolated yield: 65% (65 mg). Physical state: colorless solid, mp: 91–94 °C, *R*_f_ = 0.4 (10% EtOAc/hexane). ^1^H NMR (400 MHz, CDCl_3_): δ 9.83 (d, *J* = 0.8 Hz, 1H), 8.83 (t, *J* = 7.9 Hz, 2H), 8.19 (d, *J* = 8.0 Hz, 1H), 8.05 (d, *J* = 8.7 Hz, 1H), 7.97 (d, *J* = 8.0 Hz, 1H), 7.89 (dt, *J* = 7.9, 3.9 Hz, 1H), 7.79–7.74 (m, 2H), 7.69–7.64 (m, 2H), 7.59 (ddd, *J* = 8.1, 6.8, 1.2 Hz, 1H), 7.50–7.48 (m, 1H), 7.40 (ddd, *J* = 8.1, 7.0, 1.1 Hz, 1H), 7.32–7.26 (m, 2H). ^13^C{^1^H} NMR (101 MHz, CDCl_3_): δ 192.6, 144.9, 136.3, 133.2, 132.6, 132.5, 131.8, 131.1, 130.7, 130.4, 130.4, 129.1, 128.9, 128.9, 128.4, 127.9, 127.6, 127.5, 127.4, 127.4, 127.2, 127.2, 123.2, 122.9, 122.3. IR (neat): 3058, 2865, 1677, 1226, 744 cm^–1^. HRMS (ES+) *m*/*z*: [M + H]^+^ calcd for C_25_H_17_O, 333.1279; found: 333.1266. [α]_D_^25^ = +58.6 (*c* = 0.5, CHCl_3_). HPLC: *er* = 59.5:40.5 (10 mg of aldehyde was reduced to the corresponding alcohol with NaBH_4_ and determined the *er*). The *er* of the product was determined by chiral HPLC using a Chiral Lux 5u cellulose-1 (250 × 4.6 mm) column [hexane/isopropanol (80:20), 1.0 mL/min, λ = 280 nm, *t*_major_ = 16.02 min, *t*_minor_ = 21.26 min].

##### (*R*)-(1-([1,1′-Biphenyl]-2-yl)naphthalen-2-yl)methanol (**4l**)

According to the general procedure for kinetic resolution, substrate **4l** (100 mg, 0.322 mmol) with catalyst (1*S*,3*S*)-**1** (4.94 mg, 0.00644 mmol) in acetonitrile-EtOAc (1:1) (6.4 mL, 0.05 M) at 23–25 °C afforded aldehyde **5l** and recovered **4l** in 5 h at 65% conversion. Isolated yield: 20% (20 mg). Physical state: colorless sticky material, *R*_f_ = 0.38 (20% EtOAc/hexane). ^1^H NMR (400 MHz, CDCl_3_): δ 7.87–7.80 (m, 2H), 7.62–7.53 (m, 3H), 7.50–7.40 (m, 4H), 7.31–7.29 (m, 1H), 7.10–6.90 (m, 5H), 4.27 (AB q, *J* = 12.8 Hz, 2H). ^13^C{^1^H} NMR: (101 MHz, CDCl_3_): δ 141.9, 140.9, 136.6, 136.5, 135.6, 133.5, 132.9, 131.9, 130.3, 128.9, 128.7, 128.4, 128.2, 128.1, 128.0, 127.4, 127.1, 126.9, 126.4, 126.0, 125.7, 125.5, 63.4. IR (neat): 3556, 3386, 3054, 1446, 1037, 744 cm^–1^. HRMS (ES+) *m*/*z*: [M + Na]^+^ calcd for C_23_H_18_ONa, 333.1255; found, 333.1241. [α]_D_^25^ = +222.5 (*c* = 0.2, CHCl_3_). HPLC: *er* = 8:92. The *er* of the product was determined by chiral HPLC analysis using a Chiral Lux 5u cellulose-1 (250 × 4.6 mm) column [hexane/isopropanol (80:20), 1.0 mL/min, λ = 280 nm, *t*_minor_ = 4.78 min, *t*_major_ = 7.62 min].

##### (*S*)-1-([1,1′-Biphenyl]-2-yl)-2-naphthaldehyde (**5l**)

Isolated yield: 60% (60 mg). Physical state: colorless sticky material, *R*_f_ = 0.4 (10% EtOAc/hexane). ^1^H NMR (400 MHz, CDCl_3_): δ 9.86 (d, *J* = 0.8 Hz, 1H), 7.89–7.81 (m, 3H), 7.77–7.74 (m, 1H), 7.64–7.41 (m, 6H), 7.04–6.94 (m, 5H). ^13^C{^1^H} NMR (101 MHz, CDCl_3_): δ 192.4, 145.8, 143.3, 140.5, 136.1, 133.7, 132.9, 132.1, 130.9, 130.2, 129.1, 128.8, 128.7, 128.4, 128.3, 128.0, 127.8, 127.2, 127.1, 127.0, 122.1. IR (neat): 3054, 2846, 1677, 1234, 752 cm^–1^. HRMS (ES-) *m*/*z*: [M – H]^+^ calcd for C_23_H_15_O, 307.1123; found: 307.1133. [α]_D_^25^ = −26.1 (*c* = 0.5, CHCl_3_). HPLC: *er* = 59:41 (10 mg of aldehyde was reduced to the corresponding alcohol with NaBH_4_ and determined the *er*). The *er* of the product was determined by chiral HPLC using a Chiral Lux 5u cellulose-1 (250 × 4.6 mm) column [hexane/isopropanol (80:20), 1.0 mL/min, λ = 280 nm, *t*_major_ = 4.79 min, *t*_minor_ = 7.60 min].

##### (*R*)-(6-Methoxy-2′-(trifluoromethyl)-[1,1′-biphenyl]-2-yl)methanol (**4m**)

According to the general procedure for kinetic resolution, substrate **4m** (100 mg, 0.354 mmol) with catalyst (1*S*,3*S*)-**1** (5.43 mg, 0.007008 mmol) in acetonitrile (3.54 mL, 0.1 M) at 23–25 °C afforded aldehyde **5m** and recovered **4m** in 3 h at 63% conversion. Isolated yield: 33% (33 mg). Physical state: colorless solid, mp: 101–103 °C, *R*_f_ = 0.43 (30% EtOAc/hexane). ^1^H NMR (400 MHz, CDCl_3_): δ 7.69 (d, *J* = 8.0 Hz, 1H), 7.51 (t, *J* = 7.6 Hz, 1H), 7.41 (t, *J* = 8.0 Hz, 1H), 7.33 (t, *J* = 8.0 Hz, 1H), 7.15 (d, *J* = 7.2 Hz, 1H), 7.11 (d, *J* = 7.6 Hz, 1H), 6.82 (d, *J* = 8.0 Hz, 1H), 4.19 (AB q, *J* = 13.2 Hz, 2H), 3.61 (s, 3H). ^13^C{^1^H} NMR (101 MHz, CDCl_3_): δ 157.1, 140.5, 135.5 (q, *J* = 8.0 Hz), 132.1, 131.7, 129.9, 128.8 (q, *J* = 159.5 Hz) 127.8, 126.7, 126.4 (q, *J* = 20.4 Hz), 125.5, 122.8, 119.5, 109.9, 62.9, 55.8. IR (neat): 3243, 2927, 1461, 1253, 771, 528 cm^–1^. HRMS (ES+) *m*/*z*: [M + Na]^+^ calcd for C_15_H_13_F_3_O_2_Na, 305.0765; found, 305.0772. [α]_D_^25^ = +18.5 (*c* = 0.38, CHCl_3_). HPLC: *er* = 15.5:84.5. The *er* of the product was determined by chiral HPLC analysis using a Chiral Lux 5u cellulose-1 (250 × 4.6 mm) column [hexane/isopropanol (90:10), 1.0 mL/min, λ = 280 nm, *t*_minor_ = 6.72 min, *t*_major_ = 7.94 min].

##### (*S*)-6-Methoxy-2′-(trifluoromethyl)-[1,1′-biphenyl]-2-carbaldehyde (**5m**)

Isolated yield: 55% (55 mg). Physical state: colorless solid, mp: 88–90 °C, *R*_f_ = 0.35 (20% EtOAc/hexane). ^1^H NMR (400 MHz, CDCl_3_): δ 9.59 (s, 1H), 7.80 (d, *J* = 7.6 Hz, 1H), 7.65–7.49 (m, 4H), 7.29 (d, *J* = 7.6 Hz, 1H), 7.20 (dd, *J* = 8.4, 0.8 Hz, 1H). ^13^C{^1^H} NMR (101 MHz, CDCl_3_): δ 191.4, 157.5, 135.3, 133.1 (q, *J* = 8.4 Hz), 132.5, 131.6, 130.4, 130.0 (q, *J* = 119.1 Hz), 129.5, 128.4, 126.3 (q, *J* = 20.4 Hz), 119.3, 115.9, 56.1. IR (neat): 2931, 2842, 1697, 1465, 1311, 1110, 763 cm^–1^. HRMS (ES+) *m*/*z*: [M]^+^: calcd for C_15_H_11_F_3_O_2_: 280.0711; found: 280.0646. [α]_D_^25^ = −11.1 (*c* = 0.50, CHCl_3_). HPLC: *er* = 54.5:45.5 (10 mg of aldehyde was reduced to the corresponding alcohol with NaBH_4_ and determined the *er*). The *er* of the product was determined by a chiral HPLC Chiral Lux 5u cellulose-1 (250 × 4.6 mm) column [hexane/isopropanol (90:10), 1.0 mL/min, λ = 280 nm, *t*_major_ = 6.70 min, *t*_minor_ = 7.76 min].

##### (*R*)-(3-Methoxy-2-(naphthalen-1-yl)phenyl)methanol (**4n**)

According to the general procedure for kinetic resolution, substrate **4n** (100 mg, 0.378 mmol) with catalyst (1*S*,3*S*)-**1** (5.79 mg, 0.00756 mmol) in acetonitrile (3.78 mL, 0.1 M) at 23–25 °C afforded aldehyde **5n** and recovered **4n** in 3 h at 70% conversion. Isolated yield: 28% (28 mg). Physical state: colorless solid, mp: 101–104 °C, *R*_f_ = 0.43 (30% EtOAc/hexane). ^1^H NMR (400 MHz, CDCl_3_): δ 7.94–7.85 (m, 2H), 7.56–7.53 (m, 1H), 7.49–7.45 (m, 2H), 7.3–7.34 (m, 3H), 7.26 (d, *J* = 10.5 Hz, 1H), 7.00 (d, *J* = 8.0 Hz, 1H), 4.24 (AB q, *J* = 13.2 Hz, 2H), 3.64 (s, 3H). ^13^C{^1^H} NMR (101 MHz, CDCl_3_): δ 157.6, 141.3, 134.3, 133.8, 132.5, 129.2, 128.5, 128.0, 127.7, 127.5, 126.3, 126.0, 125.62, 125.60, 120.1, 110.4, 63.3, 56.0. IR (neat): 3243, 2927, 1461, 1253, 1037, 771, 528 cm^–1^. HRMS (ES+) *m*/*z*: [M + Na]^+^ calcd for C_18_H_16_O_2_Na, 287.1048; found, 287.1042. [α]_D_^25^ = +42.6 (*c* = 0.28, CHCl_3_). HPLC: *er* = 9:91. The *er* of the product was determined by chiral HPLC analysis using a Chiral Lux 5u cellulose-1 (250 × 4.6 mm) column [hexane/isopropanol (90:10), 1.0 mL/min, λ = 280 nm, *t*_minor_ = 6.06 min, *t*_major_ = 8.19 min].

##### (*S*)-3-Methoxy-2-(naphthalen-1-yl)benzaldehyde (**5n**)

Isolated yield: 59% (59 mg). Physical state: brown colored solid, mp: 112–115 °C, *R*_f_ = 0.59 (20% EtOAc/hexane). ^1^H NMR (400 MHz, CDCl_3_): δ 9.40 (d, *J* = 0.8 Hz, 1H), 7.84–7.82 (m, 2H), 7.63 (dd, *J* = 8.0, 1.2 Hz, 1H), 7.48–7.43 (m, 2H), 7.40–7.37 (m, 1H), 7.29–7.27 (m, 3H), 7.6 (dd, *J* = 8.0, 0.8 Hz, 1H), 3.58 (s, 3H). ^13^C{^1^H} NMR (101 MHz, CDCl_3_): δ 192.3, 157.9, 136.3, 133.5, 133.4, 133.1, 131.5, 129.3, 128.9, 128.7, 128.5, 126.6, 126.1, 126.0, 125.2, 119.1, 116.2, 56.2. IR (neat): 3359, 3054, 2842, 1685, 1585, 1461, 1253, 779 cm^–1^. HRMS (ES+) *m*/*z*: [M + Na]^+^ calcd for C_18_H_14_O_2_Na, 285.0891; found: 285.0875. [α]_D_^25^ = −48.6 (*c* = 0.50, CHCl_3_). HPLC: *er* = 62:38 (10 mg of aldehyde was reduced to the corresponding alcohol with NaBH_4_ and determined the *er*). The *er* of the product was determined by chiral HPLC using a Chiral Lux 5u cellulose-1 (250 × 4.6 mm) column [hexane/isopropanol (90:10), 1.0 mL/min, λ = 280 nm, *t*_major_ = 5.90 min, *t*_minor_ = 8.02 min].

##### (*R*)-(3-Ethoxy-2-(naphthalen-1-yl)phenyl)methanol (**4o**)

According to the general procedure for kinetic resolution, substrate **4o** (100 mg, 0.359 mmol) with catalyst (1*S*,3*S*)-**1** (5.505 mg, 0.00718 mmol) in acetonitrile (3.6 mL, 0.1 M) at 23–25 °C afforded aldehyde **5o** and recovered **4o** in 3 h at 64% conversion. Isolated yield: 33% (33 mg). Physical state: light brown colored solid, mp: 102–106 °C, *R*_f_ = 0.51 (30% EtOAc/hexane). ^1^H NMR (400 MHz, CDCl_3_): δ 7.89 (t, *J* = 8.4 Hz, 2H), 7.53 (m, 1H), 7.50–7.31 (m, 5H), 7.24 (d, *J* = 7.6 Hz, 1H), 7.00 (d, *J* = 7.6 Hz, 1H), 4.30–4.20 (AB q, *J* = 13.2 Hz 2H), 3.92 (m, 2H), 1.00 (t, *J* = 6.8 Hz, 3H). ^13^C{^1^H} NMR (101 MHz, CDCl_3_): δ 156.9, 141.3, 134.5, 133.7, 132.6, 129.1, 128.4, 127.8, 127.5, 126.1, 125.83, 125.76, 125.5, 120.1, 112.0, 64.4, 63.4, 14.6. IR (neat): 3270, 2923, 1577, 1454, 1253, 1029, 771 cm^–1^. HRMS (ES+) *m*/*z*: [M + Na]^+^ calcd for C_19_H_18_O_2_Na, 301.1205; found, 301.1191. [α]_D_^25^ = +69.7 (*c* = 0.34, CHCl_3_). HPLC: *er* = 10:90. The *er* of the product was determined by chiral HPLC analysis using a Chiral Lux 5u cellulose-1 (250 × 4.6 mm) column [hexane/isopropanol (90:10), 1.0 mL/min, λ = 280 nm, *t*_minor_ = 8.23 min, *t*_major_ = 11.76 min].

##### (*S*)-3-Ethoxy-2-(naphthalen-1-yl)benzaldehyde (**5o**)

Isolated yield: 60% (60 mg). Physical state: light brown colored solid, mp: 73–76 °C, *R*_f_ = 0.65 (20% EtOAc/hexane). ^1^H NMR (400 MHz, CDCl_3_): δ 9.51 (d, *J* = 0.8 Hz, 1H), 7.93 (d, *J* = 8.4 Hz, 2H), 7.72 (dd, *J* = 7.6, 1.2 Hz, 1H), 7.58–7.47 (m, 3H), 7.44–7.36 (m, 3H), 7.26 (dd, *J* = 8.4, 1.2 Hz, 1H), 4.03–3.91 (m, 2H), 1.04 (t, *J* = 7.2 Hz, 3H). ^13^C{^1^H} NMR (101 MHz, CDCl_3_): δ 192.5, 157.3, 136.4, 133.9, 133.5, 133.1, 131.7, 129.2, 129.0, 128.5, 128.4, 126.4, 126.2, 126.0, 125.1, 119.1, 117.7, 64.6, 14.5. IR (neat): 3054, 2969, 1681, 1581, 1249, 1060, 779 cm^–1^. HRMS (ES+) *m*/*z*: [M + Na]^+^ calcd for C_19_H_16_O_2_Na, 299.1048; found, 299.1036. [α]_D_^25^ = −68.3 (*c* = 0.5, CHCl_3_). HPLC: *er* = 62:38 (10 mg of aldehyde was reduced to the corresponding alcohol with NaBH_4_ and determined the *er*). The *er* of the product was determined by chiral HPLC using a Chiral Lux 5u cellulose-1 (250 × 4.6 mm) column [hexane/isopropanol (90:10), 1.0 mL/min, λ = 280 nm, *t*_major_ = 8.18 min, *t*_minor_ = 12.16 min].

##### (*R*)-(3-(Benzyloxy)-2-(naphthalen-1-yl)phenyl)methanol (**4p**)

According to the general procedure for kinetic resolution, substrate **4p** (100 mg, 0.294 mmol) with catalyst (1*S*,3*S*)-**1** (4.501 mg, 0.00587 mmol) in acetonitrile (2.93 mL, 0.1 M) at 23–25 °C afforded aldehyde **5p** and recovered **4p** in 3 h at 64% conversion. Isolated yield: 29% (29 mg). Physical state: colorless sticky material, *R*_f_ = 0.49 (30% EtOAc/hexane). ^1^H NMR (400 MHz, CDCl_3_): δ 7.91 (t, *J* = 8.4 Hz, 2H), 7.56 (m, 1H), 7.51–7.35 (m, 5H), 7.27 (d, *J* = 7.2 Hz, 1H), 7.15–7.10 (m, 3H), 7.02 (d, *J* = 8.4 Hz, 1H), 6.90–6.87 (m, 2H), 4.99–4.91 (m, 2H), 4.30 (s, 2H). ^13^C{^1^H} NMR (101 MHz, CDCl_3_): δ 156.5, 141.4, 137.3, 134.4, 133.8, 132.6, 129.2, 128.6, 128.5, 128.3, 128.0, 127.5, 127.5, 126.6, 126.3, 126.0, 125.8, 125.6, 120.6, 112.5, 70.3, 63.3. IR (neat): 3567, 3390, 3050, 2923, 1577, 1454, 1253, 1022, 740 cm^–1^. HRMS (ES+) *m*/*z*: [M + Na]^+^ calcd for C_24_H_20_O_2_Na, 363.1361; found, 363.1357. [α]_D_^25^ = +39.4 (*c* = 0.29, CHCl_3_). HPLC: *er* = 9.5:90.5. The *er* of the product was determined by chiral HPLC analysis using a Chiral Lux 5u cellulose-1 (250 × 4.6 mm) column [hexane/isopropanol (80:20), 1.0 mL/min, λ = 280 nm, *t*_minor_ = 8.19 min, *t*_major_ = 9.75 min].

##### (*S*)-3-(Benzyloxy)-2-(naphthalen-1-yl)benzaldehyde (**5p**)

Isolated yield: 58% (58 mg). Physical state: pale yellow sticky material, *R*_f_ = 0.59 (20% EtOAc/hexane). ^1^H NMR (400 MHz, CDCl_3_): δ 9.55 (d, *J* = 0.8 Hz, 1H), 7.94 (dd, *J* = 8.0, 0.4 Hz, 2H), 7.73 (dd, *J* = 7.6, 1.2 Hz, 1H), 7.59–7.36 (m, 6H), 7.27 (dd, *J* = 8.4, 1.2 Hz, 1H), 7.15–7.13 (m, 3H), 6.93–6.85 (m, 2H), 4.98 (s, 2H). ^13^C {^1^H} NMR (101 MHz, CDCl_3_): δ 192.3, 156.9, 136.6, 136.4, 134.2, 133.5, 133.1, 131.5, 129.3, 1290, 128.7, 128.5, 128.4, 127.7, 126.58, 126.57, 126.2, 126.18, 125.15, 119.6, 118.3, 70.6. IR (neat): 3054, 2857, 1689, 1585, 1457, 1249, 779 cm^–1^. HRMS (ES+) *m*/*z*: [M + Na]^+^ calcd for C_24_H_18_O_2_Na, 361.1205; found, 361.1198. [α]_D_^25^ = +26.9 (*c* = 0.50, CHCl_3_). HPLC: *er* = 63:37 (10 mg of aldehyde was reduced to the corresponding alcohol with NaBH_4_ and determined the *er*). The *er* of the product was determined by chiral HPLC using a Chiral Lux 5u cellulose-1 (250 × 4.6 mm) column [hexane/isopropanol (80:20), 1.0 mL/min, λ = 280 nm, *t*_major_ = 7.99 min, *t*_minor_ = 9.70 min].

##### (*R*)-(6-Methoxy-2′-methyl-[1,1′-biphenyl]-2-yl)methanol (**4q**)

According to the general procedure for kinetic resolution, substrate **4q** (100 mg, 0.438 mmol) with catalyst (1*S*,3*S*)-**1** (6.712 mg, 0.00876 mmol) in acetonitrile (4.78 mL, 0.1 M) at 23–25 °C afforded aldehyde **5q** and recovered **4q** in 3 h at 64% conversion. Isolated yield: 31% (31 mg). Physical state: pale yellow colored solid, mp: 69–72 °C, *R*_f_ = 0.51 (30% EtOAc/hexane). ^1^H NMR (400 MHz, CDCl_3_): δ 7.36 (t, *J* = 8.0 Hz, 1H), 7.28–7.20 (m, 3H), 7.15 (d, *J* = 7.3 Hz, 1H), 7.07 (d, *J* = 6.8 Hz, 1H), 6.91 (d, *J* = 8.2 Hz, 1H), 4.29 (s, 2H), 3.70 (s, 3H), 2.01 (s, 3H). ^13^C{^1^H} NMR (101 MHz, CDCl_3_): δ 156.9, 140.2, 137.0, 136.1, 130.0, 129.7, 129.1, 128.8, 127.8, 125.9, 120.0, 110.1, 63.3, 55.9, 19.8. IR (neat): 3255, 2931, 1577, 1457, 1253, 1033, 752 cm^–1^. HRMS (ES+) *m*/*z*: [M + Na]^+^ calcd for C_15_H_16_O_2_Na, 251.1048; found, 251.1031. [α]_D_^25^ = +27.7 (*c* = 0.30, CHCl_3_). HPLC: *er* = 11:89. The *er* of the product was determined by chiral HPLC analysis using a Chiral Lux 5u cellulose-1 (250 × 4.6 mm) column [hexane/isopropanol (90:10), 1.0 mL/min, λ = 280 nm, *t*_minor_ = 6.76 min, *t*_major_ = 7.05 min].

##### (*S*)-6-Methoxy-2′-methyl-[1,1′-biphenyl]-2-carbaldehyde (**5q**)

Isolated yield: 58% (58 mg). Physical state: light brown colored solid, mp: 88–90 °C, *R*_f_ = 0.62 (20% EtOAc/hexane). ^1^H NMR (400 MHz, CDCl_3_): δ 9.62 (d, *J* = 0.8 Hz, 1H), 7.63 (dd, *J* = 8.0, 0.8 Hz, 1H), 7.47 (td, *J* = 8.0, 0.8 Hz, 1H), 7.34–7.24 (m, 3H), 7.20 (dd, *J* = 8.0, 0.8 Hz, 1H), 7.13 (d, *J* = 7.2 Hz, 1H), 3.78 (s, 3H), 2.07 (s, 3H). ^13^C{^1^H} NMR (101 MHz, CDCl3): δ 192.6, 157.2, 137.4, 135.3, 134.8, 133.2, 131.0, 130.0, 128.9, 128.4, 125.6, 119.1, 116.0, 56.1, 20.1. IR (neat): 2927, 2869, 1685, 1581, 1457, 1257, 752 cm^–1^. HRMS (ES+) *m*/*z*: [M + Na]^+^ calcd for C_15_H_14_O_2_Na, 249.0891; found, 249.0880. [α]_D_^25^ = −30.7 (*c* = 0.50, CHCl_3_). HPLC: *er* = 66:34 (10 mg of aldehyde was reduced to the corresponding alcohol with NaBH_4_ and determined the *er*). The *er* of the product was determined by chiral HPLC using a Chiral Lux 5u cellulose-1 (250 × 4.6 mm) column [hexane/isopropanol (90:10), 1.0 mL/min, λ = 280 nm, *t*_major_ = 6.62 min, *t*_minor_ = 6.90 min].

##### Procedure for 1 mmol Scale OKR of Racemic Biaryl Alcohol (**4r**)

In a 25 mL single-neck round-bottom flask, racemic **4r** (325 mg, 1 mmol, 1 equiv) was dissolved in 10 mL (0.1 M) of analytical grade acetonitrile. Copper(I) bromide (2.87 mg, 0.02 mmol, 0.02 equiv), bipyridine (3.12 mg, 0.02 mmol, 0.02 equiv), hydroxylamine precatalyst (1*S*,3*S*)-**1** (15.32 mg, 0.02 mmol, 0.02 equiv), and *N*-methyl imidazole [3.28 mg, 0.04 mmol, 0.04 equiv (0.4 mL of 0.1 M acetonitrile solution)] were added to the reaction flask. The resulting brown colored reaction mixture was stirred at room temperature (23–25 °C) open to air. The reaction progress was monitored by ^1^H NMR analysis of the crude reaction mixture. After 3.5 h of oxidation, at 55% conversion, the reaction was stopped. The acetonitrile was removed from the reaction mixture under reduced pressure at room temperature on a rotary evaporator. The crude reaction mixture was subjected to flash column chromatography using hexane and ethyl acetate as the eluent to obtain recovered alcohol **4r** and the product aldehyde **5r**.

##### (*R*)-(2′-Iodo-6-methyl-[1,1′-biphenyl]-2-yl)methanol (**4r**)

Isolated yield: 22% (73 mg). Physical state: light yellow colored solid, mp: 81–84 °C, *R*_f_ = 0.4 (30% EtOAc/hexane). ^1^H NMR (400 MHz, CDCl_3_): δ 7.96 (dd, *J* = 8.0, 0.8 Hz, 1H), 7.45–7.40 (m, 2H), 7.36 (t, *J* = 7.6 Hz, 1H), 7.24 (d, *J* = 7.6 Hz, 1H), 7.17 (dd, *J* = 7.6, 1.6 Hz, 1H), 7.10–7.05 (m, 1H), 4.30 (AB q, *J* = 13.2 Hz, 2H), 1.98 (s, 3H). ^13^C{^1^H} NMR (101 MHz, CDCl_3_): δ 144.6, 142.7, 139.3, 138.3, 136.1, 129.9, 129.4, 129.1, 128.7, 128.4, 125.2, 100.6, 63.5, 20.2. IR (neat): 3301, 2911, 1446, 1060, 1002, 755 cm^–1^. HRMS (ES+) *m*/*z*: [M + Na]^+^ calcd for C_14_H_13_OINa, 346.9909; found, 346.9898. [α]_D_^25^ = +14.0 (*c* = 0.60, CHCl_3_). HPLC: *er* = 6.5:93.5. The *er* of the product was determined by chiral HPLC analysis using a Lux 5 μm cellulose-1 (250 × 4.6 mm) column [hexane/isopropanol (80:20), 0.7 mL/min, λ = 280 nm, *t*_minor_ = 6.17 min, *t*_major_ = 6.80 min].

##### (*S*)-2′-Iodo-6-methyl-[1,1′-biphenyl]-2-carbaldehyde (**5r**)

Isolated yield: 73% (231 mg). Physical state: brown colored liquid, *R*_f_ = 0.70 (20% EtOAc/hexane). ^1^H NMR (400 MHz, CDCl_3_): δ 9.62 (d, *J* = 0.4 Hz, 1H), 7.98 (dd, *J* = 8.0, 1.2 Hz, 1H), 7.87 (dd, *J* = 7.7, 0.8 Hz, 1H), 7.54 (dd, *J* = 7.6, 0.4 Hz, 1H), 7.48–7.43 (m, 2H), 7.22 (dd, *J* = 7.6, 1.6 Hz, 1H), 7.13 (td, *J* = 7.6, 1.6 Hz, 1H), 2.05 (s, 3H). ^13^C{^1^H} NMR (101 MHz, CDCl_3_): δ 192.0, 147.2, 142.2, 139.3, 137.2, 135.8, 133.8, 130.4, 129.7, 128.5, 128.5, 125.2, 100.4, 19.7. IR (neat): 3054, 2850, 1689, 1450, 1241, 1002, 748 cm^–1^. HRMS (ES+) *m*/*z*: [M + Na]^+^ calcd for C_14_H_11_OINa, 344.9752; found, 344.9739. [α]_D_^25^ = +11.5 (*c* = 0.60, CHCl_3_). HPLC: *er* = 62.5:37.5 (10 mg of aldehyde was reduced to the corresponding alcohol with NaBH_4_ and determined the *er*). The *er* of the product was determined by chiral HPLC analysis using a Lux 5 μm cellulose-1 (250 × 4.6 mm) column [hexane/isopropanol (90:10), 1.0 mL/min, λ = 254 nm, *t*_major_ = 6.69 min, *t*_minor_ = 7.49 min].

##### General Procedure for the Desymmetrization of Diols (**6a–c**)

In a 20 mL glass vial, 100 mg of prochiral diol-**6** (1 equiv) was dissolved in analytical grade acetonitrile (0.1 M). CuBr (2 mol %), bipyridine (2 mol %), chiral hydroxylamine (1*S*,3*S*)-**1** (2 mol %), and *N*-methylimidazole (4 mol %, 0.1 M acetonitrile solution) were added to the reaction mixture. The resulting red/brown colored reaction mixture was stirred at RT (23–25 °C) open to air for specified time. After the specified time, the solvent from the reaction mixture was removed under the reduced pressure. The crude reaction mixture was subjected to flash column chromatography using hexane and ethyl acetate as the eluent to afford chiral mono aldehyde **7** and dialdehyde 8.

##### 6-(Hydroxymethyl)-2′-(trifluoromethyl)-[1,1′-biphenyl]-2-carbaldehyde (**7a**)

According to the general procedure for desymmetrization, substrate **6a** (100 mg, 0.354 mmol) with catalyst (1*S*,3*S*)-**1** (5.43 mg, 0.00708 mmol) in acetonitrile (3.5 mL, 0.1 M) at 23–25 °C afforded mono aldehyde **7a** and dialdehyde **8a** (18 mg) in 3 h. Isolated yield: 58% (58 mg). Physical state: colorless sticky material, *R*_f_ = 0.31 (30% EtOAc/hexane). ^1^H NMR (400 MHz, CDCl_3_): δ 9.56 (s, 1H), 7.94 (dd, *J* = 8.0, 1.6 Hz, 1H), 7.87–7.85 (m, 1H), 7.82 (dd, *J* = 7.6, 0.8 Hz, 1H), 7.66–7.56 (m, 3H), 7.30 (d, *J* = 7.6 Hz 1H), 4.33 (AB q, *J* = 13.2 Hz, 2H). ^13^C{^1^H} NMR (101 MHz, CDCl_3_): δ 191.3, 140.4, 139.8, 134.5, 134.3, 132.9, 132.0, 131.9, 129.5 (q, *J* = 30 Hz), 129.1, 128.9, 126.97, 126.5 (q, *J* = 5.4 Hz), 125.2 (q, *J* = 273.7 Hz), 62.1. ^19^F NMR (376 MHz, CDCl_3_): δ −59.6. IR (neat): 3401, 2857, 1681, 1454, 1311, 1114, 767 cm^–1^. HRMS (ES+) *m*/*z*: [M + Na]^+^ calcd for C_15_H_11_F_3_O_2_Na, 303.0609; found, 303.0596. [α]_D_^25^ = +11.1 (*c* = 0.40, CHCl_3_). HPLC: *er* = 8:92. The *er* was determined by chiral HPLC analysis using a Chiral Pak AD-H (250 × 4.6 mm) column [hexane/isopropanol (90:10), 1.0 mL/min, λ = 254 nm, *t*_minor_ = 9.88 min, *t*_major_ = 11.59 min].

##### 3-(Hydroxymethyl)-2-(naphthalen-1-yl)benzaldehyde-(**7b**)

According to the general procedure for desymmetrization, substrate **6b** (100 mg, 0.378 mmol) with catalyst (1*S*,3*S*)-**1** (5.79 mg, 0.00756 mmol) in acetonitrile (3.8 mL, 0.1 M) at 23–25 °C afforded monoaldehyde **7b** and dialdehyde **8b** (28 mg) in 6 h. Isolated yield: 68% (68 mg). Physical state: brown colored sticky material, *R*_f_ = 0.37 (30% EtOAc/hexane). ^1^H NMR (400 MHz, CDCl_3_): δ 9.32 (d, *J* = 0.8 Hz, 1H), 7.92 (dd, *J* = 7.6, 1.2 Hz, 1H), 7.89–7.76 (m, 3H), 7.50 (t, *J* = 7.6 Hz, 1H), 7.46–7.37 (m, 2H), 7.29–7.24 (m, 2H), 7.15–7.13 (m, 1H), 4.18 (AB q, *J* = 13.5 Hz, 2H). ^13^C{^1^H} NMR (101 MHz, CDCl_3_): δ 192.5, 142.5, 141.3, 135.4, 133.8, 133.4, 133.2, 133.0, 129.3, 129.0, 128.99, 128.5, 127.51, 126.9, 126.7, 125.8, 125.6, 62.6. IR (neat): 3417, 3054, 2857, 1685, 1589, 1238, 779 cm^–1^. HRMS (ES+) *m*/*z*: [M + Na]^+^ calcd for C_18_H_14_O_2_Na, 285.0891; found, 285.0876. [α]_D_^25^ = −24.0 (*c* = 0.68, CHCl_3_). HPLC: *er* = 4.5:95.5. The *er* was determined by chiral HPLC analysis using a Chiral Pak AD-H (250 × 4.6 mm) column [hexane/isopropanol (90:10), 1.0 mL/min, λ = 254 nm, *t*_minor_ = 12.70 min, *t*_major_ = 13.75 min].

##### 6-(Hydroxymethyl)-[1,1′:2′,1″-terphenyl]-2-carbaldehyde-(**7c**)

According to the general procedure for desymmetrization, substrate **6c** (100 mg, 0.344 mmol) with catalyst (1*S*,3*S*)-**1** (5.28 mg, 0.00687 mmol) in acetonitrile (3.5 mL, 0.1 M) at 23–25 °C afforded monoaldehyde **7c** and dialdehyde **8c** (22 mg) in 7 h. Isolated yield: 73% (73 mg); brown colored sticky material, *R*_f_ = 0.43 (30% EtOAc/hexane). ^1^H NMR (400 MHz, CDCl_3_): δ 9.88 (d, *J* = 0.8 Hz, 1H), 7.94 (dd, *J* = 7.6, 1.2 Hz, 1H), 7.83–7.81 (m, 1H), 7.67–7.63 (m, 2H), 7.58–7.51 (m, 2H), 7.40–7.38 (m, 1H), 7.28–7.25 (m, 3H), 7.17–7.14 (m, 2H), 4.52 (d, *J* = 13.5 Hz, 1H), 4.41 (d, *J* = 13.5 Hz, 1H). ^13^C{^1^H} NMR (101 MHz, CDCl_3_): δ 192.3, 143.0, 141.7, 140.2, 139.9, 134.2, 133.6, 133.1, 131.1, 130.3, 129.2, 129.0, 128.2, 128.2, 127.4, 127.2, 126.5, 62.1. IR (neat): 3421, 2857, 1681, 1582, 1052, 748 cm^–1^. HRMS (ES+) *m*/*z*: [M + Na]^+^ calcd for C_20_H_16_O_2_Na, 311.1048; found, 311.1037. [α]_D_^25^ = +25.5 (*c* = 0.60, CHCl_3_). HPLC: *er* = 99:1. The *er* was determined by chiral HPLC analysis using a cellulose-1, OD-H (250 × 4.6 mm) column [hexane/isopropanol (90:10), 1.0 mL/min, λ = 254 nm, *t*_major_ = 26.54 min, *t*_minor_ = 35.04 min].

## Data Availability

The data underlying this study are available in the published article and its Supporting Information.

## Supporting Information Available

Preparation of starting materials, copies of NMR spectra and HPLC chromatograms, and X-ray structure of (*R*)-**2e** (PDF)
