# Supplementary material for: Catalytic Atroposelective Aerobic Oxidation Approaches to Axially Chiral Molecules
Source: J Org Chem. 2023 Jun 16;88(13):8413–30. doi: 10.1021/acs.joc.3c00417 (PMC10337035; doi:10.1021/acs.joc.3c00417)
Supplement: Supplementary file 1 — jo3c00417_si_001.pdf [file jo3c00417_si_001.pdf]

Supporting Information

Catalytic Atroposelective Aerobic Oxidation Approaches to Axially Chiral Molecules

Lenin Kumar Verdhi,[a] Asit Ghosh,[a] Natalia Fridman,[b] and Alex M. Szpilman*[a]

The Department of Chemical Sciences, Ariel University, 4070000 Ariel, Israel.

[b]Schulich Faculty of Chemistry, Technion – Israel Institute of Technology, Haifa 3200009, Israel.

Email: amszpilman@gmail.com

Table of Contents

(1) Synthesis of racemic biaryl aldehydes S-2
(2) General Procedure for the synthesis of *rac*-biaryl alcohols S-4
(3) Synthesis of racemic biaryl alcohol **4r** S-4
(4) Synthesis of biaryl diols **6a-c** (substrates for desymmetrization) S-5
(5) Optimization of reaction conditions for OKR of biaryl alcohol **4a** S-9
(6) Chiral HPLC traces of the products S-14
(7) X-ray structure of (R)-**7e** S-55
(8) References S-56
(9) ^{1}H and ^{13}C NMR Spectra of Products S-57

1. Synthesis of racemic biaryl aldehydes

General procedure A[1]

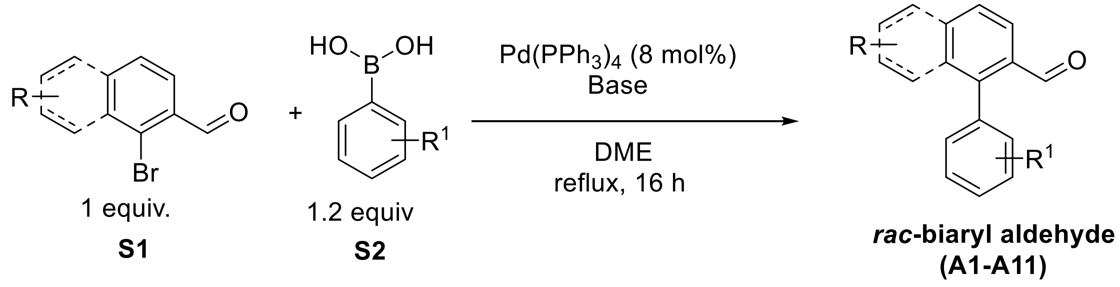

Base = 2M Na_2CO_3 solution (8 equiv) and EtOH
(or) CsF (3.5 equiv)

$Pd(PPh_3)_4$ (0.08 equiv.) was added to a mixture of 2-bromobenzaldehyde derivative **S1** (1 equiv.), aryl boronic acid derivative **S2** (1.2 equiv.), 2M aqueous Na_2CO_3 solution (8 equiv.) with EtOH (or) CsF (3.5 equiv) in DME. The resulting reaction mixture was heated at reflux temperature on oil bath for 16 h under an inert atmosphere. Upon completion, the reaction mixture was cooled to RT, and the solvents were removed under reduced pressure. The obtained residue was extracted with EtOAc: H_2O (1:1). The combined EtOAc layers were washed once with saturated brine solution and separated. The organic layer was dried over sodium sulfate, filtered, and concentrated in *vacuo*. The crude product was purified by flash column chromatography on silica gel to afford the desired biaryl aldehyde in 80-85% yields.

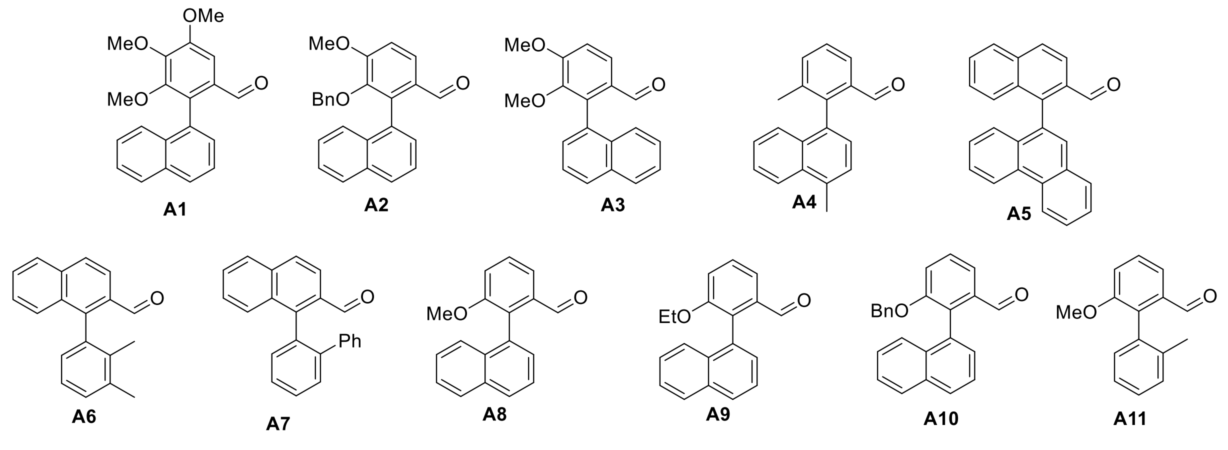

Compounds **A6** and **A7** were synthesized using CsF

General procedure B

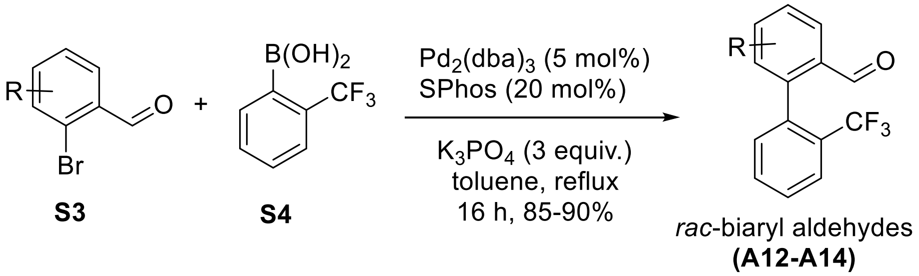

2-bromobenzaldehhyde derivative **S3** (1.5 mmol, 1 equiv.), (2-triflurometthyl)-phenyl boronic acid **S4** (3 mmol, 2 equiv.), $Pd_2(dba)_3$ (0.075 mol, 0.05 equiv.), SPhos (0.3 mmol, 0.2 equiv.) and K_3PO_4 (4.5 mmol, 3 equiv.) were charged to a 100 ml round bottom flask under positive nitrogen pressure. The reaction flask was evacuated and refilled twice with nitrogen. Toluene (12 mL) was added to the reaction mixture and heated at reflux temperature on oil bath for 18 h. The reaction mixture was cooled to RT, filtered through celite, and washed with toluene. Filtrate was concentrated in vacuo and the obtained residue was purified by flash column chromatography on silica gel to afford the compounds **A12-A13** (85-90% yield) as a colorless solid**.**

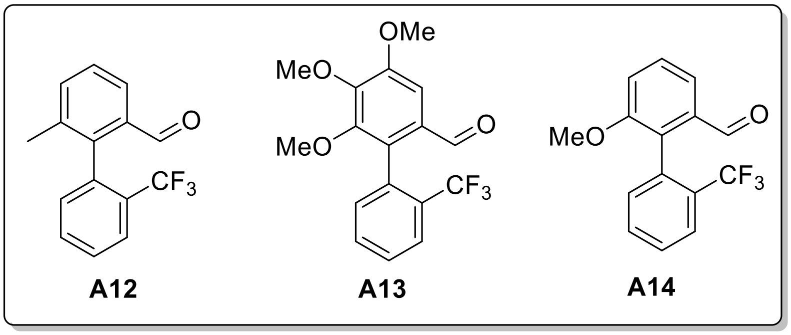

General procedure C

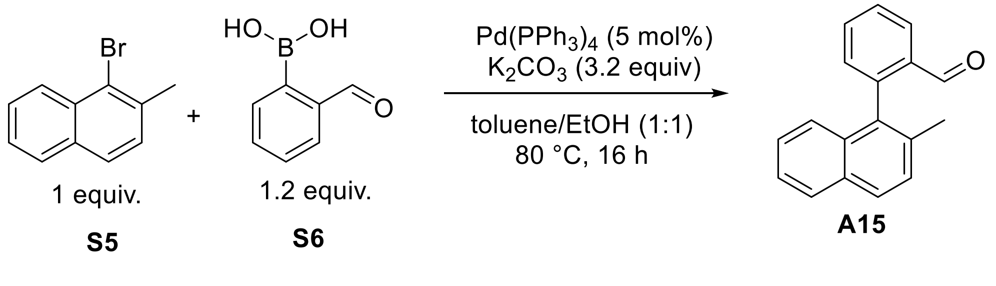

Aryl bromide **S5** (0.64 g, 2.78 mmol, 1 equiv.), aryl boronic acid **S6** (0.5 g, 3.33 mmol, 1.2 equiv.), K_2CO_3 (1.23 g, 8.9 mmol, 3.2 equiv.) and $Pd(PPh_3)_4$ (0.16 g, 0.14 mmol, 0.05 equiv.) were added to a round bottom flask under positive argon flow. A solution of toluene/ethanol (1:1) (10 mL)

was added to the reaction mixture and heated the reaction mixture on an oil bath to 80 °C for 16 h. The reaction mixture was cooled to RT, diluted with EtOAc, and washed with water. The separated EtOAc layer was dried over anhydrous sodium sulfate, filtered, and concentrated. The crude product was purified by flash column chromatography on silica gel (2-5% EtOAc/Hexane) to obtain the desired product (0.65 g, 90%).

2. General Procedure for synthesis of *rac*-biaryl alcohols (4c-q)

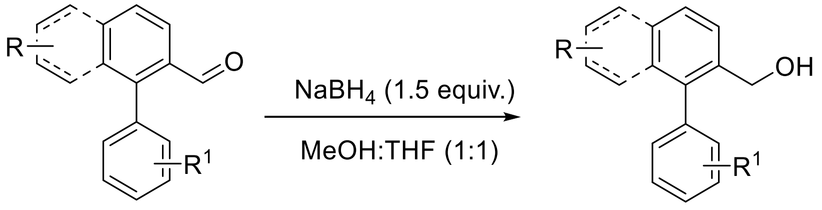

A1-A15 *rac*-**4(c-q)**

Racemic-biaryl aldehyde (1 equiv.) was dissolved in a mixture of methanol/THF (1:1) and cooled in an ice bath. $NaBH_4$ (1.5 equiv.) was added to the reaction mixture as a portion wise over a period of 10 min and allowed the reaction mixture to stir at RT until reduction completed. The reaction mixture was quenched with saturated ammonium chloride solution and stirred for 5 min. Solvents were removed from the reaction mixture in vacuo and extracted the aqueous layer with EtOAc. The combined organic layers were dried over anhydrous sodium sulfate, filtered, and concentrated. The crude product was subjected to silica gel flash column chromatography to afford the desired rac-biaryl alcohol in 85-90% yields.

3. Synthesis of racemic biaryl alcohol 4r

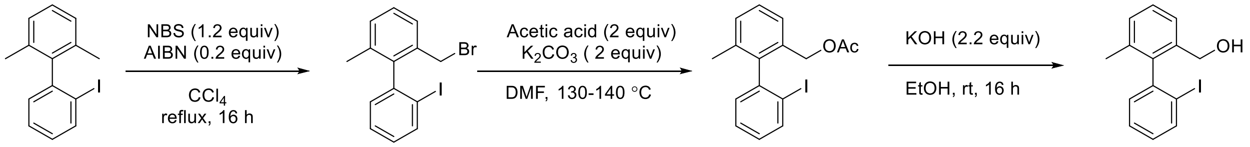

S7 **S8** **S9** **4r**

Step-1:

0.78 g (2.53 mmol, 1 equiv) of biaryl compound[2] **S7** was dissolved in CCl_4 (20 mL). N-bromosuccinimide (1.2 equiv) and AIBN (0.2 equiv) were added to the reaction flask. The reaction mixture was heated on an oil bath to reflux 16 hours. Cooled the reaction mixture to room temperature, filtered and washed with CCl_4 (2X10 mL). The filtrate was evaporated to dryness. The obtained crude product was subjected to silica gel chromatography using hexane as an eluent to afford the **S8**.

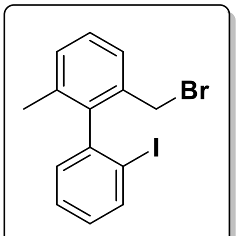

S8

Isolated yield: 56% (0.55 g); colorless solid.

^{1}H NMR (400 MHz, $CDCl_3$): δ 7.98 (dd, J = 8.0, 1.2 Hz, 1H), 7.47 (td, J = 7.6, 1.2 Hz, 1H), 7.40 – 7.36 (m, 1H), 7.33 – 7.29 (m, 2H), 7.27 – 7.24 (m, 3H), 7.11 (ddd, J = 7.9, 7.5, 1.7 Hz, 1H), 4.33 (d, J = 10.0 Hz, 2H), 4.02 (d, J = 10.0 Hz, 1H), 1.98 (s, 3H). **^{13}C NMR (101 MHz, CDCl3):** δ 143.9, 143.8, 139.3, 136.9, 135.4, 130.4, 130.4, 129.4, 128.6, 128.5, 128.1, 100.6, 32.4, 20.5.

Step-2:

0.55 g (1.41 mmol, 1 equiv) of **S8** was dissolved in 10 mL of DMF. Acetic acid (2 equiv) and dry K_2CO_3 (2 equiv) were added to the reaction mixture. The resulted reaction mixture was heated on an oil bath to 140 °C for 3 hours. The reaction mixture was cooled to room temperature and 30 mL was added. After stirring for 10 minutes, the aqueous layer was extracted with ethyl acetate (3X20 mL) and separated. The combined organic layers were dried over sodium sulfate, filtered, and concentrated under reduced pressure to get the crude acetate **S9**. The crude product for taken for next step without purification.

Step-3:

The crude acetate **S9** was dissolved in 15 mL of ethanol and solid KOH (2.2 equiv) was added. The reaction mixture was stirred at room temperature for 16 hours. The reaction mixture was concentrated to dryness and 20 mL of water. The aqueous layer was extracted with ethyl acetate (3X15 mL) and separated. The combined organic layers were dried over sodium sulfate, filtered, and concentrated under reduced pressure. The crude product was purified by silica gel column chromatography to obtain the pure alcohol **4r.**

4. Synthesis of biaryl diols (substrates for desymmetrization) (6a-c)

Synthesis of 6a:

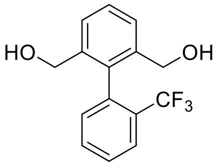

6a

Biaryl diol is a known compound, and it is synthesized by using previously reported procedure.[3]

Synthesis of 6b:

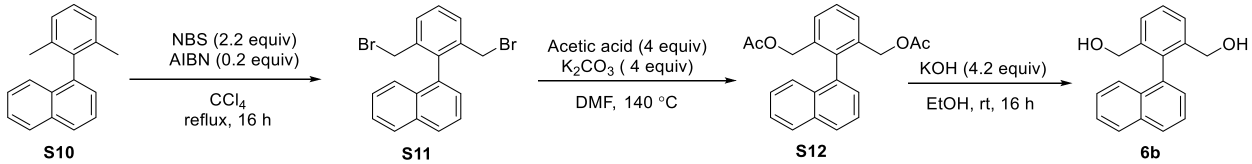

Step-1:

0.54 g (2.32 mmol, 1 equiv) of biaryl compound[4] **S10** was dissolved in CCl_4 (20 mL). N-bromosuccinimide (2.2 equiv) and AIBN (0.2 equiv) were added to the reaction flask. The reaction mixture was heated on an oil bath to reflux 16 hours. Cooled the reaction mixture to room temperature, filtered and washed with CCl_4 (2X10 mL). The filtrate was evaporated to dryness. The obtained crude product was subjected to silica gel chromatography using 1-2% ethyl acetate in hexane as an eluent to afford dibromide **S11**.

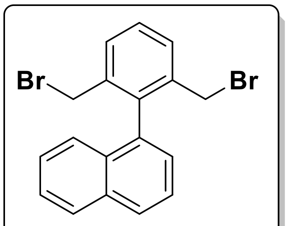

S11

Isolated yield: 87% (0.79 g); colorless solid

^{1}H NMR (400 MHz, $CDCl_3$): δ 7.91 (t, J = 9.2 Hz, 2H), 7.60 – 7.41 (m, 6H), 7.36-7.32 (m, 1H), 7.24 – 7.20 (m, 1H), 4.16 (d, J = 10.0 Hz, 2H), 3.92 (d, J = 10.4 Hz, 2H). **^{13}C NMR (101 MHz, CDCl3):** δ 139.6, 137.6, 133.7, 133.6, 132.1, 130.8, 128.98, 128.8, 128.5, 128.0, 126.5, 126.3, 125.5, 125.4, 31.6.

Sep-2:

0.53 g (1.35 mmol, 1 equiv) of **S11** was dissolved in 10 mL of DMF. Acetic acid (4 equiv) and dry K_2CO_3 (4 equiv) were added to the reaction mixture. The resulted reaction mixture was heated on an oil bath to 140 °C for 3 hours. The reaction mixture was cooled to room temperature and 30 mL was added. After stirring for 10 minutes, the aqueous layer was extracted with ethyl acetate (3X20 mL) and separated. The combined organic layers were dried over sodium sulfate, filtered, and concentrated under reduced pressure to get the crude diacetate **S12**. The crude product for taken for next step without purification.

Step-3:

The crude diacetate **S12** was dissolved in 15 mL of ethanol and solid KOH (4.2 equiv) was added. The reaction mixture was stirred at room temperature for 16 hours. The reaction mixture was concentrated to dryness and 20 mL of water. The aqueous layer was extracted with ethyl acetate (3X15 mL) and separated. The combined organic layers were dried over sodium sulfate, filtered, and concentrated under reduced pressure. The crude product was purified by silica gel column chromatography to using 50-60% ethyl acetate in hexane as an eluent to obtain the pure diol **4b.**

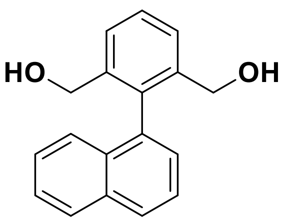

6b

isolated yield: 42% (151 mg), Colorless solid.

Compound **6b** is a known compound, and ^{1}H and ^{13}C data matches with the reported literature.[3]

Synthesis of 6c:

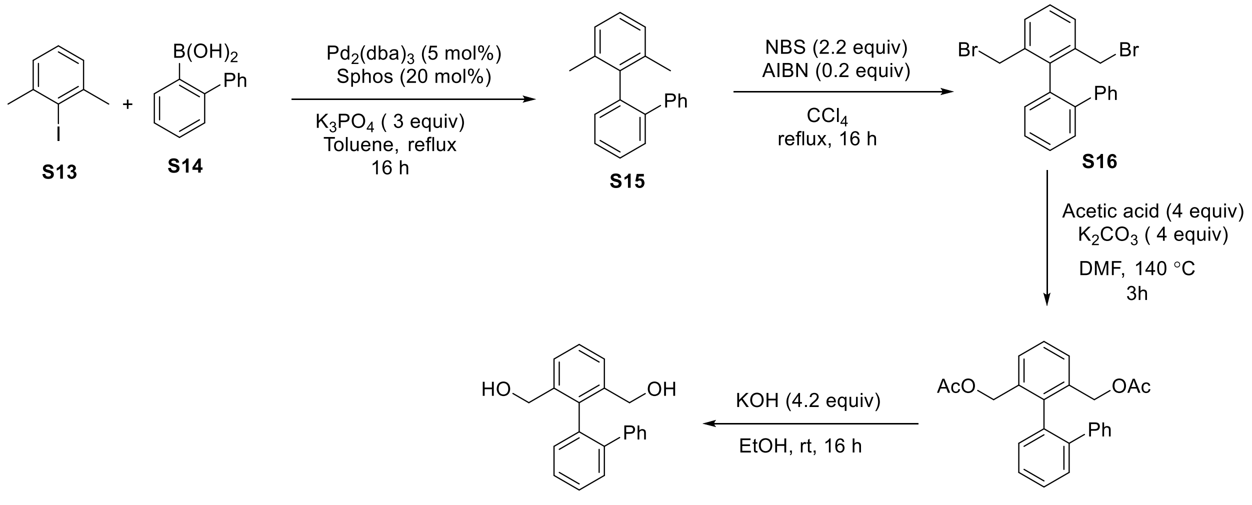

Step-1:

Iodoarene **S13** (0.58 g, 1 equiv), boronic acid **S14** (0.99 g, 2 equiv), $Pd_2(dba)_3$ (0.115 g, 0.05 equiv), SPhos (0.206 g, 0.2 equiv) and K_3PO_4 (1.595 g, 3 equiv) were charged to a 100 ml round bottom flask under positive nitrogen pressure. The reaction flask was evacuated and refilled twice with nitrogen. Toluene (12 mL) was added to the reaction mixture and heated at reflux temperature on oil bath for 18 h. The reaction mixture was cooled to RT, filtered through celite, and washed with toluene. Filtrate was concentrated in vacuo and the obtained residue was purified by flash column chromatography on silica gel to afford the compound **S15** as a colorless solid.

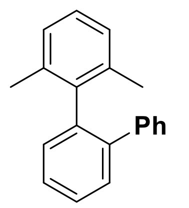

S15

isolated yield: 89% (575 mg), Colorless solid.

^{1}H NMR (400 MHz, CDCl3): δ 7.50 – 7.33 (m, 3H), 7.20 – 7.03 (m, 7H), 6.96 (d, *J* = 7.6 Hz, 2H), 1.94 (s, 6H). **^{13}C NMR (101 MHz, CDCl3):** δ 141.4, 140.9, 140.9, 139.1, 136.2, 130.5, 130.3, 128.9, 127.8, 127.6, 127.5, 127.3, 127.1, 126.7, 20.9.

Step-2:

0.57 g (2.23 mmol, 1 equiv) of biaryl compound **S15** was dissolved in CCl_4 (30 mL). N-bromosuccinimide (2.2 equiv) and AIBN (0.2 equiv) were added to the reaction flask. The reaction mixture was heated on an oil bath to reflux 16 hours. Cooled the reaction mixture to room temperature, filtered and washed with CCl_4 (2X10 mL). The filtrate was evaporated to dryness. The obtained crude product was taken for next step without purification.

Step-3:

Crude **S16** was dissolved in 10 mL of DMF. Acetic acid (4 equiv) and dry K_2CO_3 (4 equiv) were added to the reaction mixture. The resulted reaction mixture was heated on an oil bath to 140 °C for 3 hours. The reaction mixture was cooled to room temperature and 30 mL was added. After stirring for 10 minutes, the aqueous layer was extracted with ethyl acetate (3X20 mL) and separated. The combined organic layers were dried over sodium sulfate, filtered, and concentrated under reduced pressure to get the crude diacetate **S17**. The crude product for taken for next step without purification.

Step-4:

The crude diacetate **S17** was dissolved in 15 mL of ethanol and solid KOH (4.2 equiv) was added. The reaction mixture was stirred at room temperature for 16 hours. The reaction mixture was concentrated to dryness and 20 mL of water. The aqueous layer was extracted with ethyl acetate (3X15 mL) and separated. The combined organic layers were dried over sodium sulfate, filtered, and concentrated under reduced pressure. The crude product was purified by silica gel column chromatography using 50-60% ethyl acetate in hexane as an eluent to obtain the pure diol **6c.**

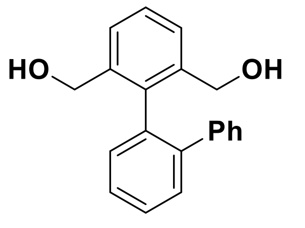

6c

isolated yield: 20% (119 mg), Colorless sticky material.

^{1}H NMR (400 MHz, CDCl3): δ 7.51 – 7.44 (m, 2H), 7.40 – 7.27 (m, 4H), 7.18 – 7.06 (m, 6H), 4.29 (d, *J* = 13.2 Hz, 2H), 4.20 (d, *J* = 13.2 Hz, 2H).**^{13}C NMR (101 MHz, CDCl3):** δ 140.7, 140.5, 138.8, 137.8, 135.98, 130.6, 130.3, 129.1, 128.4, 128.1, 128.0, 127.5, 127.1, 126.7, 63.0.

5. Optimization of reaction conditions for OKR of biaryl alcohol 4a

Table S1. Optimization of Reaction Conditions[a]

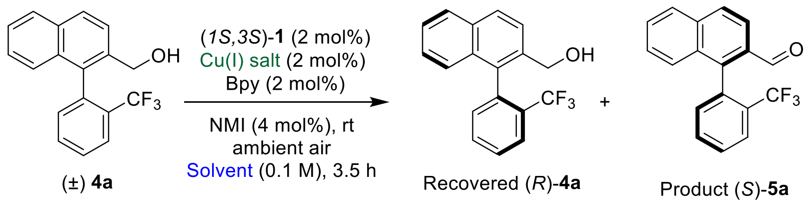

entry	solvent	Cu(I)-salt	*t* (h)	C[b]	(*R*)-**4a** (%)[c]	(*S*)-**5a** (%)[c]	*er* **4a**	*er* **5a**	s
1	MeCN	CuBr	2.5	64	28	54	5.5:94.5	60:40	8.4
2	DCM	CuBr	3.5	75	19	72	23.5:76.5	55:45	2.2
3	EtOAc	CuBr	20	4	--	--	--	--	--
4	DMF	CuBr	6	28	69	23	34.5:65.5	90:10	12.0
5	THF	CuBr	20	2	--	--	--	--	--
6	MeCN	CuCl	5	38	55	34	42.5:57.5	60.5:39.5	1.9
7	MeCN	CuI	4.5	19	74	17	45.5:54.5	61.5:38.5	2.4

[a]Unless otherwise stated, reactions were carried out with 100 mg of **4a**, (*1S,3S*)-**1** (2 mol%), Cu(I)-salt (2 mol%), bipyridine (2 mol%) and *N*-methyl imidazole (4 mol%) in solvent (0.1 M). [b]C represents conversion (by ^{1}H NMR analysis). Enantiomeric ratio (*er)* values were determined by chiral HPLC. In all cases, 10 mg of product **5** was reduced to the corresponding alcohol and determined *er*. [c]Isolated yields are mentioned. The selectivity factor (*s*) was calculated by $s = \ln[(1\text{-}C)(1\text{-}ee)]/\ln[(1\text{-}C)(1\text{+}ee)]$ where ee = enantiomeric excess of recovered alcohol **4a**.

*Chiral HPLC chromatograms of rac **4a**, recovered (R)-**4a** and aldehyde (S)-**5a** for different solvents*

Solvent : DCM

<Chromatogram>

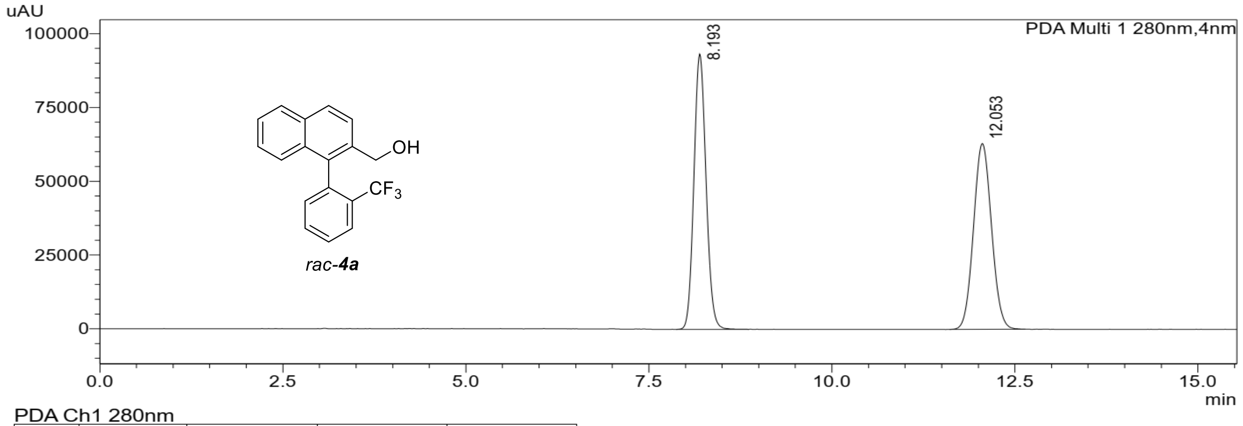

PDA Ch1 280nm

Peak#	Ret. Time	Area	Area%	Height
1	8.193	1077207	50.004	93240
2	12.053	1077029	49.996	62987
Total		2154235	100.000	156226

<Chromatogram>

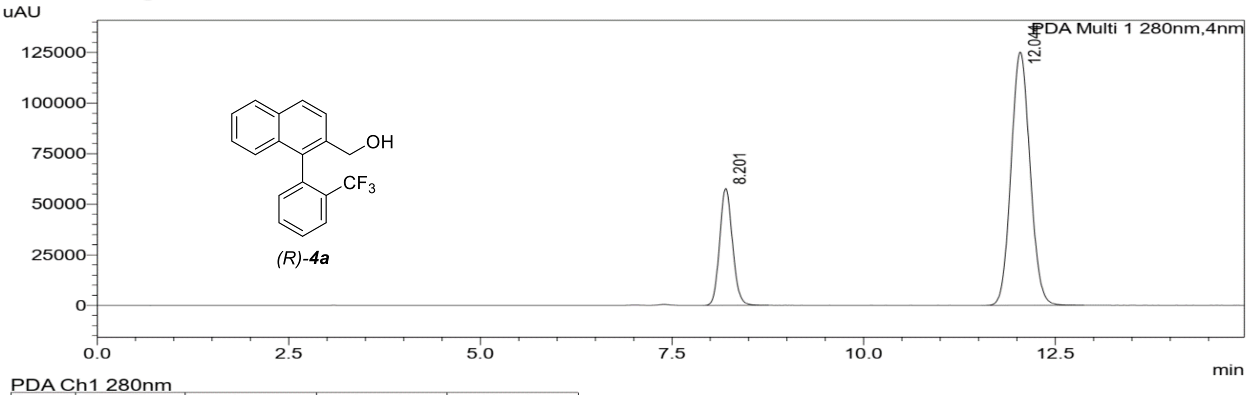

PDA Ch1 280nm

Peak#	Ret. Time	Area	Area%	Height
1	8.201	672981	23.760	57727
2	12.044	2159423	76.240	125159
Total		2832404	100.000	182886

<Chromatogram>

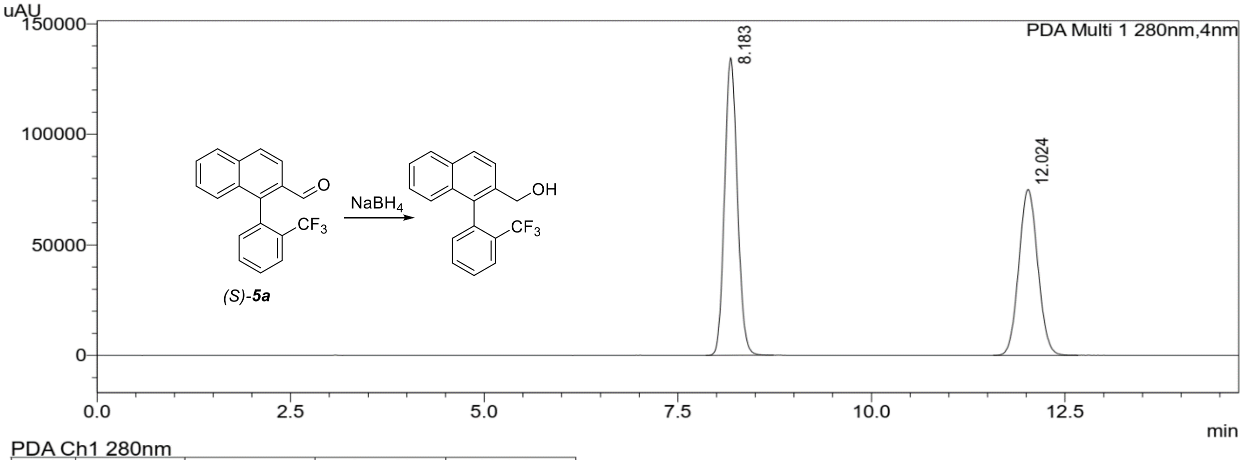

PDA Ch1 280nm

Peak#	Ret. Time	Area	Area%	Height
1	8.183	1549009	54.769	134408
2	12.024	1279253	45.231	75002
Total		2828262	100.000	209411

Solvent : DMF

[illegible]

<Chromatogram>

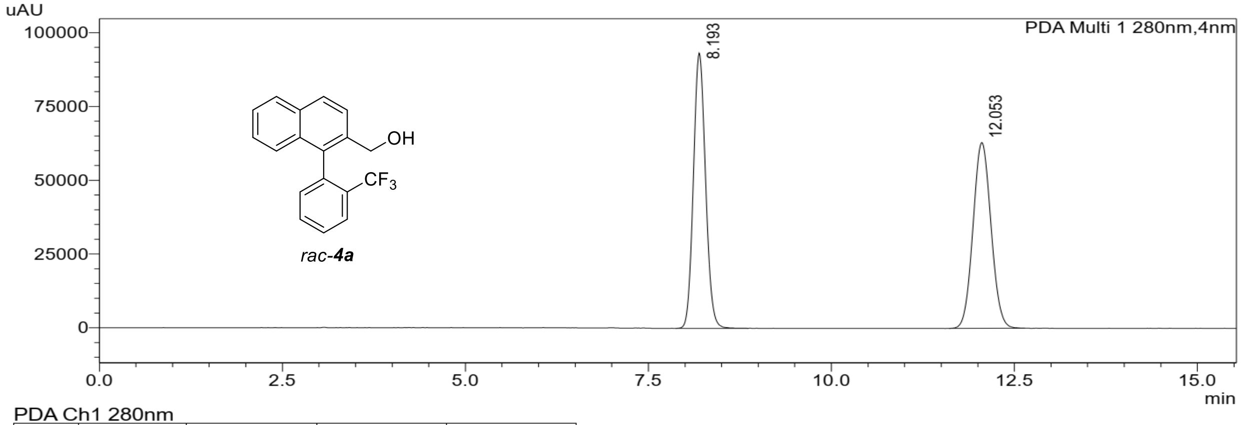

PDA Ch1 280nm

Peak#	Ret. Time	Area	Area%	Height
1	8.193	1077207	50.004	93240
2	12.053	1077029	49.996	62987
Total		2154235	100.000	156226

<Chromatogram>

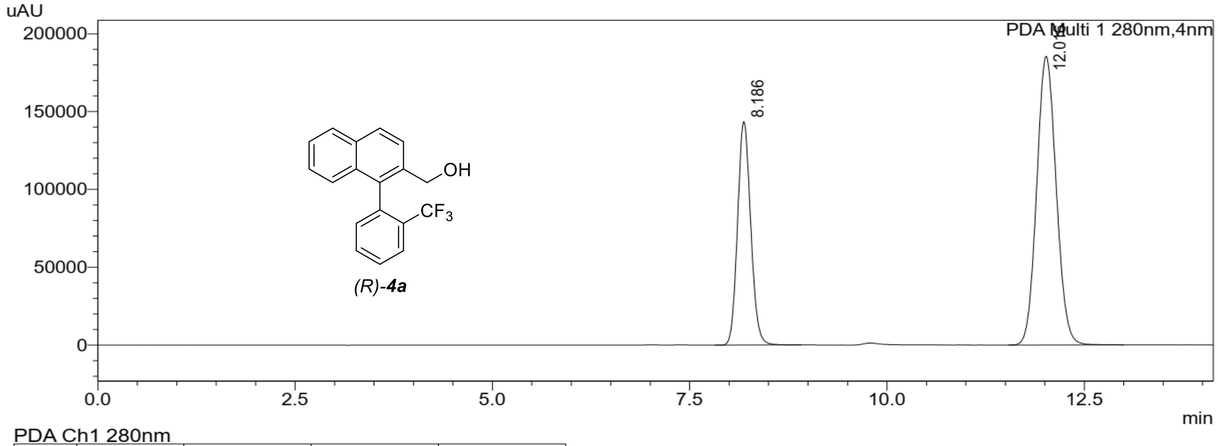

PDA Ch1 280nm

Peak#	Ret. Time	Area	Area%	Height
1	8.186	1668982	34.339	143429
2	12.016	3191319	65.661	185269
Total		4860302	100.000	328697

<Chromatogram>

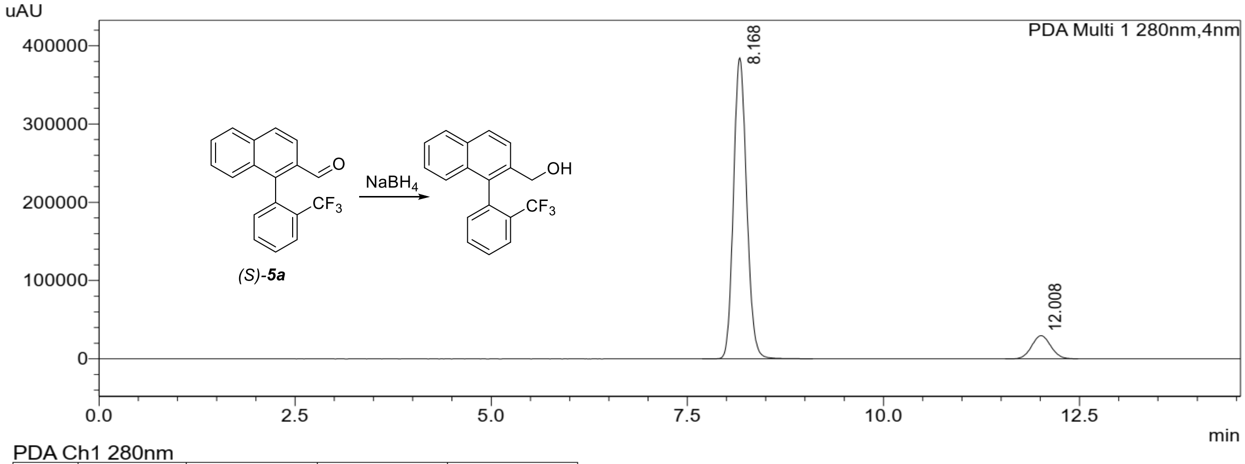

PDA Ch1 280nm

Peak#	Ret. Time	Area	Area%	Height
1	8.168	4486663	89.933	384302
2	12.008	502235	10.067	29530
Total		4988898	100.000	413832

*Chiral HPLC chromatograms of rac **4a**, recovered (R)-**4a** and aldehyde (S)-**5a** for different Cu(I)*

CuCl

<Chromatogram>

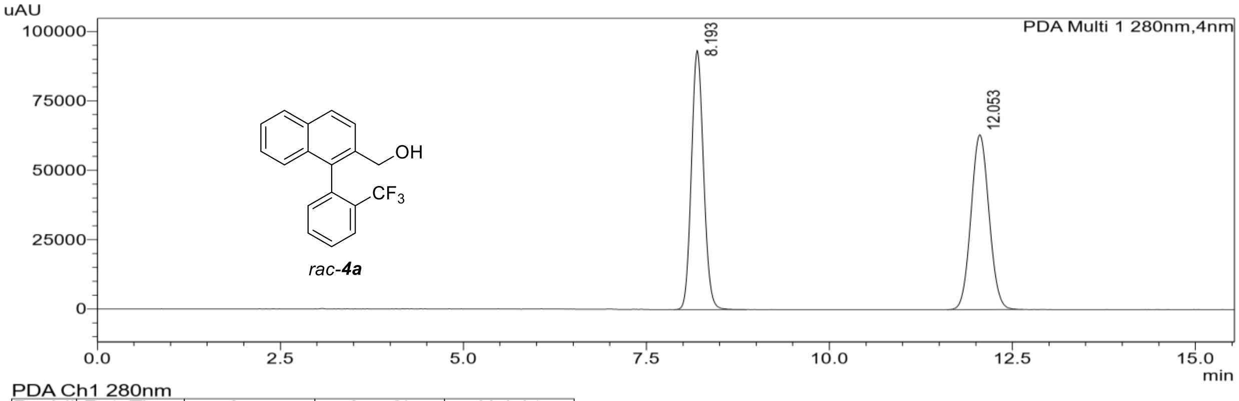

PDA Ch1 280nm

Peak#	Ret. Time	Area	Area%	Height
1	8.193	1077207	50.004	93240
2	12.053	1077029	49.996	62987
Total		2154235	100.000	156226

<Chromatogram>

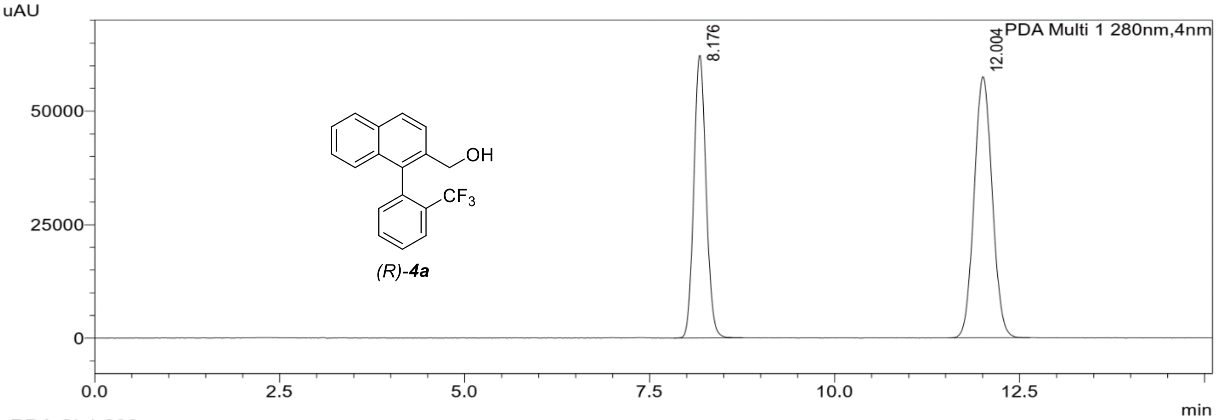

PDA Ch1 280nm

Peak#	Ret. Time	Area	Area%	Height
1	8.176	717012	42.265	62222
2	12.004	979448	57.735	57530
Total		1696460	100.000	119752

<Chromatogram>

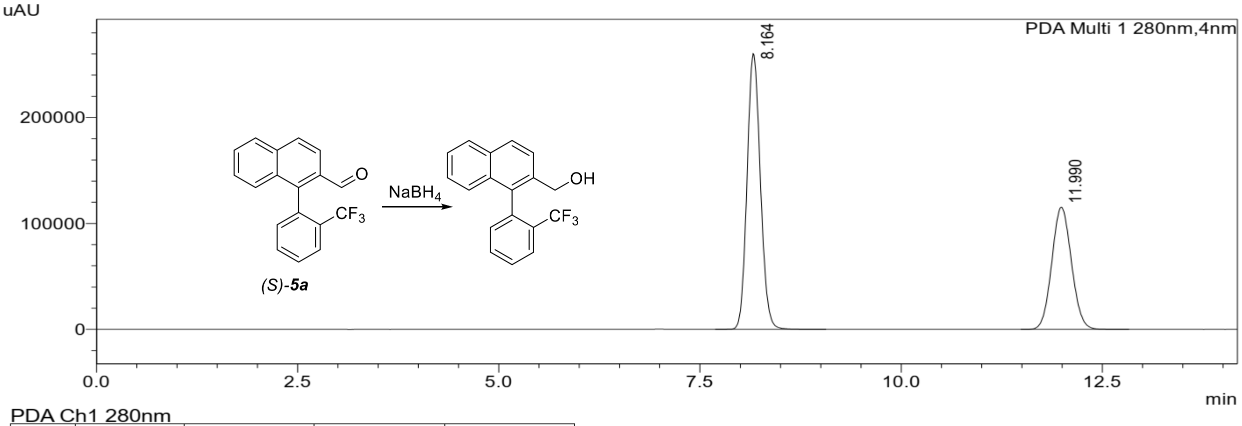

PDA Ch1 280nm

Peak#	Ret. Time	Area	Area%	Height
1	8.164	3009354	60.527	260125
2	11.990	1962570	39.473	115340
Total		4971925	100.000	375465

CuI

<Chromatogram>

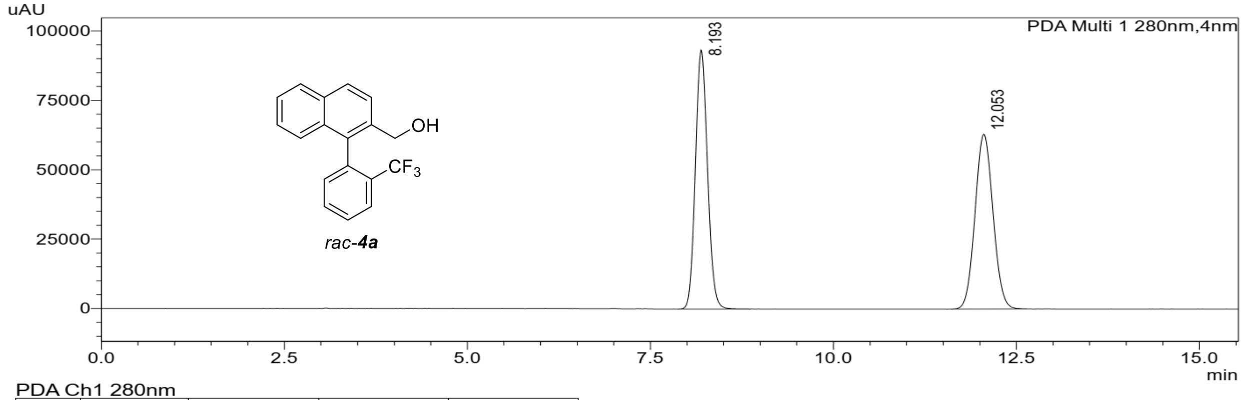

PDA Ch1 280nm

Peak#	Ret. Time	Area	Area%	Height
1	8.193	1077207	50.004	93240
2	12.053	1077029	49.996	62987
Total		2154235	100.000	156226

<Chromatogram>

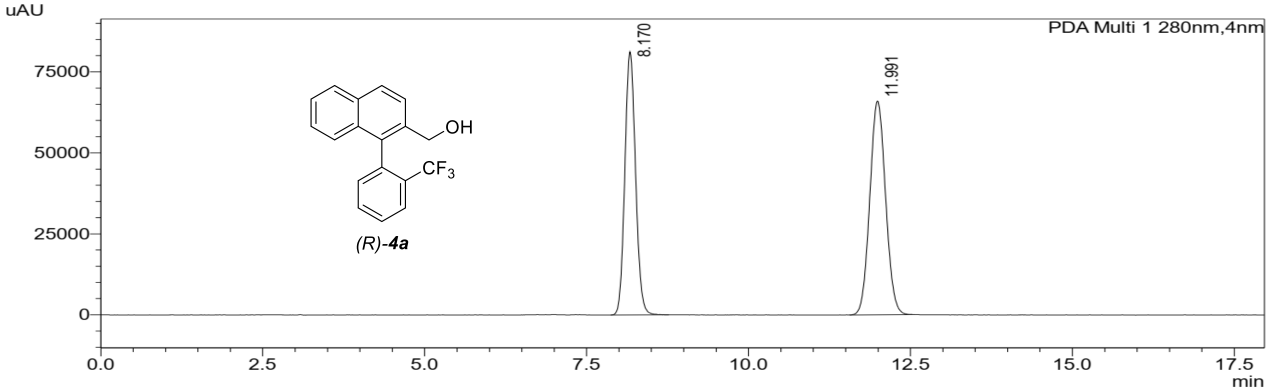

PDA Ch1 280nm

Peak#	Ret. Time	Area	Area%	Height
1	8.170	933923	45.466	81164
2	11.991	1120183	54.534	65954
Total		2054106	100.000	147118

<Chromatogram>

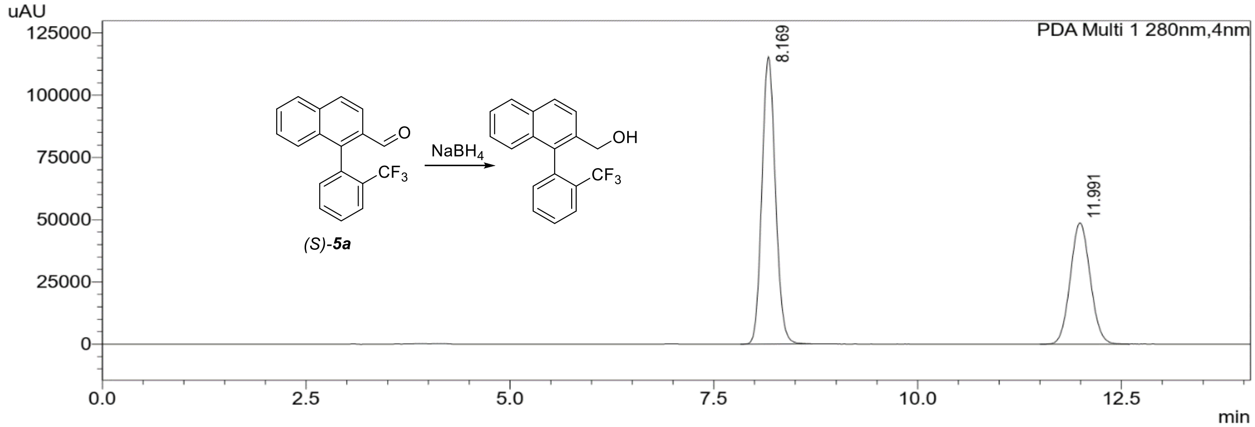

PDA Ch1 280nm

Peak#	Ret. Time	Area	Area%	Height
1	8.169	1332170	61.608	115413
2	11.991	830147	38.392	48720
Total		2162317	100.000	164133

6. Chiral HPLC traces of the products

*Chiral HPLC chromatograms of rac **2a**, recovered (R)-**2a** and aldehyde (S)-**3a***

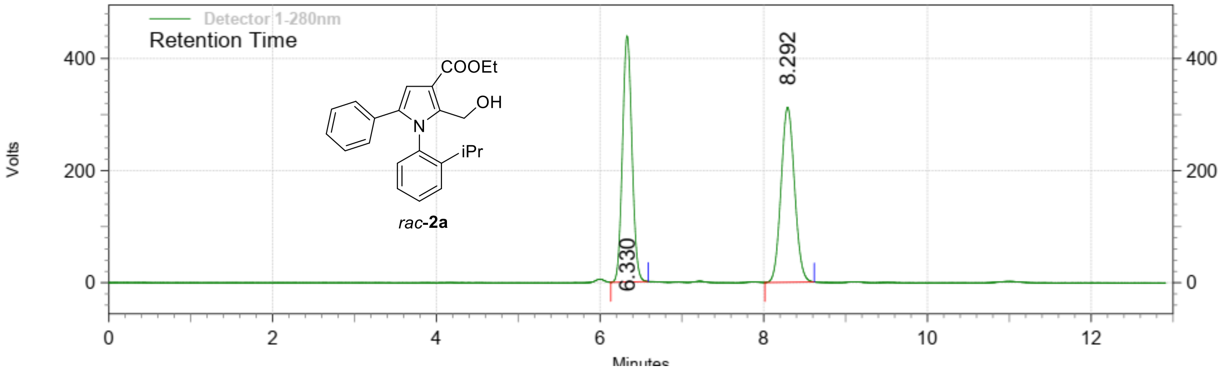

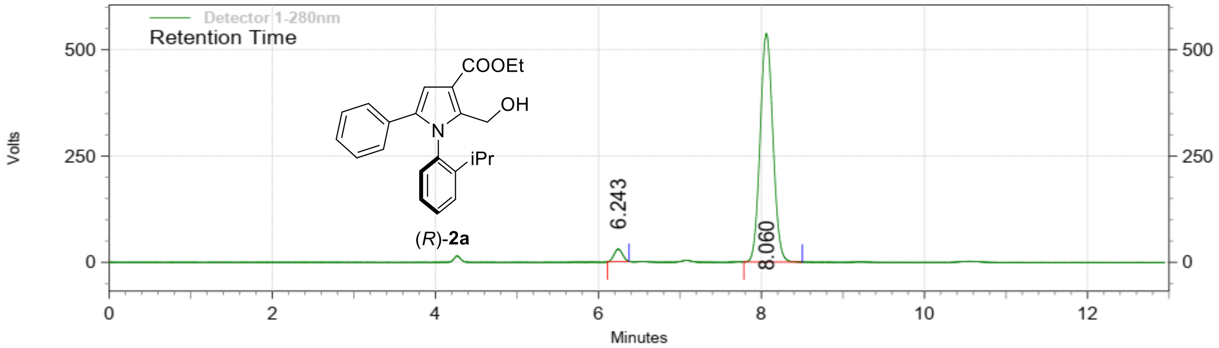

Retention Time	Area	Area %	Height	Height %
6.243	214862	3.54	29377	5.18
8.060	5855233	96.46	537394	94.82
Totals	6070095	100.00	566771	100.00

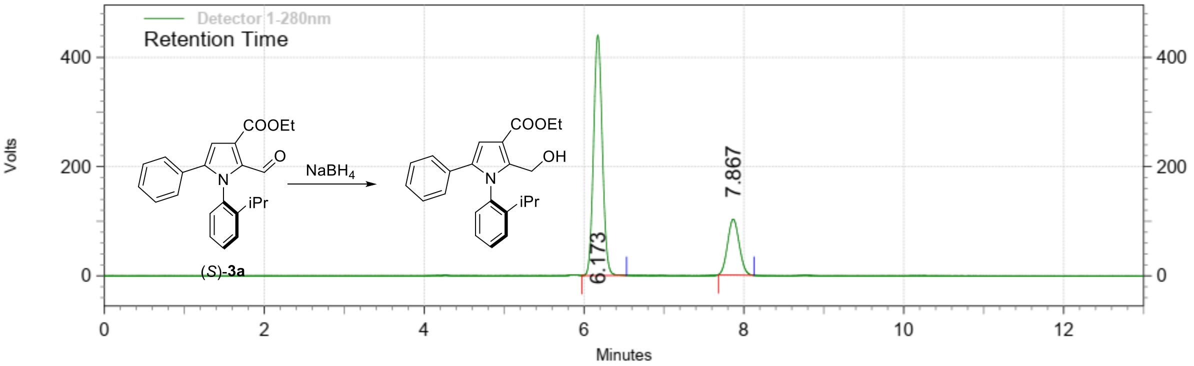

Retention Time	Area	Area %	Height	Height %
6.173	3343915	76.39	440365	81.17
7.867	1033788	23.61	102181	18.83
Totals	4377703	100.00	542546	100.00

*Chiral HPLC chromatograms of rac **2b**, recovered (R)-**2b** and aldehyde (S)-**3b***

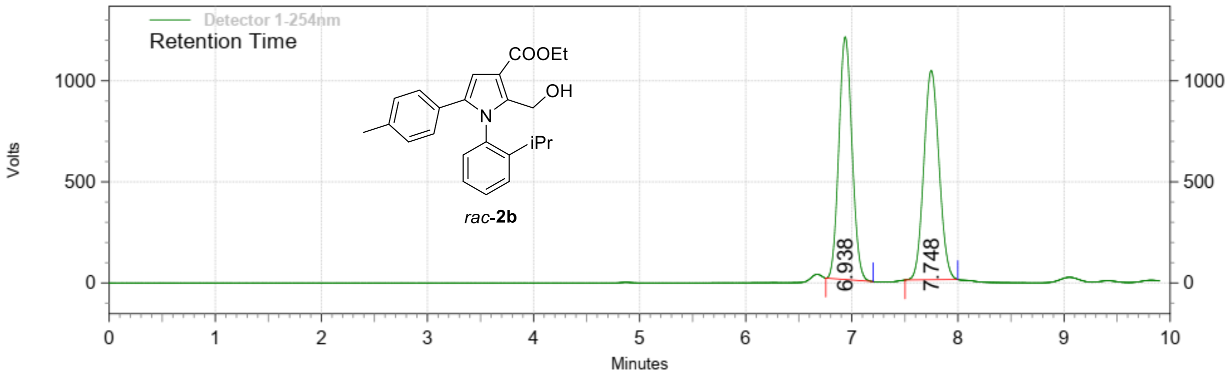

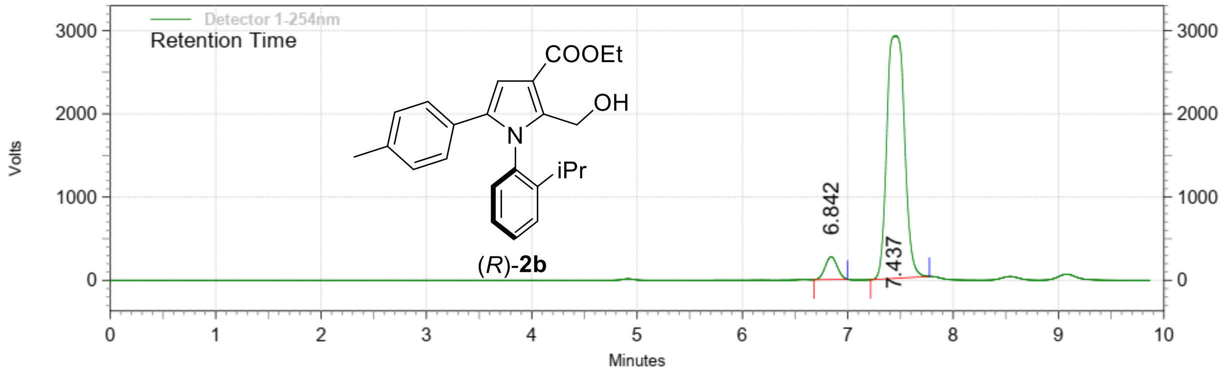

Retention Time	Area	Area %	Height	Height %
6.842	2178981	6.01	273625	8.59
7.437	34074166	93.99	2911175	91.41
Totals	36253147	100.00	3184800	100.00

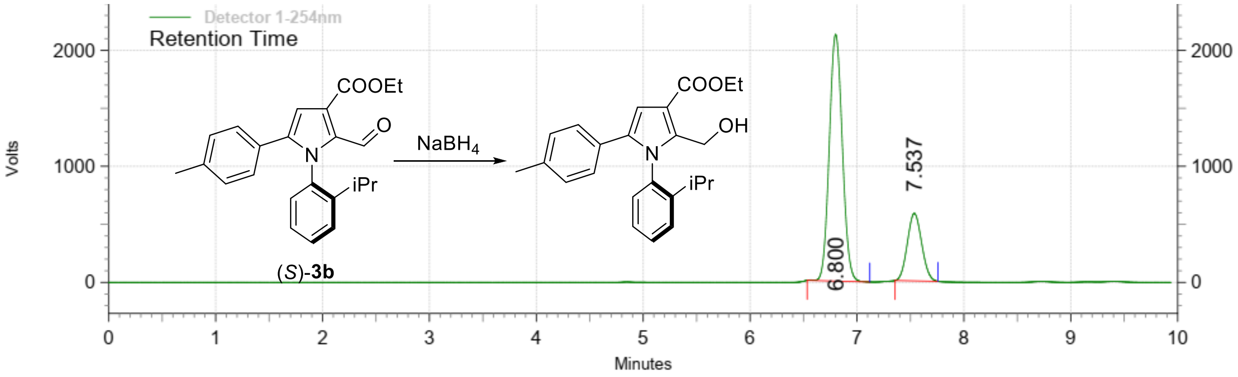

Retention Time	Area	Area %	Height	Height %
6.800	18025033	76.86	2125730	78.51
7.537	5426591	23.14	581837	21.49
Totals	23451624	100.00	2707567	100.00

*Chiral HPLC chromatograms of rac **2c**, recovered (R)-**2c** and aldehyde (S)-**3c***

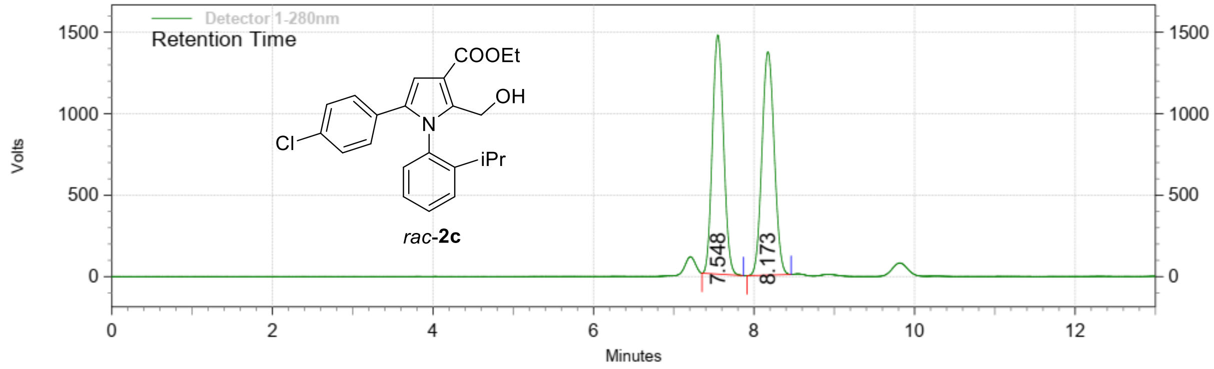

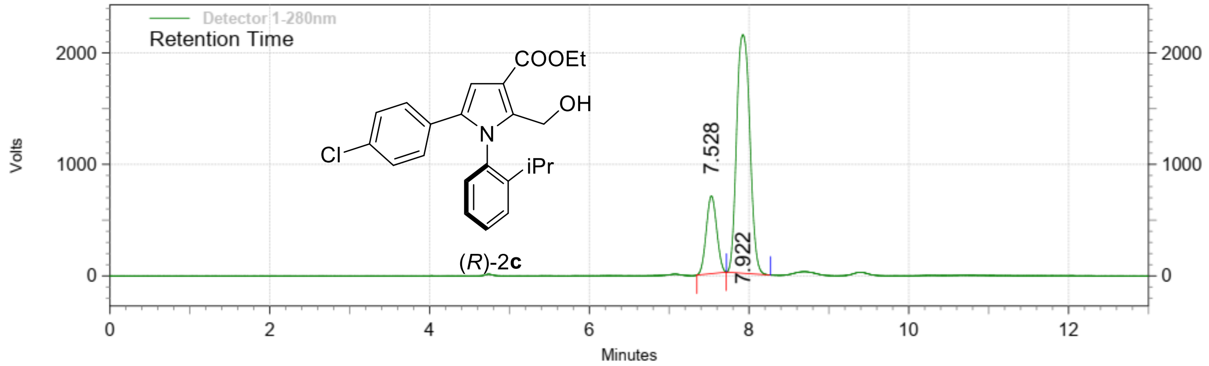

Retention Time	Area	Area %	Height	Height %
7.528	6344317	20.45	697050	24.59
7.922	24674161	79.55	2137952	75.41
Totals	31018478	100.00	2835002	100.00

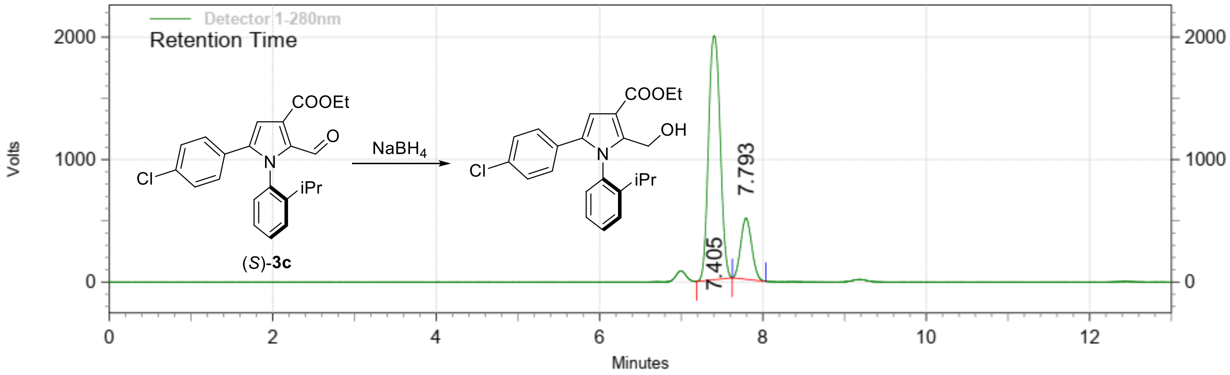

Retention Time	Area	Area %	Height	Height %
7.405	19776035	80.94	1991038	79.97
7.793	4656318	19.06	498545	20.03
Totals	24432353	100.00	2489583	100.00

*Chiral HPLC chromatograms of rac **2d**, recovered (R)-**2d** and aldehyde (S)-**3d***

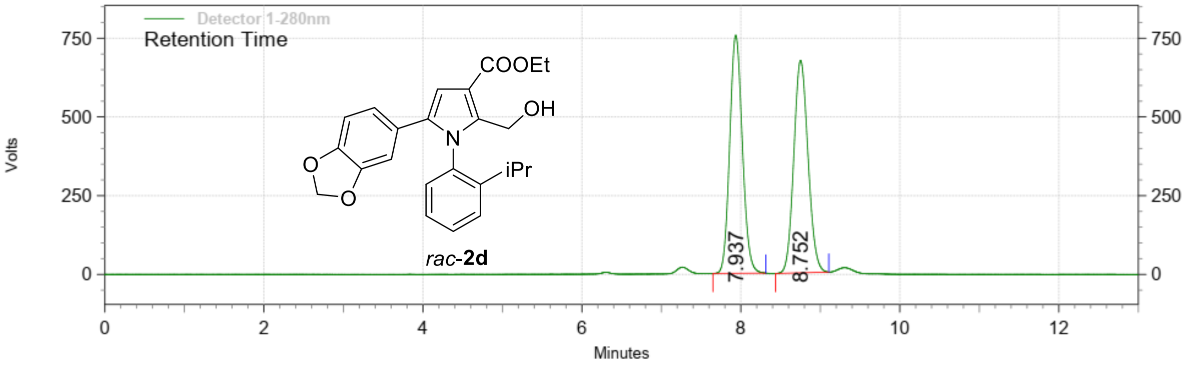

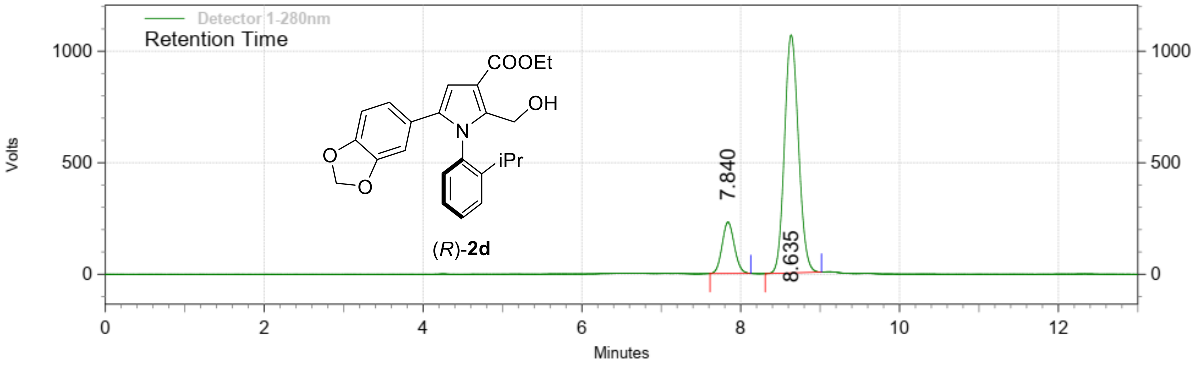

Retention Time	Area	Area %	Height	Height %
7.840	2467417	15.93	230492	17.77
8.635	13025670	84.07	1066734	82.23
Totals	15493087	100.00	1297226	100.00

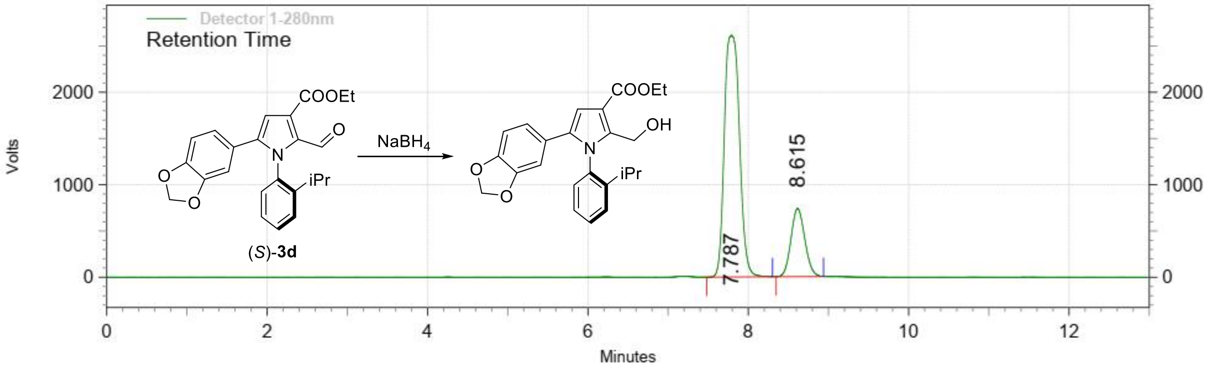

Retention Time	Area	Area %	Height	Height %
7.787	34332938	79.56	2611217	78.03
8.615	8820834	20.44	735108	21.97
Totals	43153772	100.00	3346325	100.00

*Chiral HPLC chromatograms of rac **2e**, recovered (R)-**2e** and aldehyde (S)-**3e***

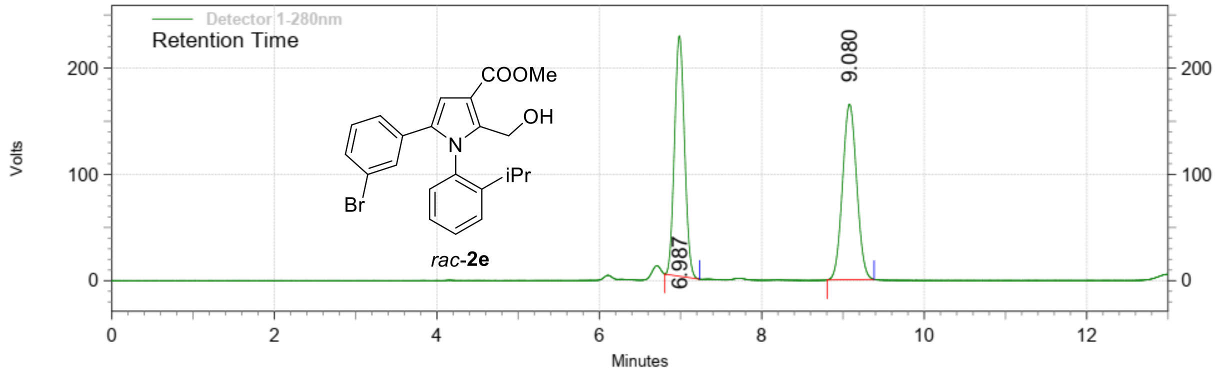

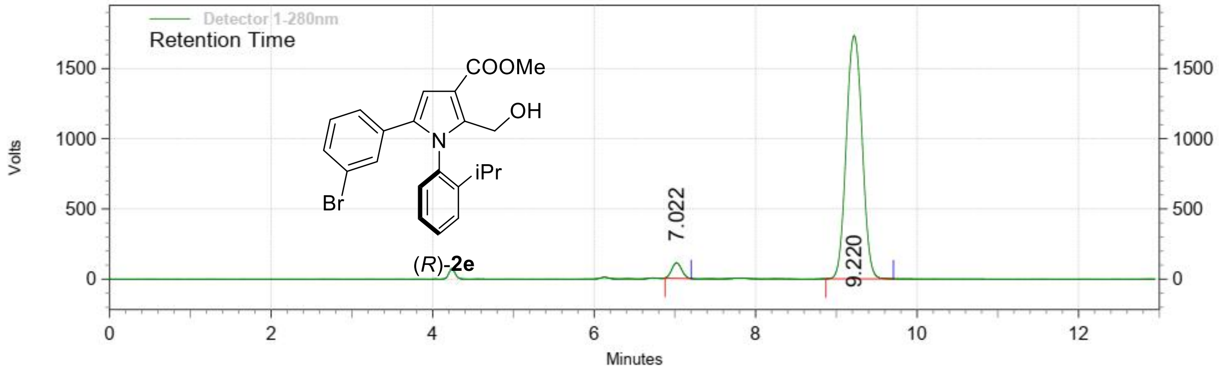

Retention Time	Area	Area %	Height	Height %
7.022	941388	3.83	110723	6.01
9.220	23661076	96.17	1731760	93.99
Totals	24602464	100.00	1842483	100.00

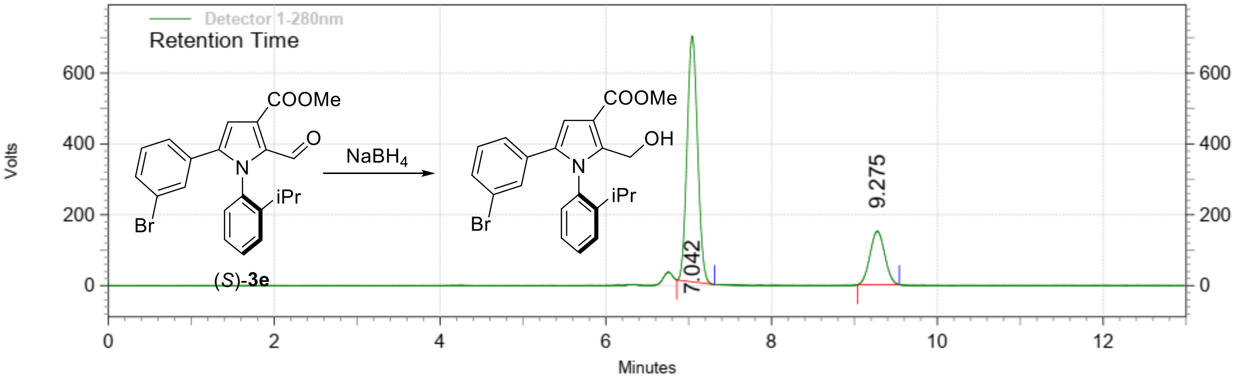

Retention Time	Area	Area %	Height	Height %
7.042	6135268	76.72	694005	82.15
9.275	1862179	23.28	150779	17.85
Totals	7997447	100.00	844784	100.00

*Chiral HPLC chromatograms of rac **2e**, recovered (R)-**2e** and aldehyde (S)-**3e (1 mmol Scale, under ambient air)***

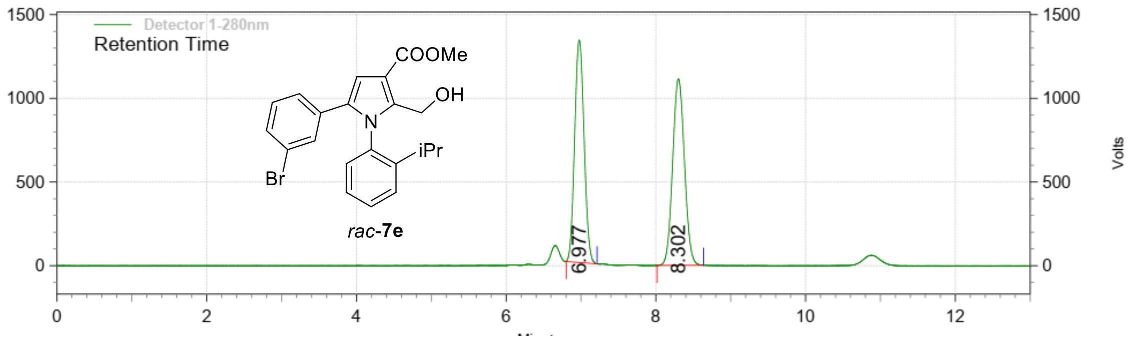

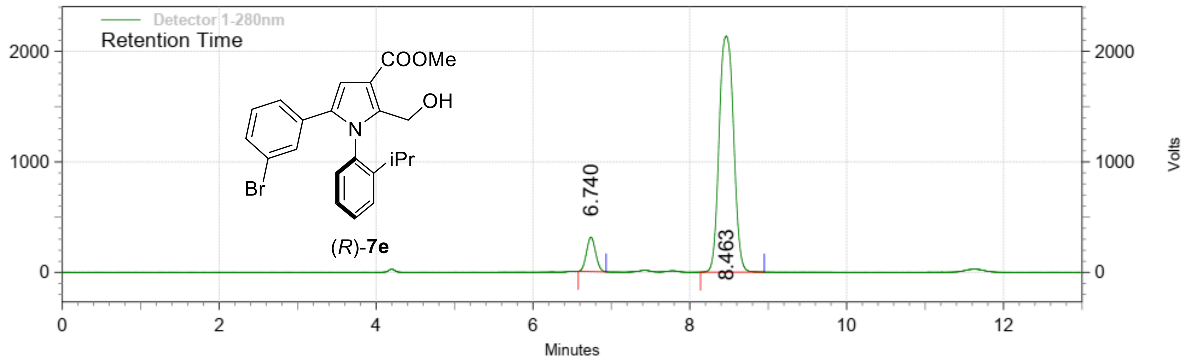

Retention Time	Area	Area %	Height	Height %
6.740	2520244	8.42	311832	12.73
8.463	27403740	91.58	2136843	87.27
Totals	29923984	100.00	2448675	100.00

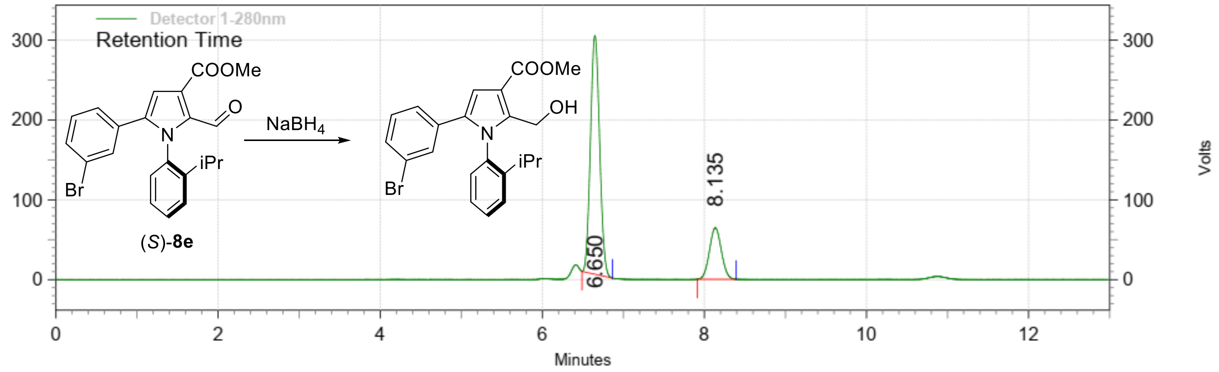

Retention Time	Area	Area %	Height	Height %
6.650	2343464	78.03	298856	82.27
8.135	659989	21.97	64409	17.73
Totals	3003453	100.00	363265	100.00

Chiral HPLC chromatograms of rac ***7e****, recovered (R)-****7e*** *and aldehyde (S)-****8e (1 mmol Scale, under pure oxygen)***

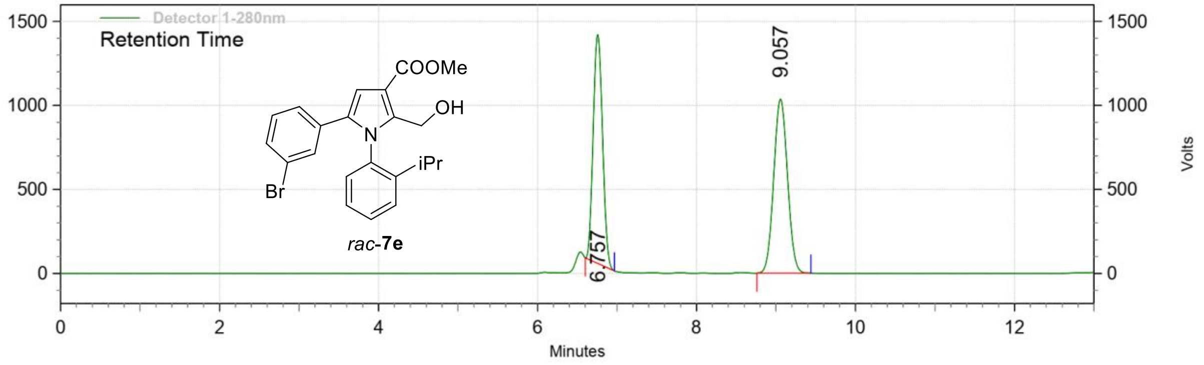

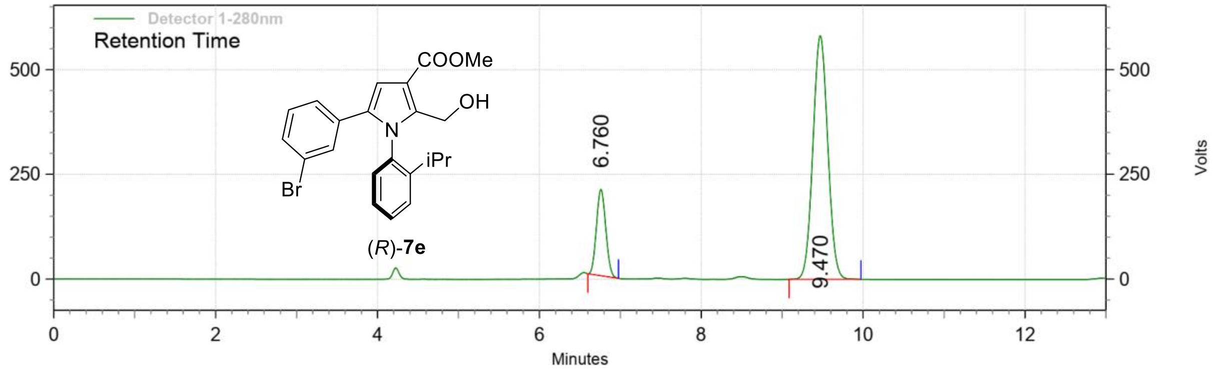

Retention Time	Area	Area %	Height	Height %
6.760	1653875	18.22	205848	26.14
9.470	7423102	81.78	581699	73.86
Totals	9076977	100.00	787547	100.00

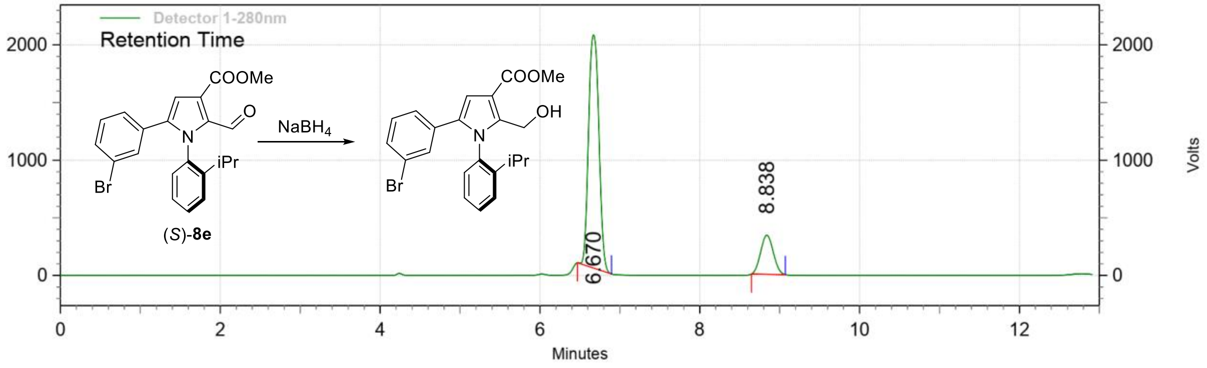

Retention Time	Area	Area %	Height	Height %
6.670	18190371	82.98	2023785	85.63
8.838	3729801	17.02	339489	14.37
Totals	21920172	100.00	2363274	100.00

*HPLC chromatograms of rac **2f**, recovered (R)-**2f** and aldehyde (S)-**3f***

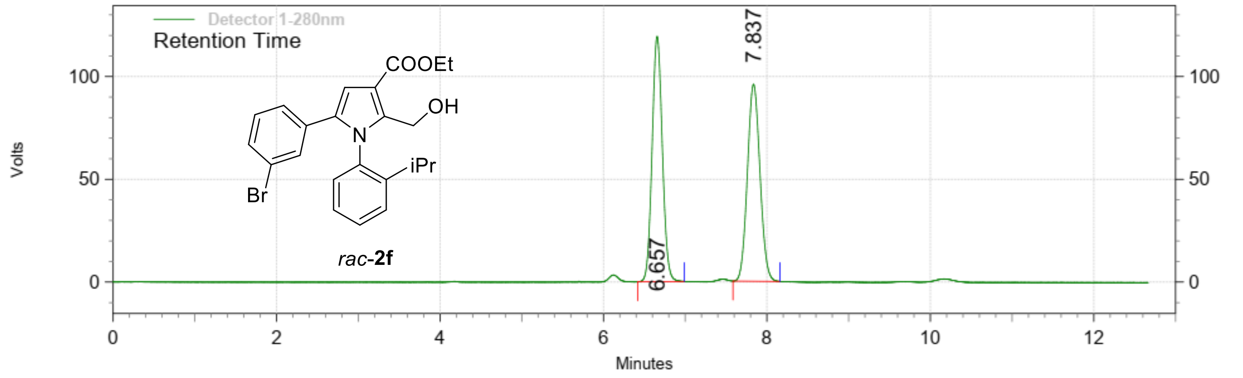

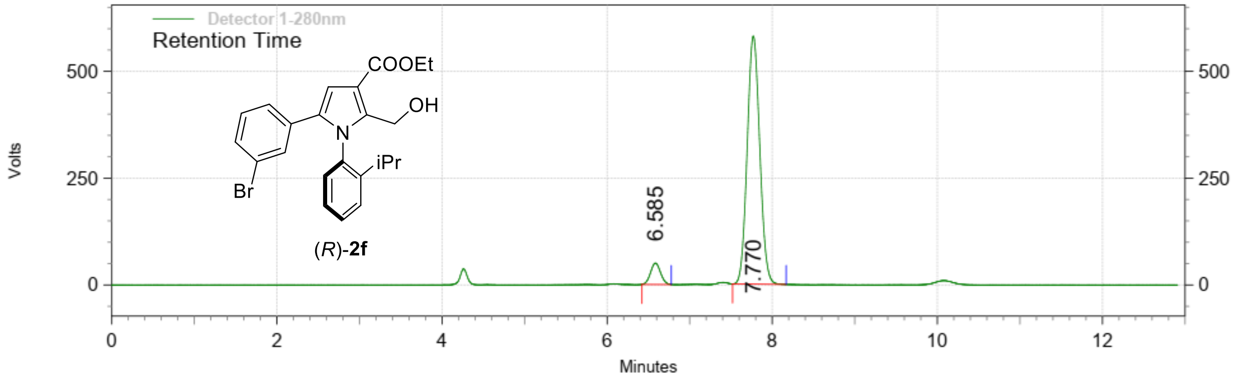

Retention Time	Area	Area %	Height	Height %
6.585	421016	6.41	50063	7.94
7.770	6142919	93.59	580782	92.06
Totals	6563935	100.00	630845	100.00

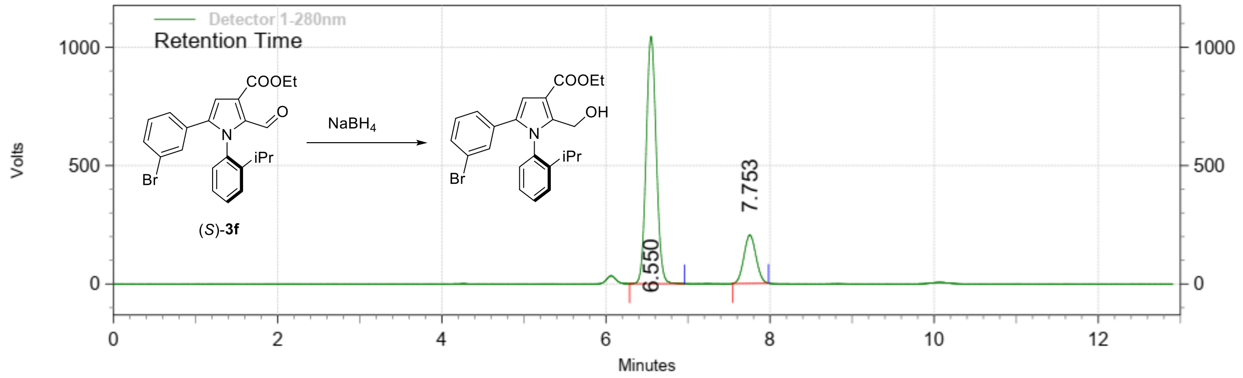

Retention Time	Area	Area %	Height	Height %
6.550	8882786	80.92	1044681	83.64
7.753	2094879	19.08	204398	16.36
Totals	10977665	100.00	1249079	100.00

*Chiral HPLC chromatograms of rac **2g**, recovered (R)-**2g** and aldehyde (S)-**3g***

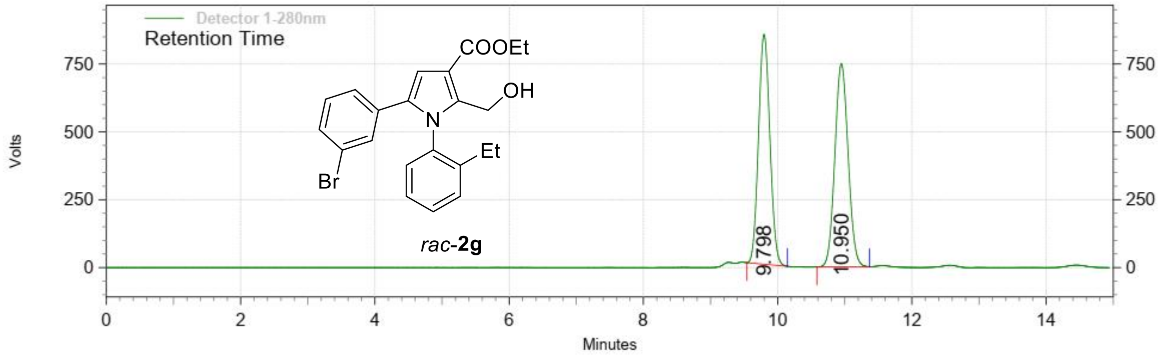

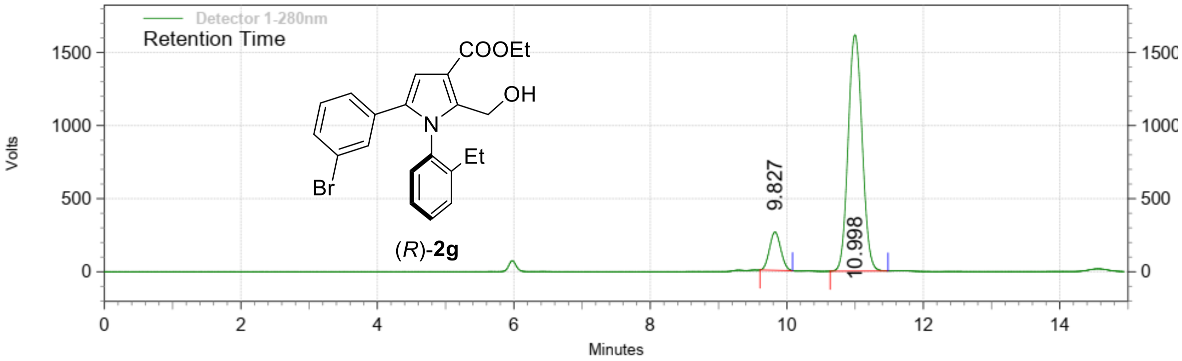

Retention Time	Area	Area %	Height	Height %
9.827	3012388	11.53	261972	13.94
10.998	23111198	88.47	1617299	86.06
Totals	26123586	100.00	1879271	100.00

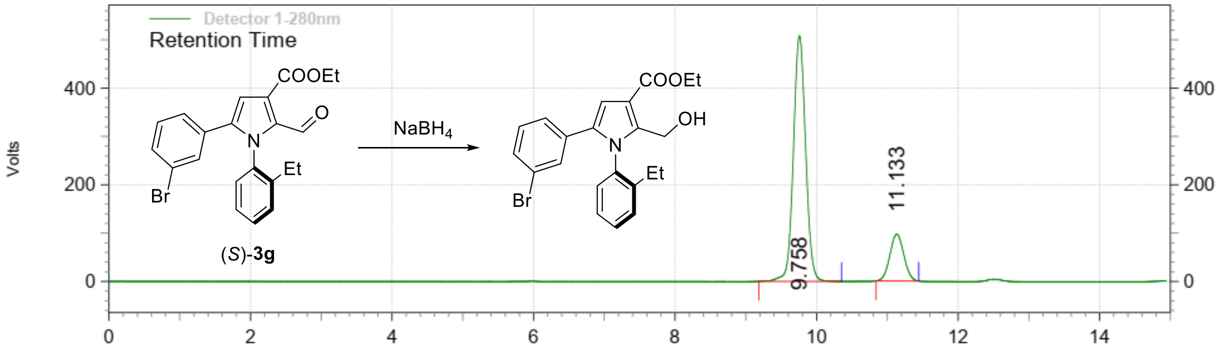

Retention Time	Area	Area %	Height	Height %
9.758	6117187	82.16	508045	83.97
11.133	1327978	17.84	96984	16.03
Totals	7445165	100.00	605029	100.00

*Chiral HPLC chromatograms of rac **2h**, recovered (R)-**2h** and aldehyde (S)-**2h***

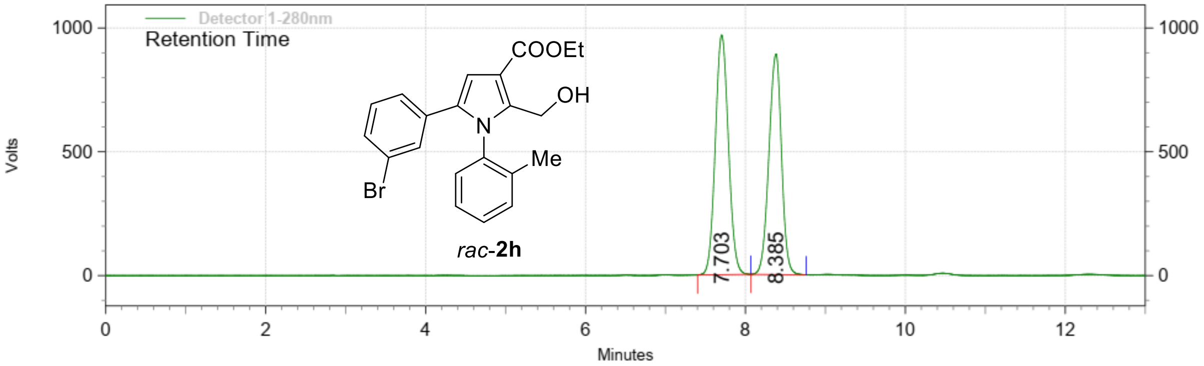

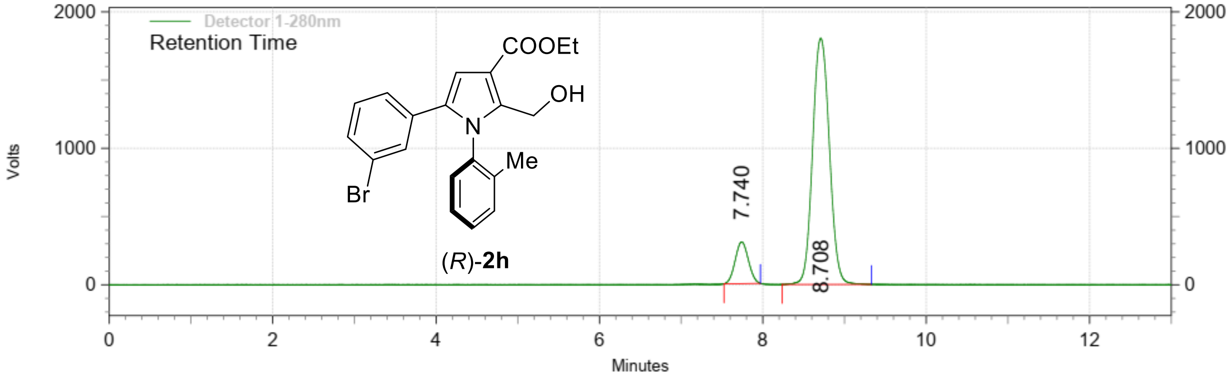

Retention Time	Area	Area %	Height	Height %
7.740	3434194	11.77	305975	14.50
8.708	25743507	88.23	1803930	85.50
Totals	29177701	100.00	2109905	100.00

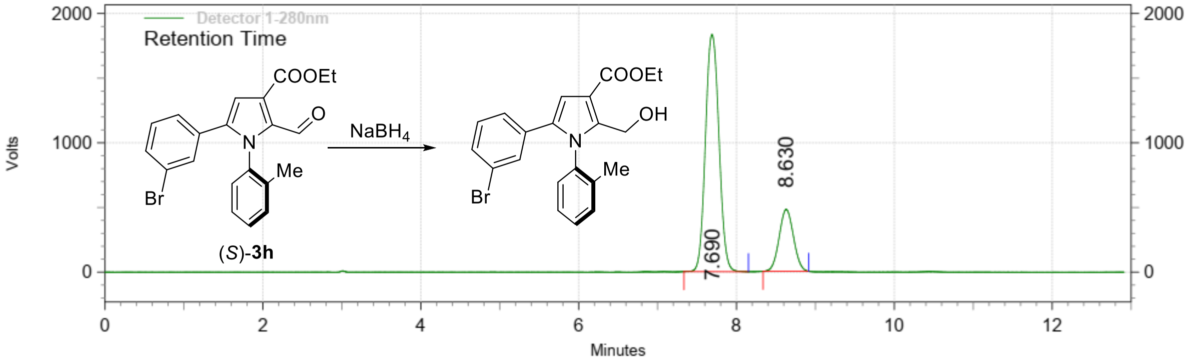

Retention Time	Area	Area %	Height	Height %
7.690	21822781	78.31	1834299	79.30
8.630	6042821	21.69	478897	20.70
Totals	27865602	100.00	2313196	100.00

*Chiral HPLC chromatograms of rac **2i**, recovered (R)-**2i** and aldehyde (S)-**3i***

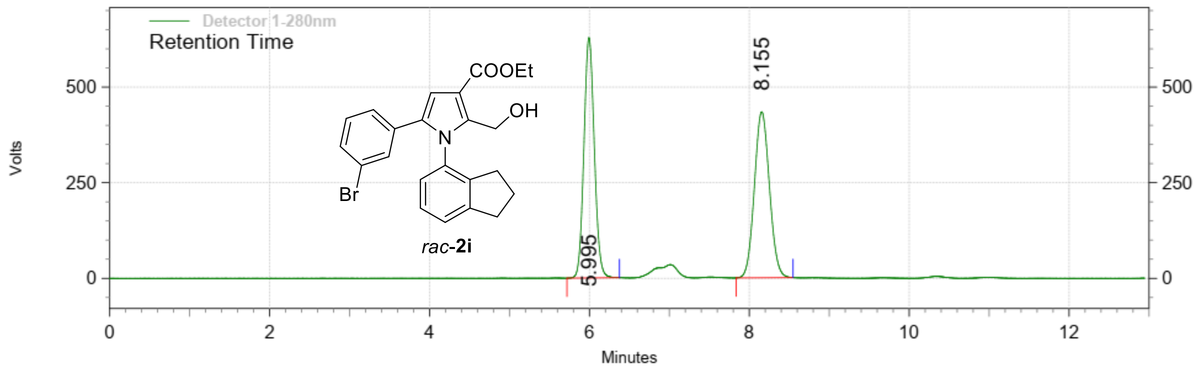

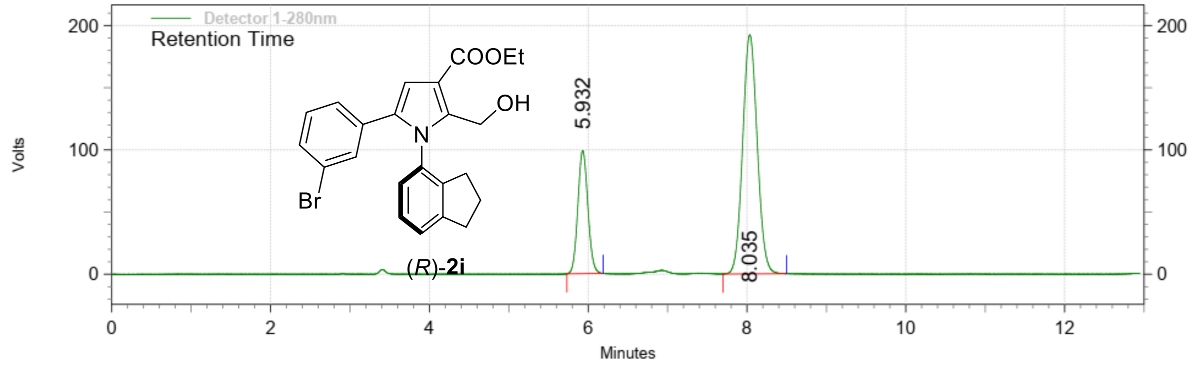

Retention Time	Area	Area %	Height	Height %
5.932	867791	26.14	99065	34.01
8.035	2452040	73.86	192241	65.99
Totals	3319831	100.00	291306	100.00

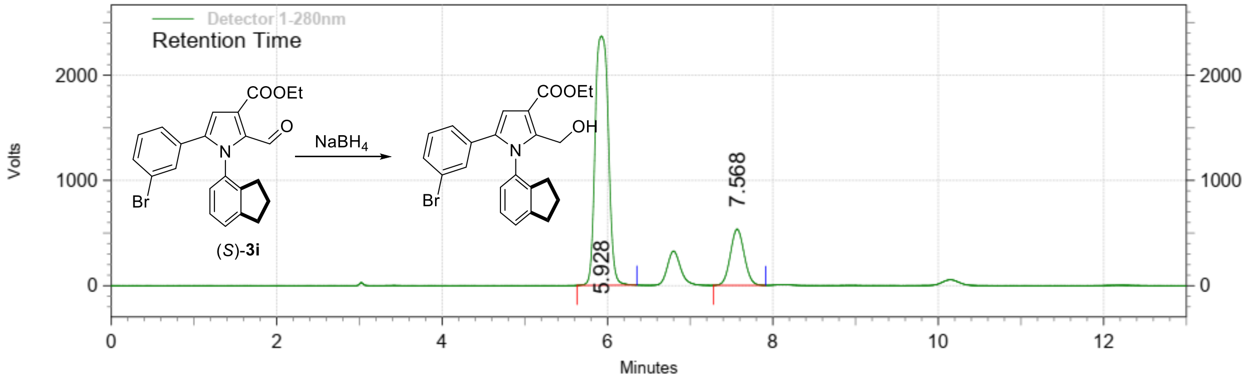

Retention Time	Area	Area %	Height	Height %
5.928	26432411	80.94	2367763	81.61
7.568	6222801	19.06	533518	18.39
Totals	32655212	100.00	2901281	100.00

*Chiral HPLC chromatograms of rac **2j**, recovered (R)-**2j** and aldehyde (S)-**3j***

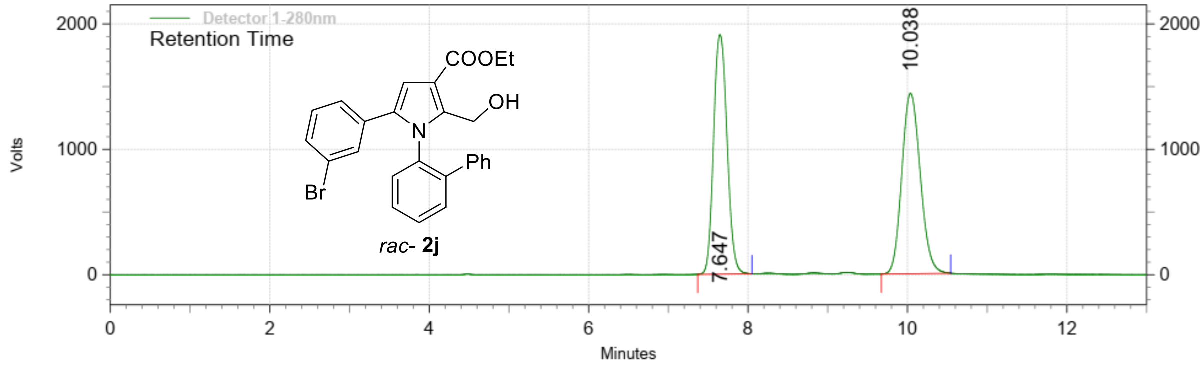

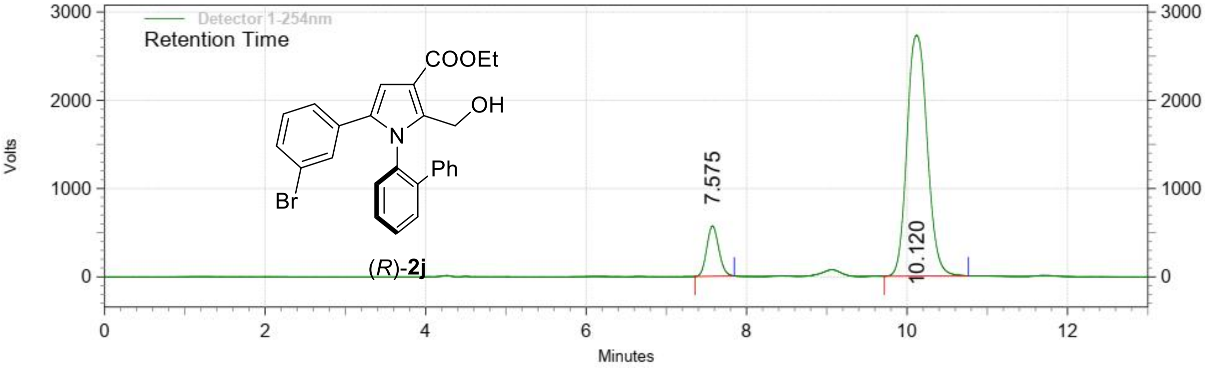

Retention Time	Area	Area %	Height	Height %
7.575	5937501	11.04	569662	17.27
10.120	47844574	88.96	2728187	82.73
Totals	53782075	100.00	3297849	100.00

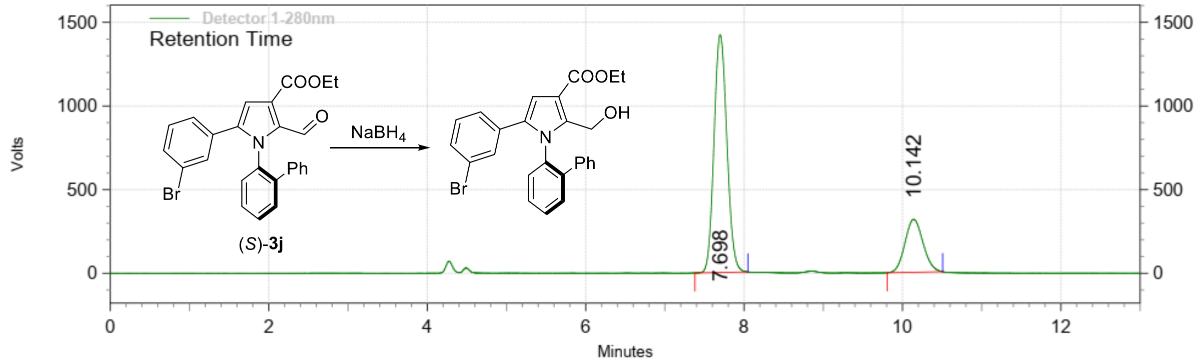

Retention Time	Area	Area %	Height	Height %
7.698	16075414	76.19	1420477	81.78
10.142	5023892	23.81	316436	18.22
Totals	21099306	100.00	1736913	100.00

*Chiral HPLC chromatograms of rac **2k**, recovered (R)-2**k** and aldehyde (S)-**3k***

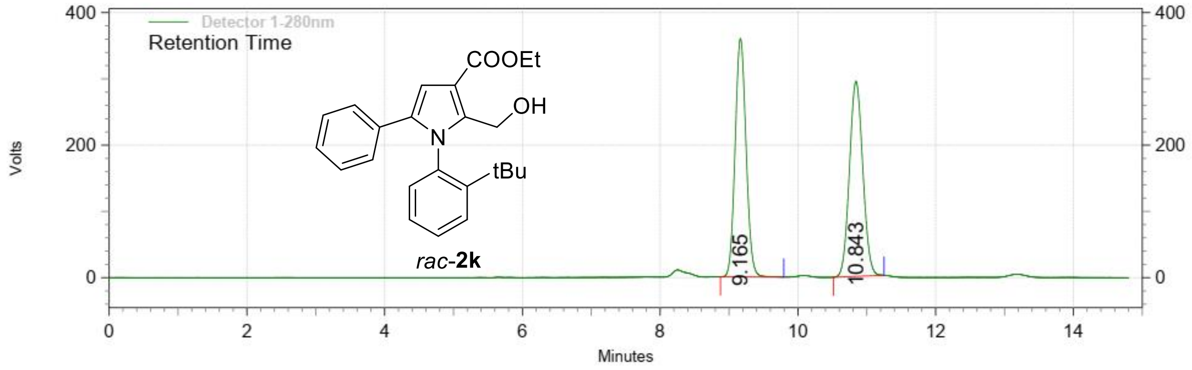

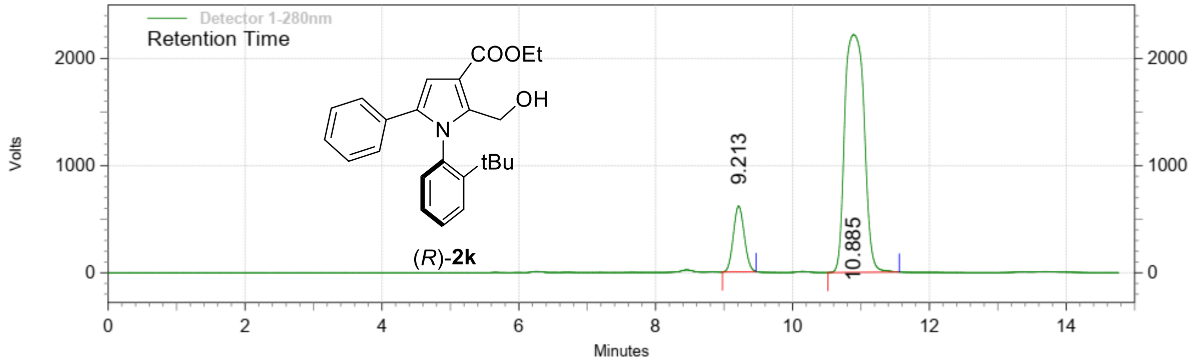

Retention Time	Area	Area %	Height	Height %
9.213	6801155	13.41	614023	21.68
10.885	43924648	86.59	2218093	78.32
Totals	50725803	100.00	2832116	100.00

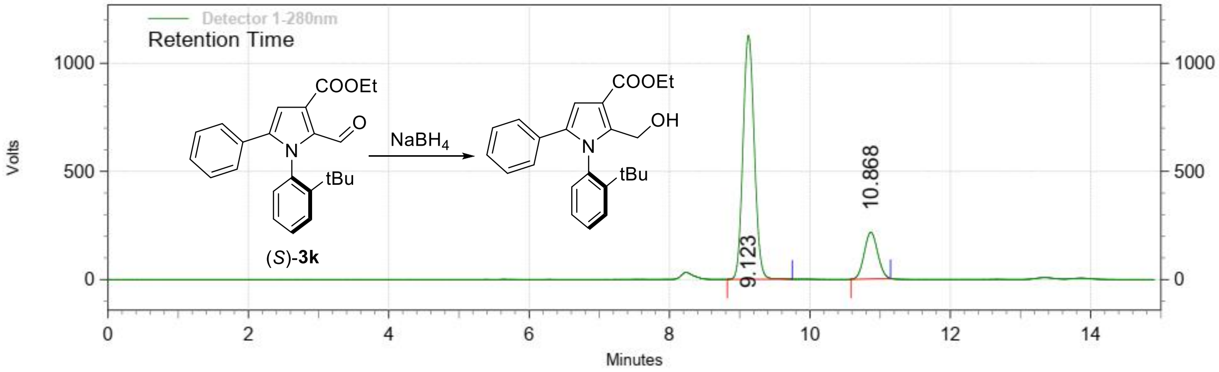

Retention Time	Area	Area %	Height	Height %
9.123	12627433	81.55	1127750	83.96
10.868	2856798	18.45	215467	16.04
Totals	15484231	100.00	1343217	100.00

Chiral HPLC *chromatograms* of *rac* **2l**, *recovered (R)-**2l** and aldehyde (S)-**3l***

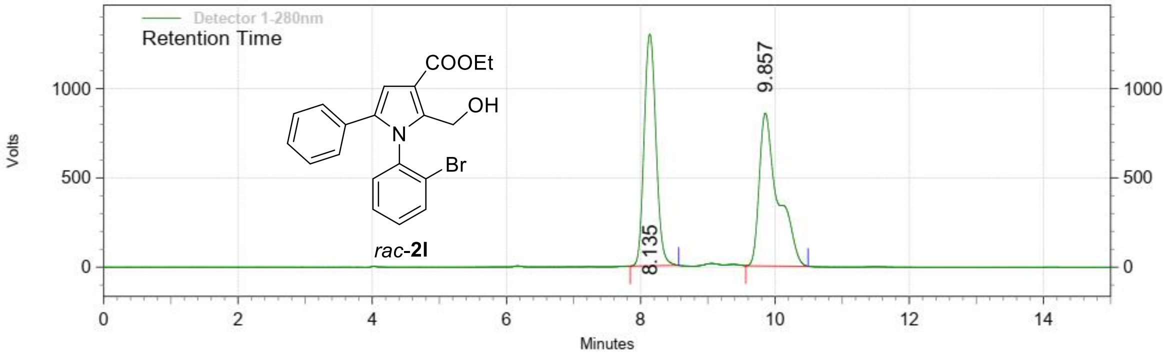

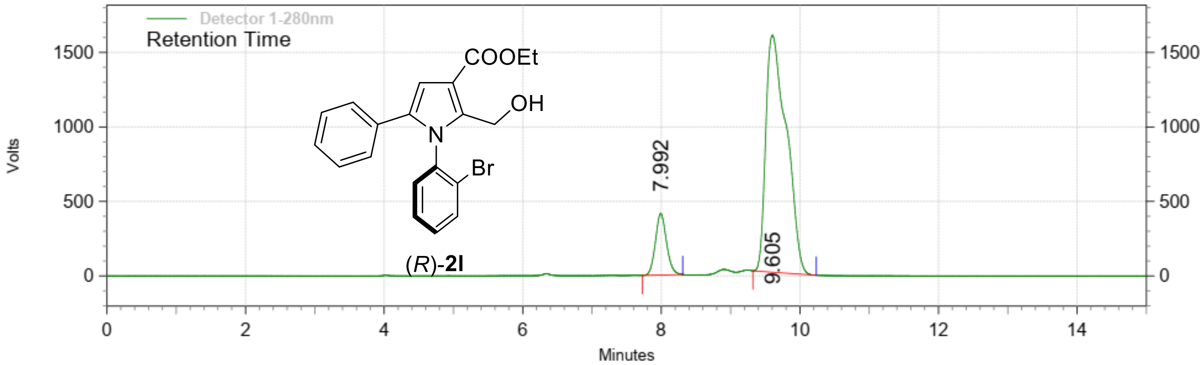

Retention Time	Area	Area %	Height	Height %
7.992	4596348	12.23	413006	20.61
9.605	32977849	87.77	1590604	79.39

Totals	37574197	100.00	2003610	100.00

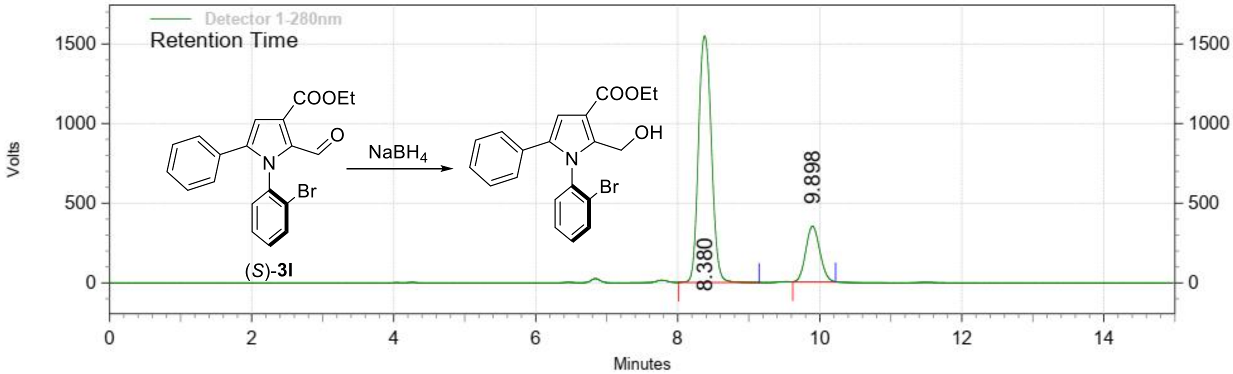

[illegible]

Retention Time	Area	Area %	Height	Height %
8.380	19555529	80.37	1547691	81.61
9.898	4776227	19.63	348779	18.39

Totals	24331756	100.00	1896470	100.00

*Chiral HPLC chromatograms of rac **2m**, recovered (R)-**2m** and aldehyde (S)-**3m***

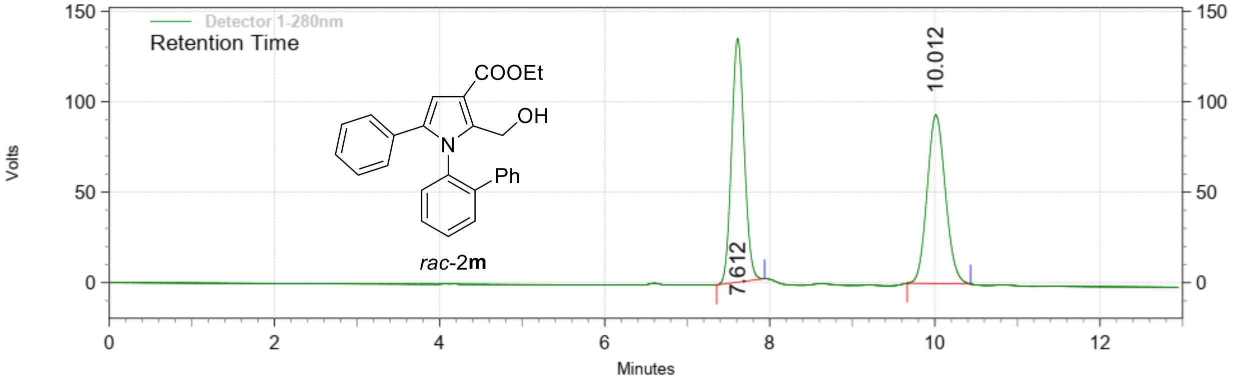

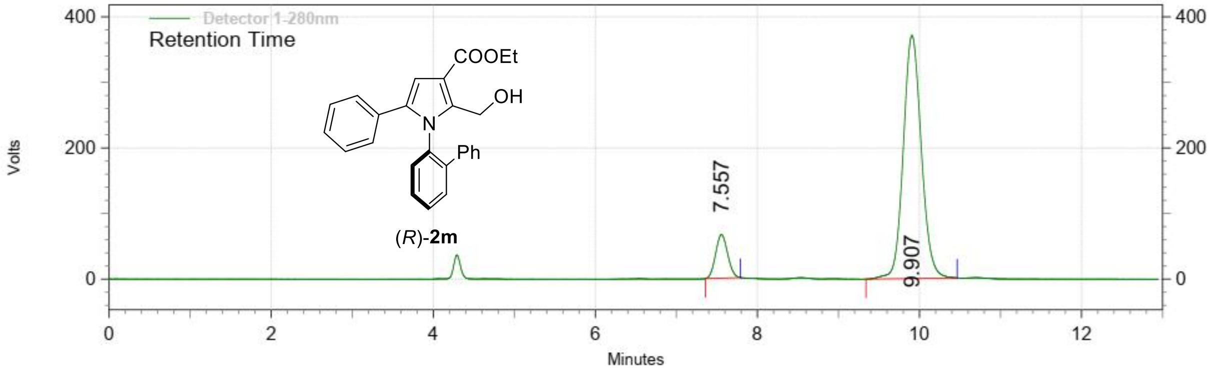

Retention Time	Area	Area %	Height	Height %
7.557	683798	10.67	66361	15.18
9.907	5724427	89.33	370812	84.82
Totals	6408225	100.00	437173	100.00

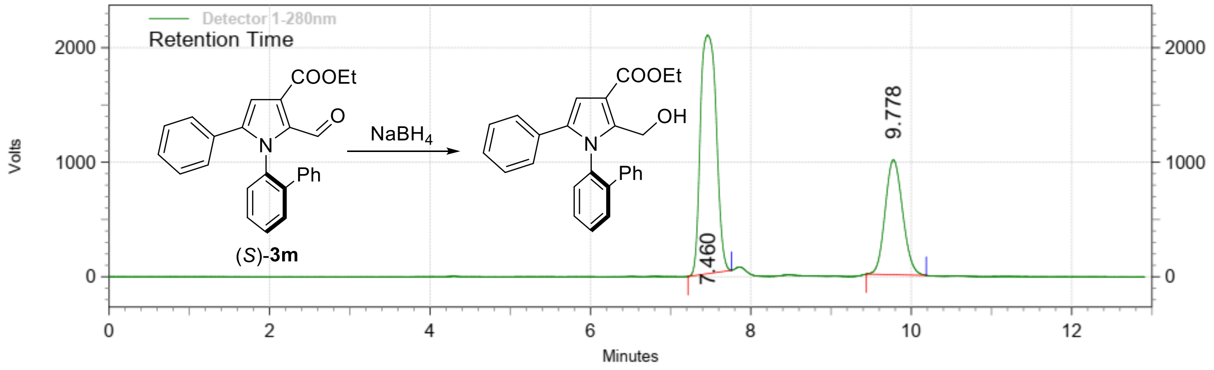

Retention Time	Area	Area %	Height	Height %
7.460	28753204	65.41	2081821	67.50
9.778	15204859	34.59	1002412	32.50
Totals	43958063	100.00	3084233	100.00

*Chiral HPLC chromatograms of rac **2n**, recovered (R)-**2n** and aldehyde (S)-**3n***

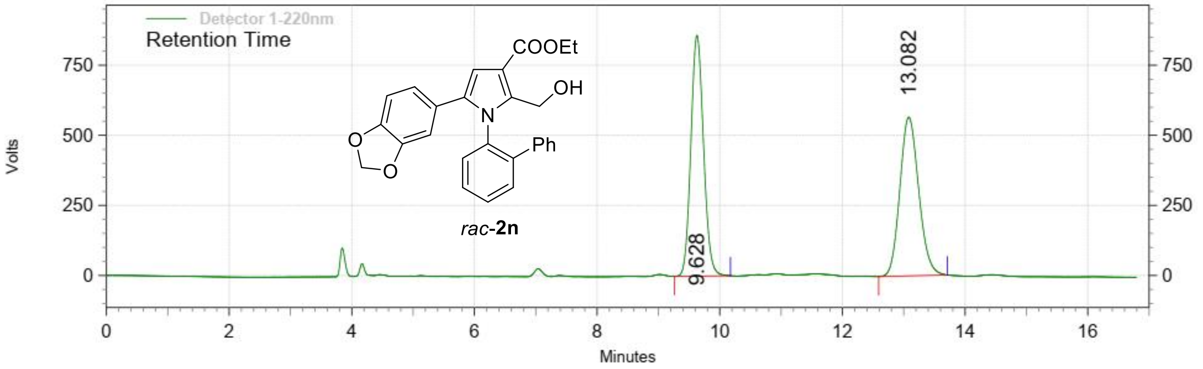

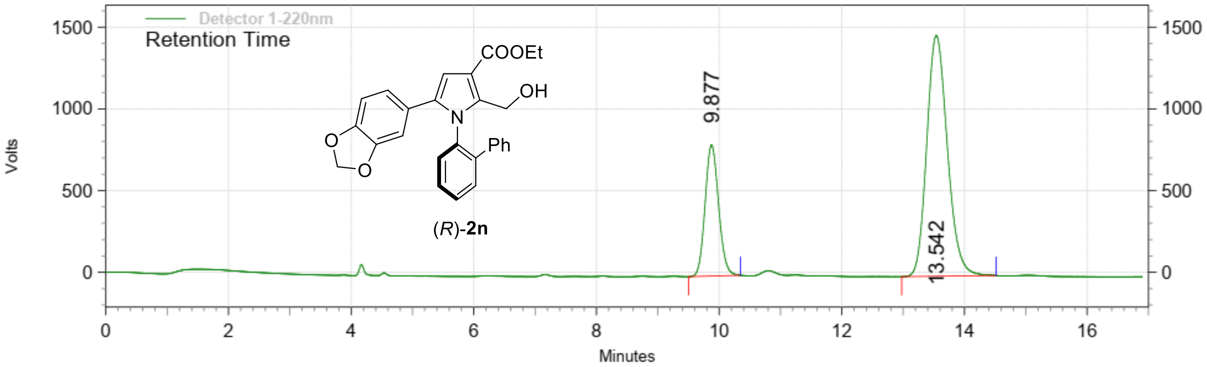

Retention Time	Area	Area %	Height	Height %
9.877	12259734	26.24	802440	35.25
13.542	34462412	73.76	1473684	64.75
Totals	46722146	100.00	2276124	100.00

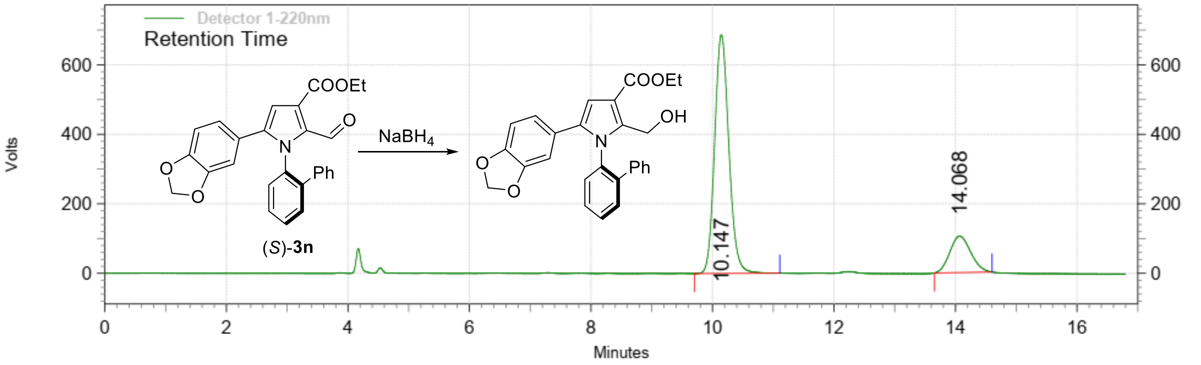

Retention Time	Area	Area %	Height	Height %
10.147	11191164	81.68	686856	86.76
14.068	2510059	18.32	104850	13.24
Totals	13701223	100.00	791706	100.00

*Chiral HPLC chromatograms of rac **2o**, recovered (R)-**2o** and aldehyde (S)-**3o***

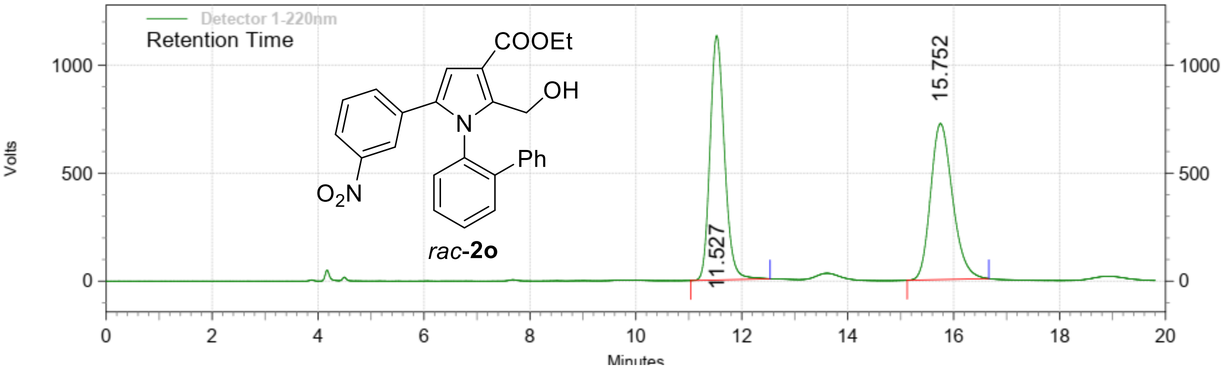

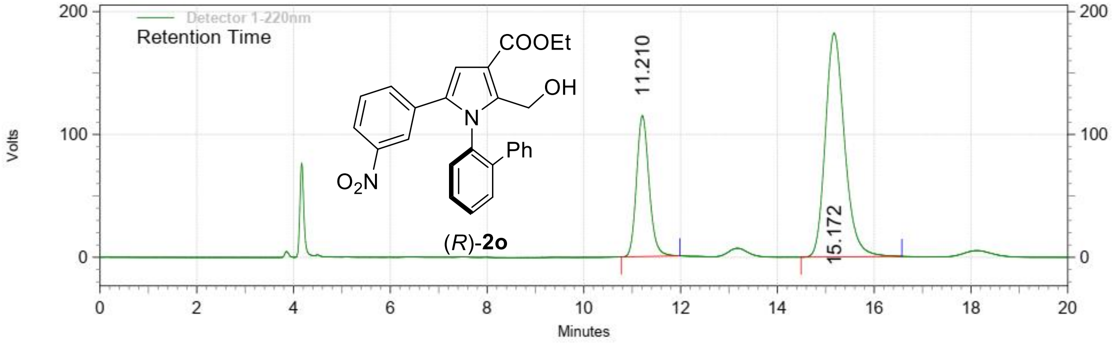

Retention Time	Area	Area %	Height	Height %
11.210	2066738	29.13	114641	38.63
15.172	5028582	70.87	182113	61.37

Totals				
	7095320	100.00	296754	100.00

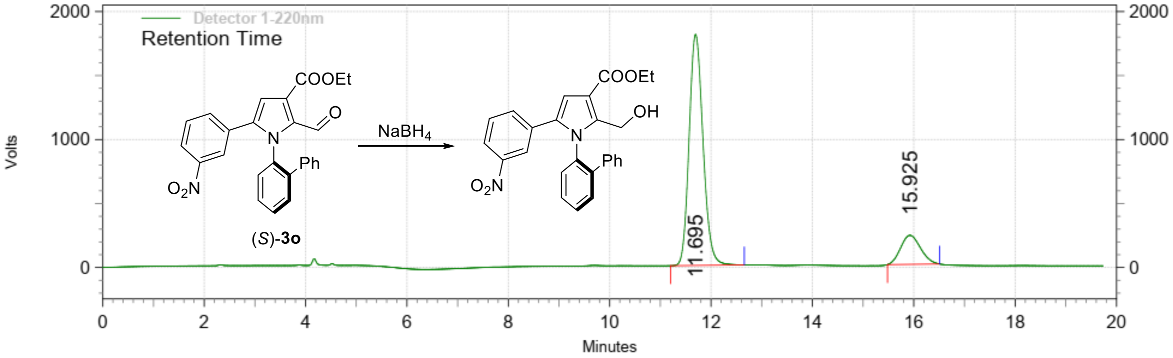

Retention Time	Area	Area %	Height	Height %
11.695	34493509	84.77	1805051	88.84
15.925	6195345	15.23	226833	11.16

Totals				
	40688854	100.00	2031884	100.00

*Chiral HPLC chromatograms of rac **2p**, recovered (R)-2**p** and aldehyde (S)-**3p***

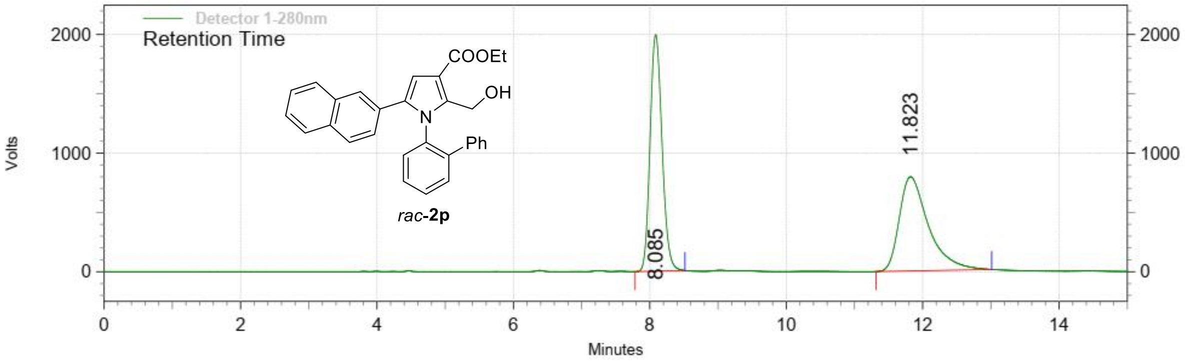

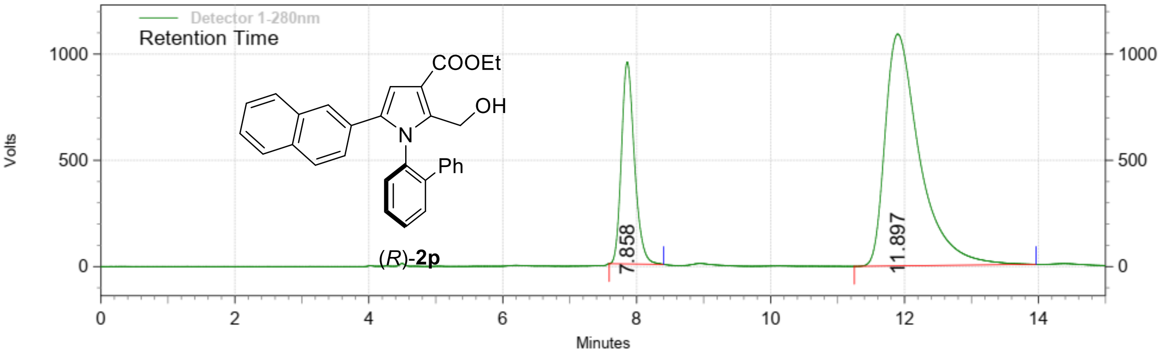

Retention Time	Area	Area %	Height	Height %
7.858	12816029	24.67	950758	46.56
11.897	39137145	75.33	1091391	53.44
Totals	51953174	100.00	2042149	100.00

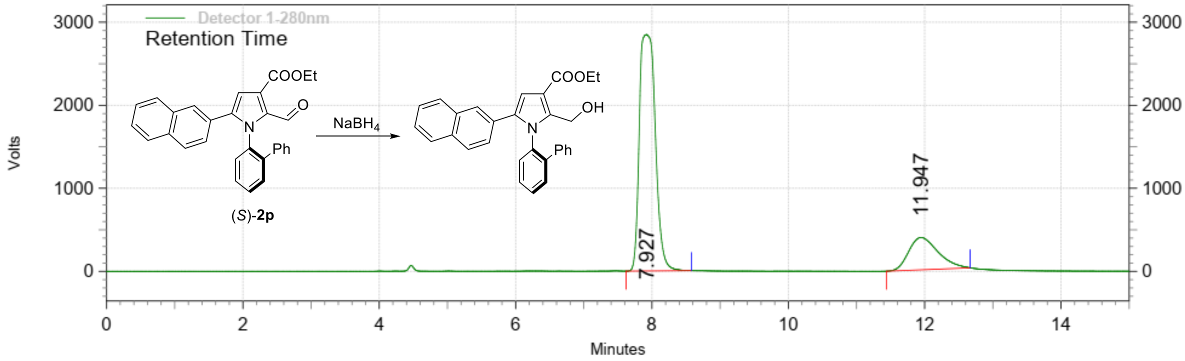

Retention Time	Area	Area %	Height	Height %
7.927	46506987	79.95	2848753	88.00
11.947	11664217	20.05	388469	12.00
Totals	58171204	100.00	3237222	100.00

*Chiral HPLC chromatograms of rac **2q**, recovered (R)-**2q** and aldehyde (S)-**3q***

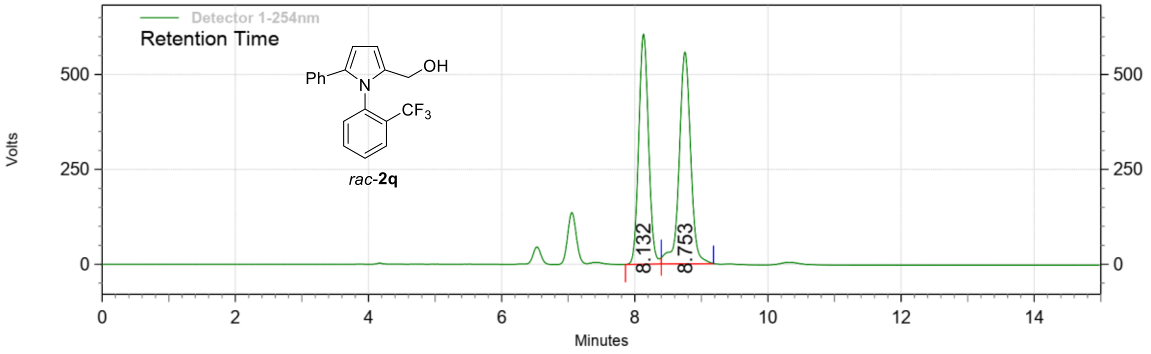

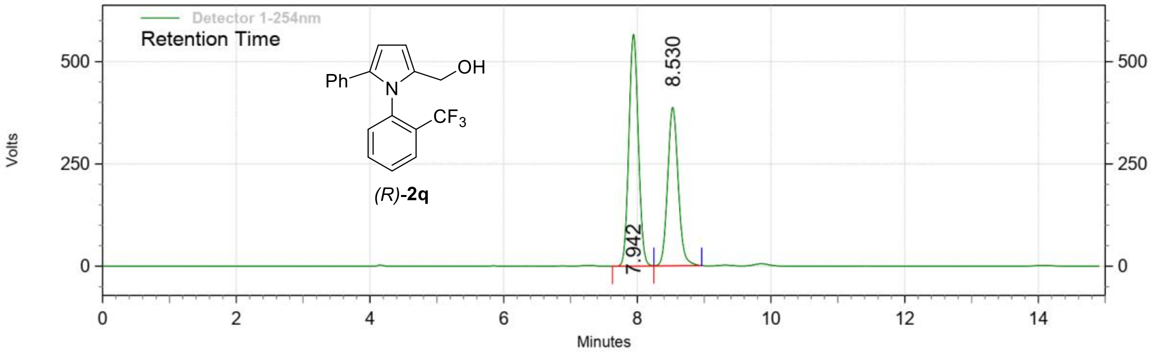

Retention Time	Area	Area %	Height	Height %
7.942	5552424	56.61	566339	59.40
8.530	4255847	43.39	387134	40.60
Totals	9808271	100.00	953473	100.00

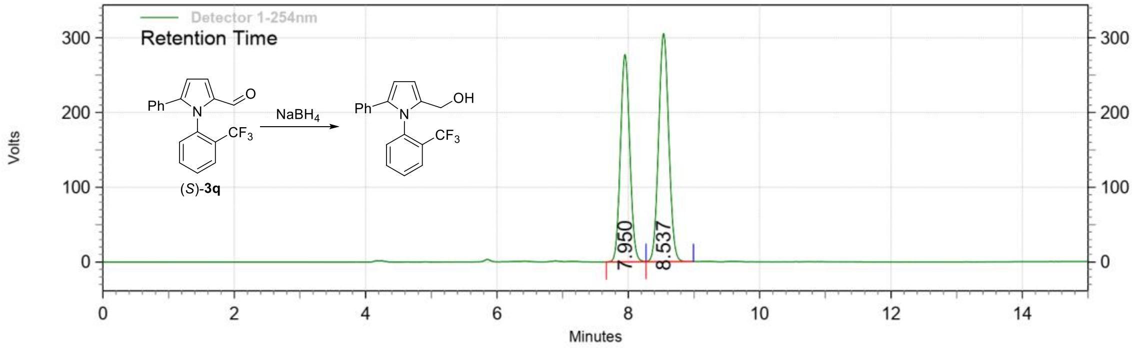

Retention Time	Area	Area %	Height	Height %
7.950	2745630	45.54	277531	47.61
8.537	3283121	54.46	305419	52.39
Totals	6028751	100.00	582950	100.00

*Chiral HPLC chromatograms of rac **4a**, recovered (R)-**4a** and aldehyde (S)-**5a***

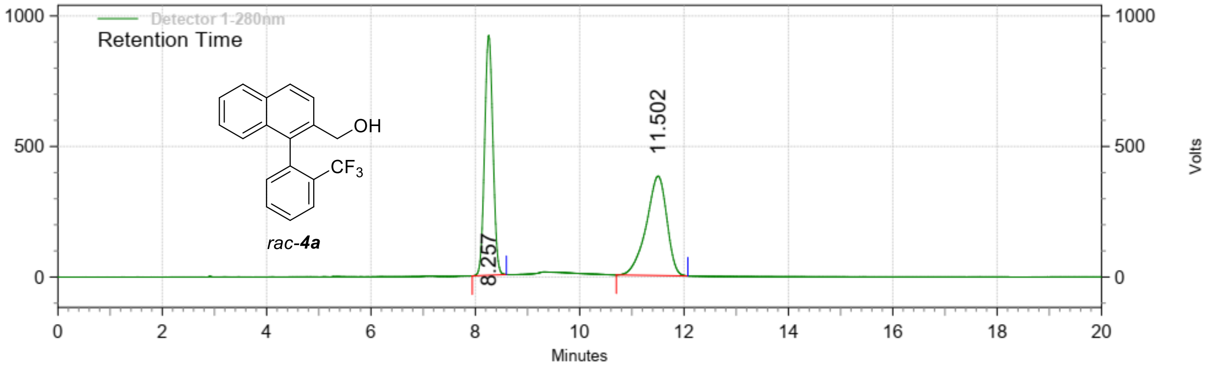

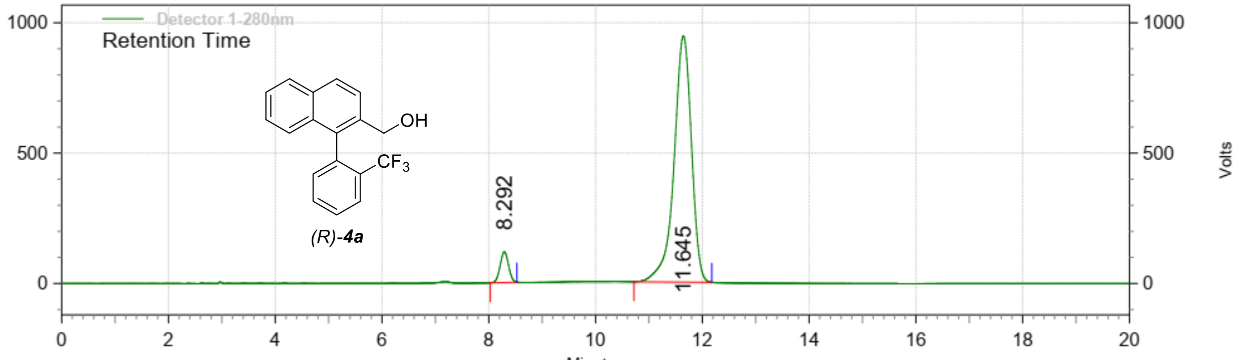

Retention Time	Area	Area %	Height	Height %
8.292	1287940	5.67	117649	11.09
11.645	21425772	94.33	943626	88.91
Totals	22713712	100.00	1061275	100.00

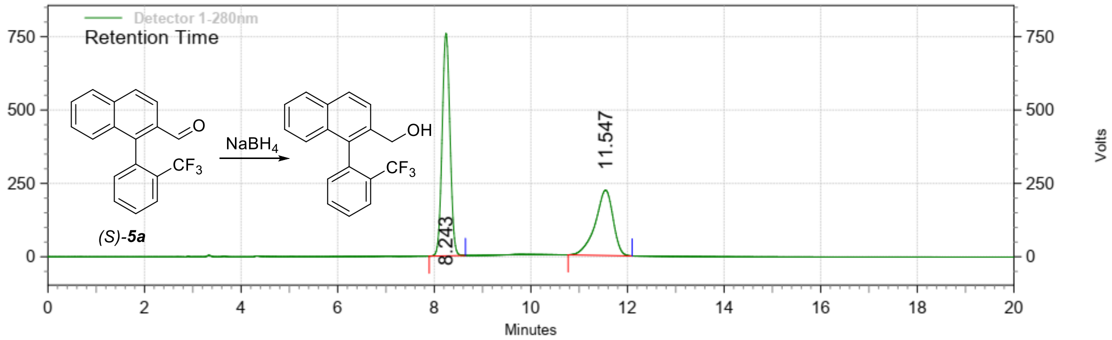

Retention Time	Area	Area %	Height	Height %
8.243	8544153	59.99	757965	77.28
11.547	5699107	40.01	222857	22.72
Totals	14243260	100.00	980822	100.00

*Chiral HPLC chromatograms of rac **4b**, recovered (R)-**4b** and aldehyde (S)-**5b***

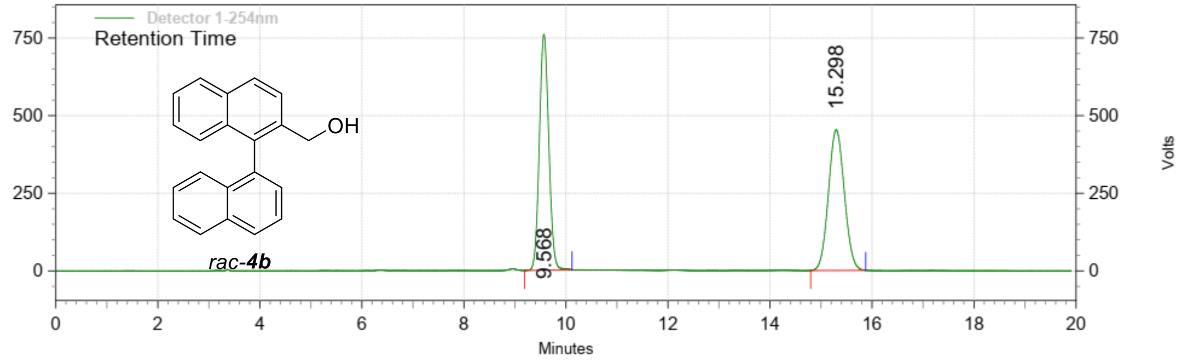

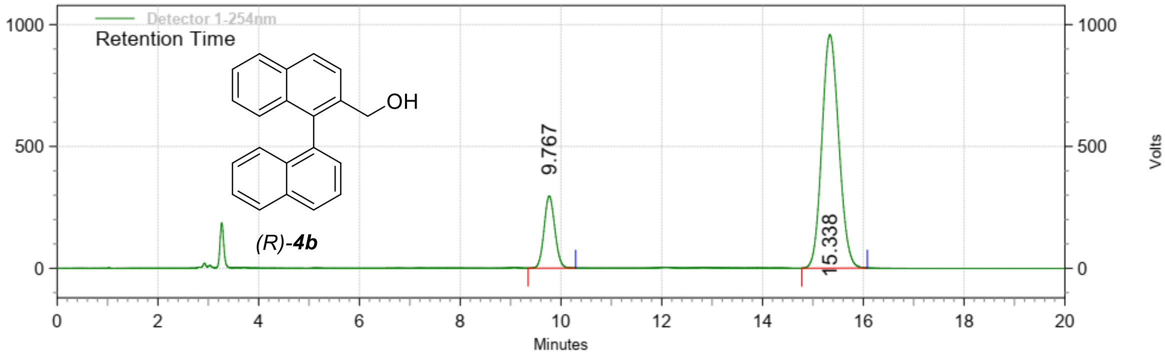

Retention Time	Area	Area %	Height	Height %
9.767	4390407	16.43	296167	23.61
15.338	22338007	83.57	958021	76.39
Totals	26728414	100.00	1254188	100.00

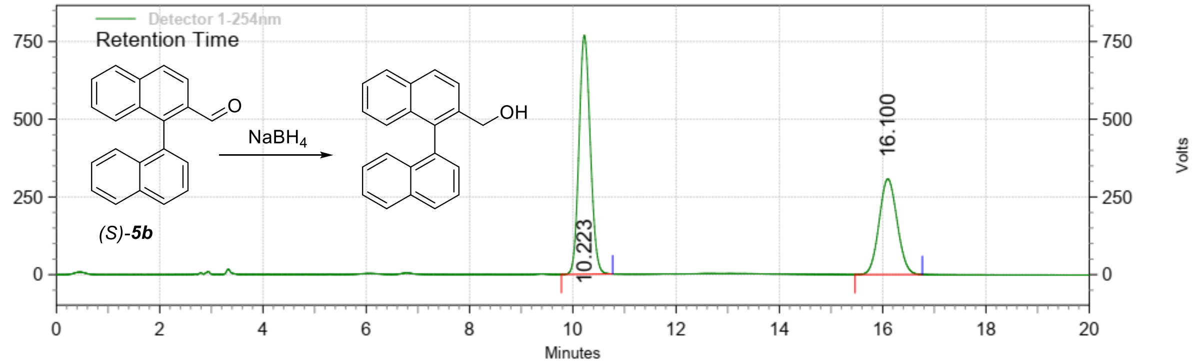

Retention Time	Area	Area %	Height	Height %
10.223	11680797	60.97	768656	71.41
16.100	7476141	39.03	307674	28.59
Totals	19156938	100.00	1076330	100.00

Chiral HPLC *chromatograms* of *rac* **4c**, *recovered (R)-**4c** and aldehyde (S)-**5c***

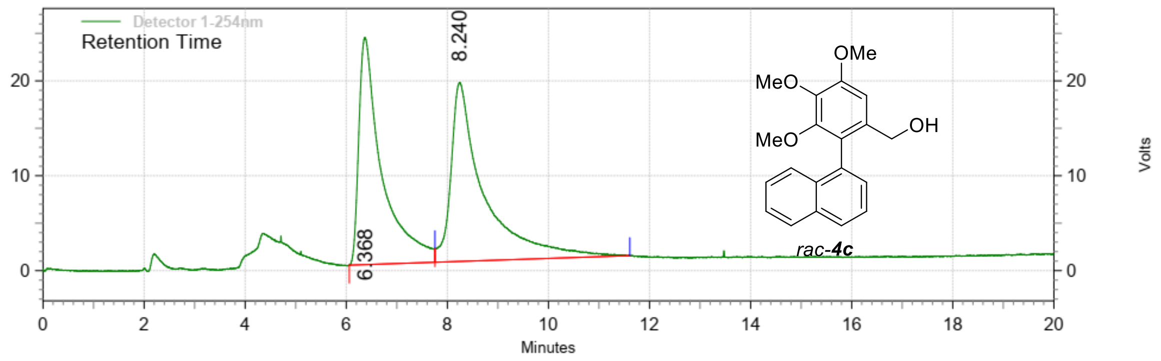

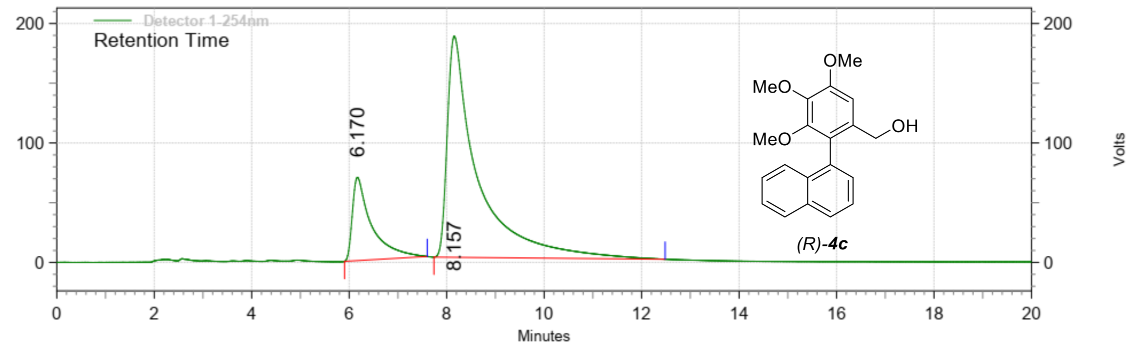

Retention Time	Area	Area %	Height	Height %
6.170	1911586	19.18	69704	27.36
8.157	8056934	80.82	185065	72.64
Totals	9968520	100.00	254769	100.00

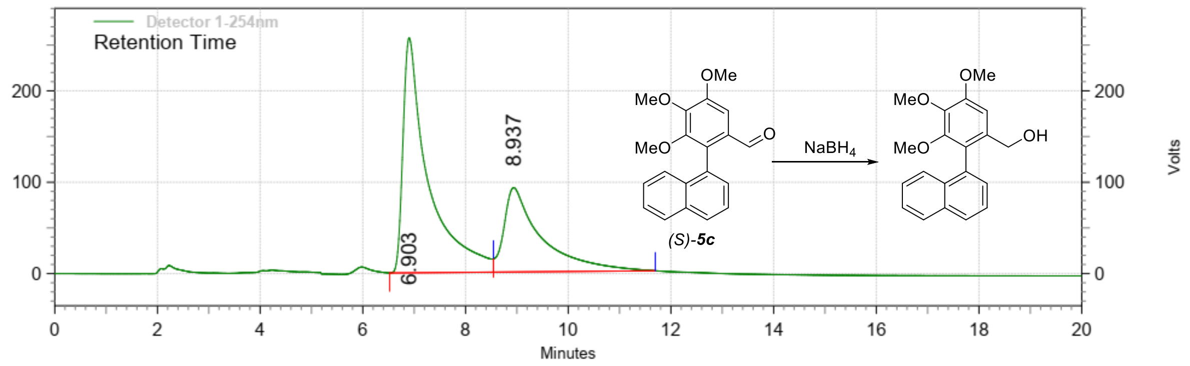

Retention Time	Area	Area %	Height	Height %
6.903	8787930	65.43	256887	73.58
8.937	4643535	34.57	92227	26.42
Totals	13431465	100.00	349114	100.00

Chiral HPLC *chromatograms* of *rac* **4d**, *recovered (R)-**4d** and aldehyde (S)-**5d***

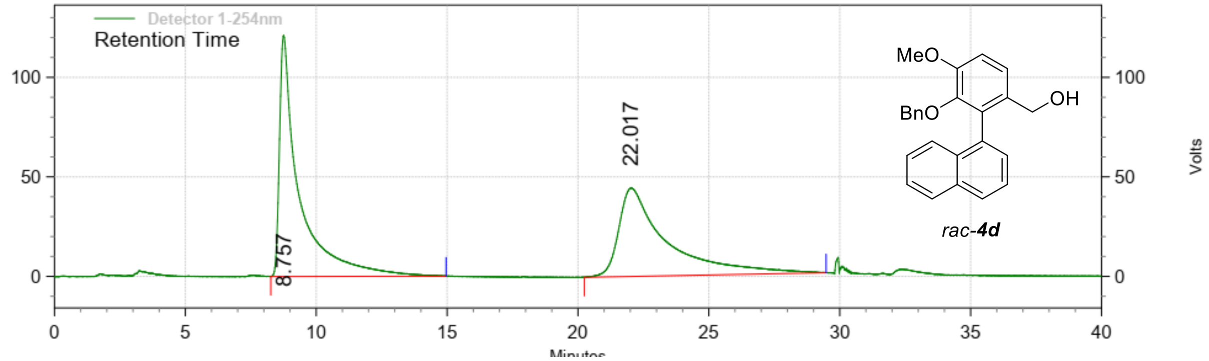

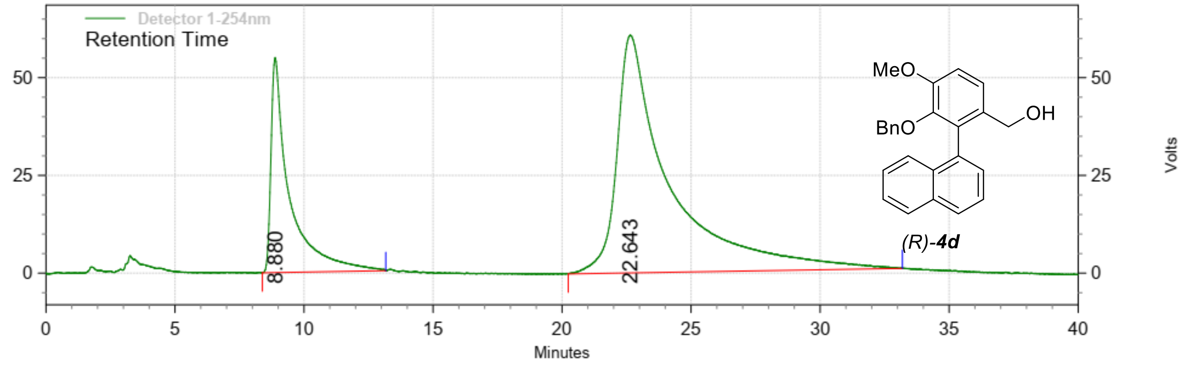

Retention Time	Area	Area %	Height	Height %
8.880	2724914	23.87	55003	47.47
22.643	8689910	76.13	60866	52.53

Totals				
	11414824	100.00	115869	100.00

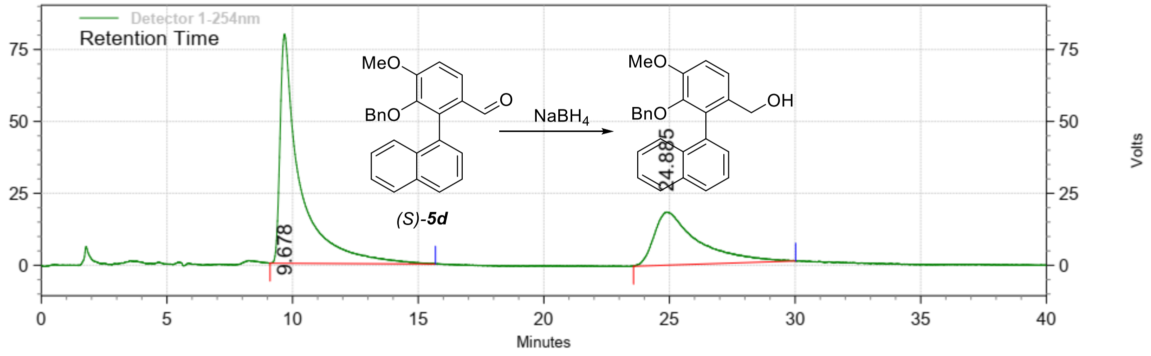

Retention Time	Area	Area %	Height	Height %
9.678	4383320	66.07	79669	81.18
24.885	2251492	33.93	18472	18.82

Totals				
	6634812	100.00	98141	100.00

Chiral HPLC *chromatograms* of *rac* **4e**, *recovered (R)-**4e** and aldehyde (S)-**5e***

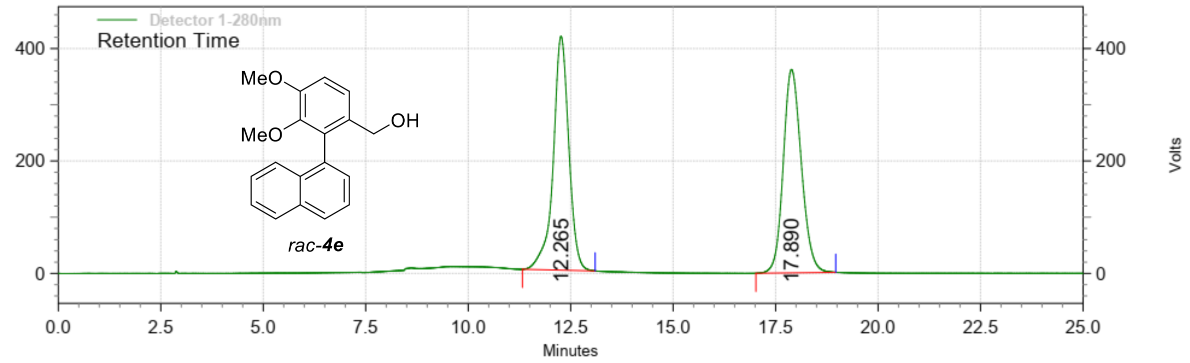

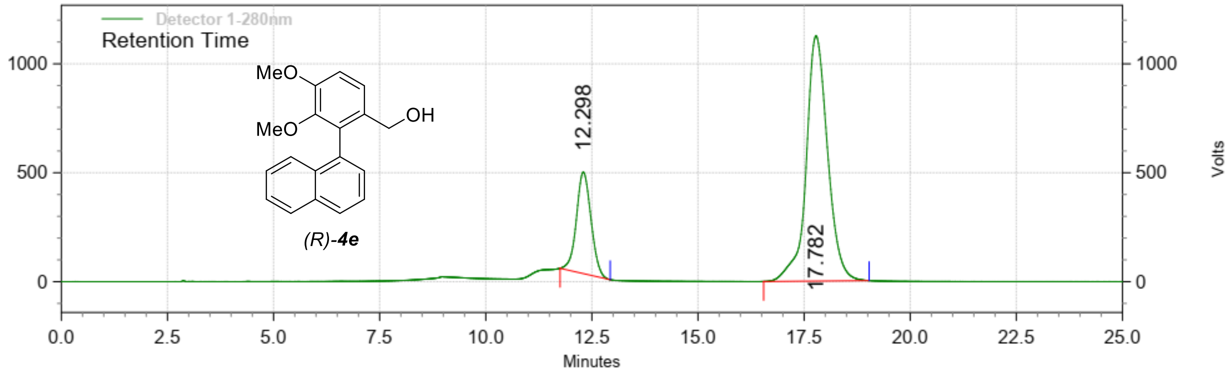

Retention Time	Area	Area %	Height	Height %
12.298	11172141	21.99	465492	29.26
17.782	39640000	78.01	1125207	70.74
Totals	50812141	100.00	1590699	100.00

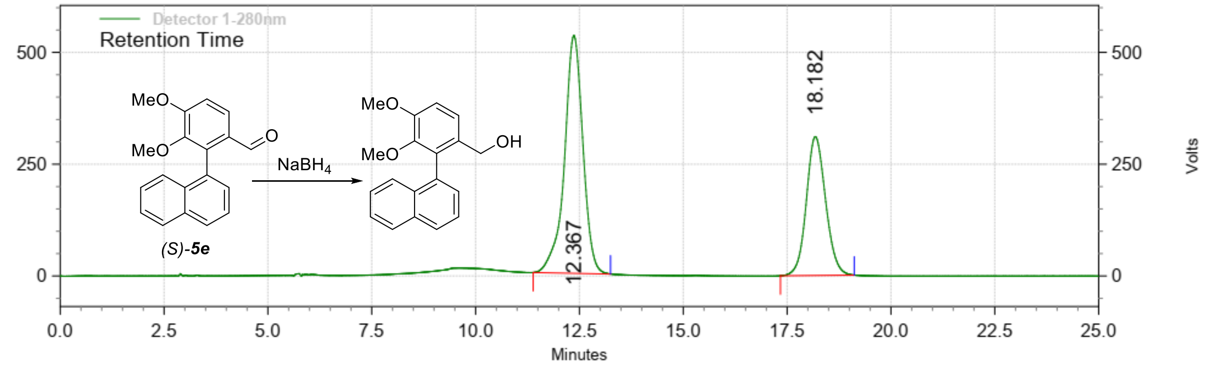

Retention Time	Area	Area %	Height	Height %
12.367	16451398	62.12	531990	63.14
18.182	10029829	37.88	310517	36.86
Totals	26481227	100.00	842507	100.00

Chiral HPLC *chromatograms* of *rac* **4f**, *recovered (R)-**4f** and aldehyde (S)-**5f***

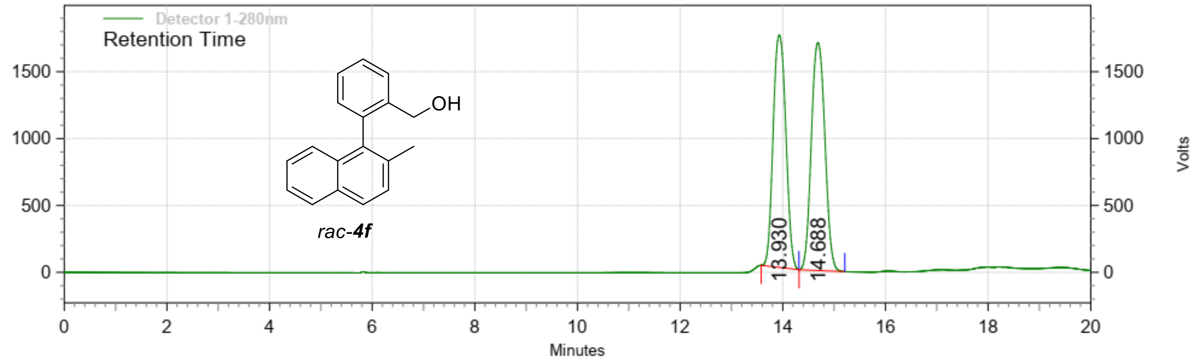

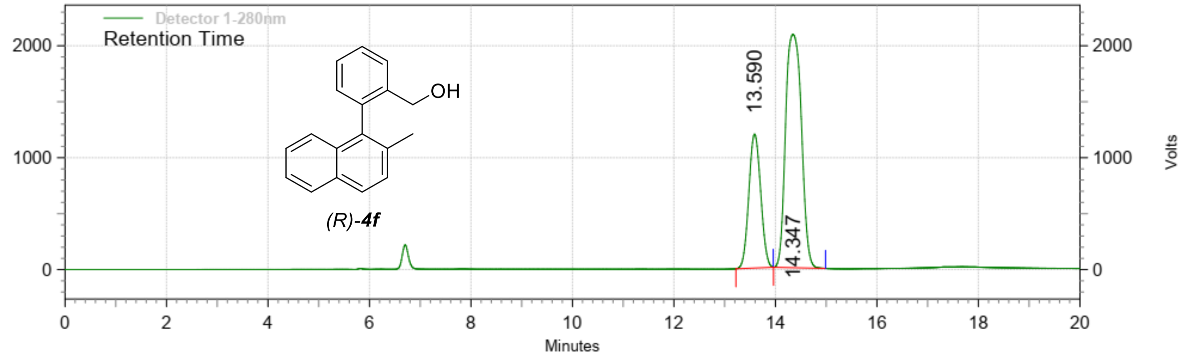

Retention Time	Area	Area %	Height	Height %
13.590	19430343	30.06	1196438	36.47
14.347	45206997	69.94	2084038	63.53
Totals	64637340	100.00	3280476	100.00

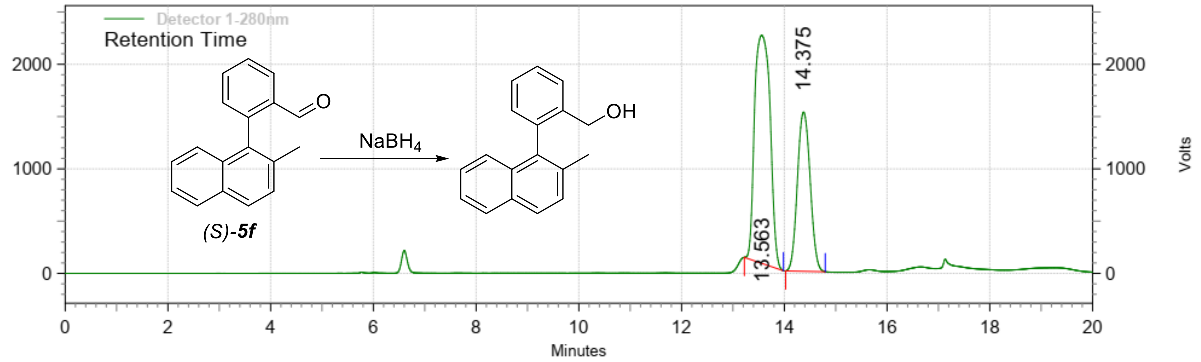

Retention Time	Area	Area %	Height	Height %
13.563	46075645	63.68	2181086	58.89
14.375	26275178	36.32	1522698	41.11
Totals	72350823	100.00	3703784	100.00

Chiral HPLC *chromatograms* of *rac* **4g**, *recovered (R)-**4g** and aldehyde (S)-**5g***

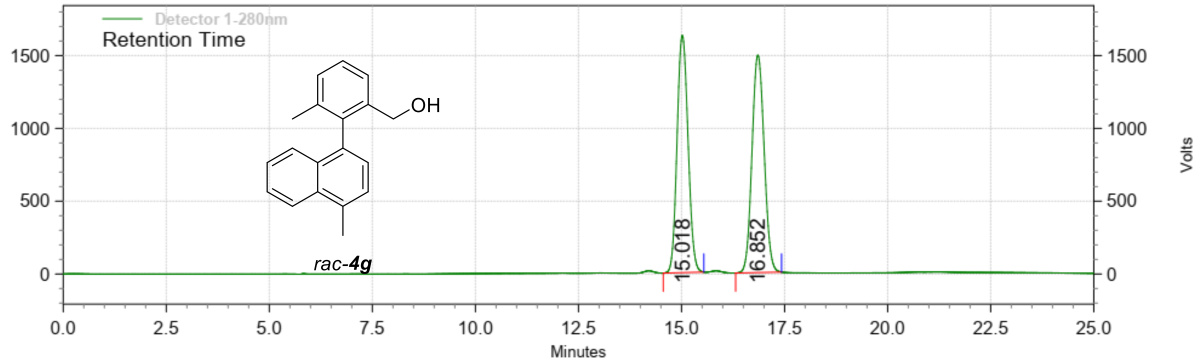

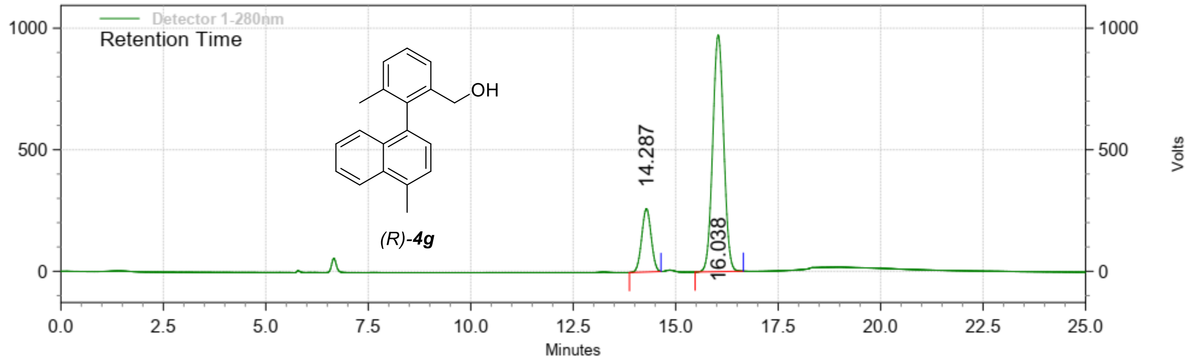

Retention Time	Area	Area %	Height	Height %
14.287	4175360	18.71	260540	21.12
16.038	18145301	81.29	973121	78.88
Totals	22320661	100.00	1233661	100.00

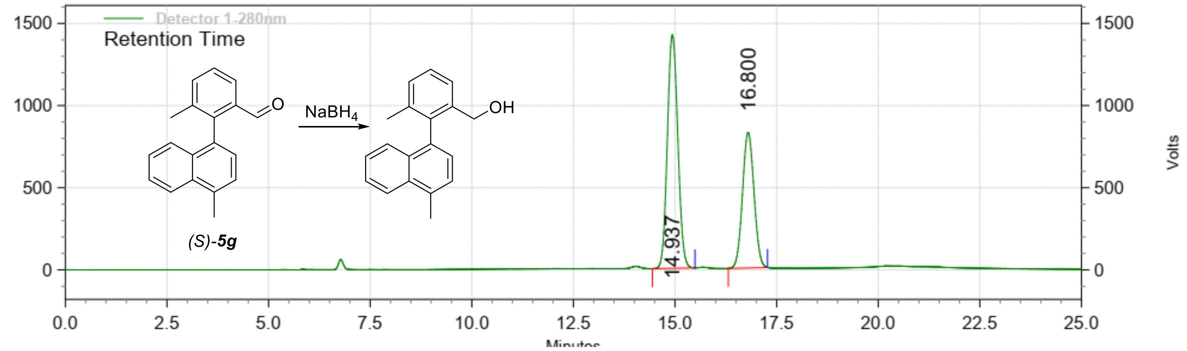

Retention Time	Area	Area %	Height	Height %
14.937	25431593	61.07	1421190	63.25
16.800	16214246	38.93	825778	36.75
Totals	41645839	100.00	2246968	100.00

Chiral HPLC *chromatograms* of *rac* **4h**, *recovered (R)-* **4h** *and aldehyde (S)-**3f***

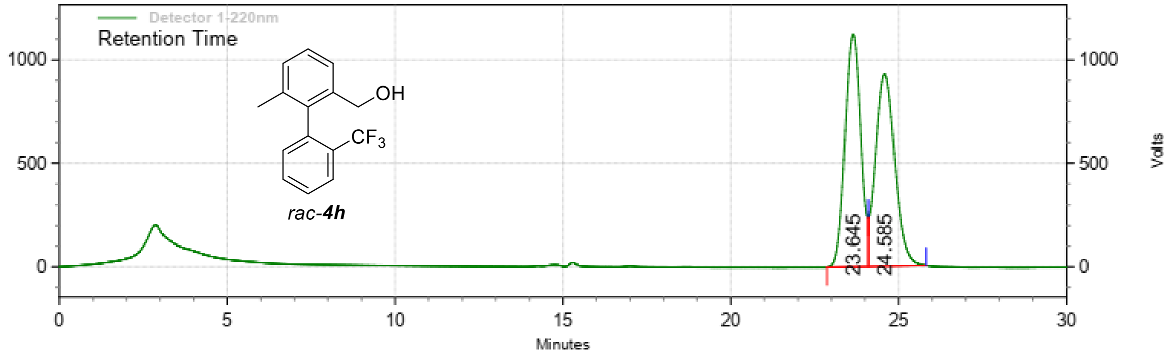

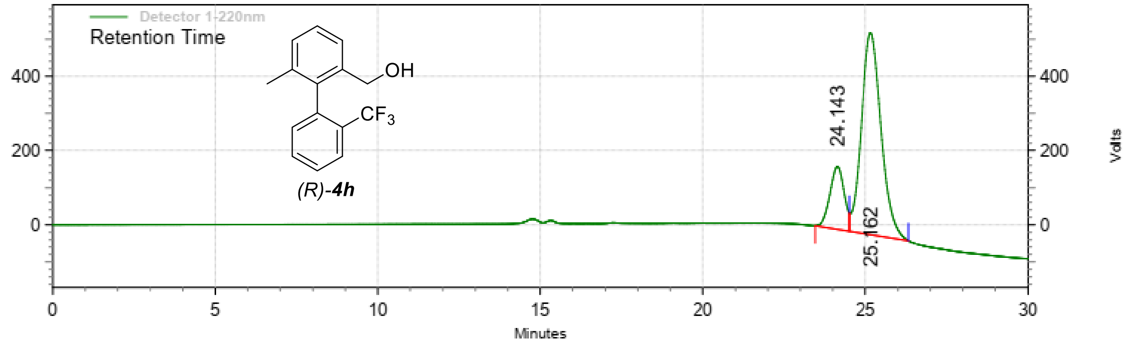

Retention Time	Area	Area %	Height	Height %
24.143	5101761	18.22	169129	23.74
25.162	22902888	81.78	543434	76.26
Totals	28004649	100.00	712563	100.00

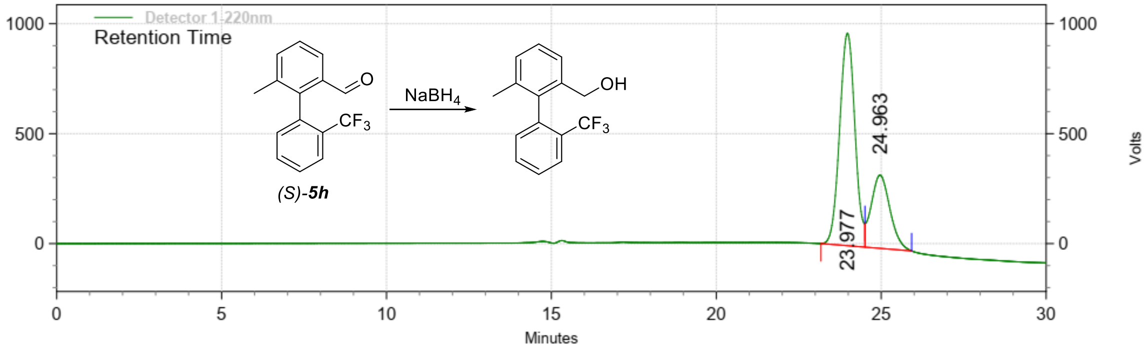

Retention Time	Area	Area %	Height	Height %
23.977	31179725	70.23	965225	74.33
24.963	13219381	29.77	333405	25.67
Totals	44399106	100.00	1298630	100.00

Chiral HPLC *chromatograms* of *rac* **4i**, *recovered (R)-* **4i** *and aldehyde (S)-**5i***

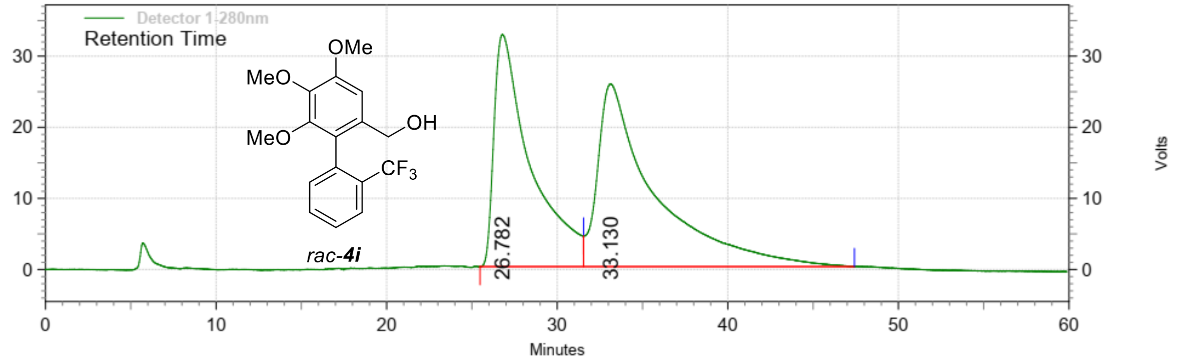

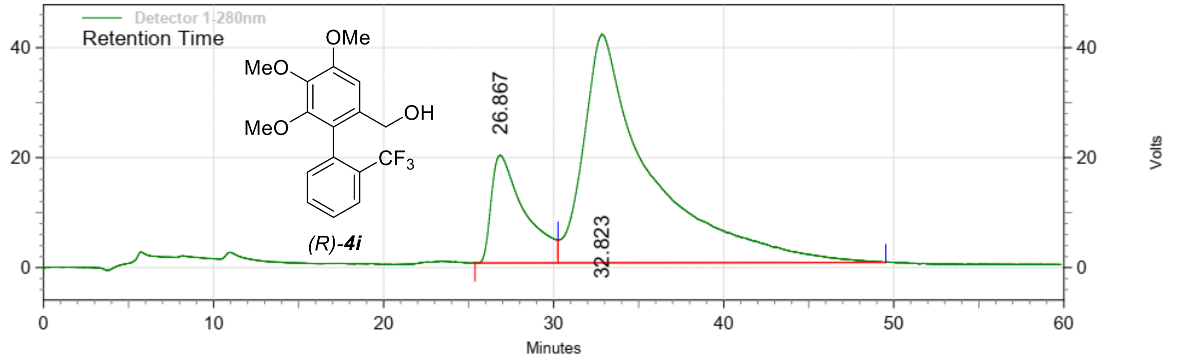

Retention Time	Area	Area %	Height	Height %
26.867	2666126	18.94	19599	32.04
32.823	11414278	81.06	41575	67.96
Totals	14080404	100.00	61174	100.00

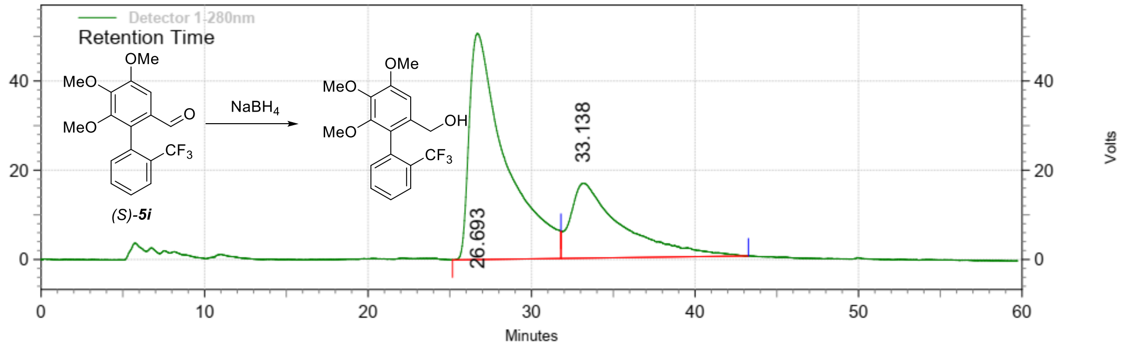

Retention Time	Area	Area %	Height	Height %
26.693	7848718	68.01	50667	75.10
33.138	3691789	31.99	16801	24.90
Totals	11540507	100.00	67468	100.00

Chiral HPLC *chromatograms* of *rac* **4j**, *recovered (R)-* **4j** *and aldehyde (S)-**5j***

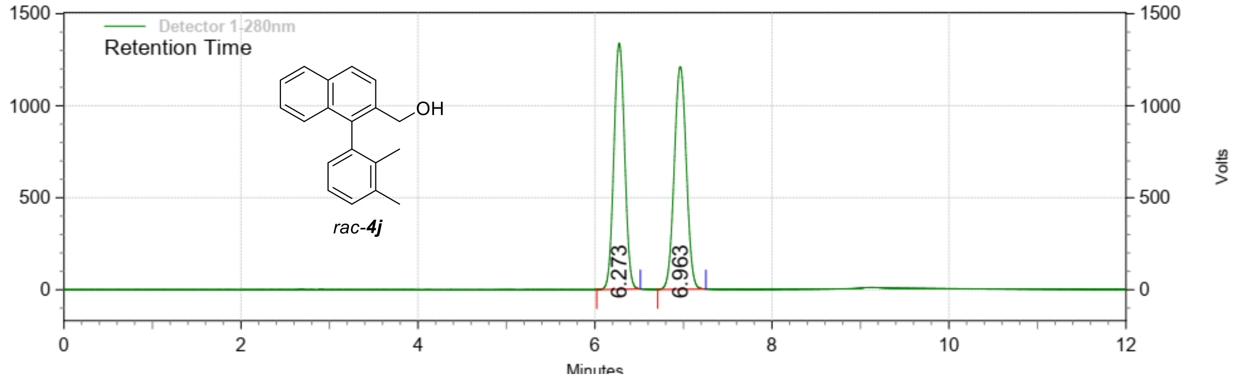

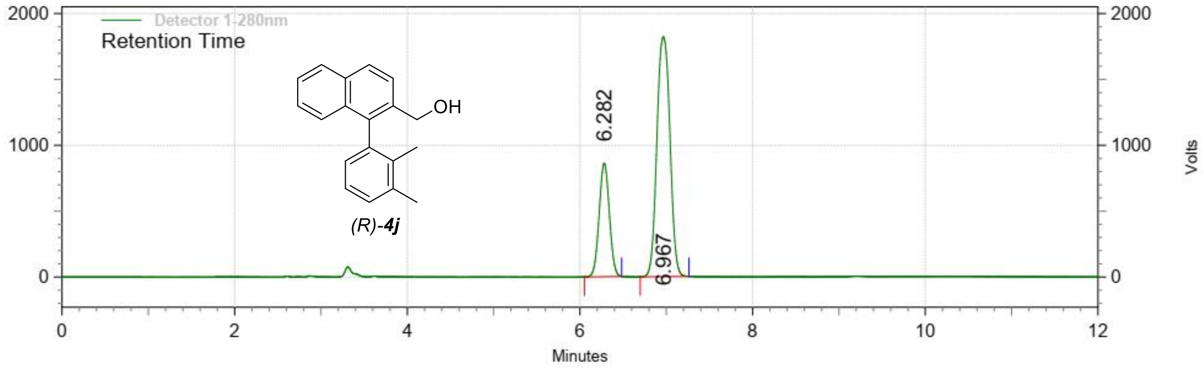

Retention Time	Area	Area %	Height	Height %
6.282	7099055	27.06	861470	32.11
6.967	19133897	72.94	1821248	67.89
Totals	26232952	100.00	2682718	100.00

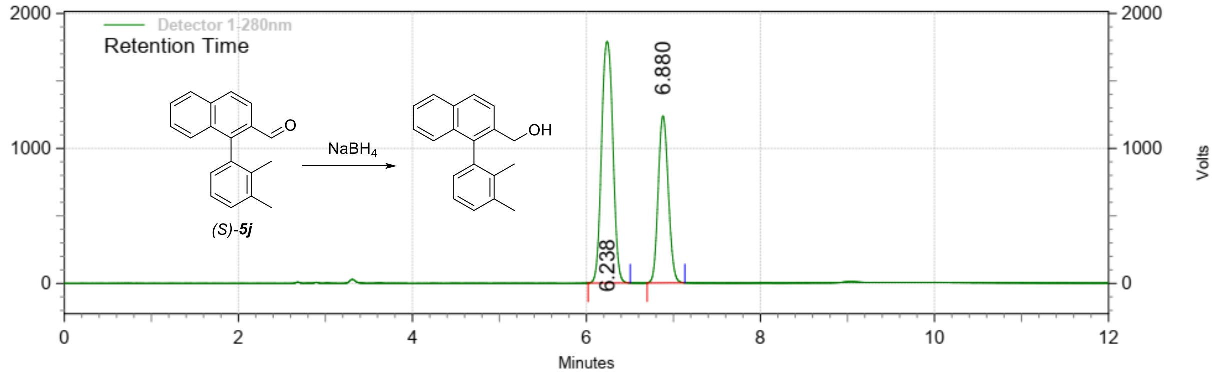

Retention Time	Area	Area %	Height	Height %
6.238	16206140	61.85	1788176	59.14
6.880	9995153	38.15	1235601	40.86
Totals	26201293	100.00	3023777	100.00

Chiral HPLC *chromatograms* of *rac* **4k**, *recovered (R)*-**4k** *and aldehyde (S)*-***5k***

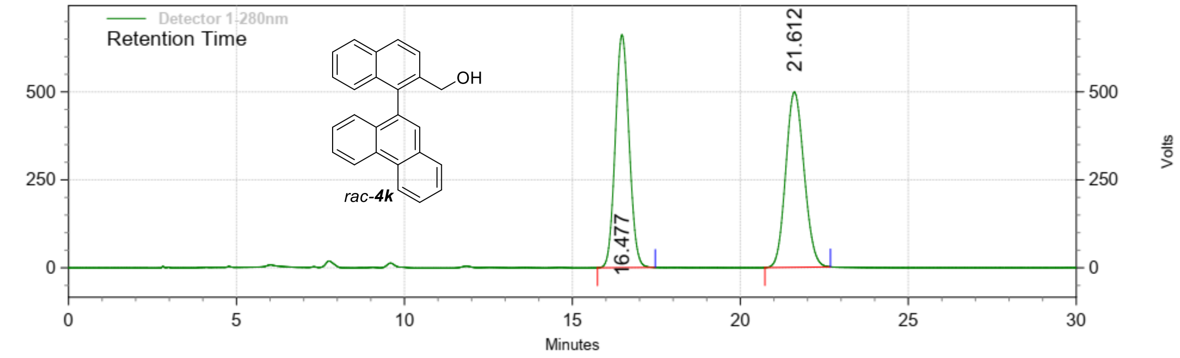

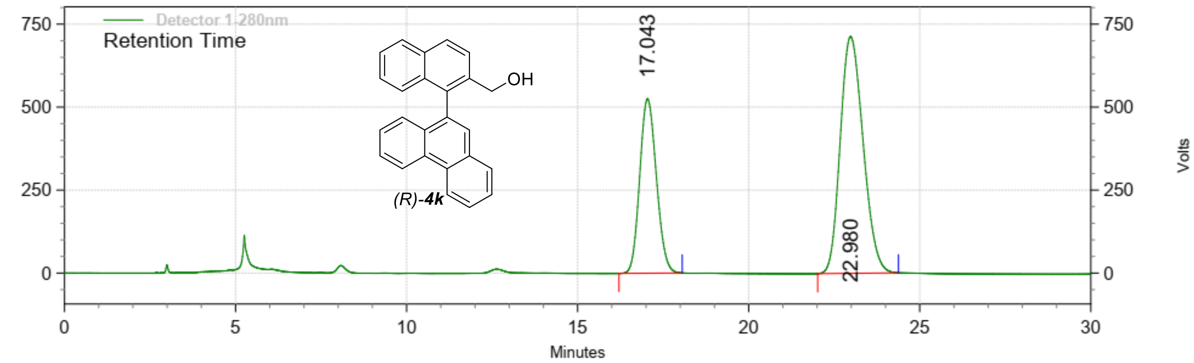

Retention Time	Area	Area %	Height	Height %
17.043	17822777	34.99	525906	42.43
22.980	33111429	65.01	713682	57.57
Totals	50934206	100.00	1239588	100.00

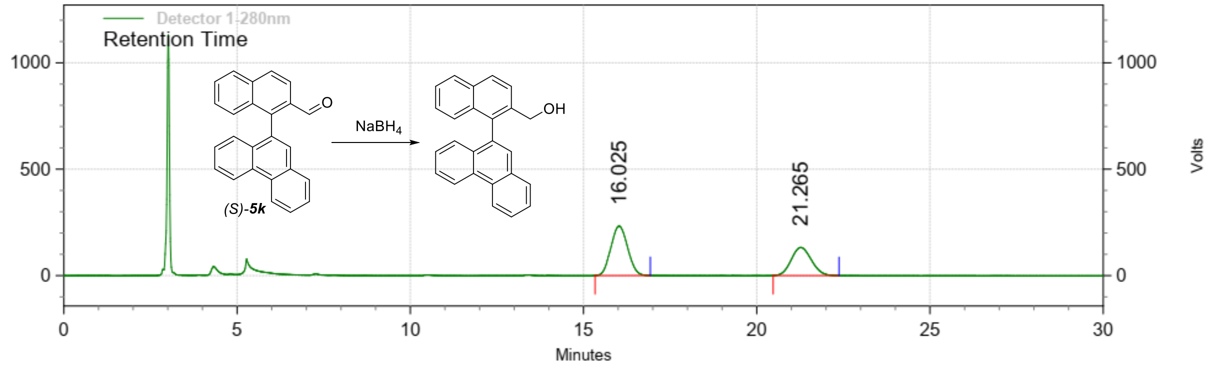

Retention Time	Area	Area %	Height	Height %
16.025	7834984	59.45	232467	63.78
21.265	5343505	40.55	132010	36.22
Totals	13178489	100.00	364477	100.00

Chiral HPLC *chromatograms* of *rac* **4l**, *recovered (R)*-**4l** *and aldehyde (S)*-***5l***

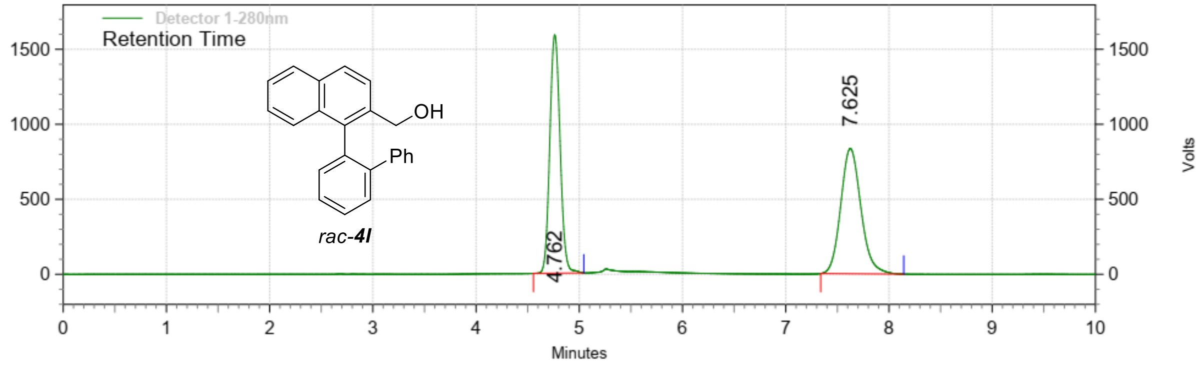

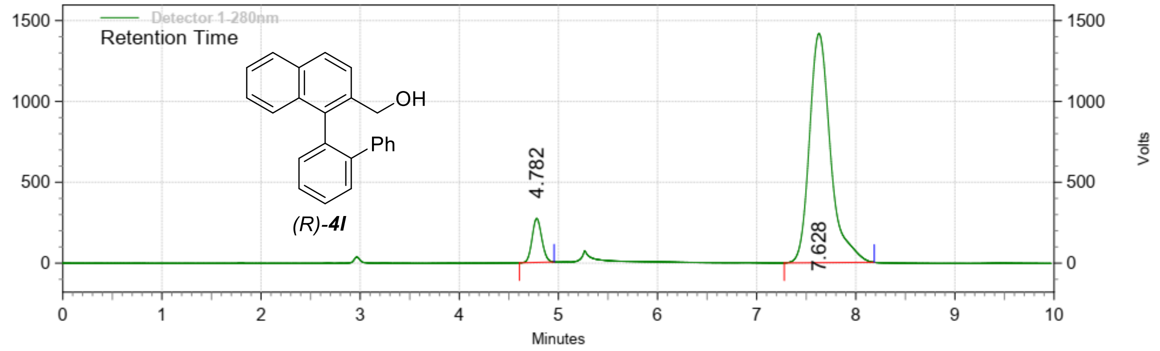

Retention Time	Area	Area %	Height	Height %
4.782	1842573	8.11	271691	16.10
7.628	20887713	91.89	1416318	83.90
Totals	22730286	100.00	1688009	100.00

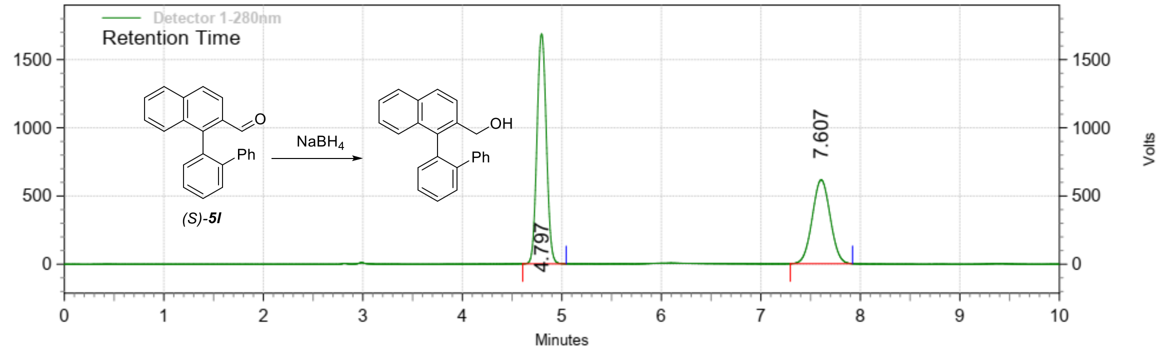

Retention Time	Area	Area %	Height	Height %
4.797	11019847	58.79	1685940	73.26
7.607	7724309	41.21	615447	26.74
Totals	18744156	100.00	2301387	100.00

Chiral HPLC *chromatograms* of *rac* **4m**, *recovered (R)*-**4m** *and aldehyde (S)-**5m***

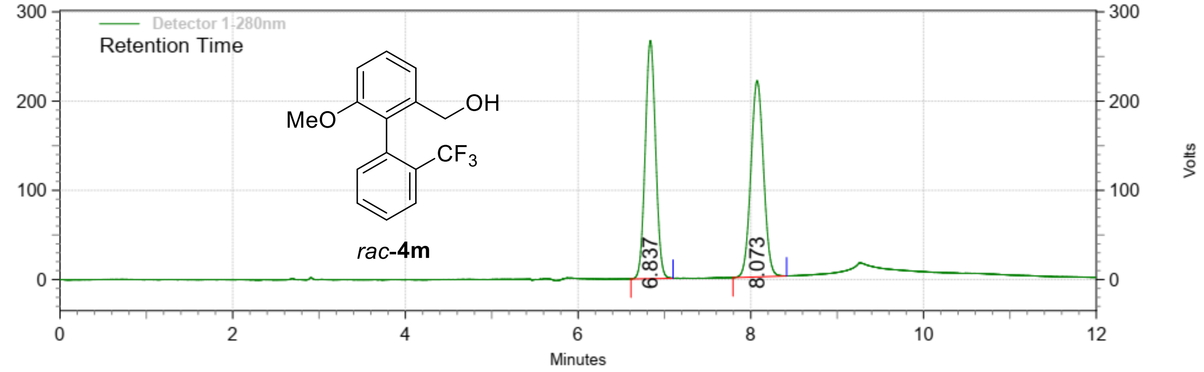

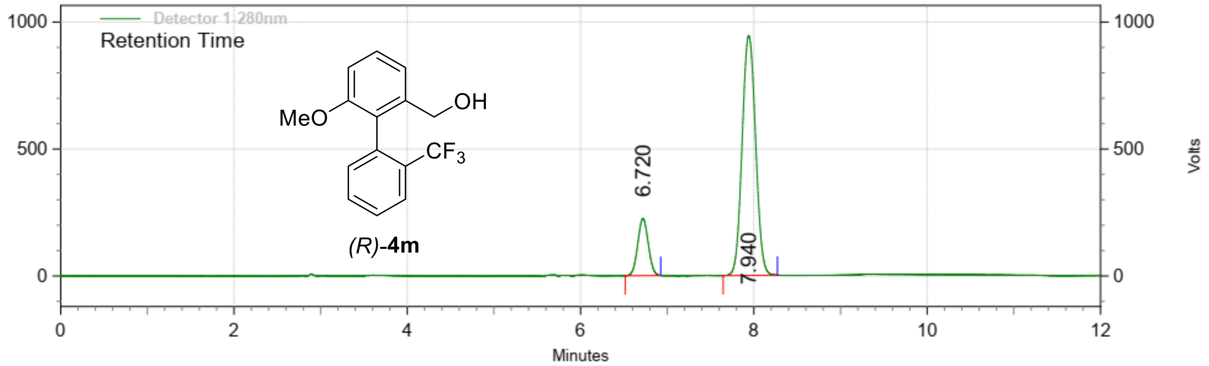

Retention Time	Area	Area %	Height	Height %
6.720	1903123	15.73	225346	19.26
7.940	10192064	84.27	944662	80.74
Totals	12095187	100.00	1170008	100.00

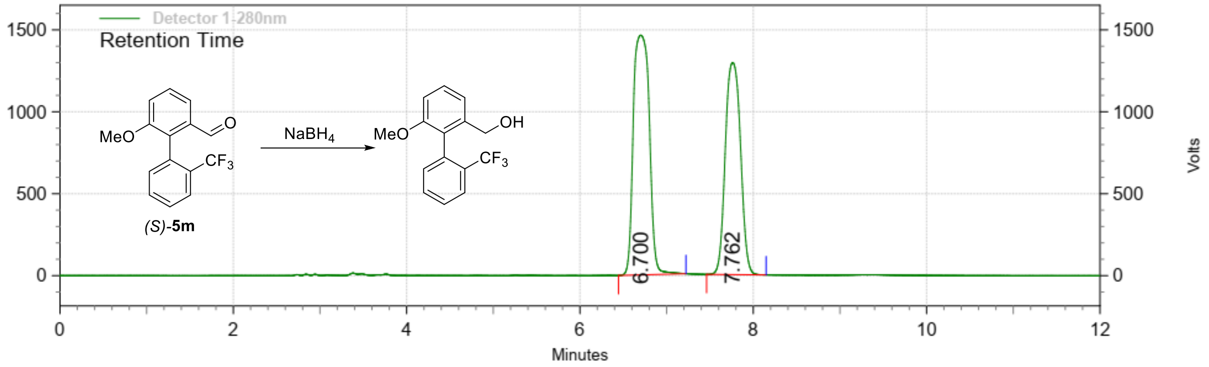

Retention Time	Area	Area %	Height	Height %
6.700	18823197	54.36	1462665	53.06
7.762	15805341	45.64	1294185	46.94
Totals	34628538	100.00	2756850	100.00

Chiral HPLC *chromatograms* of *rac* **4n**, *recovered (R)-***4n** *and aldehyde (S)-***5n**

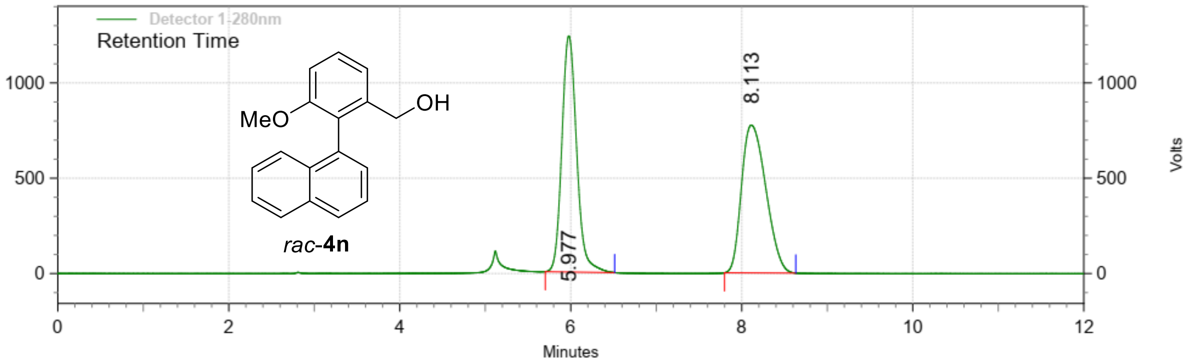

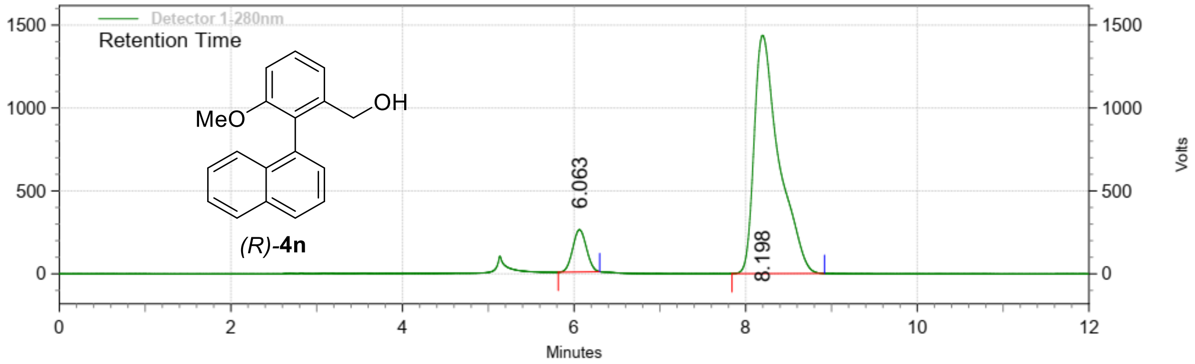

Retention Time	Area	Area %	Height	Height %
6.063	2839490	8.95	255004	15.07
8.198	28886641	91.05	1436694	84.93
Totals	31726131	100.00	1691698	100.00

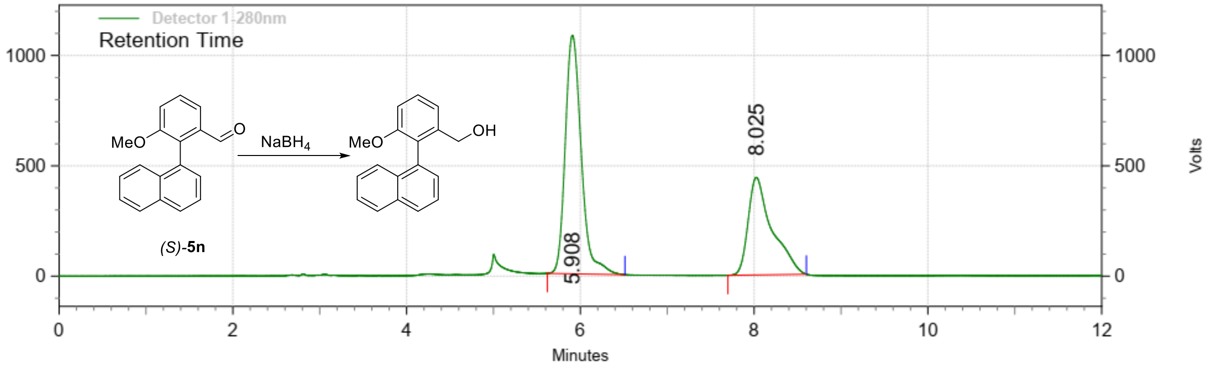

Retention Time	Area	Area %	Height	Height %
5.908	13831222	62.10	1081324	70.99
8.025	8442072	37.90	441941	29.01
Totals	22273294	100.00	1523265	100.00

Chiral HPLC chromatograms of *rac* **4o**, *recovered (R)-***4o** *and aldehyde (S)-**5o***

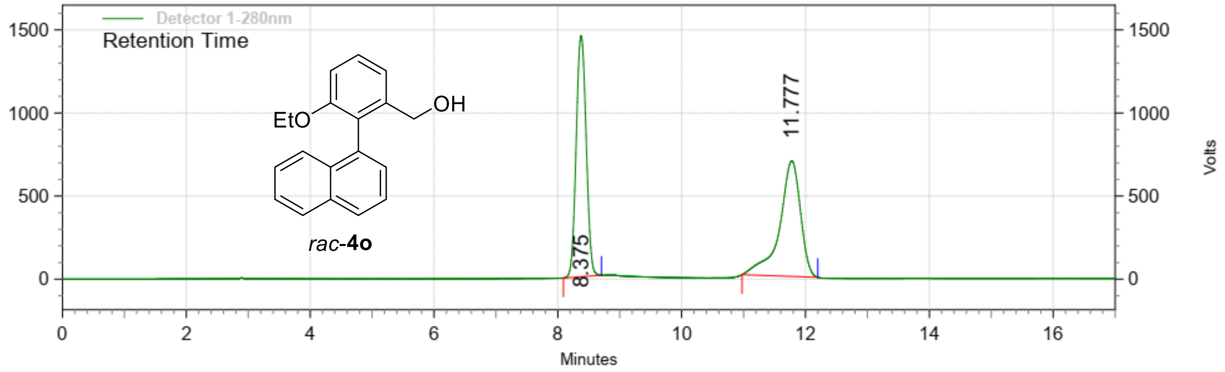

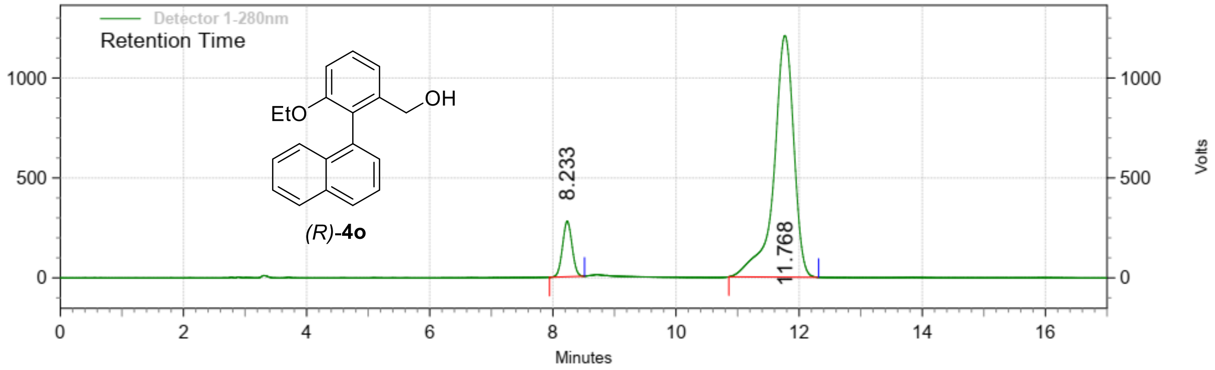

Retention Time	Area	Area %	Height	Height %
8.233	2991354	9.91	277769	18.69
11.768	27207029	90.09	1208426	81.31
Totals	30198383	100.00	1486195	100.00

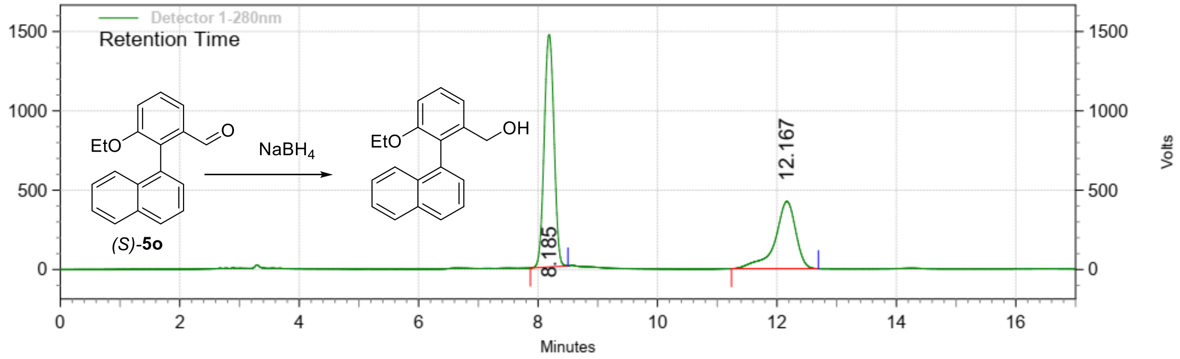

Retention Time	Area	Area %	Height	Height %
8.185	16660635	61.96	1465462	77.56
12.167	10227004	38.04	423915	22.44
Totals	26887639	100.00	1889377	100.00

Chiral HPLC chromatograms of *rac* **4p**, *recovered (R)*-**4p** *and aldehyde (S)*-***5p***

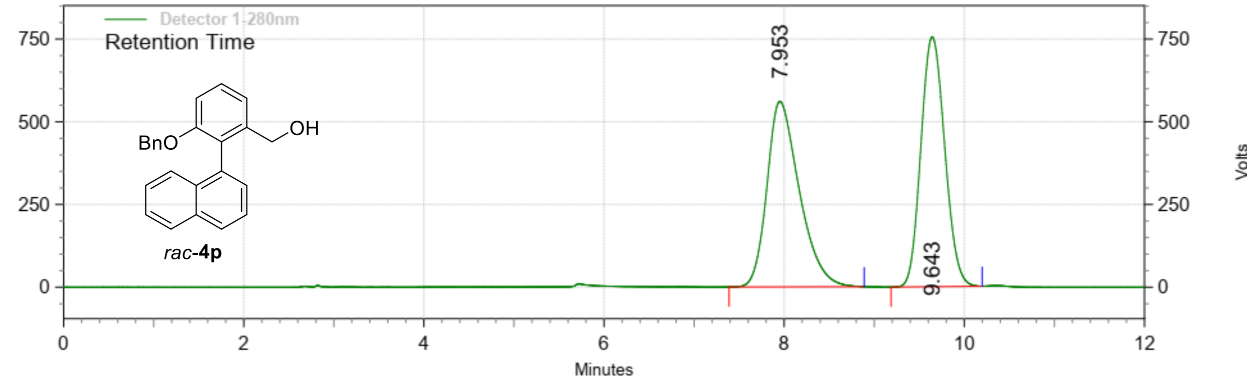

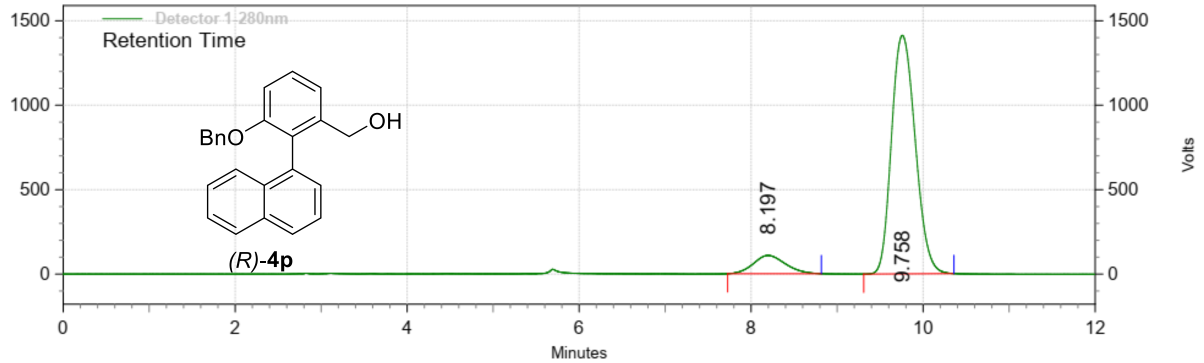

Retention Time	Area	Area %	Height	Height %
8.197	2806732	9.38	108348	7.12
9.758	27102379	90.62	1412570	92.88
Totals	29909111	100.00	1520918	100.00

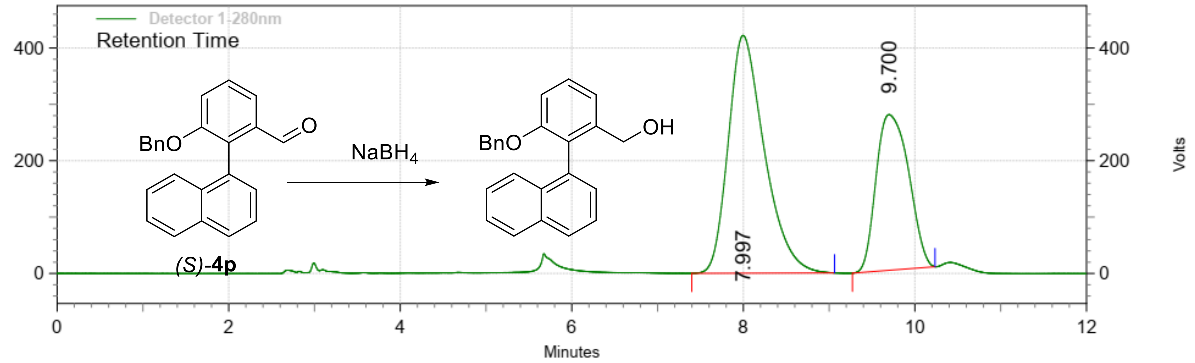

Retention Time	Area	Area %	Height	Height %
7.997	12343813	62.83	421755	60.44
9.700	7302738	37.17	276003	39.56
Totals	19646551	100.00	697758	100.00

Chiral HPLC chromatograms of *rac* **4q**, *recovered (R)-***4q** *and aldehyde (S)-***5q**

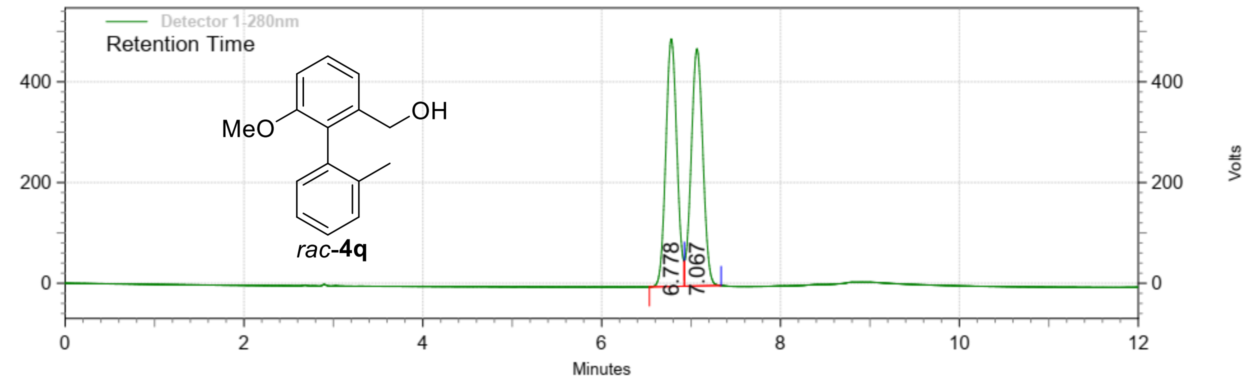

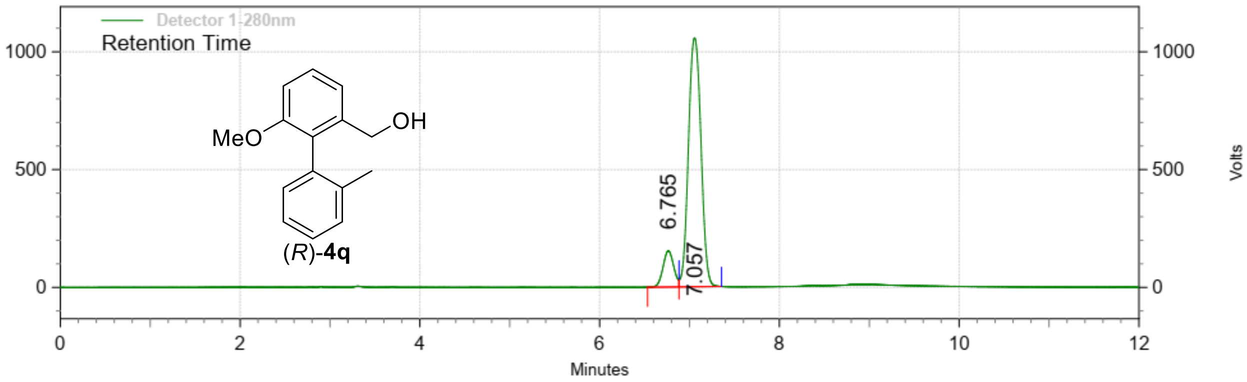

Retention Time	Area	Area %	Height	Height %
6.765	1267804	11.01	153552	12.69
7.057	10249976	88.99	1056228	87.31
Totals	11517780	100.00	1209780	100.00

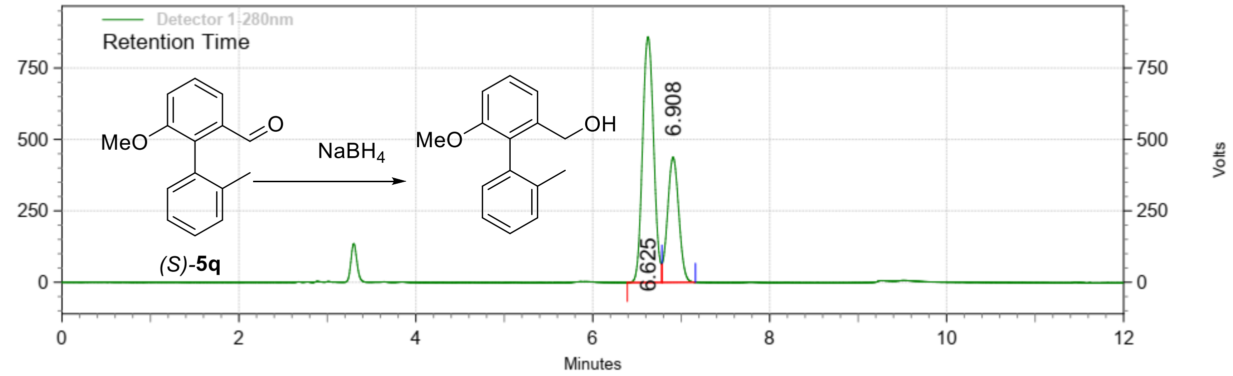

Retention Time	Area	Area %	Height	Height %
6.625	7531167	66.01	860210	66.26
6.908	3877296	33.99	438047	33.74
Totals	11408463	100.00	1298257	100.00

1 mmol Scale OKR of Racemic Biaryl Alcohol (4r)

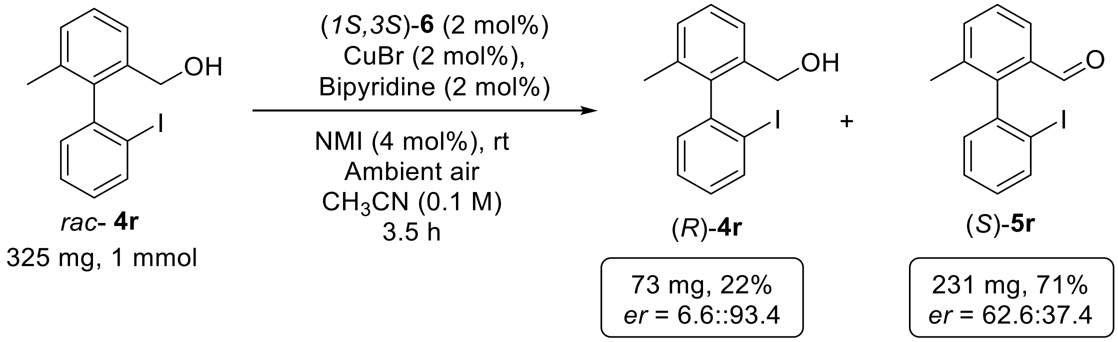

In a 25 ml single neck round bottom flask, racemic **4r** (325 mg, 1 mmol, 1 equiv.) was dissolved in 10 ml (0.1M) of analytical grade acetonitrile. Copper (I) bromide (2.87 mg, 0.02 mmol, 0.02 equiv.), bipyridine (3.12 mg, 0.02 mmol, 0.02 equiv.), hydroxylamine precatalyst (*1S,3S*)-**1** (15.32 mg, 0.02 mmol, 0.02 equiv.) and N-methyl imidazole [3.28 mg, 0.04 mmol, 0.04 equiv. (0.4 ml of 0.1 M acetonitrile solution)] were added to the reaction flask. The resulting brown color reaction mixture was stirred at room temperature (23-25 °C) open to air. The reaction progress was monitored by ^{1}H NMR analysis of the crude reaction mixture. After 3.5 hours of oxidation, at 55% conversion, the reaction was stopped. The acetonitrile was removed from the reaction mixture under reduced pressure at room temperature on a rotavapor. The crude reaction mixture was subjected to flash column chromatography using hexane and ethyl acetate as the eluent to obtain recovered alcohol **4r** and the product aldehyde **5r**.

Chiral HPLC chromatograms of *rac* **4r**, *recovered (R)-**4r** and aldehyde (S)-**5r***

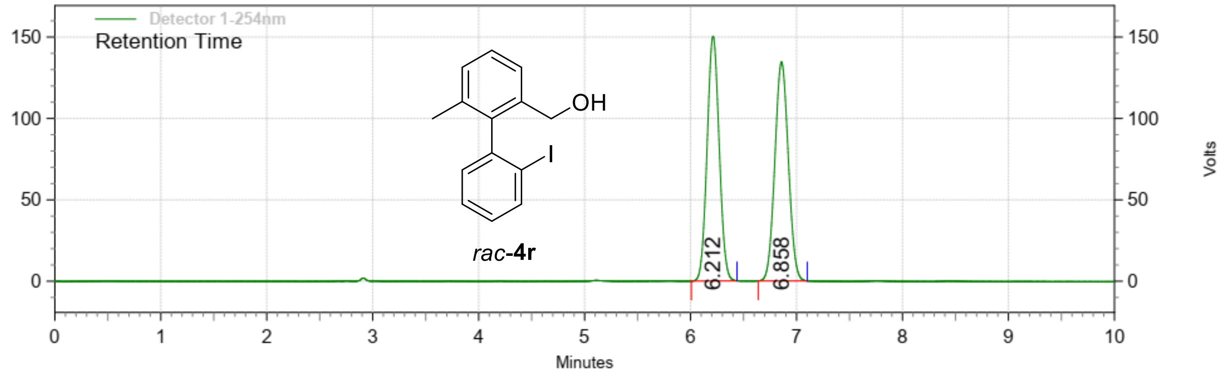

Retention Time	Area	Area %	Height	Height %
6.212	1188260	49.91	150375	52.78
6.858	1192584	50.09	134559	47.22

Totals				
	2380844	100.00	284934	100.00

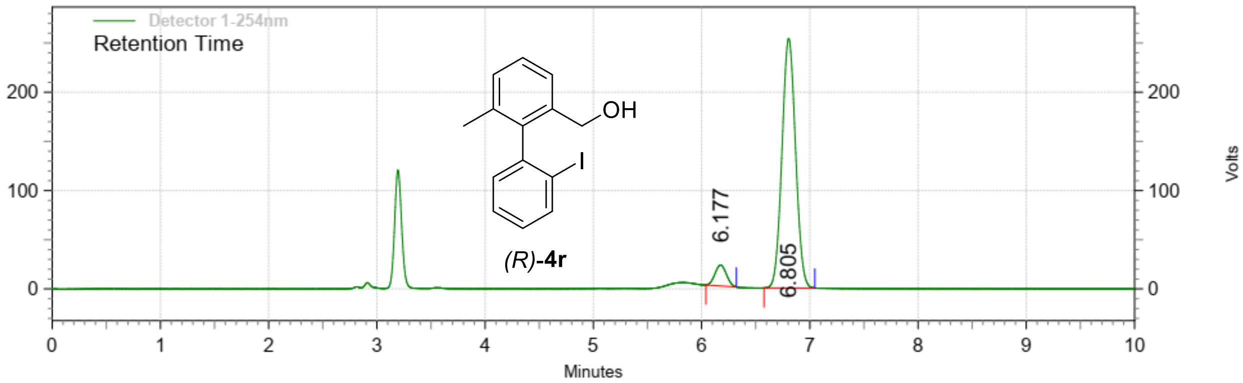

Retention Time	Area	Area %	Height	Height %
6.177	156280	6.59	21283	7.74
6.805	2213434	93.41	253835	92.26
Totals	2369714	100.00	275118	100.00

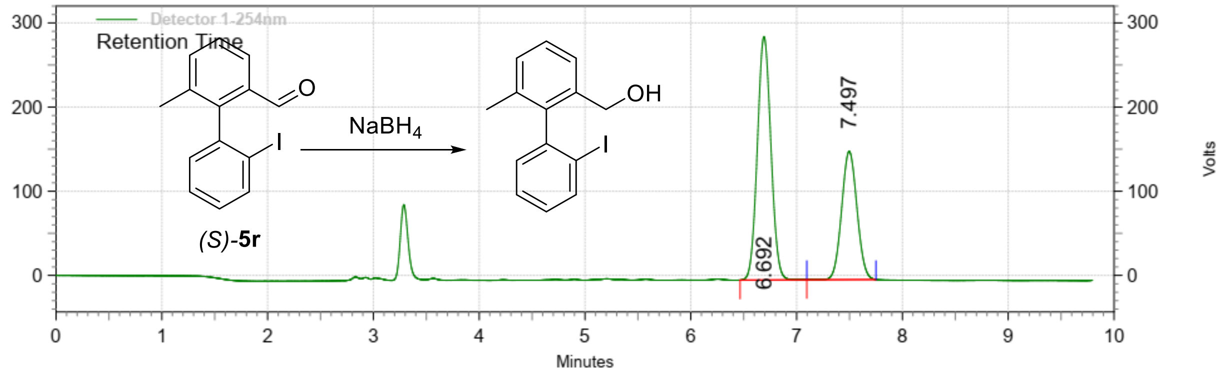

Retention Time	Area	Area %	Height	Height %
6.692	2581226	62.56	289257	65.48
7.497	1545031	37.44	152482	34.52
Totals	4126257	100.00	441739	100.00

Chiral HPLC chromatograms of *rac* **7a** and optically pure ***7a***

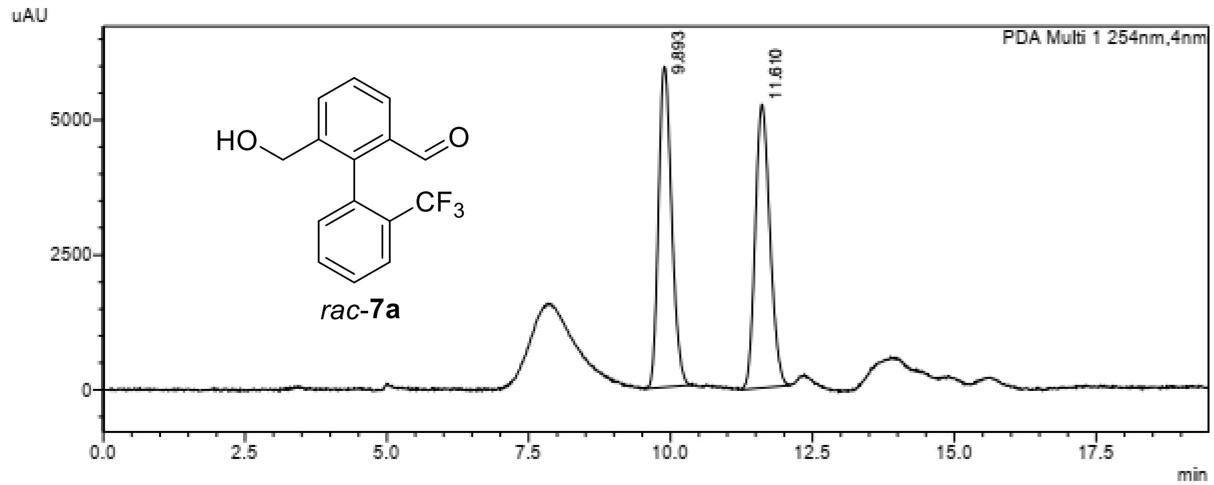

PDA Ch1 254nm

Peak#	Ret. Time	Area	Area%	Height
1	9.893	95894	50.249	5937
2	11.610	94945	49.751	5254
Total		190839	100.000	11192

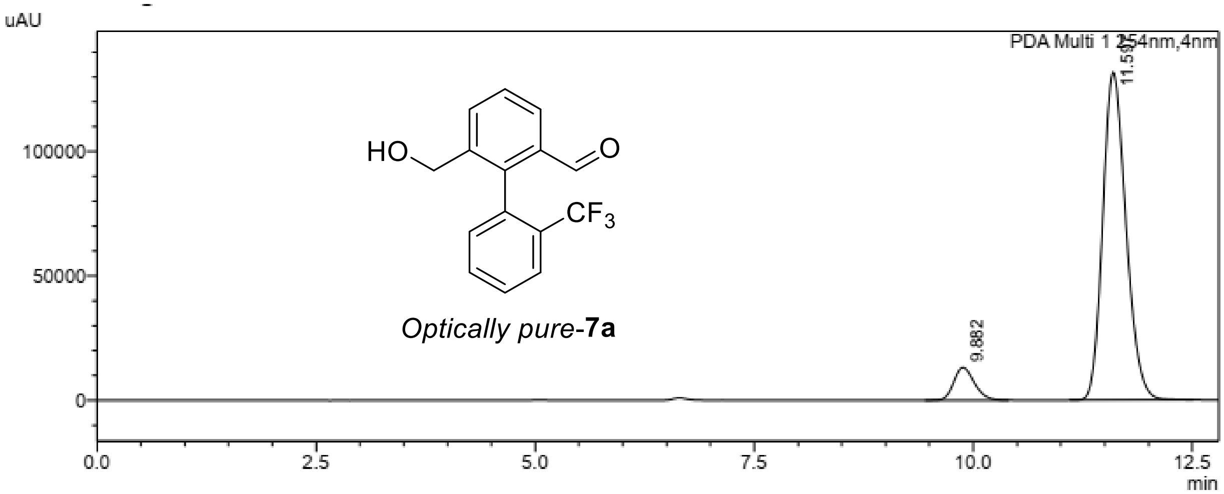

PDA Ch1 254nm

Peak#	Ret. Time	Area	Area%	Height
1	9.882	215036	8.119	13153
2	11.597	2433661	91.881	131788
Total		2648697	100.000	144941

Chiral HPLC chromatograms of *rac* **7b** and optically pure ***7b***

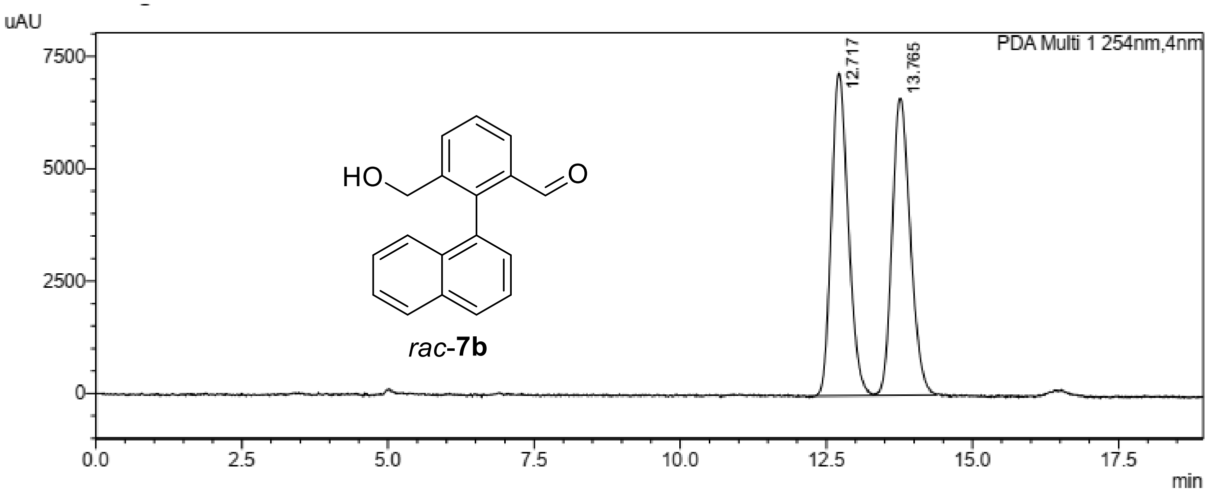

PDA Ch1 254nm

Peak#	Ret. Time	Area	Area%	Height
1	12.717	144521	50.220	7173
2	13.765	143256	49.780	6607
Total		287777	100.000	13780

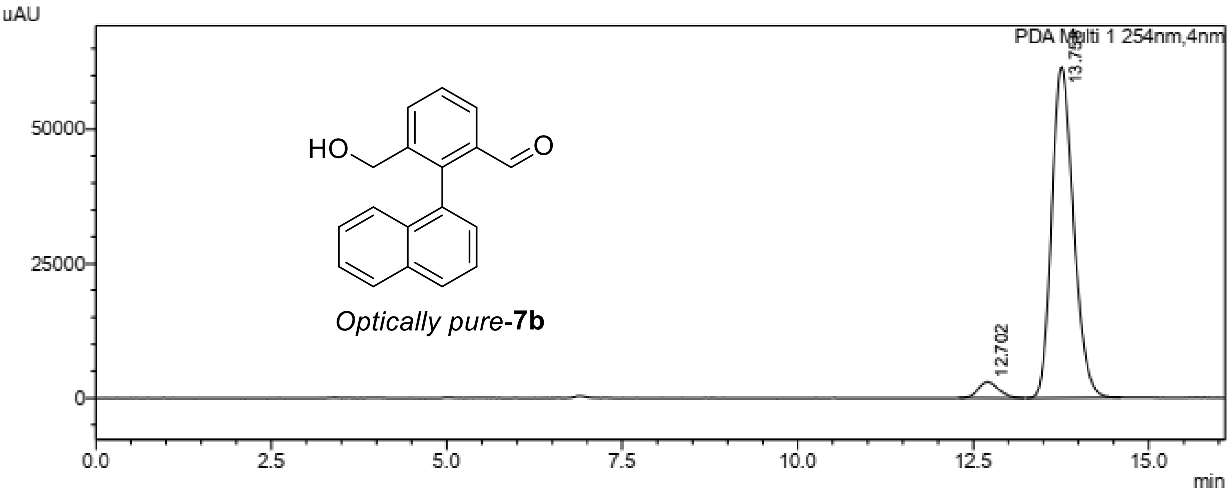

PDA Ch1 254nm

Peak#	Ret. Time	Area	Area%	Height
1	12.702	60338	4.326	2964
2	13.758	1334448	95.674	61477
Total		1394786	100.000	64441

Chiral HPLC chromatograms of *rac* **7c** and optically pure ***7c***

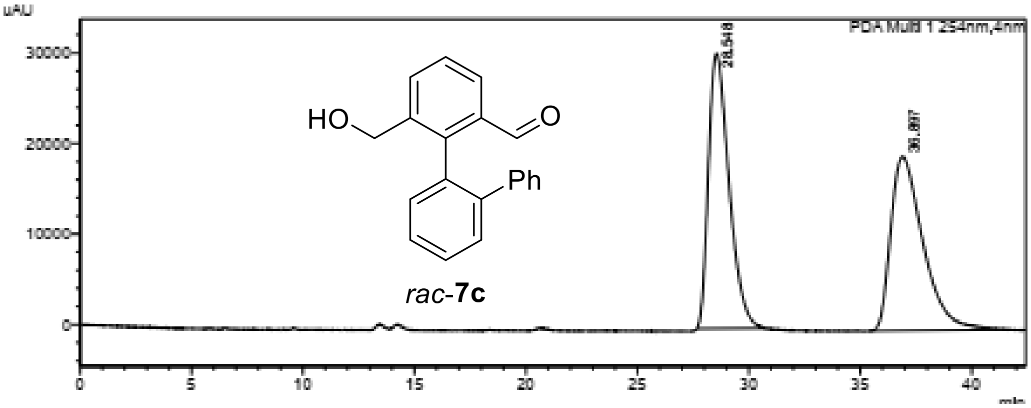

PDA Ch1 254nm

Peak#	Ret. Time	Area	Area%	Height
1	28.548	1959287	50.270	30357
2	36.897	1938225	49.730	19219
Total		3897512	100.000	49576

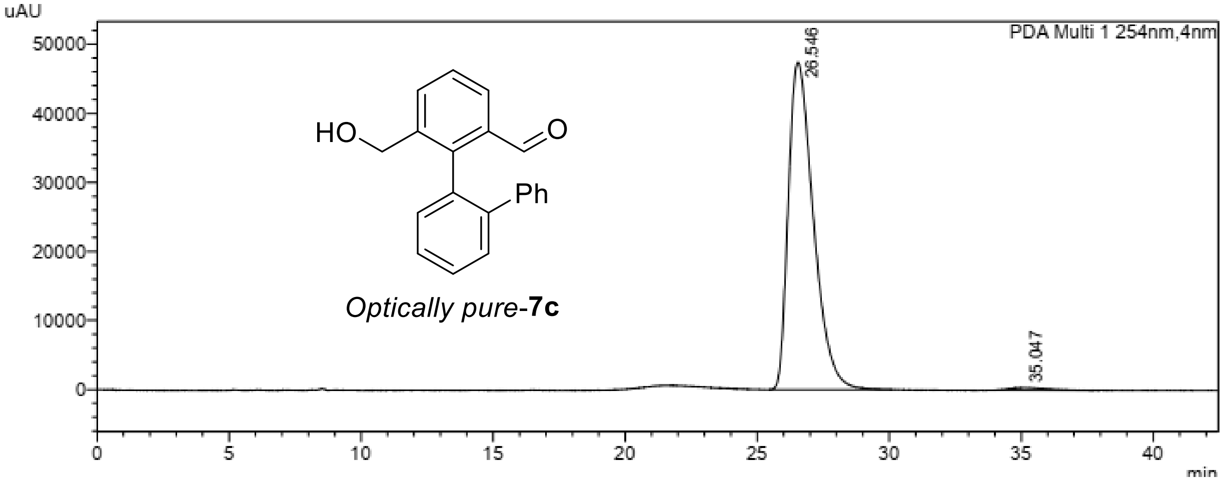

PDA Ch1 254nm

Peak#	Ret. Time	Area	Area%	Height
1	26.546	3137486	98.821	47392
2	35.047	37428	1.179	397
Total		3174913	100.000	47790

7. X-ray structure of (*R*)-2e

<u>Method of crystal growth</u>: In a 20 mL glass vial, 30 mg of the (*R*)-**2e** was dissolved in 0.5 mL of ethyl acetate at RT and slowly added 1 mL of hexane on side walls of the vial. The resulted clear solution was allowed for slow evaporation to obtain suitable crystals for X-ray.

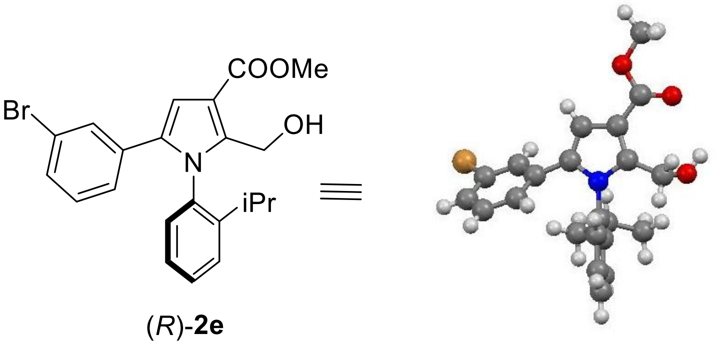

(*R*)-**2e**

Ellipsoids are shown at 50% probability

Datablock: Yoav397b

Bond precision: C-C = 0.0066 A Wavelength=0.71073

Cell:	a=8.378(2)	b=12.451(3)	c=19.560(4)
	alpha=90	beta=90	gamma=90
Temperature:	140 K		

	Calculated	Reported
Volume	2040.4(8)	2040.3(8)
Space group	P 21 21 21	P 21 21 21
Hall group	P 2ac 2ab	P 2ac 2ab
Moiety formula	C22 H22 Br N O3	C22 H22 Br N O3
Sum formula	C22 H22 Br N O3	C22 H22 Br N O3
Mr	428.31	428.31
Dx,g cm-3	1.394	1.394
Z	4	4
Mu (mm-1)	2.036	2.036
F000	880.0	880.0
F000'	879.21	
h,k,lmax	10,14,23	10,14,22
Nref	3641[2093]	3451
Tmin,Tmax	0.659,0.693	0.654,0.691
Tmin'	0.646	

Correction method= # Reported T Limits: Tmin=0.654 Tmax=0.691
AbsCorr = MULTI-SCAN

Data completeness= 1.65/0.95 Theta(max)= 25.130

R(reflections)= 0.0361(2293) wR2(reflections)= 0.0781(3451)

S = 0.966 Npar= 228

8.References:

1. Tnay, Y. L.; Chen, C.; Chua, Y. Y.; Zhang, L.; Chiba, S. *Org. Lett*. **2012**, *14*, 3550-3553.
2. Staniland, S.; Yuan, B.; Gimenez-Agullo, N.; Marcelli, T.; Willies, S. C.; Grainger, D. M.; Turner, N. J.; Clayden, J. *Chem. - Eur. J.* **2014**, *20*, 13084−13088.
3. Carbó López, M.; Royal, G.; Philouze, C.; Chavant, P. Y.; Blandin, V. *Eur. J. Org. Chem.* **2014**, *2014,* 4884−4896.

9. ^{1}H and ^{13}C NMR Spectra of Products

^{1}H and $^{13}C\{^{1}H\}$ NMR spectra of **S8**

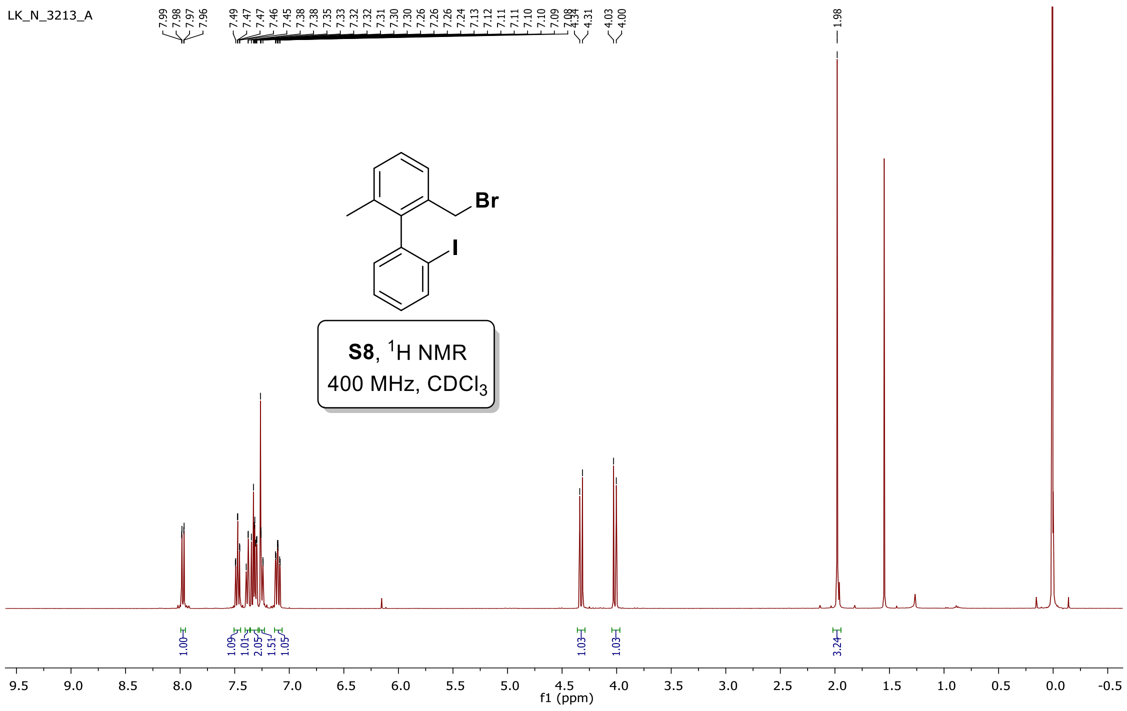

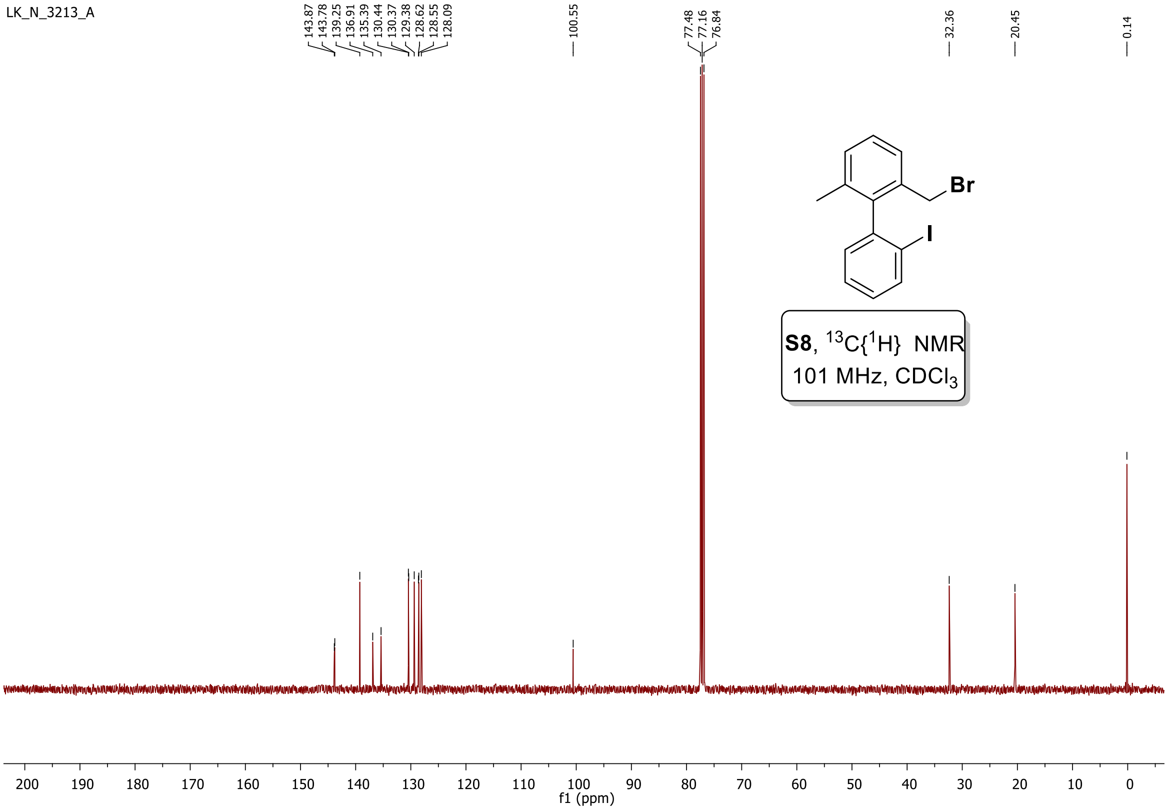

^1H and $^{13}C\{^1H\}$ NMR spectra of **S11**

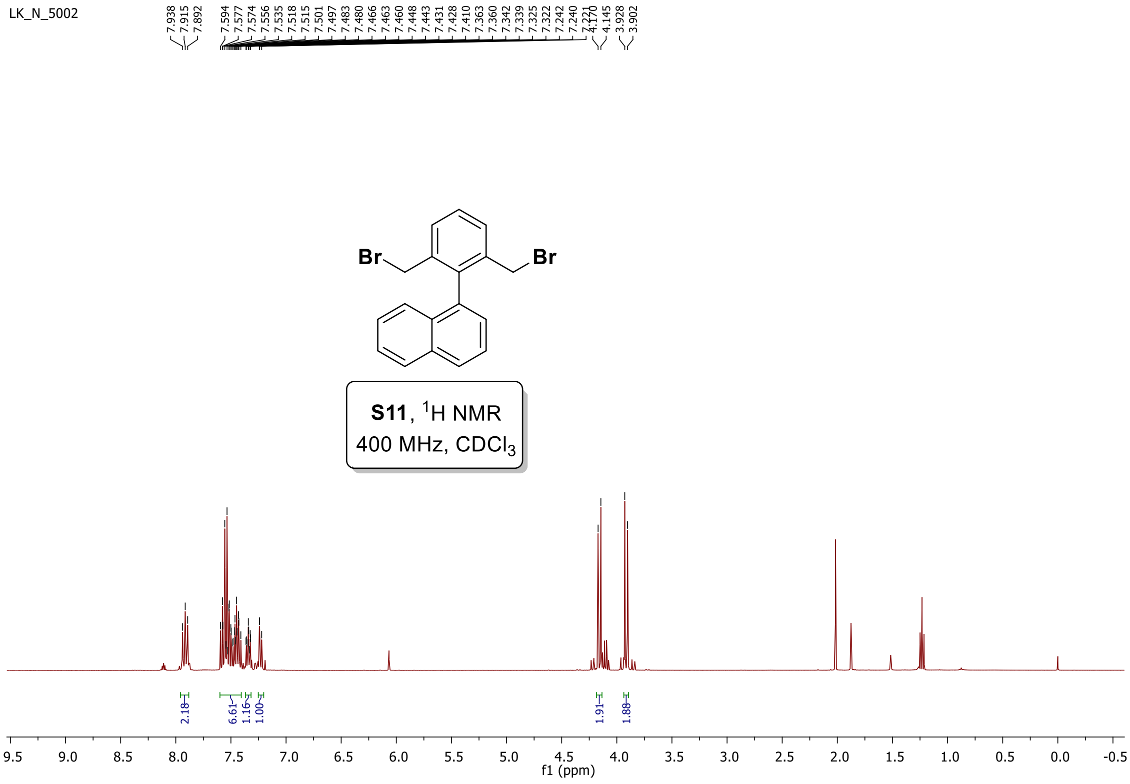

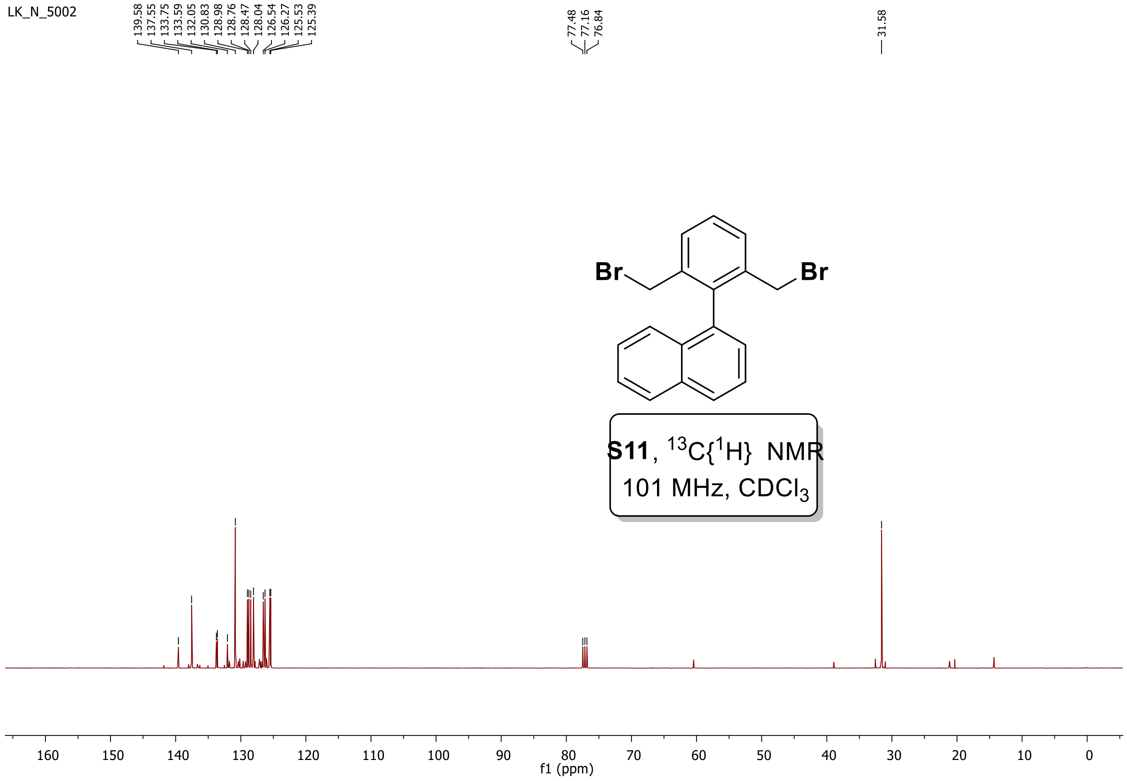

^{1}H and $^{13}C\{^{1}H\}$ NMR spectra of **S15**

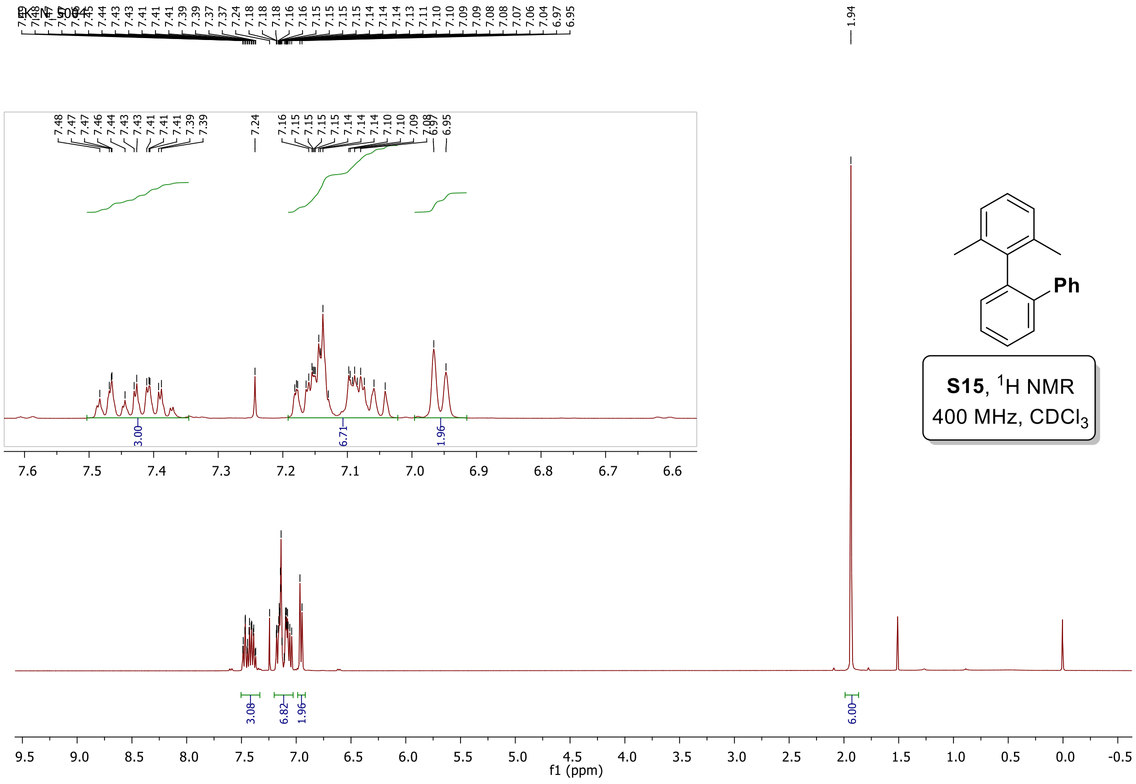

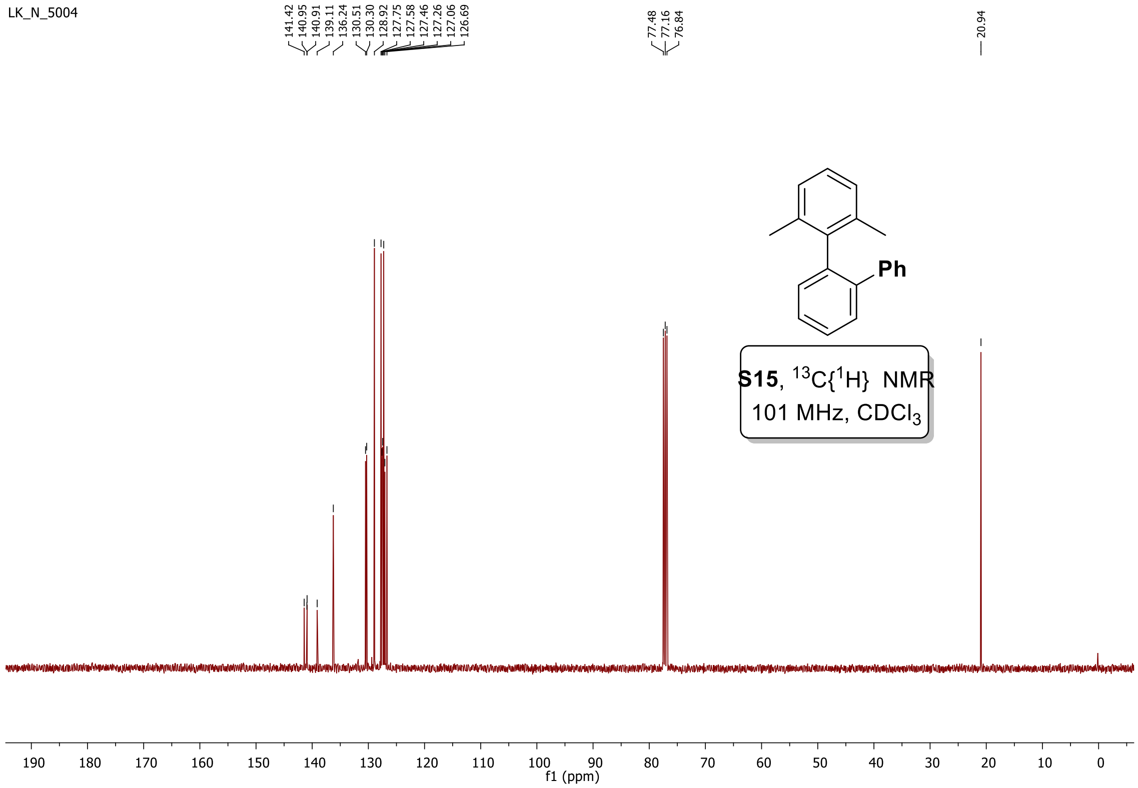

^{1}H and $^{13}C\{^{1}H\}$ NMR spectra of **6a**

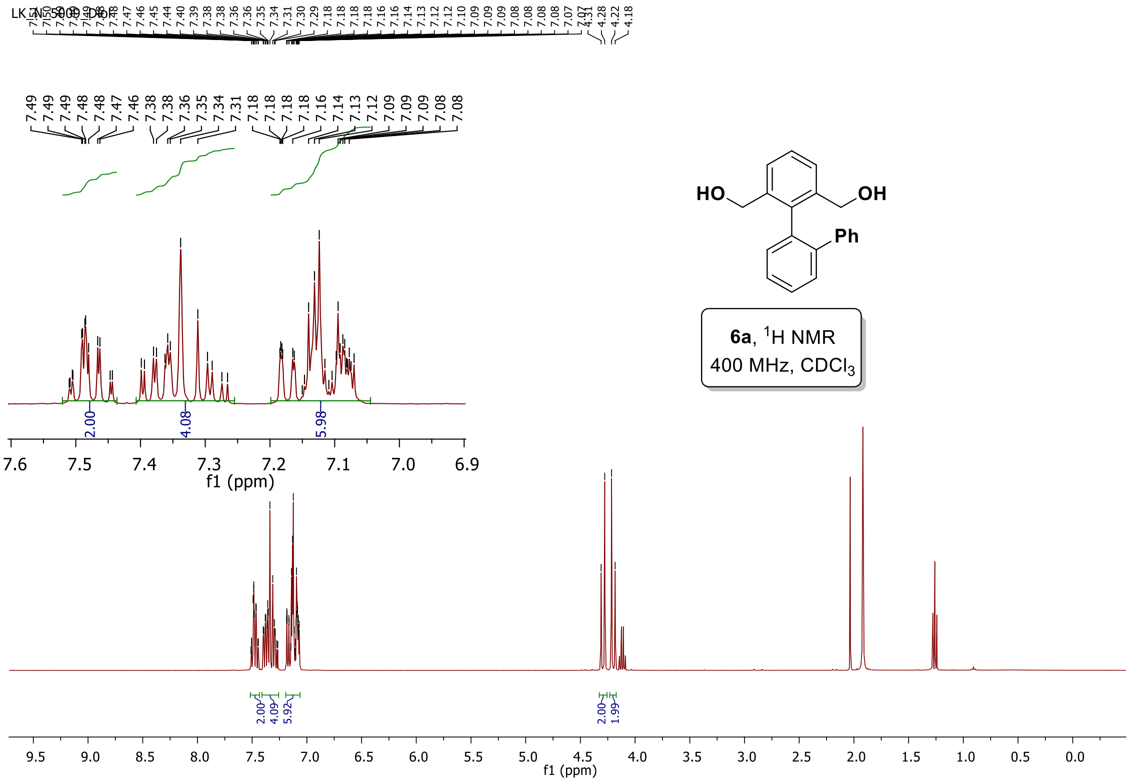

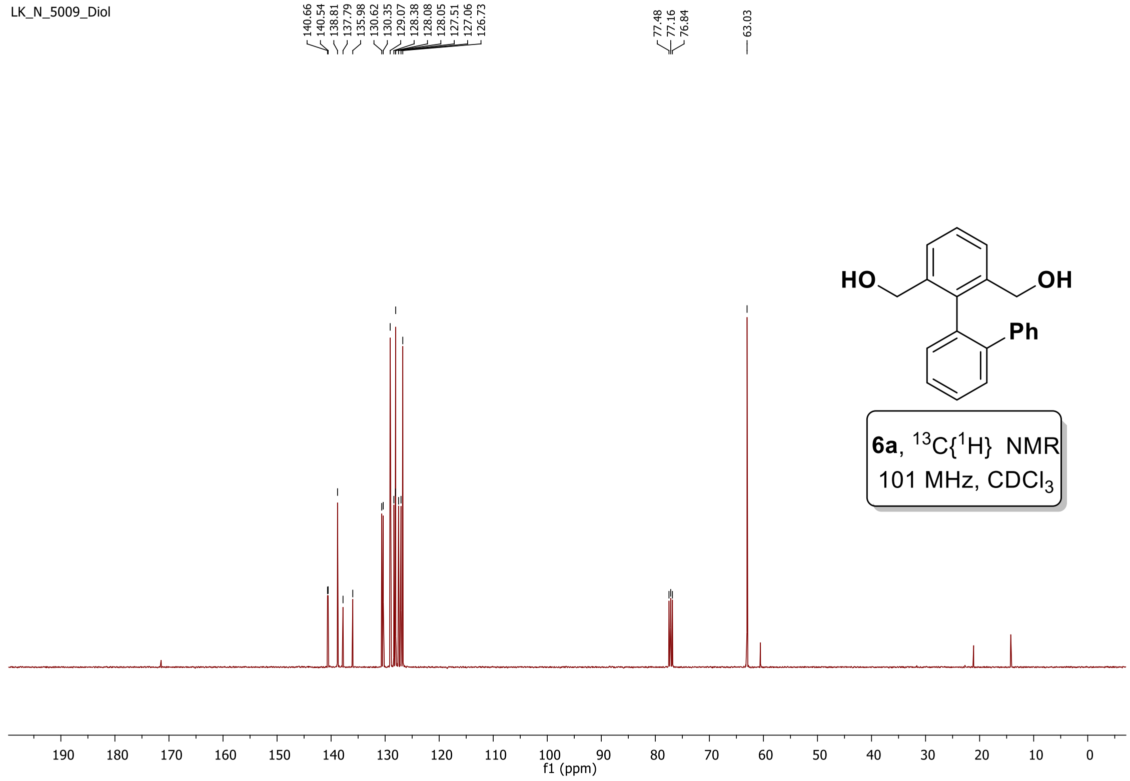

^{1}H and $^{13}C\{^{1}H\}$ NMR spectra of (*R*)-**2a**

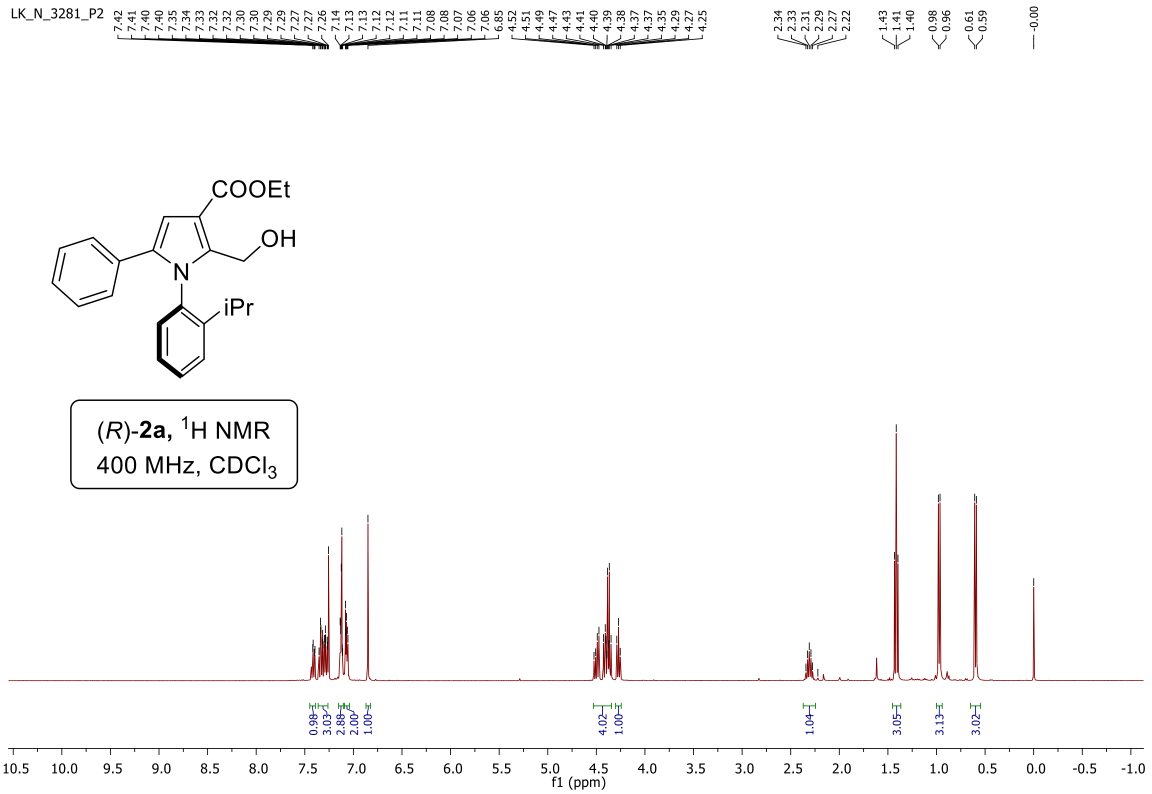

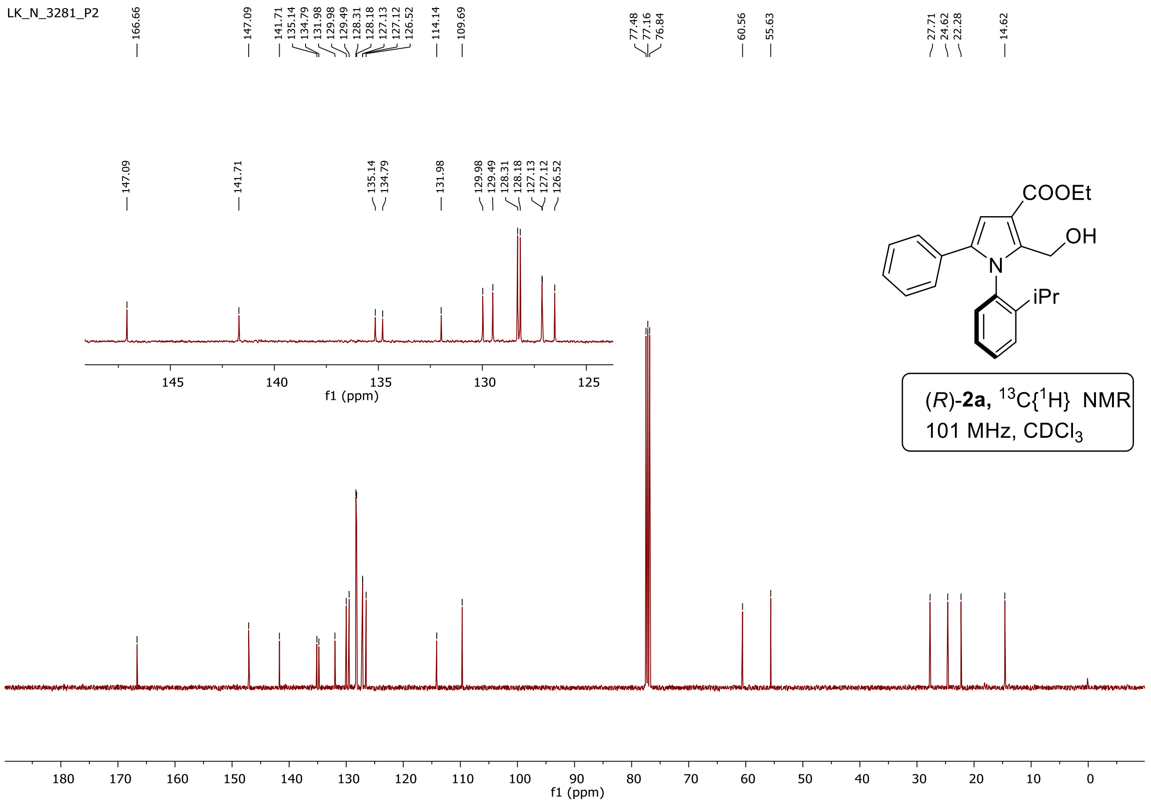

^{1}H and $^{13}C\{^{1}H\}$ NMR spectra of (*S*)-**3a**

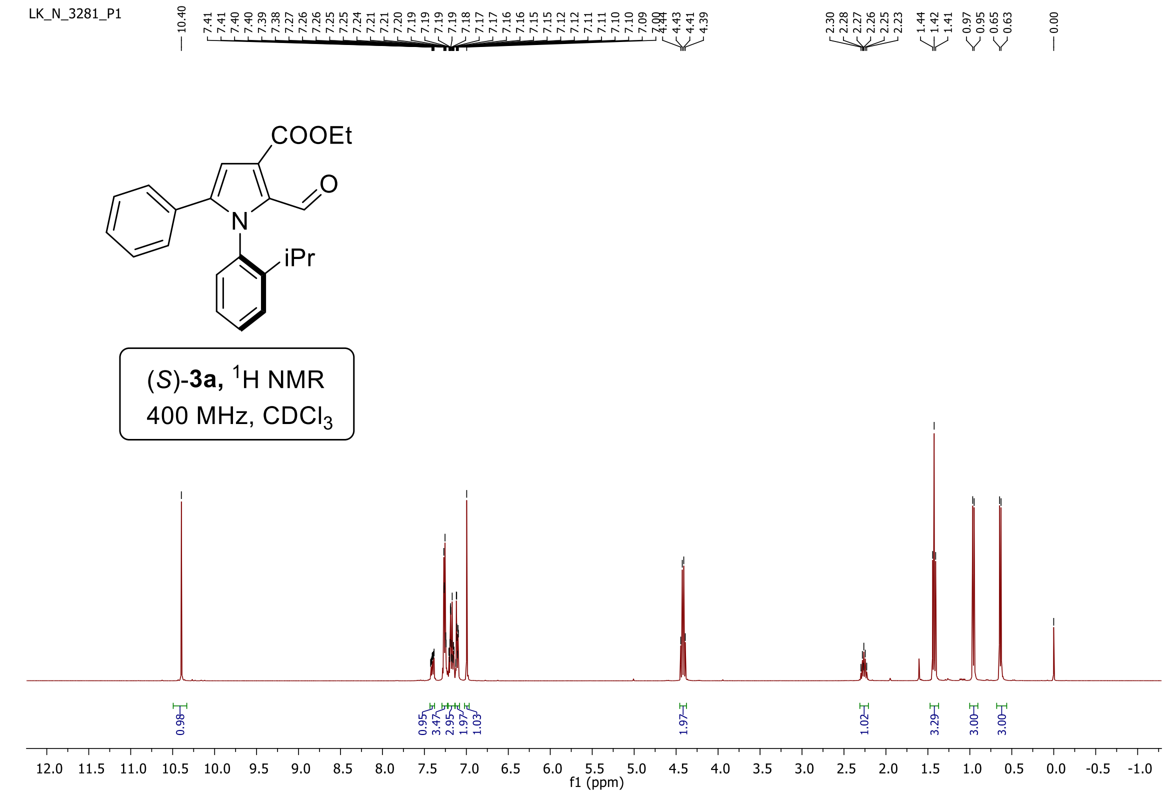

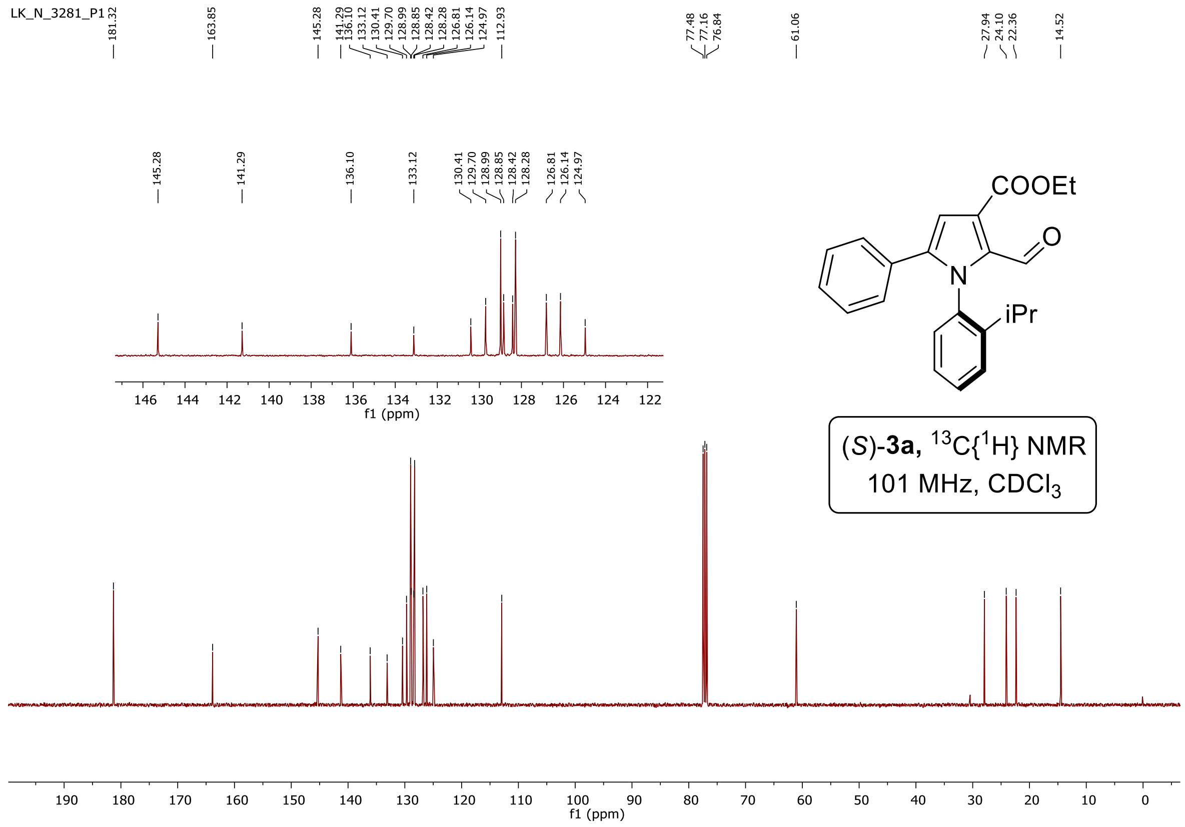

^{1}H and $^{13}C\{^{1}H\}$ NMR spectra of (*R*)-**2b**

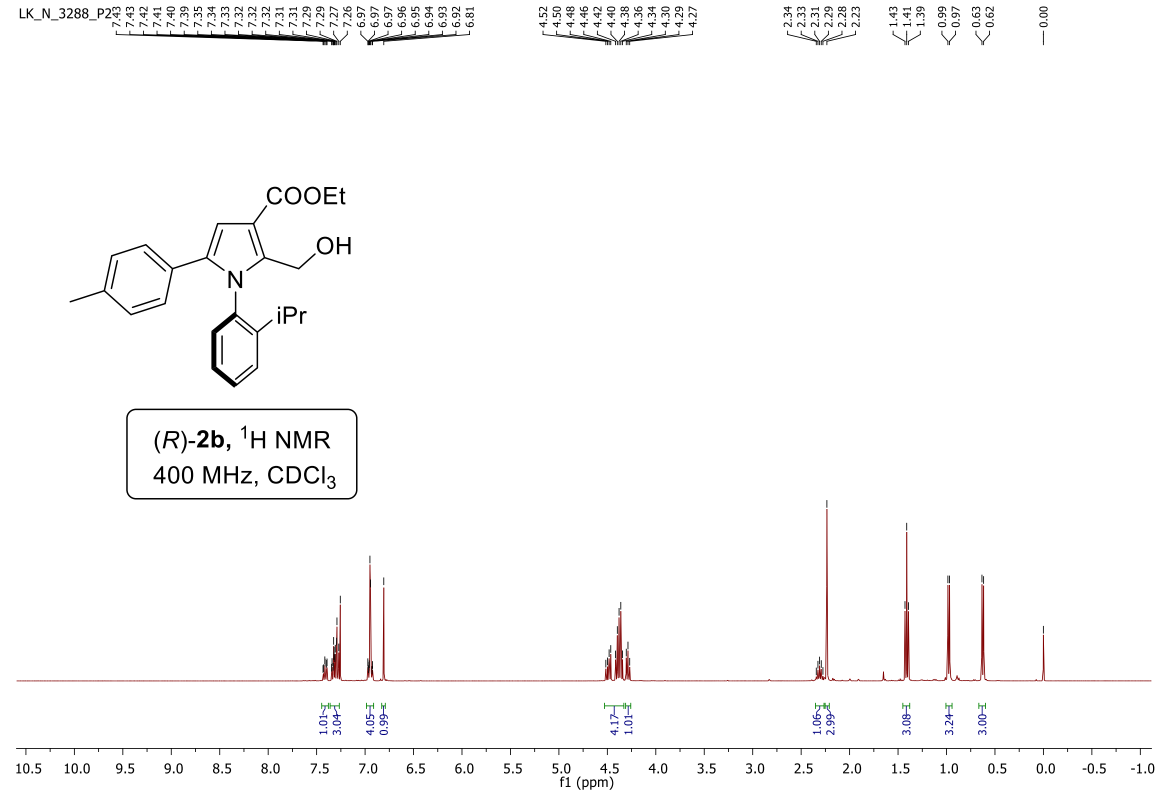

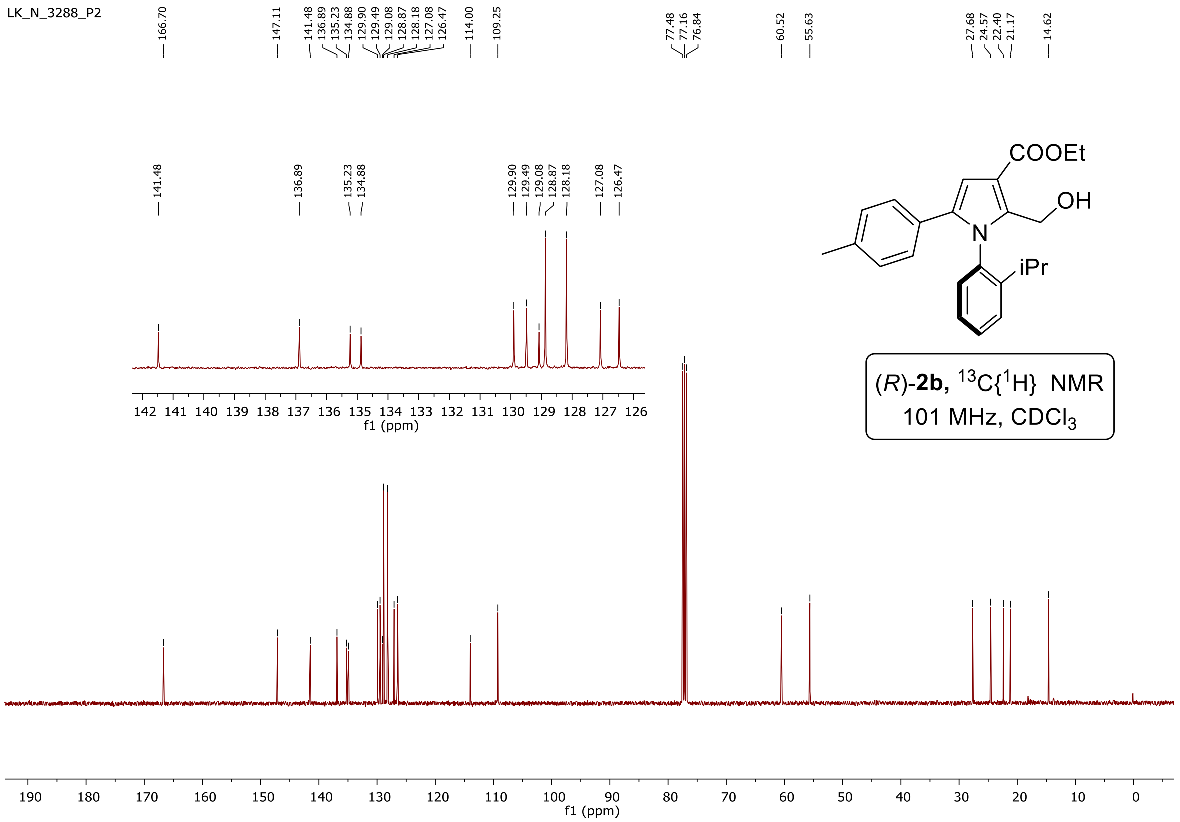

^{1}H and $^{13}C\{^{1}H\}$ NMR spectra of (*S*)-**3b**

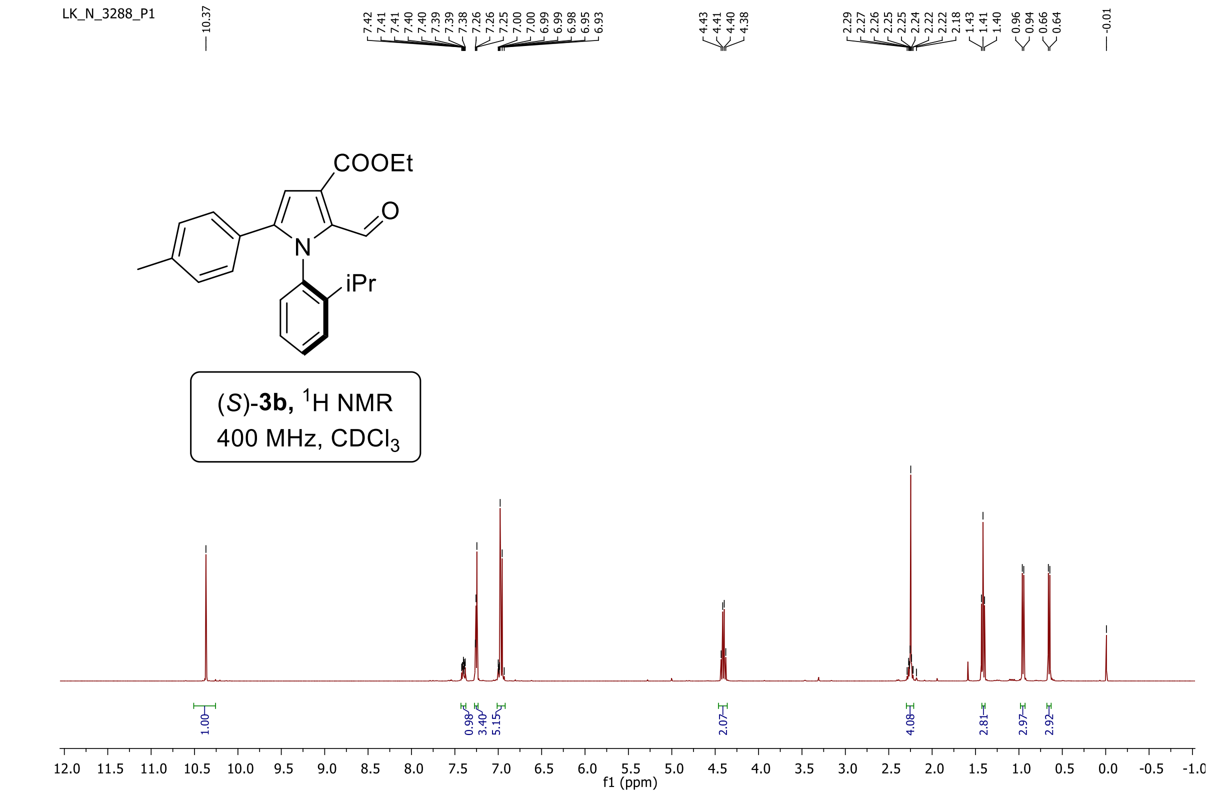

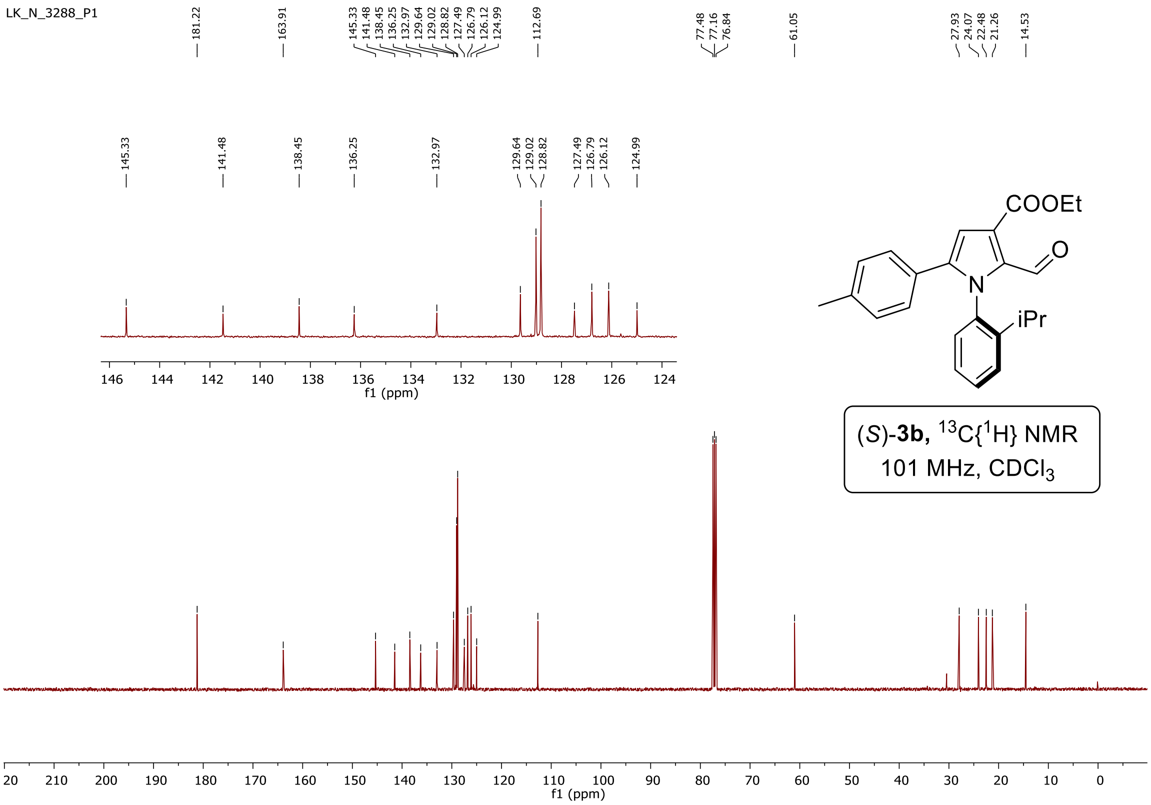

^{1}H and $^{13}C\{^{1}H\}$ NMR spectra of (*R*)-**2c**

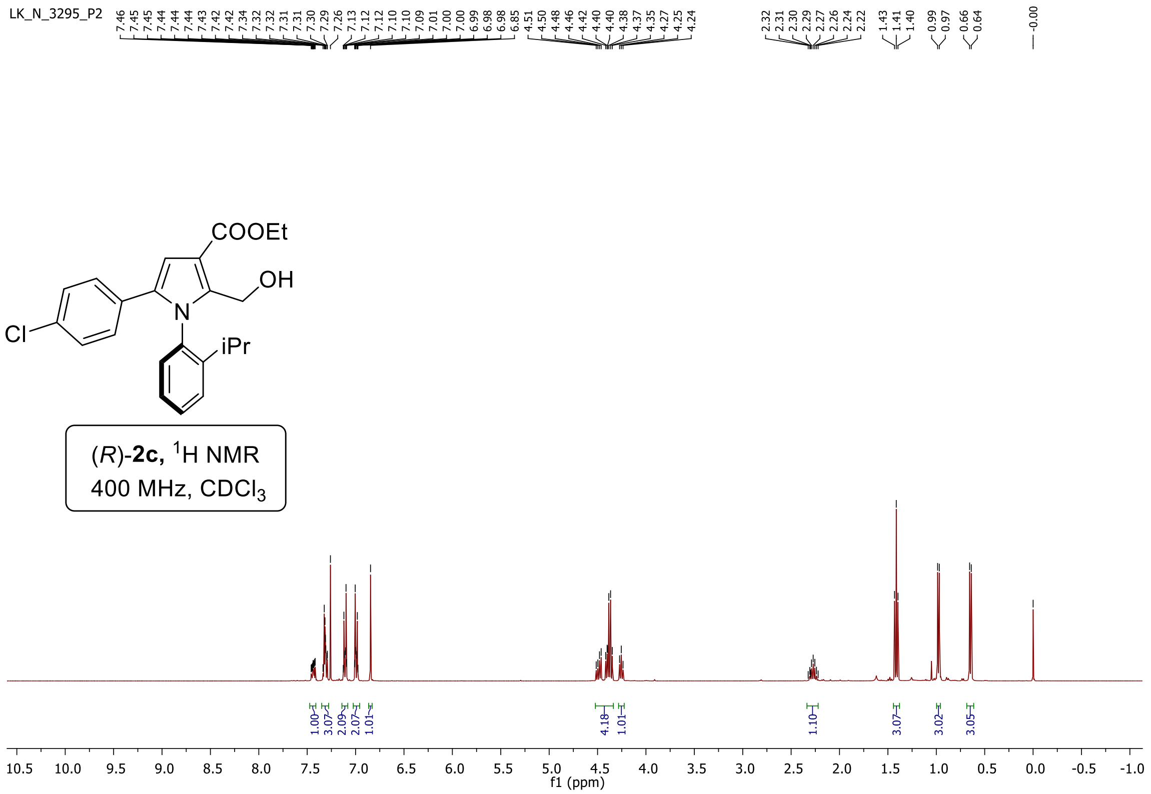

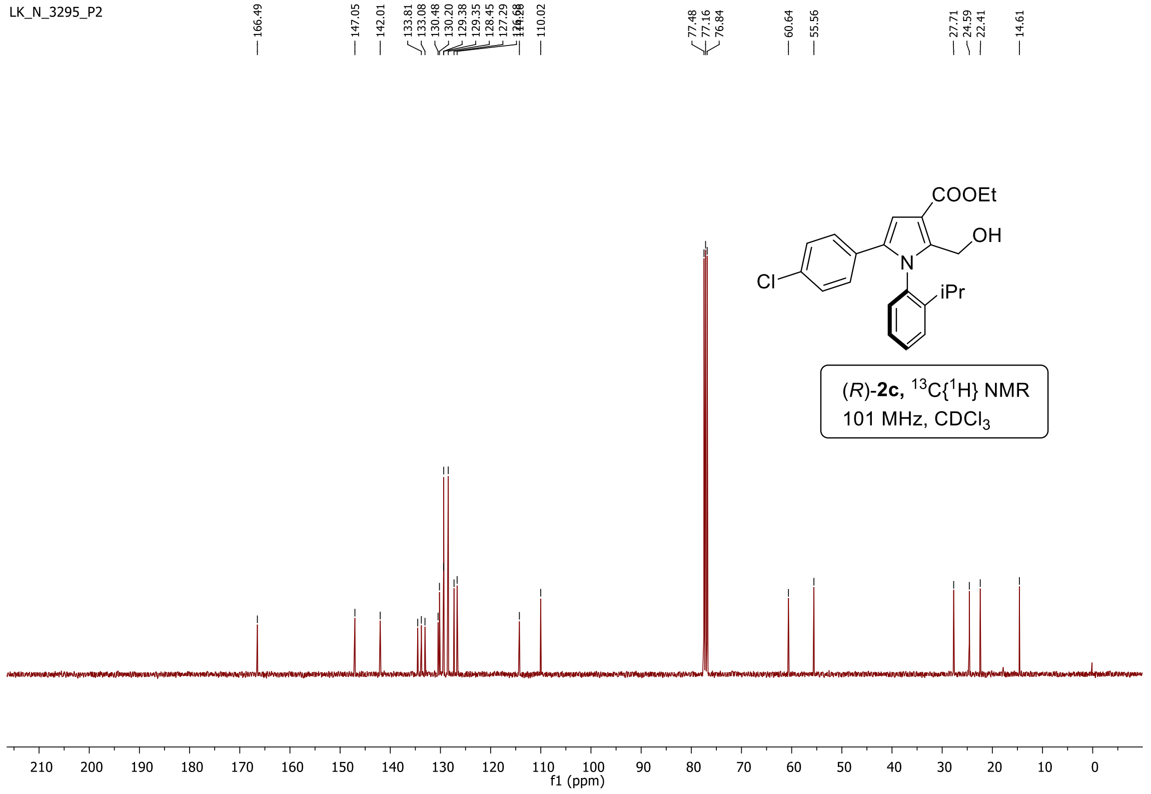

^{1}H and ^{13}C{^{1}H} NMR spectra of (*S*)-**3c**

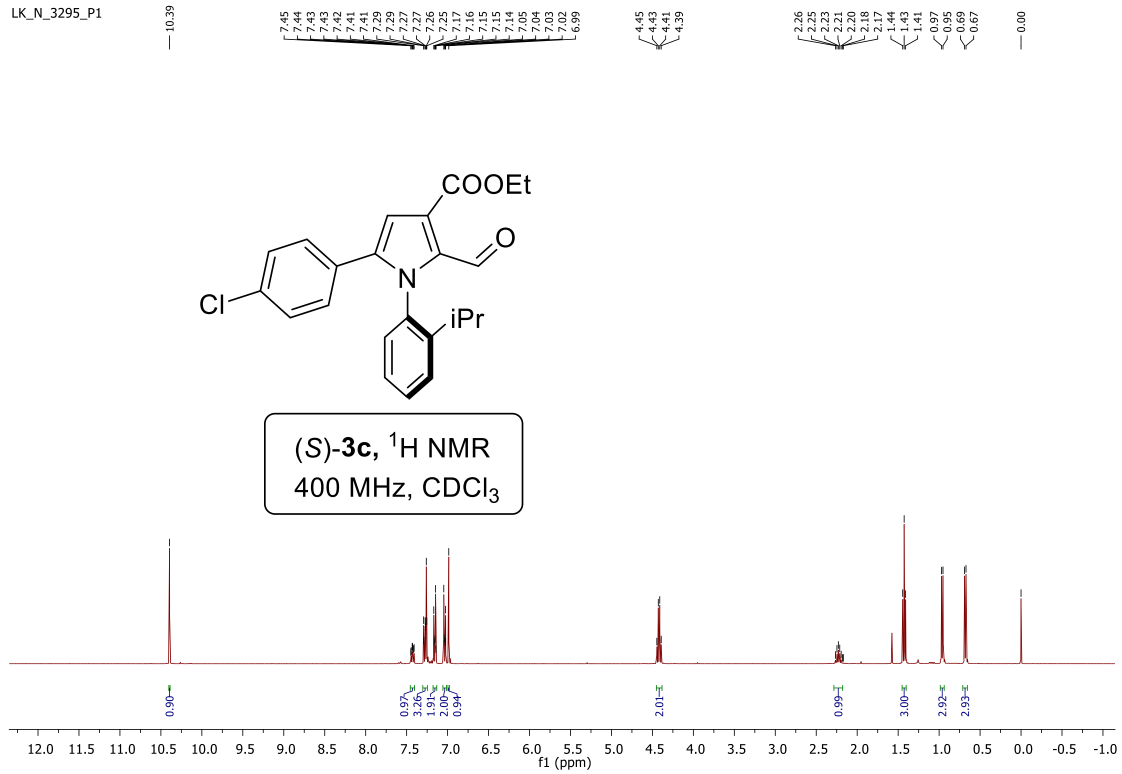

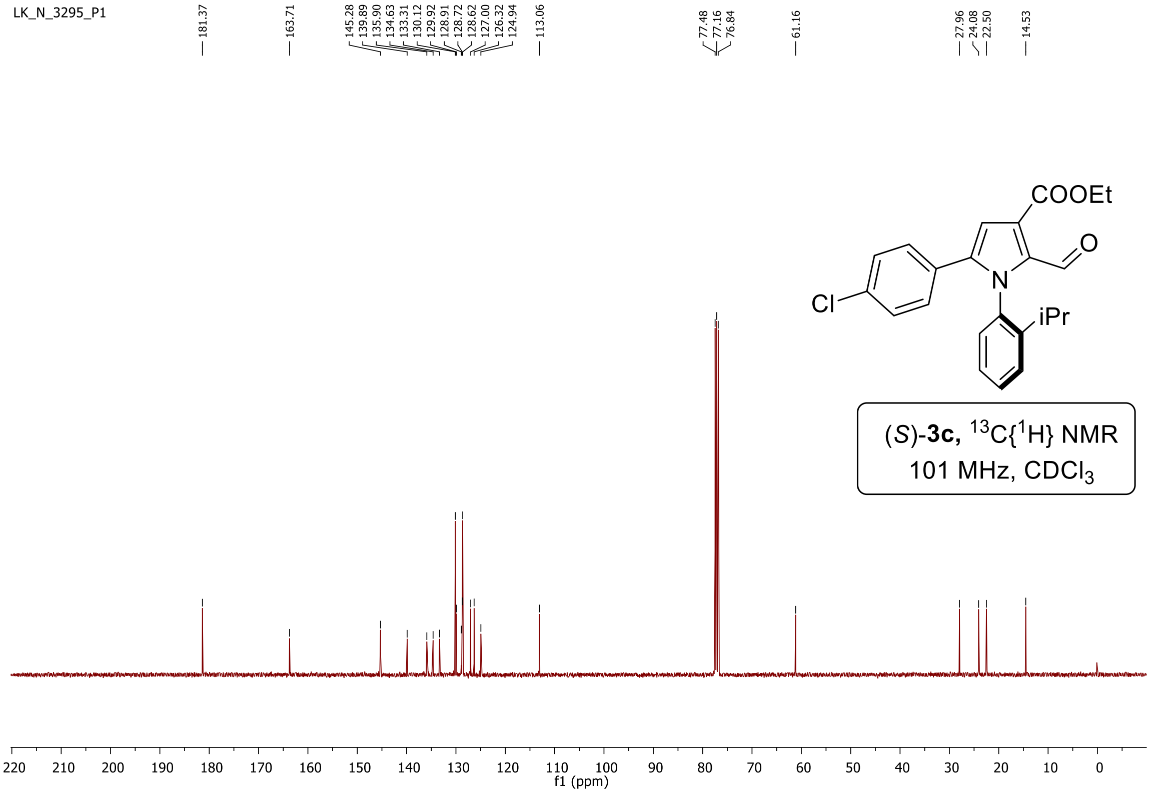

^{1}H and $^{13}C\{^{1}H\}$ NMR spectra of (*R*)-**2d**

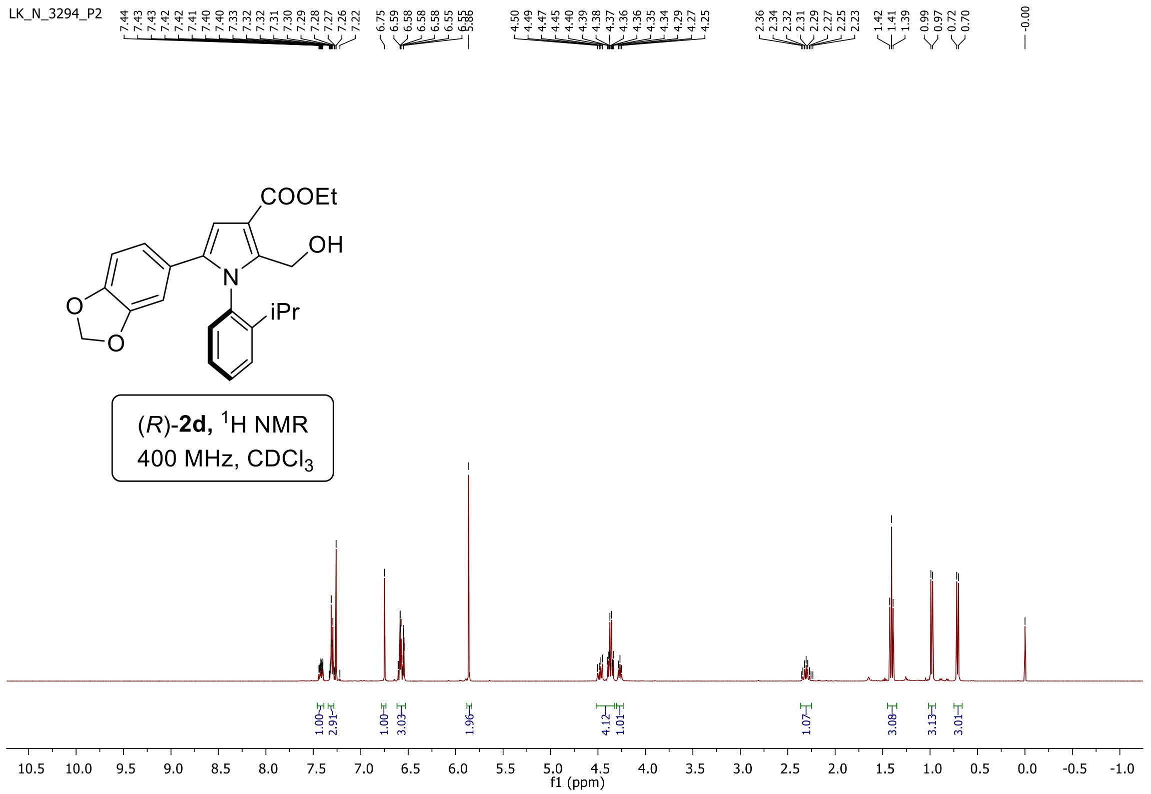

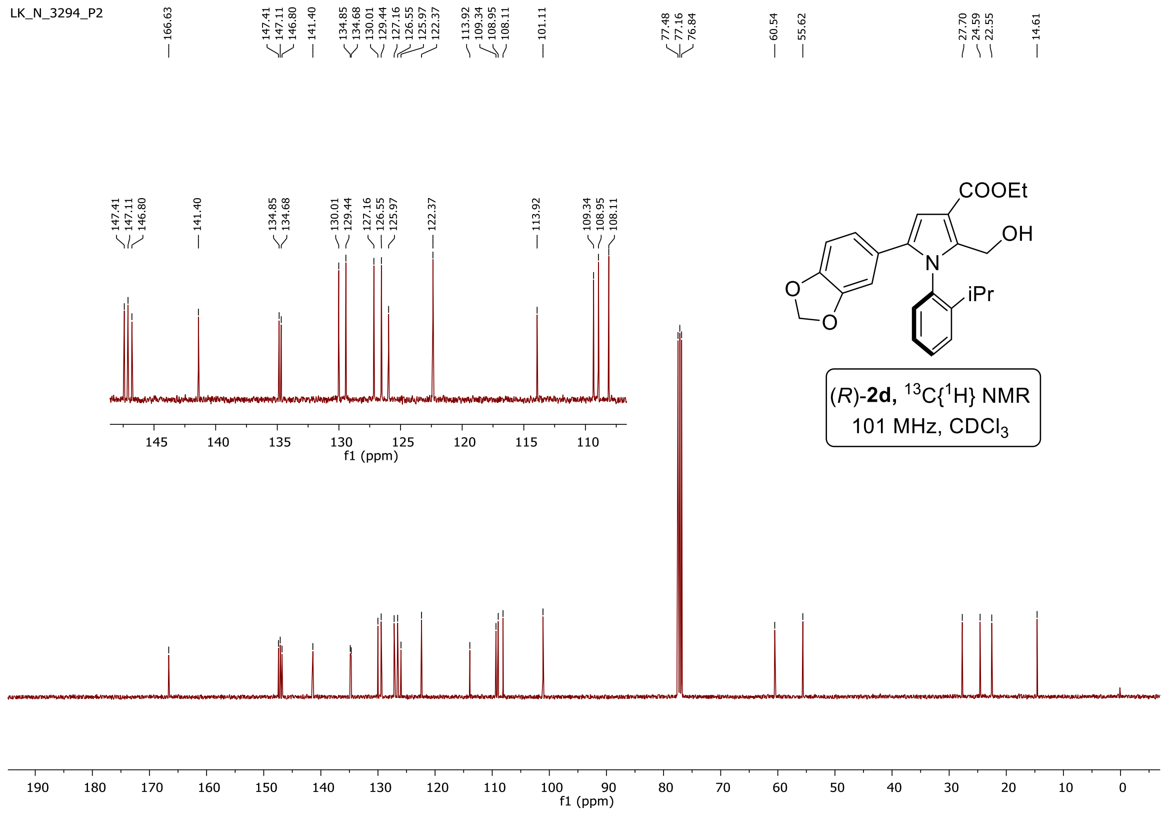

^1H and $^{13}C\{^1H\}$ NMR spectra of (S)-**3d**

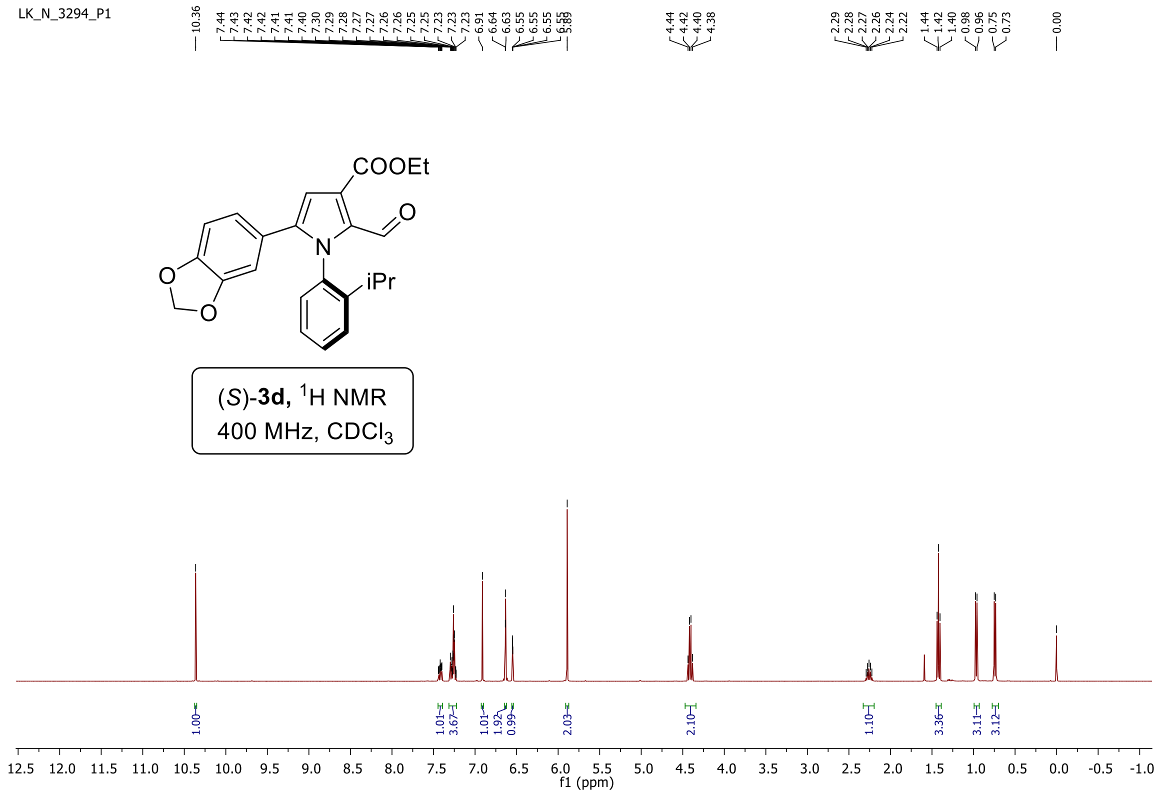

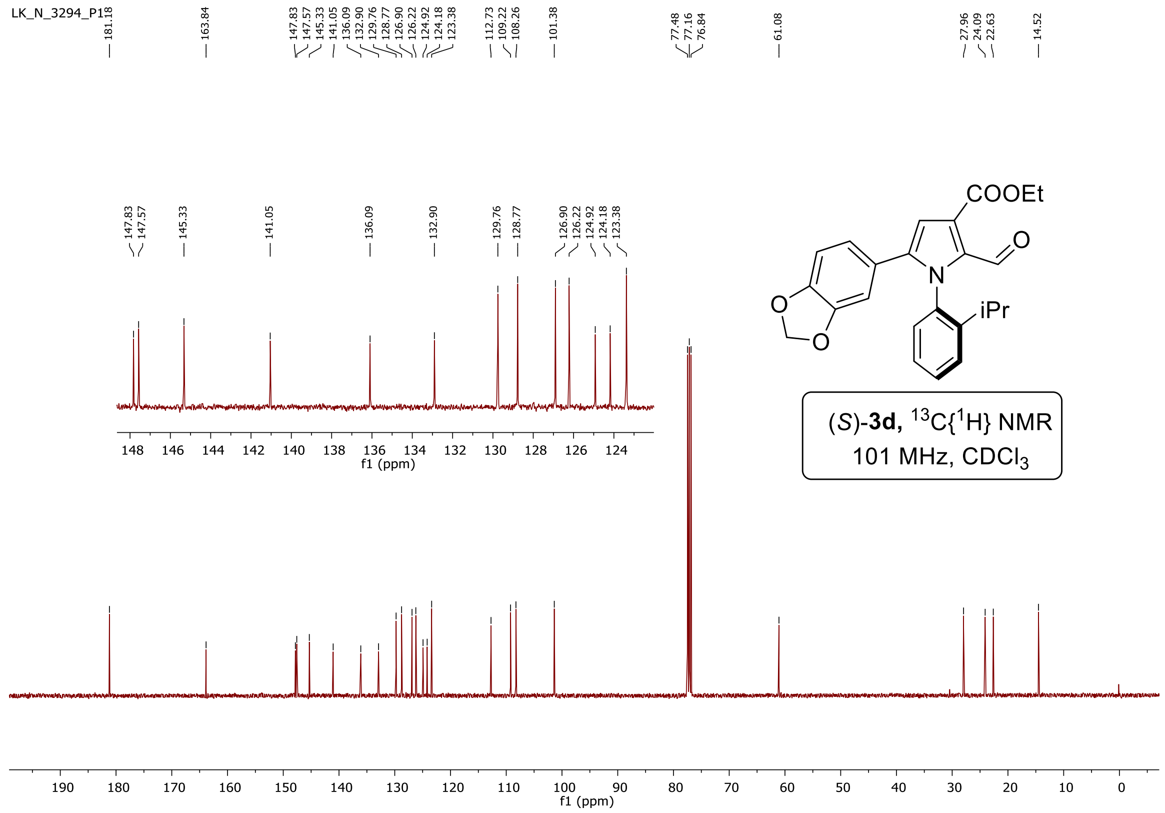

^{1}H and $^{13}C\{^{1}H\}$ NMR spectra of (*R*)-**2e**

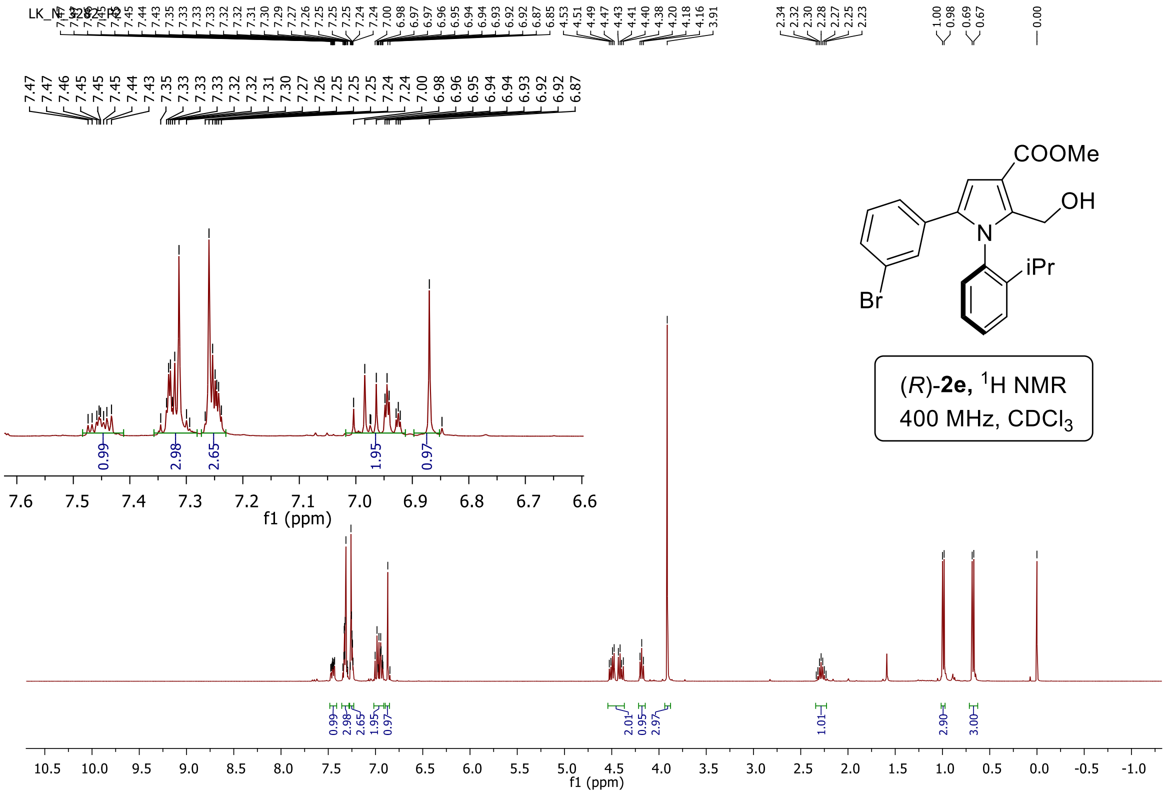

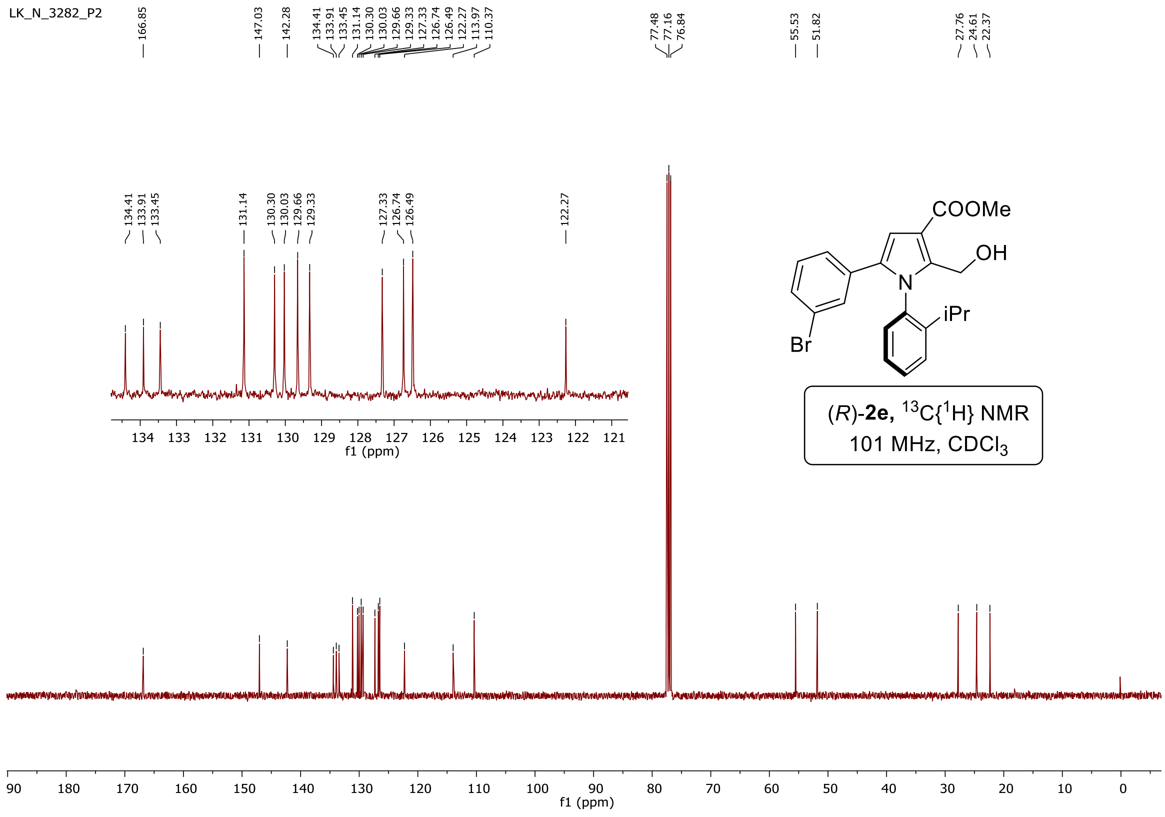

^{1}H and $^{13}C\{^{1}H\}$ NMR spectra of (*S*)-**3e**

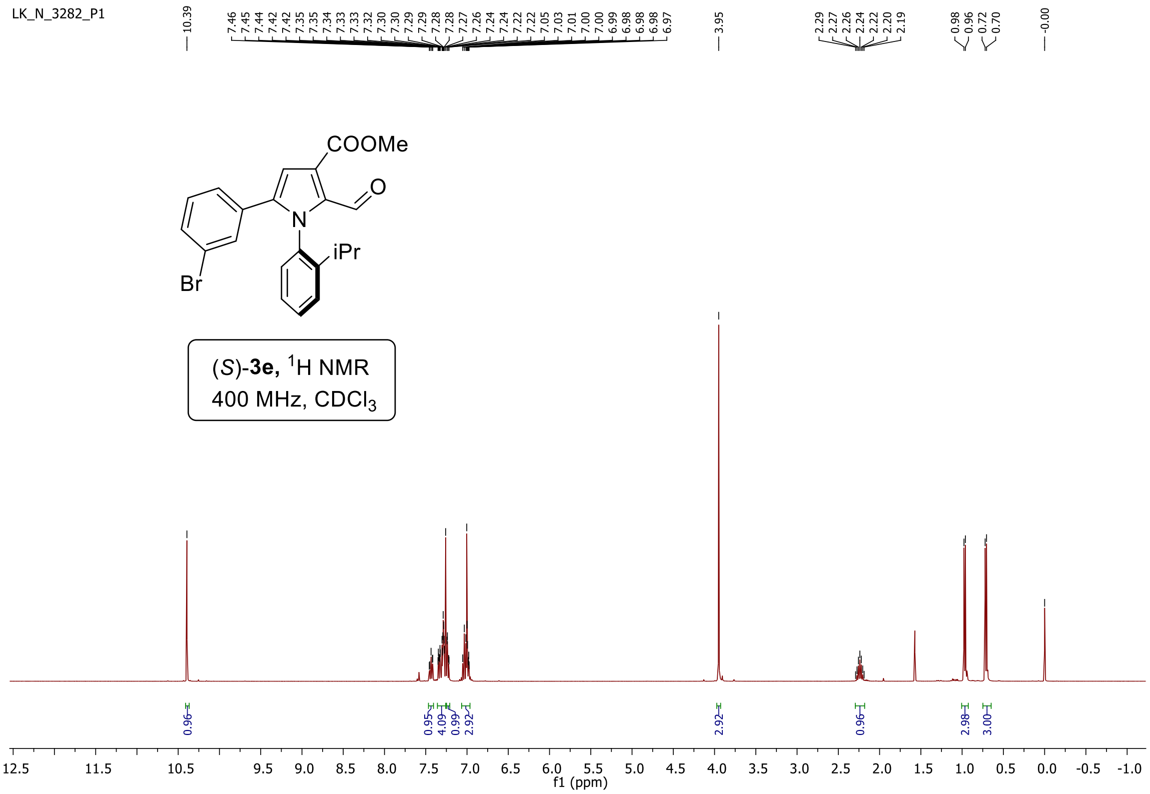

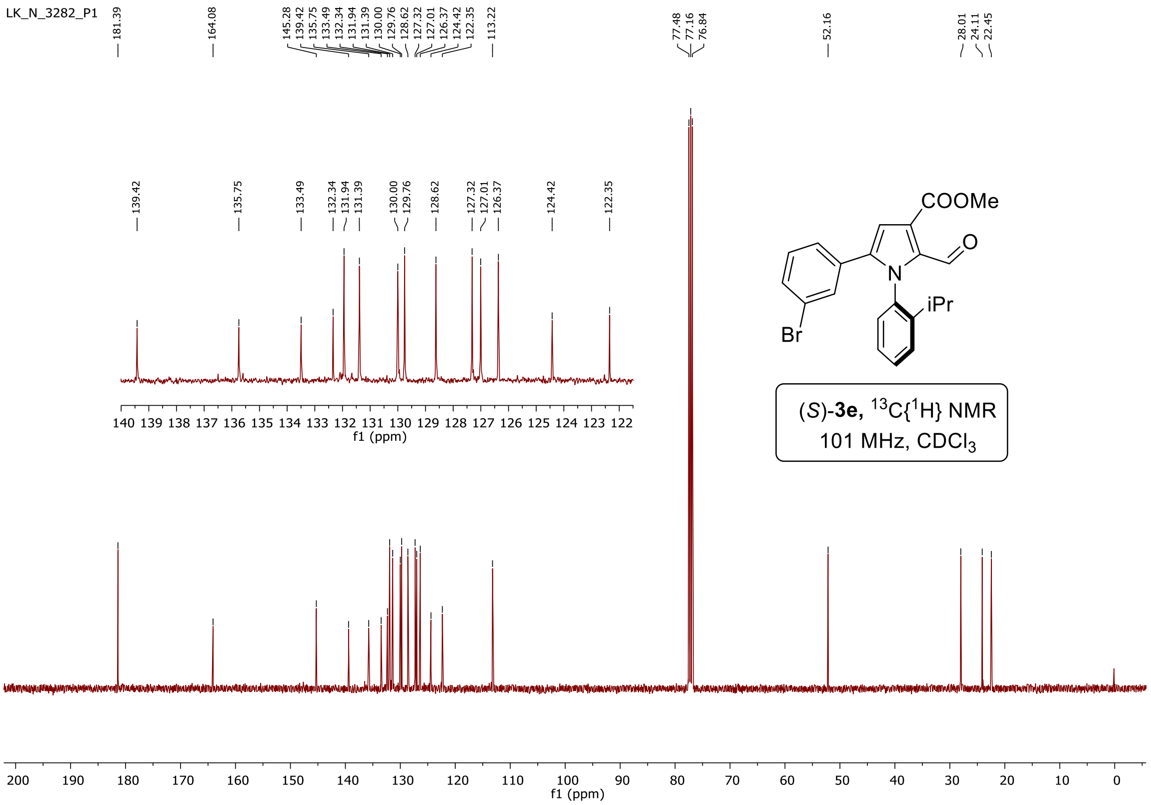

^{1}H and $^{13}C\{^{1}H\}$ NMR spectra of (*R*)-**2f**

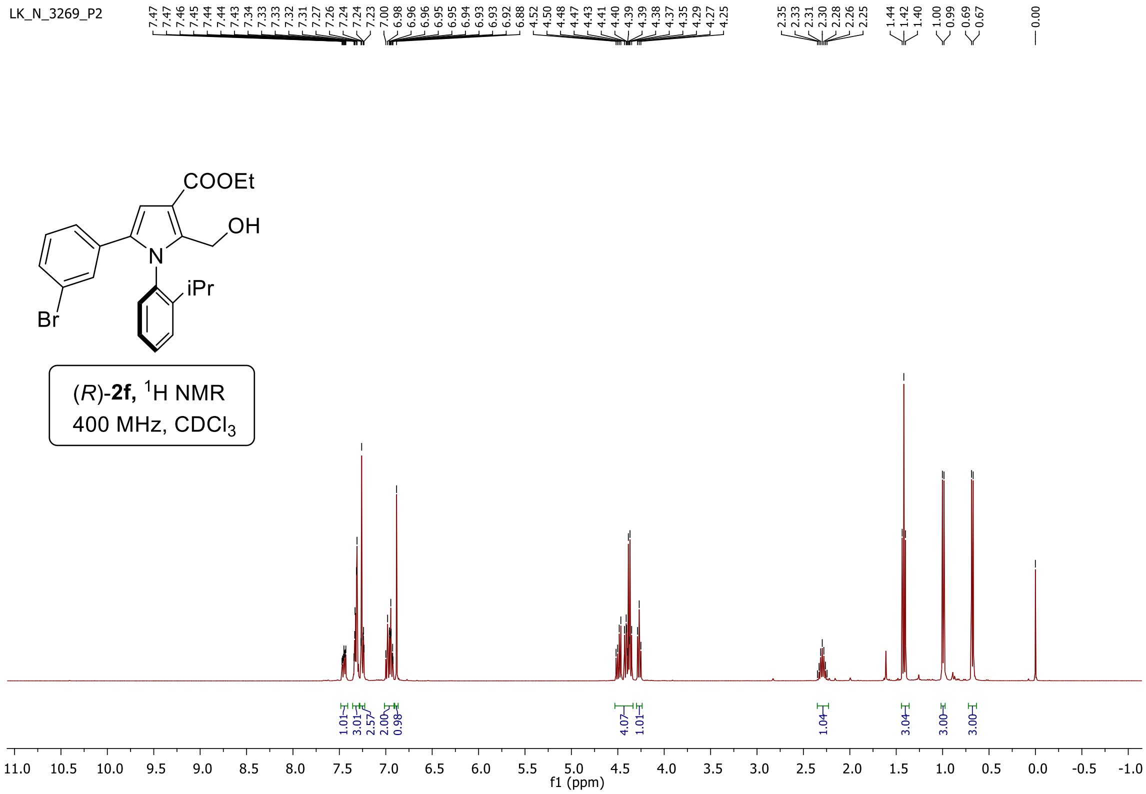

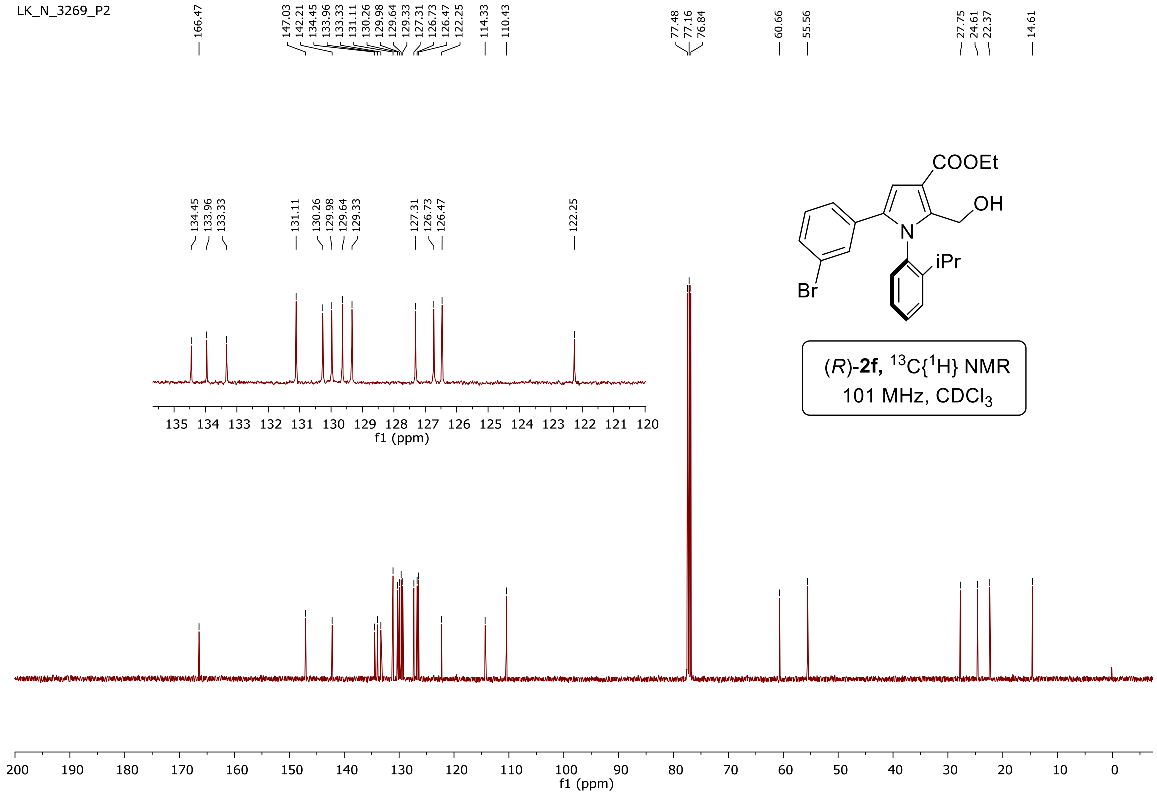

^{1}H and ^{13}C{^{1}H} NMR spectra of (*S*)-**3f**

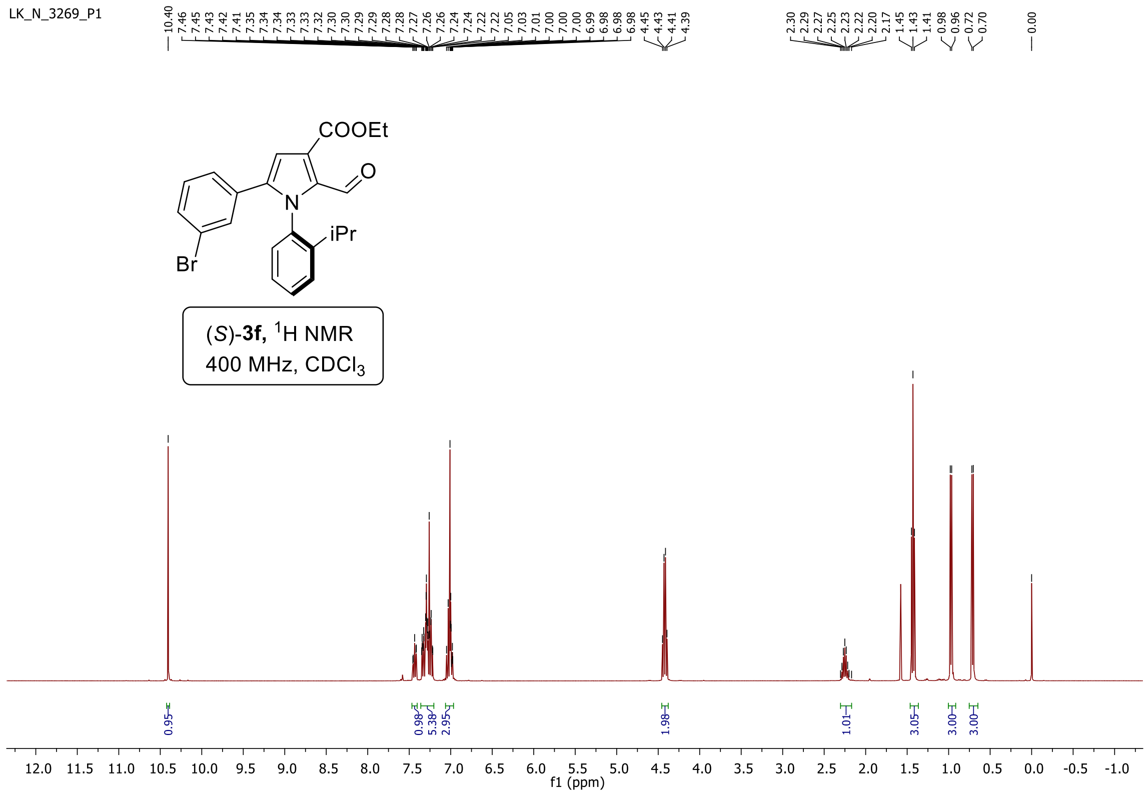

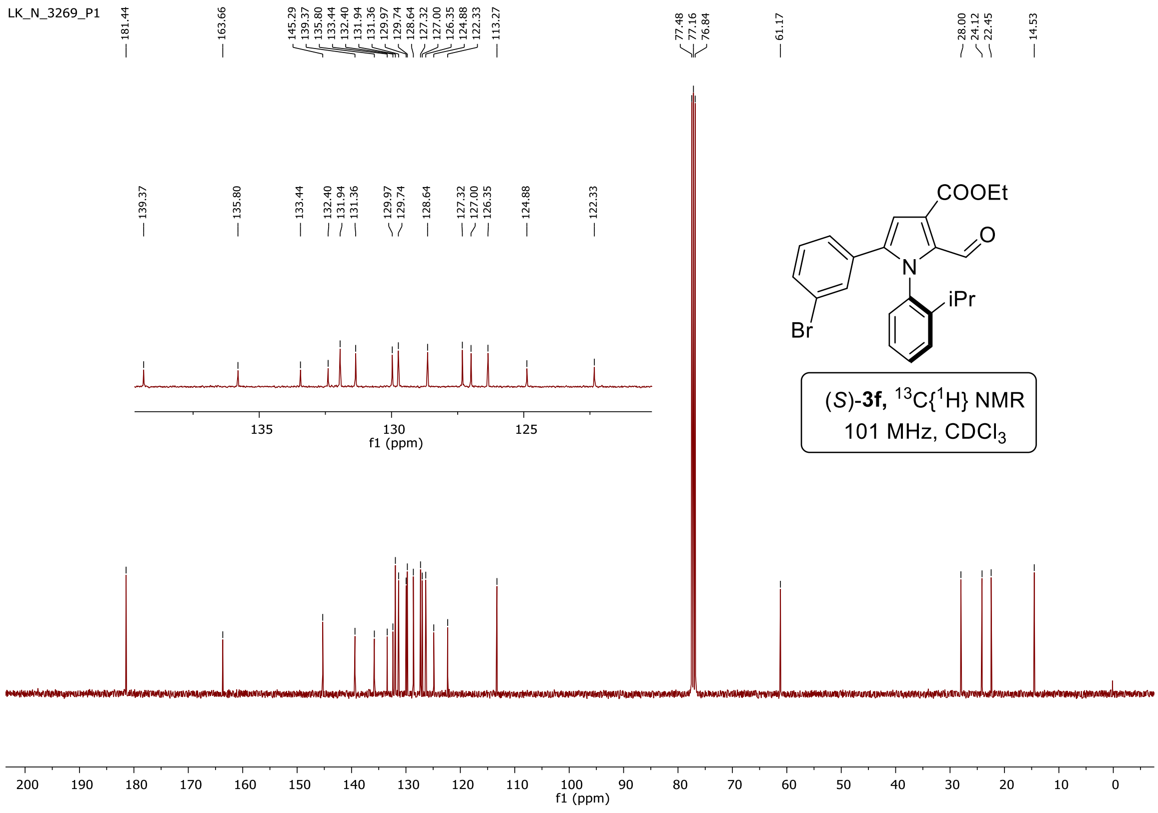

^{1}H and $^{13}C\{^{1}H\}$ NMR spectra of (*R*)-**2g**

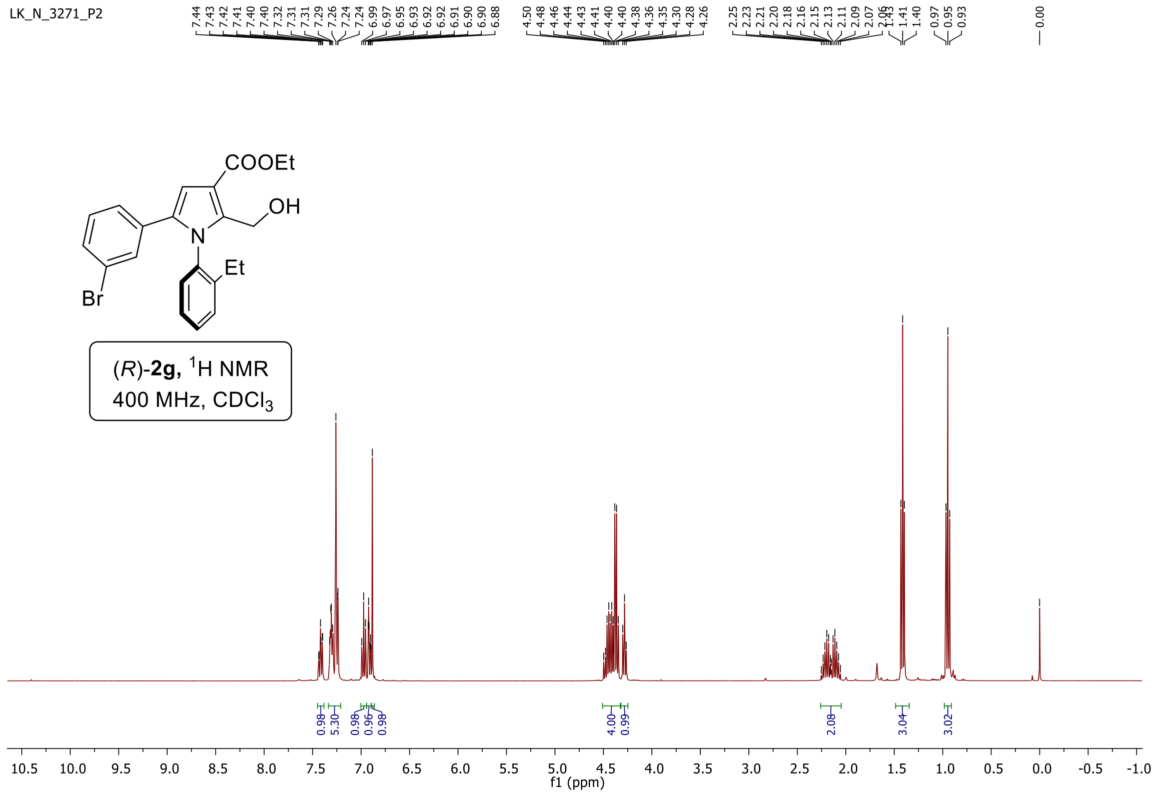

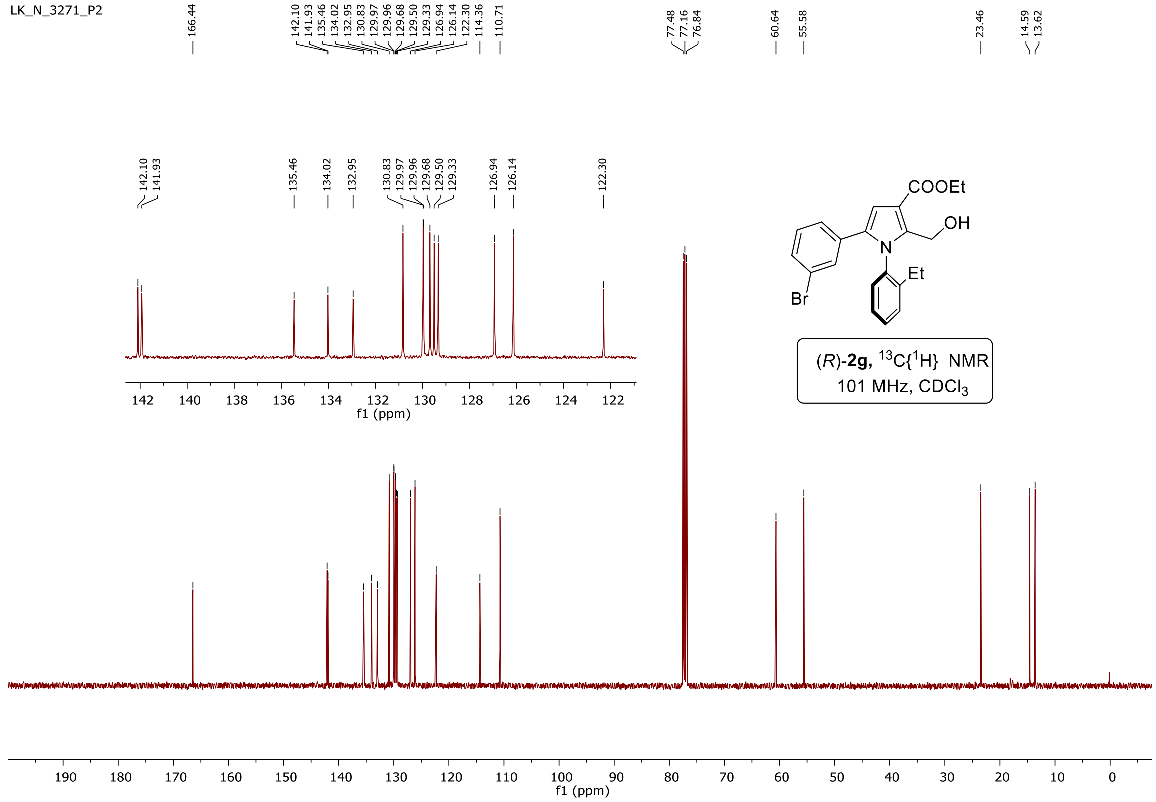

^{1}H and $^{13}C\{^{1}H\}$ NMR spectra of (*S*)-**3g**

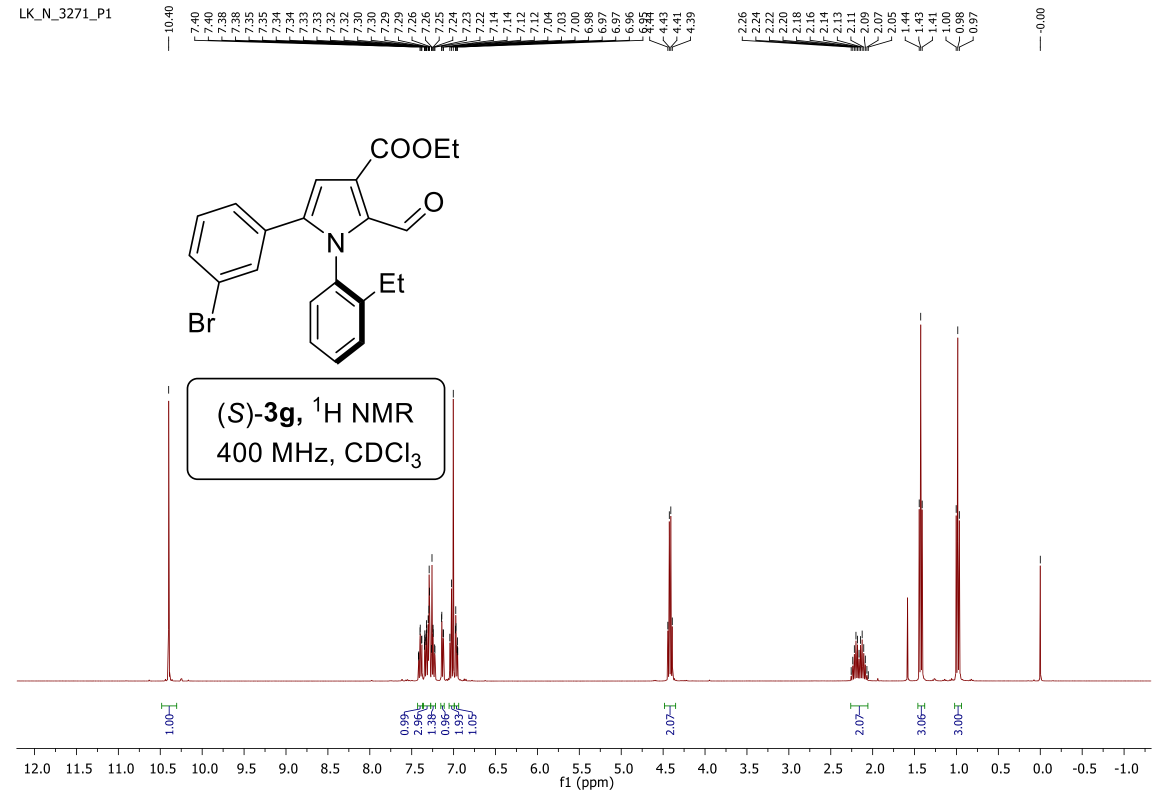

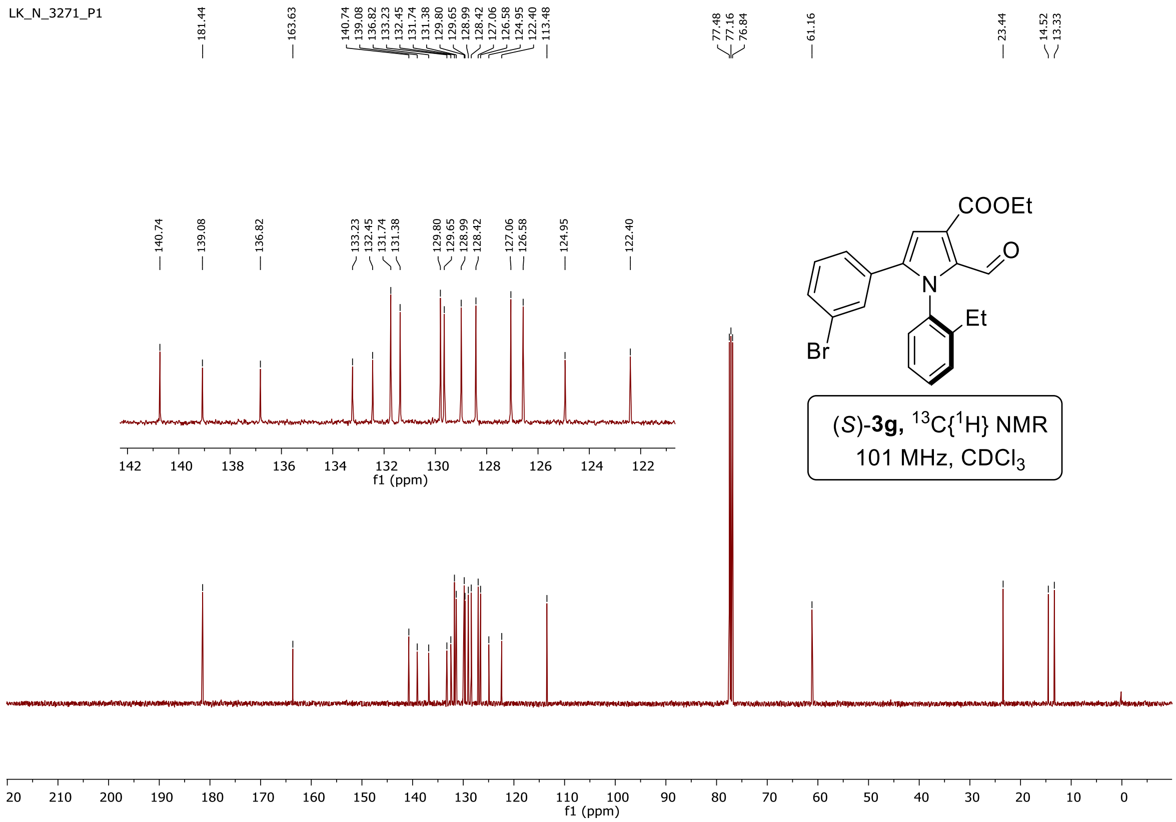

^{1}H and $^{13}C\{^{1}H\}$ NMR spectra of (*R*)-**2h**

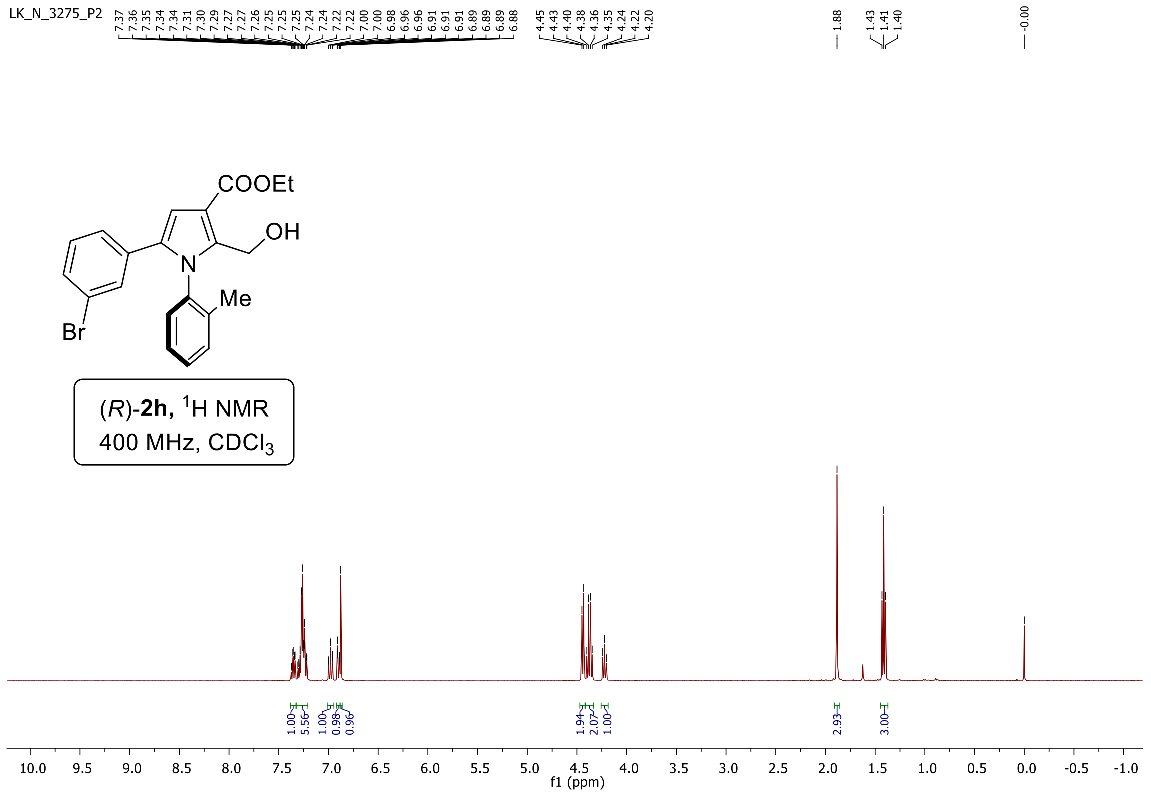

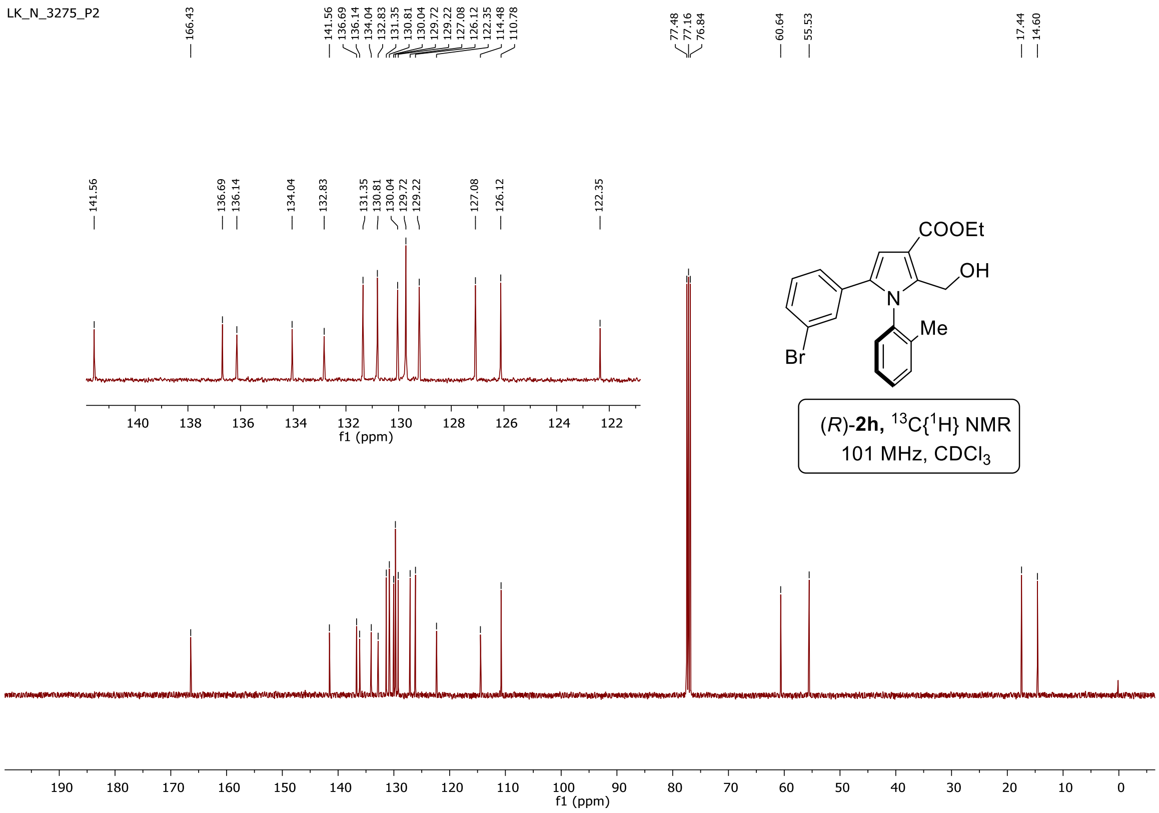

^{1}H and $^{13}C\{^{1}H\}$ NMR spectra of (*S*)-**3h**

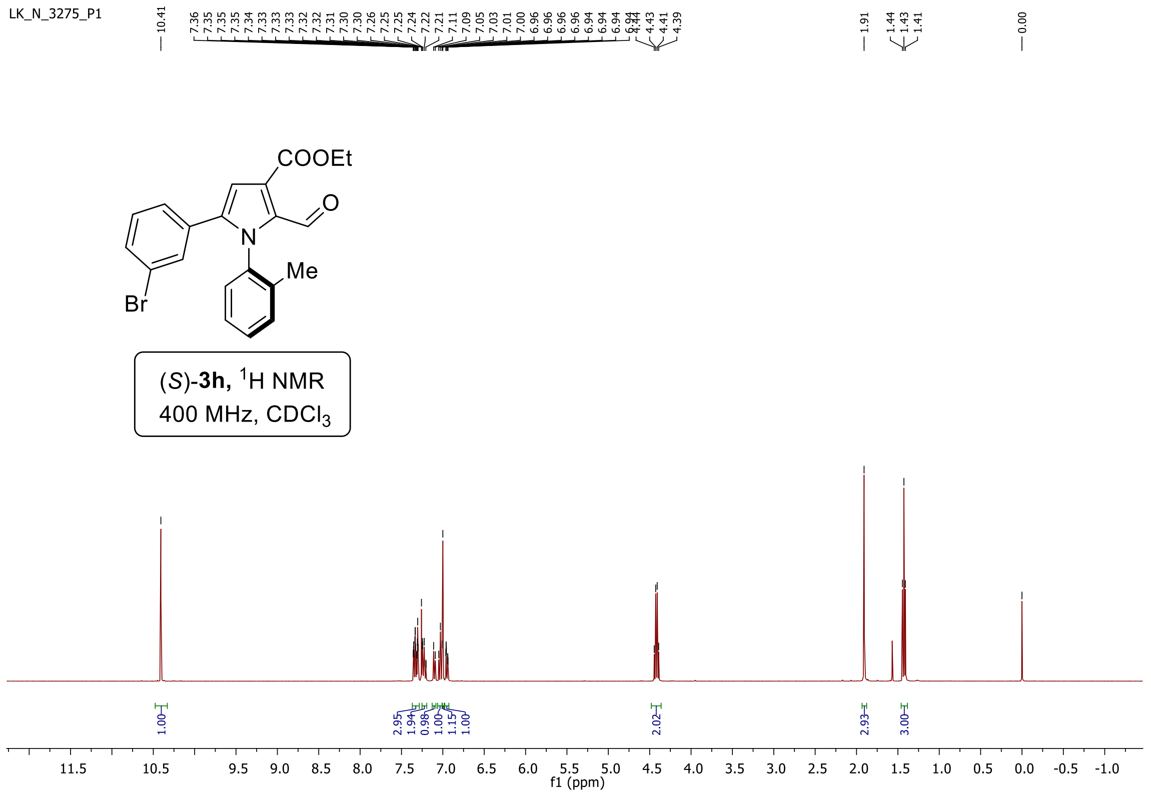

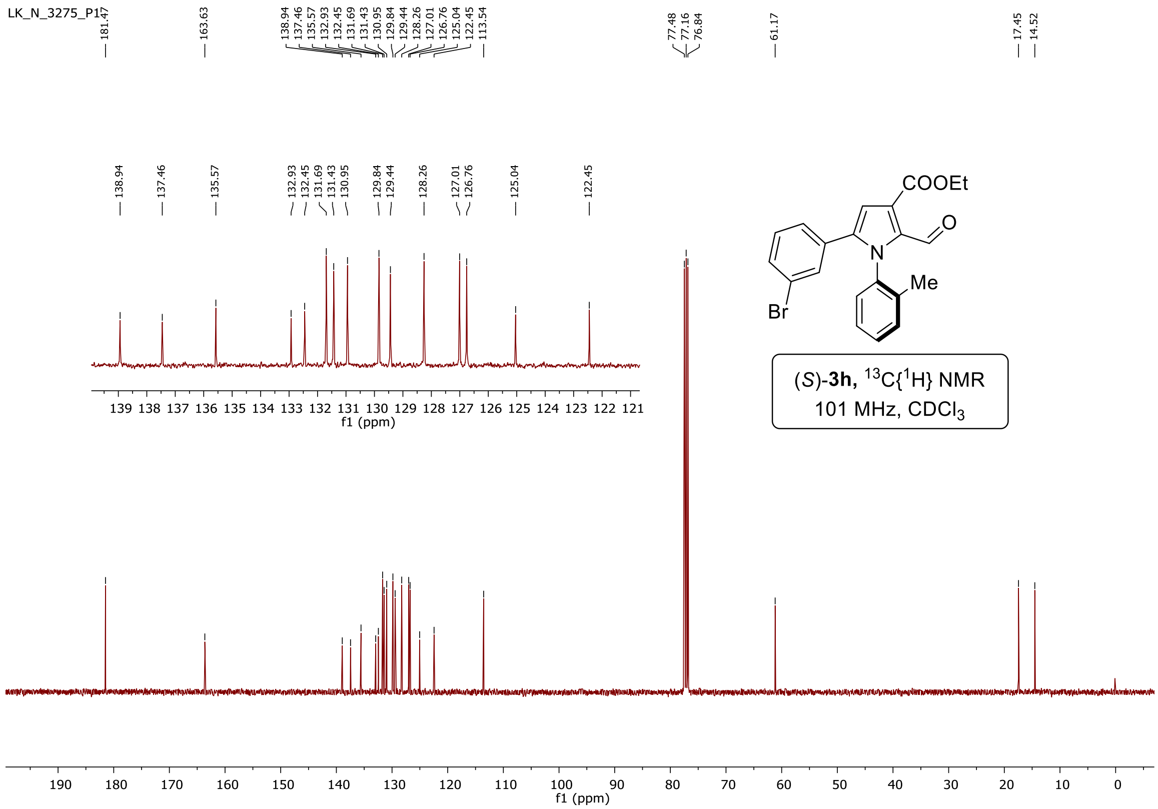

^{1}H and $^{13}C\{^{1}H\}$ NMR spectra of (*R*)-**2i**

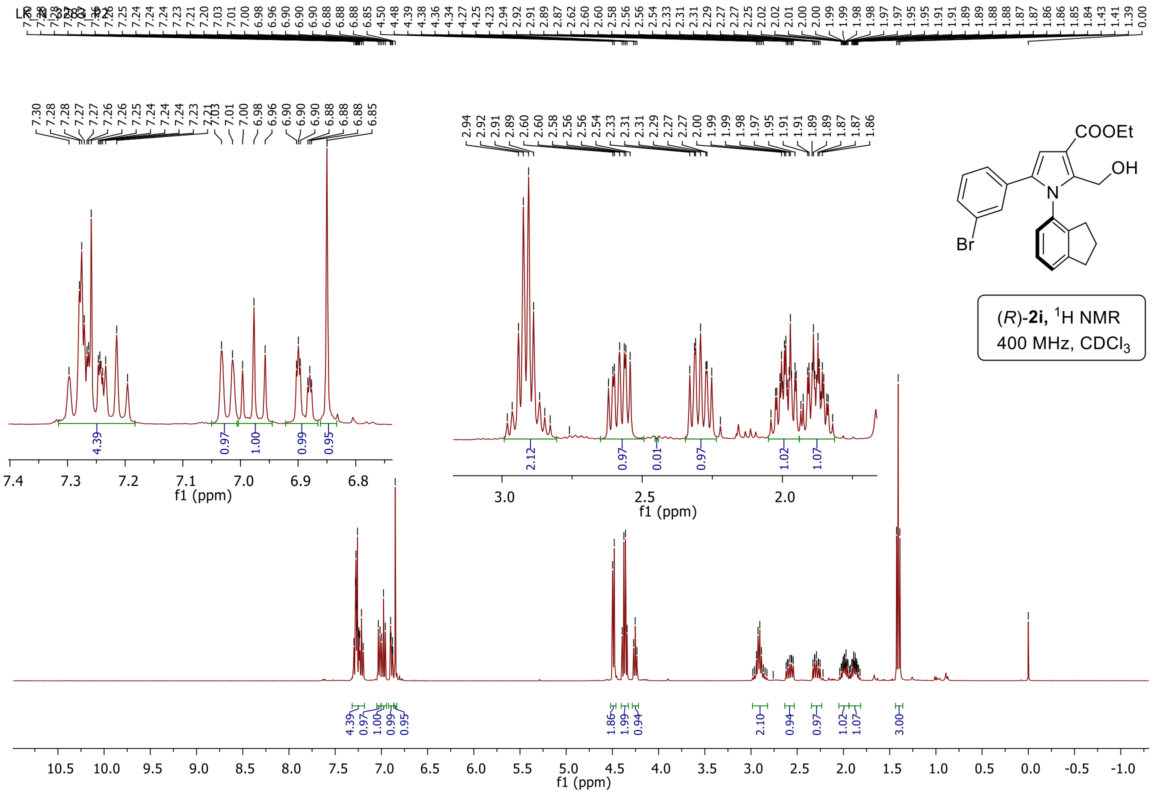

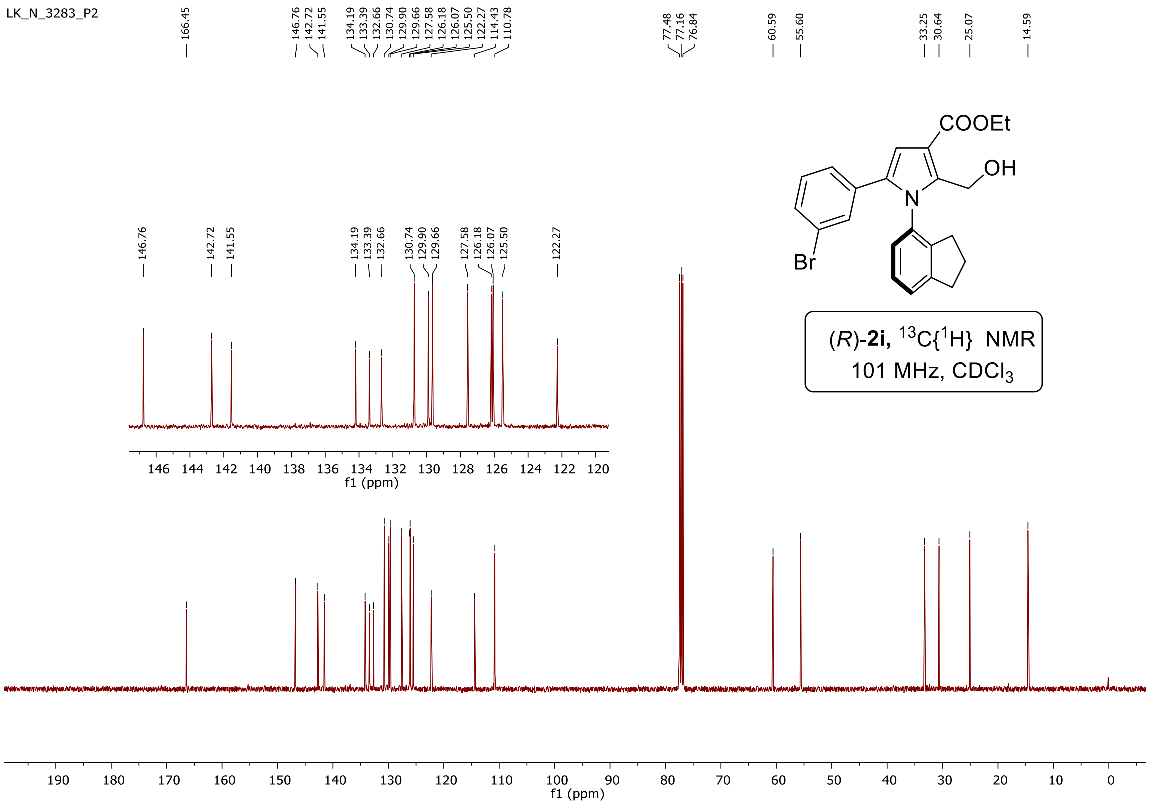

^{1}H and $^{13}C\{^{1}H\}$ NMR spectra of (*S*)-**3i**

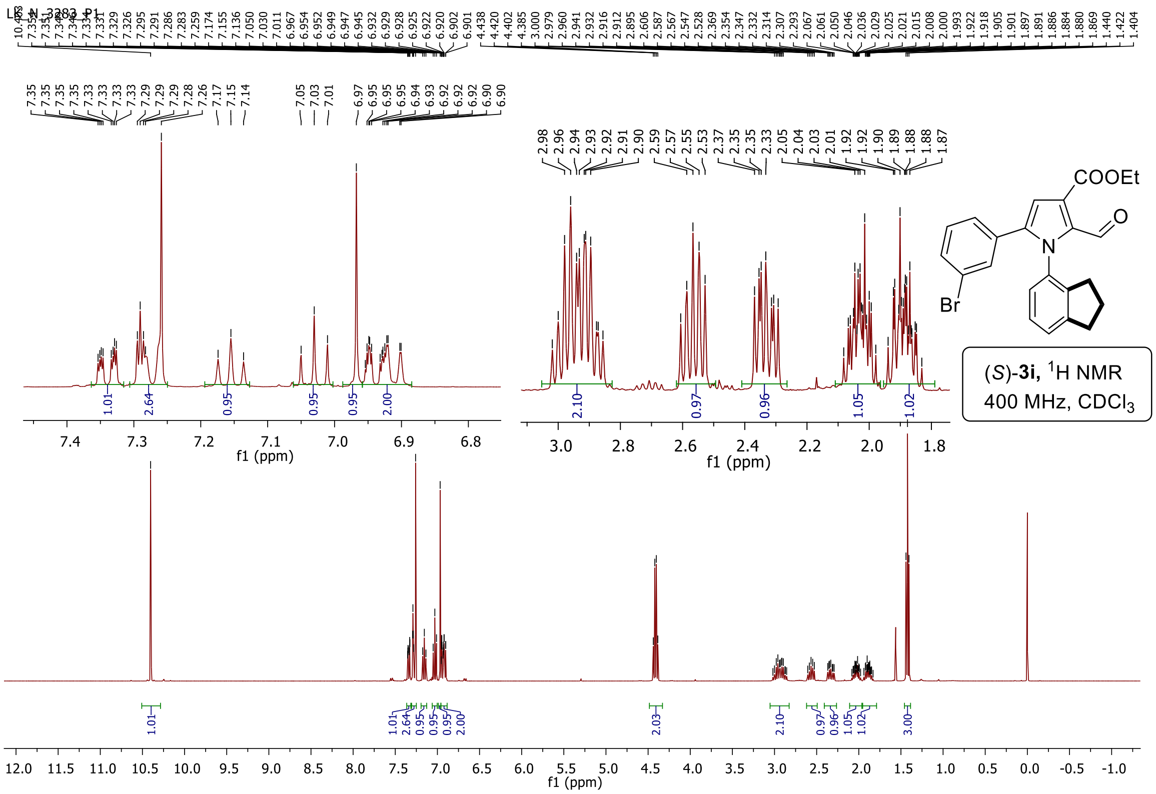

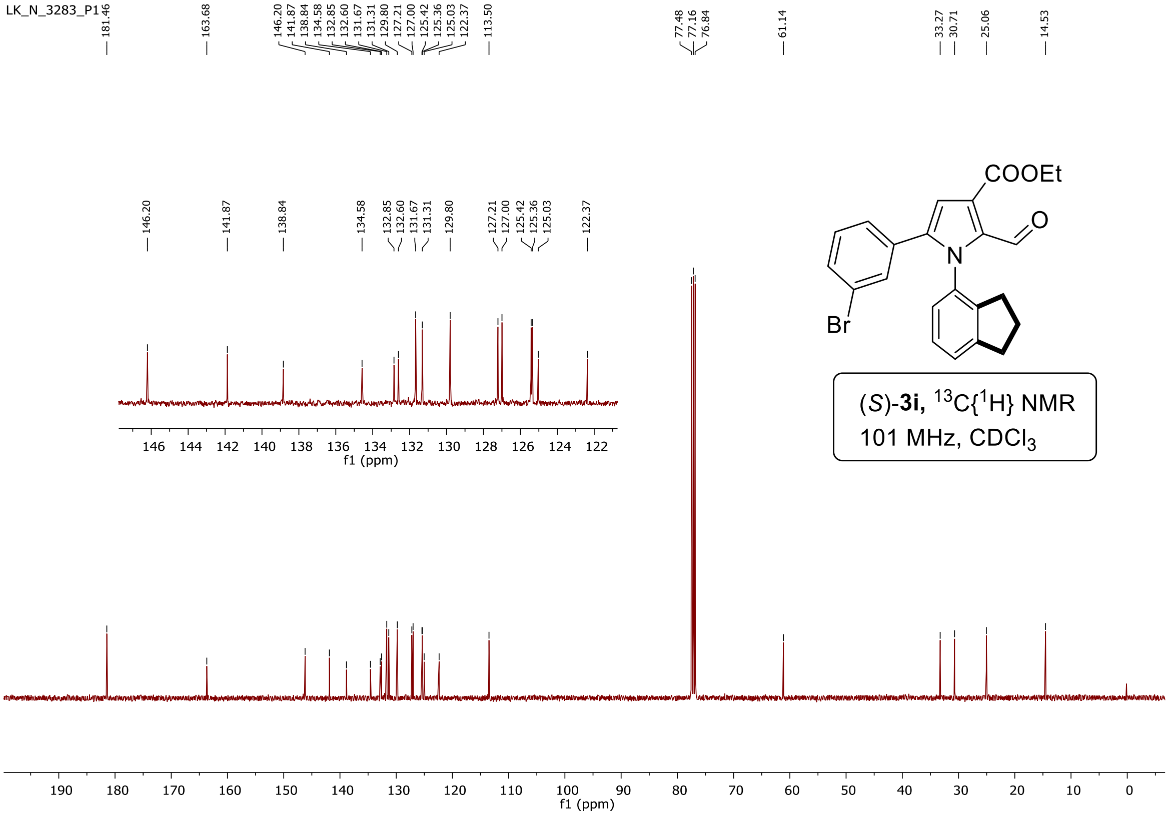

^{1}H and $^{13}C\{^{1}H\}$ NMR spectra of (*R*)-**2j**

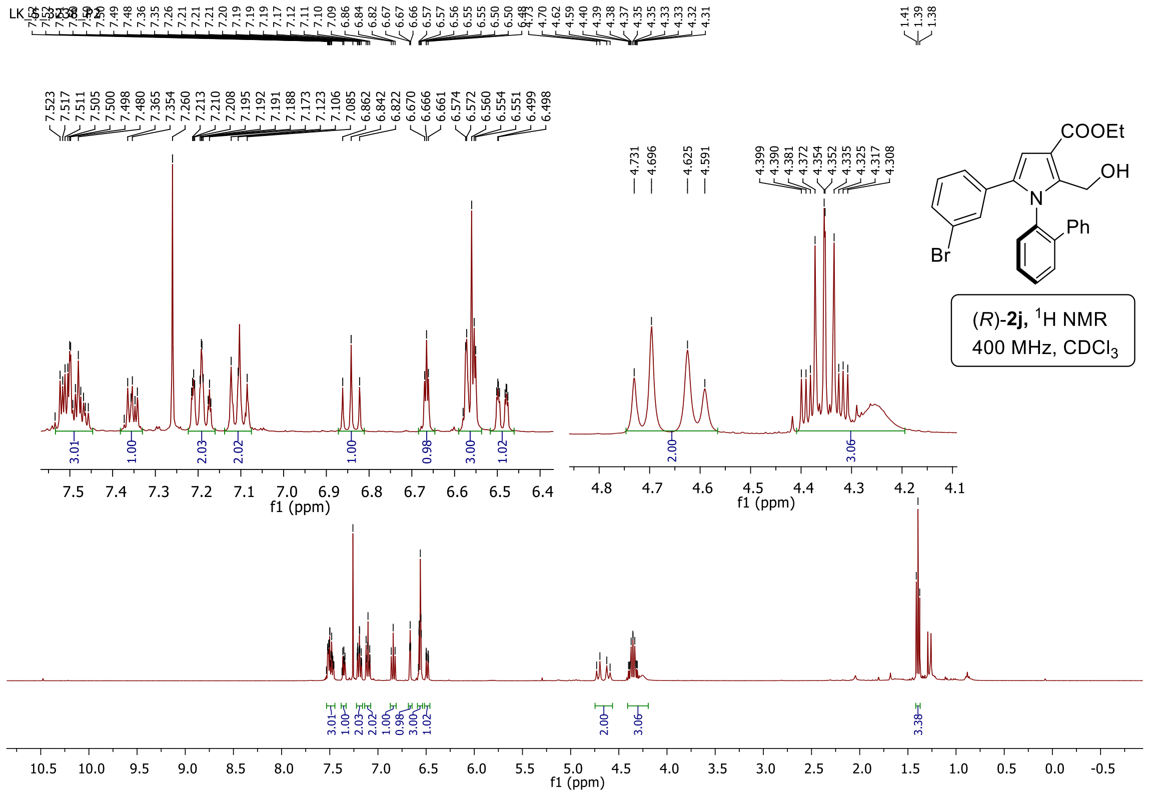

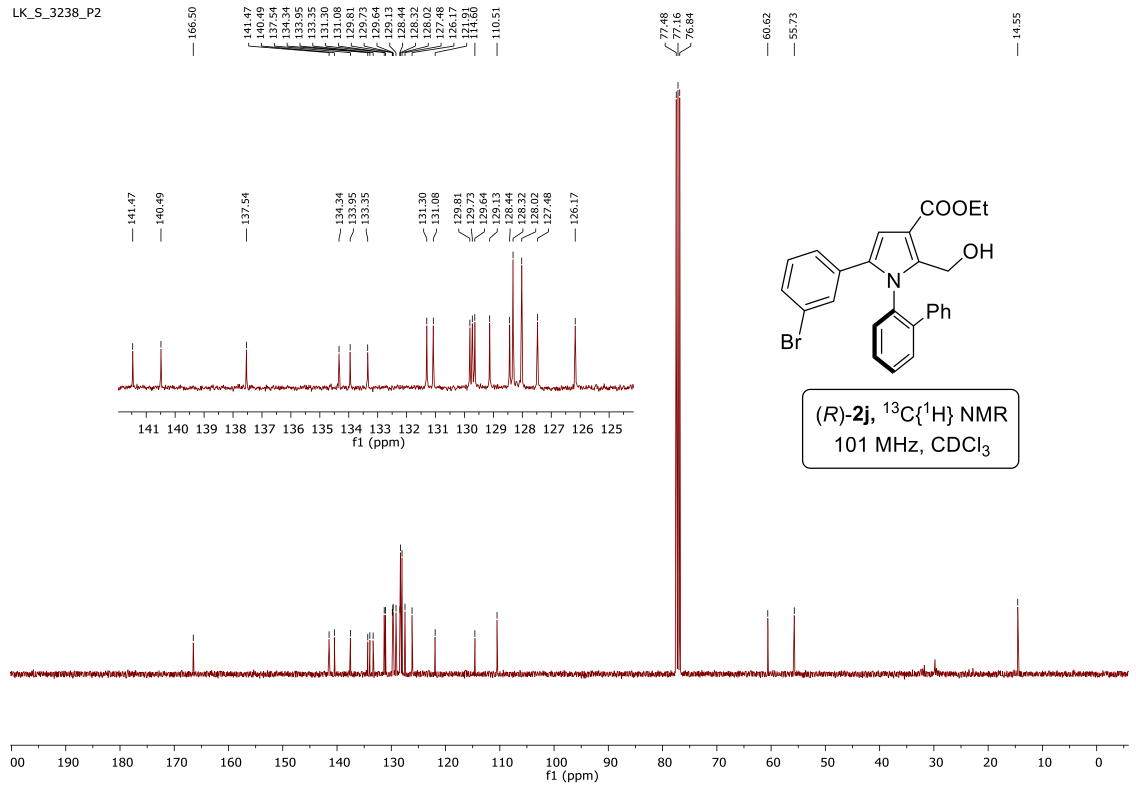

^{1}H and $^{13}C\{^{1}H\}$ NMR spectra of (*S*)-3**j**

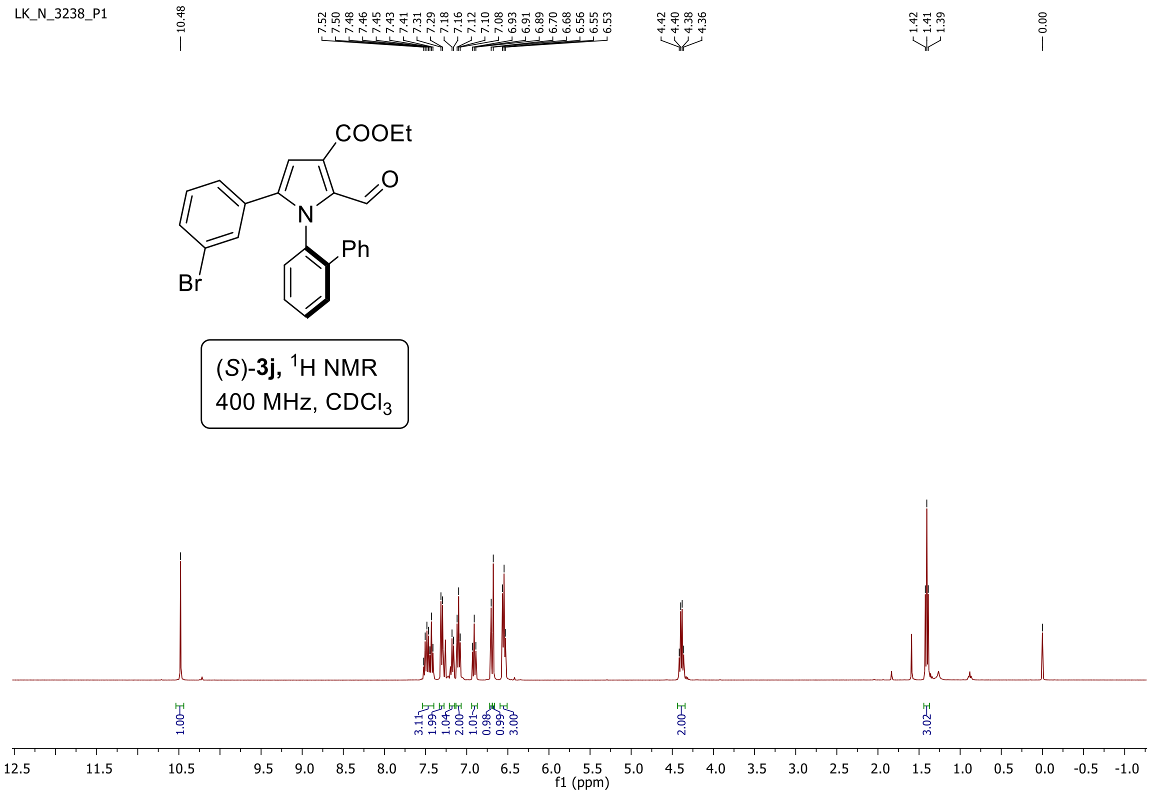

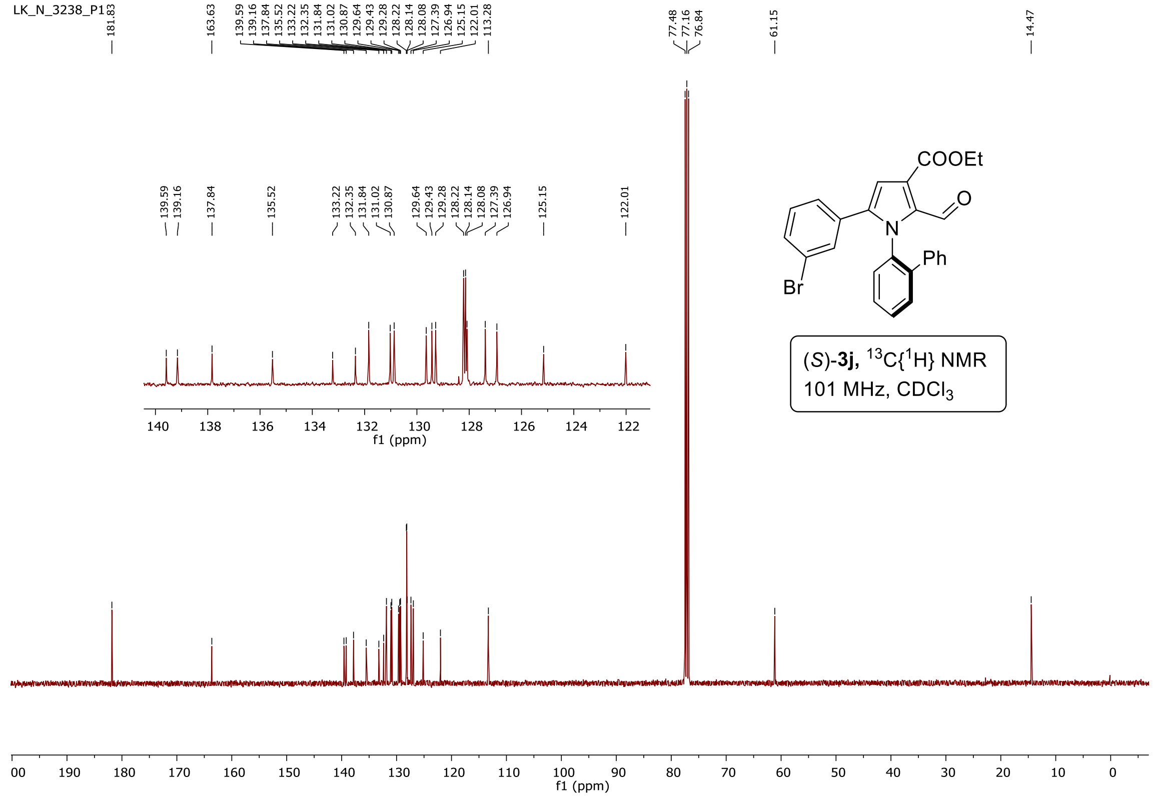

^{1}H and ^{13}C{^{1}H} NMR spectra of (*R*)-**2k**

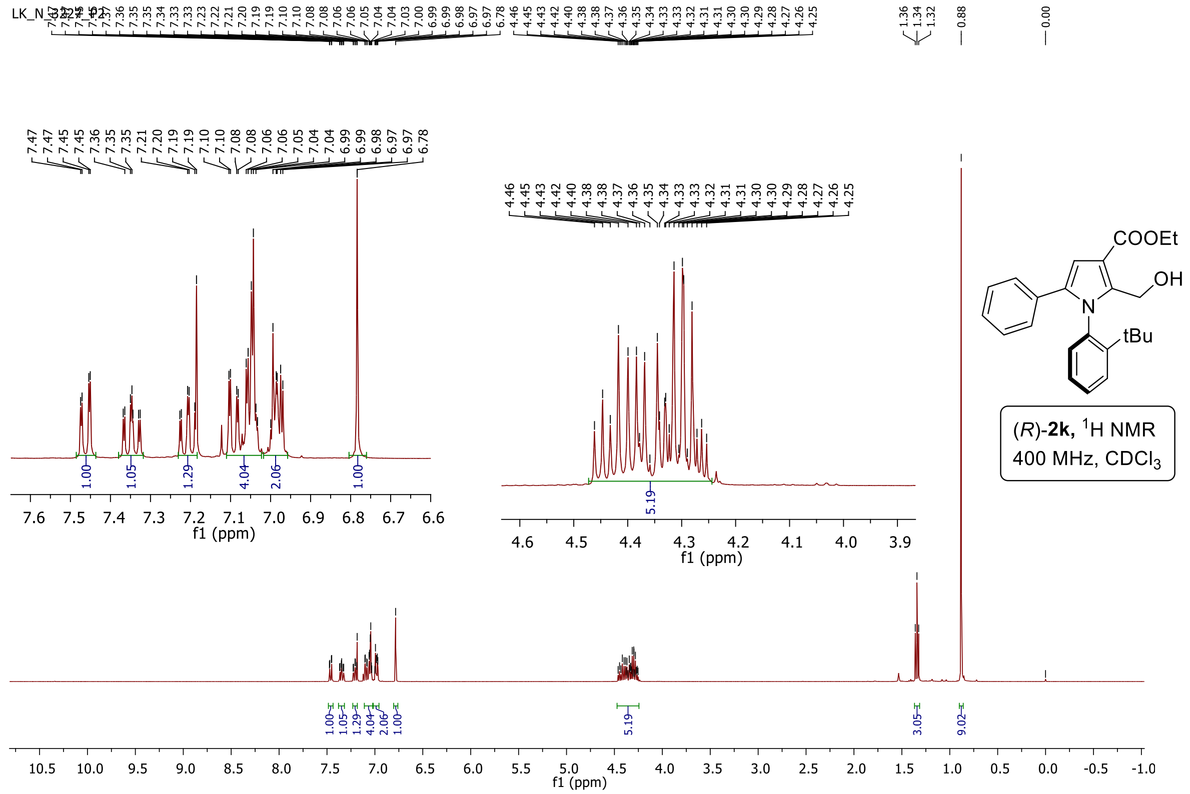

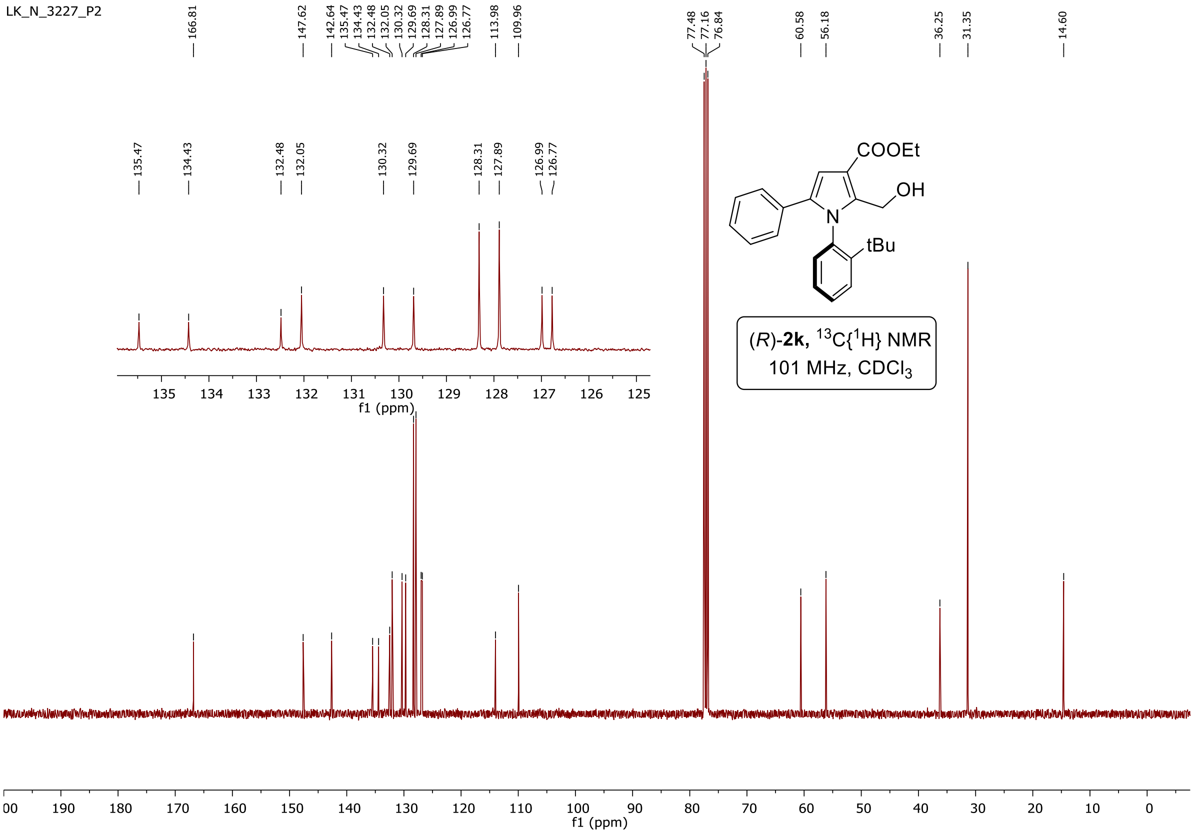

^{1}H and $^{13}C\{^{1}H\}$ NMR spectra of (*S*)-3**k**

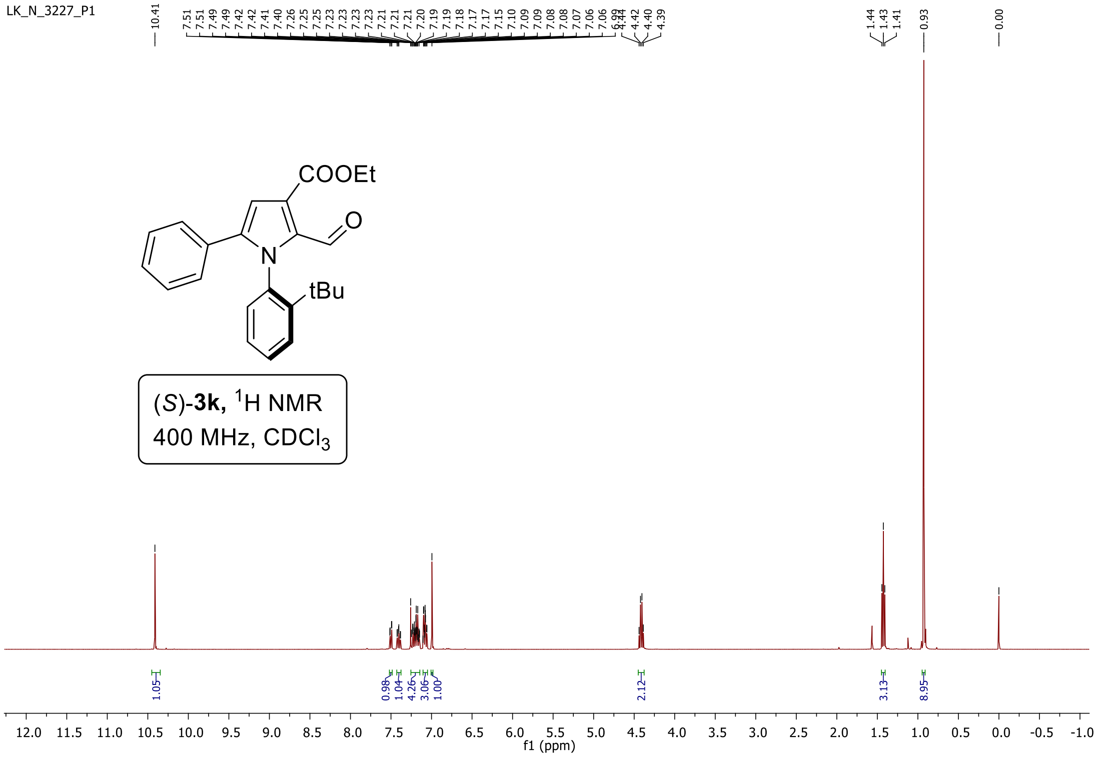

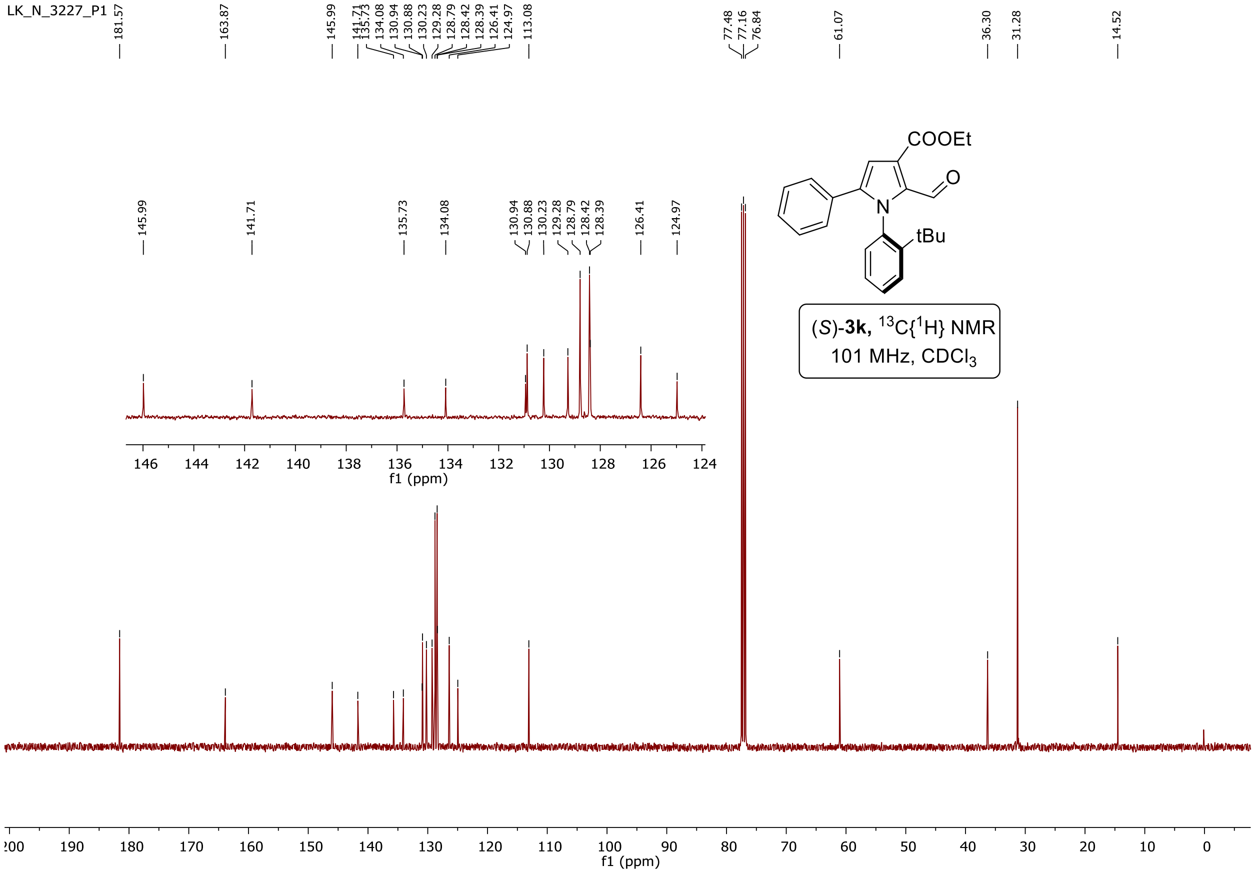

¹H and ¹³C{¹H} NMR spectra of (*R*)-**2l**

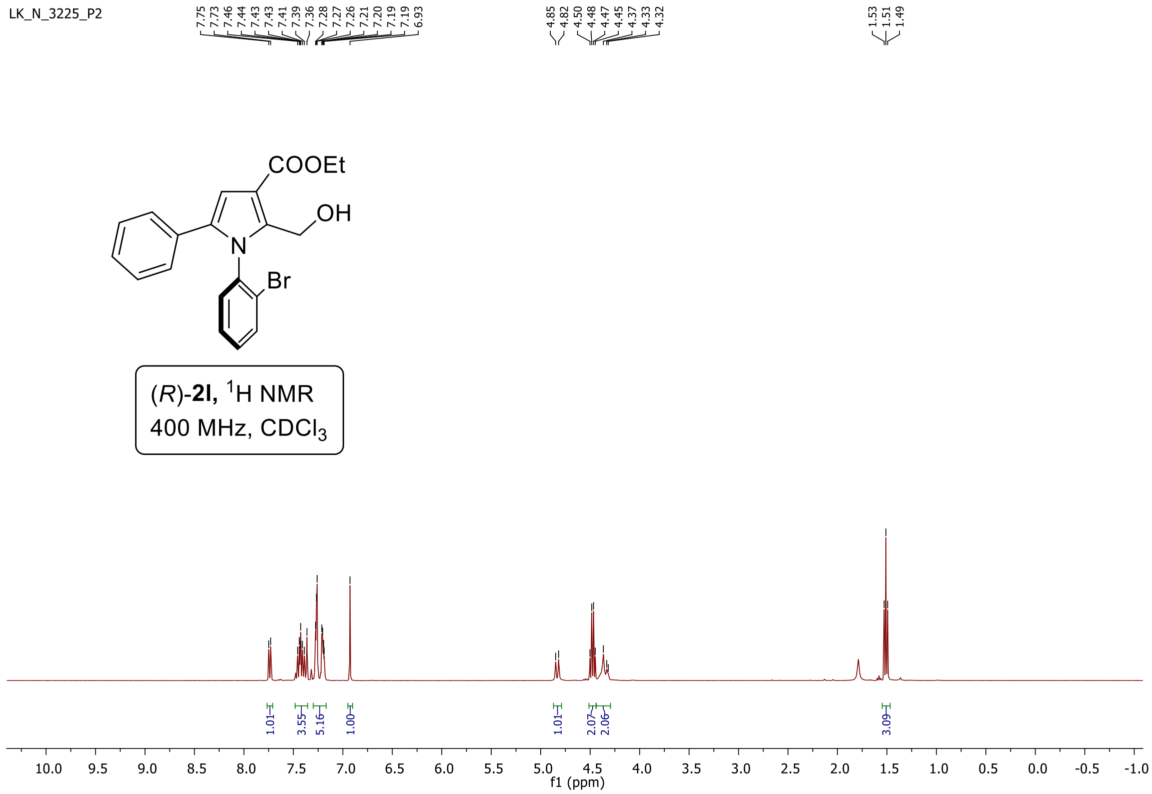

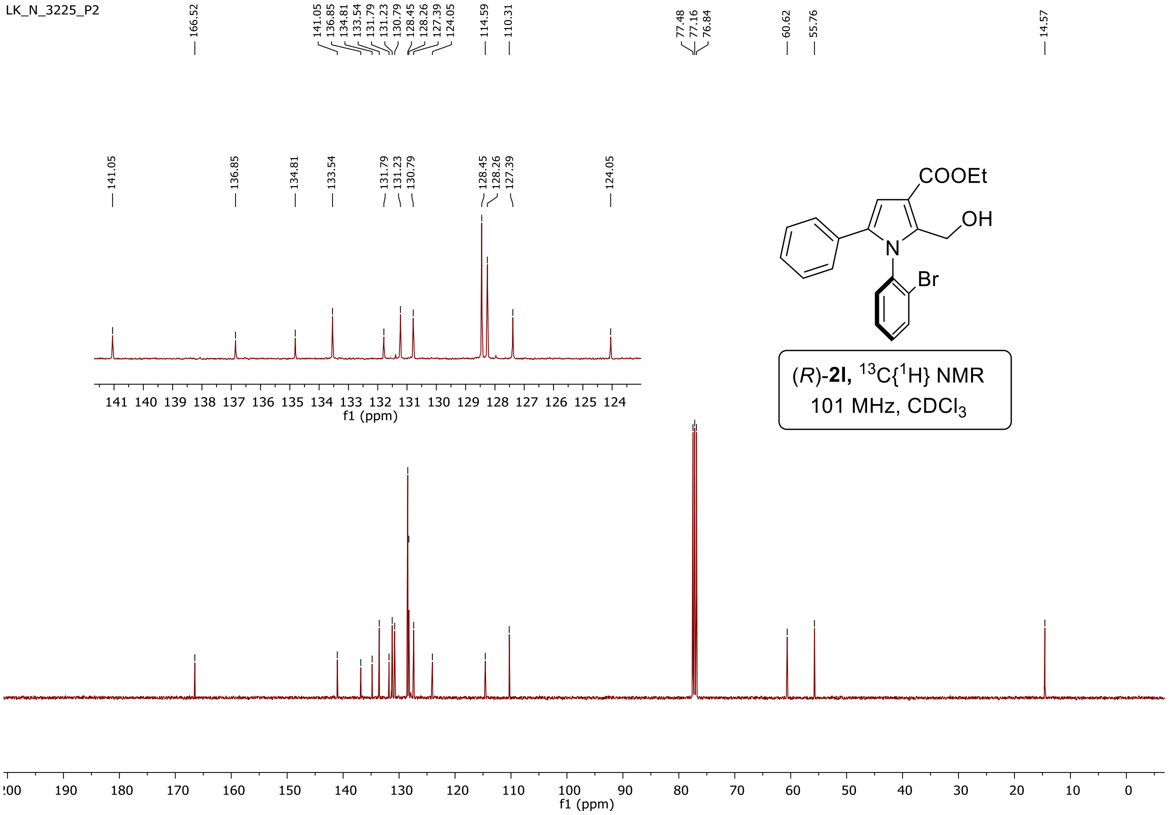

^{1}H and $^{13}C\{^{1}H\}$ NMR spectra of (*S*)-**3l**

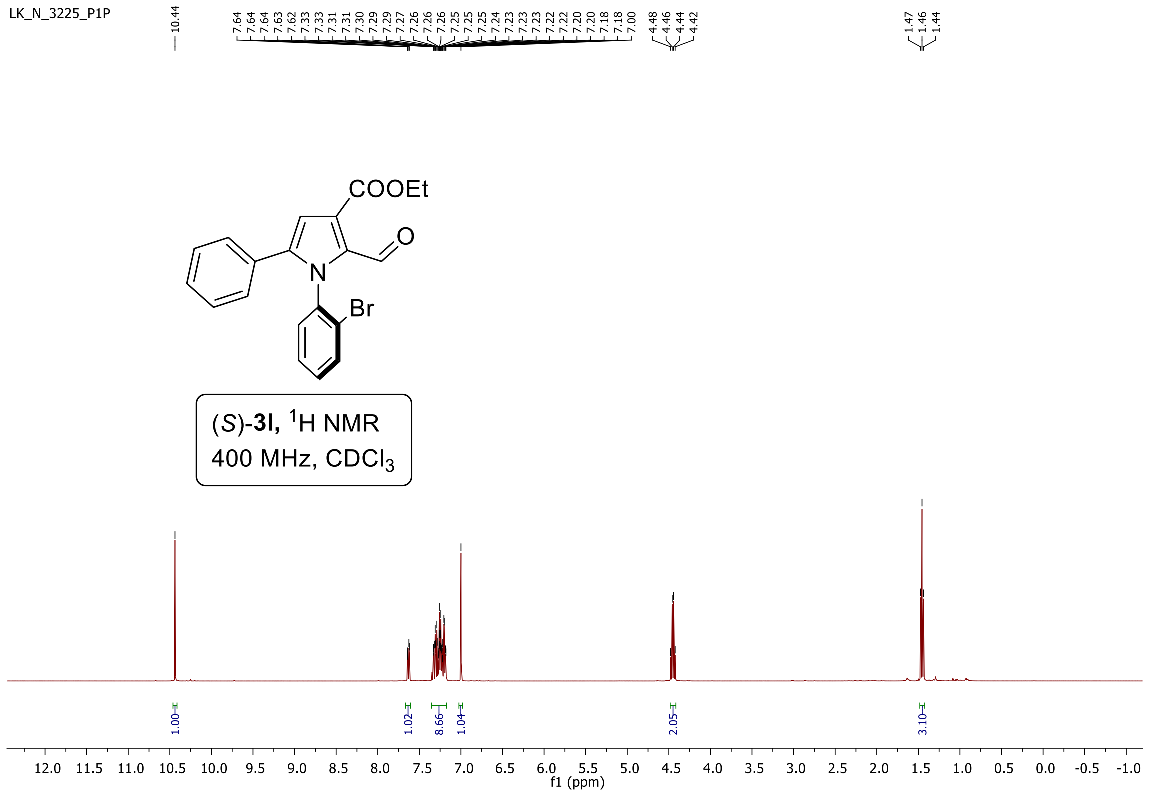

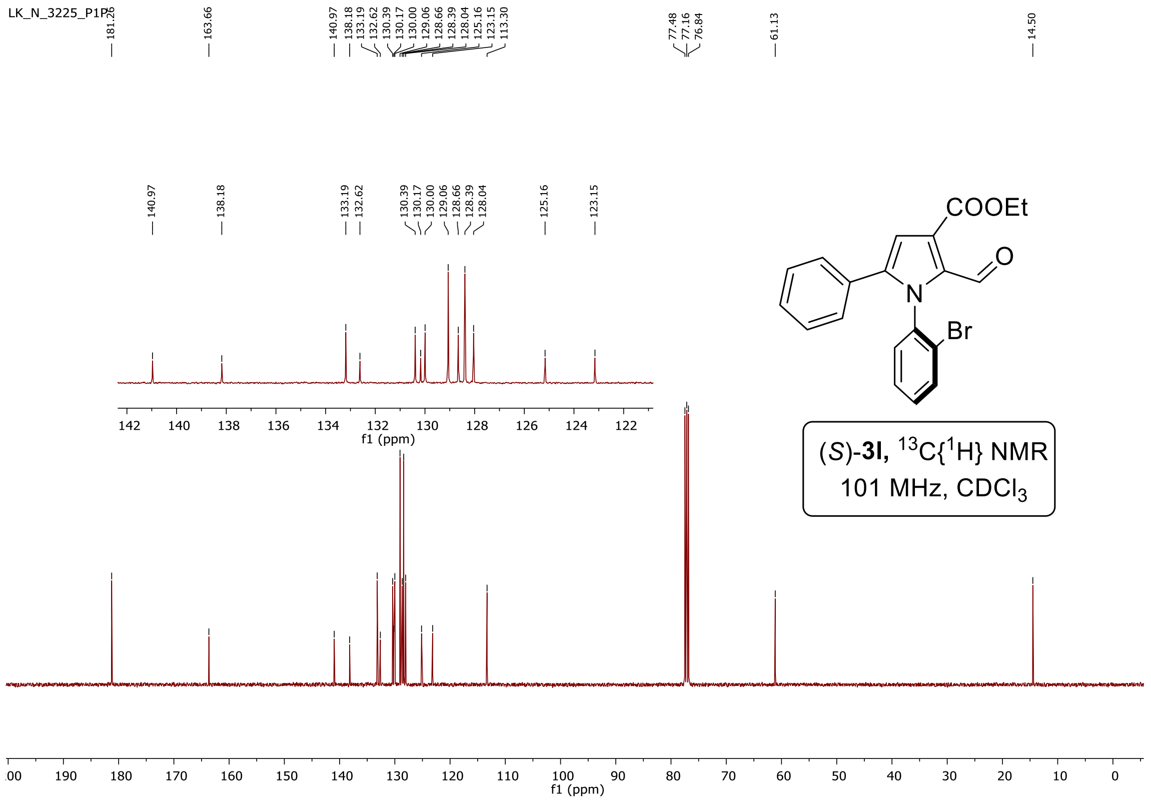

^{1}H and ^{13}C{^{1}H} NMR spectra of (*R*)-**2m**

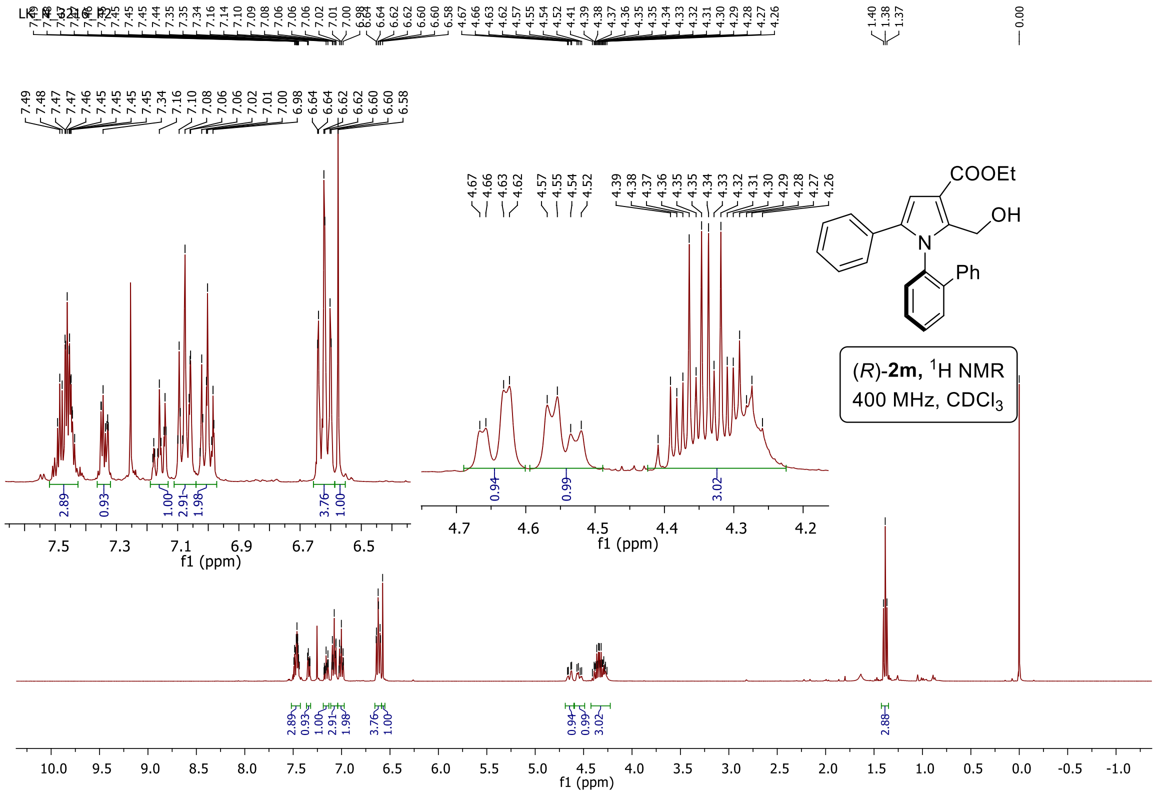

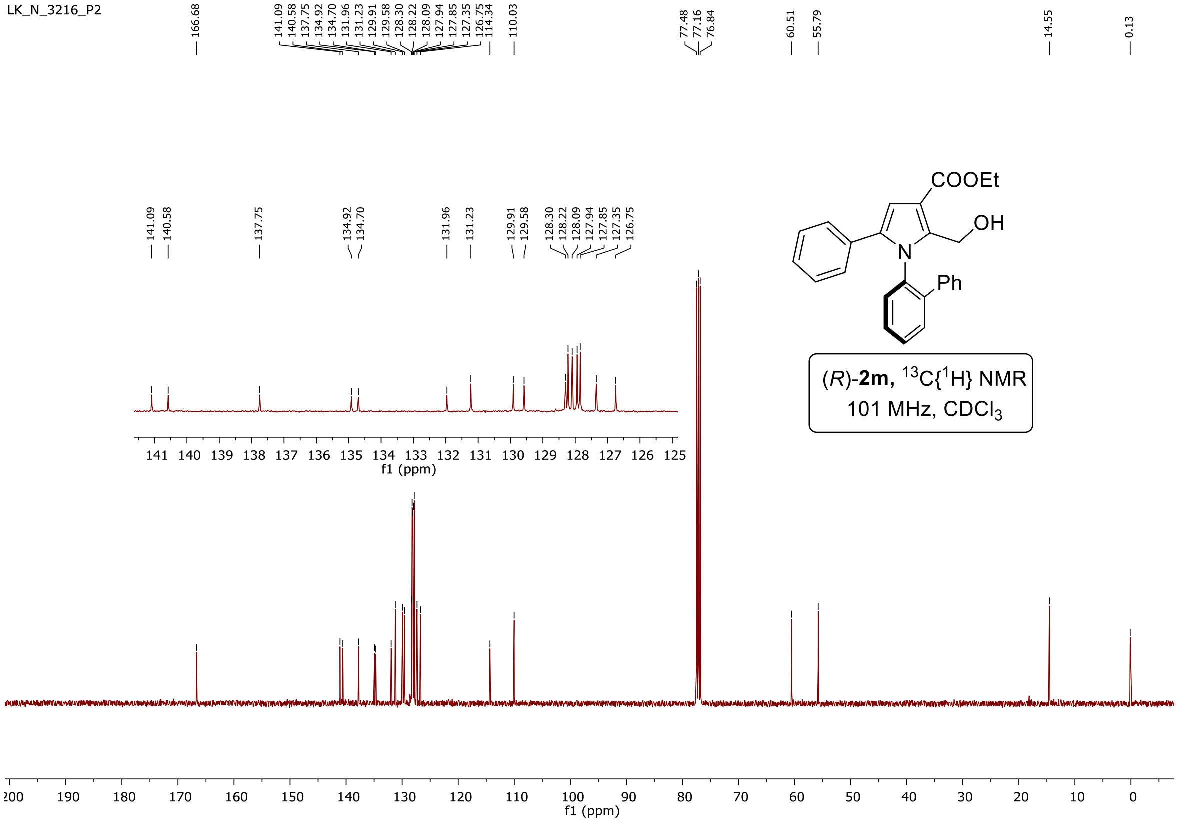

^1H and $^{13}C\{^1H\}$ NMR spectra of (*S*)-**3m**

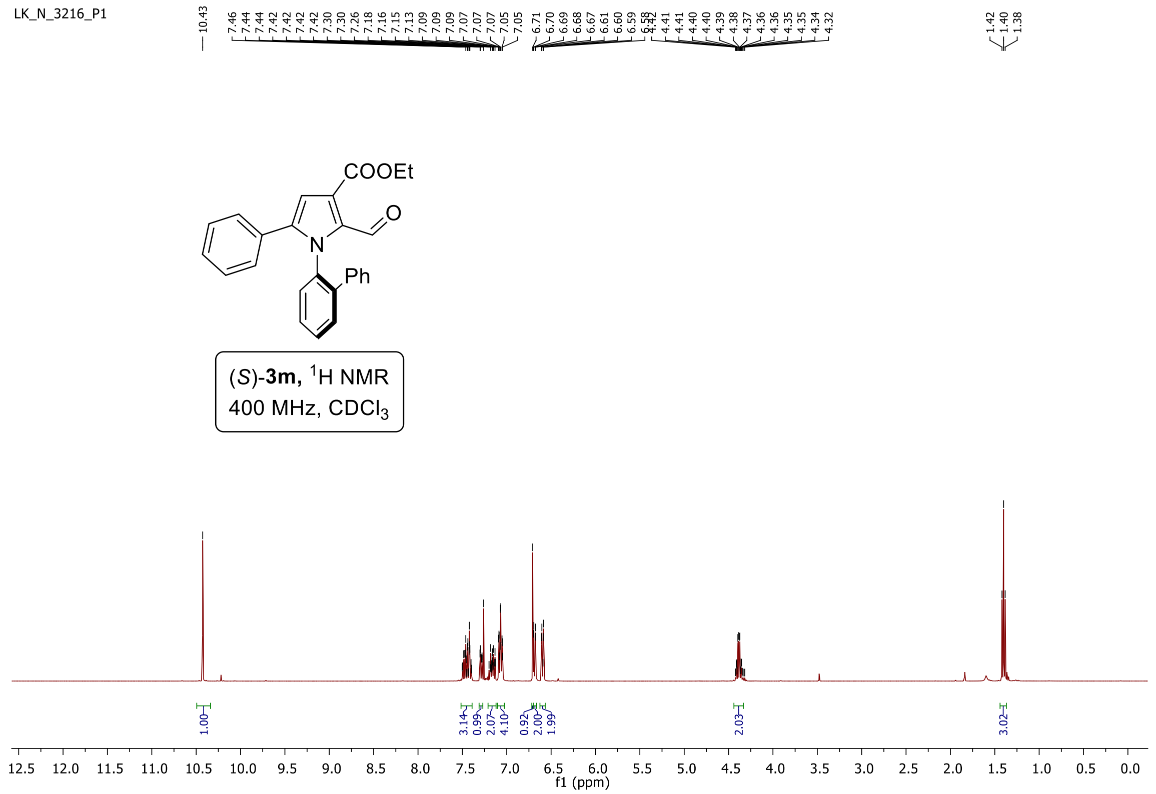

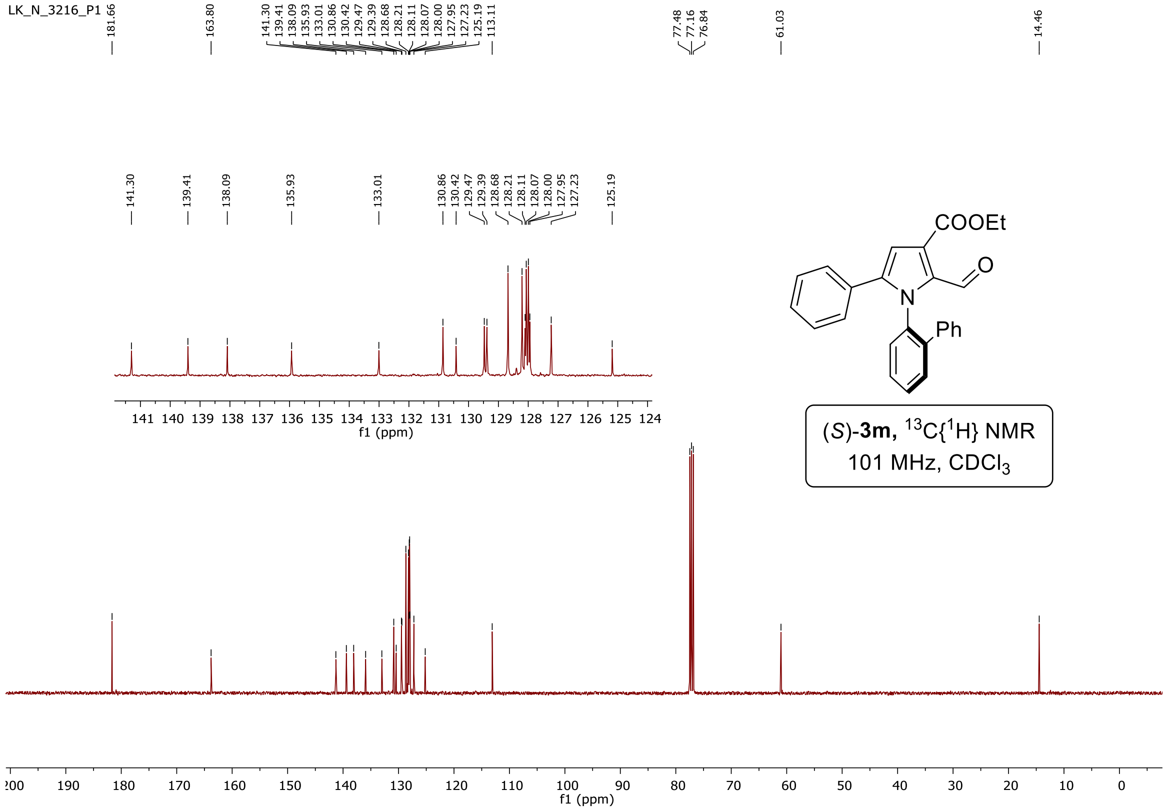

^{1}H and ^{13}C{^{1}H} NMR spectra of (*R*)-**2n**

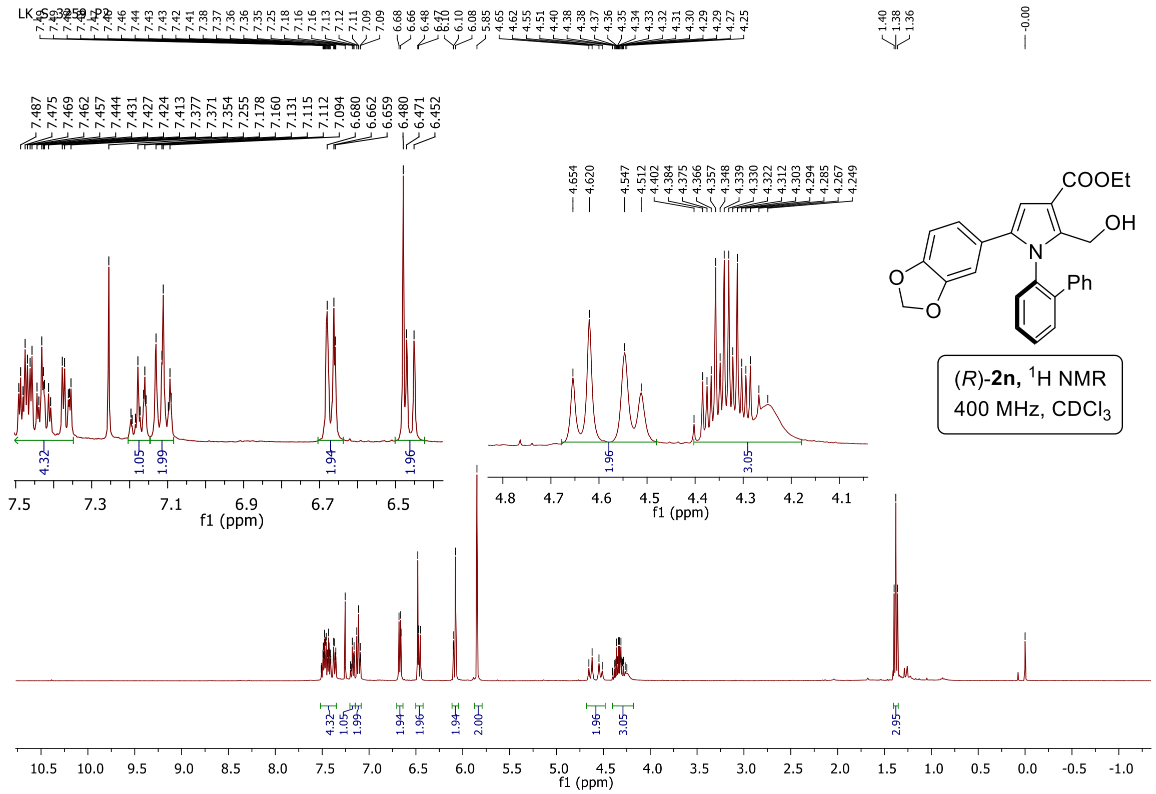

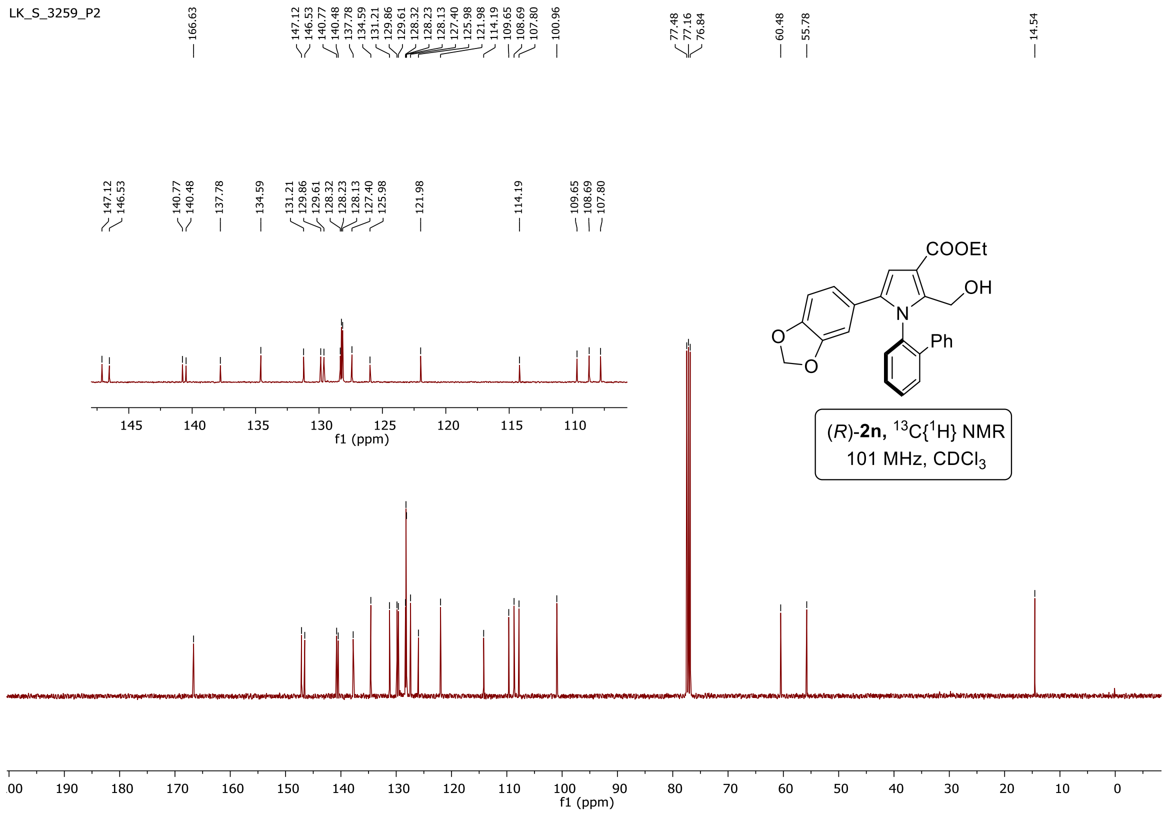

^{1}H and $^{13}C\{^{1}H\}$ NMR spectra of (*S*)-**3n**

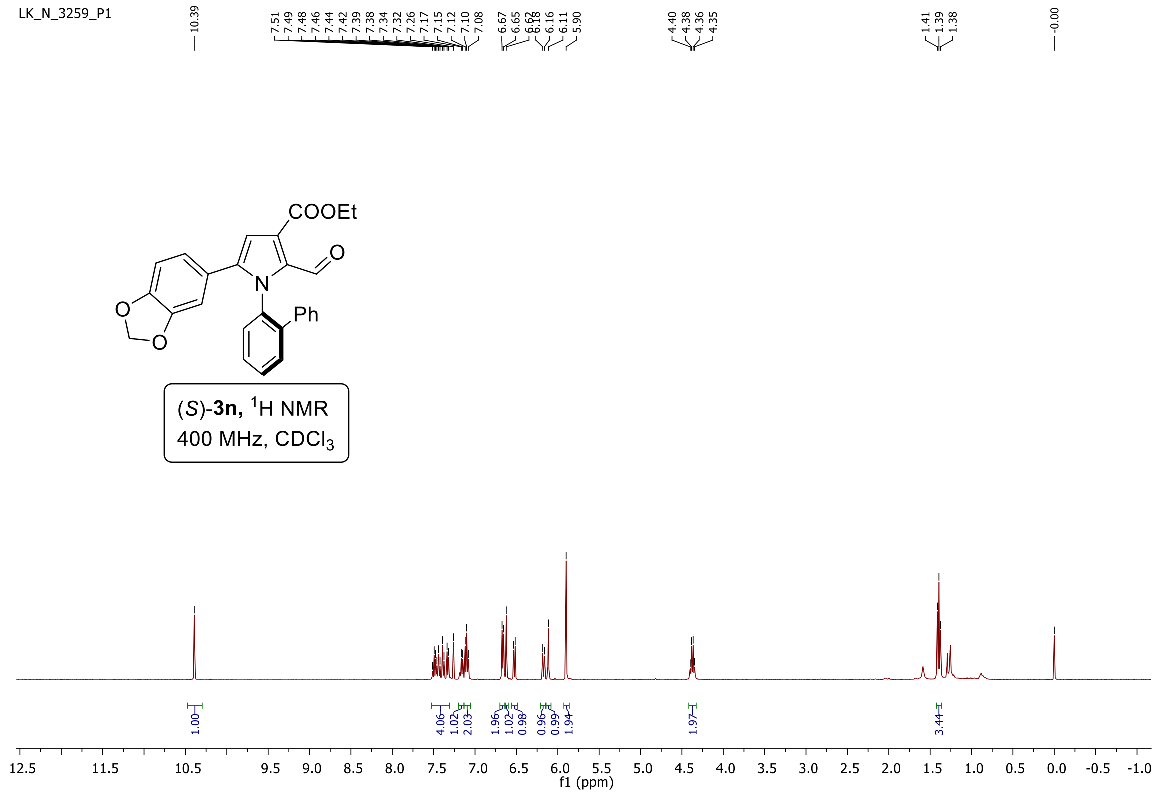

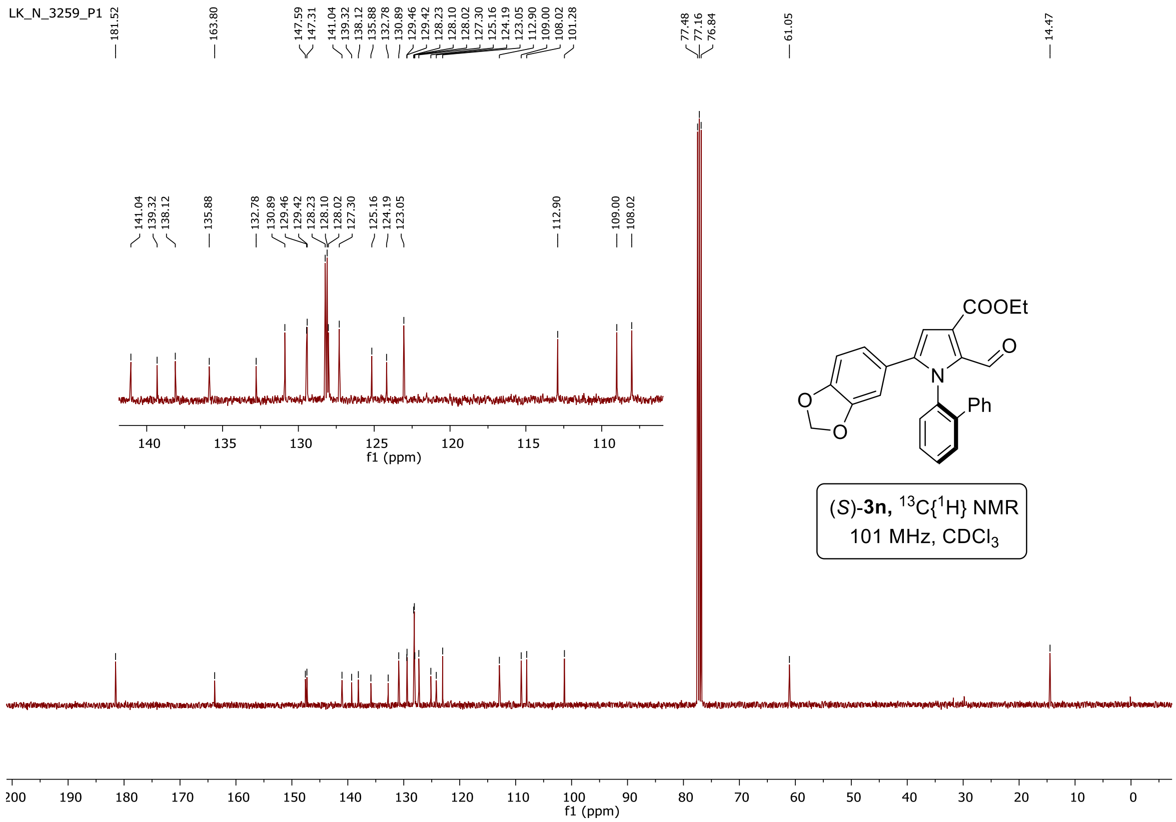

^{1}H and $^{13}C\{^{1}H\}$ NMR spectra of (*R*)-**2o**

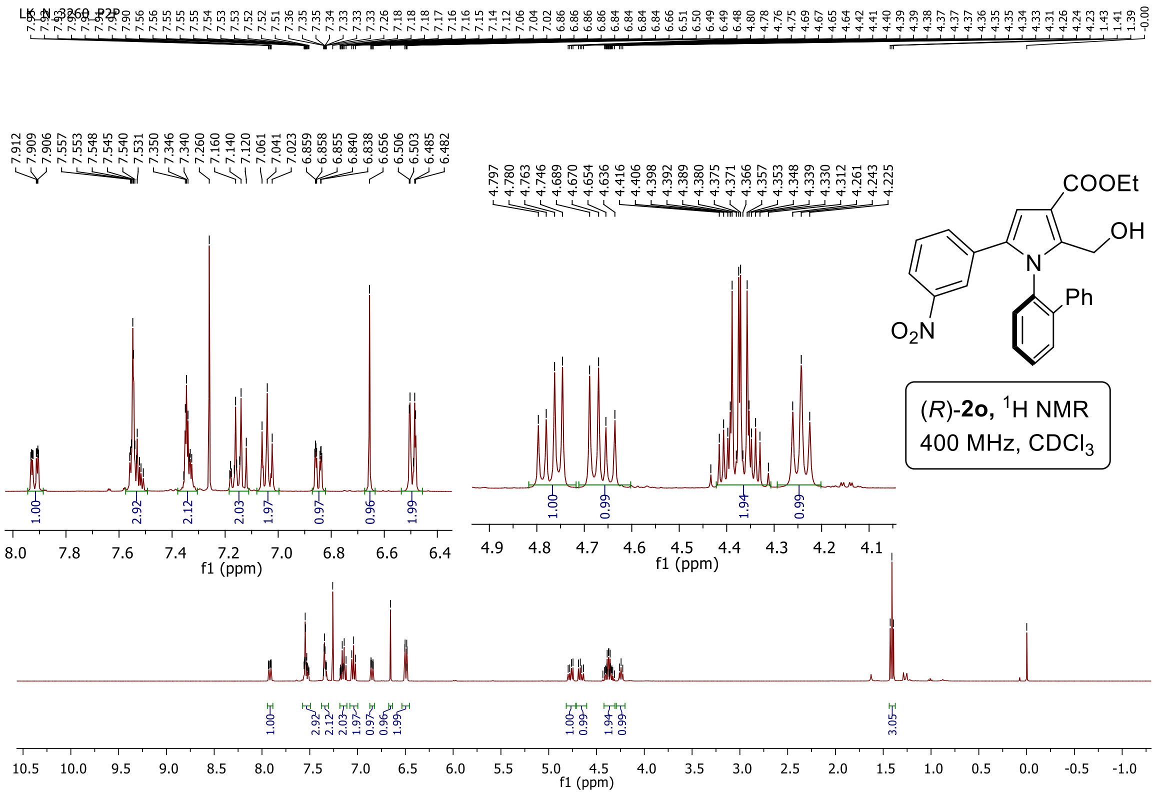

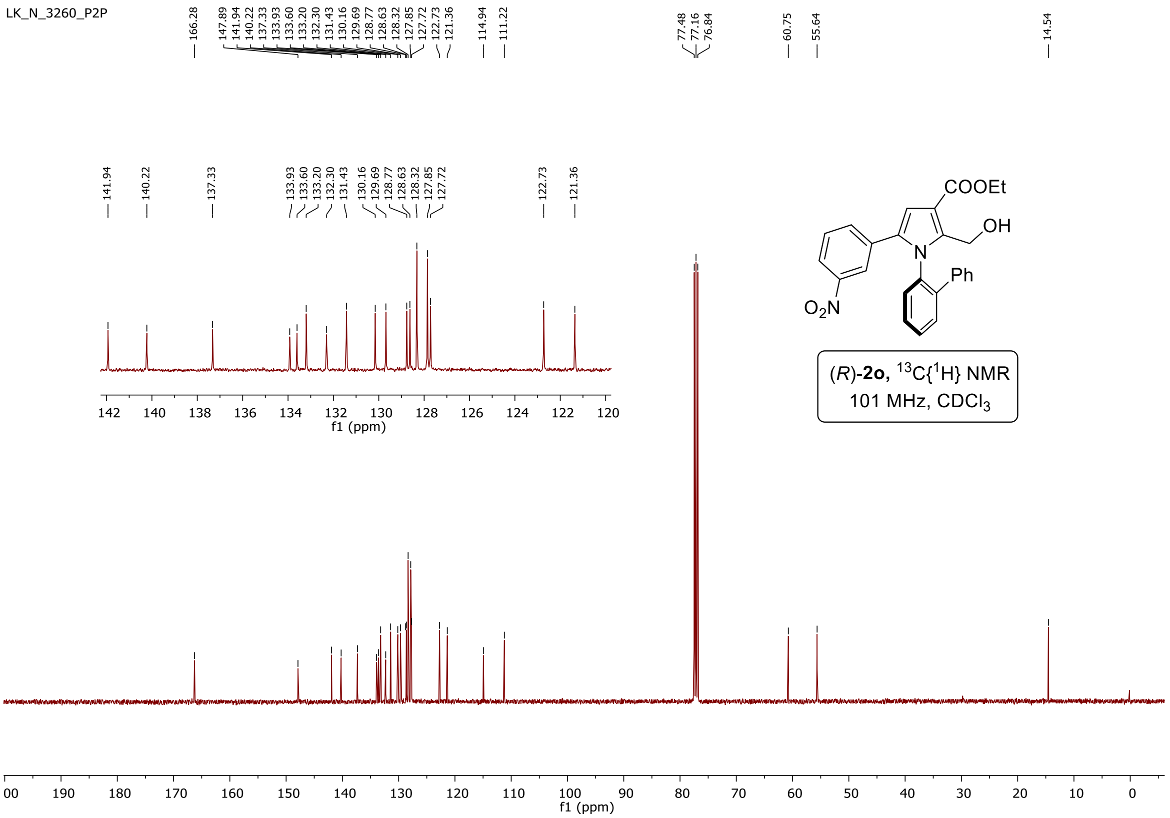

^1H and $^{13}C\{^1H\}$ NMR spectra of (*S*)-**3o**

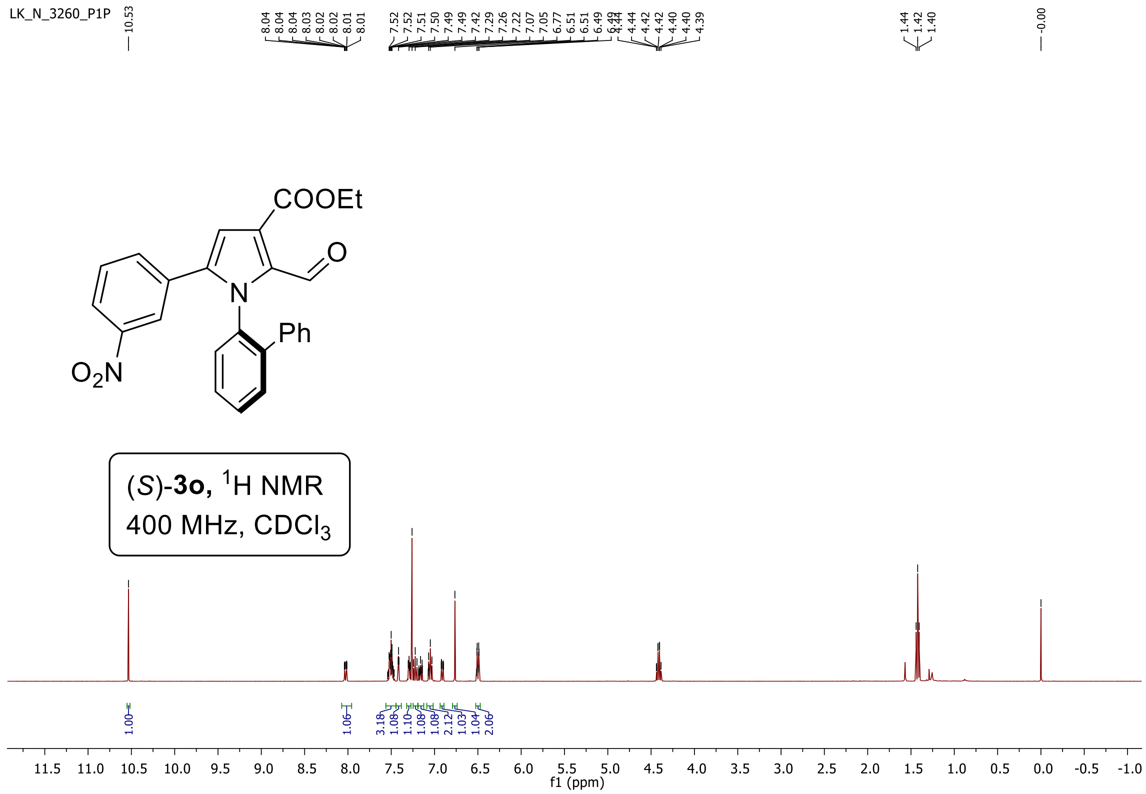

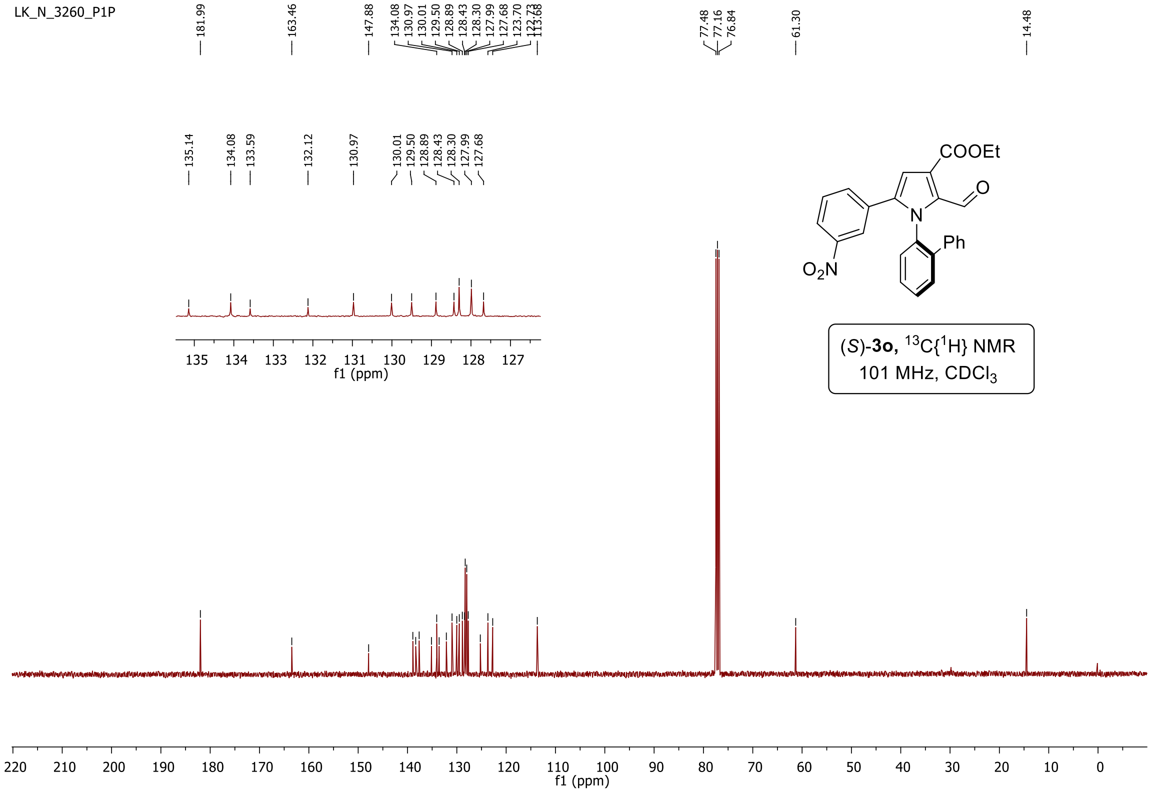

^1H and $^{13}C\{^1H\}$ NMR spectra of (*R*)-**2p**

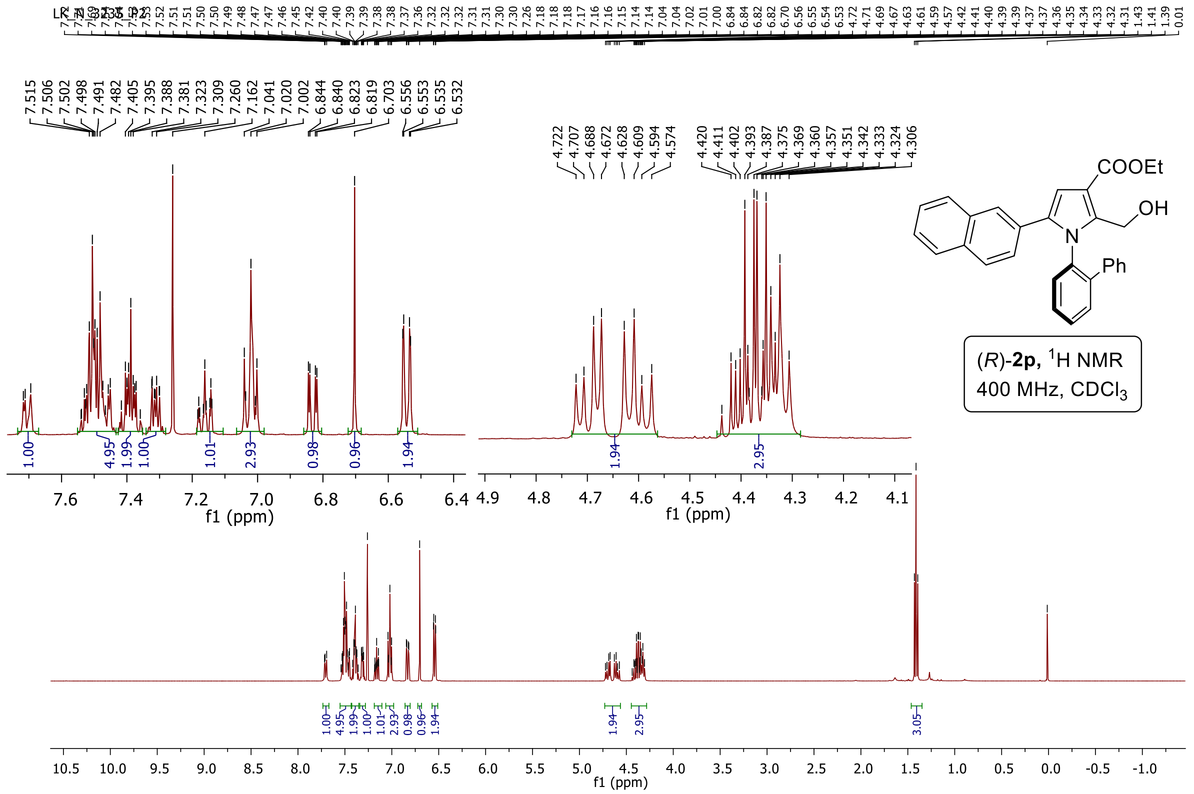

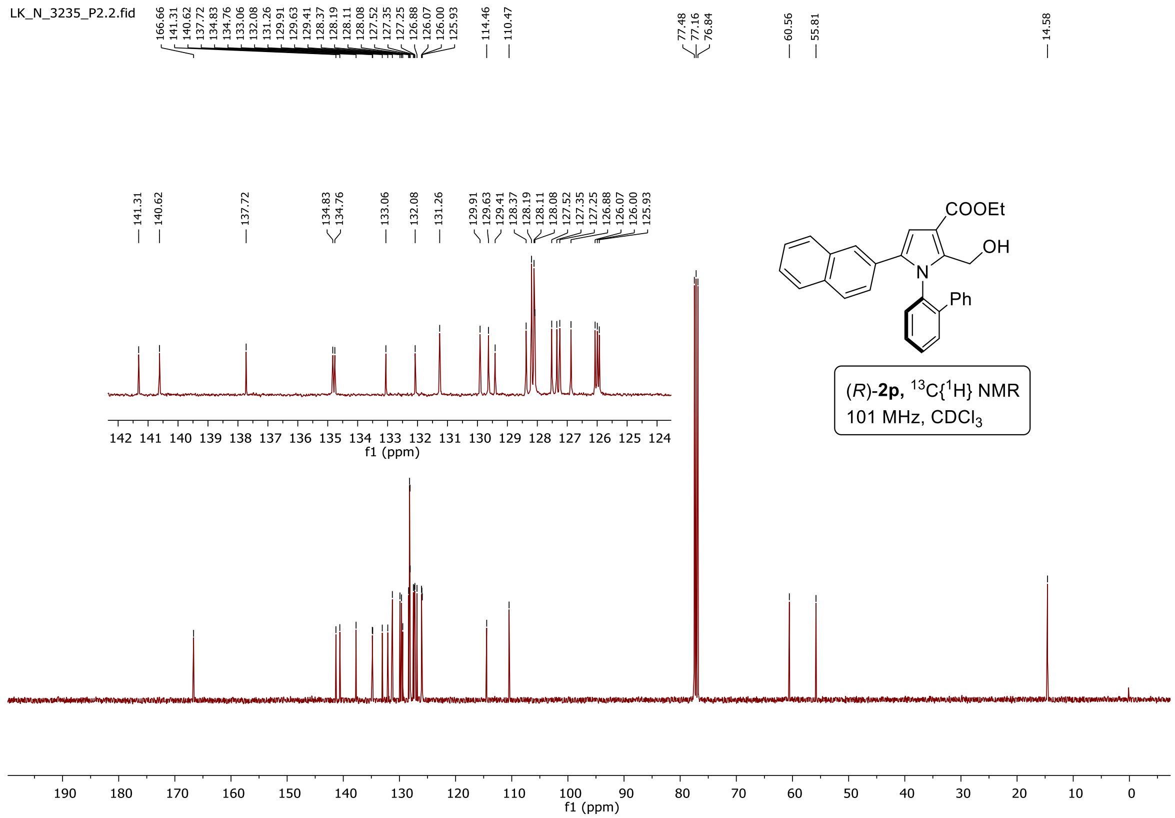

^{1}H and ^{13}C{^{1}H} NMR spectra of (*S*)-**3p**

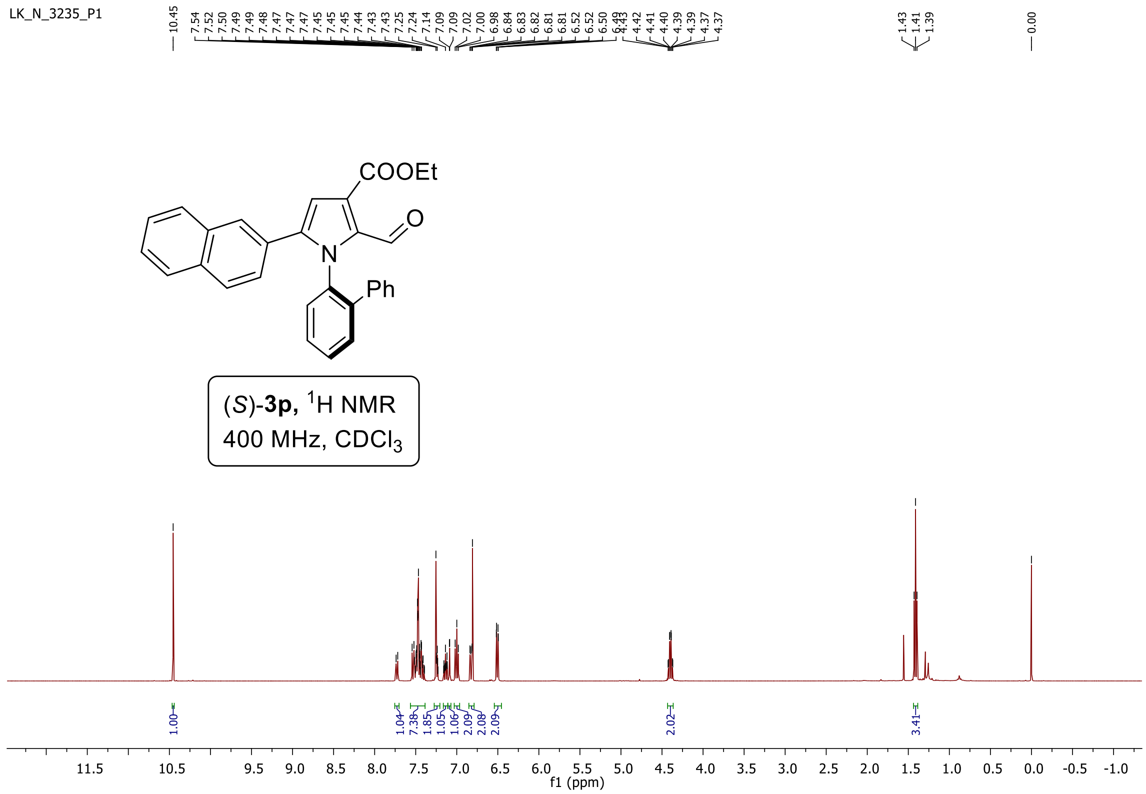

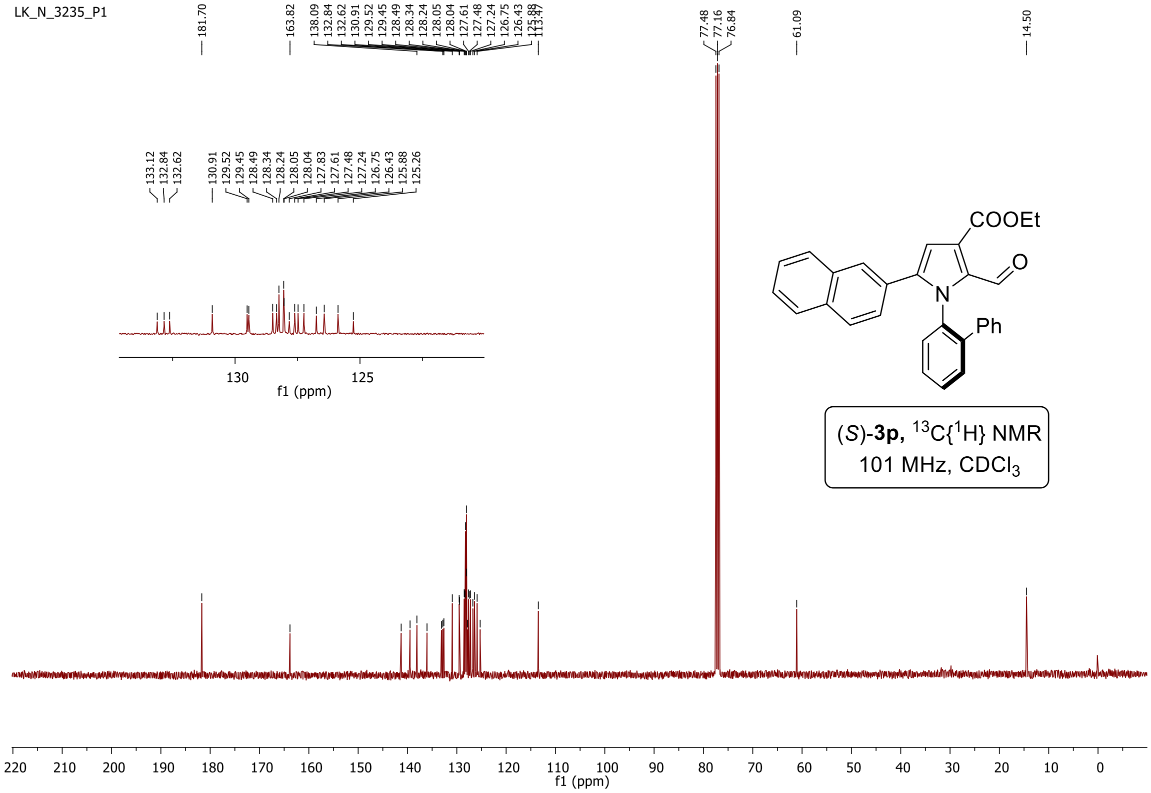

^{1}H ,$^{13}C\{^{1}H\}$ and ^{19}F NMR spectra of (*R*)-**2q**

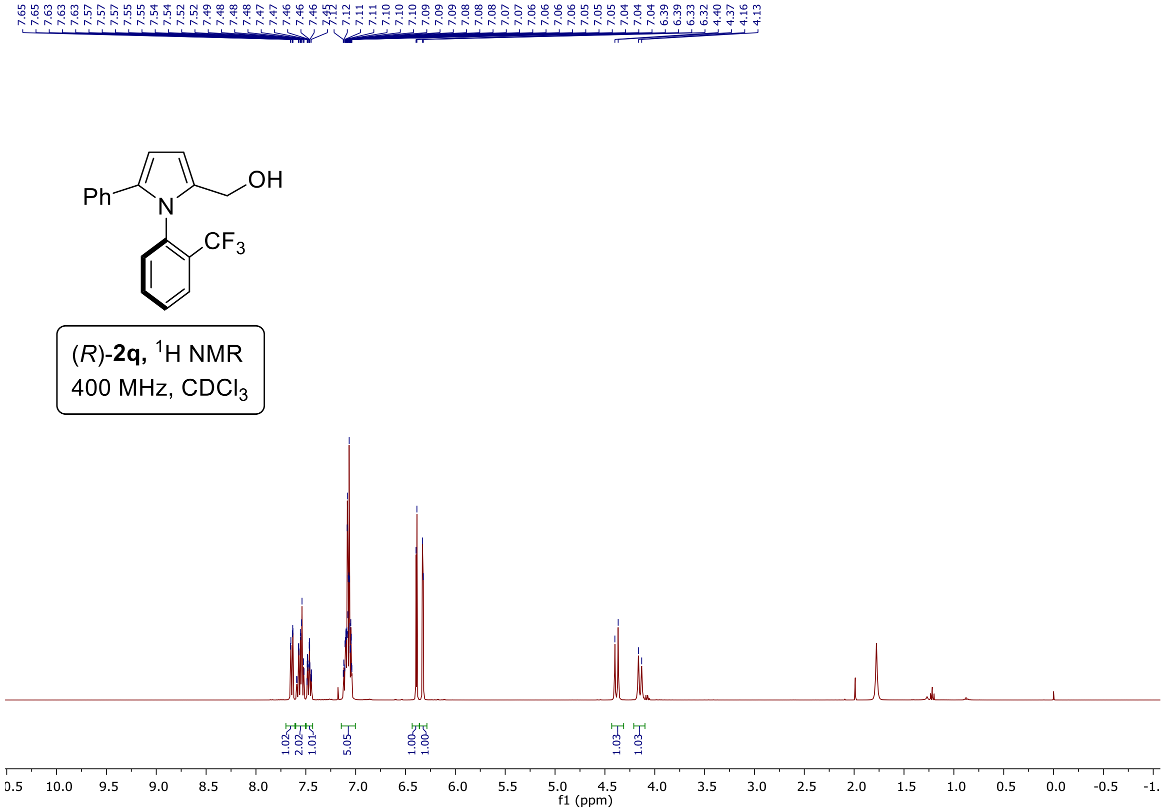

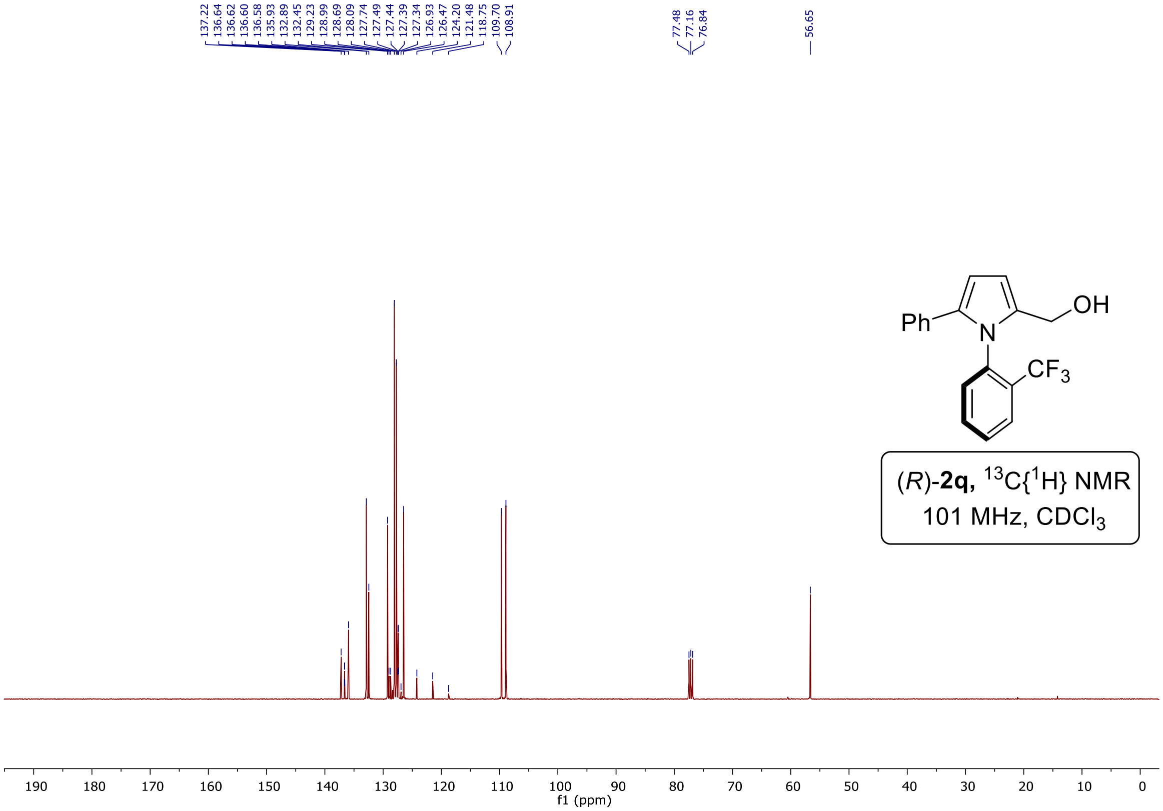

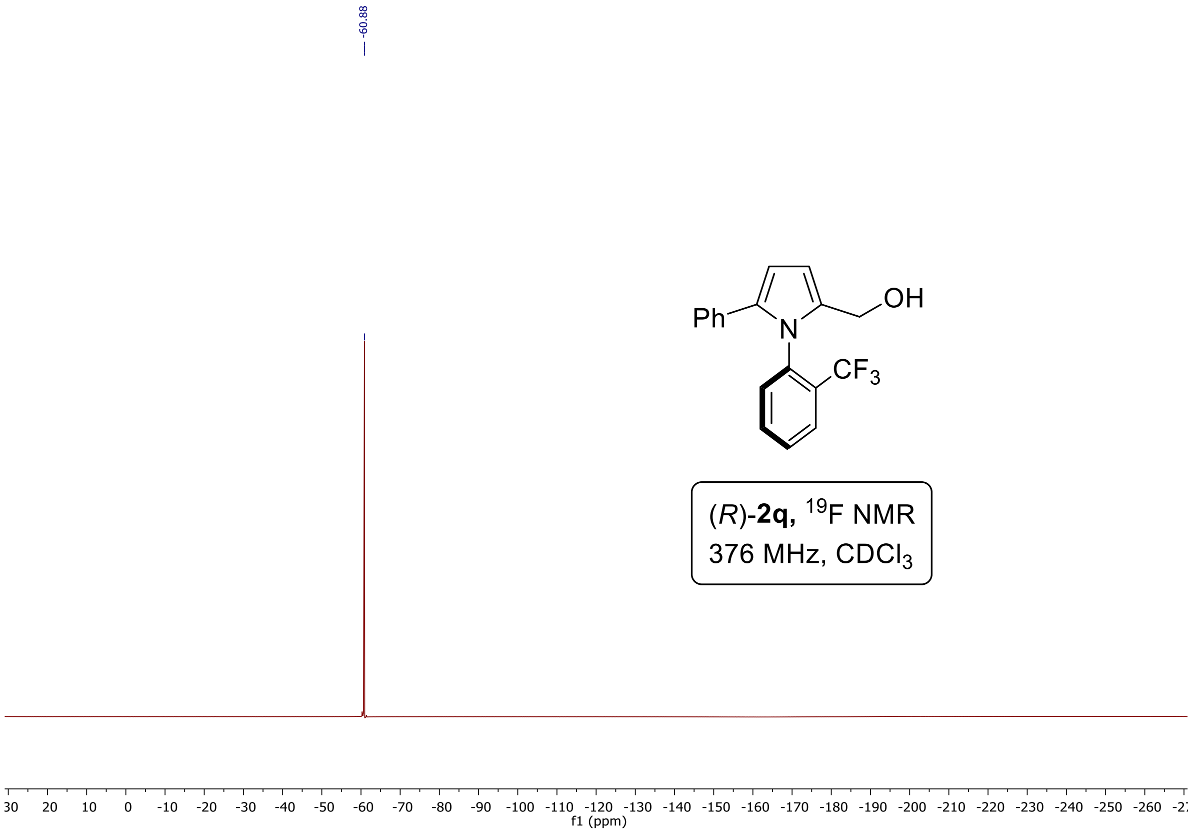

^{1}H ,$^{13}C\{^{1}H\}$ and ^{19}F NMR spectra of (*S*)-**3q**

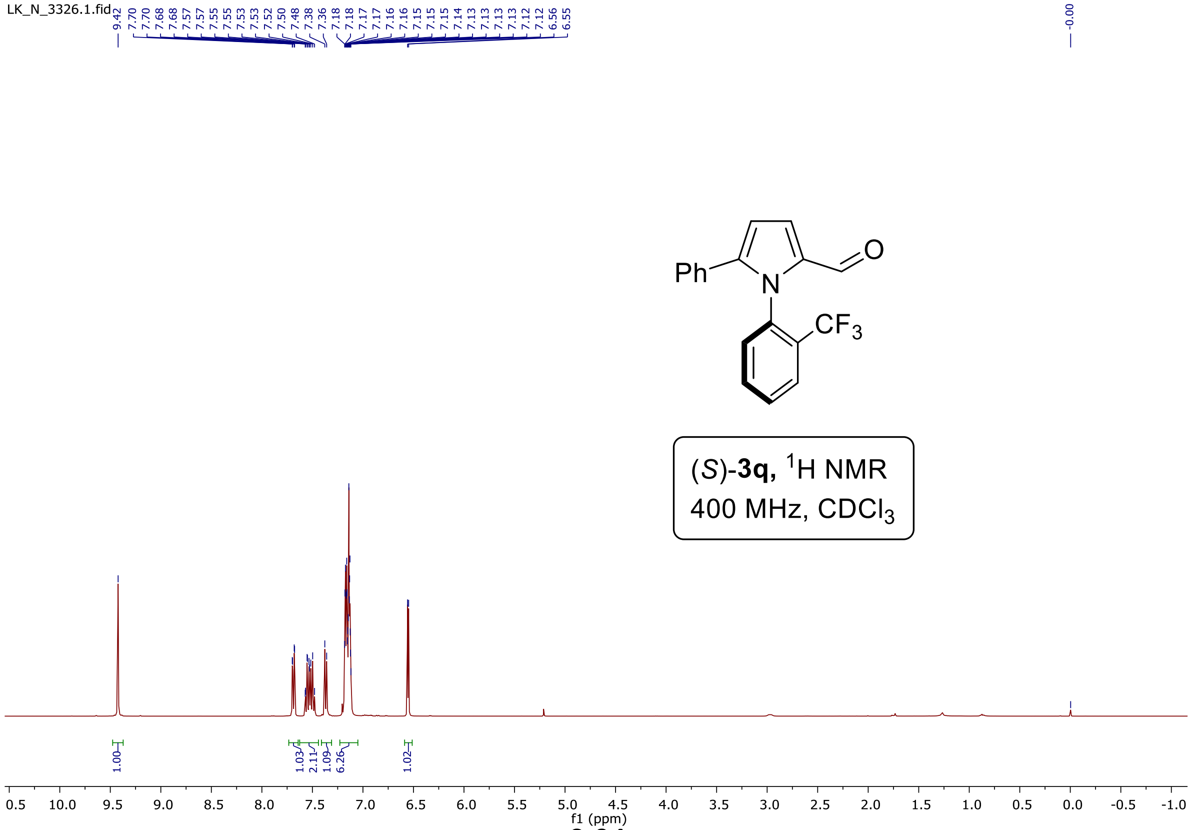

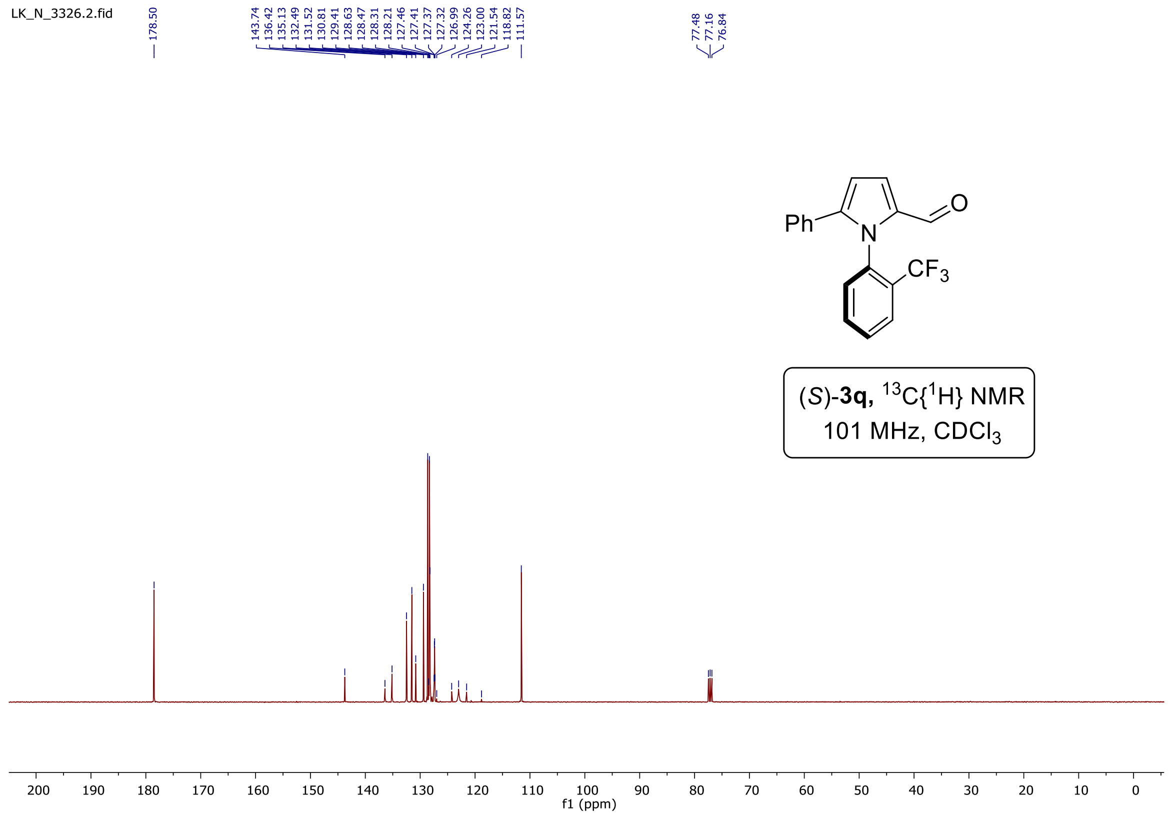
LK_N_3326.2.fid
178.50
143.74
136.42
135.13
132.49
131.52
130.81
129.41
128.63
128.47
128.31
128.21
127.46
127.41
127.37
127.32
126.99
124.26
123.00
121.54
118.82
111.57
77.48
77.16
76.84
Ph
N
O
CF_3
(*S*)-**3q,** $^{13}C\{^{1}H\}$ NMR
101 MHz, $CDCl_3$
f1 (ppm)

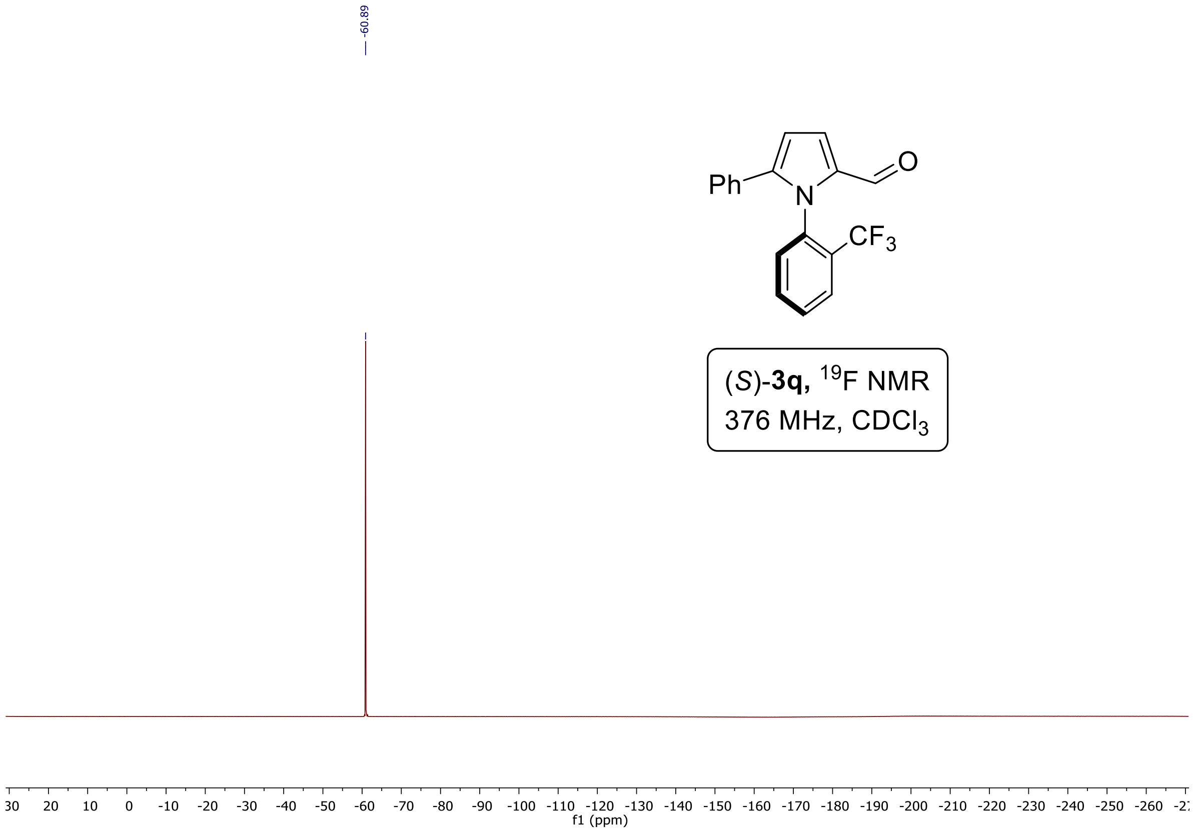
-60.89
Ph
N
O
CF_3
(*S*)-**3q,** ^{19}F NMR
376 MHz, $CDCl_3$
f1 (ppm)

^{1}H, ^{13}C{^{1}H} and ^{19}F NMR spectra of (R)-**4a**

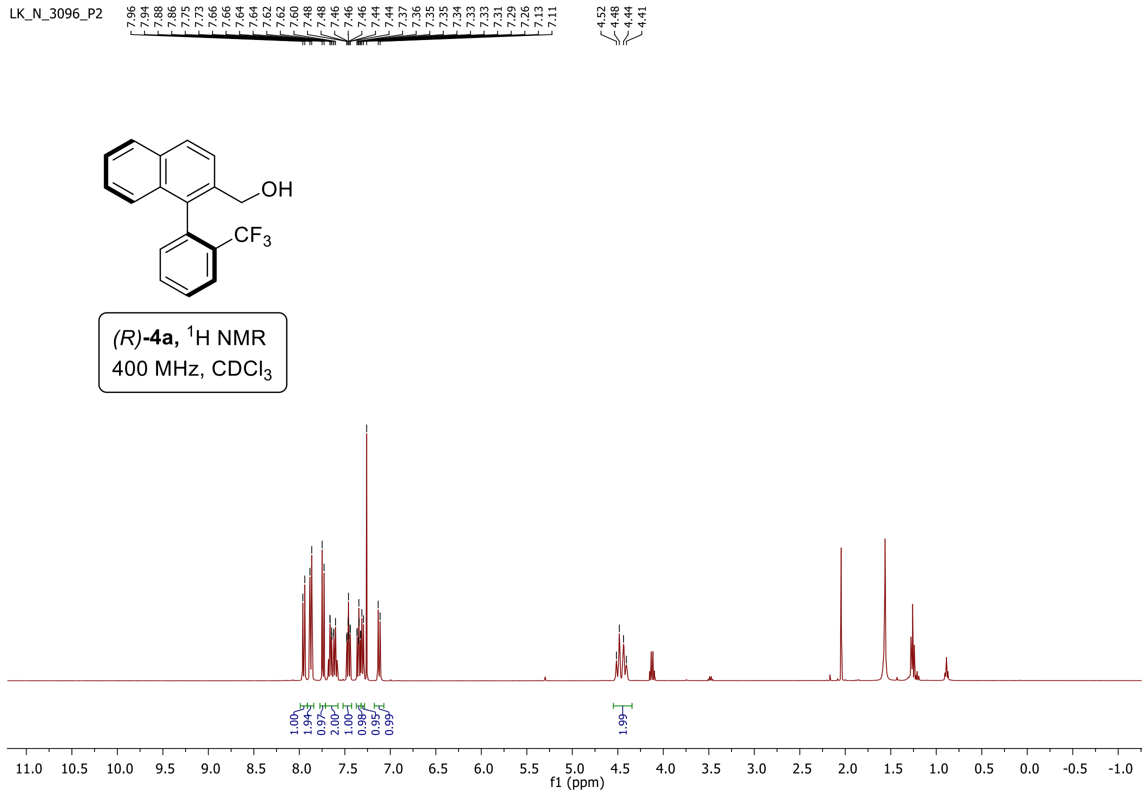

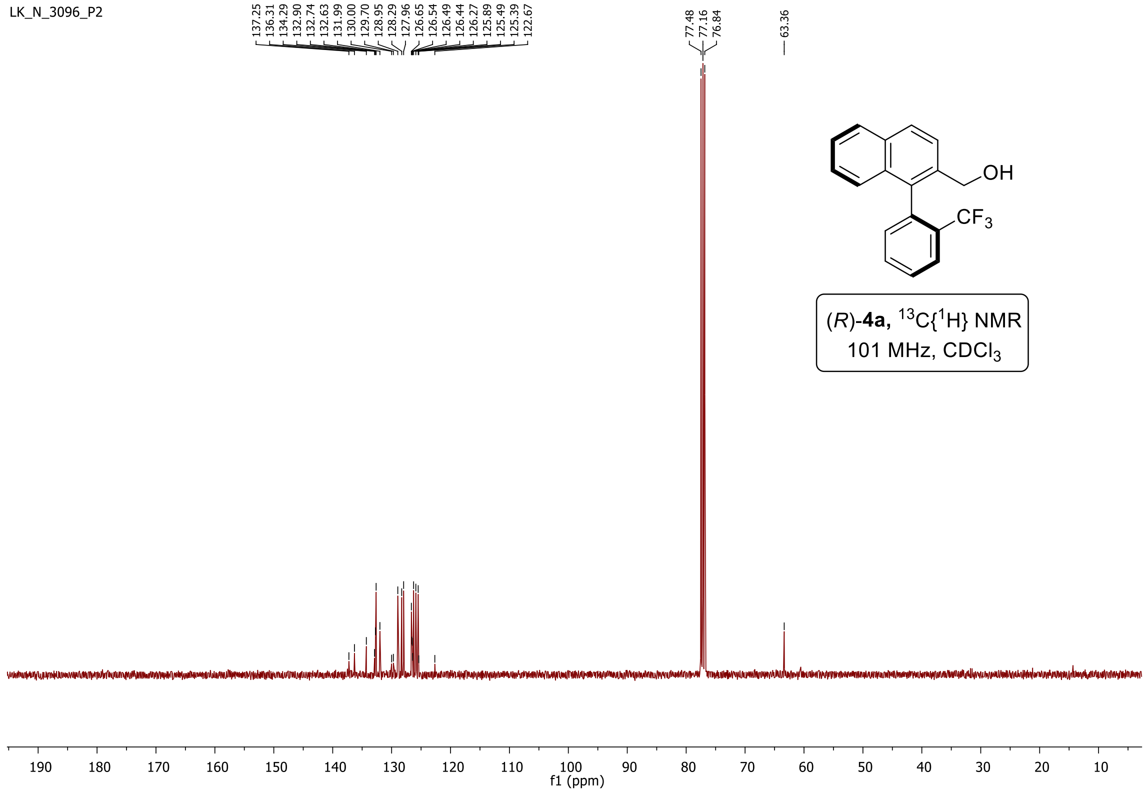

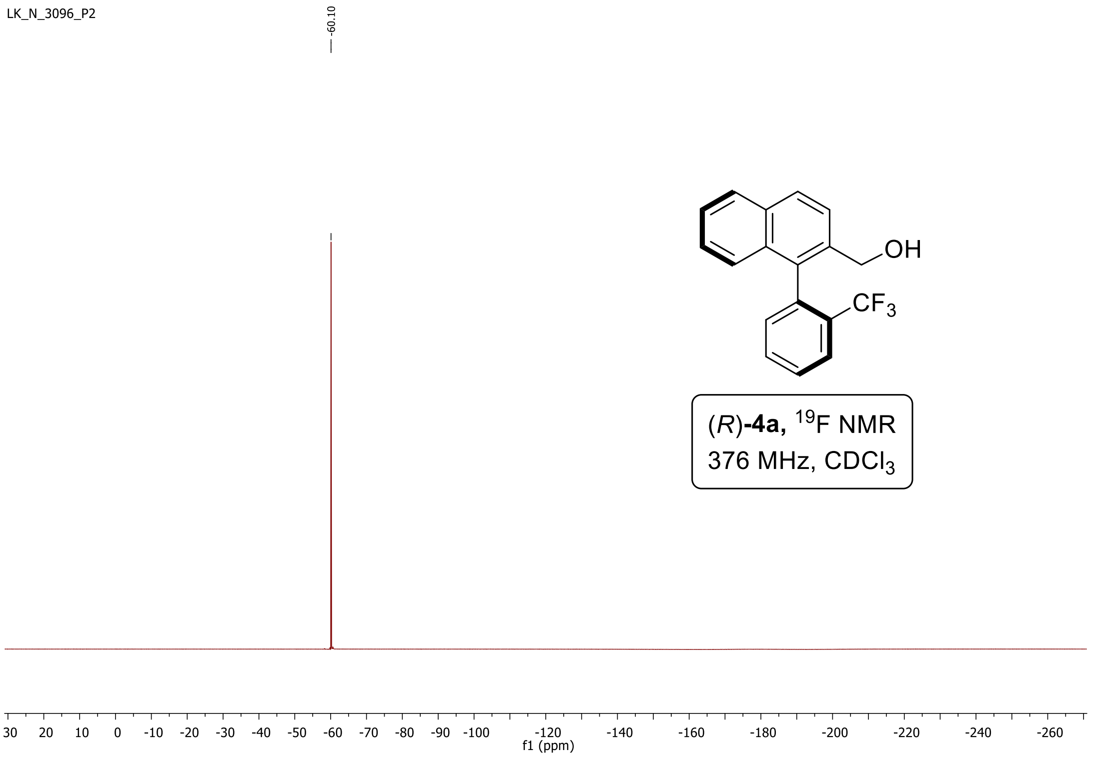

^{1}H, $^{13}C\{^{1}H\}$ and ^{19}F NMR spectra of (*S*)-**5a**

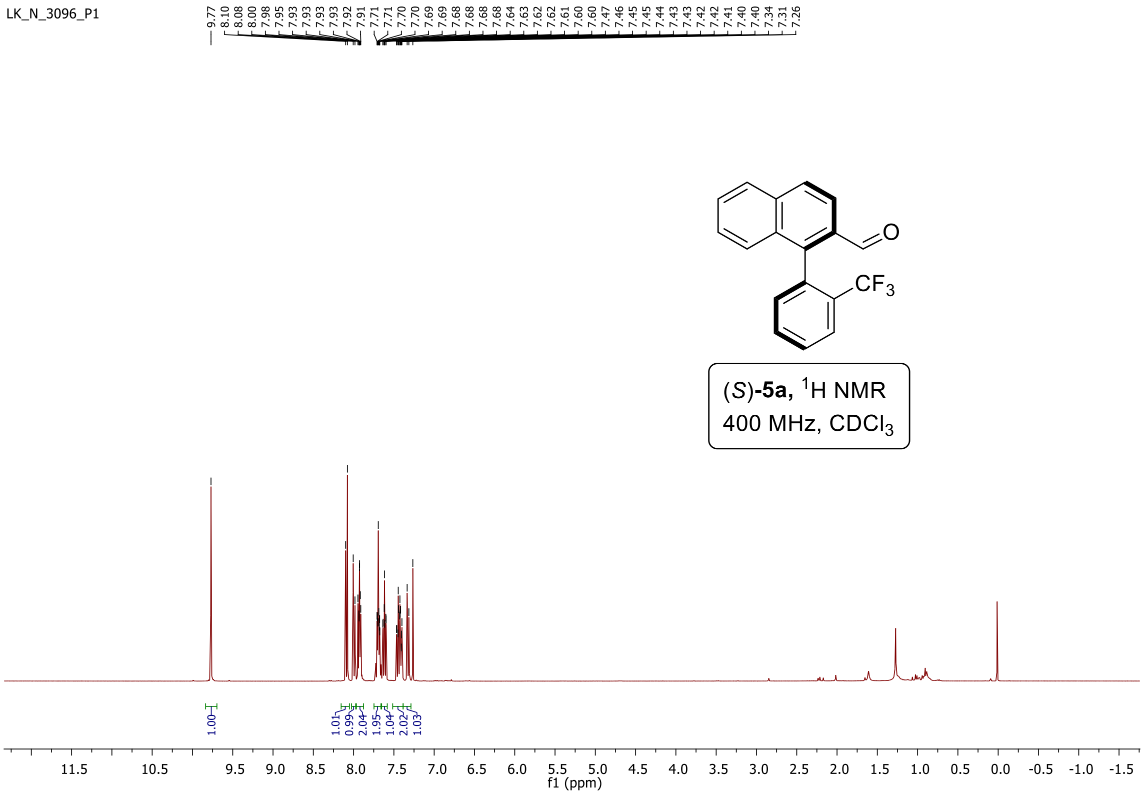

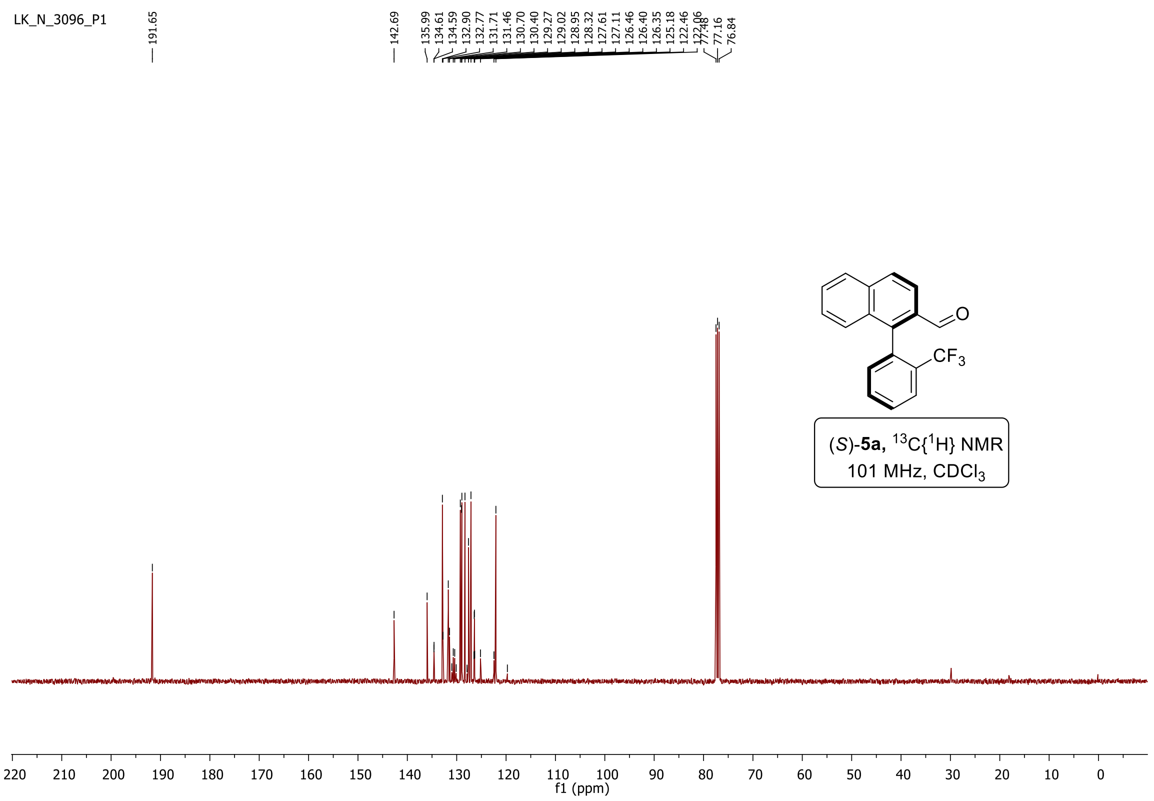
LK_N_3096_P1
191.65
142.69
135.99
134.61
134.59
132.90
132.77
131.71
131.46
130.70
130.40
129.27
129.02
128.95
128.32
127.61
127.11
126.46
126.40
126.35
125.18
122.46
122.06
77.48
77.16
76.84
O
CF3
(S)-5a, 13C{1H} NMR
101 MHz, CDCl3
220 210 200 190 180 170 160 150 140 130 120 110 100 90 80 70 60 50 40 30 20 10 0
f1 (ppm)

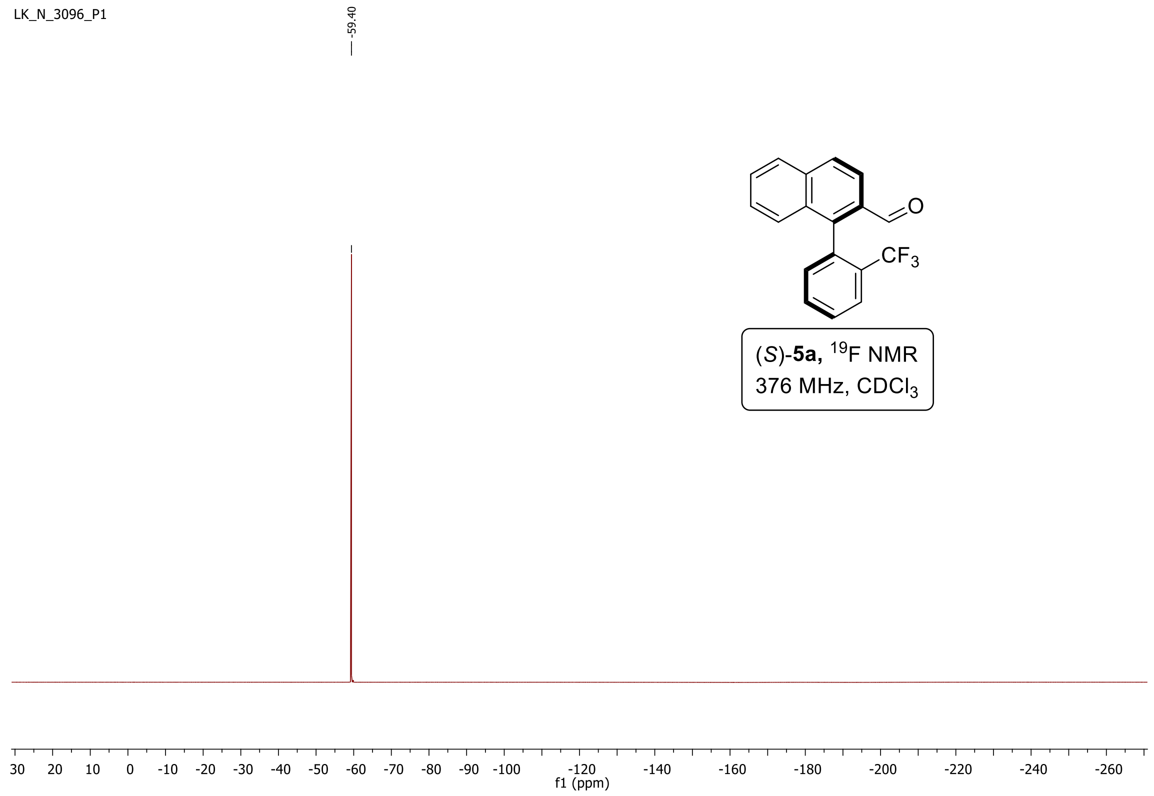
LK_N_3096_P1
-59.40
O
CF3
(S)-5a, 19F NMR
376 MHz, CDCl3
30 20 10 0 -10 -20 -30 -40 -50 -60 -70 -80 -90 -100 -120 -140 -160 -180 -200 -220 -240 -260
f1 (ppm)

^{1}H and $^{13}C\{^{1}H\}$ NMR spectra of (R)-**4b**

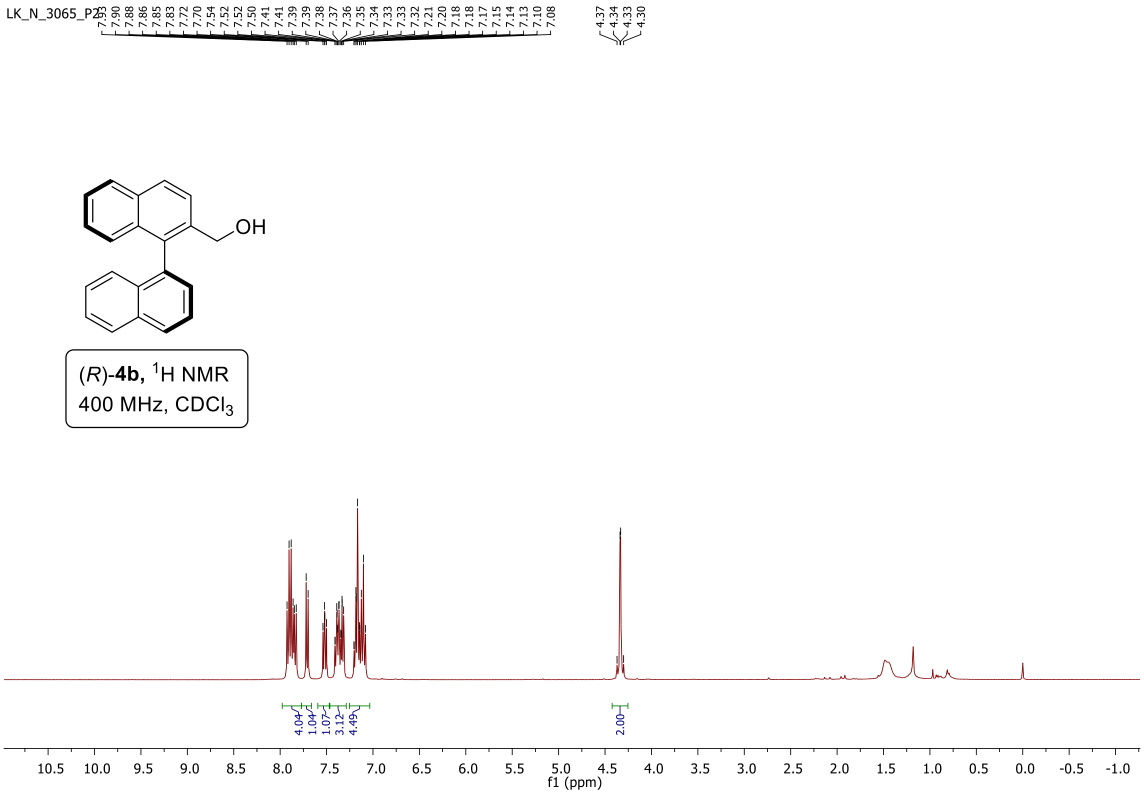

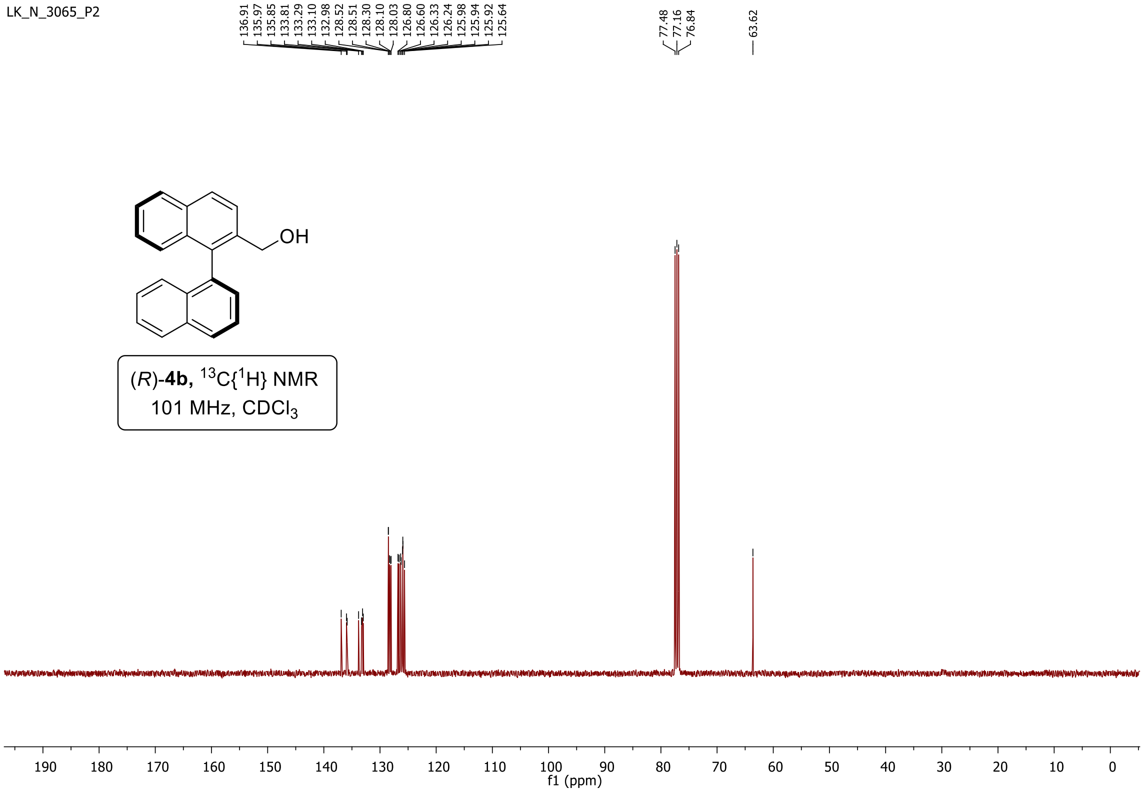

^1H and $^{13}C\{^1H\}$ NMR spectra of (S)-**5b**

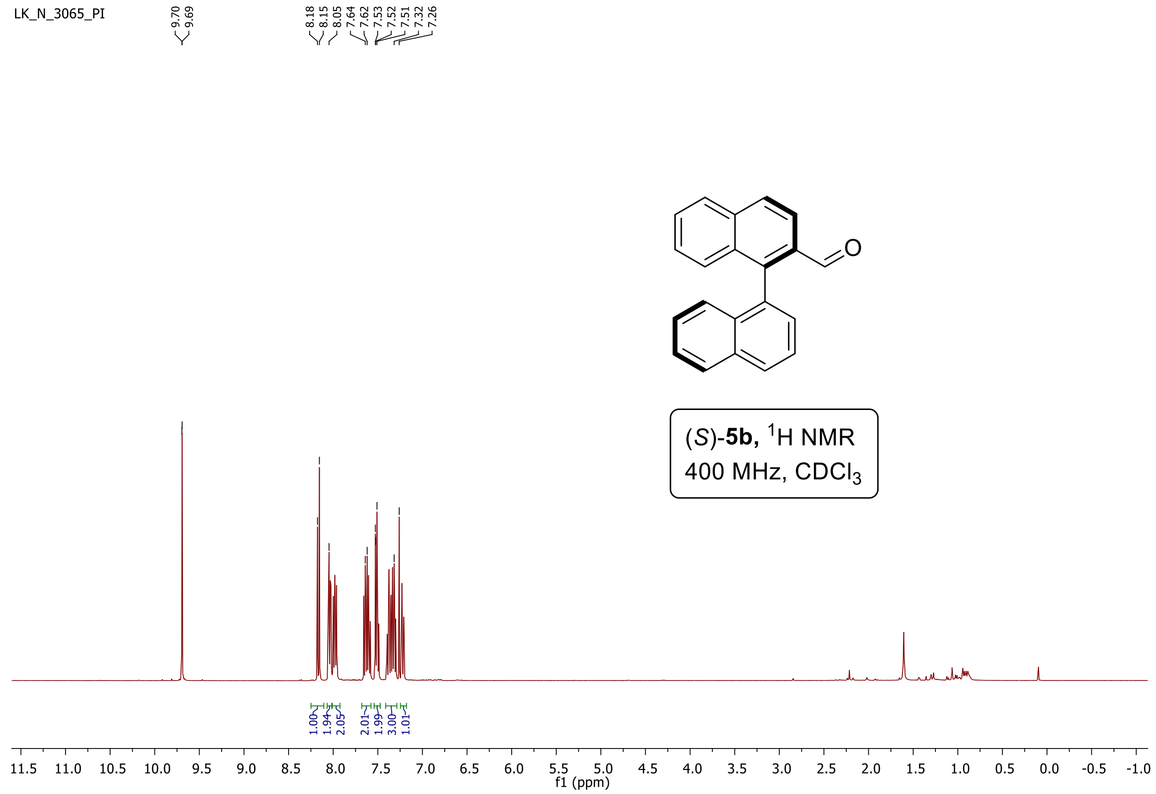

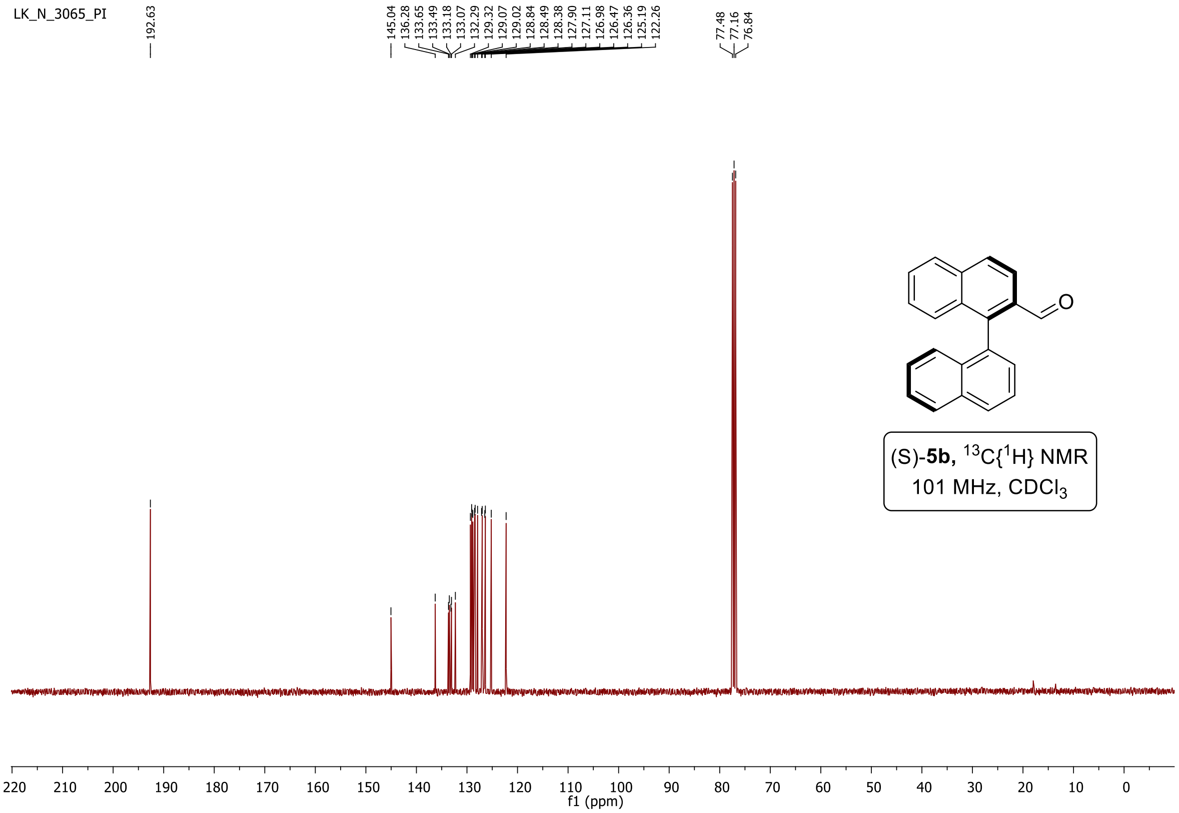

^{1}H and ^{13}C{^{1}H} NMR spectra of (*R*)-**4c**

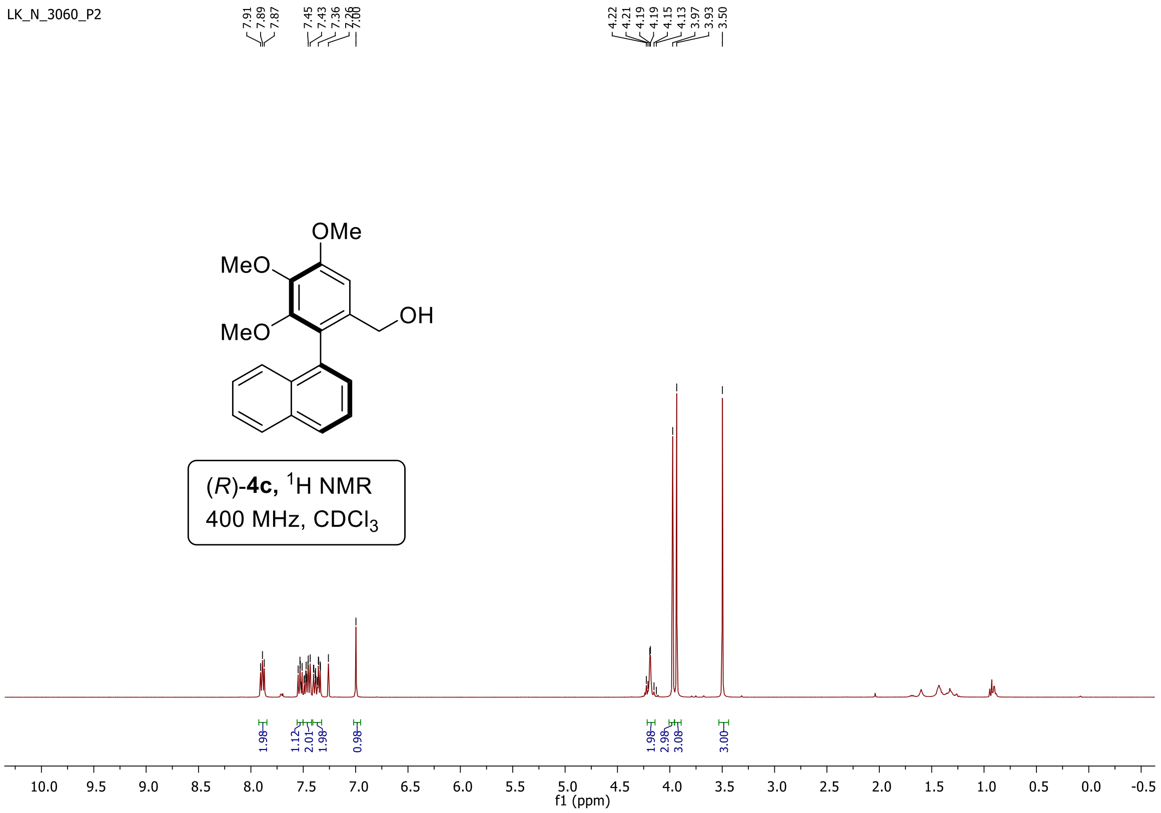

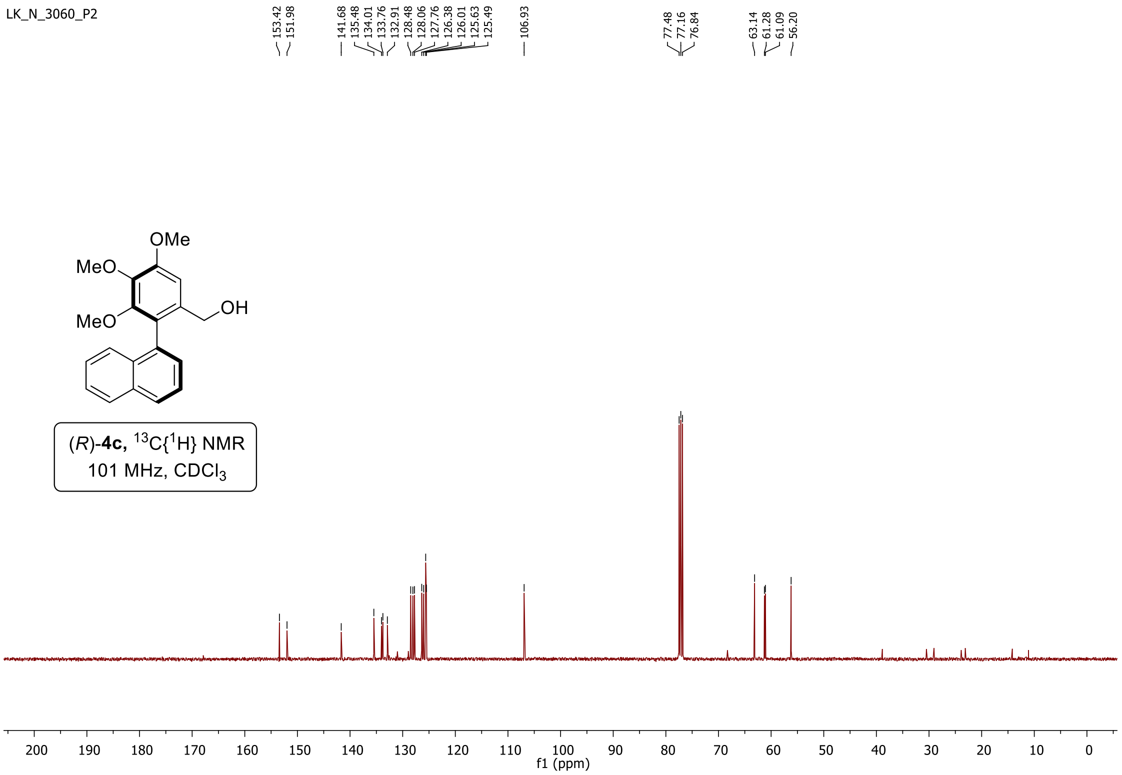

^1H and $^{13}C\{^1H\}$ NMR spectra of (*S*)-**5c**

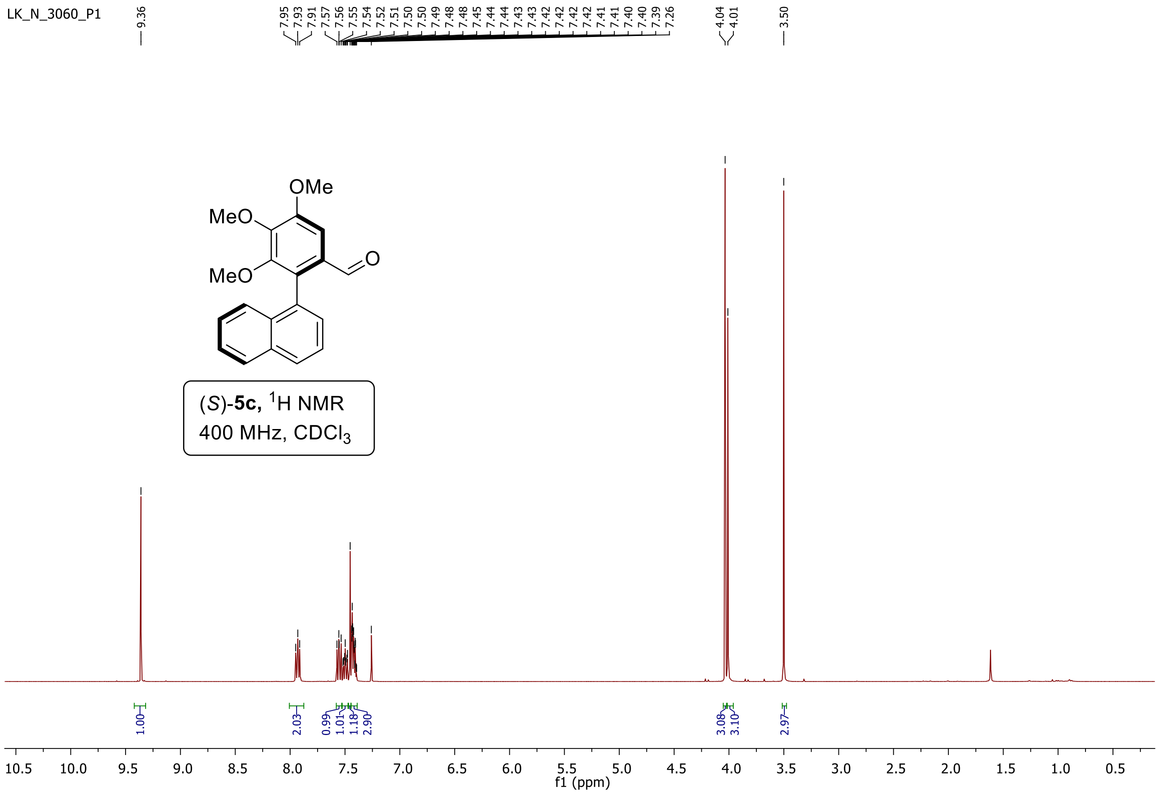

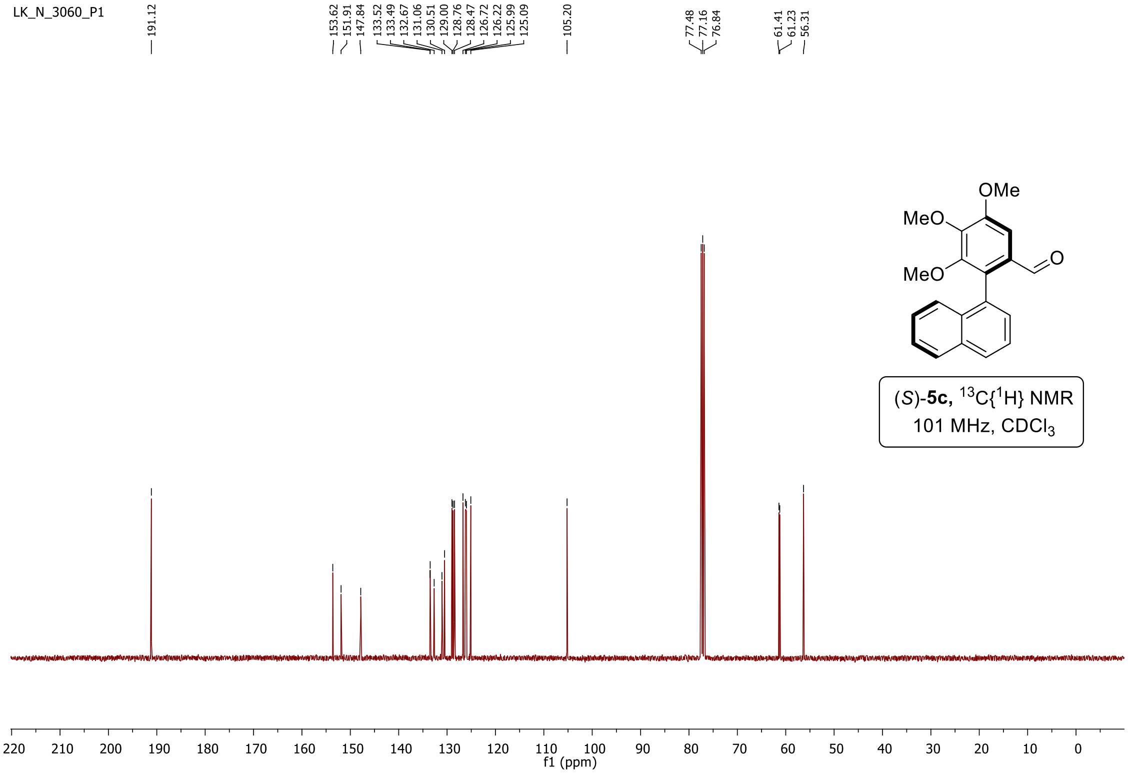

^1H and $^{13}C\{^1H\}$ NMR spectra of (*R*)-**4d**

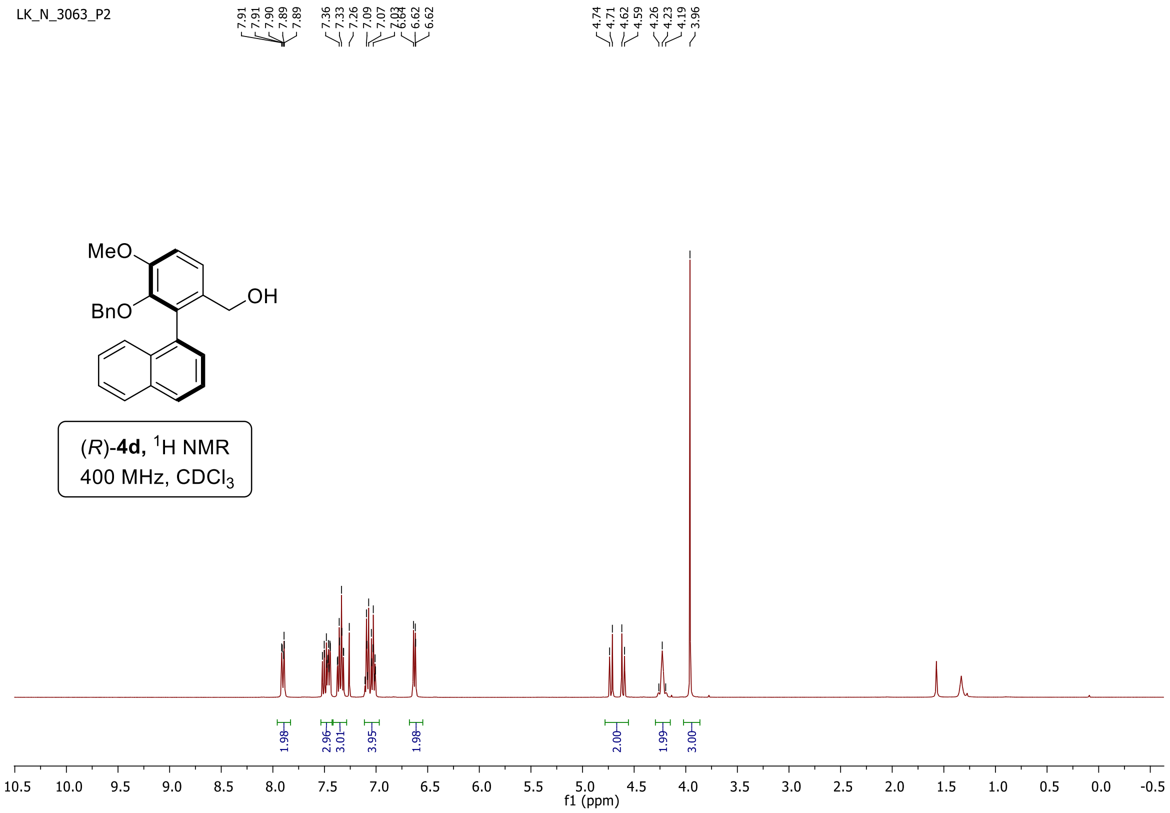

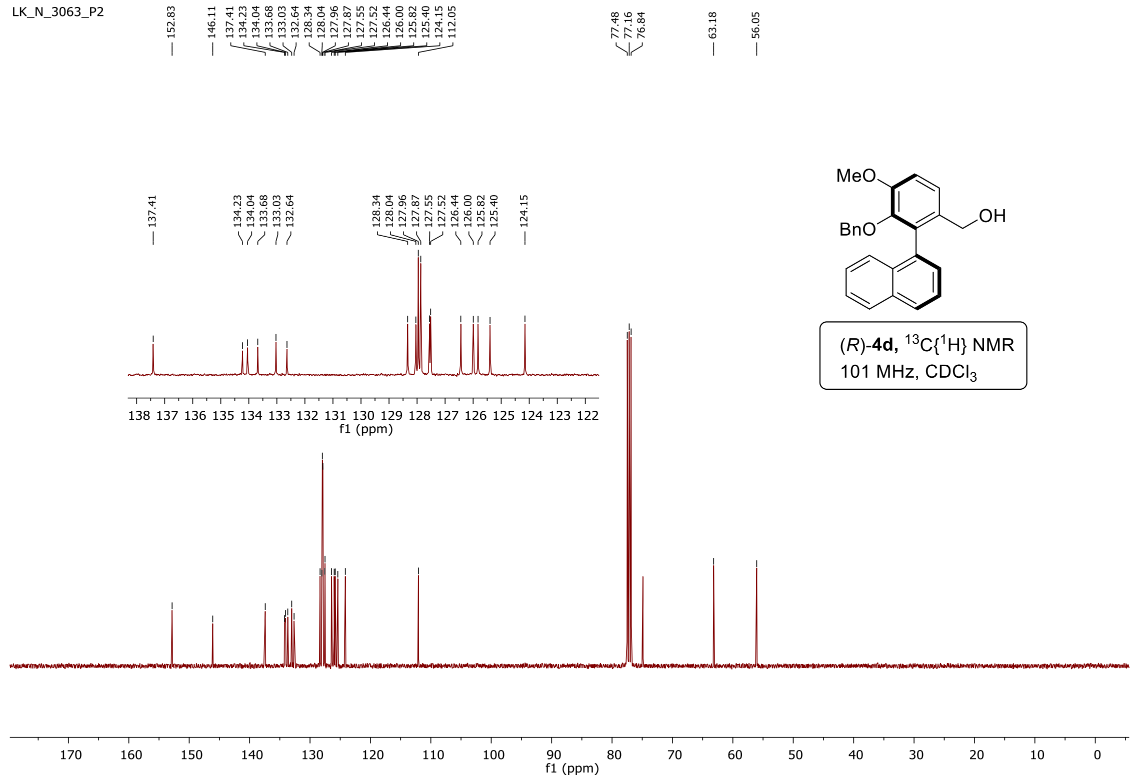

^{1}H and $^{13}C\{^{1}H\}$ NMR spectra of (*S*)-**5d**

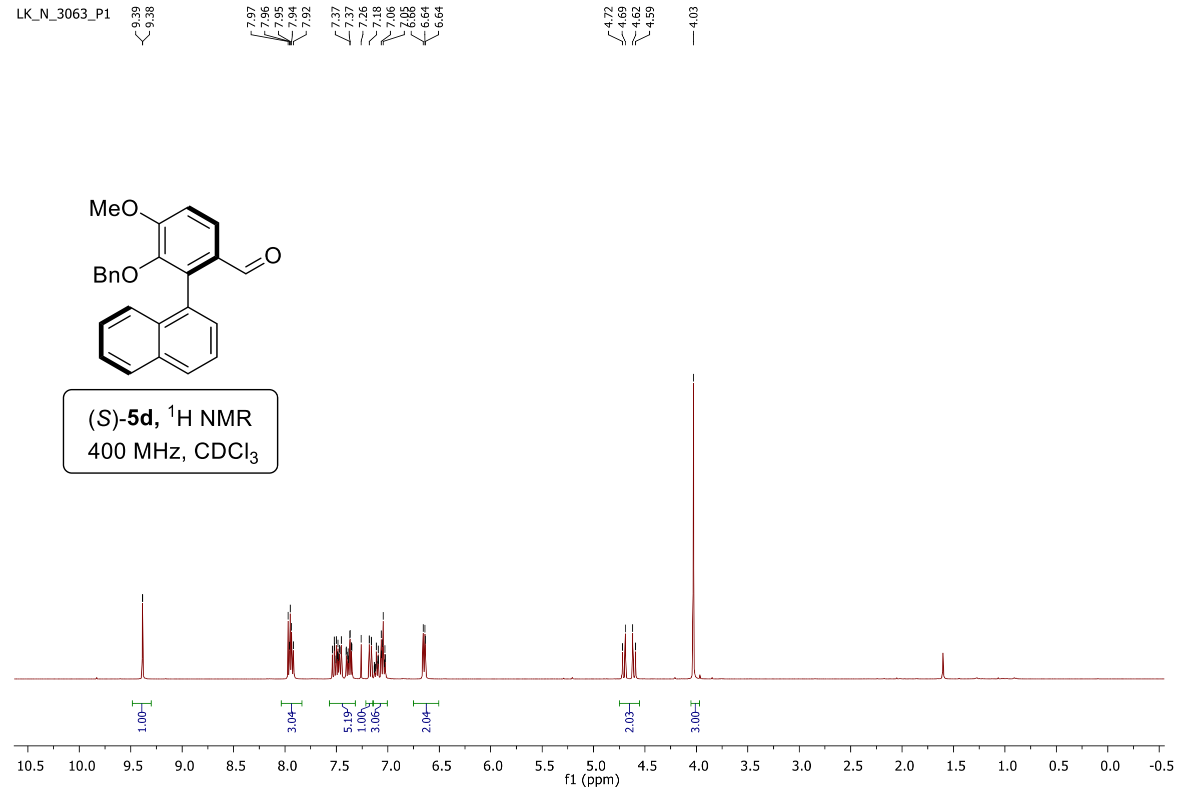

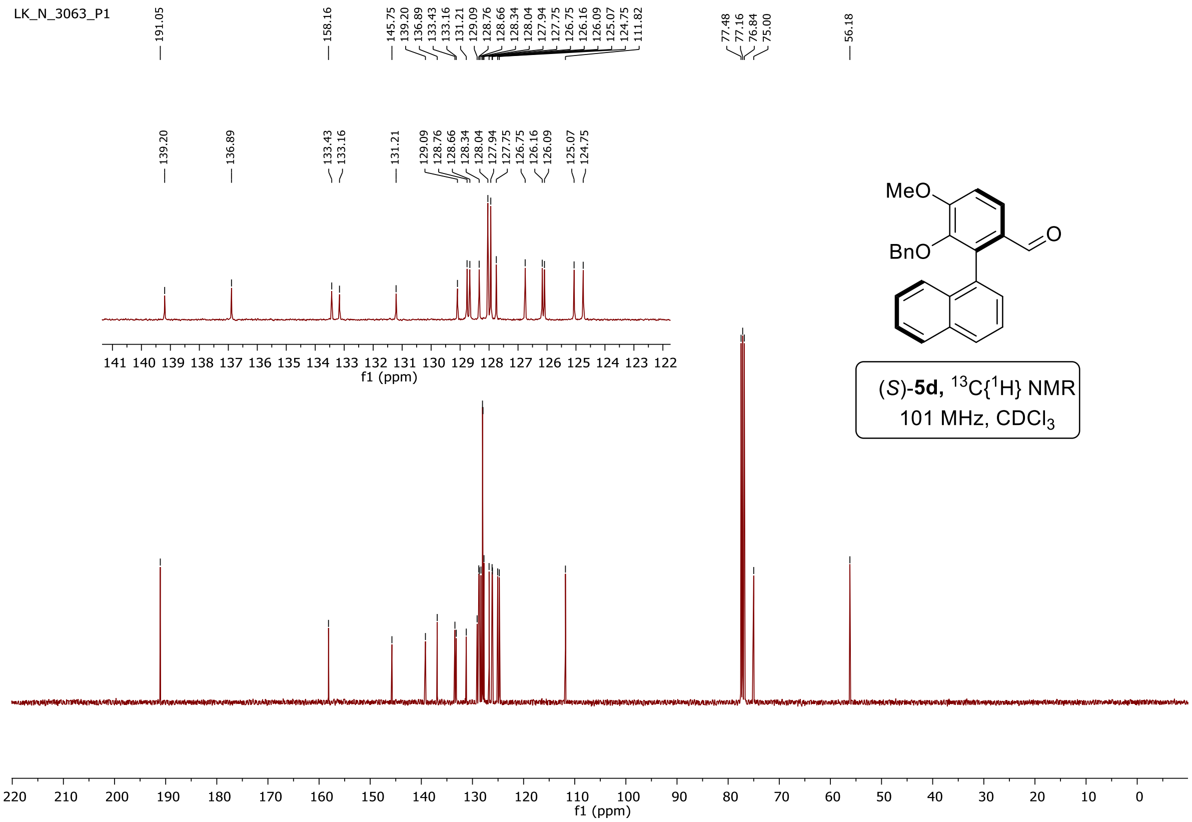

^{1}H and ^{13}C{^{1}H} NMR spectra of (*R*)-**4e**

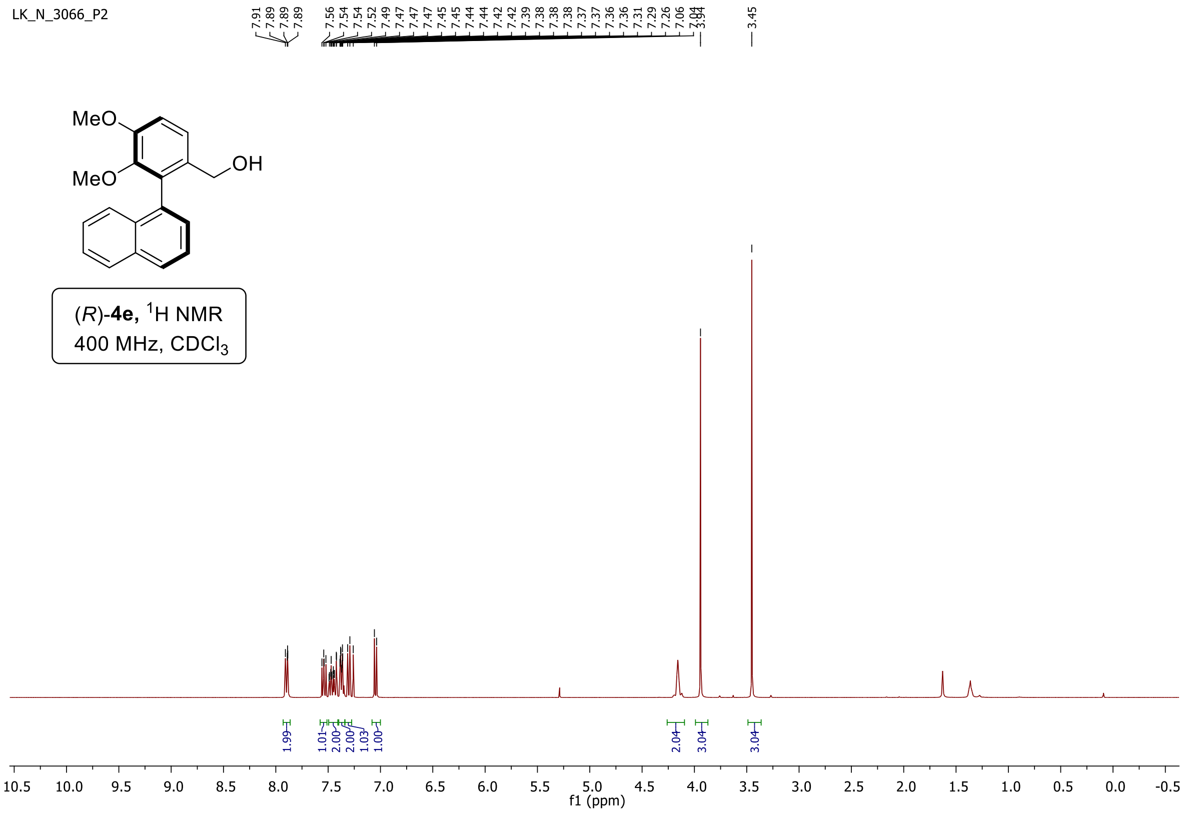

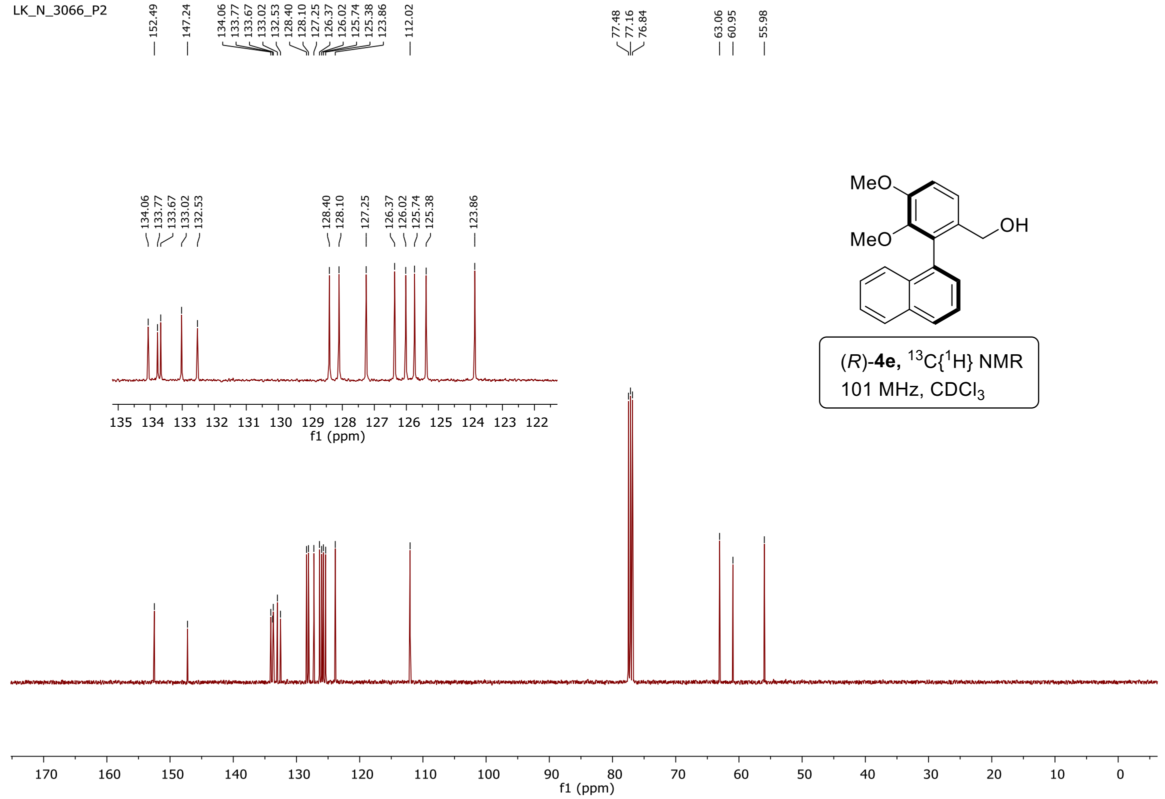

^{1}H and $^{13}C\{^{1}H\}$ NMR spectra of (*S*)-**5e**

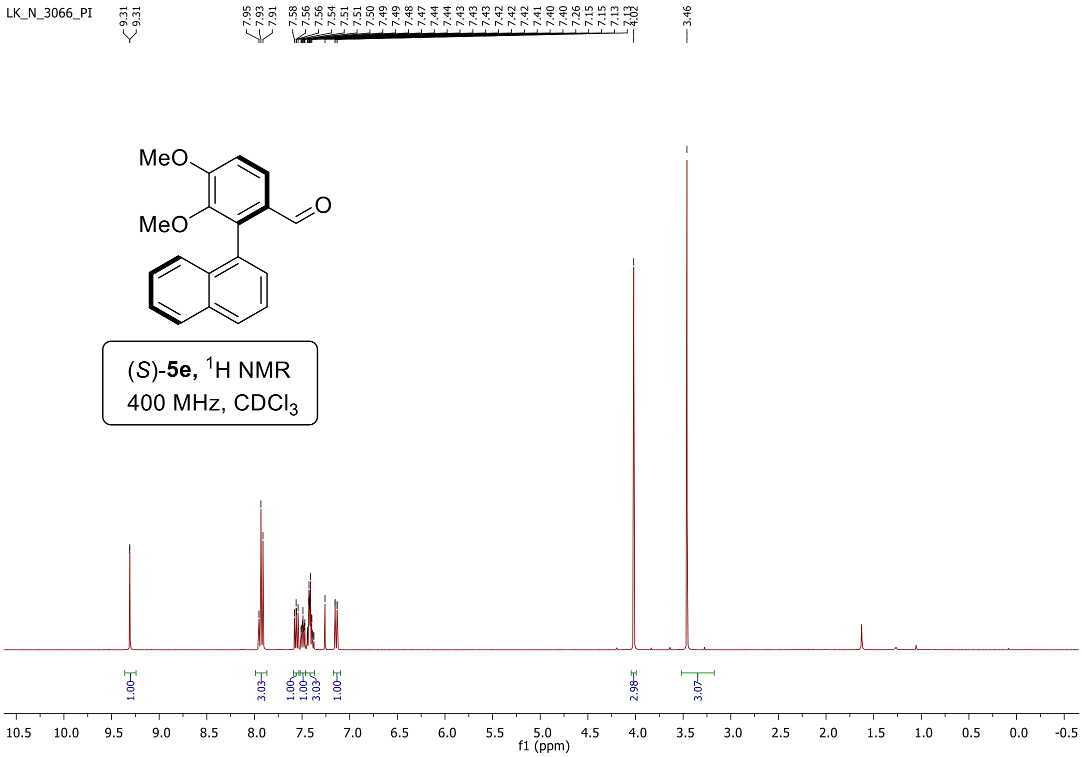

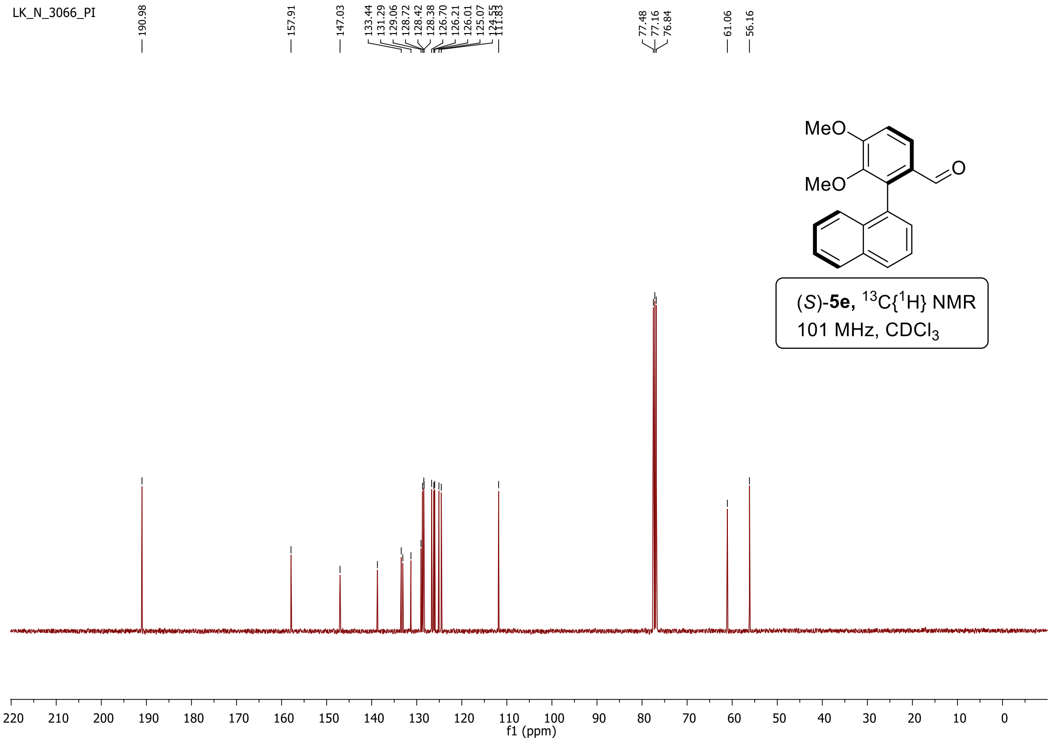

^{1}H and ^{13}C{^{1}H} NMR spectra of (*R*)-**4f**

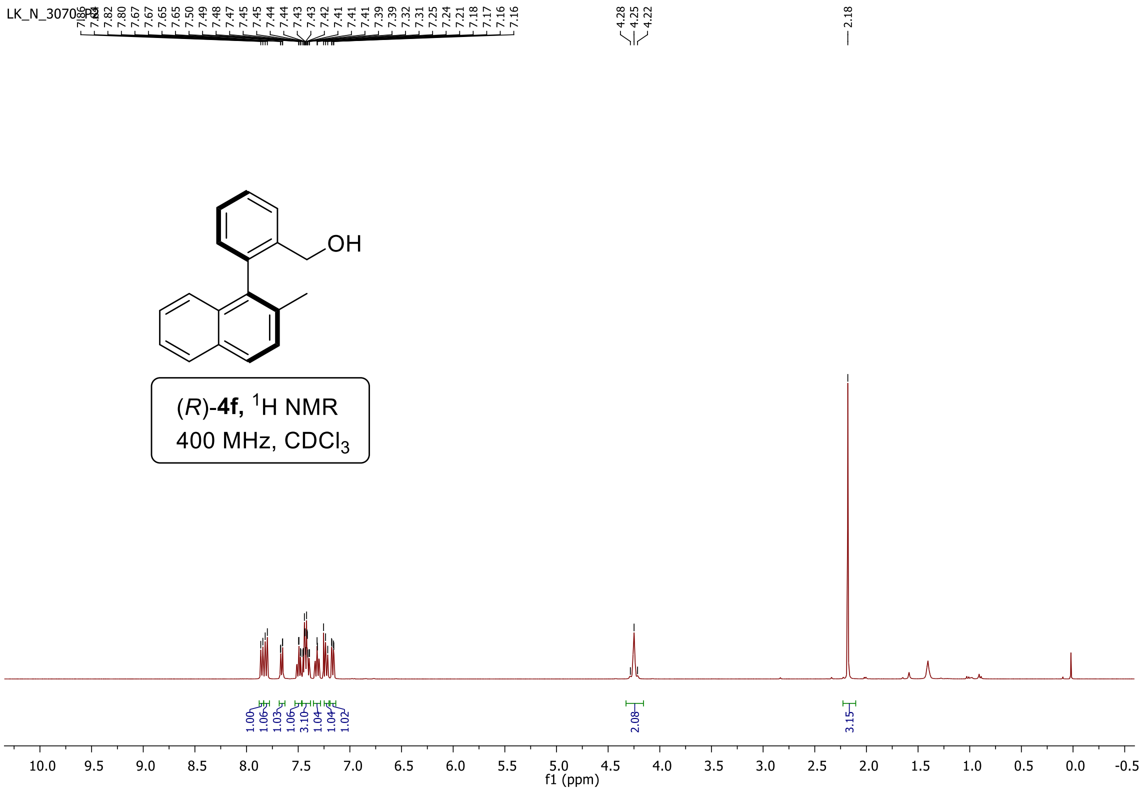

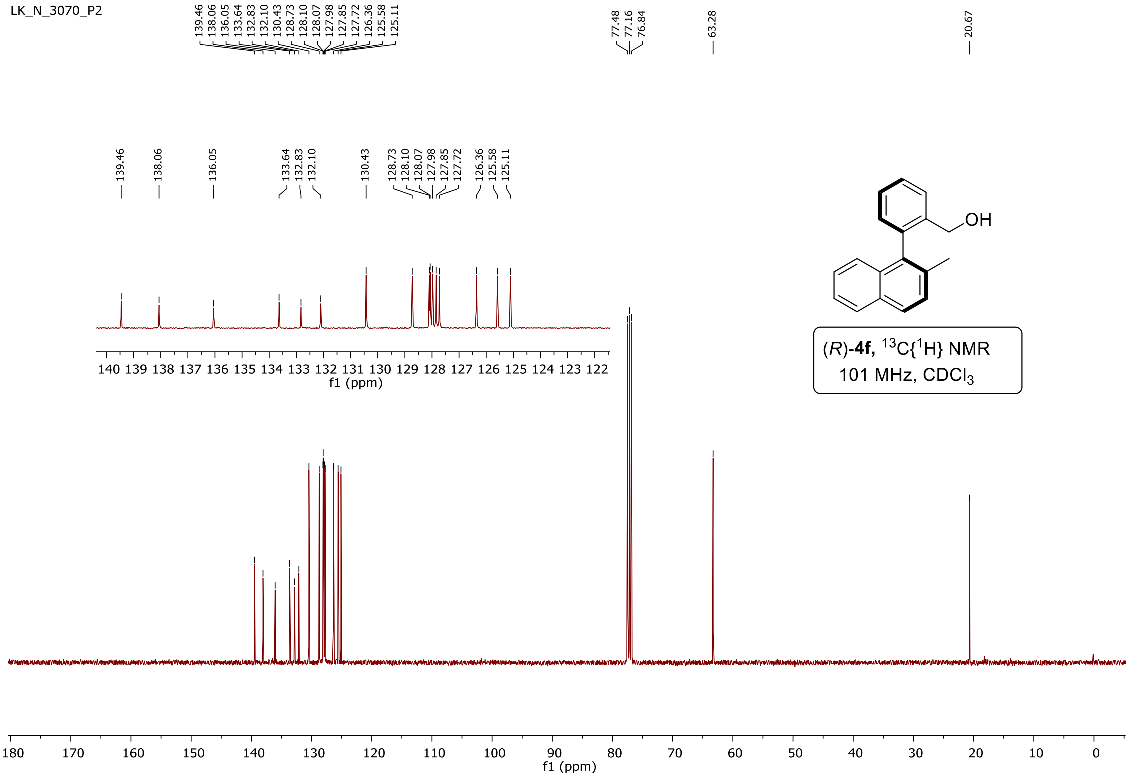

^{1}H and ^{13}C{^{1}H} NMR spectra of (*S*)-**5f**

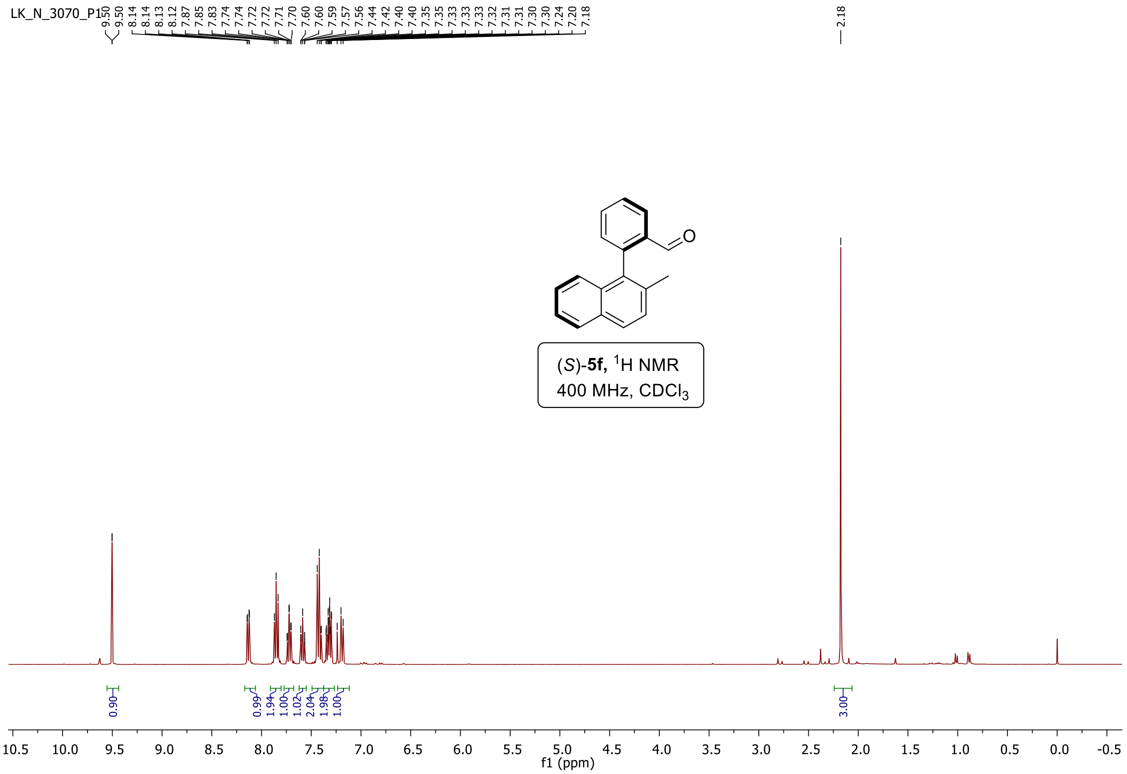

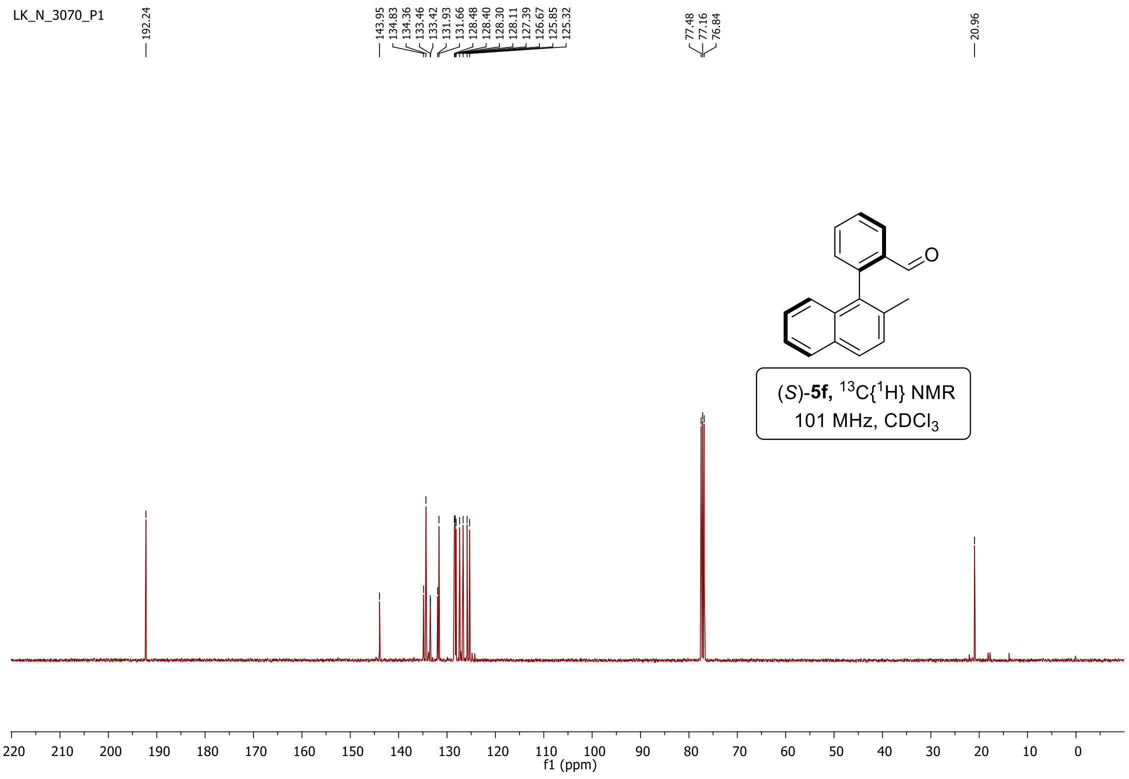

^{1}H and $^{13}C\{^{1}H\}$ NMR spectra of (*R*)-**4g**

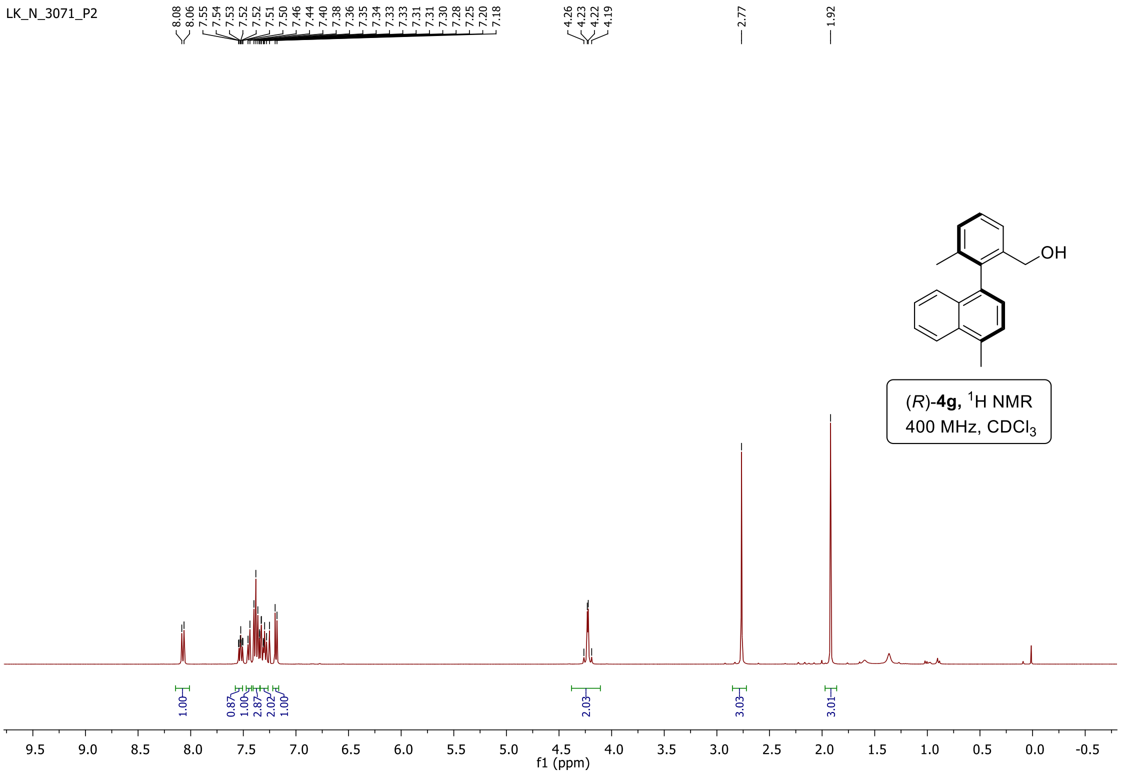

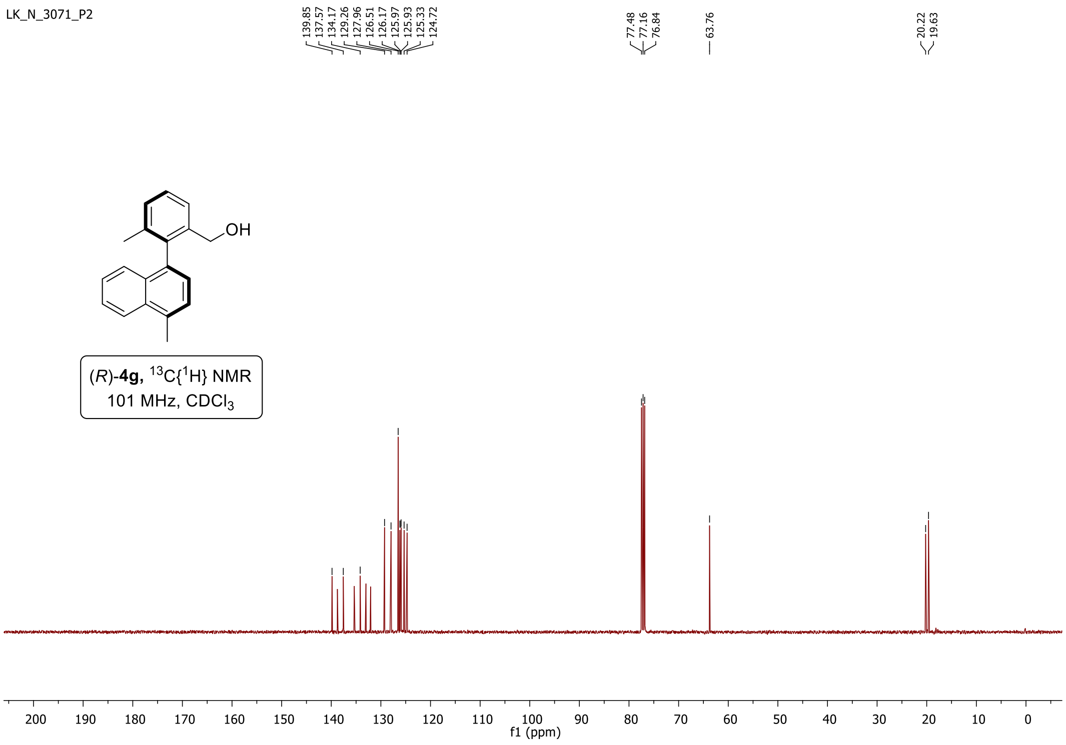

^{1}H and ^{13}C{^{1}H} NMR spectra of (*S*)-**5g**

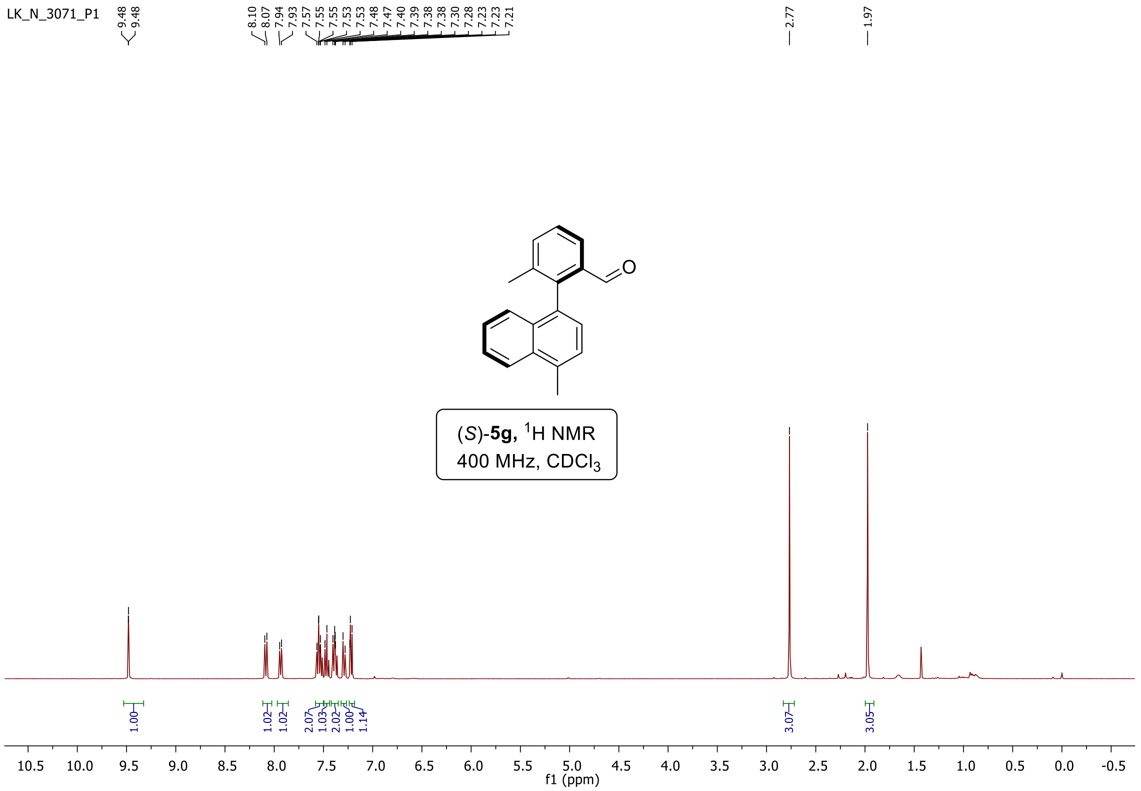

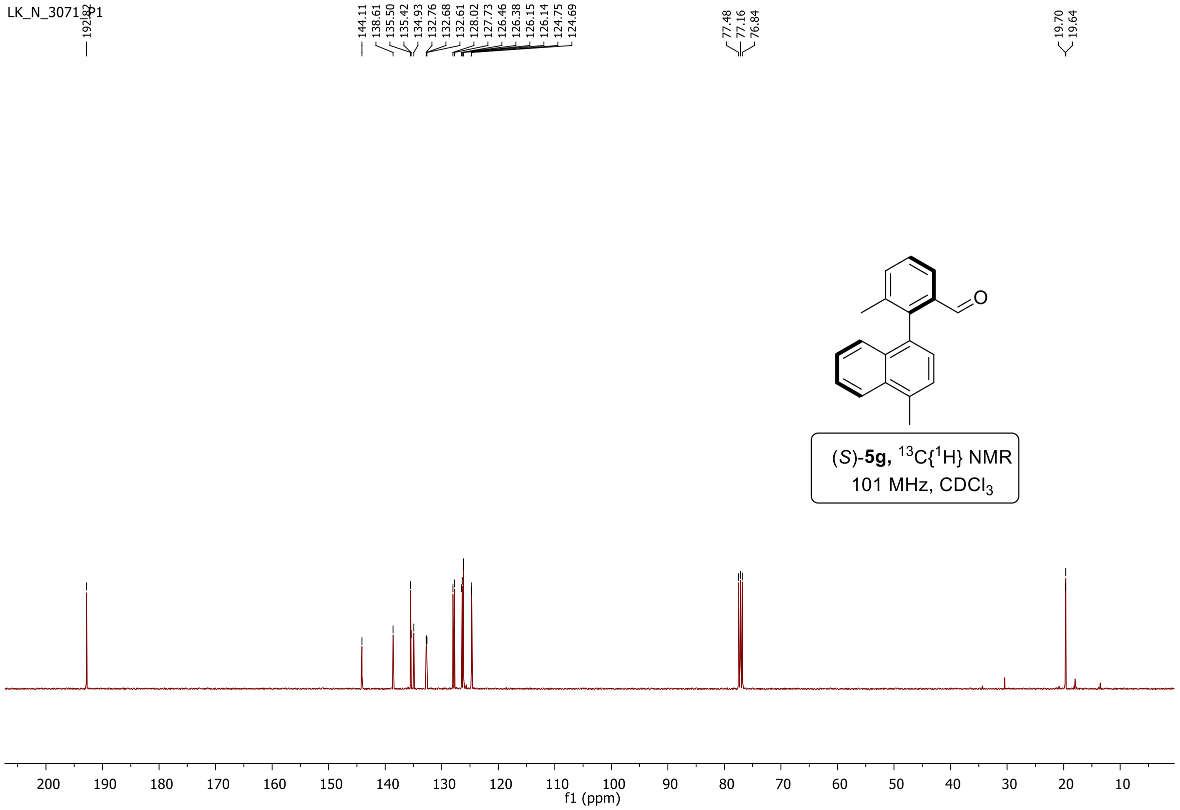

^1H, $^{13}C\{^1H\}$ and ^{19}F NMR spectra of (*R*)-**4h**

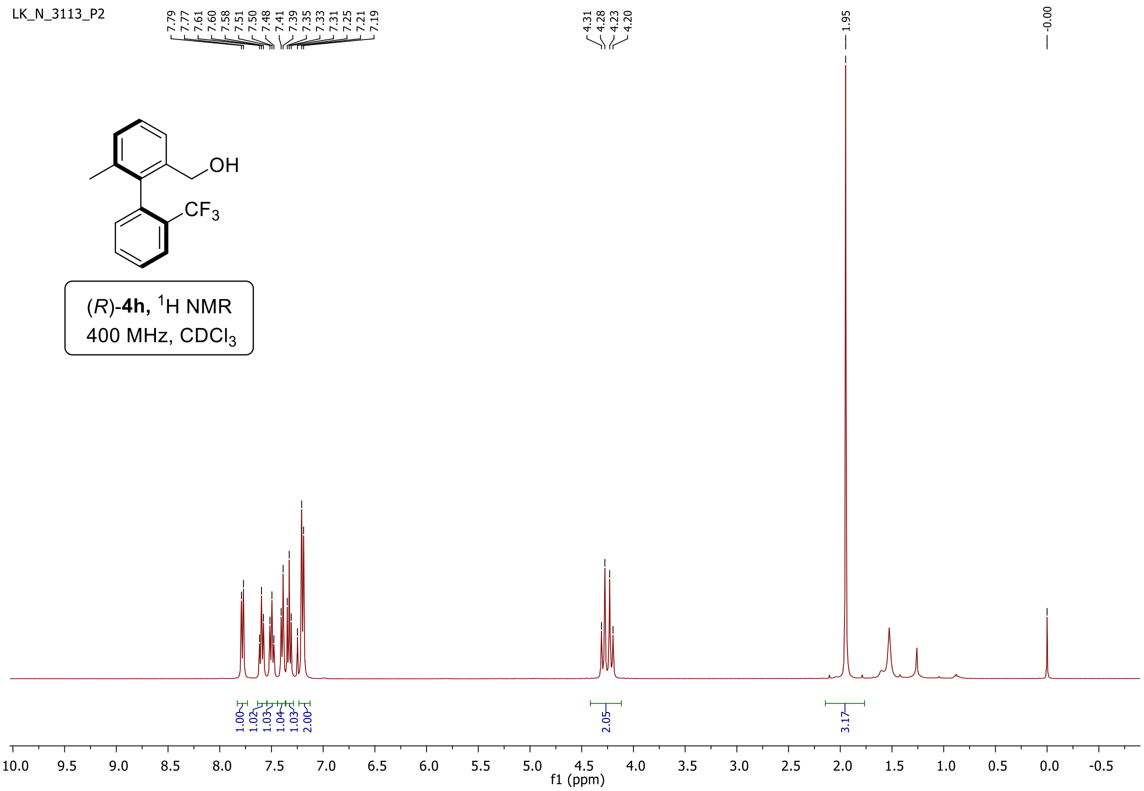

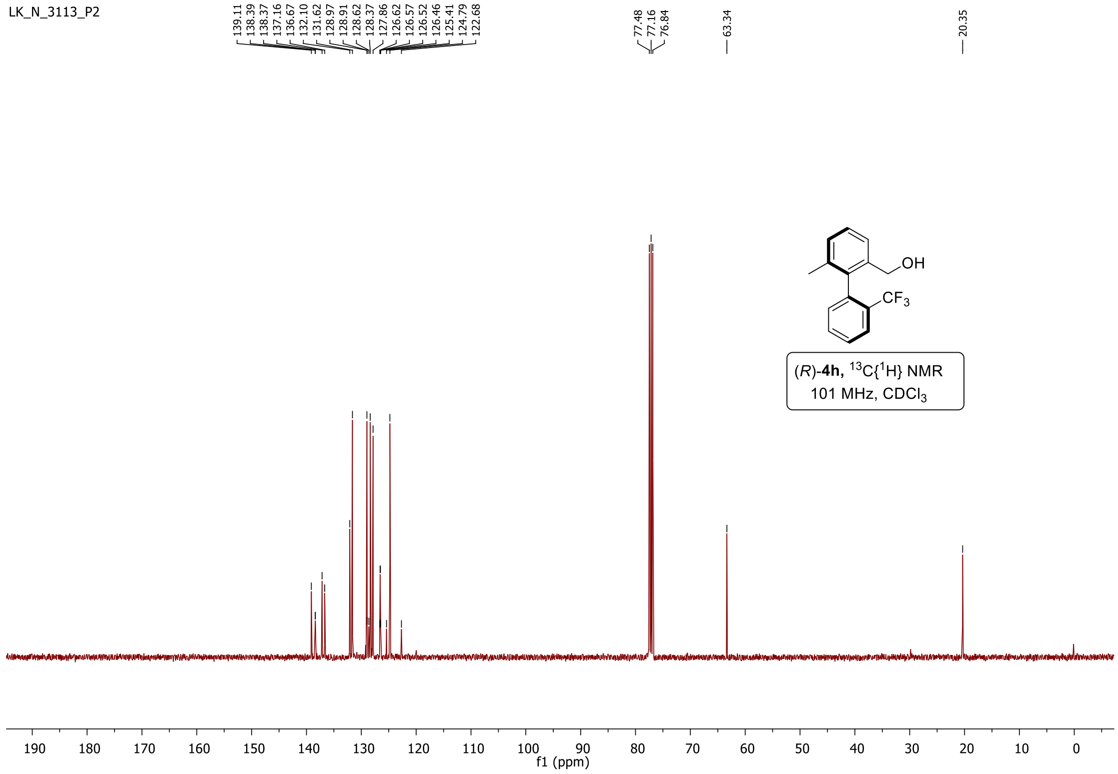

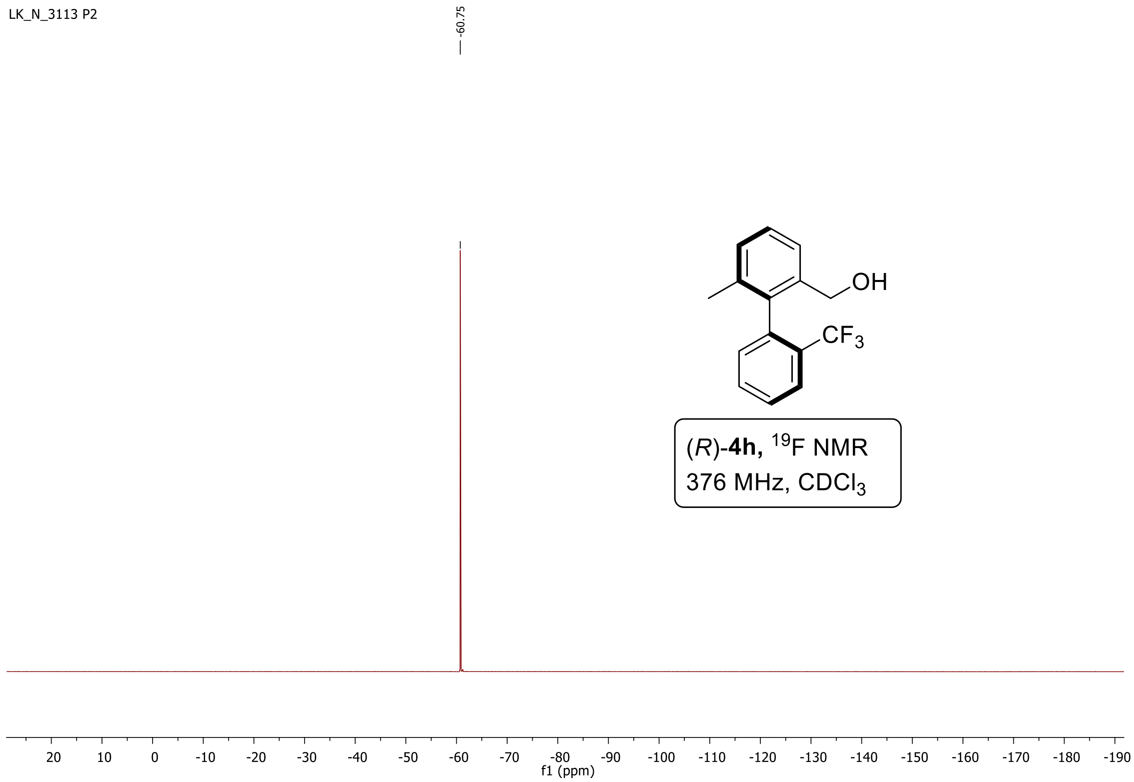

^{1}H, $^{13}C\{^{1}H\}$ and ^{19}F NMR spectra of (*S*)-**5h**

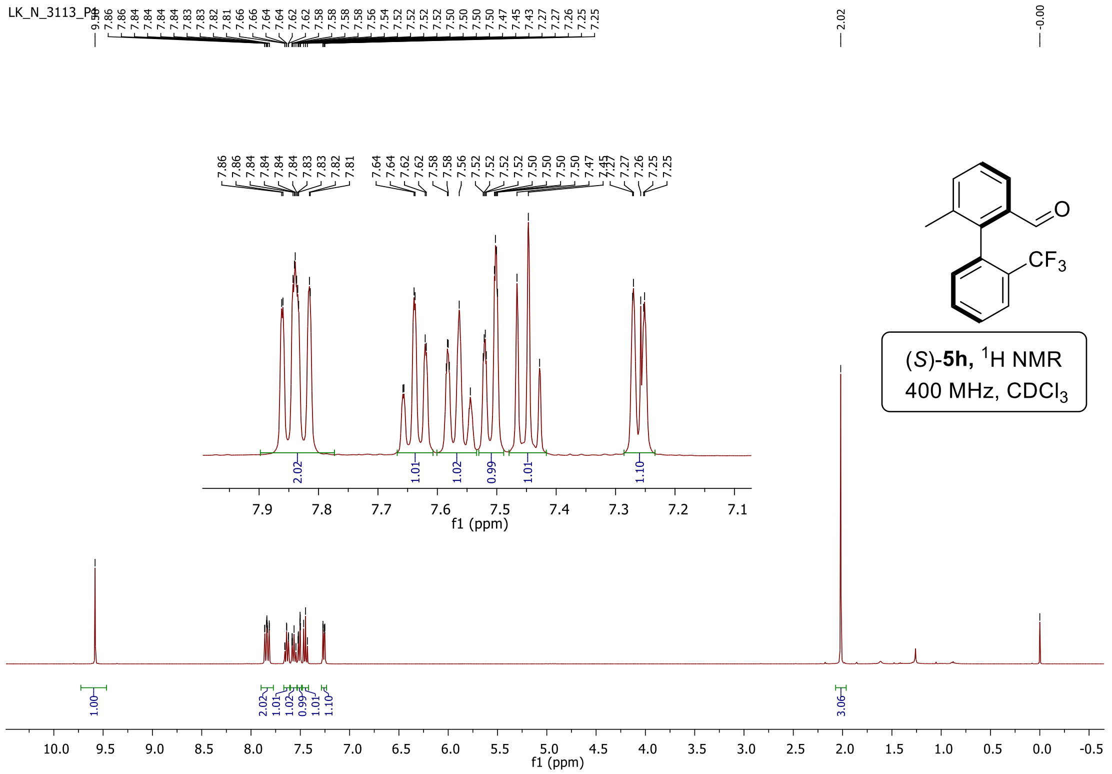

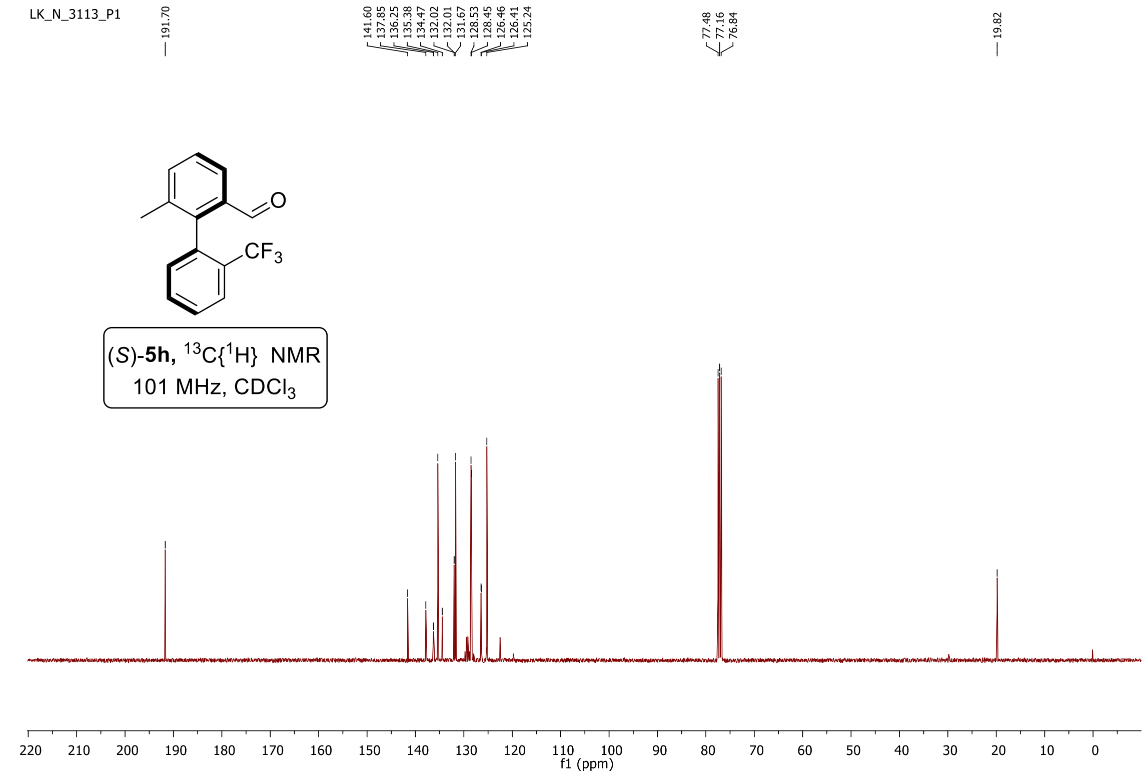
LK_N_3113_P1
191.70
141.60
137.85
136.25
135.38
134.47
132.02
132.01
131.67
128.53
128.45
126.46
126.41
125.24
77.48
77.16
76.84
19.82
CF3
O
(S)-5h, 13C{1H} NMR
101 MHz, CDCl3
f1 (ppm)

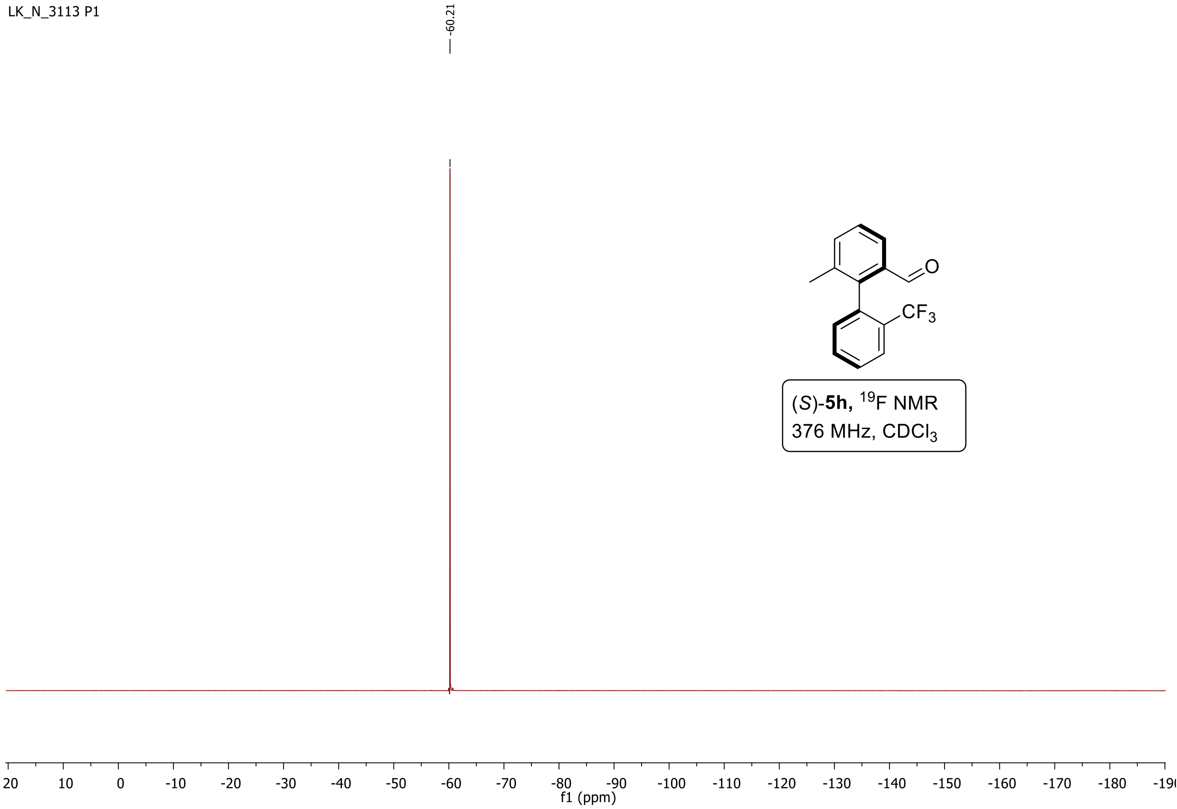
LK_N_3113 P1
-60.21
CF3
O
(S)-5h, 19F NMR
376 MHz, CDCl3
f1 (ppm)

^1H,$^{13}C\{^1H\}$ and ^{19}F NMR spectra of (*R*)-**4i**

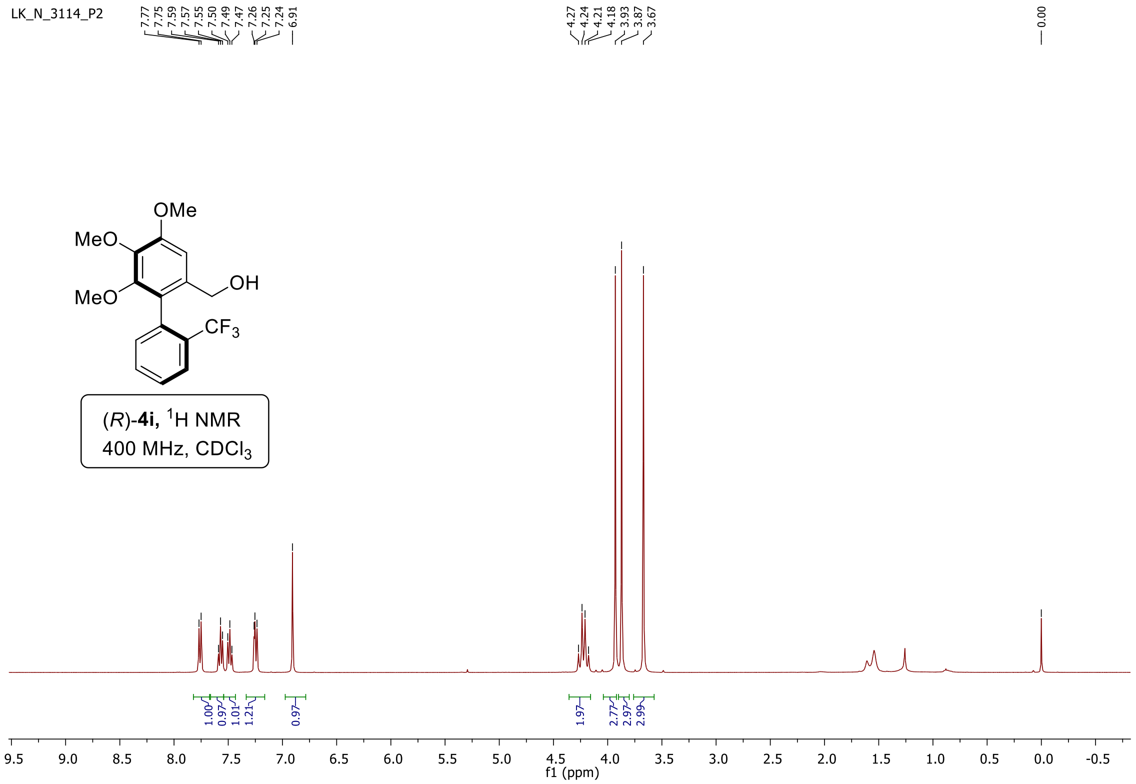

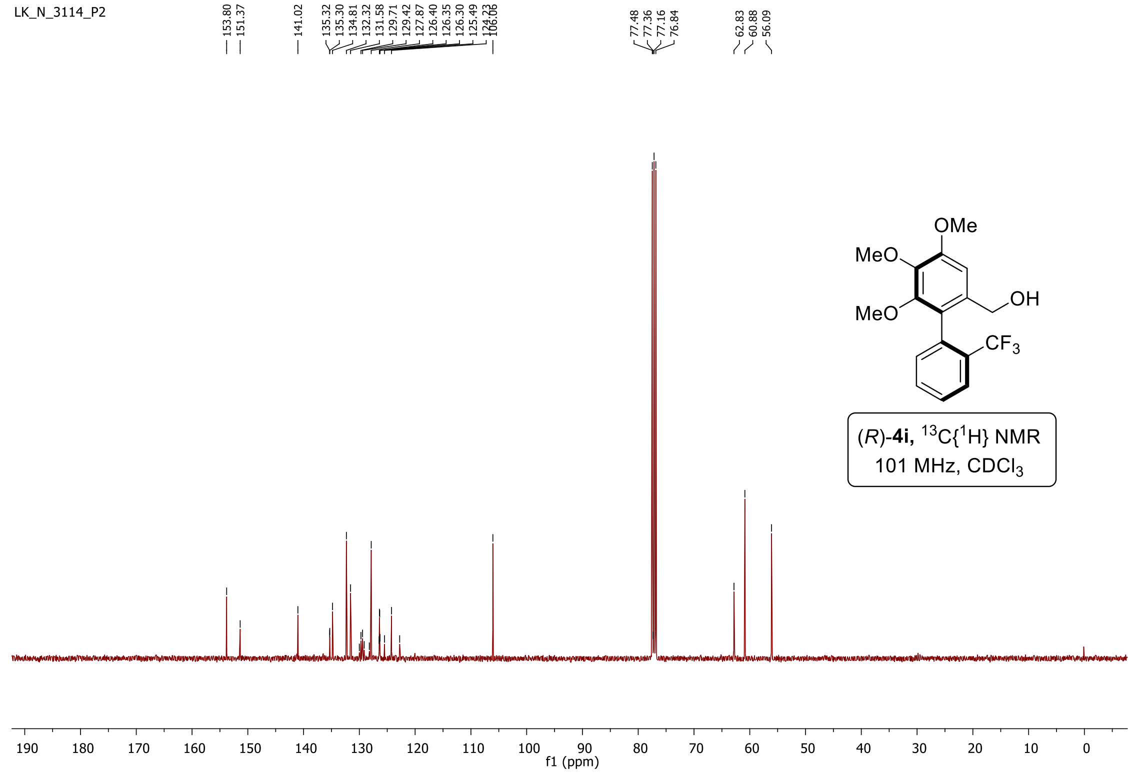

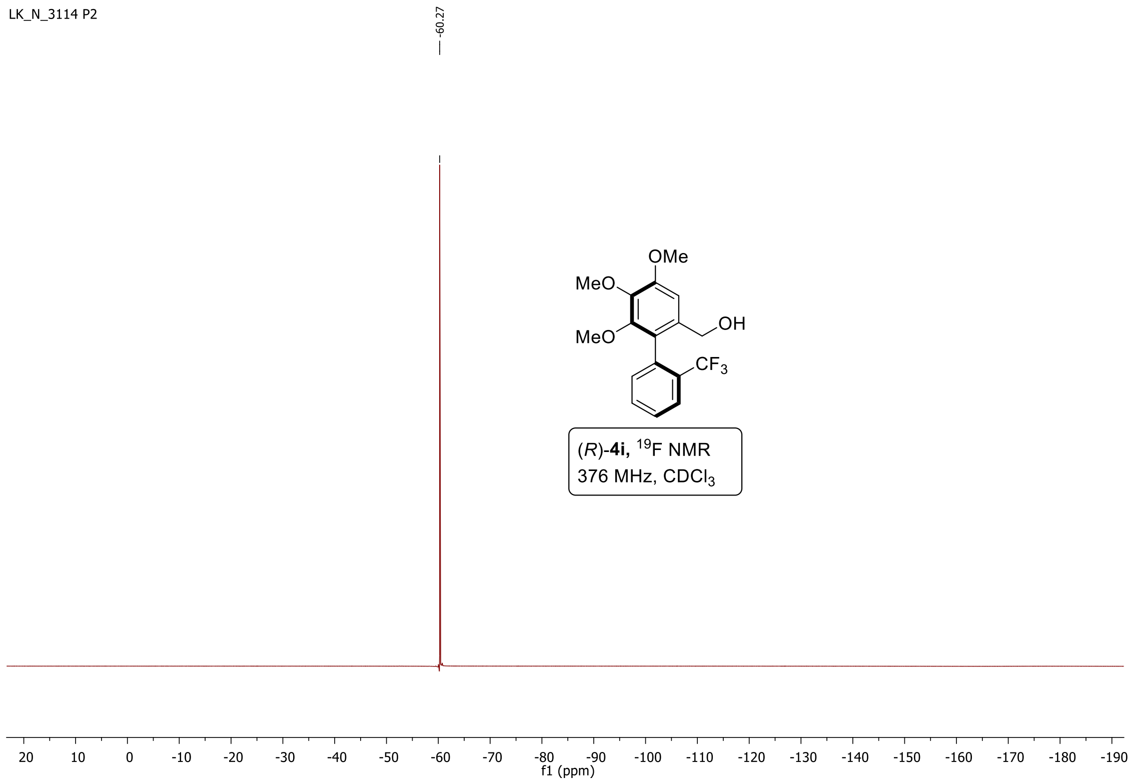

^{1}H and ^{13}C{^{1}H} NMR spectra of (*S*)-**5i**

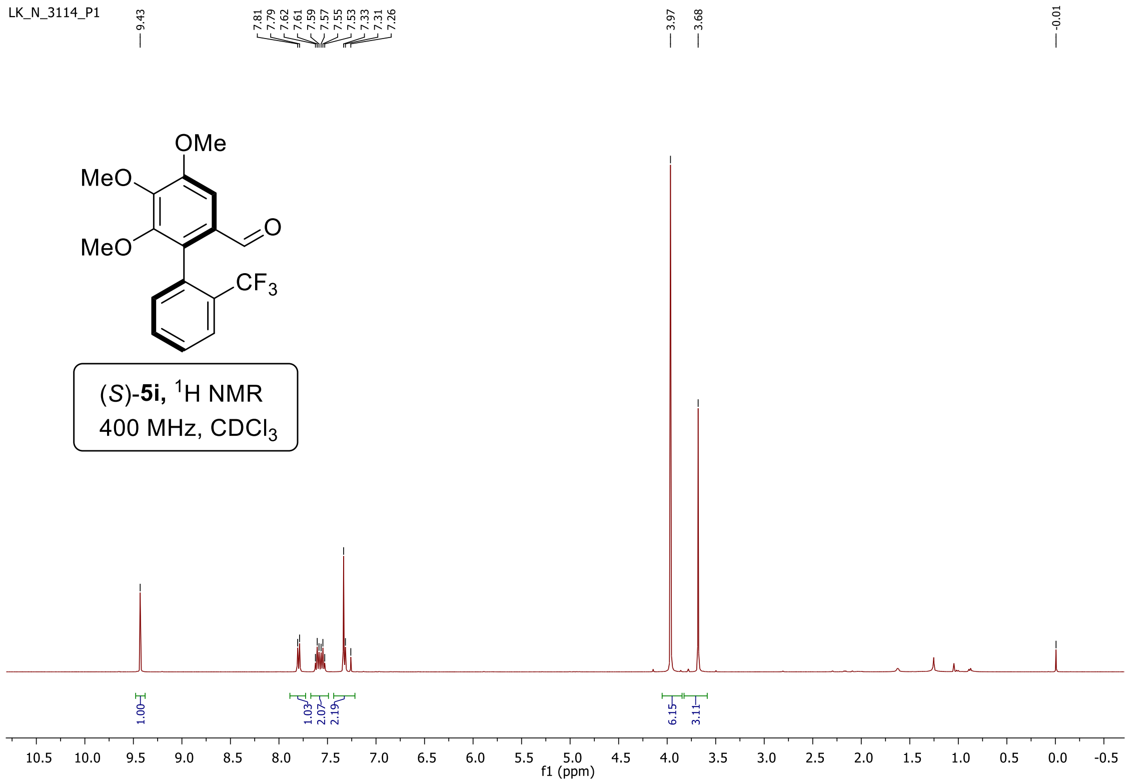

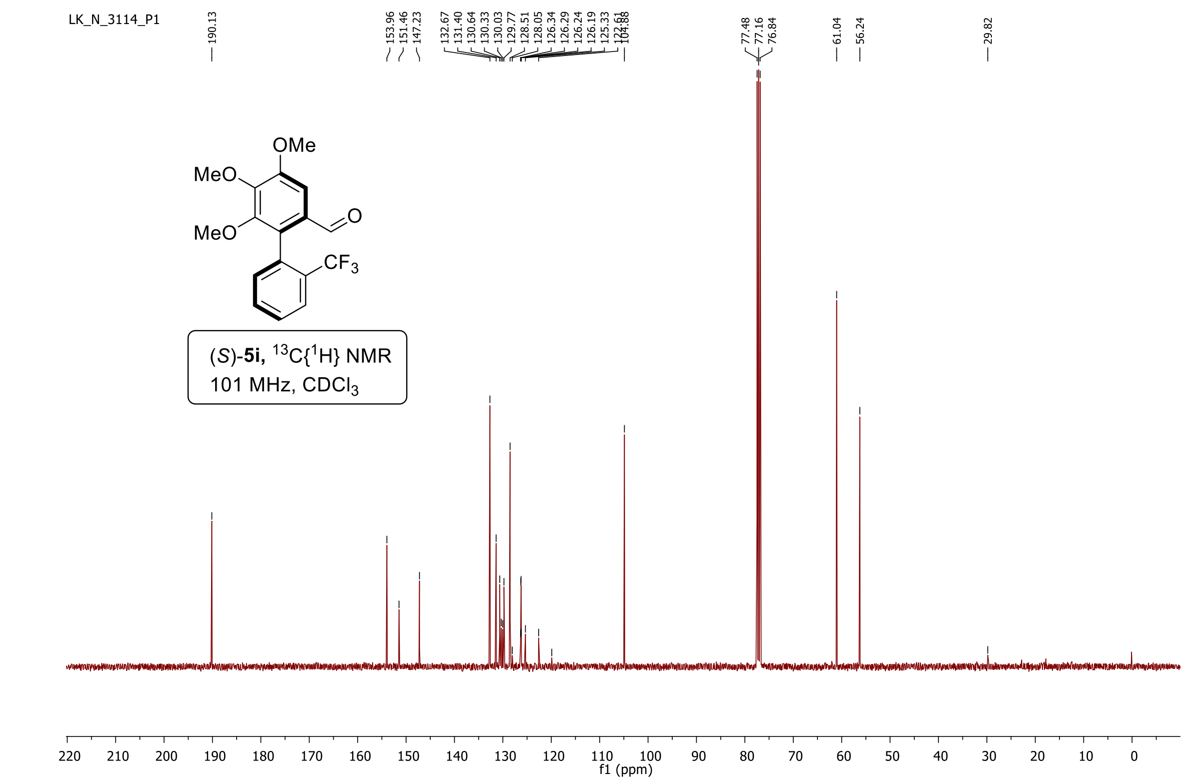

LK_N_3114_P1
190.13
153.96
151.46
147.23
132.67
131.40
130.64
130.33
130.03
129.77
128.51
128.05
126.34
126.29
126.24
126.19
125.33
122.61
104.88
77.48
77.16
76.84
61.04
56.24
29.82
OMe
MeO
MeO
O
CF3
(S)-5i, 13C{1H} NMR
101 MHz, CDCl3
f1 (ppm)

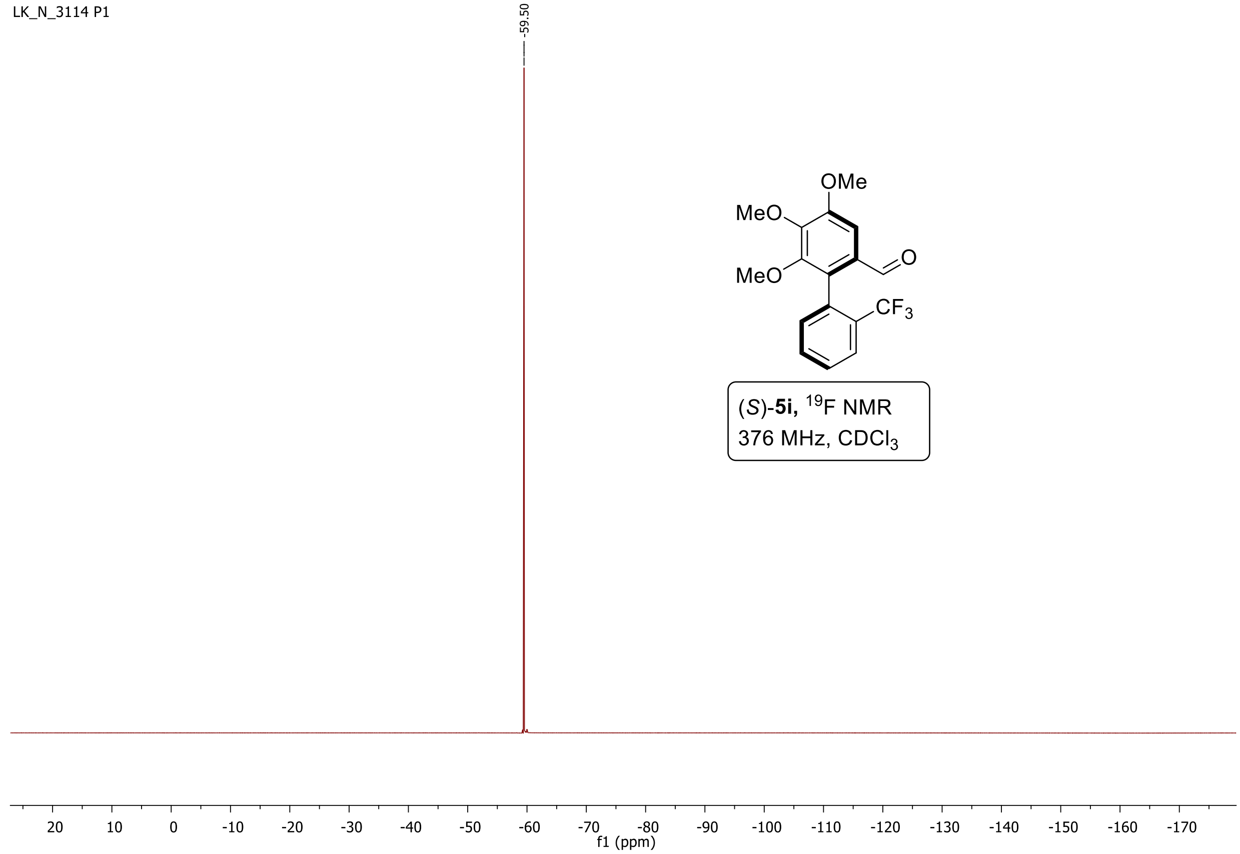

LK_N_3114 P1
-59.50
OMe
MeO
MeO
O
CF3
(S)-5i, 19F NMR
376 MHz, CDCl3
f1 (ppm)

^1H and $^{13}C\{^1H\}$ NMR spectra of (*R*)-**4j**

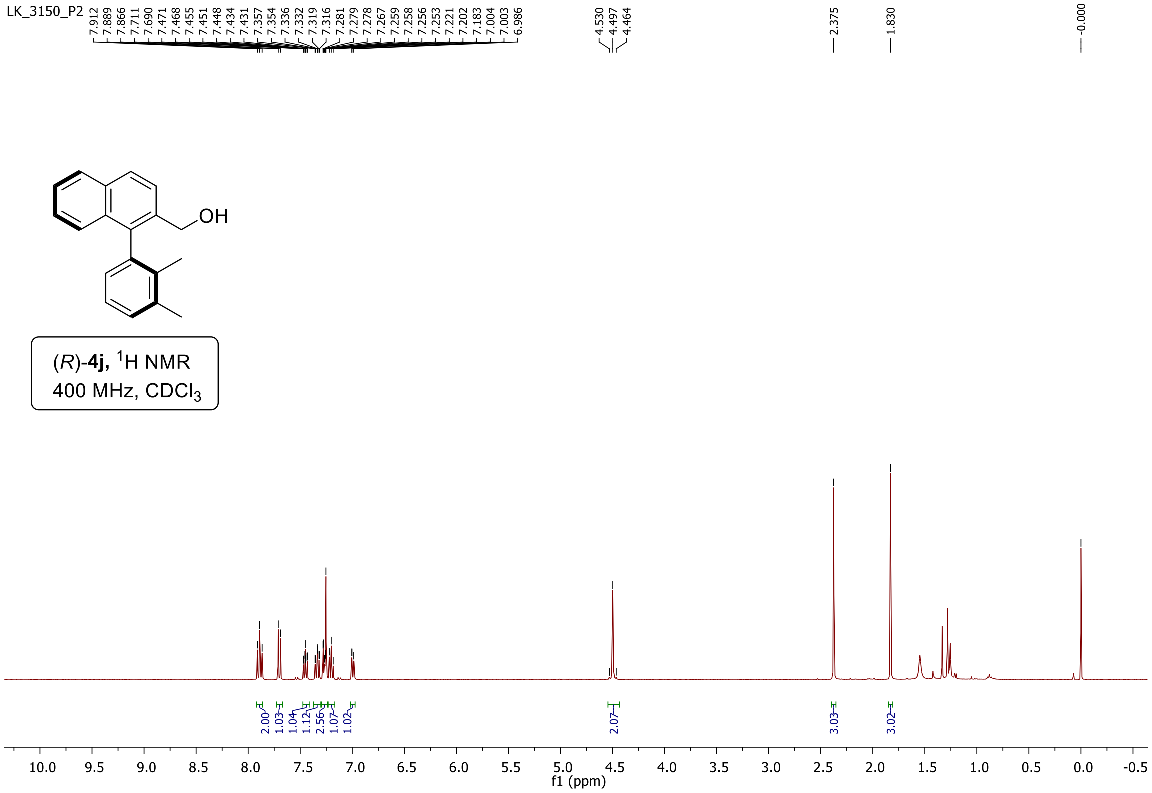

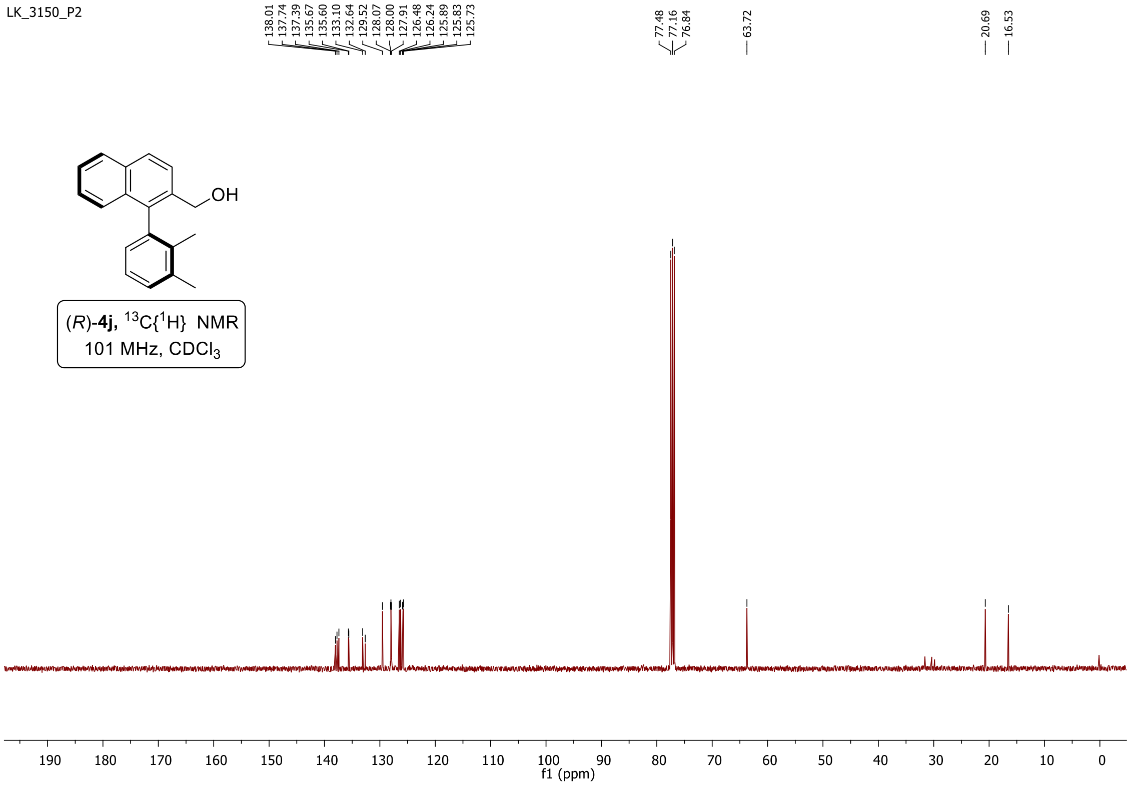

^{1}H and $^{13}C\{^{1}H\}$ NMR spectra of (*S*)-**5j**

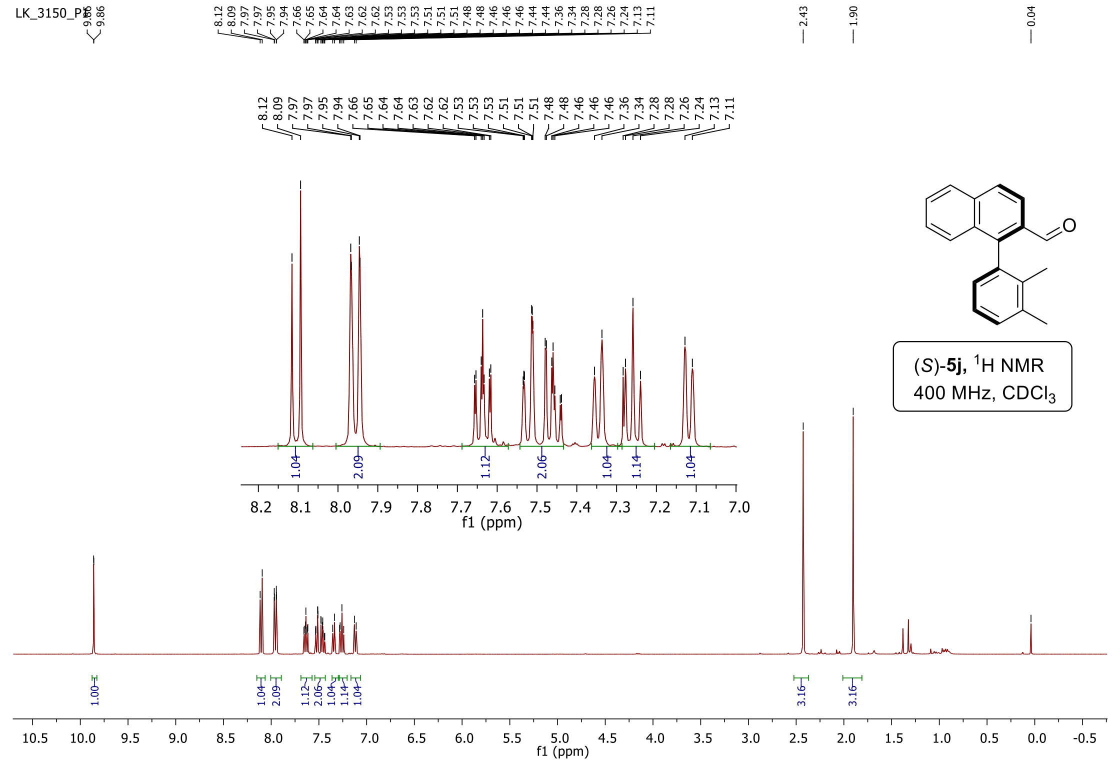

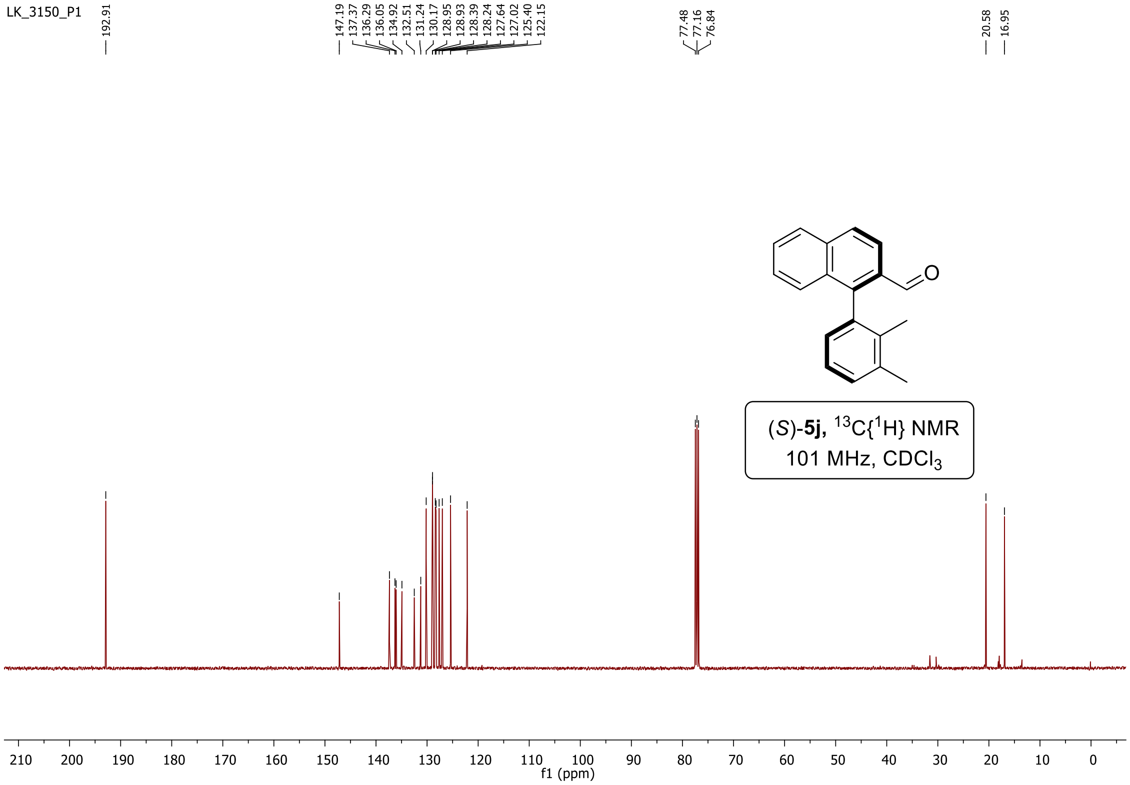

^{1}H and $^{13}C\{^{1}H\}$ NMR spectra of (*R*)-**4k**

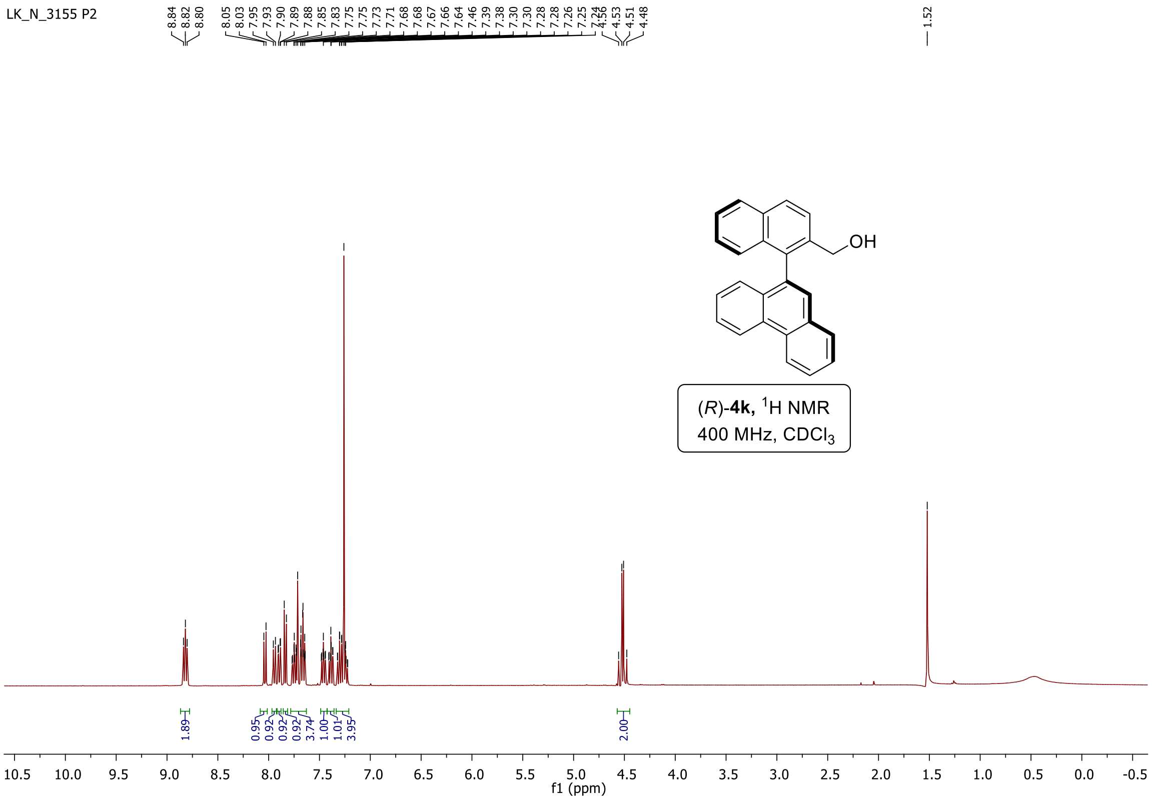

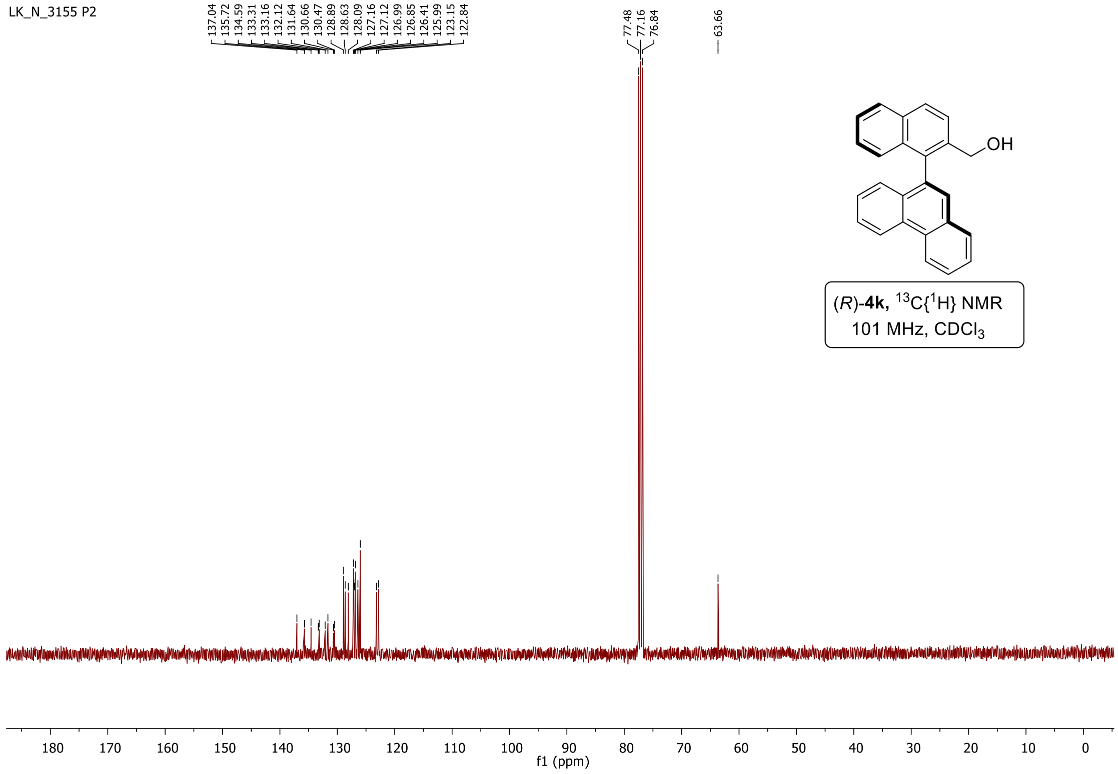

^1H and $^{13}C\{^1H\}$ NMR spectra of (*S*)-**5k**

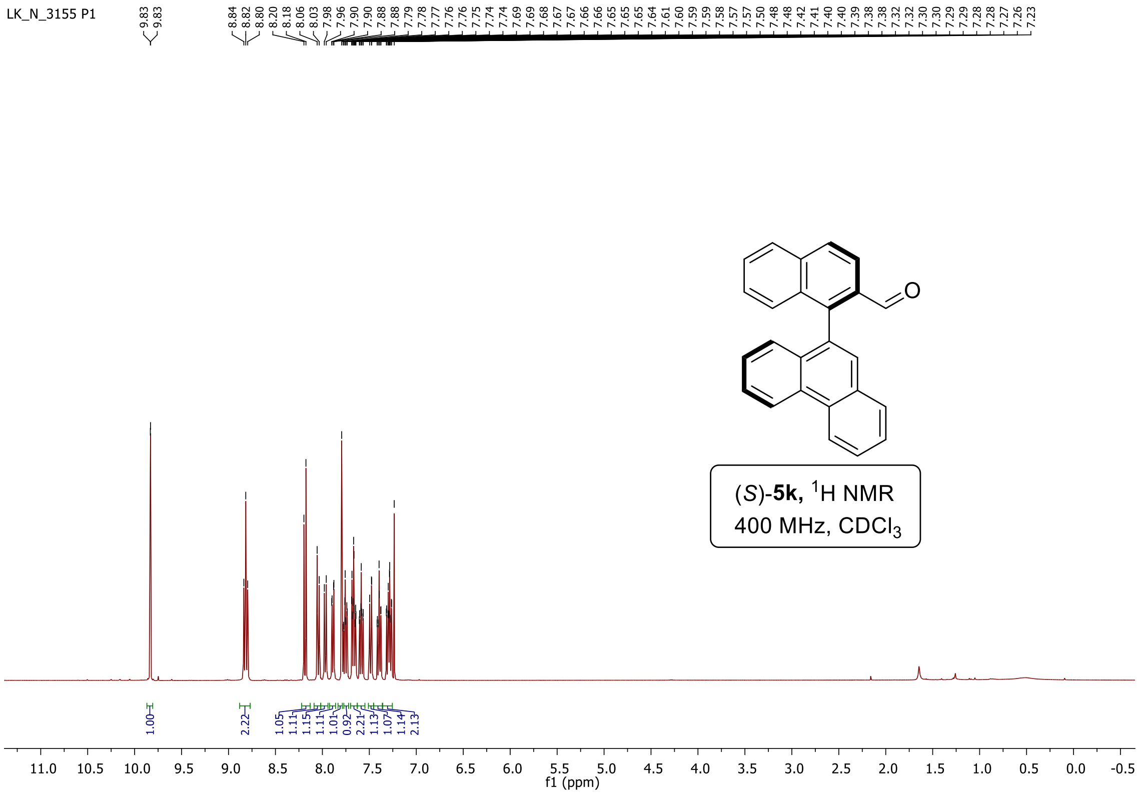

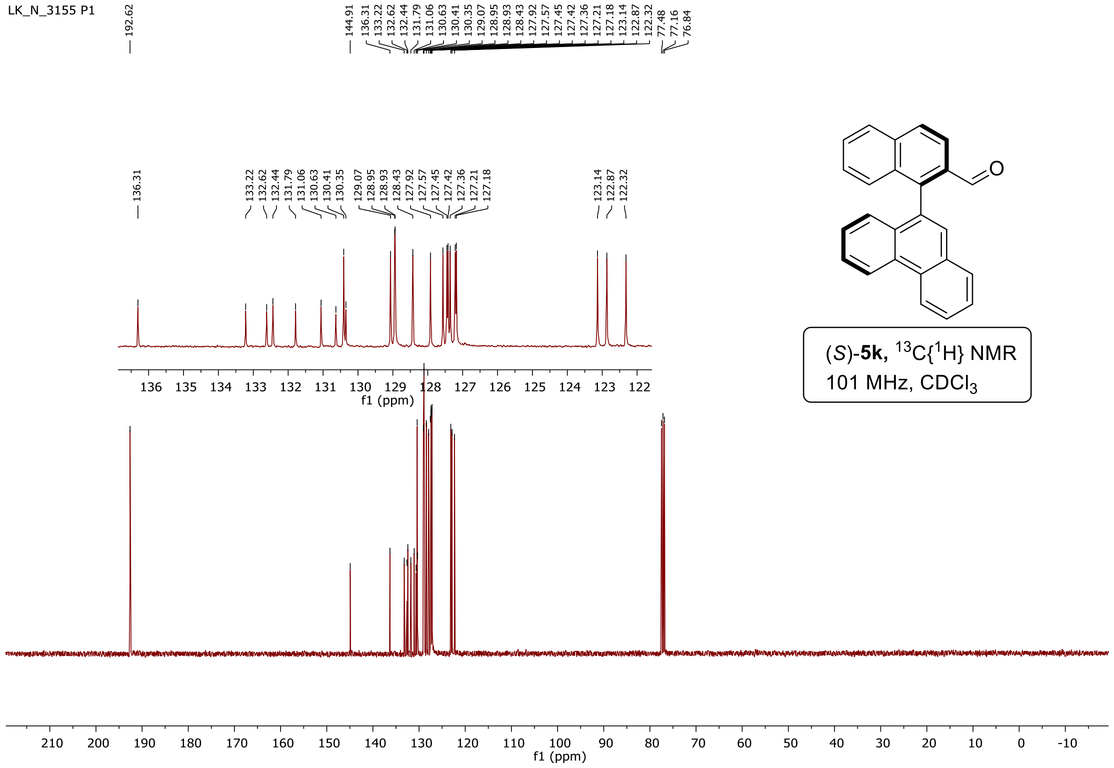

^{1}H and $^{13}C\{^{1}H\}$ NMR spectra of (*R*)-**4l**

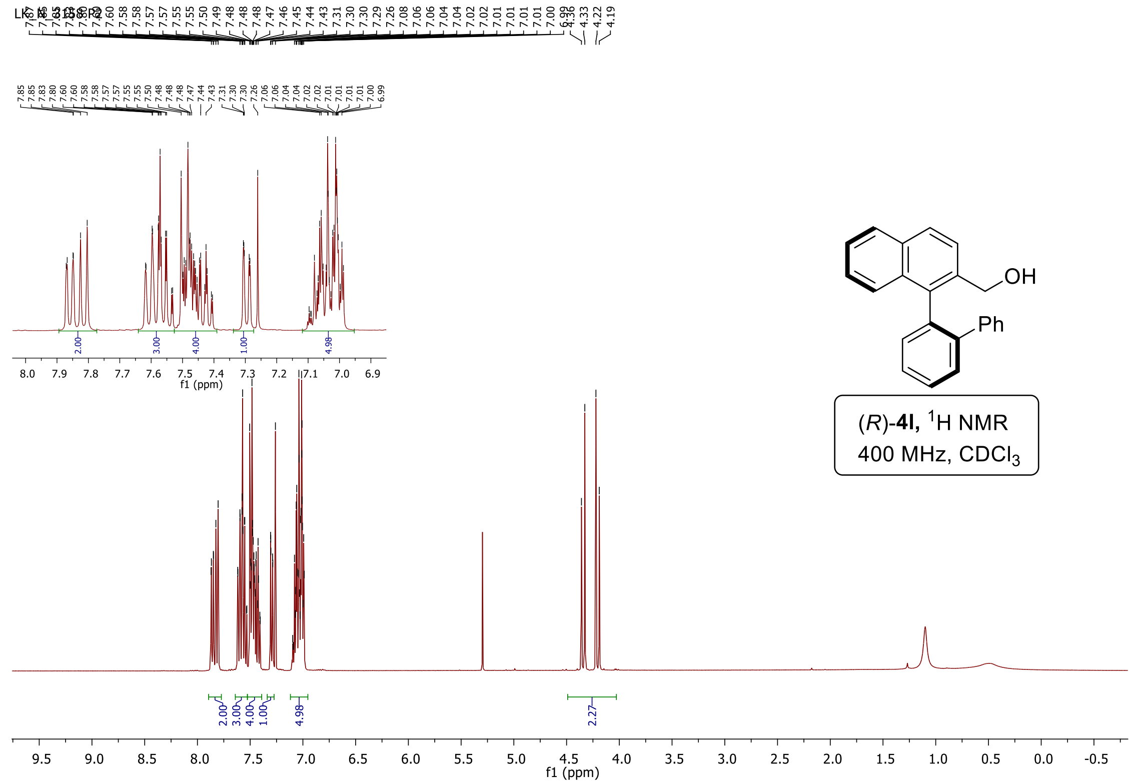

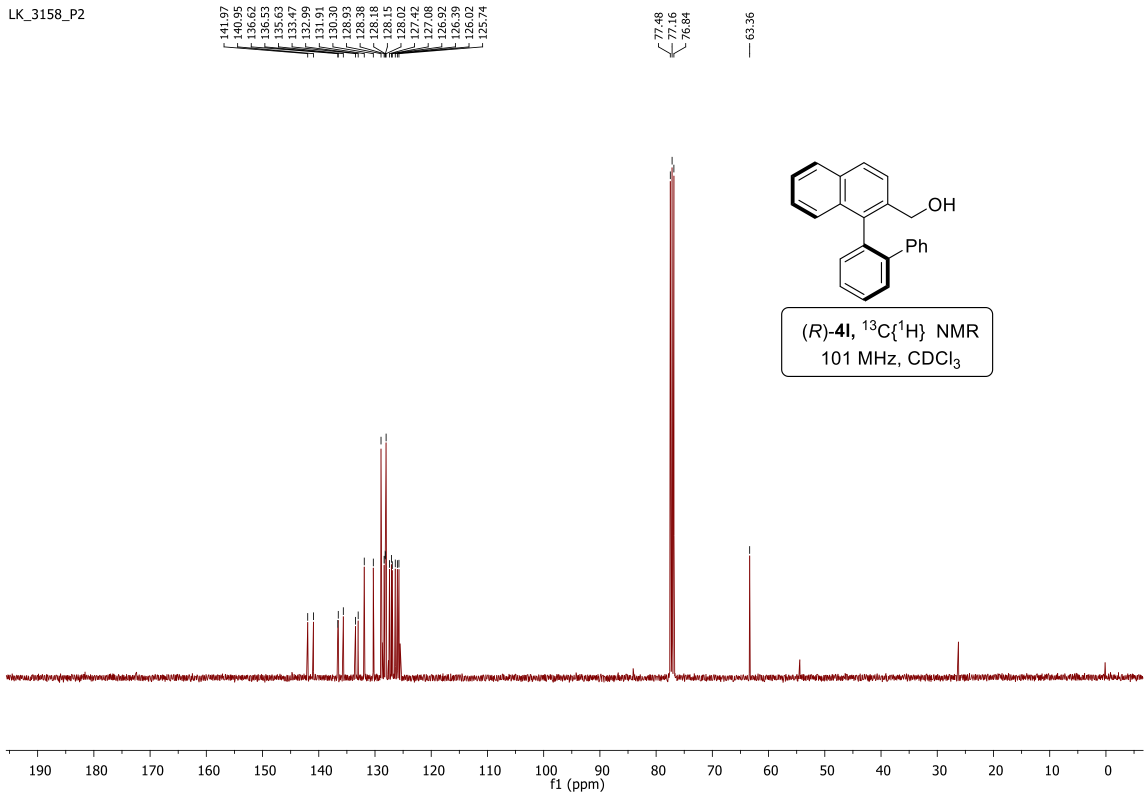

^{1}H and $^{13}C\{^{1}H\}$ NMR spectra of (*S*)-**5l**

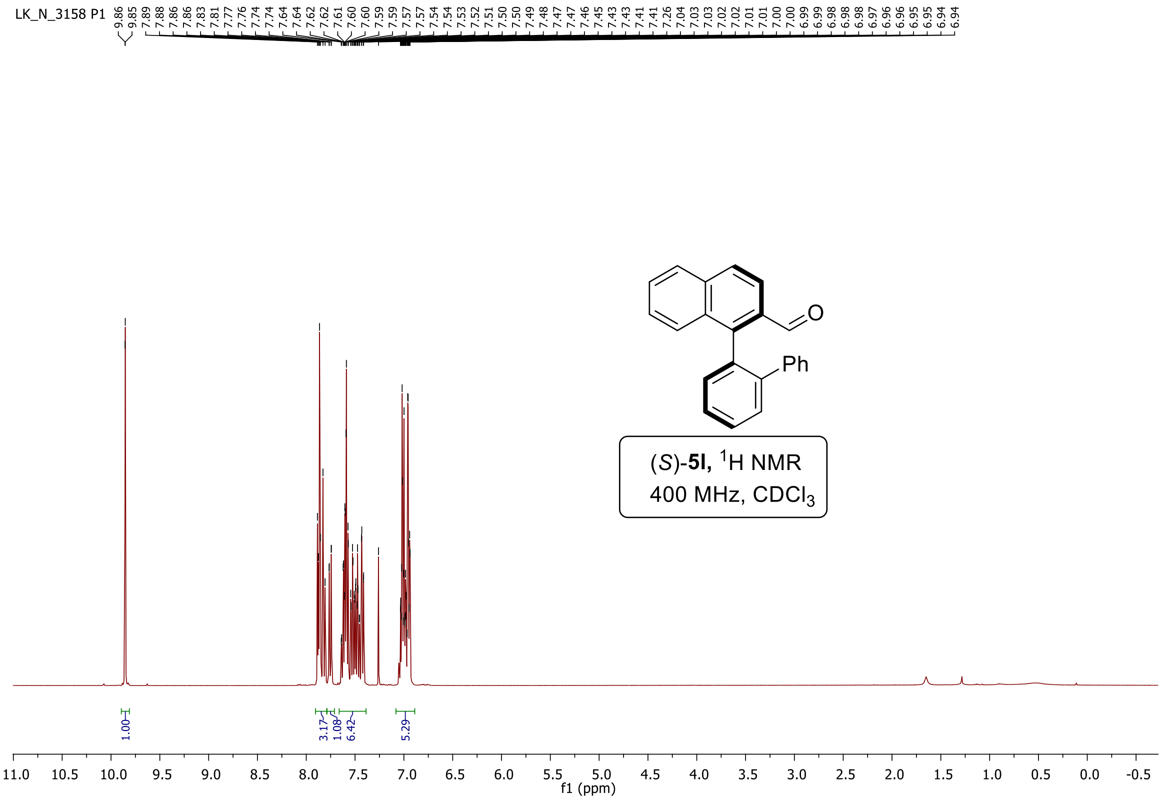

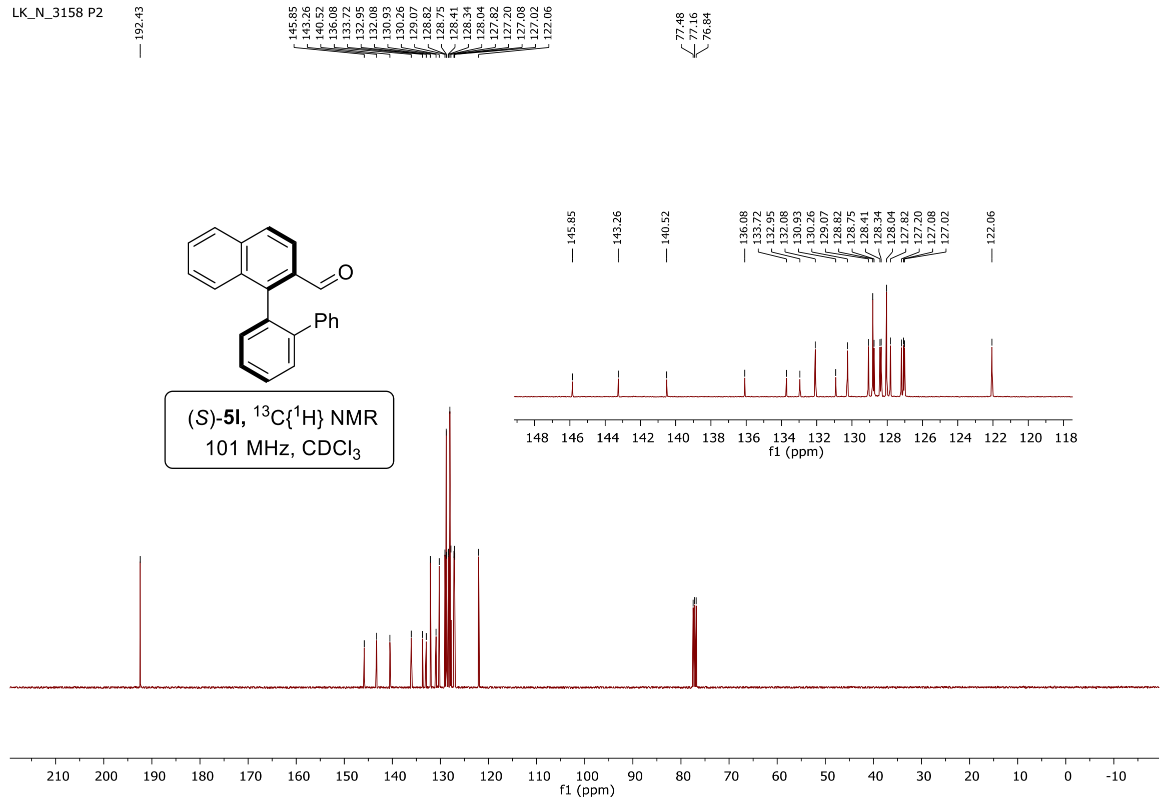

^{1}H and $^{13}C\{^{1}H\}$ NMR spectra of (*R*)-**4m**

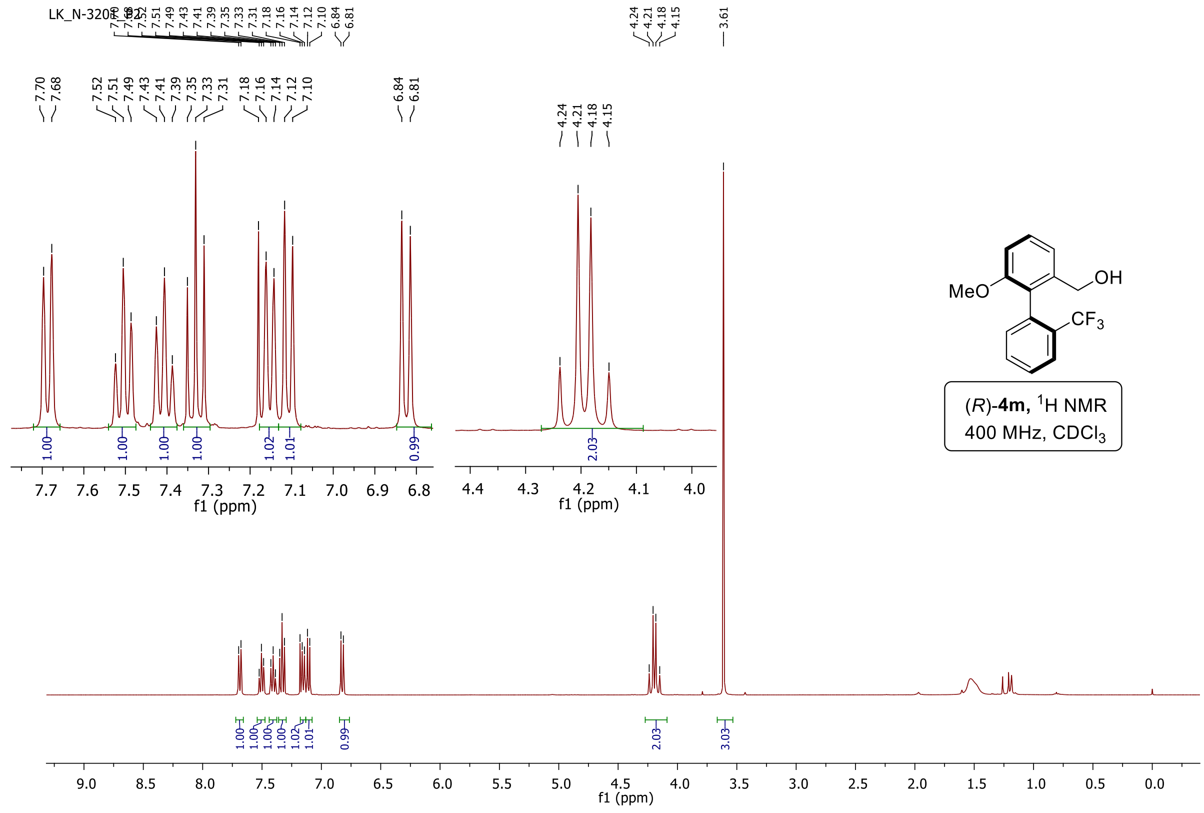

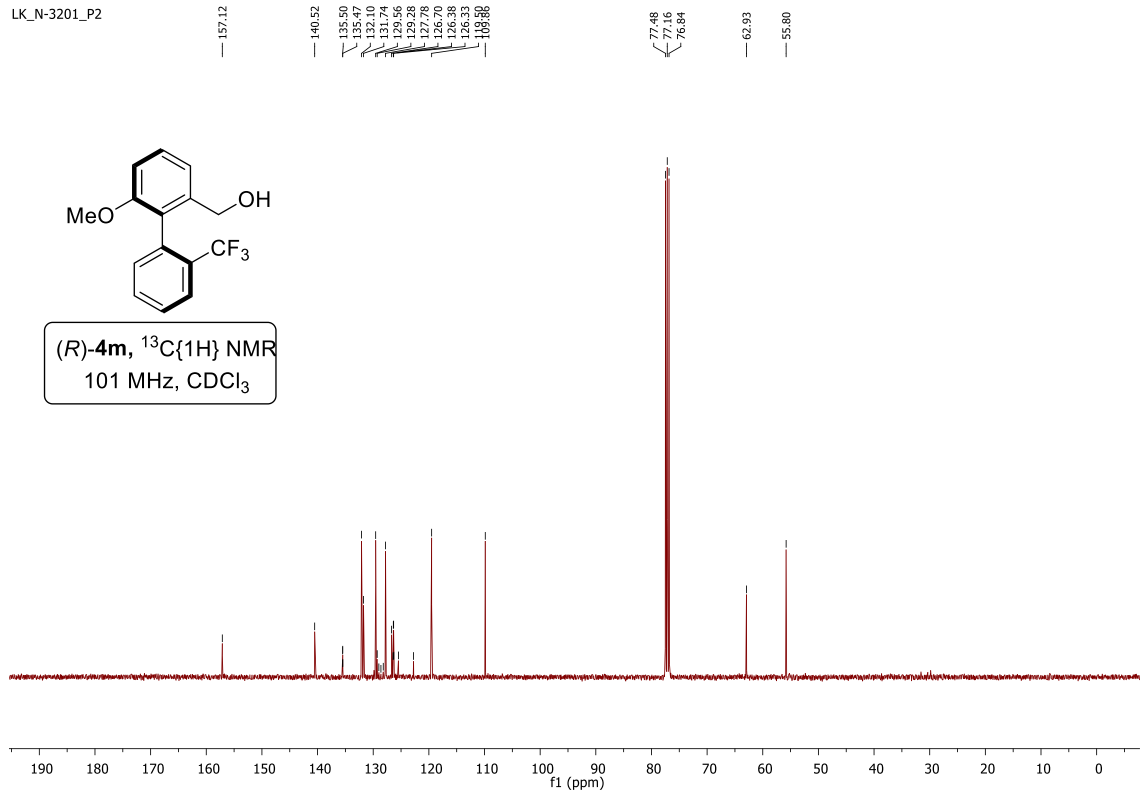

^{1}H and $^{13}C\{^{1}H\}$ NMR spectra of (*S*)-**5m**

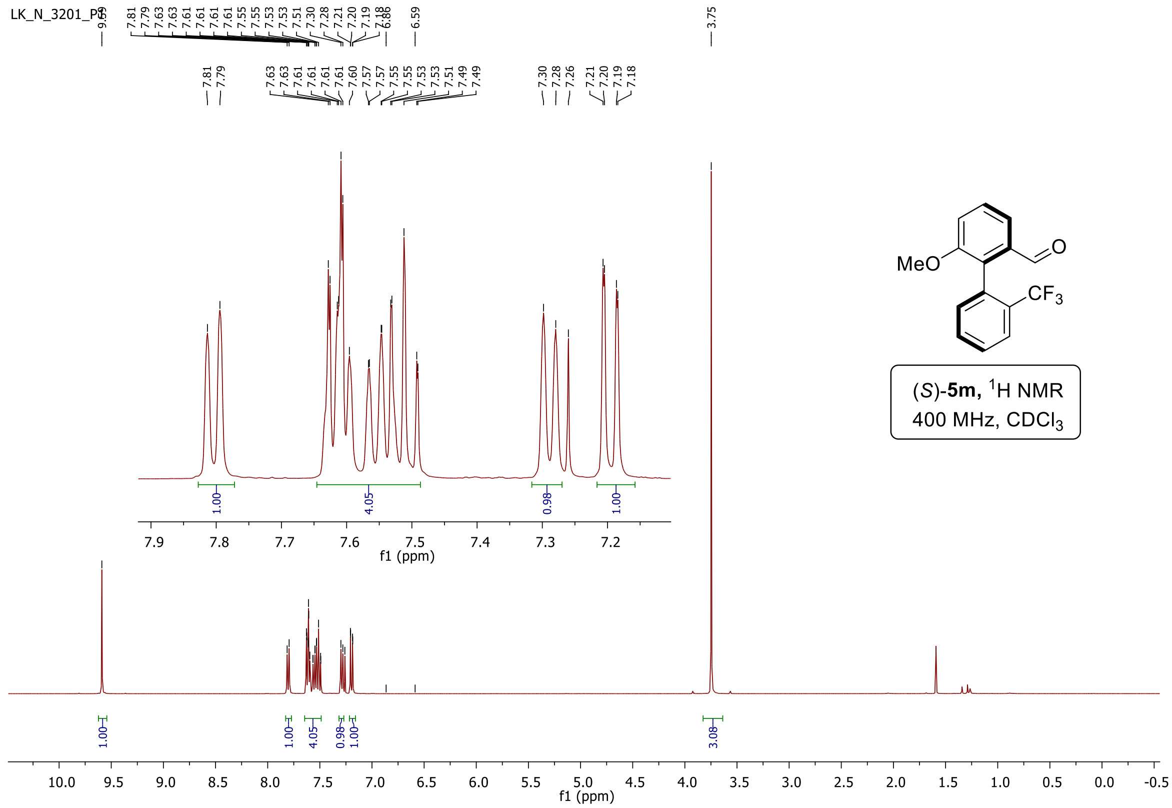

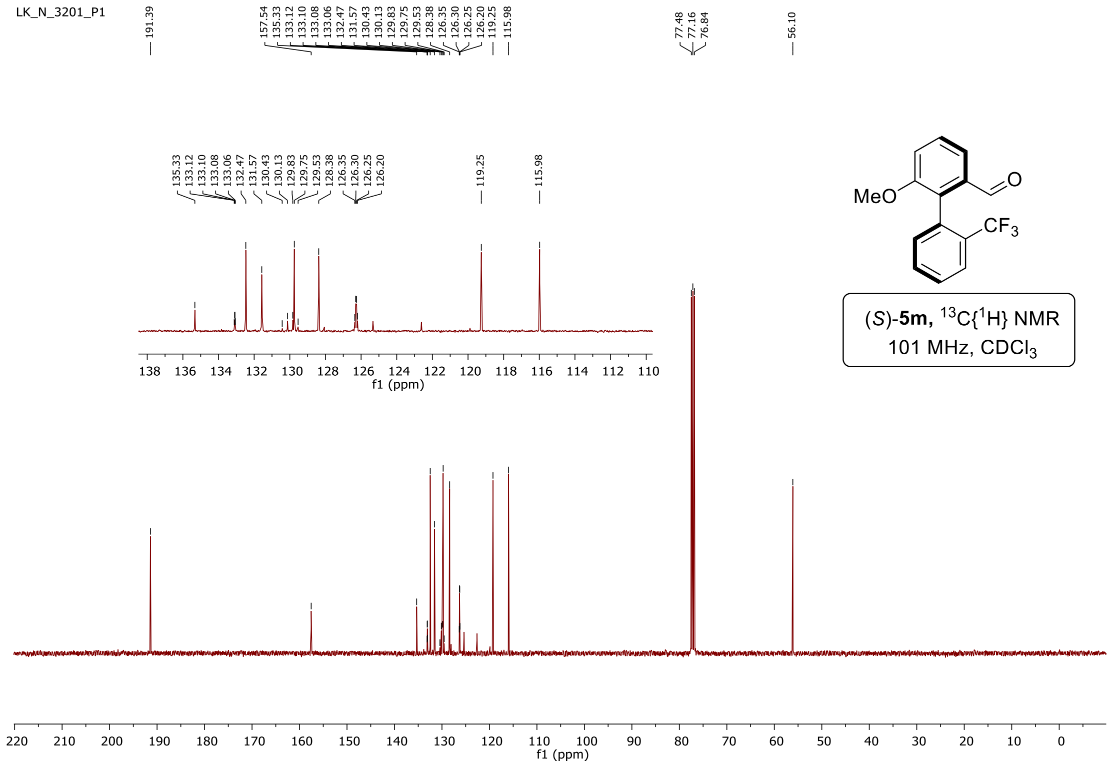

^{1}H and $^{13}C\{^{1}H\}$ NMR spectra of (*R*)-**4n**

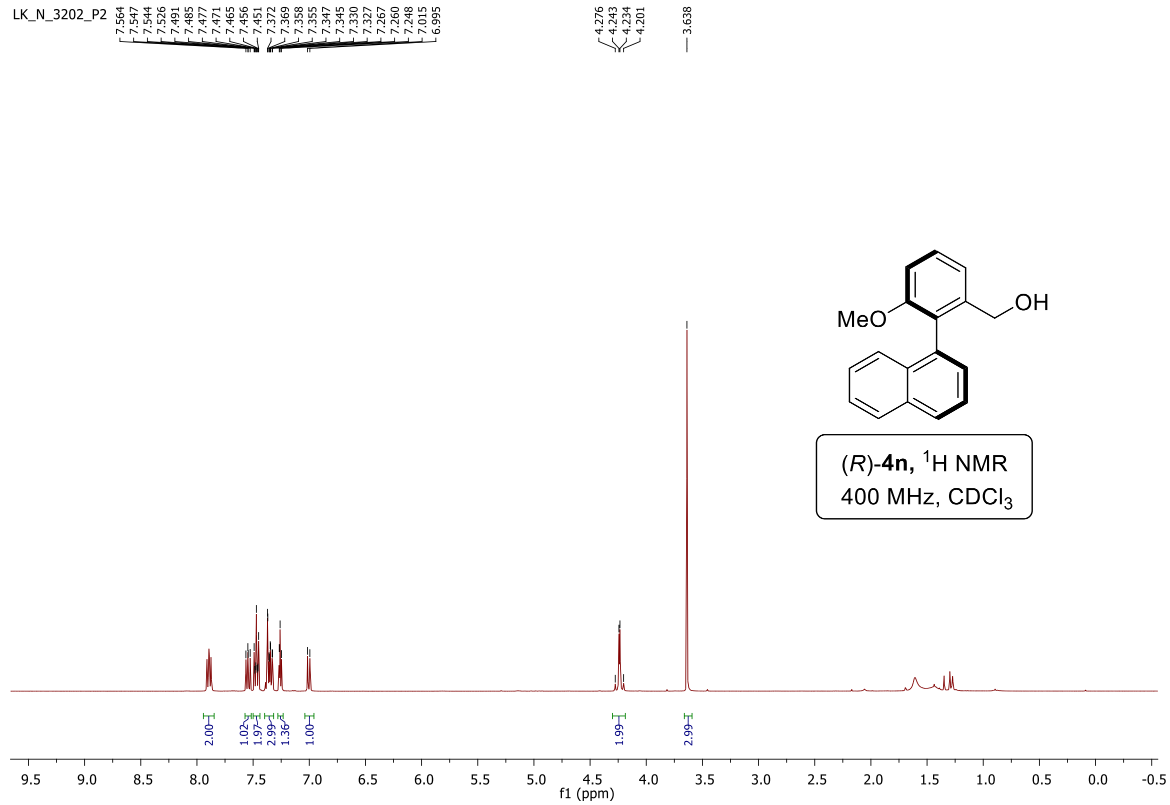

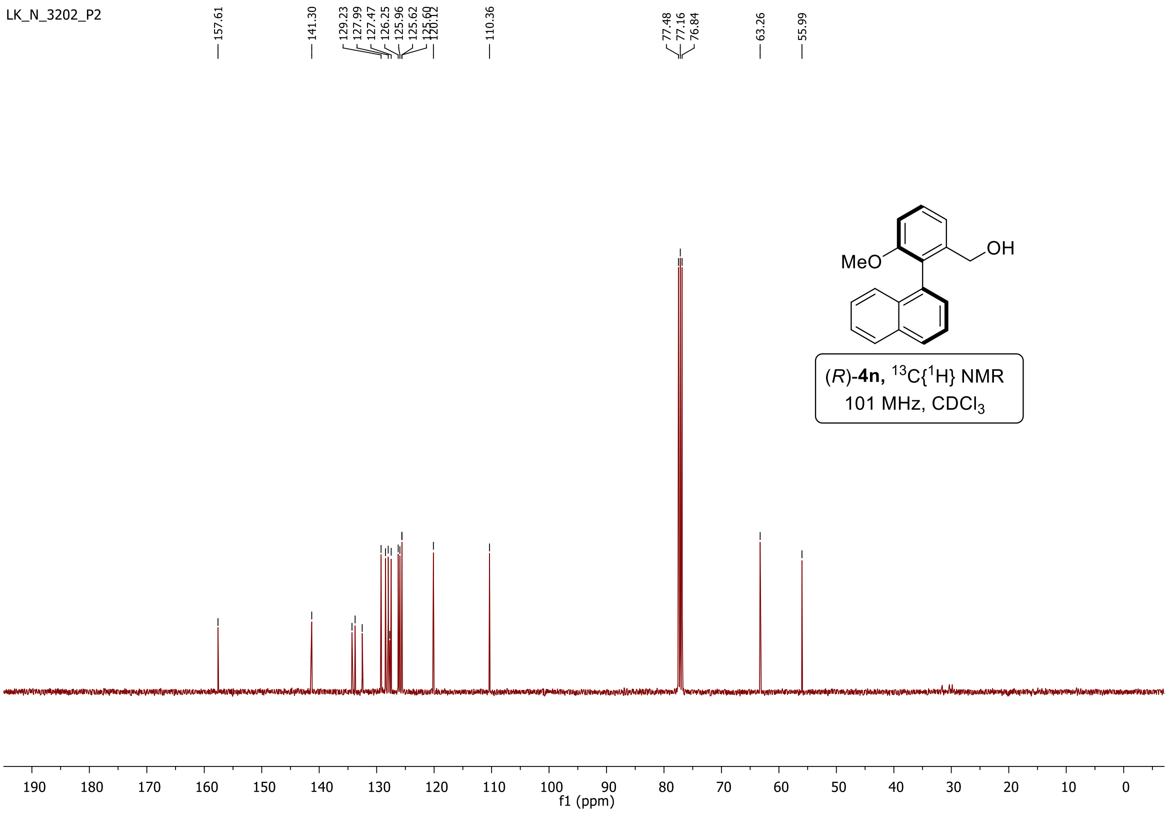

^{1}H and ^{13}C{^{1}H} NMR spectra of (*S*)-**5n**

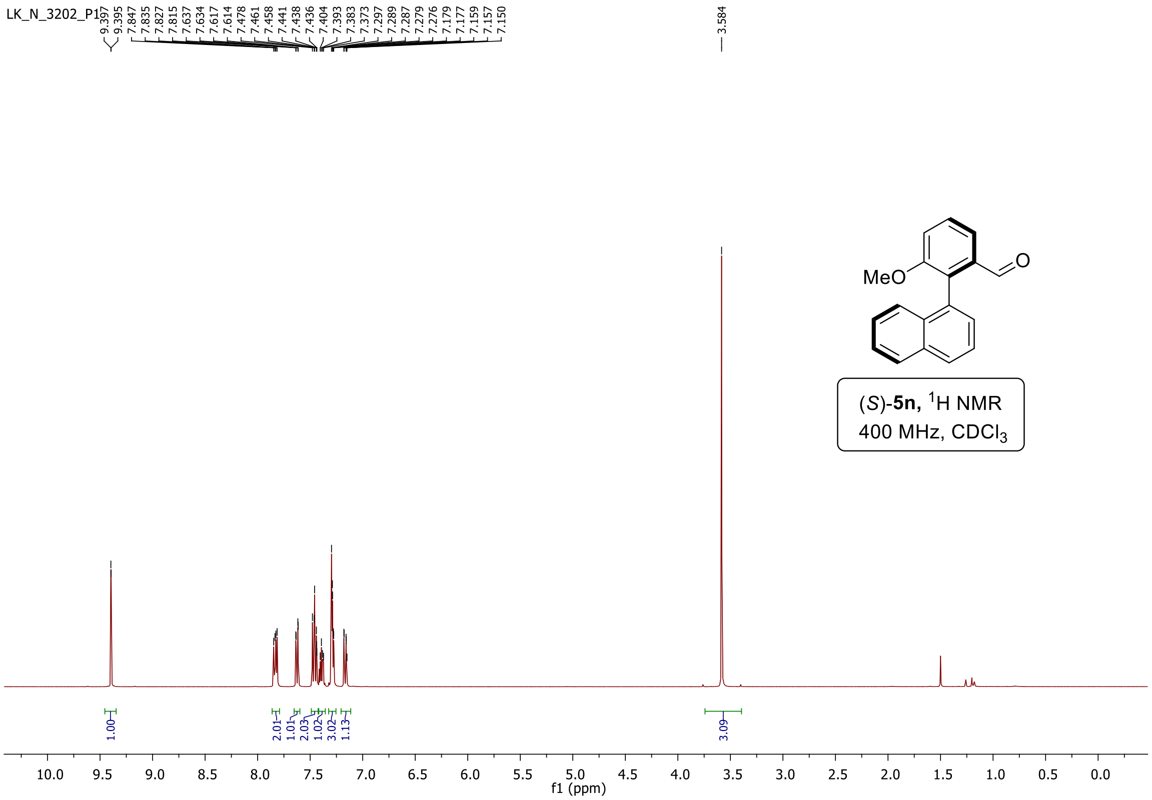

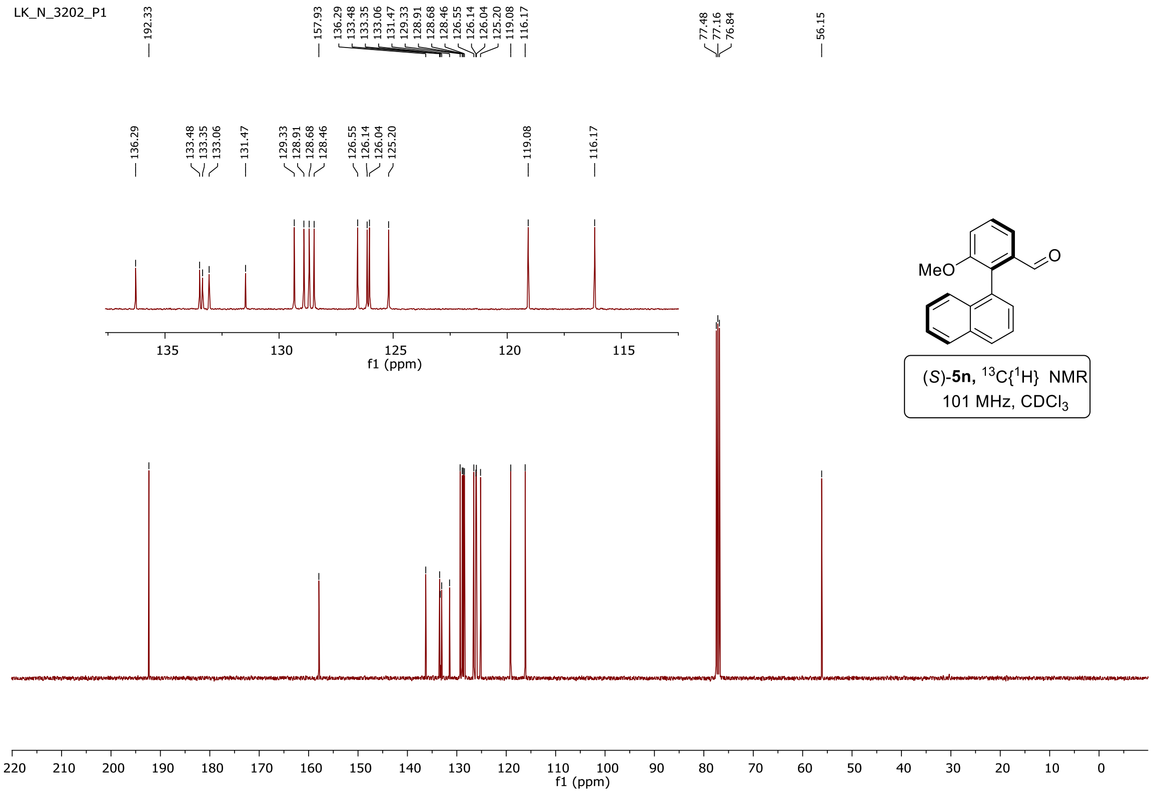

^1H and $^{13}C\{^1H\}$ NMR spectra of (*R*)-**4o**

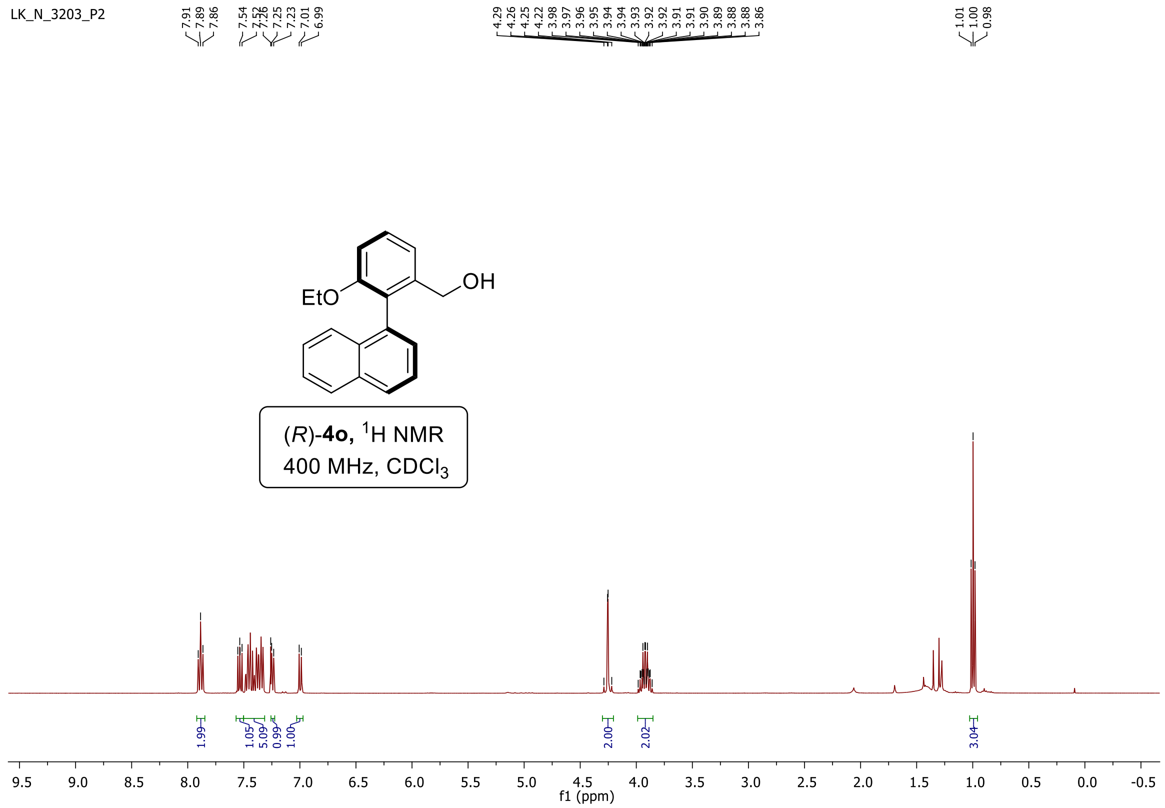

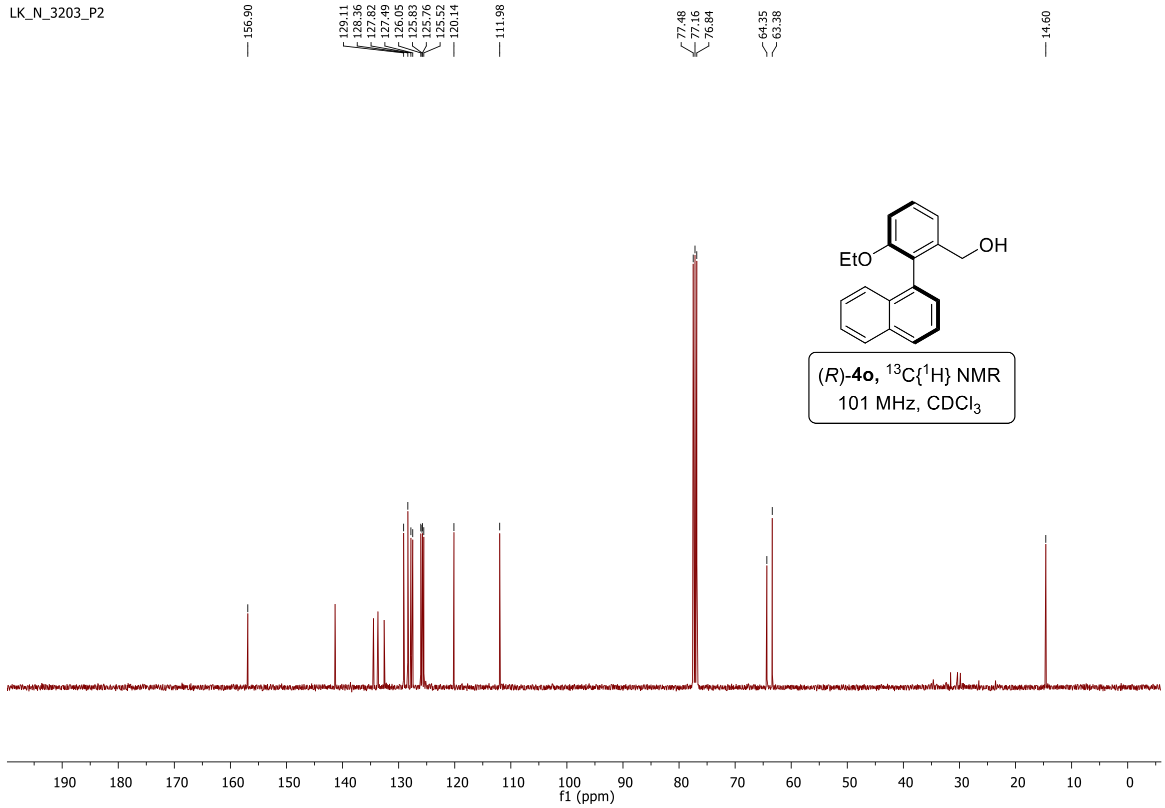

^{1}H and $^{13}C\{^{1}H\}$ NMR spectra of (*S*)-**5o**

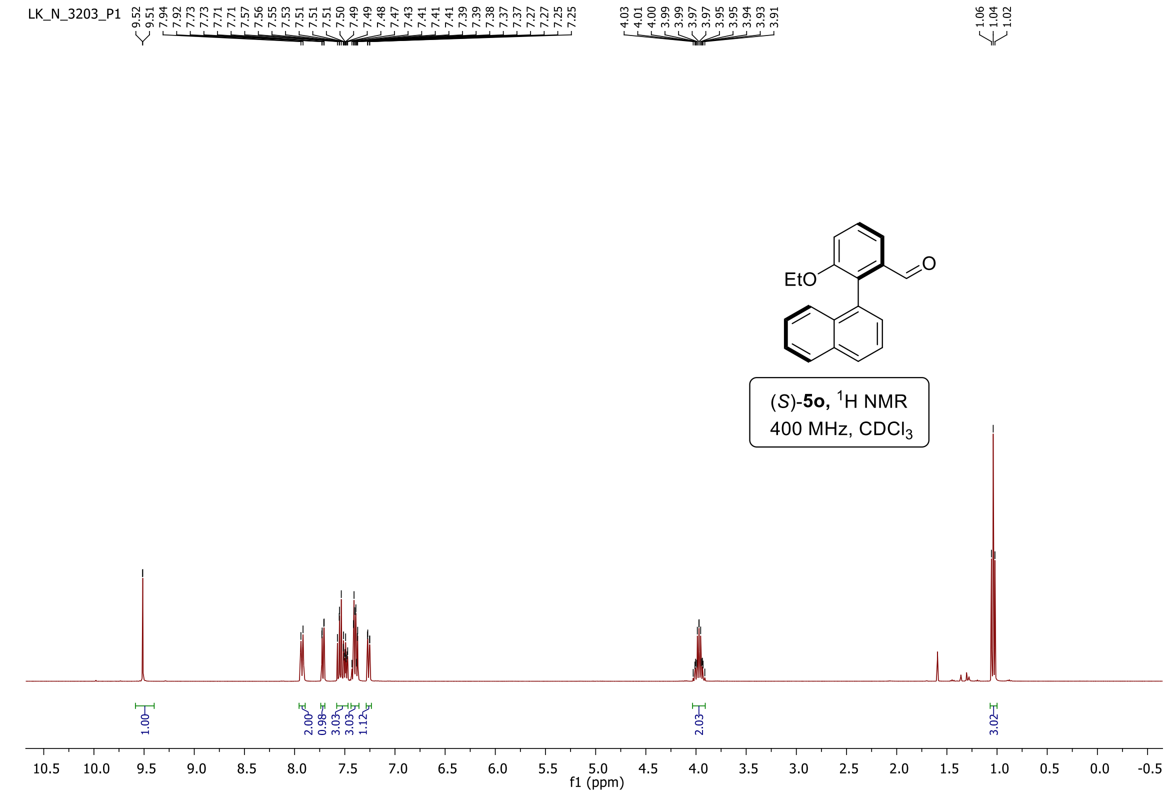

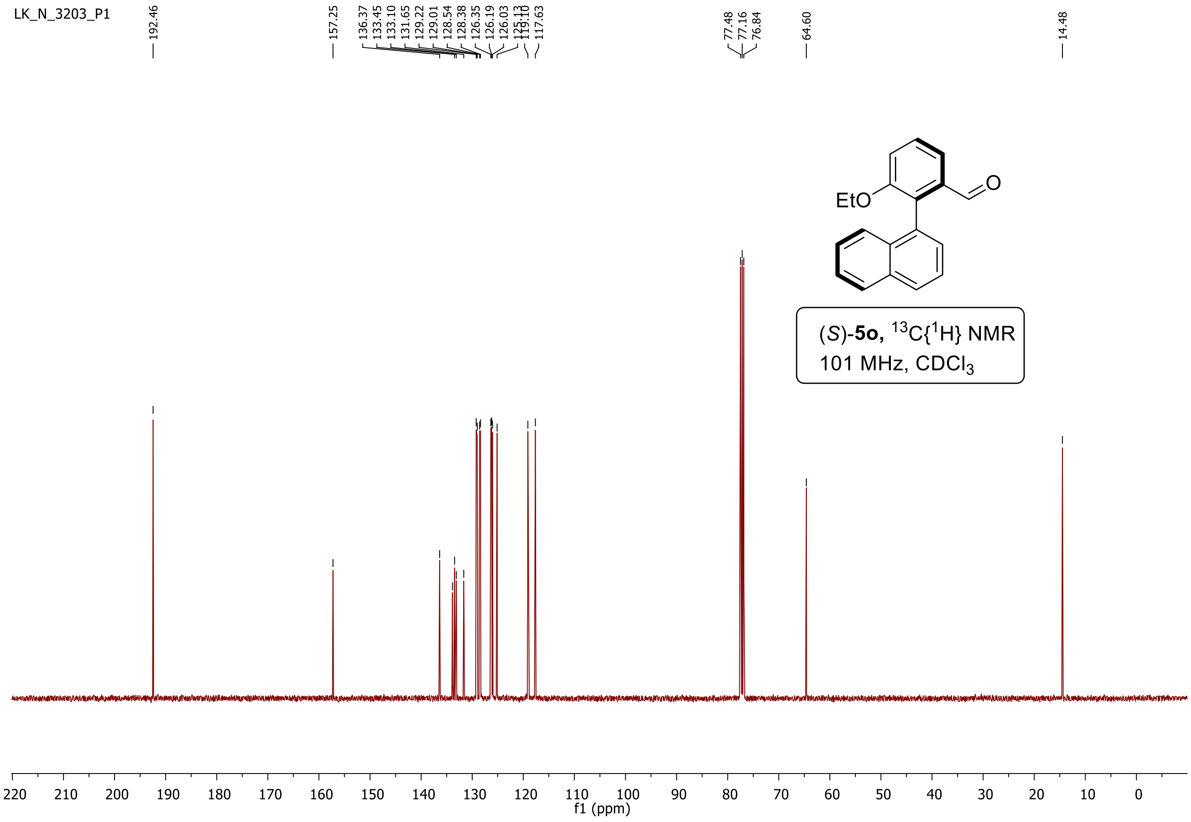

^{1}H and ^{13}C{^{1}H} NMR spectra of (*R*)-**4p**

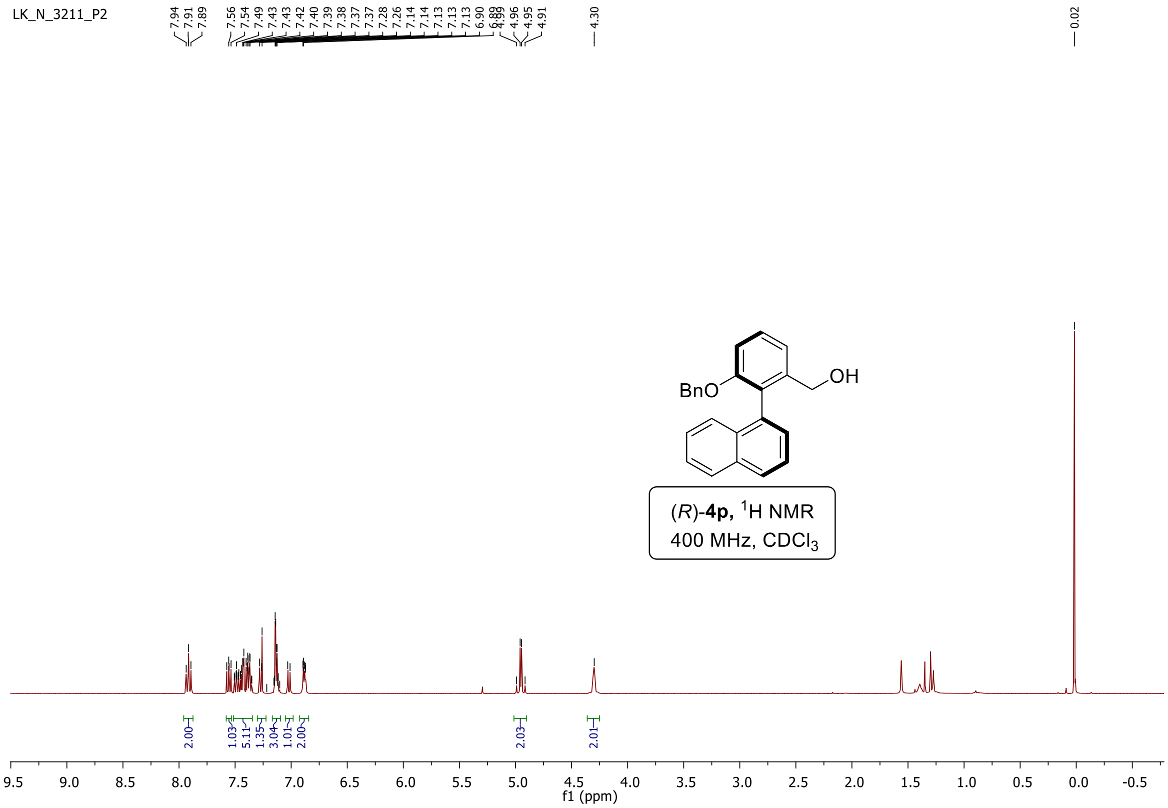

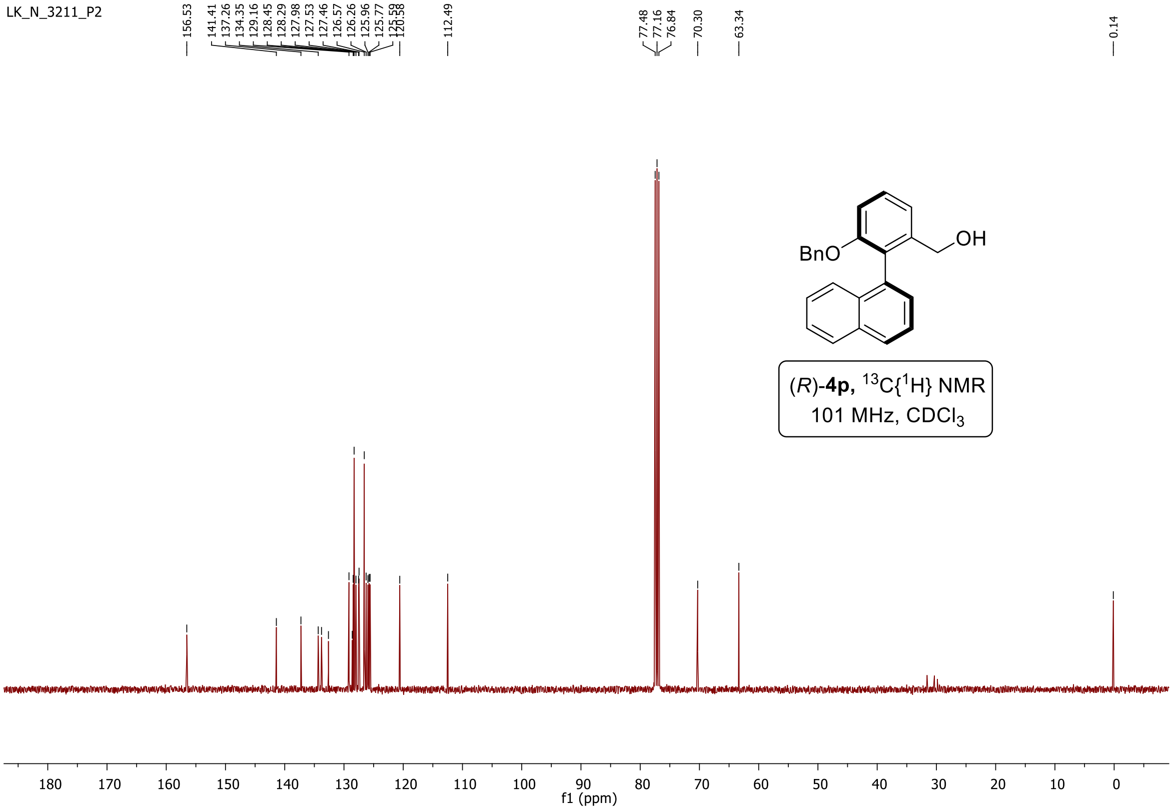

^{1}H and $^{13}C\{^{1}H\}$ NMR spectra of (*S*)-**5p**

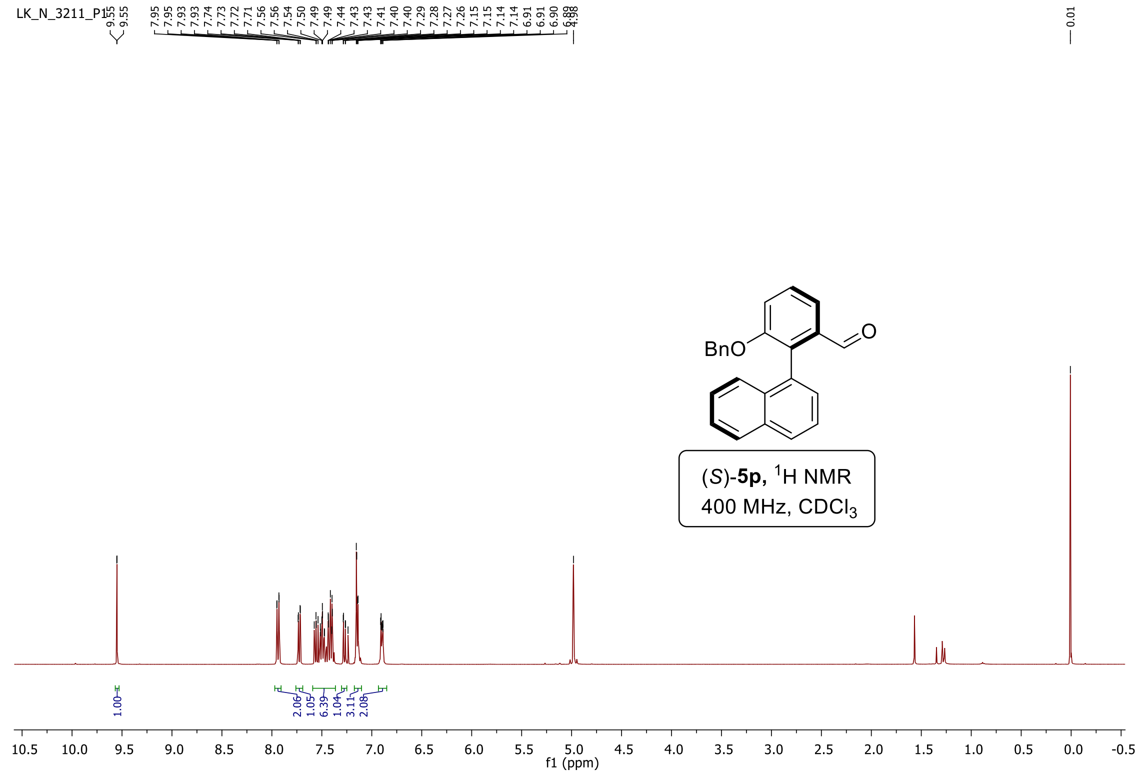

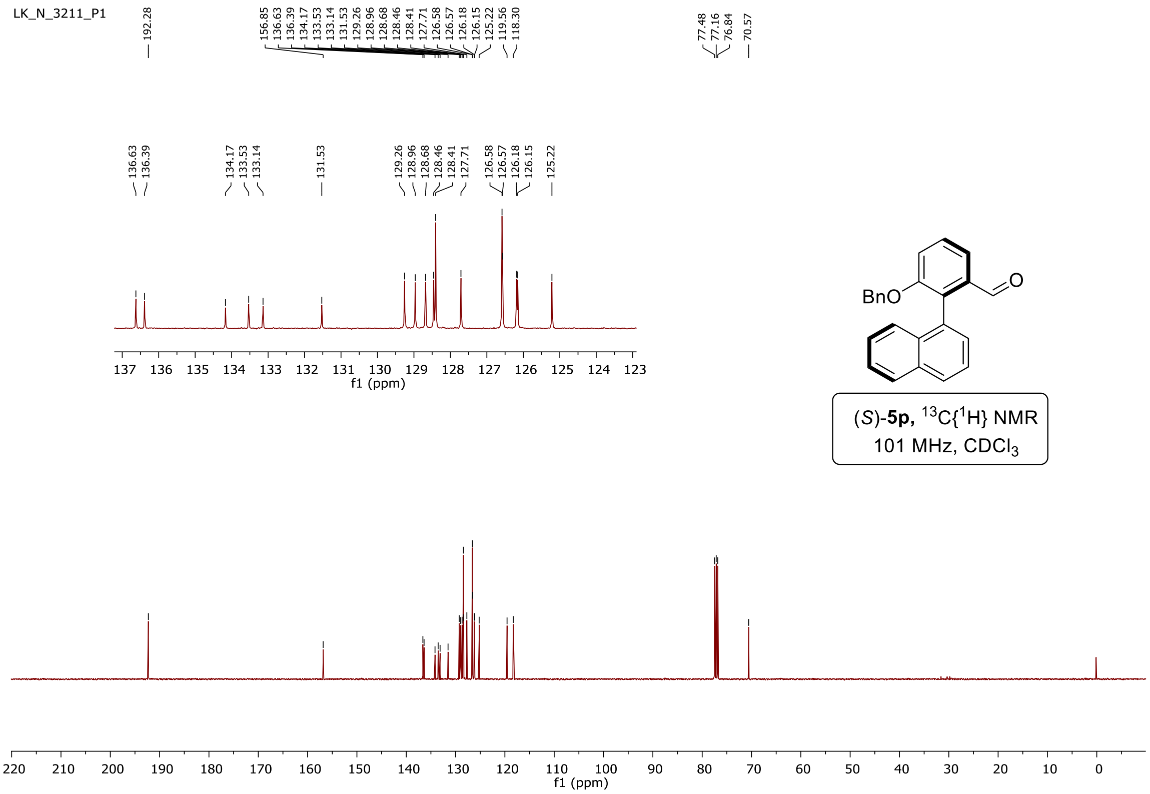

^{1}H and $^{13}C\{^{1}H\}$ NMR spectra of (*R*)-**4q**

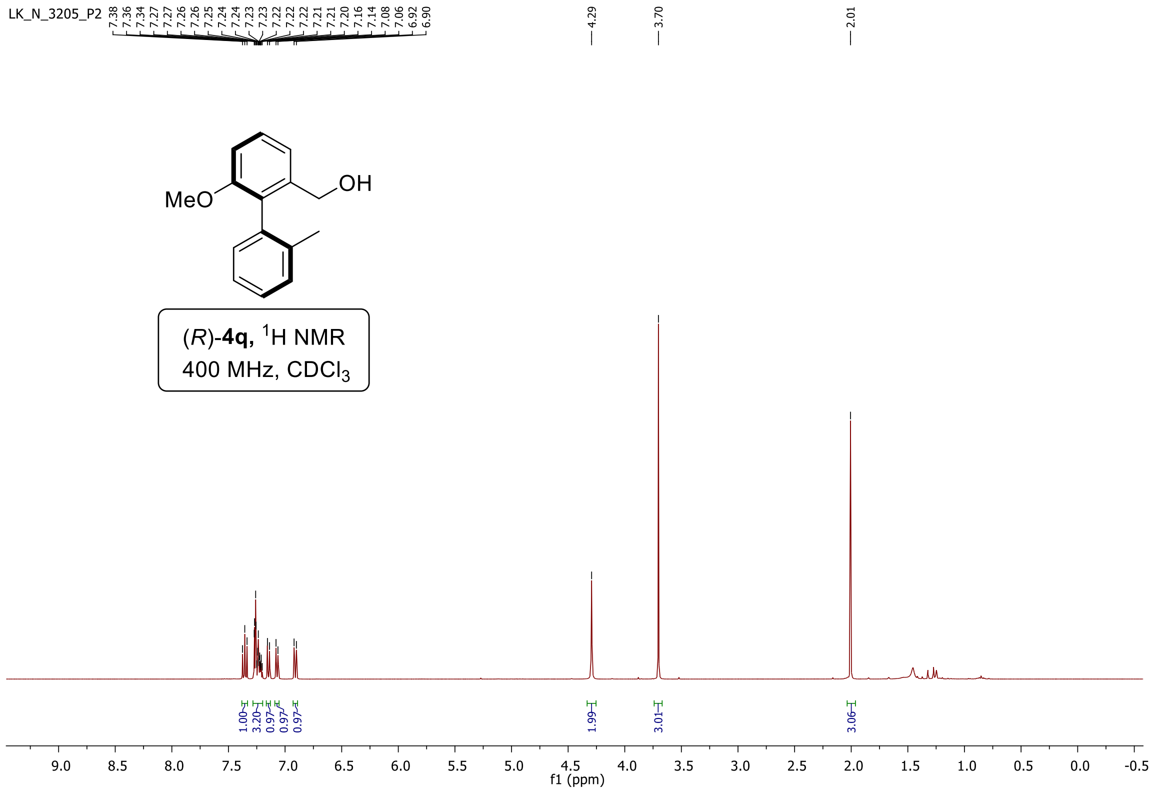

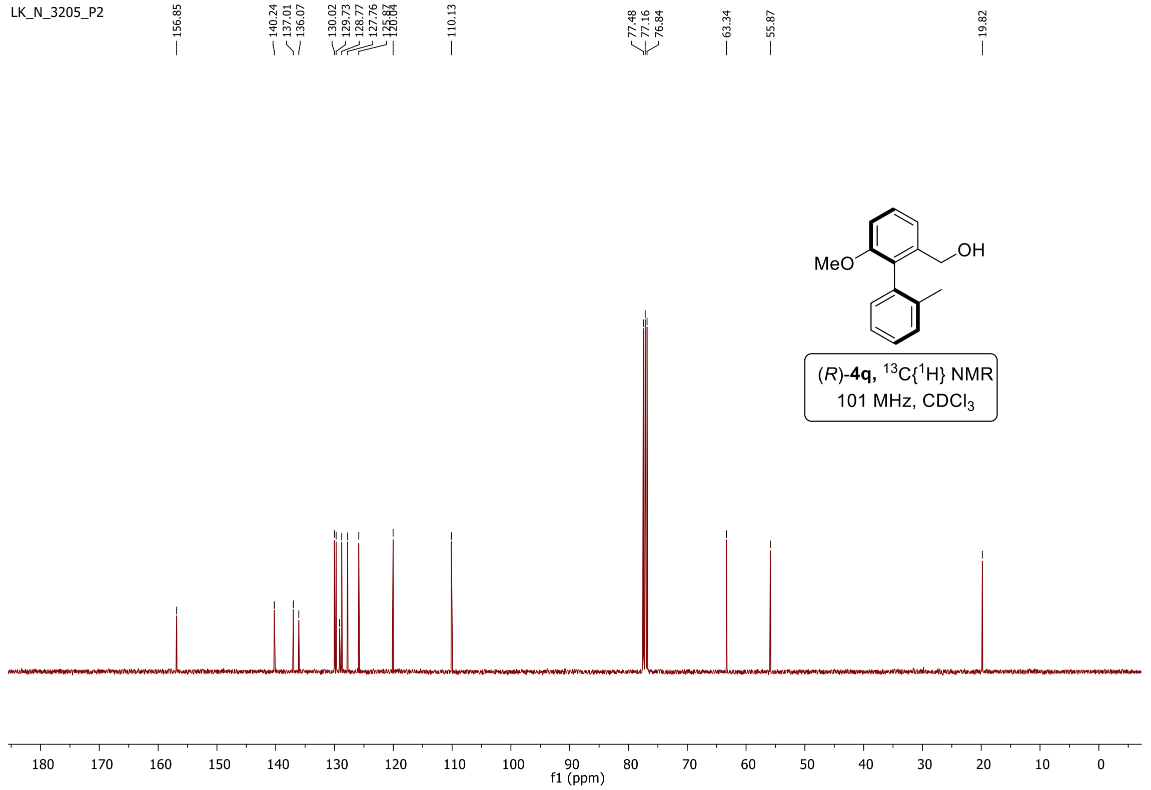

^{1}H and ^{13}C{^{1}H} NMR spectra of (*S*)-**5q**

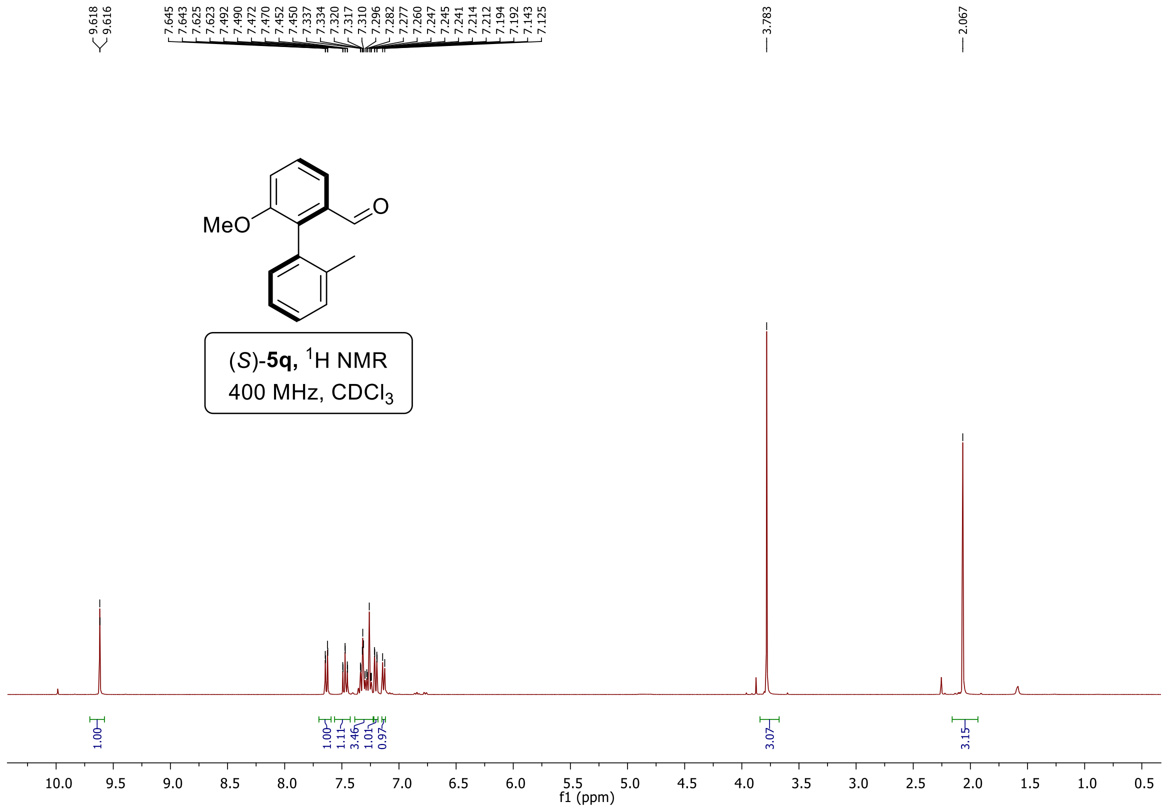

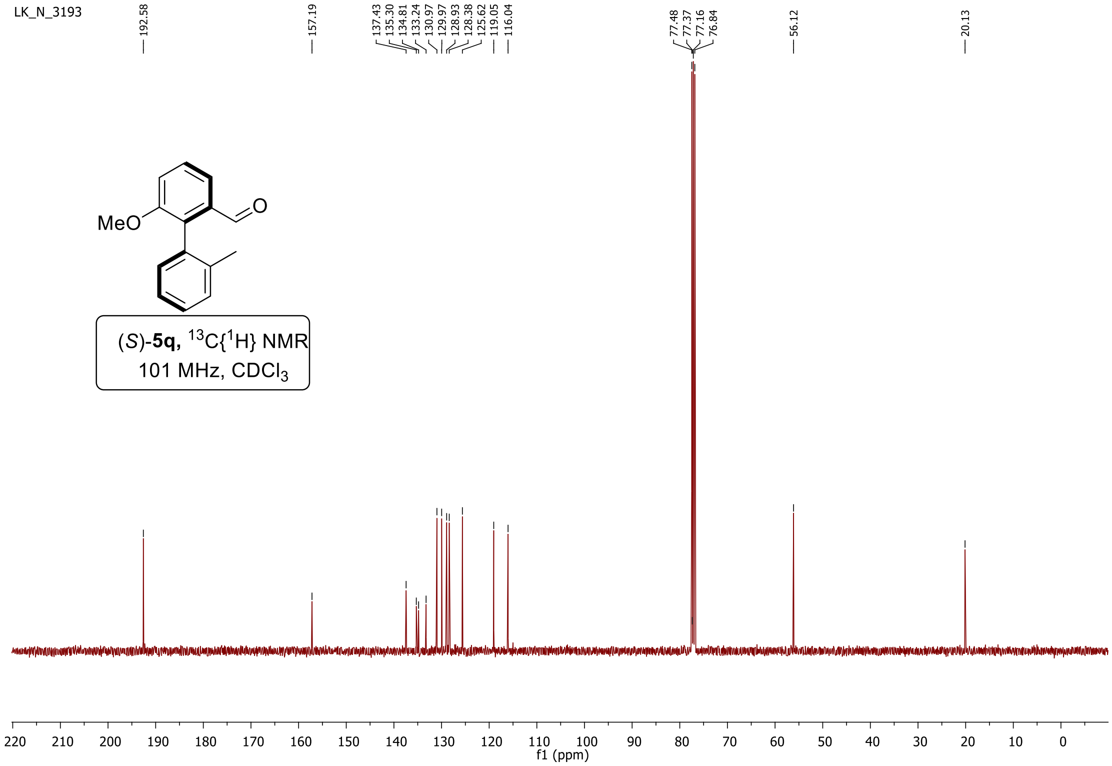

^{1}H and $^{13}C\{^{1}H\}$ NMR spectra of (*R*)-**4r**

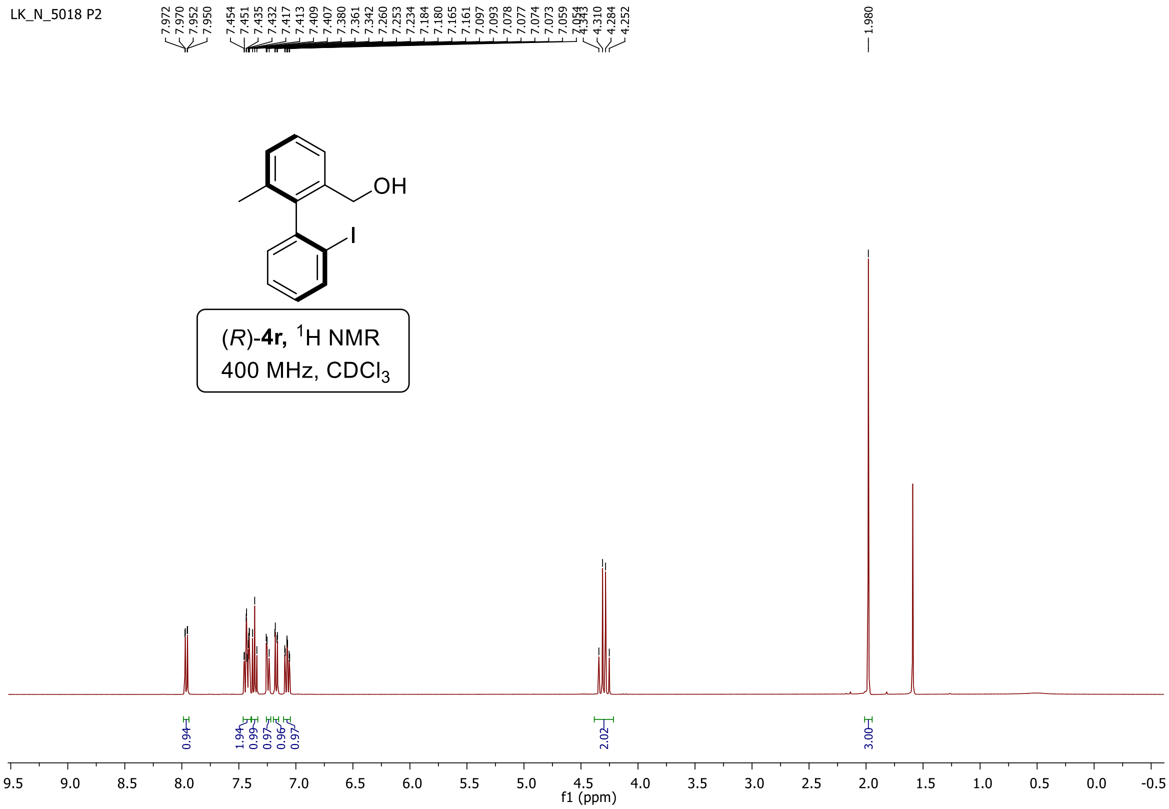

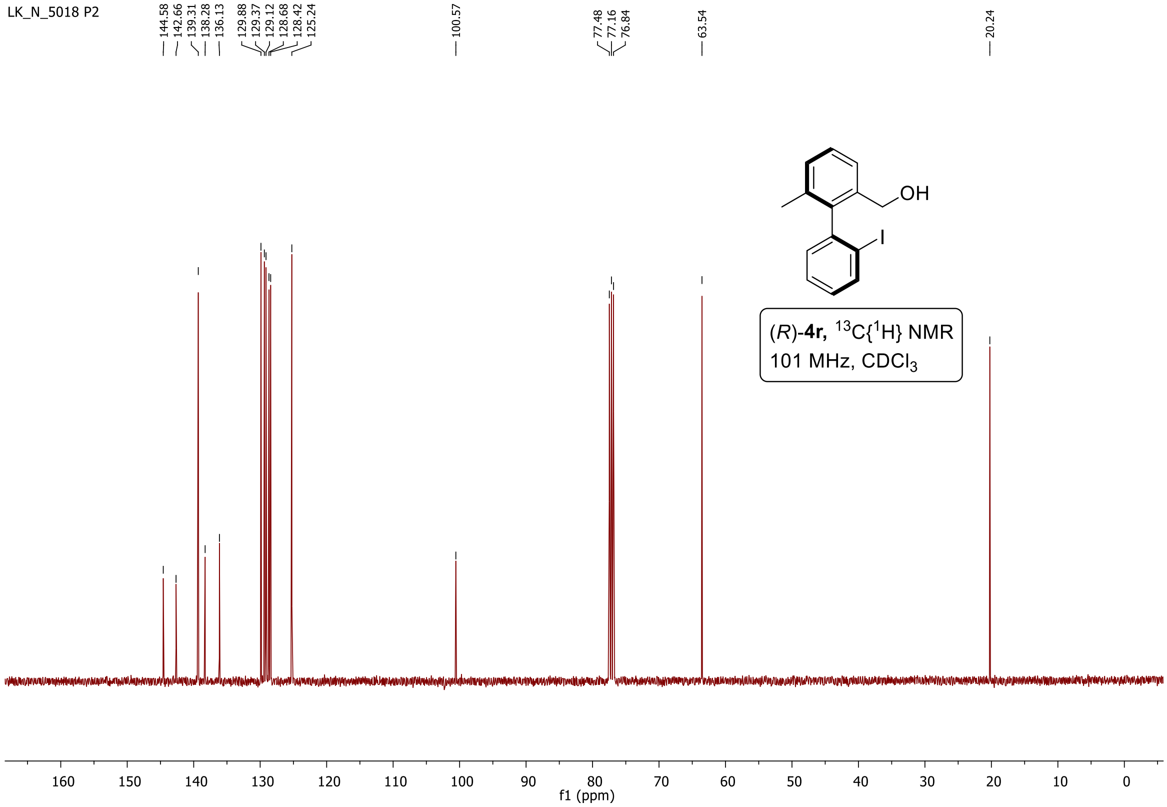

^1H and $^{13}C\{^1H\}$ NMR spectra of (*S*)-**5r**

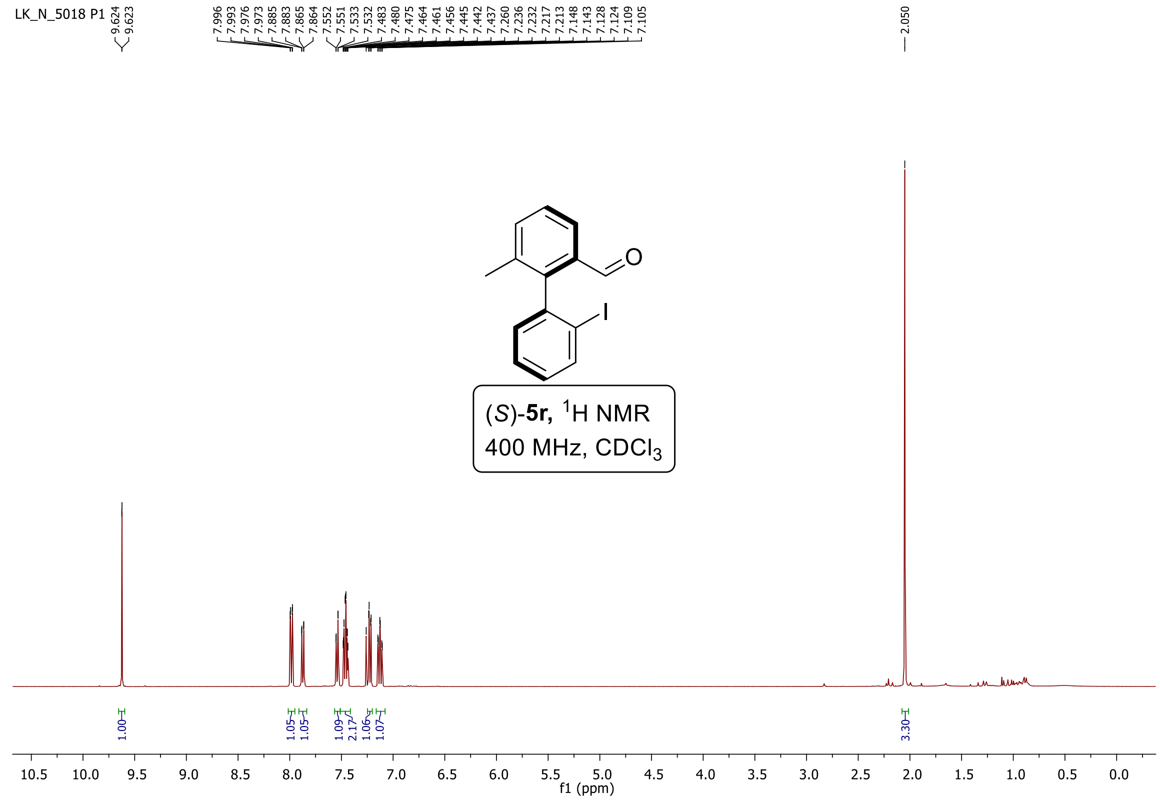

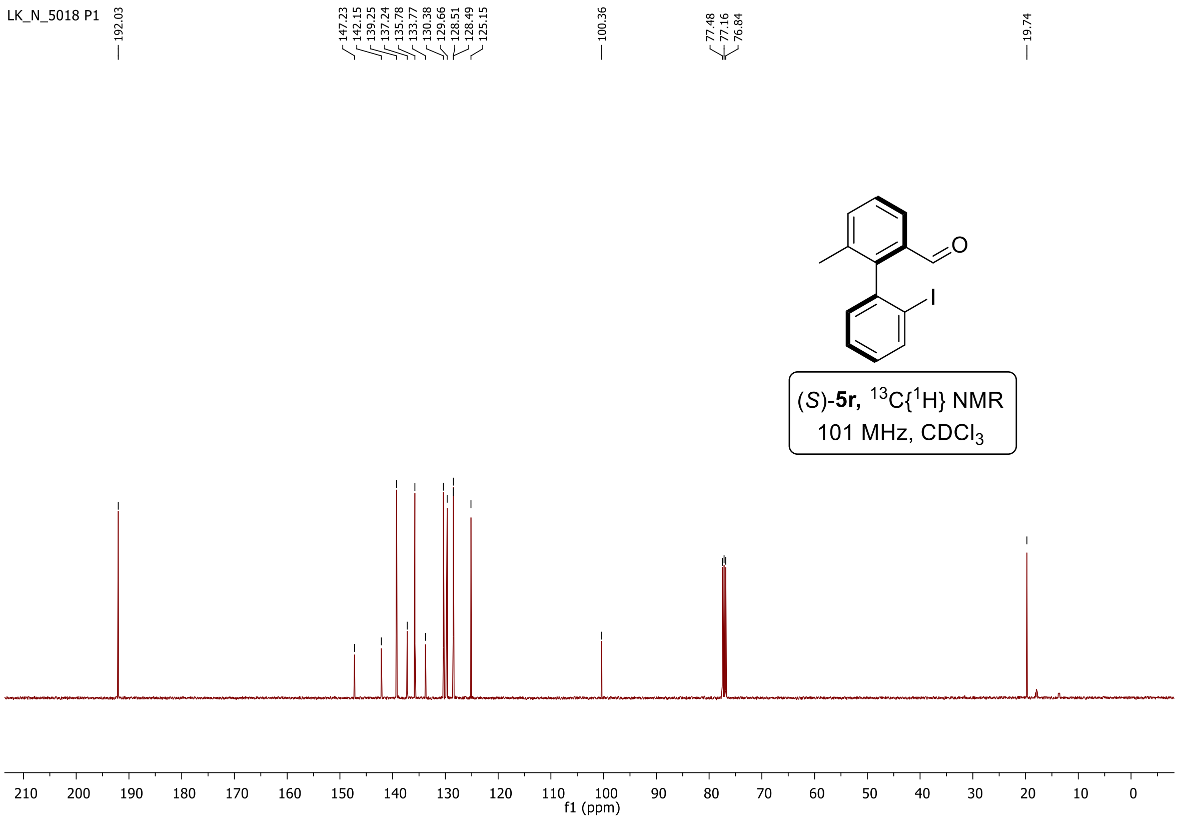

^{1}H ,$^{13}C\{^{1}H\}$ and ^{19}F NMR spectra of optically pure-**7a**

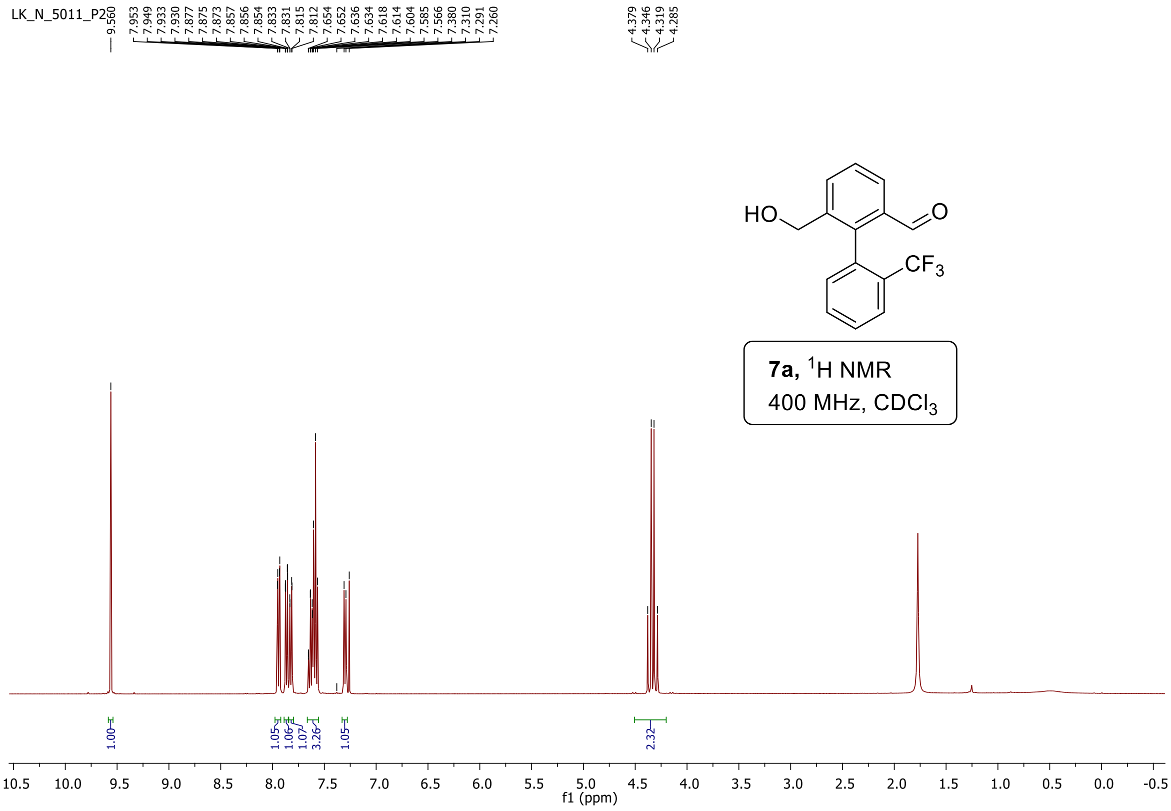

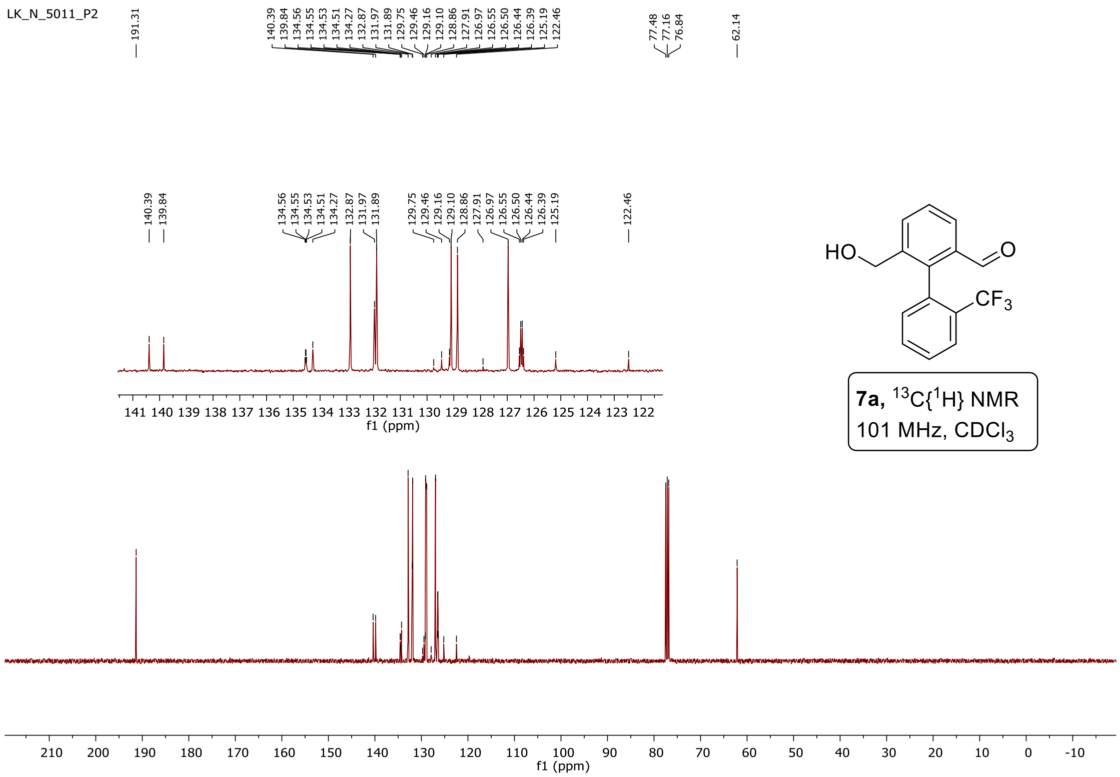

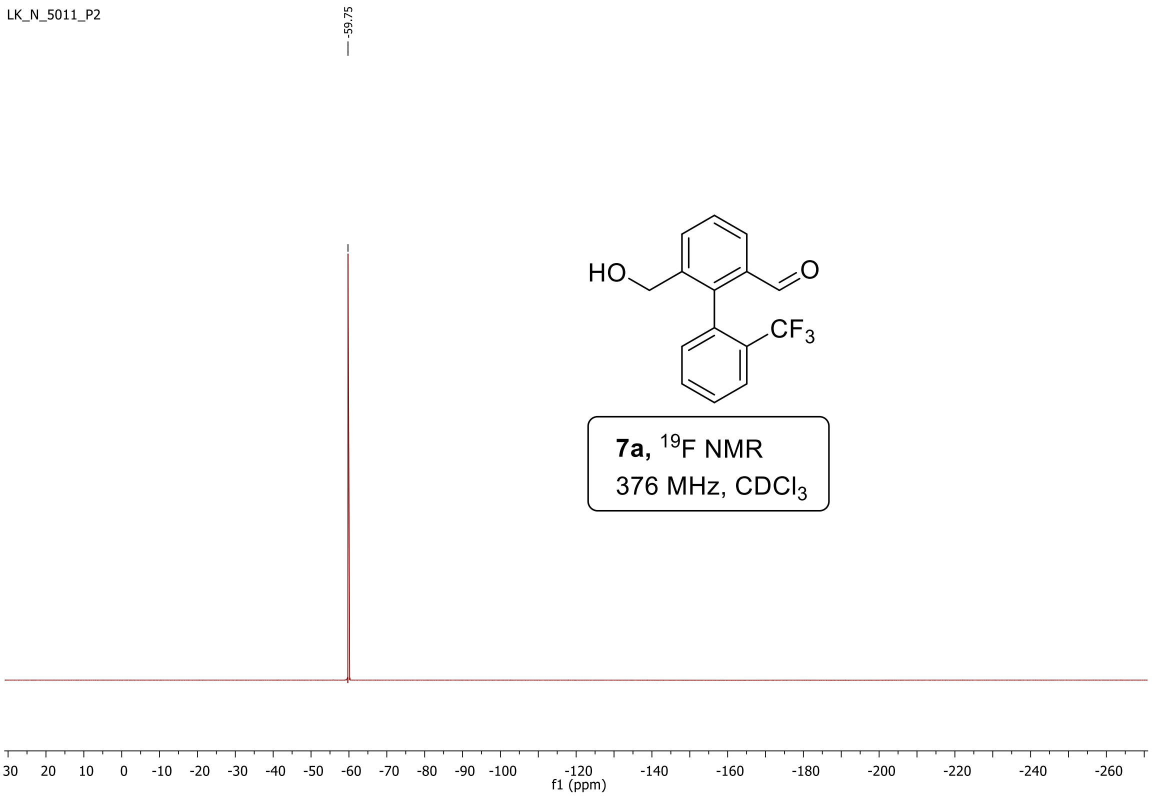

^{1}H and $^{13}C\{^{1}H\}$ NMR spectra of optically pure-**7b**

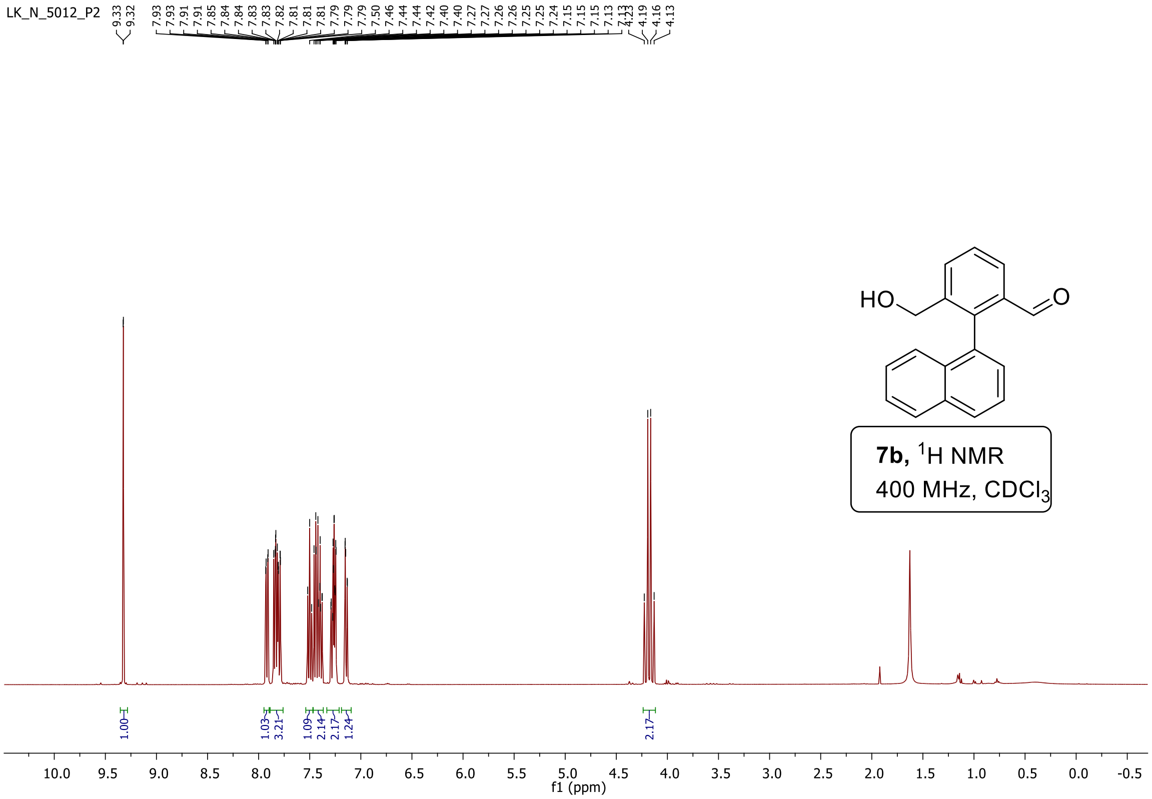

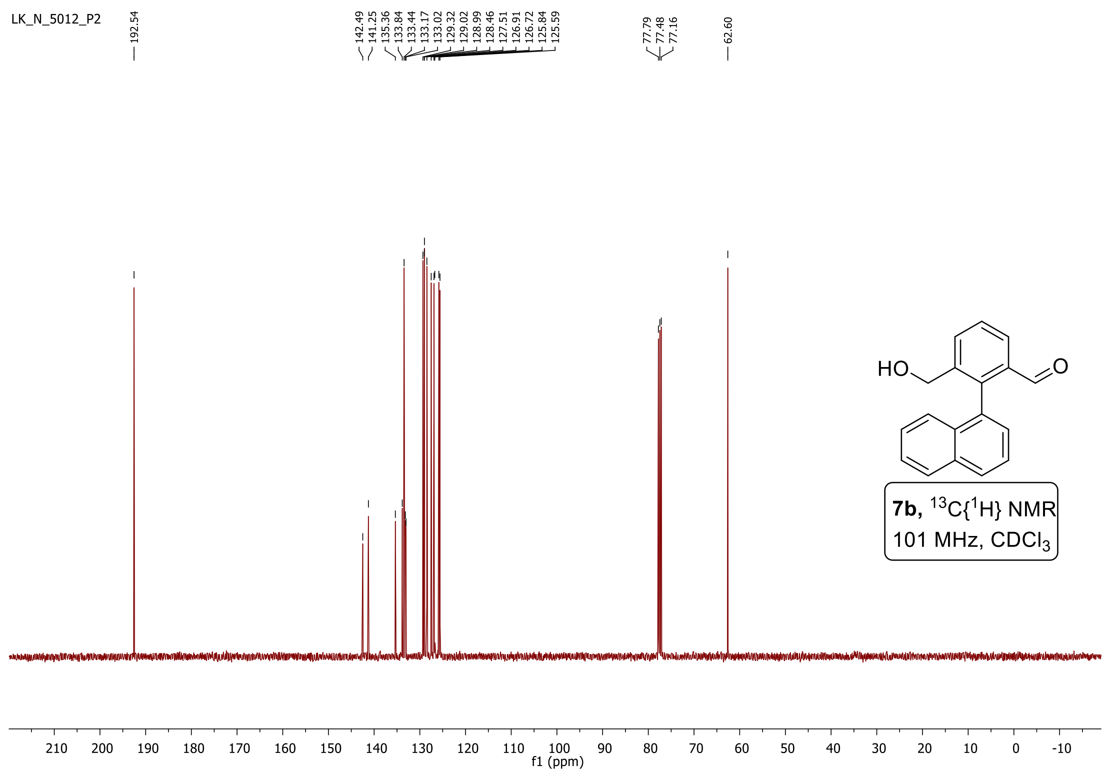

^{1}H and $^{13}C\{^{1}H\}$ NMR spectra of optically pure-**7c**

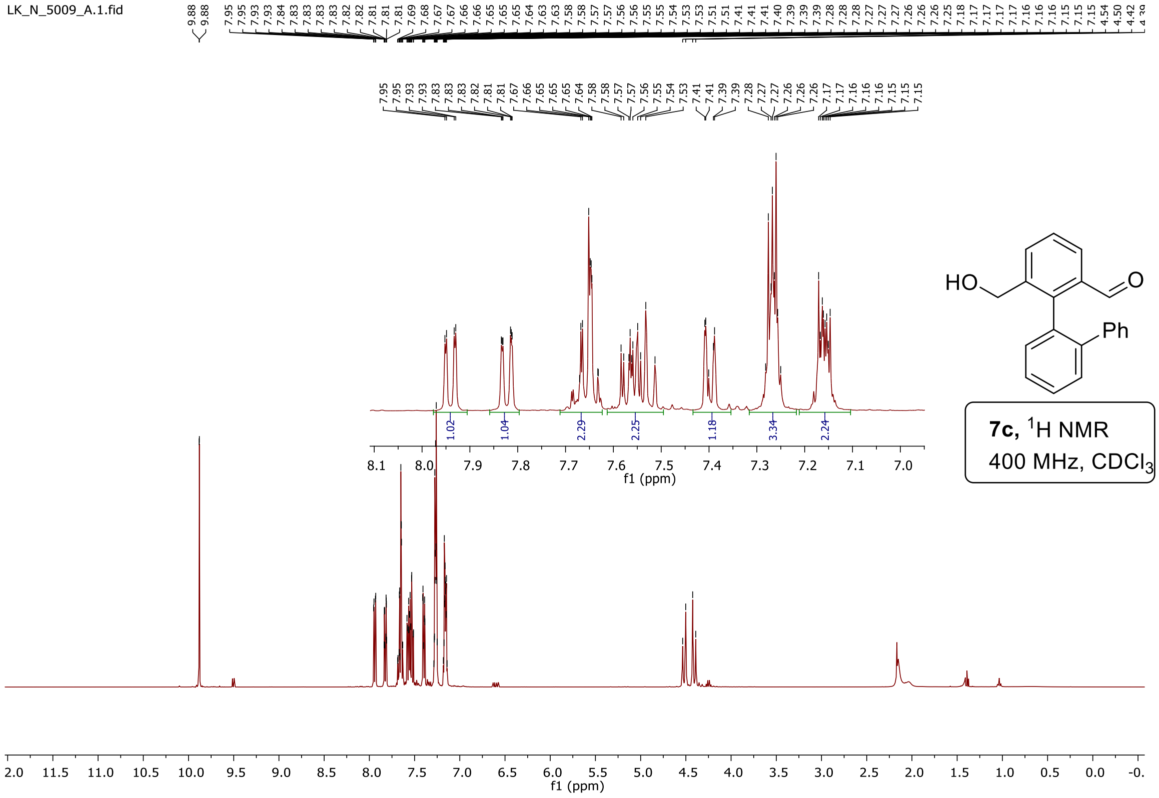

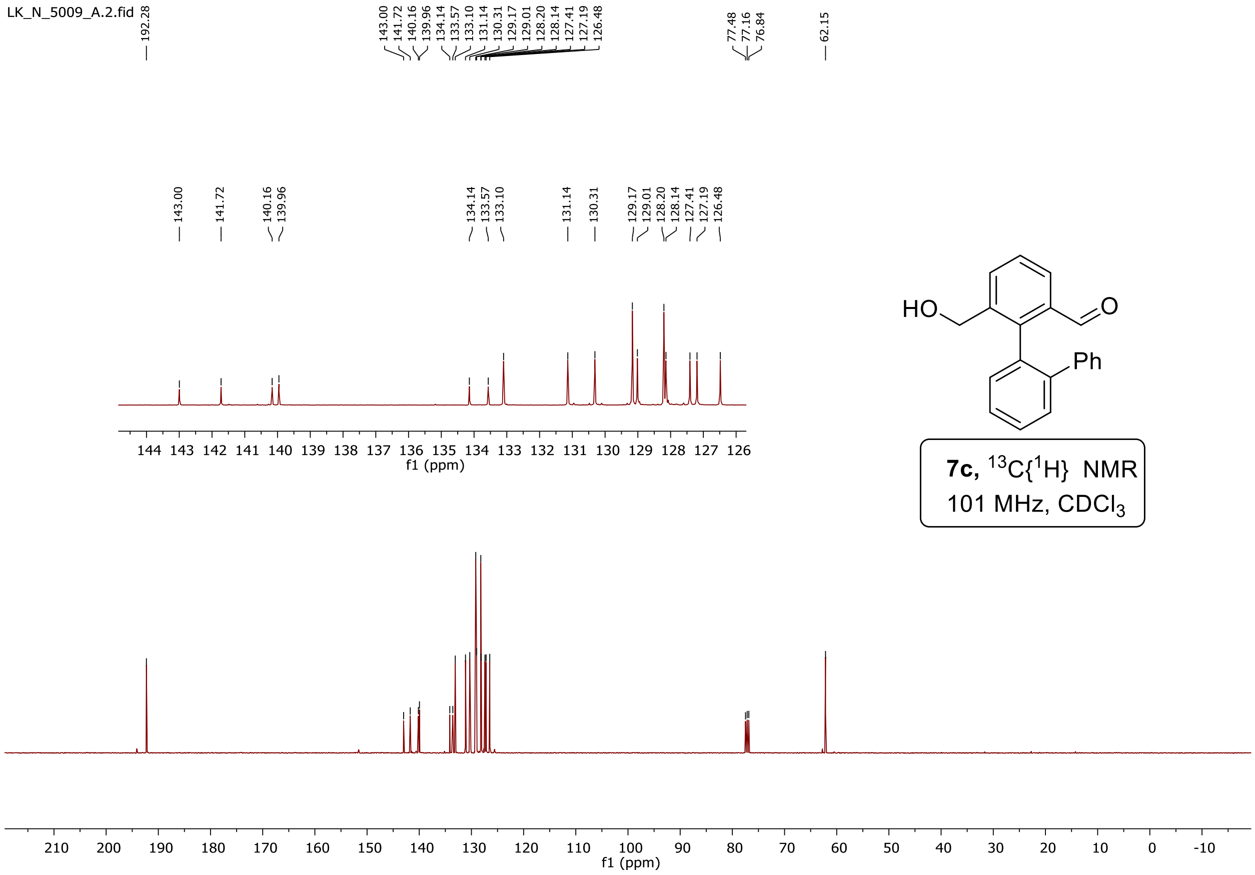
LK_N_5009_A.2.fid
192.28
143.00
141.72
140.16
139.96
134.14
133.57
133.10
131.14
130.31
129.17
129.01
128.20
128.14
127.41
127.19
126.48
77.48
77.16
76.84
62.15
HO
O
Ph
7c, $^{13}C\{^{1}H\}$ NMR
101 MHz, $CDCl_3$
f1 (ppm)
f1 (ppm)
